# Computational Identification of Potential Organocatalysts (CIPOC) Reveals a 2-aminoDMAP/Urea Catalyst Superior to Its Thiourea Analogue

**DOI:** 10.1021/jacs.4c10634

**Published:** 2025-03-12

**Authors:** Sezen Alsancak-Koca, Yeşim Çamlısoy, İrem Bakırcı, Murat Işık, Nihan Çelebi-Ölçüm, Cihangir Tanyeli

**Affiliations:** † Department of Chemical Engineering, Yeditepe University, 34755 İstanbul, Türkiye; ‡ Department of Chemistry, Middle East Technical University, 06800 Ankara, Türkiye; § Department of Food Engineering, Bingöl University, 12000 Bingöl, Türkiye

## Abstract

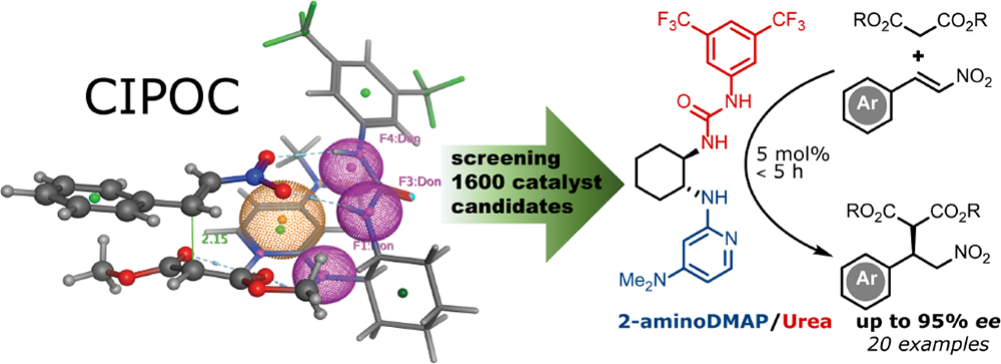

Asymmetric organocatalysis by bifunctional acid- and base-type small organic molecules has emerged as a promising way to enhance stereoselective organic transformations since the beginning of this millennium. Takemoto’s *tert*-amine/thiourea catalyst, an archetype in these endeavors, has encouraged many to design new multifunctional alternatives. However, the discovery of efficient catalysts in a library of thousands of candidates containing the desired functionalities in their structures remains a great challenge both synthetically and computationally. We, toward these ends, developed a computational protocol (CIPOC—Computational Identification of POtential (Organo)Catalysts), which discovered a chiral 2-aminoDMAP/urea catalyst among 1600 multifunctional catalyst candidates enabling conjugate addition of malonates to *trans*-β-nitroalkenes rapidly (in a few hours) with exquisite selectivities and yields, producing superior results than that of Takemoto’s. The unique activity of this chiral 2-aminoDMAP/urea is attributed to the dual function of the 2-aminoDMAP unit (double H-bonding and π-stacking interactions) in addition to the exceptional performance of the urea unit compared to thiourea, as a result of a lower energetic penalty required to distort the catalyst to its active conformation to provide optimal catalytic interactions.

## 1 Introduction

**Figure 1:**
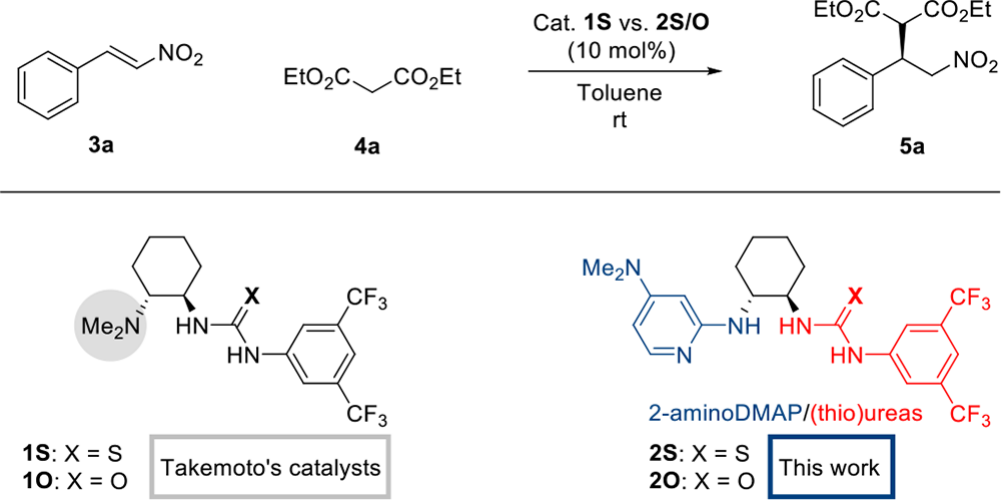
2-AminoDMAP/(thio)ureas described herein and Takemoto’s catalyst.

Asymmetric organocatalysis by bifunctional acid/base type small organic molecules has enjoyed widespread use in stereoselective transformations.^1−3^ The design of such catalysts essentially relies on the appropriate placement of an acid (H-bond donor, HBD) and a base (H-bond acceptor, HBA) together around some stereogenic elements.^4^ The simplicity of this guiding design principle has given inspiration to many hundreds to develop their own bifunctional organocatalysts,^5,6^ yet based largely on experimental trial and error, as two-dimensional structures of these multifunctional organocatalysts do not give clues as to what scaffolds could provide the desired three-dimensional constellations of these functional groups leading to efficient catalysis. An automated tool that can identify potential organocatalyst candidates in a given pool for a target reaction could enormously facilitate an organocatalyst screening process in addition to providing insights into the origins of catalysis and selectivity. Here, we report a computational protocol, CIPOC (Computational Identification of POtential (Organo)Catalysts), that successfully identified a novel chiral 2-amino-DMAP/urea organocatalyst for the reaction of malonates with *trans*-β-nitrostyrene among 1600 multifunctional catalyst candidates, which is experimentally validated to show superior activity than the best-known catalyst (**1S**) for the reaction of interest (Figure 1). The catalyst has a broad substrate scope and is successfully employed for the gram-scale synthesis of pharmaceutically important gamma-aminobutyric acid (GABA) analogues. Besides its success in catalyst discovery, CIPOC revealed that the catalyst distortion is responsible for the unexpected poorer performance of the thiourea derivative compared to urea, and 2-aminoDMAP is a privileged superbase motif, taking advantage of stabilizing double H-bonding and π-stacking interactions.

Much of the interest in the development of organocatalytic strategies in asymmetric synthesis has been centered on HBDs.^7−16^ Although relatively less explored, Bro̷nsted basic HBAs, have been shown to be game-changers in these endeavors.^17^ Guanidines,^18−22^ iminophosphoranes,^23,24,6a,6d^ cyclopropenimines,^25−27^ and amidines^28−32^ are among some influential, emerging organo-superbase^33,34^ motifs developed to this end. Inspired by these organobases, in particular with that of Wulff et al.,^35,36^ we have found 2-aminoDMAP superbase a fruitful precatalyst readily yielding sulfonamides^37^ and squaramides^38^ which powerfully catalyze Michael additions of 1,3-diketones to *trans*-nitroolefins. Besides an earlier metal-based protocol,^39^ the first efficient organocatalytic process for this reaction was made possible by the *tert*-amine/thiourea catalyst (**1S**), a.k.a. Takemoto’s catalyst.^7,40^ Reactions involving **1S** demanded 10 mol % catalyst loadings and day-long reaction times to afford Michael adducts in good yields (86%) and excellent enantioselectivities (93% ee).

Several groups computationally explored the origins of enantioselectivity and the rate-determining step of the reaction between malonates and *trans*-β-nitroalkenes in the presence of Takemoto’s catalyst. Understanding the selectivity and catalytic mechanism strongly depends on one simple question: How does the catalyst use HBD and HBA units for binding the substrates and lowering the activation barrier? In contrast to the binding mode proposed by Takemoto, in which malonate H-bonds with the protonated amine, whereas thiourea stabilizes the nitroolefin, theoretical investigations supported a reversed binding mode, in which the malonate interacts with the thiourea and nitroolefin interacts with the protonated amine.^41−44^ Another alternative pathway in which thiourea moiety orients the two substrates by forming H-bonds with both malonate and nitrostyrene was also proposed, yet calculated to be higher in energy.^43^ Inclusion of the solvation effects identified the stereo-determining C–C bond formation also as the rate-determining step.^41,43^

**Figure 2:**
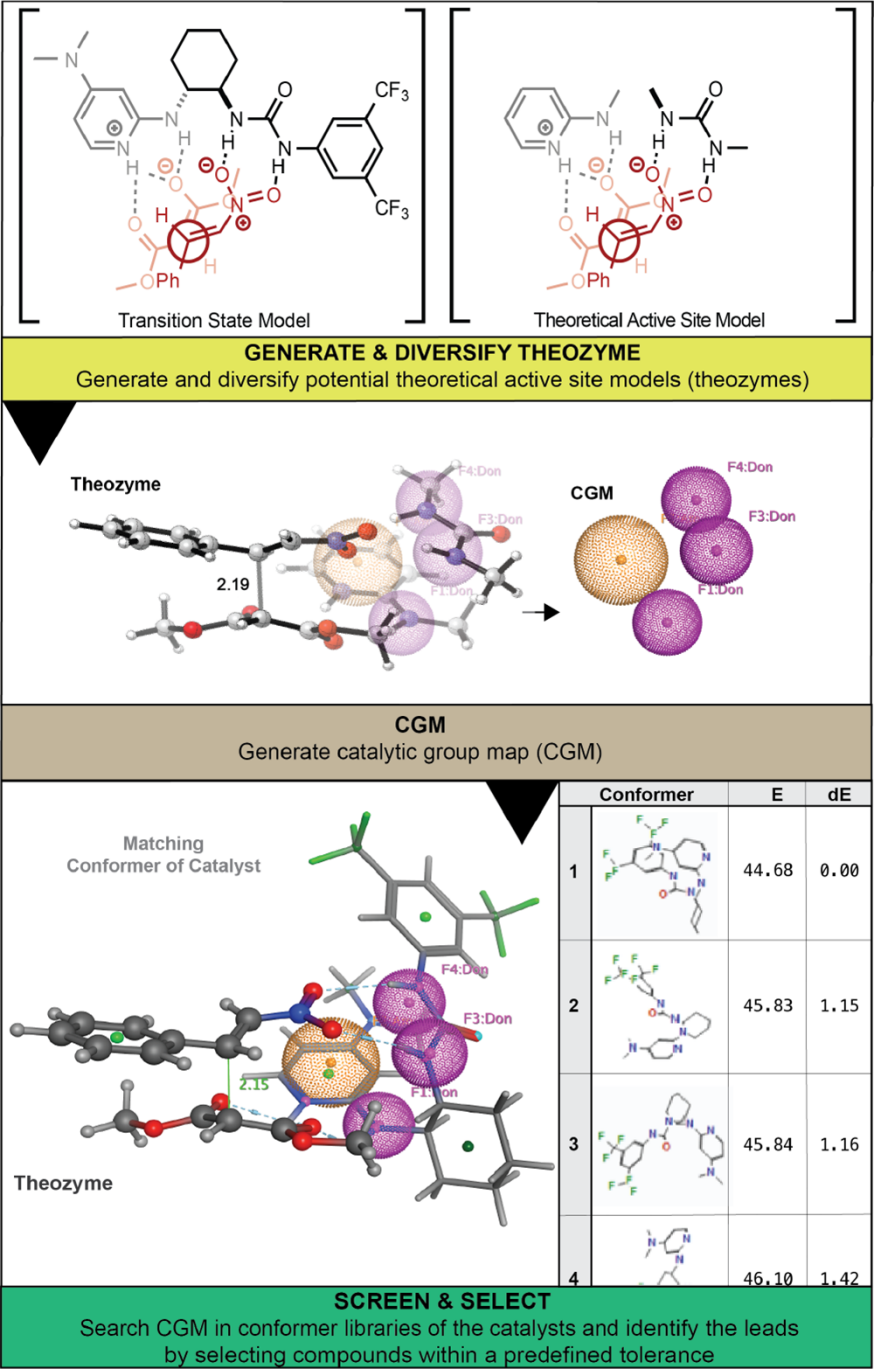
Outline of the CIPOC.

**Figure 3:**
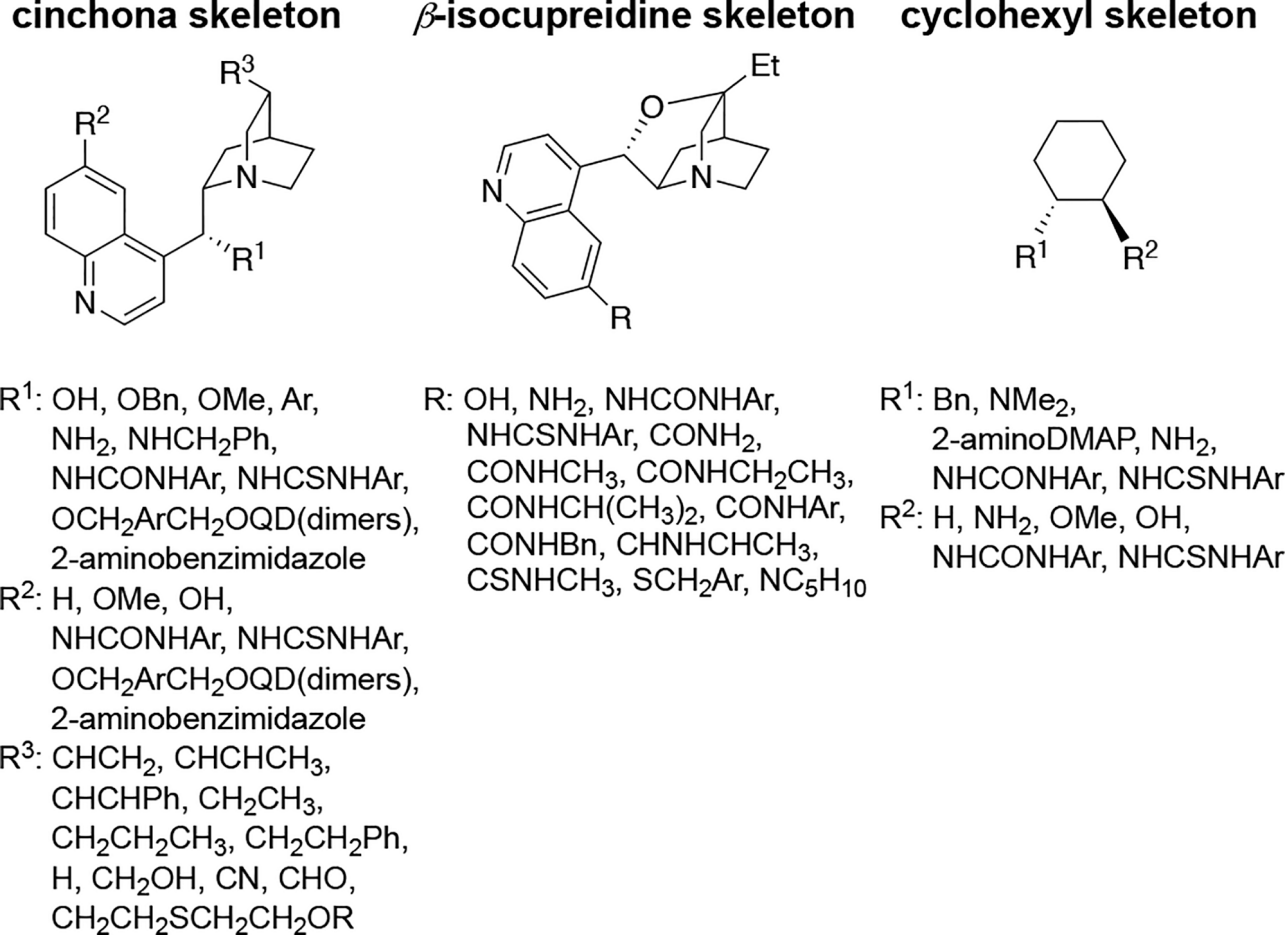
Catalyst database.

In this work, we take the challenge of identifying a catalyst superior to the best-known organocatalyst for a reaction of broad interest when numerous alternative binding patterns in a highly flexible multifunctional catalytic scaffold obscure the preferred pathway and the catalytic outcome of the reaction. We describe a protocol, CIPOC (Figure 2), which allows rigorous exploration of the reaction’s potential energy surface intrinsically taking into account the diversity in the catalytic arrangement to rapidly screen the most promising candidates in an organocatalyst database. For this purpose, we collected about 1600 molecules containing various HBAs and HBDs in their structure centered around 3 well-known chiral scaffolds (Figure 3). CIPOC identified a novel chiral 2-aminoDMAP/thiourea catalyst (**2S**) as being superior to Takemoto’s for the conjugate addition of trans-β-nitrostyrene (**3a**) and diethyl malonate (**4a**). Intriguingly, the urea analogue (**2O**) is predicted to be more active than the thiourea (**2S**). To our delight, experiments show that both thiourea (**2S**) and urea (**2O**) gave comparable yields and enantioselectivities to Takemoto’s catalyst (**1S**) in just a few hours rather than day-long reaction times, with **2O** showing a significantly better catalytic performance than **2S**. 2-Amino-DMAP emerges as an excellent superbase motif that allows substantial stabilization via a combination of double H-bonding and π-stacking interactions. Calculations also explain for the first time that the unusual reactivity of the urea analogue compared to its more acidic thiourea counterpart is a result of the lower energetic penalty paid for distorting **2O** to its active conformation compared to **2S**.

## 2 Methodology

### 2.1 Computational

CIPOC, summarized in Figure 2, was employed to identify novel catalytic structures for the addition reaction of dimethyl malonate to *trans*-β-nitrostyrene. Initially, theoretical active site models (theozymes) including representative functional groups involved in the catalyst library (Figure 3) were generated at the B3LYP-D3/6-311+G(d,p)(toluene) //B3LYP-D3/6-31G(d)(toluene) level to evaluate their catalytic performances and optimal three-dimensional arrangements for maximum transition state (TS) stabilization taking into account different binding modes. Based on the lowest energy theozymes catalytic group maps (CGMs) were generated and diversified followed by their screening in the conformer library of the catalyst database. The final step involves the evaluation of selected hit catalyst candidates using quantum mechanical calculations. The details of each step are described in the following subsections.

#### 2.1.1 Theozyme Generation

Theozymes are quantum mechanically optimized transition state models in the presence of catalytic functional groups. They provide valuable quantitative information on the degree of transition state stabilization and individual contributions of atomic interactions to catalysis.^45^ Theozymes have shed light on the factors that contribute to catalysis for various biotransformations, and constituted the core of “inside-out” approaches for de novo catalyst design that successfully led to computationally designed enzymes^46^ and spiroligozymes.^47^ Here, we use theozymes for the first time as templates for three-dimensional virtual screening of molecular databases to identify promising organocatalyst candidates.

We generated theozymes using different catalytic groups (HBA: trimethylamine/2-aminopyridine, HBD: thiourea/urea) to understand their effect on the activation barrier of the rate-determining C–C bond formation step (see SI, Figure S1.3). The first model consists of *tert*-amine and thiourea groups and is created based on the cinchona/β-isocupredine derivatives and Takemoto’s catalyst, the most efficient organocatalysts identified for the target reaction so far.^40,48−50^ Other model consists of 2-aminopyridine and thiourea groups and is constructed based on studies showing the efficiency of the 2-aminoDMAP superbase motif.^35,37,38^ The generated theozymes were diversified to reflect different binding modes as proposed by Takemoto^40^ (Binding Mode A (**BMA**)) and Pápai^41^ (Binding Mode B (**BMB**)) (see SI, Figure S1.4). Although Izzo proposed an alternative binding mode, it has not been considered in this study as it is shown to be energetically unfavorable.^43^ These theozymes were further diversified to consider the enantiotopic faces (see SI, Figure S1.5). The next step involved the diversification of transition states via rotation around the forming C–C bond, which determines the relative orientations of catalytic functional groups (see SI, Figures S1.6 and S1.7). The diversification resulted in 24 structures for each model (see SI, Figures S1.8 and S1.9); only the lowest energy ones are given and discussed in the text.

#### 2.1.2 Creating Catalytic Group Map (CGM) and Catalophore Query

CGMs were constructed using MOE^51^ with features such as H-bond acceptor and donor, aromatic ring centers, volume constraints, etc. (see SI, Table S1.1), based on the quantum mechanically optimized theozymes. An inclusive catalytic group map is necessary to ensure matching with three different catalyst skeletons and to avoid missing any matching possibilities. CGMs allow further diversification of the catalytic functional groups, for example, the HBD feature may involve all functionalities such as urea, thiourea, alcohol, amine, etc. For screening, a partial matching mode was applied to include all catalysts in our database. Partial matching mode is defined as a match of at least two but not all catalophoric features. Selected features for CGMs (see SI, Table S1.2) were used to build the three-dimensional catalophore models (see SI, Figure S1.10).

#### 2.1.3 Generating Conformer Libraries of Organocatalysts

A conformer library for each catalyst included in the database was generated to identify the catalyst that could provide the catalytic functional groups in the optimal arrangements depicted in the low-energy theozyme. An extensive conformational search was carried out for each explored catalyst with MOE^51^ software with MMFF94x. Stochastic search was employed because it is well suited for exploring the chiral space of organic molecules.^52^ The rejection and iteration limits were used as 1000 to achieve sufficient conformational sampling for an initial assessment in a reasonable amount of computational time. Root mean square deviation (RMSD) of all the conformations less than 0.25 were considered duplicates. All of the catalysts including their conformational libraries were saved in a database (mdb) and later employed in catalophore screening. All the conformations of the matched catalysts were ranked according to their molecular mechanics (MM) energies, of which the lowest 20 in addition to the matching active conformers were further optimized by using density functional theory for full evaluation.

#### 2.1.4 Catalophore Search

The catalyst conformer library was screened against the CGM to identify the conformers that could satisfy a theozyme-like arrangement of catalytic functional groups. Partial matches were allowed to obtain all possible catalytic candidates. The resulting hits were primarily sorted by their matching scores and deviations from their optimum catalytic geometries followed by quantum mechanical evaluation of selected leads (see the SI for ranking and selection criteria).

#### 2.1.5 Quantum Mechanical (QM) Evaluation

Replacing the model catalytic groups of the corresponding theozyme with the matching conformer of the catalyst gives the transition structure in the presence of the full chiral multifunctional catalyst (SI, Figure S1.11). QM calculations were used to evaluate the activities and selectivities of the full catalytic systems. All calculations were carried out with Gaussian 16^53^ at B3LYP-D3/6-311+G(d,p)(toluene)//B3LYP-D3/6-31G(d)(toluene) level. The success of B3LYP/6-31G(d)^54−57^ in explaining the experimental trends in reactivity and selectivity for many organocatalyzed transformations,^58^ and in reproducing theoretical models of naturally occurring enzyme active sites,^59,60^ allowed us to use this standard and cost-effective methodology to identify the placement of catalytic groups targeted in organocatalysis. Due to the presence of aromatic motifs, however, where dispersion interactions are particularly important, a dispersion correction energy was necessary to better account for H-bonding and π–π interactions,^61,62^ and the molecular electronic energies were determined using dispersion-corrected density functional theory (DFT). This involved combining self-consistent-field (SCF) energy obtained from KS-DFT calculations with the standard atom pairwise London dispersion energy derived from the D3 theory.^63^ The intrinsic reaction coordinate (IRC)^64,65^ method was used to determine minimum energy paths from the transition states to verify the reactants and the products. Since the system being studied contains zwitterionic intermediates, the effect of the solvent was taken into account both in geometry optimization and in the calculation of single-point energies. Solvent effects were considered with the SMD model^66^ as implemented in Gaussian 16. Toluene was used as the solvent to represent the experimental conditions. Frequency calculations were used to verify all stationary points as minima or saddle points and to calculate thermal corrections at 298.15 K. The energy corrections to enthalpy and free energy, which accounted for zero-point vibrational energies, were applied to the molecular electronic energies. The calculations were performed at 298.15 K by using the harmonic oscillator approximation. Truhlar’s quasi-harmonic correction was utilized, and all frequencies below 100 cm^–1^ were adjusted to 100 cm^–1^ to minimize errors in entropy estimation associated with low-frequency vibrational modes.^67^ All energies reported throughout the text are given in terms of Gibbs free energies with quasiharmonic corrections in kcal mol^–1^. All distances are given in Å. Enantiomeric excess (% ee) was determined by the Boltzmann distribution of transition states leading to each enantiomer. Only the lowest energy catalyst and analogous systems for comparative purposes are discussed in the text. Other identified matches from different catalytic scaffolds are given in the SI.

### 2.2 Experimental Section

#### 2.2.1 General Procedure for the Syntheses of 2-AminoDMAP/(thio)urea Catalysts

To a Na/benzophenone-dried THF solution (1 mL) of (*R*,*R*)-configurated 2-aminoDMAP^37^ (47 mg, 0.2 mmol) was added either of 3,5-bis(trifluoromethyl)phenyl isothiocyanate (54 mg, 37 μL, 0.2 mmol; for **2S**) or 3,5-bis(trifluoromethyl)phenyl isocyanate (51 mg, 35 μL, 0.2 mmol; for **2O**) dropwise in 1 min at 0 °C under an argon atmosphere. The resulting solution was allowed to reach room temperature and was further stirred overnight at this temperature. The reaction mixture was then directly loaded onto a silica gel column and eluted with DCM which was saturated with ammonia to afford both 2-aminoDMAP/(thio)ureas **2S** and **2O** in 90% chemical yield as off-white amorphous solids.

#### 2.2.2 General Procedure for Asymmetric Organocatalytic Michael Addition

To a stirred solution of *trans*-β-nitroalkenes, **3a**–**r** (0.2 mmol) and 2-aminoDMAP/urea **2O** (4.89 mg, 5 mol %, 0.01 mmol) in toluene (1 mL) was added malonate derivatives **4a–d** (0.4 mmol, 2 equiv) at room temperature under an argon atmosphere. Stirring was continued until the consumption of the limiting reagent, *trans*-β-nitroalkenes (monitored by TLC). The reaction mixture was then directly subjected to silica gel flash column chromatography using EtOAc/*n*-hexanes mixtures as an eluant to afford the conjugate addition products **4a**–**r**.

## 3 Results and Discussion

**Figure 4:**
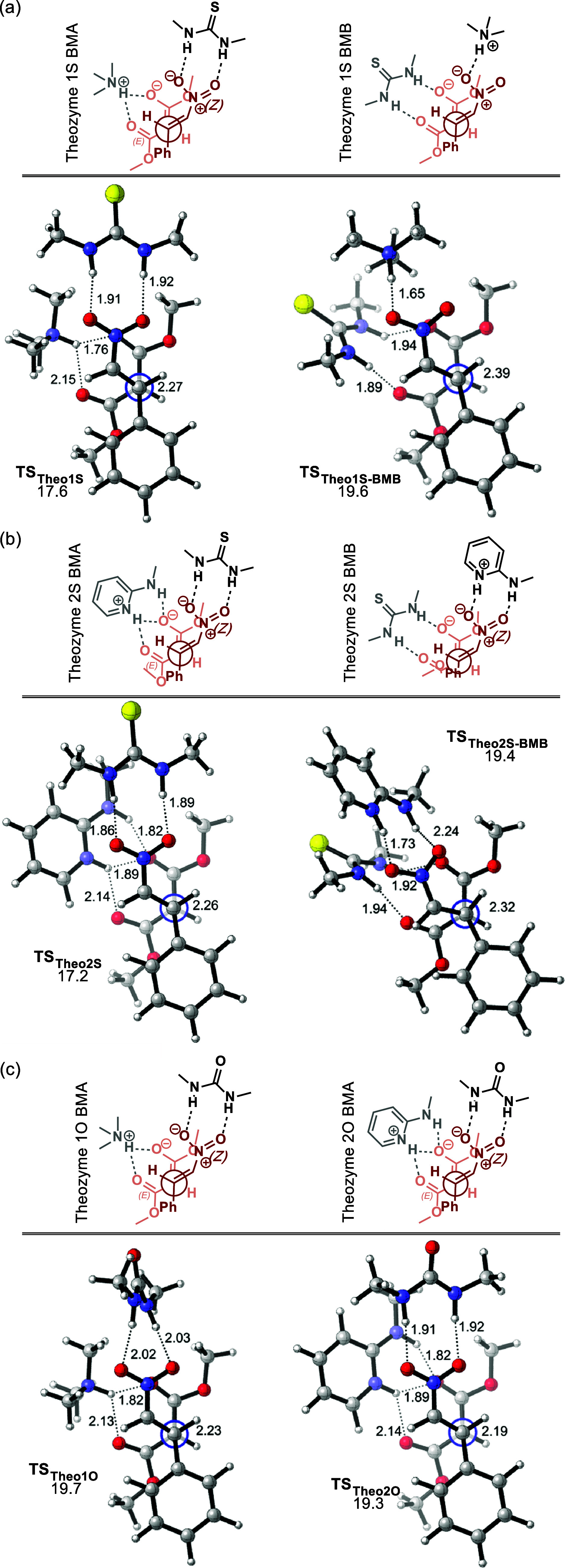
Optimized geometries and activation free energies (relative to the starting materials in kcal mol^–1^) of theozymes for (a) **1S**, (b) **2S**, (c) **1O**, and **2O** (B3LYP-D3/6-311+G(d,p)(toluene)//B3LYP-D3/6-31G(d)(toluene)).

The catalytic cycle starts with the abstraction of the acidic hydrogen of the malonate by the basic moiety of the catalyst, activating it as a nucleophile followed by its conjugate addition to *trans*-β-nitrostyrene forming an intermediate. In this latter rate and stereo-determining C–C coupling step,^43^ Takemoto suggests that while the protonated basic moiety of the bifunctional catalyst stabilizes the negatively charged malonate, the acidic part of the catalyst binds and orients the nitroolefin (**BMA**).^40^ Pápai, on the other hand, proposes a reversed binding mode (**BMB**) for the basic and acidic moieties of the catalyst.^41^ In the final step, the transfer of a proton from the protonated catalyst to the intermediate forms the Michael adduct and regenerates the catalyst. We generated theozymes using different functional groups to understand their effect on the activation barrier of the rate-determining C–C bond formation step. Figure 4 shows the transition state geometries of the corresponding theozymes and their activation energies relative to those of the starting materials. **Theo1S** consists of *tert-*amine (mono HBA) and thiourea groups (double HBD), to particularly represent the functional groups involved in the cinchona/β-isocupredine derivatives and Takemoto’s catalyst. **Theo2S** consists of 2-aminopyridine (double HBA) and thiourea groups (double HBD), generated upon the success of 2-aminoDMAP as a superbase motif. **TS**_**Theo2S**_ is 0.4 kcal mol^–1^ lower in energy than **TS**_**Theo1S**_, suggesting that double H-bonding is only slightly favored over a single H-bonding interaction. **TS**_**Theo2O**_, which represents the urea analogue, is found to be 2.1 kcal mol^–1^ higher in energy than **TS**_**Theo2S**_. Even though both theozymes include double H-bonding, the thiourea group provides better TS stabilization than urea. These results are in line with the higher acidity of thiourea compared to urea (p*K*_a_ = 21.1 vs 26.9, respectively, in DMSO)^68,69^ indicating its better H-bonding ability. Similarly, **TS**_**Theo1O**_ is 2.1 kcal mol^–1^ higher in energy than **TS**_**Theo1S**_. We also considered the binding mode as proposed by Pápai for the lowest energy theozymes. **TS**_**Theo1S-BMB**_ and **TS**_**Theo2S-BMB**,_ are both found to be 2.2 kcal mol^–1^ higher in energy than their corresponding theozymes displaying the binding mode as originally proposed by Takemoto and co-workers (**BMA**). These results agree with a stronger stabilization between the positively charged basic moiety of the catalyst and negatively charged malonate favoring binding mode **BMA**.

**Figure 5:**
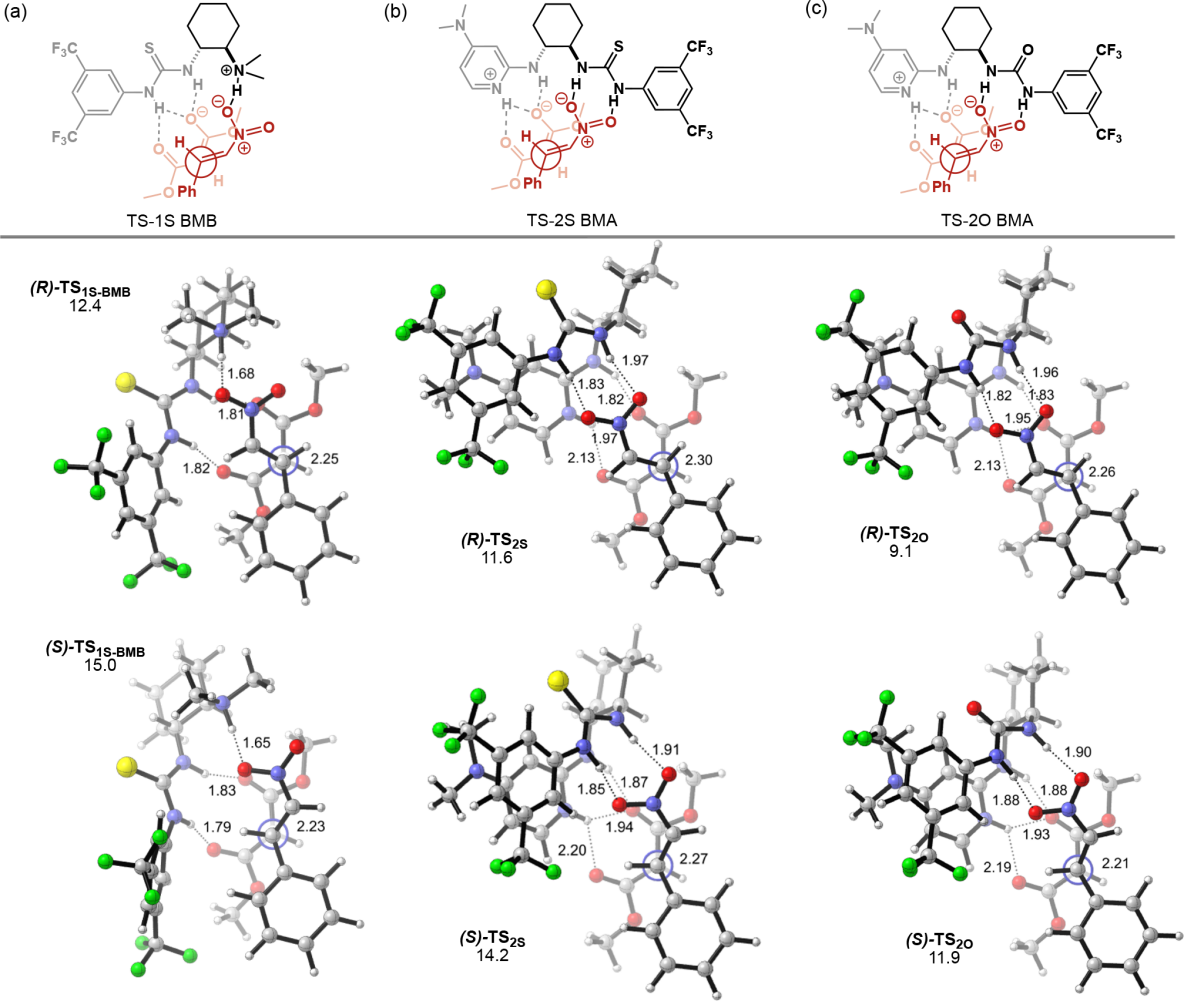
Optimized geometries of the lowest energy enantiomeric transition states and their activation free energies (relative to the starting materials in kcal mol^–1^) in the presence of (a) **1S**, (b) **2S**, and (c) **2O** (B3LYP-D3/6-311+G(d,p)(toluene)//B3LYP-D3/6-31G)(d)(toluene)).

The catalyst database containing about 1600 candidates based on 3 privileged chiral scaffolds^70^ (Figure 3) was screened against the CGMs to discover effective catalysts with exquisite stereocontrol for the target reaction. The most promising catalyst candidates with maximum catalytic group match, avoiding the steric clash between substrates and catalysts, are evaluated using QM. The lowest energy enantiomeric transition structures for full catalysts and their Gibbs free energies relative to separated reactants are given in Figure 5.

The lowest energy TSs for all models lead to the (*R*) enantiomer. The lowest energy TS for the C–C coupling step in the presence of **1S**, our reference catalyst, is **(*R*)-TS**_**1S-BMB**_ and has an activation barrier of 12.4 kcal mol^–1^, 2.6 kcal mol^–1^ lower in energy than its enantiomeric TS (Figure 5a). This structure displays a **BMB** binding mode in agreement with previous reports,^41,43^ while the corresponding TS for **BMA** binding mode (**(*****R*****)-TS**_**1S-BMA**_) is found to be 10 kcal mol^–1^ higher in energy (see SI, Figure S1.13). The most promising candidate, 2-aminoDMAP/urea derivative **(*R*)-TS**_**2O**_**BMA**, benefits from stabilizing double H-bonding and π-stacking interactions compared to those of **1S** and has an activation energy of 9.1 kcal mol^–1^ (Figure 5c). The enantiomeric TS, **(*S*)-TS**_**2O**_, is 2.8 kcal mol^–1^ higher in energy leading to a calculated 98.2% ee. Intriguingly, even though the thiourea analogue was predicted to be more stable by 2.1 kcal mol^–1^ compared to urea in model calculations (Figure 4b,c), **(*****R*****)-TS**_**2S**_ is disfavored by 2.5 kcal mol^–1^ in the presence of full catalyst compared to **(*R*)-TS**_**2O**_ with an activation free energy of 11.6 kcal mol^–1^ (Figure 5b,c). The calculations suggest a slightly lower enantioselectivity for **2S** compared to **2O**, with **(*R*)-TS**_**2S**_ being 2.6 kcal mol^–1^ lower in energy than **(*S*)-TS**_**2S**_. **(*R*)-TS**_**2S**_ also shows stabilizing double H-bonding and π-stacking interactions including identical H-bond distances with **(*R*)-TS**_**2O**_. Even though it is not straightforward to quantify the strength of H-bonds and comment on their strength based solely on distances, urea and thiourea displaying identical H-bond distances in **(*R*)-TS_2O_** and **(*R*)*-*TS_2S_**, respectively, are unexpected considering the difference in their acidities. Their corresponding theozymes, however, are free from the strain imposed by the chiral scaffold on the catalytic functional groups and show the expected H-bonding scheme: H-bond distances between thiourea and nitro groups in **TS**_**Theo2S**_ are 0.3–0.5 Å shorter than those between urea and nitro groups in **TS**_**Theo2O**_, indicative of a stronger interaction as anticipated from thiourea’s more acidic character. The inclusion of the chiral scaffold results in a rather skewed interaction with the distal −NH–O distance being 0.14 Å longer than the proximal one. These results suggest the effect of strain imposed by the chiral linker on the H-bonding interactions displayed in the full TSs. In contrast to **1S**, the lowest energy TS for **2S BMB** binding mode, **(*R*)-TS**_**2S-BMB**_ is 4.5 kcal mol^–1^ higher in energy than **(*R*)-TS_2S_**, suggesting that double H-bonding by 2-aminoDMAP favors **BMA** pathway over **BMB** (see SI, Figure S1.13).

Experimental evaluation of promising catalyst candidates identified by CIPOC (**2S** and **2O**) under identical reaction conditions to Takemoto’s^40^ (10 mol % catalyst loading at room temperature) shows an excellent agreement with the computationally predicted trends in activities and selectivities (Table 1, entries 1 and 2). In the conjugate additions of *trans*-β-nitrostyrene (**3a**) and diethyl malonate (**4a**), **2S** and **2O** acted two- to 8-fold faster (80% yield, 75% ee in 12 h, and 89% yield, 91% ee in 3 h, respectively) than **1S**; and to our delight, the urea catalyst (**2O**) gave complete conversion in a few hours affording distinctively higher enantioselectivity and yield than **2S**, comparable to those of **1S**.

**Table 1:**
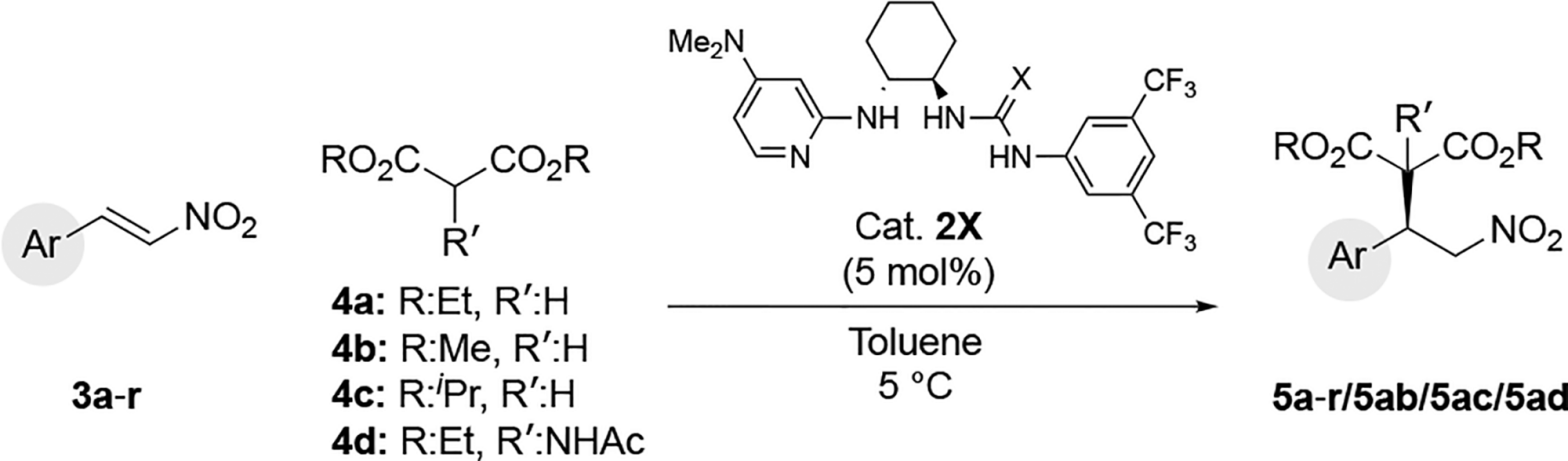
Scope of Nitroolefins^a^

entry	cat.	Ar	adduct	time^b^ (h)	yield^c^ (%)	ee^d^ (%)
1^f^	**2S**	Ph	**5a**	12	80	75
2^f^	**2O**	Ph	**5a**	3	89	91
3	**2O**	Ph	**5a**	4	91	94
4	**2O**	2-NO_2_-C_6_H_4_	**5b**	3	65	80
5	**2O**	4-CH_3_-C_6_H_4_	**5c**	9	90	91
6	**2O**	2-Cl-C_6_H_4_	**5d**	2	95	90
7	**2O**	3-Cl-C_6_H_4_	**5e**	3	82	88
8	**2O**	4-Cl-C_6_H_4_	**5f**	3	81	90
9	**2O**	2,4-(Cl)_2_-C_6_H_4_	**5g**	3	83	92
10	**2O**	2-MeO-C_6_H_4_	**5h**	7	93	92
11	**2O**	3-MeO-C_6_H_4_	**5i**	4	84	88
12	**2O**	4-MeO-C_6_H_4_	**5j**	6	88	94
13	**2O**	3-Br-C_6_H_4_	**5k**	4	76	87
14	**2O**	4-Br-C_6_H_4_	**5l**	4	84	92
15	**2O**	2-F-C_6_H_4_	**5m**	4	91	93
16	**2O**	4-F-C_6_H_4_	**5n**	3	88	94
17	**2O**	2-furyl	**5o**	4	84	91
18	**2O**	2-thienyl	**5p**	7	75	88
19	**2O**	4-BnO-C_6_H_4_	**5r**	10	83	95
20	**2O**	Ph	**5ab**	4	88	92
21	**2O**	Ph	**5ac**	5	93	85
22	**2O**	Ph	**5ad**	24	93	66

a Reactions were run in 0.2 M concentration of *trans*-β-nitroalkene **3a–r** (0.2 mmol) with 2 equiv of dialkyl malonate (**4a–d**).

b Time for complete conversion.

c Isolated yields.

d Enantiomeric excess (ee) determined by HPLC analysis on a chiral stationary phase.

f Reactions were run using a 10 mol % catalyst loading at room temperature.

**Figure 6:**
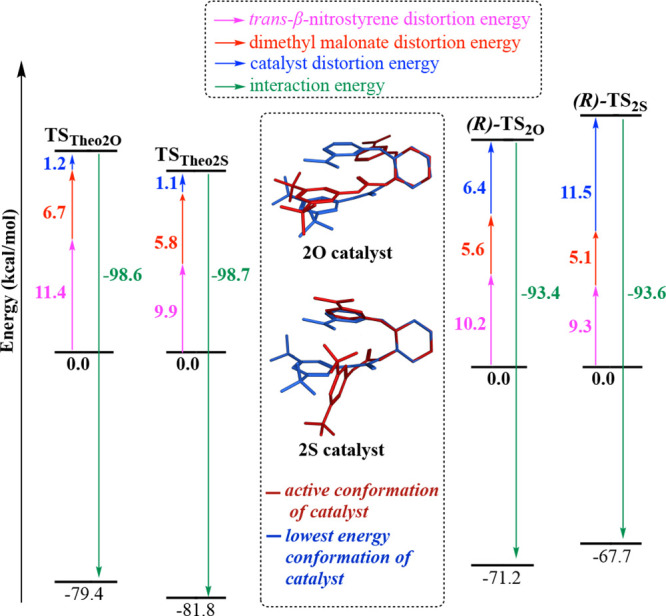
Distortion/interaction analysis of **(*R*)-TS_2O_** and **(*R*)-TS**_**2S**_ and their theozymes **TS**_**Theo2O**_ and **TS_Theo2S_** (B3LYP-D3/6-311+G(d,p)(toluene)//B3LYP-D3/6-31G(d)(toluene)).

It is unusual but not unique for catalysts based on urea to exhibit superior performance compared to their thiourea analogues.^71,72^ To better understand this unexpected result, as well as the energy difference between **(*R*)-TS**_**2S**_ and **(*R*)-TS_2O_** despite essentially the same stabilizing interactions, a distortion/interaction analysis^73^ was performed. Figure 6 shows distortion/interaction analysis of the full catalysts and model systems involving urea and thiourea. In the model system, independently treated catalytic units led to similar catalyst distortion energies, whereas lower distortion of the substrate in the thiourea favors **Theo2S** over **Theo2O**. Identical interaction energies for **Theo2S** and **Theo2O** despite stronger H-bonding of the thiourea unit and the lower distortion of substrates in the thiourea model can be explained by forming C–C bond lengths in the located TSs. The forming C–C bond in **TS**_**Theo2S**_ is 2.26 Å compared to 2.19 Å in **TS**_**Theo2O**_ (Figure 4), indicating an earlier TS along the reaction coordinate leading to a loss in favorable interaction energy compensated by less distorted substrates.

The distortion energies of substrates (*trans*-β-nitrostyrene and dimethyl malonate) are in favor of the DMAP/thiourea catalyst, **(*R*)-TS**_**2S**_, by 1.4 kcal mol^–1^ in the full catalytic system as for the model system. However, a 5.1 kcal mol^–1^ higher energetic cost of the thiourea catalyst (**2S**) compared to its urea analogue (**2O**) results in a much higher activation barrier for **(*R*)-TS_2S_**. These results suggest that the energetic penalty of distorting the 2-aminoDMAP/thiourea (**2S**) catalyst to the active conformation plays a significant role in the destabilization of **(*R*)-TS**_**2S**_ with respect to **(*R*)*-*TS_2O_**. This leads to an unexpected decrease in the reactivity of the **2S** catalyst despite the higher acidity of the HBD moiety.

We were also intrigued by the reversal of reactivity from theozyme models to full catalysts between binding modes **BMA** and **BMB** for **1S**. Distortion/interaction analysis for **TS**_**Theo1S-BMA**_ and **TS**_**Theo1S-BMB**_, as well as **TS**_**1S-BMA**_ and **TS**_**1S-BMB**_, again points out the importance of energetic cost of distorting the catalyst to its active conformation, favoring binding mode **BMB** (see SI, Tables S1.5 and S1.6).

Following the distortion/interaction energies along the IRC path and energy decomposition analysis to quantify the components of interaction energies support the pivotal role of catalyst distortion in governing the reactivity trends reversed in the presence of full catalysts compared to that of model systems. When **TS**_**Theo2S**_ and **TS**_**Theo2O**_ are considered, the slightly more favorable interaction energy of the more acidic thiourea decreases the energy along the IRC path for **TS**_**Theo2S**_ (see SI, Figures S1.19 and S1.20). For full catalytic systems, **(*R*)-TS**_**2S**_ and **(*R*)-TS**_**2O**_, the interaction energy again slightly favors the thiourea catalyst (see SI, Figure S1.21); but now, the distortion energies of the substrates are essentially the same along the reaction coordinate, the difference in catalyst distortion energies between **2S** and **2O** reverses the reactivity trend in favor of **(*R*)-TS**_**2O**_ leading to unexpected reactivity of 2-aminoDMAP/urea catalyst compared to its thiourea analogue (see SI, Figure S1.22). The preferred binding pattern for **1S**, **BMA**, or **BMB**, is also determined by lower distortion energies driven by the differences in catalyst distortion terms, while interaction energies are identical along the IRC (see SI, Figures S1.27–S1.30). Natural energy decomposition analysis was performed using NBO 7.0^74^ to analyze the trend in interaction energies. Total interaction energies slightly favor thiourea analogues compared to urea and **BMA** binding mode compared to **BMB**, for the full catalysts as for the model systems (see SI, Tables S1.15 and S1.16) supporting the key role of catalyst distortion in the observed reactivity patterns. The thiourea analogues exhibit more stabilizing interaction energies compared to urea, primarily due to enhanced electronic and charge transfer effects, despite experiencing unfavorable Pauli repulsions.

These results highlight a decisive role of catalyst distortion energy for reactions occurring on conformationally complex potential energy surfaces in the presence of highly flexible multifunctional organocatalytic structures that can successfully be captured by CIPOC uncovering pathways and catalysts seemingly less plausible based on known trends of reactivity and chemical intuition.

Having screened a number of conditions (catalyst loadings, solvent, concentration, and temperature), we have found that this enantioselective reaction proceeds seamlessly with 5 mol % urea catalyst (**2O**) in toluene (0.2 M **3a**) at 5 °C, giving **5a** in 94% ee with 91% yield. It is worth noting that the reaction can also work well at 1% catalyst loading to yield similar selectivity (93% ee), albeit with a lower yield (75%) taking a longer reaction time (7 h) (see the SI).

To show the generality of this optimized condition, we subjected 17 more *trans*-β-nitroalkenes **3b–r** to this enantioselective reaction (Table 1). Almost all gave conjugate adducts with very high enantioselectivities and yields in a few hours. The selectivity and yields of the Michael reactions were apparently insensitive to the electronic nature of the substituents and their substitution patterns. Remarkably, about two-thirds of entries in Table 1 yield adducts with very high enantioselectivities (≥90%).

The scope of malonates was also sought by reacting dime-thyl malonate (**4b**), diisopropyl malonate (**4c**), and diethyl acetamidomalonate (**4d**) with *trans*-β-nitrostyrene (**3a**) under optimized conditions (see SI) Reactions were well tolerated with malonates having higher and lower alkyl end-caps (**5ab**, 92% ee; **5ac**, 85% ee; respectively), but not so well with the 2-substituted derivative (**5ad**, 66% ee). Encouraged by these results, we also applied catalyst **2O** in the gram-scale enantioselective synthesis of **5f** and **5n**, key precursors for Baclofen and its fluorine analogue, respectively (Figure S2.1). Gratifyingly, both batches, carried out at 2 g scale, afforded excellent enantioselectivities (90% ee for **5f**, 94% ee for **5n**) and high yields (>81%) within 3 h.

In conclusion, we have described a simple, chiral 2-aminoDMAP/urea (**2O**) catalyst uncovered by CIPOC among 1600 multifunctional catalyst candidates that enable conjugate addition of malonate esters and *trans*-β-nitroalkenes very rapidly in a few hours with excellent enantioselectivities and yields. The ease of its preparation in a two-step synthesis, robustness, air-stability, and high activities at very low catalyst loadings (1–5%) makes it a favorite for our next efforts along these lines of research. CIPOC identified a combination of double H-bonding, π-stacking interactions, and favorable catalyst distortion as the origin of its superior activities to Takemoto’s *tert*-amine/thiourea catalyst (**1S**). Given its experimentally validated success, we anticipate that CIPOC, with its promising computational catalyst design and discovery capabilities, will inspire the development of new, highly efficient organocatalysts for a wide range of synthetic transformations.

## Supporting Information Available

Detailed computational methodology, additional computational results, distortion/interaction and natural energy decomposition analysis, Cartesian coordinates, absolute energies and thermal corrections of optimized structures, experimental (gram-scale) synthesis procedures, characterization data of the catalysts and products, and copies of NMR spectra and chiral HPLC chromatograms (PDF)

## Abbreviations

CIPOC: computational identification of potential (organo)catalysts
DMAP: 4-dimethylaminopyridine
DMSO: dimethyl sulfoxide
BMA/BMB: binding mode A/B
QM: quantum mechanics
DFT: density functional theory
TS: transition state
CGM: catalytic group map
Theo: theozyme
HBD: H-bond donor
HBA: H-bond acceptor
