# Supplementary material for: Computational Identification of Potential Organocatalysts (CIPOC) Reveals a 2-aminoDMAP/Urea Catalyst Superior to Its Thiourea Analogue
Source: J Am Chem Soc. 2025 Mar 12;147(12):10078–87. doi: 10.1021/jacs.4c10634 (PMC11951081; doi:10.1021/jacs.4c10634)
Supplement: Supplementary file 1 — ja4c10634_si_001.pdf [file ja4c10634_si_001.pdf]

Supporting Information

for

Computational Identification of Potential Organocatalysts (CIPOC) Reveals a 2-aminoDMAP/Urea Catalyst Superior to its Thiourea Analogue

Sezen Alsancak-Koca[a], Yeşim Çamlısoy[a], İrem Bakırcı[b], Murat Işık[c], Nihan Çelebi-Ölçüm[a*] and Cihangir Tanyeli[b*]

[a] *Department of Chemical Engineering, Yeditepe University, 34755 Istanbul, Türkiye.*

[b] *Department of Chemistry, Middle East Technical University, 06800 Ankara, Türkiye.*

[c] *Department of Food Engineering, Bingöl University, 12000 Bingöl, Türkiye.*

TABLE OF CONTENTS

1. COMPUTATIONAL 3

1.1. COMPUTATIONAL PROTOCOL – CIPOC 3

1.1.1. Theozyme Generation 5

1.1.1.1. Catalytic Cycle 5

1.1.1.2. Theozymes Based on Different Catalytic Groups 6

1.1.1.3. Theozymes Based on Different Binding Modes 6

1.1.1.4. Diversification of Theozymes 7

1.1.2. Creating Catalytic Group Map & Catalophore Search 13

1.1.3. Generating Conformer Library of Organocatalyst 14

1.1.4. Matching the Theozyme 14

1.1.5. Ranking & Selection Criteria 15

1.1.6. Quantum Mechanical Calculations 16

1.2. DISTORTION/INTERACTION ANALYSIS 20

1.2.1. Distortion/Interaction Analysis Along the Instrinsic Reaction Coordinates 22

1.3. NATURAL ENERGY DECOMPOSITION ANALYSIS 42

1.4. CATALYST CONFORMER LIBRARIES 50

1.5. CARTESIAN COORDINATES, ABSOLUTE ENERGIES AND CORRECTIONS OF OPTIMIZED STRUCTURES 57

1.6. CARTESIAN COORDINATES OF CATALYST CONFORMER LIBRARY 277

1.7. REFERENCES 377

2. EXPERIMENTAL 379

2.1. GENERAL 379

2.2. CHARACTERIZATION DATA FOR CHIRAL 2-AMINODMAP/(THIO)UREA COMPOUNDS 380

2.3. CHARACTERIZATION DATA FOR CHIRAL CONJUGATE ADDITION PRODUCTS 381

2.4. GRAM-SCALE FORMAL SYNTHESES OF (R)-BACLOFEN AND ITS (S)-CONFIGURATED FLUORINE ANALOG 388

2.5. COPIES OF NMR SPECTRA 389

2.6. COPIES OF CHIRAL HPLC CHROMATOGRAMS 411

2.7. REFERENCES 431

1. COMPUTATIONAL

1.1. COMPUTATIONAL PROTOCOL – CIPOC

We describe a computational approach, CIPOC (Computational Identification of Potential (Organo)Catalysts) that combines the quantitative power of quantum mechanical calculations with drug design tools to shed light on the ambiguous active conformations and mode of action of multifunctional organocatalysts with complex structures, and to discover new catalysts.

This approach (Figure S1.1) is based on the construction of a theoretical active site model including functional groups that are involved in the structures of the multifunctional organocatalysts. It basically is a quantum mechanically optimized transition state of the rate-determining step of the target reaction with optimal 3D arrangement of catalytic functional groups around the substrates. These models are called theozymes. Catalytic group maps (CGM) are constructed with pharmacophore-like features such as an H-bond acceptor and donor, aromatic ring centers, etc. Then, the catalyst conformer library is screened against CGM in order to identify the conformers that can stabilize the optimized transition structure. On the quantum mechanically optimized theozyme, replacing the model catalytic groups with the matching conformer of the catalyst gives the transition structure in the presence of the chiral multifunctional catalyst. Finally, the rate and selectivity of the leads are evaluated using quantum mechanical calculations.

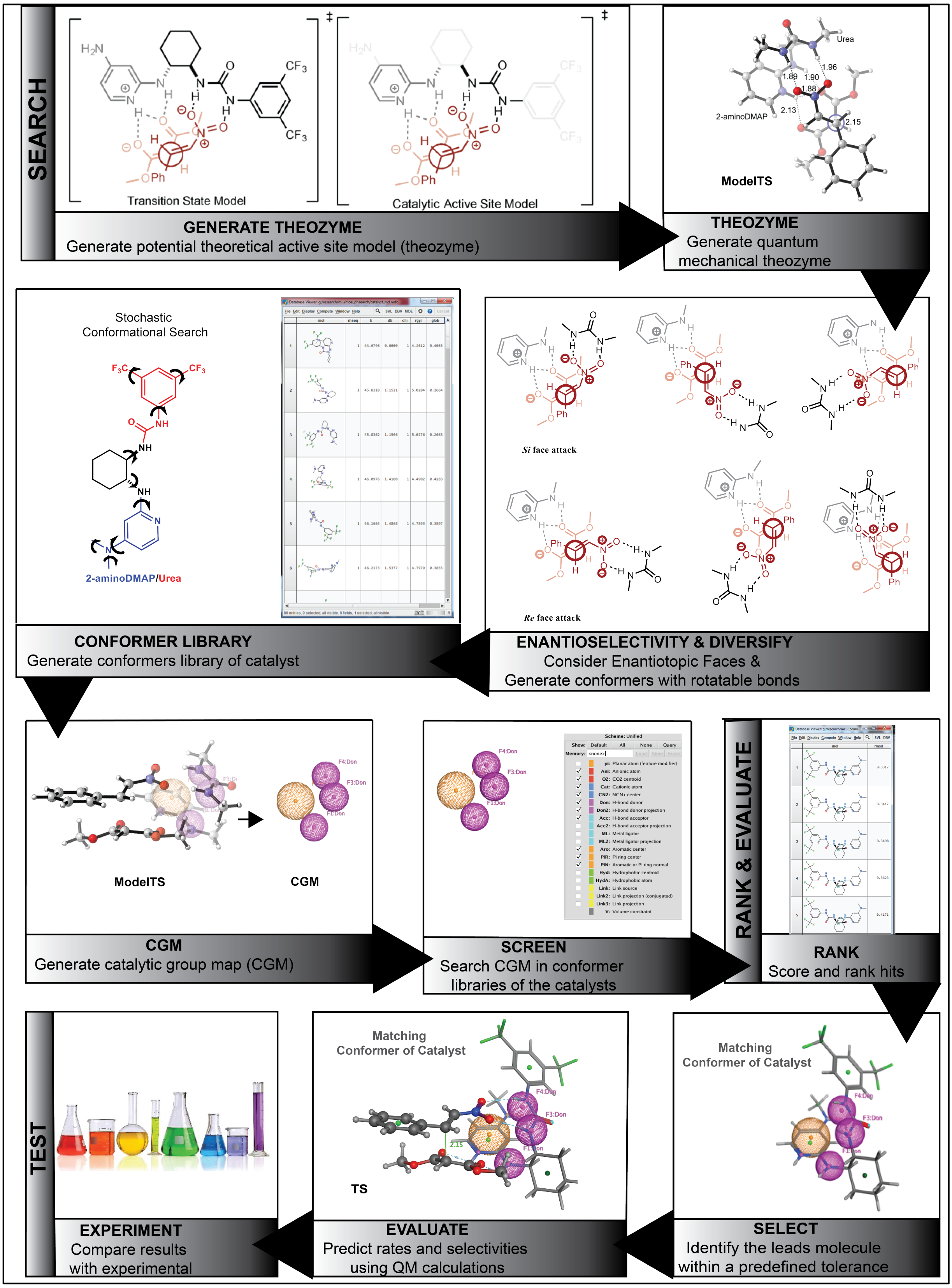

Figure S1.1 | Outline of the developed computational approach

1.1.1. Theozyme Generation

Theozymes created with QM transition state optimization in the presence of catalytic functional groups provide valuable quantitative information on the degree of individual atomic interactions' contribution to catalysis and transition state stabilization. It basically is used to explore the optimal 3D arrangement of catalytic functional groups for maximum transition state stabilization.

1.1.1.1. Catalytic Cycle

We investigate the catalytic cycle for the reaction, which starts with the abstraction of the acidic hydrogen of the malonate by the basic moiety of the catalyst activating it as a nucleophile followed by the conjugate addition of the nucleophile to trans-β-nitrostyrene forming an intermediate (Figure S1.2). In this latter rate and stereo determining C-C coupling step, Takemoto[1] suggests while the protonated basic moiety of the bifunctional catalyst stabilizes the negatively charged nucleophile, the acidic part of the catalyst binds and orients the nitroolefin (BMA) (Fig S1.4). Pápai[2], on the other hand, proposes a reversed binding mode (BMB) for the basic and acidic moieties of the catalyst (Fig S1.4). In the final step, proton transfer from the protonated catalyst to the intermediate forms the Michael adduct and regenerates the catalyst. Here, since our aim was to identify potential catalysts among 1600 organocatalyst candidates rather than modelling reaction mechanism and selectivities, we focused our attention on stereo- and rate determining C-C bond formation step of the catalytic cycle based on alternative binding modes. To clarify the catalytic pathway and proposed alternative binding modes for bifunctional Takemoto's catalyst for the C-C coupling step, which were studied in this work, a brief section describing the catalytic cycle shown in Fig S1.2.

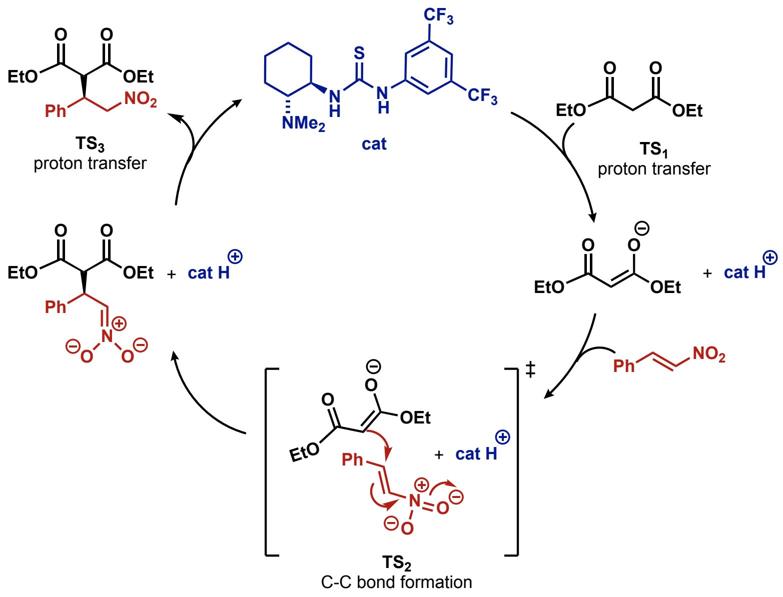

Figure S1.2 | Catalytic cycle for bifunctional thiourea catalyzed Michael Addition

1.1.1.2. Theozymes Based on Different Catalytic Groups

We generated theozymes using different catalytic groups to understand their effect on the activation barrier of the rate determining C-C bond formation step. Three main theozymes which are shown in Figure S1.3 were constructed using different catalytic groups for the most inclusive catalytic functional groups.

First model (Figure S1.3 **Theozyme 1S**) consists of *tert*-amine and thiourea groups and is created based on the cinchona/*β*-isocupredine derivatives and Takemoto's catalyst, which is the most efficient organocatalyst for the target reaction identified so far.[1,3] Other model (Figure S1.3 **Theozyme 2S**) consists of 2-aminoDMAP and thiourea groups and is constructed based on the studies of Tanyeli and co-workers showing the efficiency of 2-aminoDMAP superbase motif to access sulfonamides[5] and squaramides[6]. Another model consisting of 2-aminoDMAP and urea groups (Figure S1.3 **Theozyme 2O**), is urea analogue of **Theozyme 2S**.

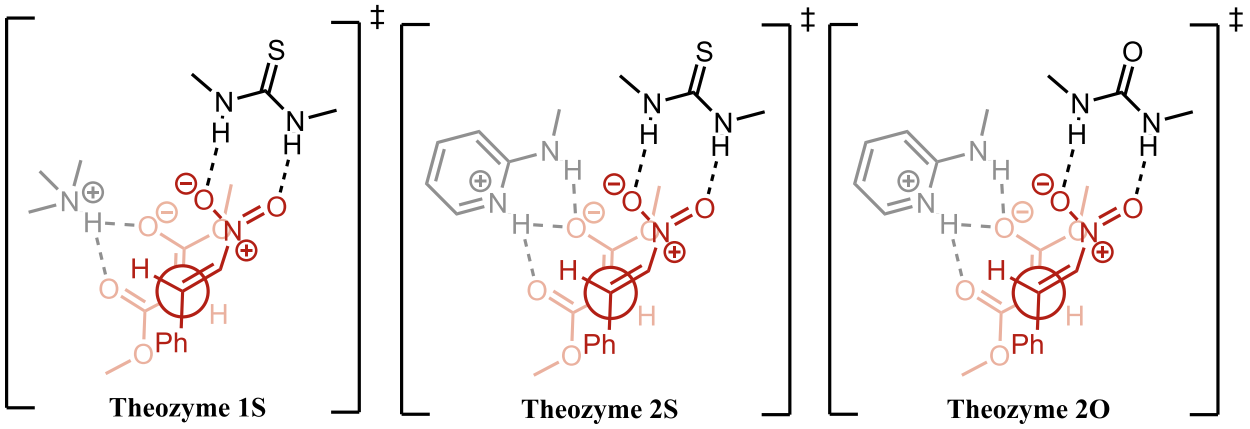

Figure S1.3 | Newman projections (viewed along the forming C-C bond) of the theozymes with different catalytic groups

1.1.1.3. Theozymes Based on Different Binding Modes

We generated theozymes using different binding modes to understand their effect on the activation barrier of the rate determining C-C bond formation step. Two main theozymes which are shown in Figure S1.4 were constructed based on proposal by Takemoto (**Binding mode A** (**BMA**)) and Pápai (**Binding mode B** (**BMB**)). Although Izzo[6] proposed an alternative binding mode, it has not been considered in this study as it is shown to be energetically unfavorable.

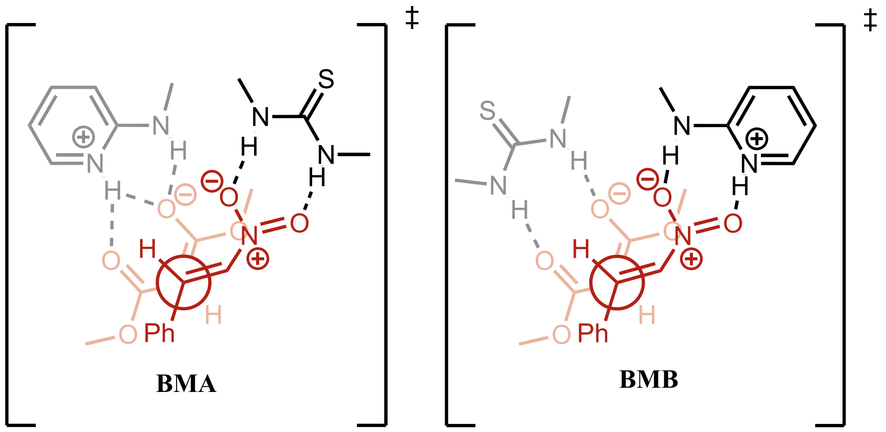

Figure S1.4 | Newman projections (viewed along the forming C-C bond) of the theozymes with different binding modes

1.1.1.4. Diversification of Theozymes

Enantiotopic faces are considered to examine selectivity. The enantioselectivity was explored at the C-C bond formation step through the nucleophilic attack of the enolate to the *Si* face or *Re* face of *trans-β*-nitrostyrene (Figure S1.5).

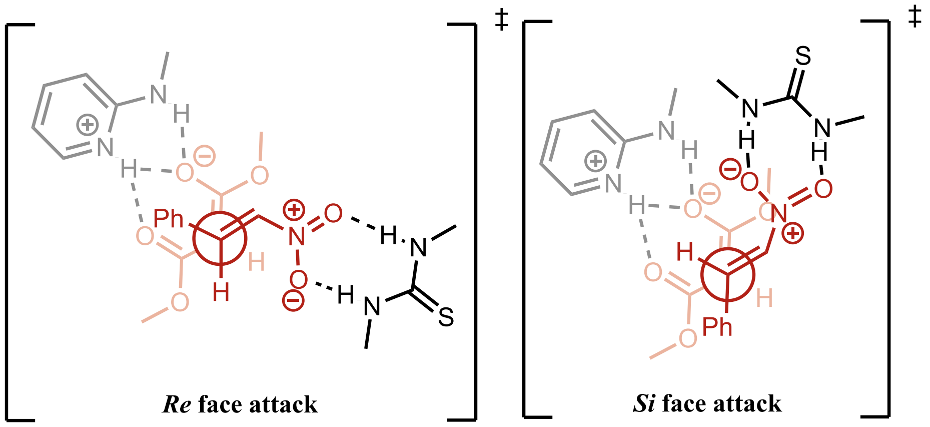

Figure S1.5 | Nucleophilic effect of the enolate to the *Si* face or *Re* face of *trans-β*-nitrostyrene

The next step involved the diversification of transition states via rotation around the forming C-C bond, which determines the relative orientations of catalytic functional groups (Figure S1.6).
For example, diversification for **Theozyme 2S BMA** gives a total of 24 TSs, 12 of which are enantiomers. Located TS structures with promising activation barriers for the C-C bond formation step were used to generate catalytic groups maps (Figure S1.8 & S1.9).

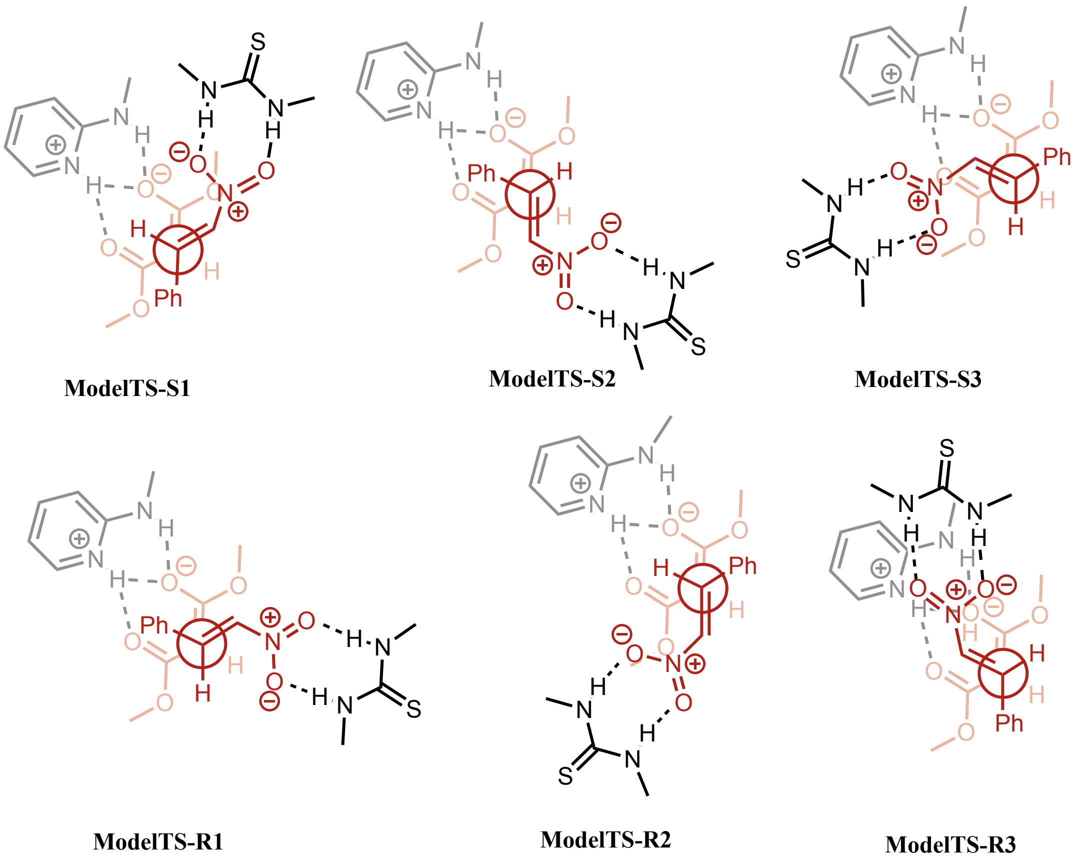

Figure S1.6 | Relative orientations of representative catalytic functional groups

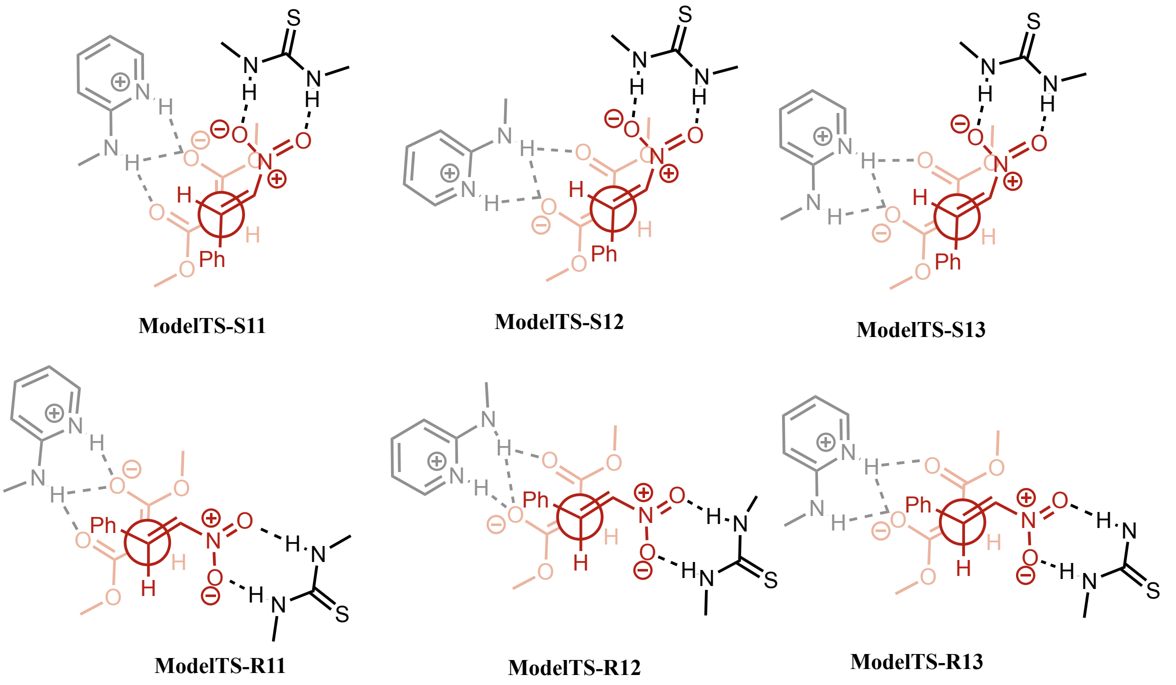

Figure S1.7 | Relative orientations of the 2-amino-pyridine group for *Si* and *Re* face model

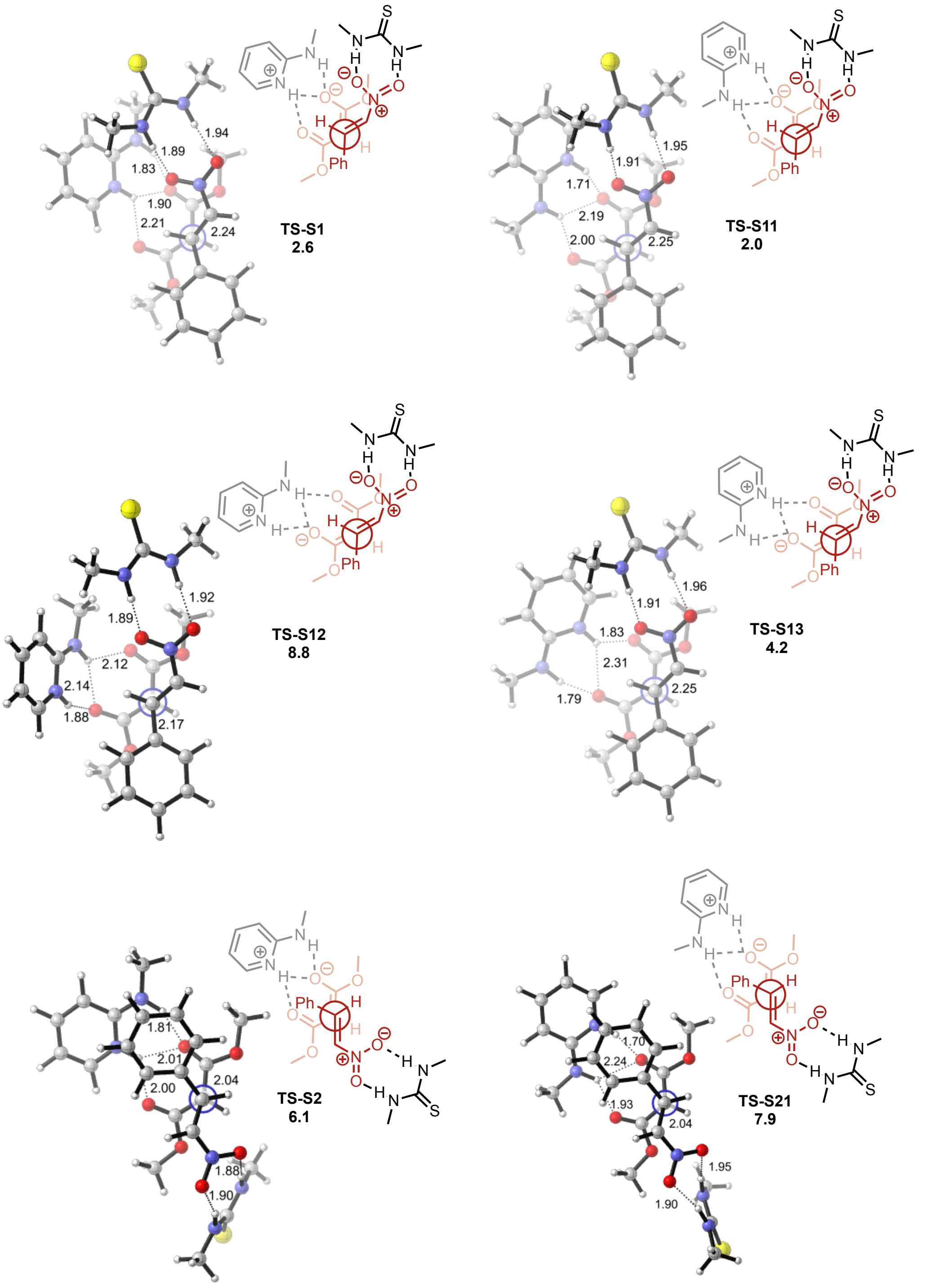

Figure S1.8 | Optimized geometries and activation free energies (relative to the lowest theozyme in kcal mol^{-1}) of diversified theozymes for *Si* face in the presence of **2S** [B3LYP-D3/6-311+G(d,p)-SMD(Toluene)//B3LYP-D3/6-31G(d)-SMD(Toluene)].

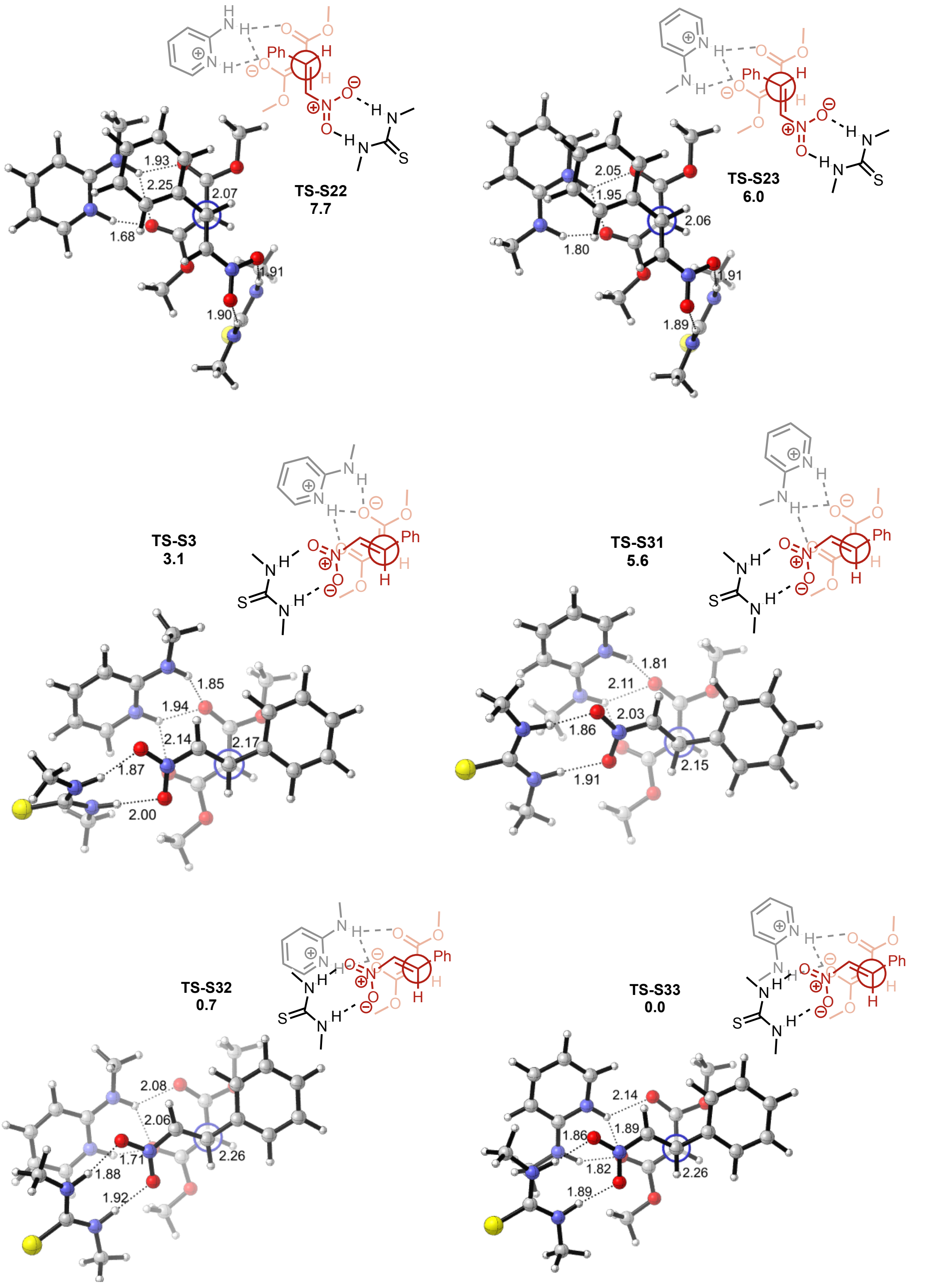

Figure S1.8 | Optimized geometries and activation free energies (relative to the lowest theozyme in kcal mol^{-1}) of diversified theozymes for *Si* face in the presence of **2S** [B3LYP-D3/6-311+G(d,p)-SMD(Toluene)//B3LYP-D3/6-31G(d)-SMD(Toluene)] ***cont'd***.

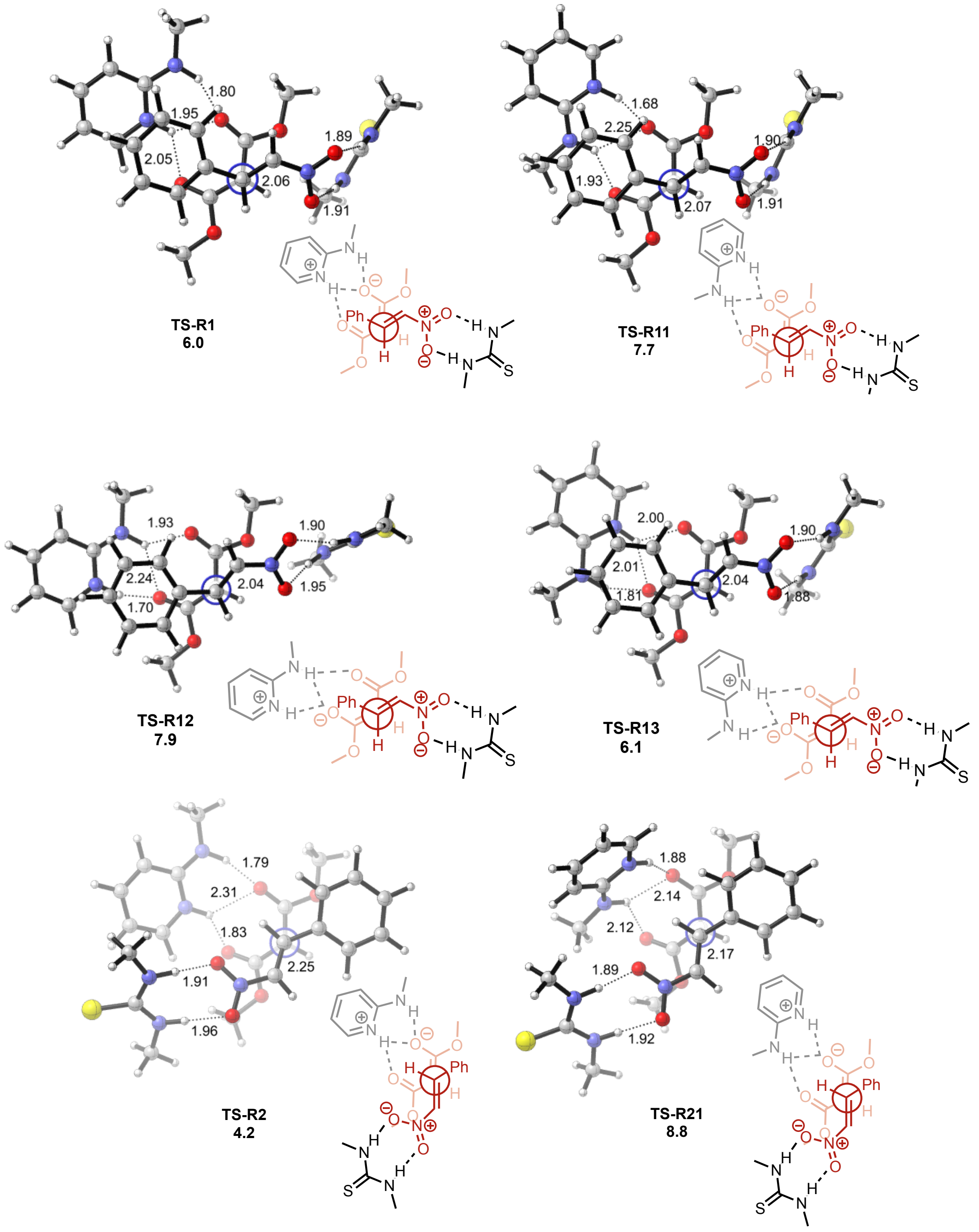

Figure S1.9 | Optimized geometries and activation free energies (relative to the lowest theozyme in kcal mol^{-1}) of diversified theozymes for *Re* face in the presence of **2S** [B3LYP-D3/6-311+G(d,p)-SMD(Toluene)//B3LYP-D3/6-31G(d)-SMD(Toluene)].

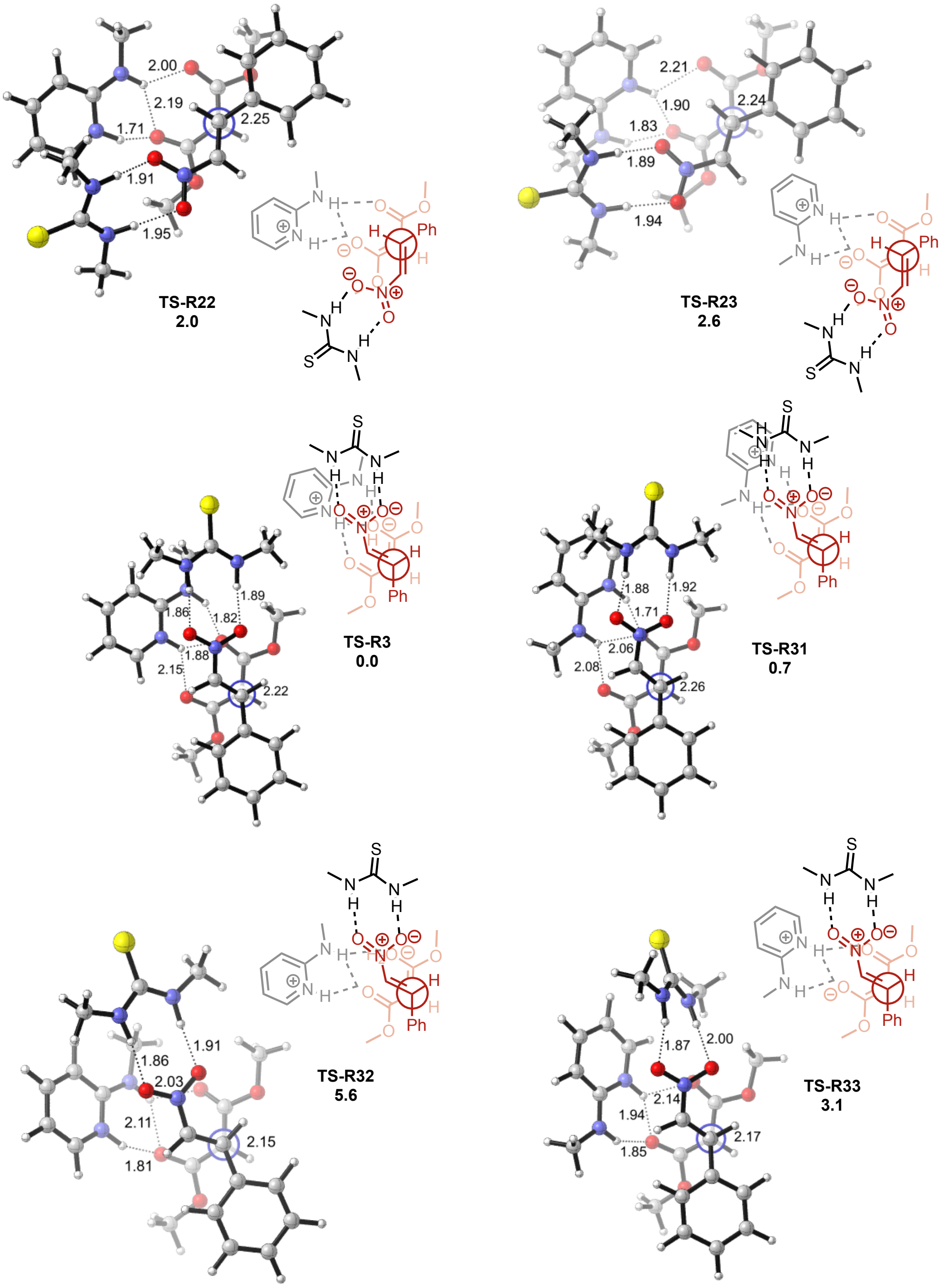

Figure S1.9 | Optimized geometries and activation free energies (relative to the lowest theozyme in kcal mol^{-1}) of diversified theozymes for *Re* face in the presence of **2S** [B3LYP-D3/6-311+G(d,p)-SMD(Toluene)//B3LYP-D3/6-31G(d)-SMD(Toluene)] ***cont'd***.

1.1.2. Creating Catalytic Group Map & Catalophore Search

A catalytic group map (CGM) was constructed using MOE[7] with pharmacophore-like features, such as H-bond acceptor and donor, aromatic ring centers for stabilizing interactions, and volume constraints for stereo-discriminating elements etc., based on the quantum mechanically optimized theozymes. Available features in MOE for pharmacophore query are given in Table S1.1. An inclusive catalytic group map is necessary to ensure matching with three different catalyst skeletons and to avoid missing any matching possibilities. For screening, a partial matching mode was applied to include all our catalyst database with three different skeletons. Partial matching mode is defined as a match of at least two, but not all of pharmacophoric features. Two nitrogens of HBD (alcohol/amine/urea/thiourea) were represented as H-bond donors. One nitrogen of HBA (amine) was represented as H-bond donor/acceptor to include both cyclohexyl and cinchona skeletons. The aromatic ring of HBA was chosen as an aromatic center. Selected features for CGMs (Table S1.2) were used to build the 3D pharmacophore models (Figure S1.10).

Table S1.1 | Examples of Available Features in MOE for Pharmacophore Query

**Feature**	**Explanation**
pi	Planar atom (feature modifier)
Ani	Anionic atom
O2	CO2 centroid
Cat	Cationic atom
CN2	NCN + center
Don	H-bond donor
Don2	H-bond donor projection
Acc	H-bond acceptor
Acc2	H-bond acceptor projection
ML	Metal ligator
ML2	Metal ligator projection
Aro	Aromatic center
PiR	Pi ring center
PiN	Aromatic or Pi ring normal
Hyd	Hydrophobic centroid
HydA	Hydrophobic atom
Link	Link source
Link2	Link projection (conjugated)
Link3	Link projection
V	Volume constraint
Atom Q	Any atom

Table S1.2 | Selected Pharmacophore Groups for CGM (3D layout is shown in Figure S1.10)

Catalytic Group Map	HBA Group			HBD Group		
		Radius	Feature		Radius	Feature
**CGM-1**	F1	1.0	Don / Acc	F3	1.0	Don
	F2	1.4	Aromatic	F4	1.0	Don
**CGM-2**	F1	1.0	Don / Acc	F3	1.0	Don
	F2	1.4	Don2 projection	F4	1.0	Don
				F5	1.4	Don2 projection
				F6	1.4	Don2 projection

1.1.3. Generating Conformer Library of Organocatalyst

A conformer library for each catalyst included in the database is generated to identify the catalyst that could provide the catalytic functional groups in the optimal 3D arrangements depicted in the low-energy theozyme.

An extensive conformational search was carried out for each explored catalyst with MOE (Molecular Operating Environment)[7] software with MMFF94x. Because including chirality in the explored space makes a stochastic search well-suited for organic molecules[8], stochastic search was preferred. The rejection and iteration limit were used as 1000 to achieve a sufficient conformational sampling for an initial assessment in a reasonable amount of computational time.

Root mean square deviation (RMSD) of the two conformations are less than 0.25, these were considered duplicates. All of the catalysts and their conformations were saved in *.mdb* format as database and later employed for pharmacophore screening. All the conformations of matched catalysts were ranked according to the molecular mechanics (MM) force field calculations, and the lowest 20 of them were optimized with QM to calculate the lowest energy conformer of each studied catalyst.

1.1.4. Matching the Theozyme

The catalyst conformer library was screened against the CGM to identify the conformers that can stabilize the determine theozyme structure. The resulting hit conformers were primarily sorted by their matching scores (RMSD) and their deviations from their optimum catalytic geometries. Partial matches were allowed to obtain all possible catalytic candidates.

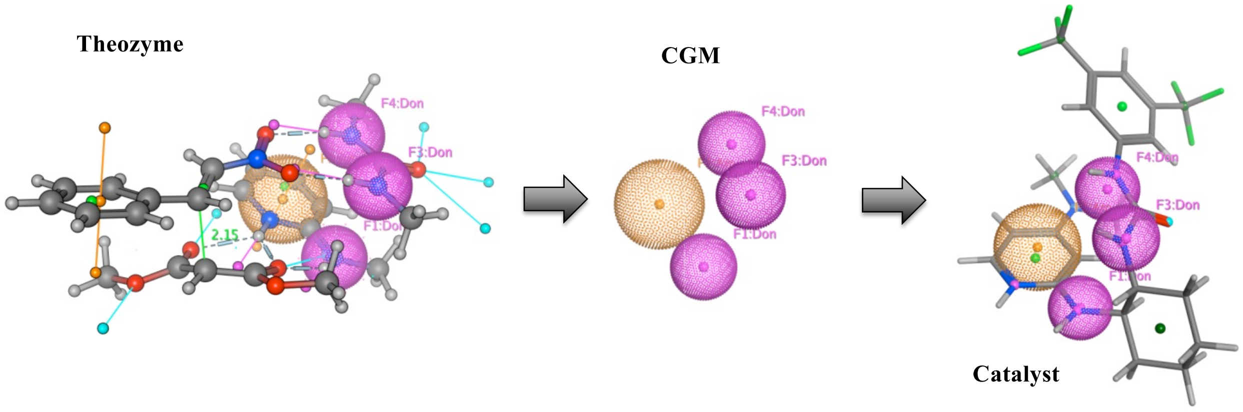

Figure S1.10 | Generation of catalytic group map (CGM) using theozyme and matched conformer of bifunctional catalyst, orange sphere=aromatic center, pink sphere= hydrogen-bond donors

1.1.5. Ranking & Selection Criteria

In principle, for screening purposes, one does not require to generate and diversify many different theozyme models. Since catalytic groups maps (CGMs) are generated based on chemical features described within a spatial tolerance, they have the exquisite ability to identify a variety of functional groups partly or fully involving these chemical features. All theozyme variants, including different binding modes and various orientations of catalytic functional groups, were screened in conformational libraries of 1600 organocatalyst candidates. The resulting matches were ranked and prioritized for QM evaluation. Theozymes including different functional groups, although may not be required for screening, become very handy at this point, as they provide valuable insights on the effect of different functional groups and their optimal arrangements for optimal transition state stabilization. All resulting matches avoiding the steric clash between substrates and catalysts were primarily sorted with respect to the number of matching chemical features. The more the number of stabilizing interactions, the higher the priority of the match considered for QM evaluation. The second criterion considered for ranking is the deviation of the catalytic group map from the ideal geometry depicted in the theozyme. The closer the agreement with the theozyme, the higher the priority of the match considered for QM evaluation. The third criterion taken into account is the relative energy of the matching theozyme compared to the lowest energy one. Finally, since evaluation of as many different compounds as possible has been purposed, matches from different catalyst candidates were primarily selected from the priority lane for initial evaluation. Catalytic structures were evaluated using QM calculations starting from the highest priority matches (called hits) and their activation barriers were compared to a parent catalyst taken as the reference.

1.1.6. Quantum Mechanical Calculations

On the quantum mechanically optimized theozyme, replacing the model catalytic groups with the matching conformer of the catalyst gives the transition structure in the presence of the chiral bifunctional catalyst (Figure S1.11). Finally, the rates and selectivity of the leads were evaluated using QM calculations.

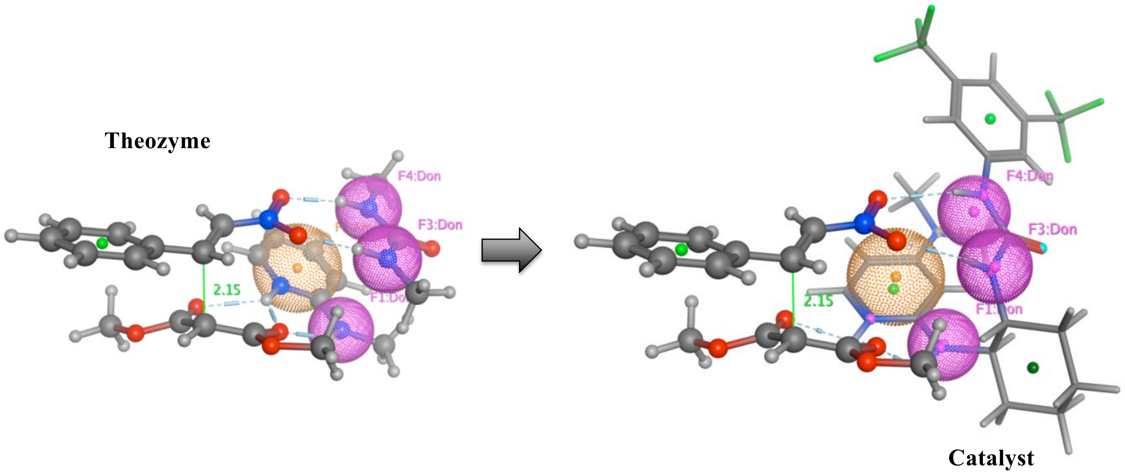

Figure S1.11 | Matching the catalytic active site model, orange sphere=aromatic center, pink sphere= hydrogen-bond donors.

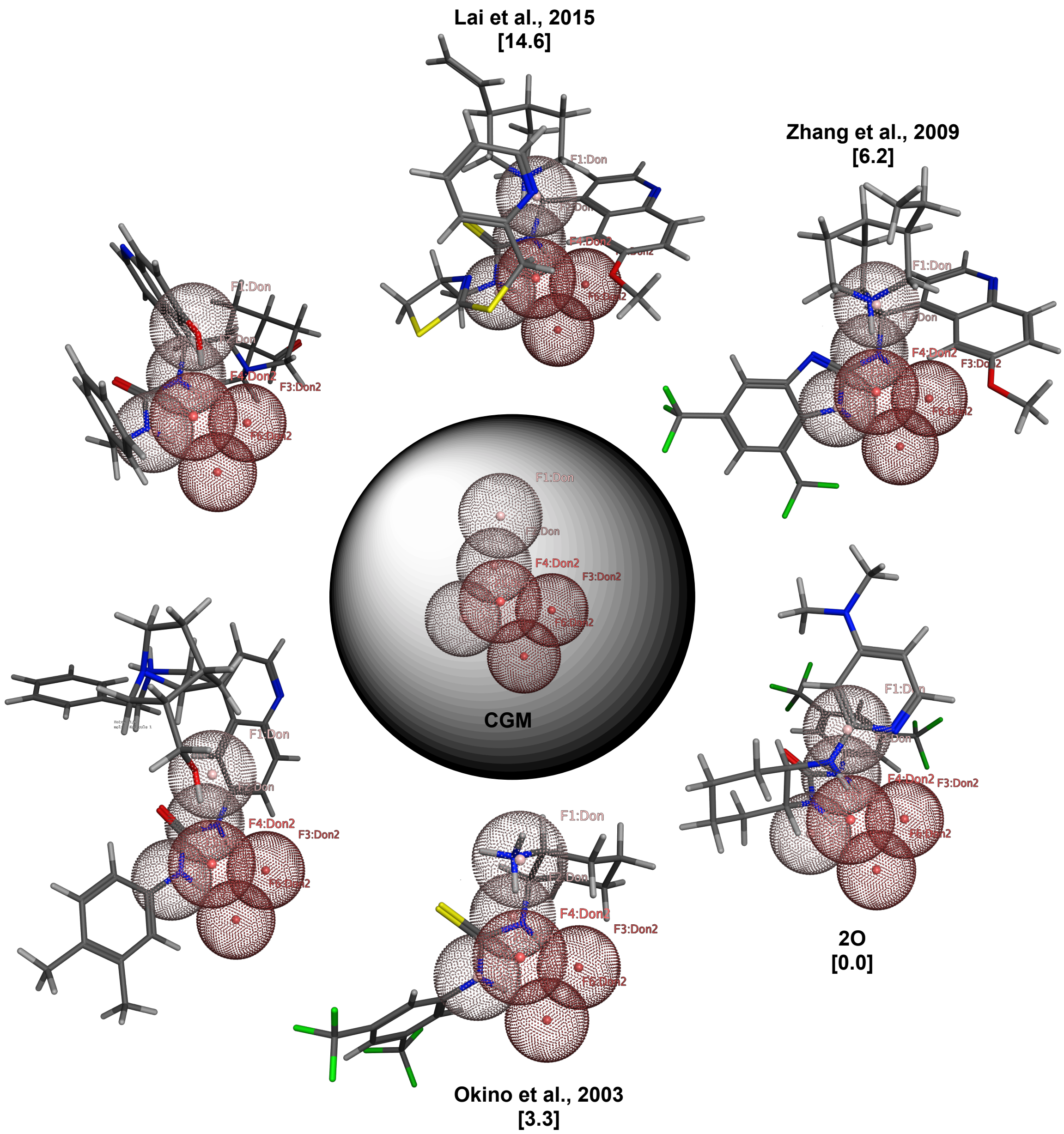

Figure S1.12 | Examples of matched conformers of different chiral organic compounds for selected CGM. References[9-11] were given for structures previously determined catalytic activity and their activation free energies relative to the lowest energy **2O** in kcal/mol. [B3LYP-D3/6-311+G(d,p)-SMD(Toluene)//B3LYP-D3/6-31G(d)-SMD(Toluene)]

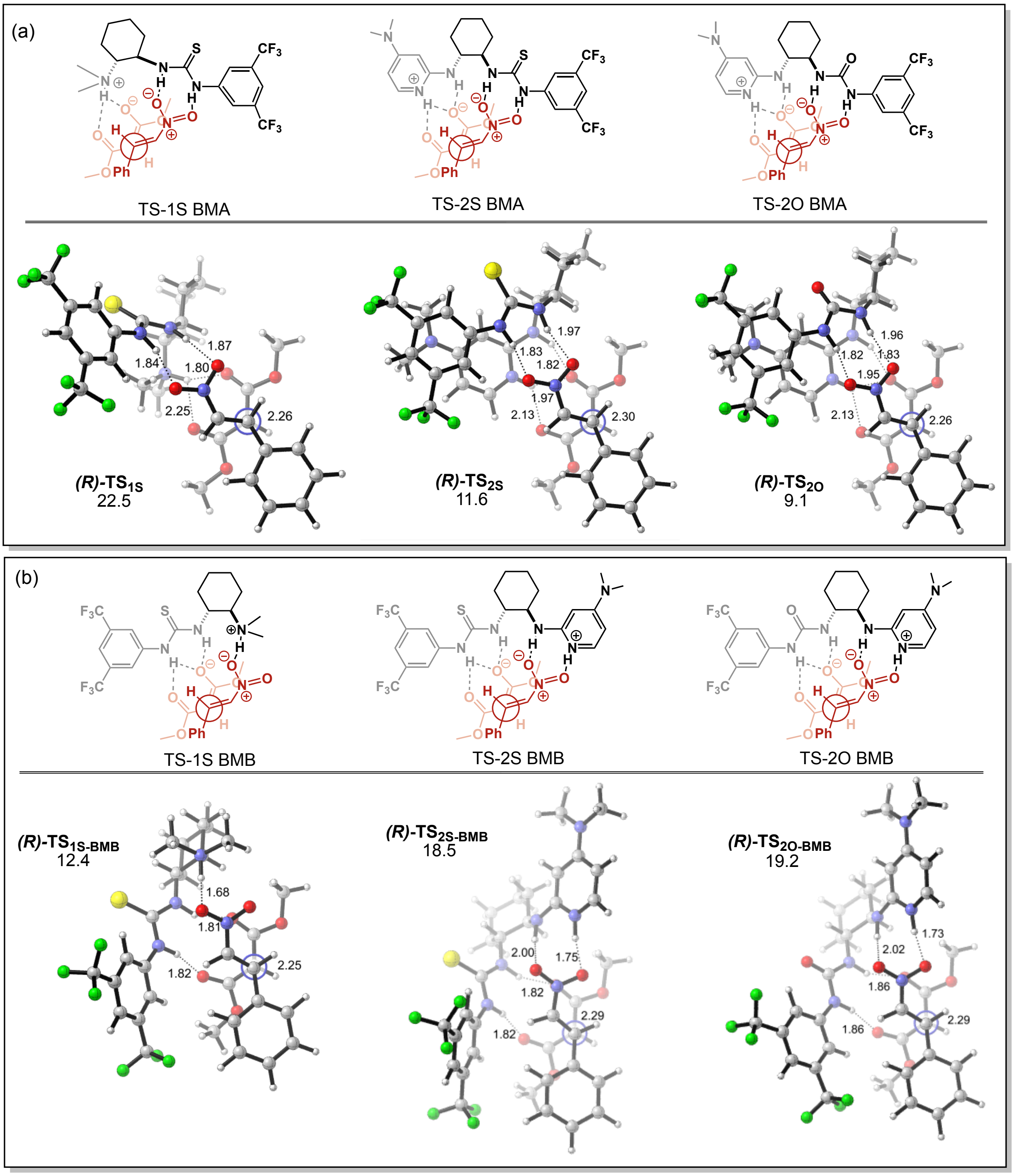

Figure S1.13 | Newman projections (viewed along the forming C-C bond) of the transition states for *Re* face in the presence of **1S**, **2S**, and **2O** with different binding modes a) **BMA** and b) **BMB** and their activation free energies in kcal/mol [B3LYP-D3/6-311+G(d,p)-SMD(Toluene)//B3LYP-D3/6-31G(d)-SMD(Toluene)].

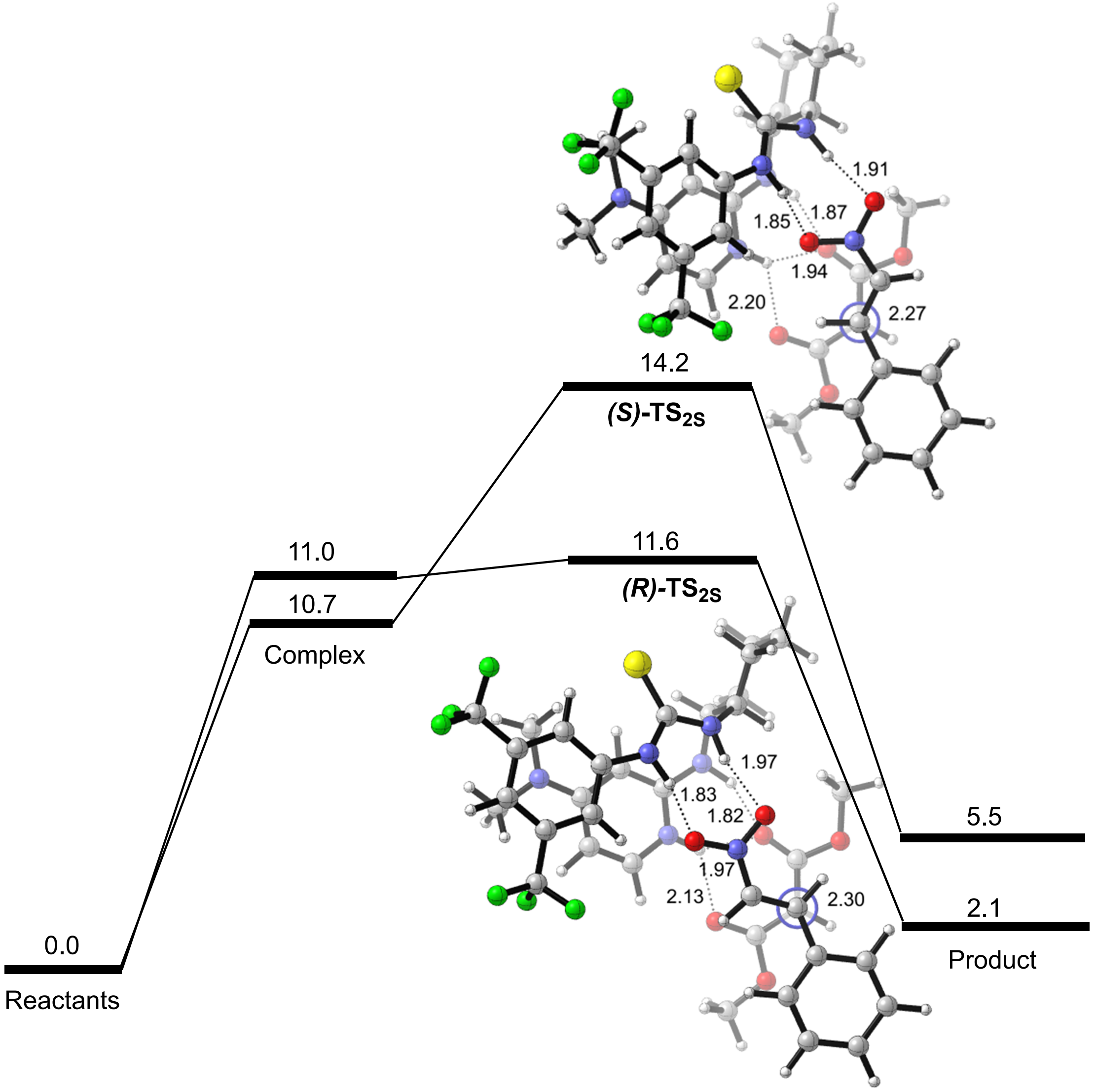

Figure S1.14 | Free energy profile ($\Delta G^{\neq}$) in kcal/mol for the addition reaction of dimethylmalonate to *trans*-*β*-nitrostyrene [B3LYP-D3/6-311+G(d,p)-SMD(Toluene)//B3LYP-D3/6-31G(d)-SMD(Toluene)].

1.2. DISTORTION/INTERACTION ANALYSIS

Distortion/interaction analysis[12] is a tool used to analyze activation barriers that determine reaction rates. In the case of bimolecular reactions, the activation energies are the combined energies needed to distort the reactants into the geometries they have in TS, as well as the interaction energies between the two distorted molecules. The energy required for distorting the molecules is referred to as the distortion energy, which is the primary contributor to the activation barrier. The TS is reached when this distortion energy is overcome by the stabilizing interaction energy.

The distortion/interaction analysis was performed for transition states. Single point energy calculations were performed for each necessary structure (optimized reactants and catalyst, TSs, and the separated, distorted reactants and catalyst from TS structure) with Gaussian 16[13] at B3LYP-D3/6-311+G(d,p)(toluene)//B3LYP-D3/6-31G(d)(toluene) level. The distortion and interaction energies were computed using the electronic energies. The activation energies, ΔE, the distortion energies, ΔE_{dist}, and the interaction energies, ΔE_{int}, are given in kcal/mol.

Table S1.3 | Distortion/Interaction Analysis of ***(R)*-TS$_{2O}$** & ***(R)*-TS$_{2S}$** [B3LYP-D3/6-311+G(d,p)-SMD(Toluene)//B3LYP-D3/6-31G(d)-SMD(Toluene)]

	ΔE_{dist} (dimethyl malonate)	ΔE_{dist} (*trans-(β)*-nitrostyrene)	ΔE_{dist} (catalyst)	ΔE_{int}	TS C-C lentgh (Å)
***(R)*-TS$_{2O}$**	5.6	10.2	6.4	-93.6	2.26
***(R)*-TS$_{2S}$**	5.1	9.3	11.5	-93.4	2.30

Table S1.4 | Distortion/Interaction Analysis of **TS$_{Theo2O}$** & **TS$_{Theo2S}$** [B3LYP-D3/6-311+G(d,p)-SMD(Toluene)//B3LYP-D3/6-31G(d)-SMD(Toluene)]

	ΔE_{dist} (dimethyl malonate)	ΔE_{dist} (*trans-(β)*-nitrostyrene)	ΔE_{dist} (catalyst-DMAP)	ΔE_{dist} (catalyst-urea/thiourea)	ΔE_{int}	TS C-C lentgh (Å)
**TS$_{Theo2O}$**	6.7	11.4	0.7	0.5	-98.6	2.19
**TS$_{Theo2S}$**	5.8	9.9	0.7	0.4	-98.7	2.26

Table S1.5 | Distortion/Interaction Analysis of ***(R)*-TS$_{\mathbf{1S\text{-}BMA}}$** & ***(R)*-TS$_{\mathbf{1S\text{-}BMB}}$** [B3LYP-D3/6-311+G(d,p)-SMD(Toluene)//B3LYP-D3/6-31G(d)-SMD(Toluene)]

	ΔE_{dist} (dimethyl malonate)	ΔE_{dist} (*trans-(β)*-nitrostyrene)	ΔE_{dist} (catalyst)	ΔE_{int}	TS C-C lentgh (Å)
***(R)*-TS$_{\mathbf{1S\text{-}BMA}}$**	5.7	10.7	17.8	-96.9	2.26
***(R)*-TS$_{\mathbf{1S\text{-}BMB}}$**	5.0	10.1	9.7	-97.8	2.25

Table S1.6 | Distortion/Interaction Analysis of **TS$_{\mathbf{Theo1S\text{-}BMA}}$** & **TS$_{\mathbf{Theo1S\text{-}BMB}}$** [B3LYP-D3/6-311+G(d,p)-SMD(Toluene)//B3LYP-D3/6-31G(d)-SMD(Toluene)]

	ΔE_{dist} (dimethyl malonate)	ΔE_{dist} (*trans-(β)*-nitrostyrene)	ΔE_{dist} (catalyst-TMA)	ΔE_{dist} (catalyst-urea/thiourea)	ΔE_{int}	TS C-C lentgh (Å)
**TS$_{\mathbf{Theo1S\text{-}BMA}}$**	5.2	9.7	0.6	0.3	-95.3	2.27
**TS$_{\mathbf{Theo1S\text{-}BMB}}$**	3.3	7.3	1.1	0.8	-89.2	2.39

1.2.1. Distortion/Interaction Analysis Along the Instrinsic Reaction Coordinates

Table S1.7 | Distortion/Interaction Analysis Along the IRC for $\mathbf{TS_{Theo2O}}$

$d_{(C\text{-}C)}$ (Å)	E_{dist}-malonate	E_{dist}-styrene	E_{dist}-catalyst	E_{dist}-total	E_{int}	$E_{IRCpath}$*
2.745	2.9	2.0	1.3	6.2	-88.5	-1657.379830
2.709	3.0	2.2	1.2	6.5	-88.6	-1657.379644
2.669	3.1	2.5	1.2	6.9	-88.8	-1657.379422
2.628	3.3	2.8	1.2	7.3	-88.9	-1657.379162
2.581	3.5	3.3	1.2	8.0	-89.3	-1657.378864
2.532	3.7	3.8	1.2	8.6	-89.7	-1657.378529
2.480	4.0	4.4	1.2	9.6	-90.3	-1657.378166
2.426	4.3	5.3	1.2	10.7	-91.1	-1657.377790
2.369	4.8	6.4	1.2	12.4	-92.5	-1657.377429
2.312	5.3	7.8	1.1	14.2	-94.0	-1657.377117
2.252	5.9	9.4	1.2	16.4	-96.0	-1657.376899
2.192	6.7	11.4	1.1	19.2	-98.6	-1657.376817
2.132	7.6	13.7	1.2	22.5	-101.9	-1657.376908
2.072	8.5	16.3	1.1	25.9	-105.5	-1657.377226
2.010	9.6	19.1	1.2	29.8	-109.9	-1657.377912
1.947	10.9	22.5	1.2	34.5	-115.4	-1657.379064
1.884	12.2	26.1	1.2	39.4	-121.4	-1657.380640
1.823	13.6	29.8	1.2	44.6	-127.8	-1657.382456
1.763	14.9	33.4	1.2	49.5	-133.9	-1657.384260
1.708	16.1	36.4	1.3	53.7	-139.2	-1657.385772
1.663	16.9	38.6	1.3	56.8	-143.0	-1657.386772

*Given in Hartree at B3LYP-D3/6-31G(d)-SMD(Toluene)

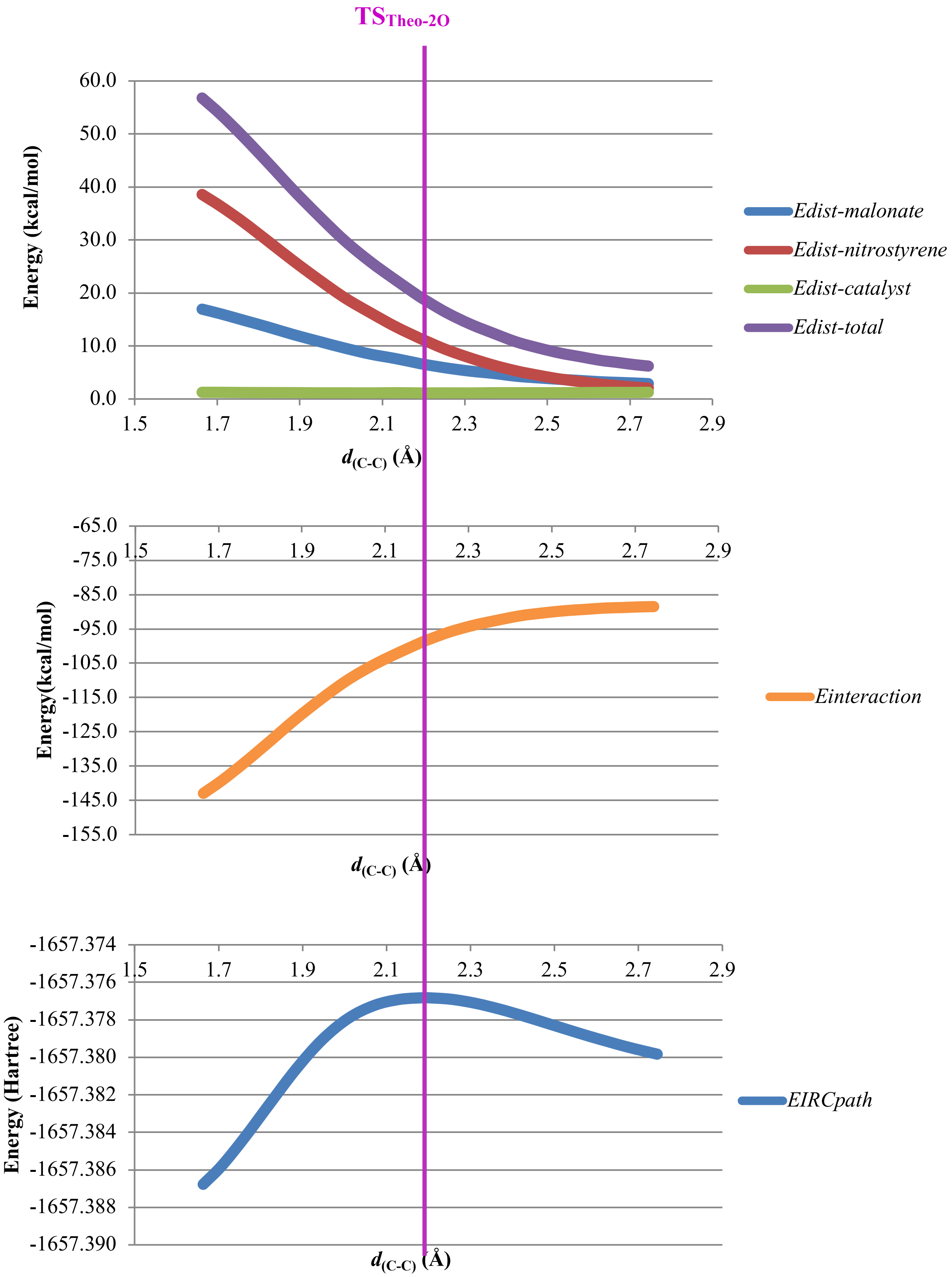

Figure S1.15 | Distortion/interaction analysis along the IRC for **TS$_{Theo2O}$** [B3LYP-D3/6-311+G(d,p)-SMD(Toluene)//B3LYP-D3/6-31G(d)-SMD(Toluene)].

Table S1.8 | Distortion/Interaction Analysis Along the IRC for $\mathbf{TS_{Theo2S}}$

$d_{(C\text{-}C)}$ (Å)	E_{dist}-malonate	E_{dist}-styrene	E_{dist}-catalyst	E_{dist}-total	E_{int}	$E_{IRCpath}$*
2.643	3.2	2.9	1.2	7.2	-91.4	-1980.342740
2.575	3.4	3.5	1.2	8.1	-90.0	-1980.342365
2.503	3.8	4.4	1.2	9.4	-91.6	-1980.341921
2.423	4.3	5.7	1.1	11.2	-93.8	-1980.341443
2.342	4.9	7.4	1.1	13.5	-96.5	-1980.340995
2.259	5.8	9.9	1.1	16.9	-98.7	-1980.340601
2.212	6.4	11.2	1.1	18.7	-100.6	-1980.340742
2.148	7.2	13.4	1.1	21.8	-104.0	-1980.340921
2.059	8.7	17.3	1.2	27.1	-110.3	-1980.341532
1.967	10.3	21.9	1.2	33.4	-118.2	-1980.342986
1.874	12.4	27.4	1.2	40.9	-127.6	-1980.345387
1.785	14.3	32.3	1.3	48.0	-136.3	-1980.348213

*Given in Hartree at B3LYP-D3/6-31G(d)-SMD(Toluene)

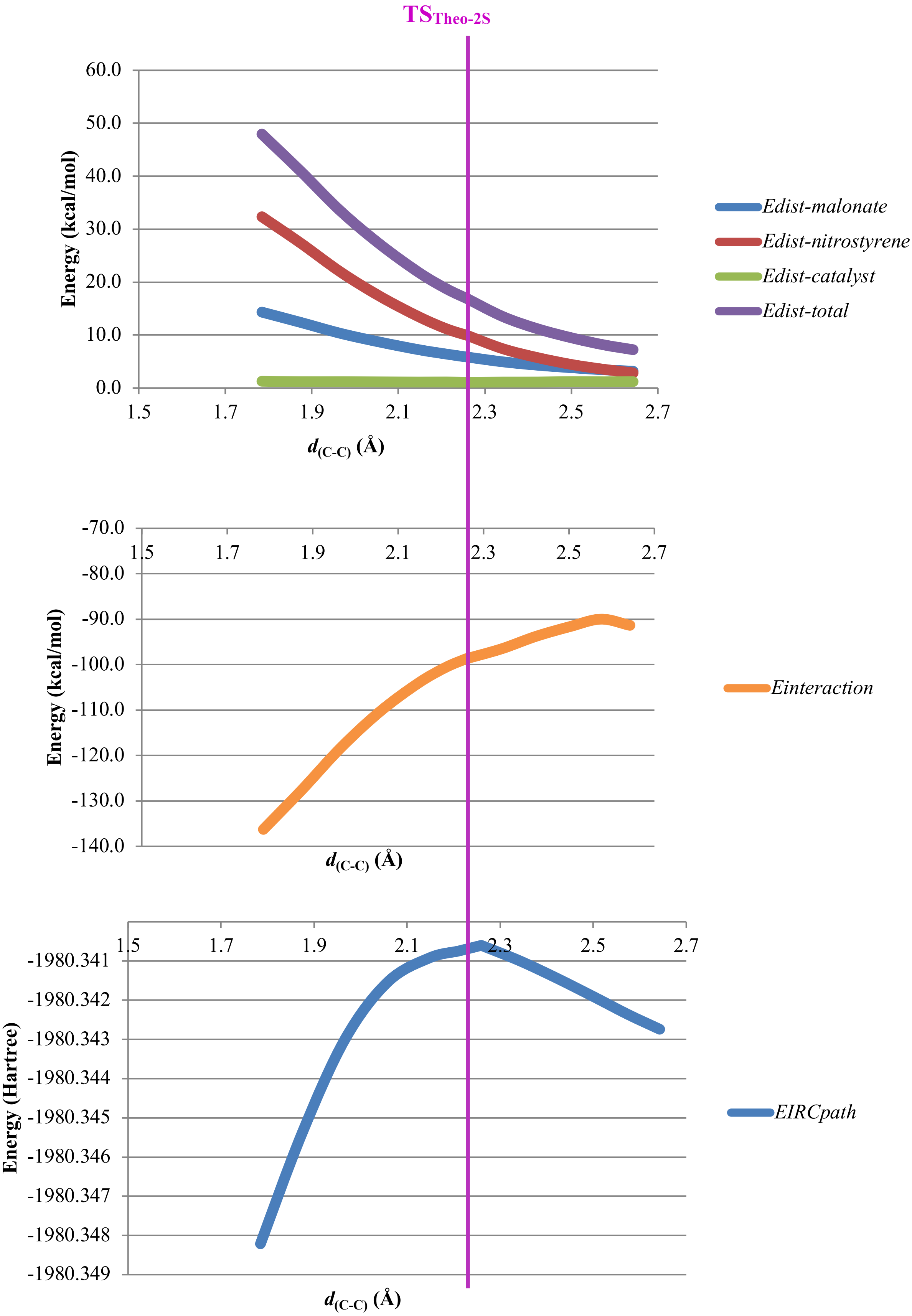

Figure S1.16 | Distortion/interaction analysis along the IRC for **TS$_{\textbf{Theo2S}}$** [B3LYP-D3/6-311+G(d,p)-SMD(Toluene)//B3LYP-D3/6-31G(d)-SMD(Toluene)].

Table S1.9 | Distortion/Interaction Analysis Along the IRC for ***(R)*-TS$_{2O}$**

$d_{(C-C)}$ (Å)	E_{dist}-malonate	E_{dist}-styrene	E_{dist}-catalyst	E_{dist}-total	E_{int}	$E_{IRCpath}$*
2.716	2.7	2.6	6.4	11.7	-84.7	-2812.097584
2.693	2.8	2.7	6.4	12.0	-84.8	-2812.097483
2.662	2.9	3.0	6.4	12.3	-85.0	-2812.097362
2.626	3.0	3.3	6.3	12.6	-85.2	-2812.097216
2.583	3.2	3.7	6.4	13.3	-85.7	-2812.097040
2.537	3.4	4.2	6.4	14.0	-86.2	-2812.096835
2.487	3.6	4.9	6.4	14.9	-86.9	-2812.096608
2.431	4.0	5.9	6.4	16.3	-88.0	-2812.096373
2.374	4.4	7.0	6.4	17.9	-89.4	-2812.096154
2.316	5.0	8.3	6.4	19.8	-91.0	-2812.095988
2.255	5.6	10.2	6.4	22.2	-93.4	-2812.095922
2.195	6.4	12.2	6.5	25.1	-96.2	-2812.096003
2.133	7.2	14.5	6.5	28.2	-99.5	-2812.096271
2.071	8.2	17.3	6.6	32.1	-103.7	-2812.096779
2.008	9.4	20.3	6.6	36.3	-108.5	-2812.097666
1.944	10.7	23.9	6.7	41.3	-114.4	-2812.099024
1.881	12.0	27.6	6.7	46.3	-120.7	-2812.100795
1.818	13.5	31.2	6.8	51.5	-127.1	-2812.102778
1.758	14.9	35.0	6.8	56.7	-133.7	-2812.104704
1.703	16.0	37.7	6.9	60.7	-138.8	-2812.106282

*Given in Hartree at B3LYP-D3/6-31G(d)-SMD(Toluene)

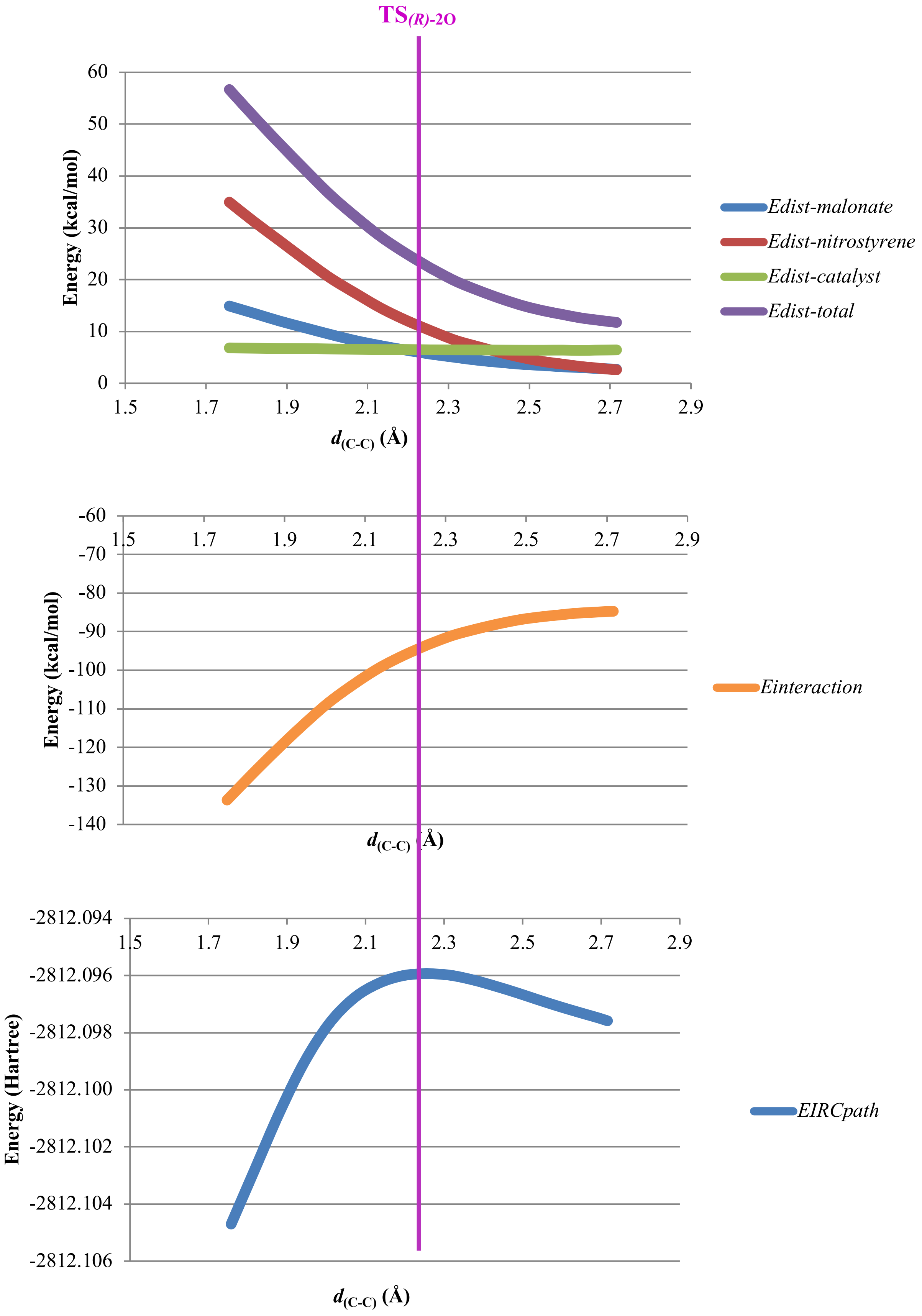

Figure S1.17 | Distortion/interaction analysis along the IRC for ***(R)*-TS$_{2O}$** [B3LYP-D3/6-311+G(d,p)-SMD(Toluene)//B3LYP-D3/6-31G(d)-SMD(Toluene)].

Table S1.10 | Distortion/Interaction Analysis Along the IRC for ***(R)*-TS$_{2S}$**

$d_{(C-C)}$ (Å)	E_{dist}-malonate	E_{dist}-styrene	E_{dist}-catalyst	E_{dist}-total	E_{int}	$E_{IRCpath}$*
2.675	2.7	3.1	11.5	17.2	-86.3	-3135.045344
2.643	2.9	3.4	11.5	17.7	-86.6	-3135.045254
2.608	2.9	3.7	11.5	18.1	-87.0	-3135.045147
2.566	3.2	4.2	11.5	18.8	-87.5	-3135.045017
2.521	3.4	4.7	11.5	19.6	-88.1	-3135.044867
2.470	3.7	5.5	11.5	20.7	-89.0	-3135.044705
2.415	4.1	6.5	11.5	22.1	-90.2	-3135.044548
2.359	4.6	7.8	11.5	23.8	-91.7	-3135.044423
2.300	5.1	9.3	11.5	25.8	-93.6	-3135.044371
2.241	5.7	11.0	11.5	28.2	-95.9	-3135.044438
2.181	6.4	13.1	11.5	31.1	-98.9	-3135.044665
2.120	7.4	15.7	11.6	34.6	-102.7	-3135.045087
2.060	8.4	18.5	11.6	38.5	-107.0	-3135.045760
1.997	9.5	21.5	11.7	42.7	-111.9	-3135.046823
1.935	10.9	25.1	11.7	47.7	-117.9	-3135.048339
1.873	12.2	28.8	11.8	52.7	-124.2	-3135.050227
1.811	13.5	32.5	11.8	57.8	-130.6	-3135.052279
1.752	14.9	36.0	11.9	62.9	-137.0	-3135.054230
1.699	16.0	38.6	11.9	66.6	-141.8	-3135.055798

*Given in Hartree at B3LYP-D3/6-31G(d)-SMD(Toluene)

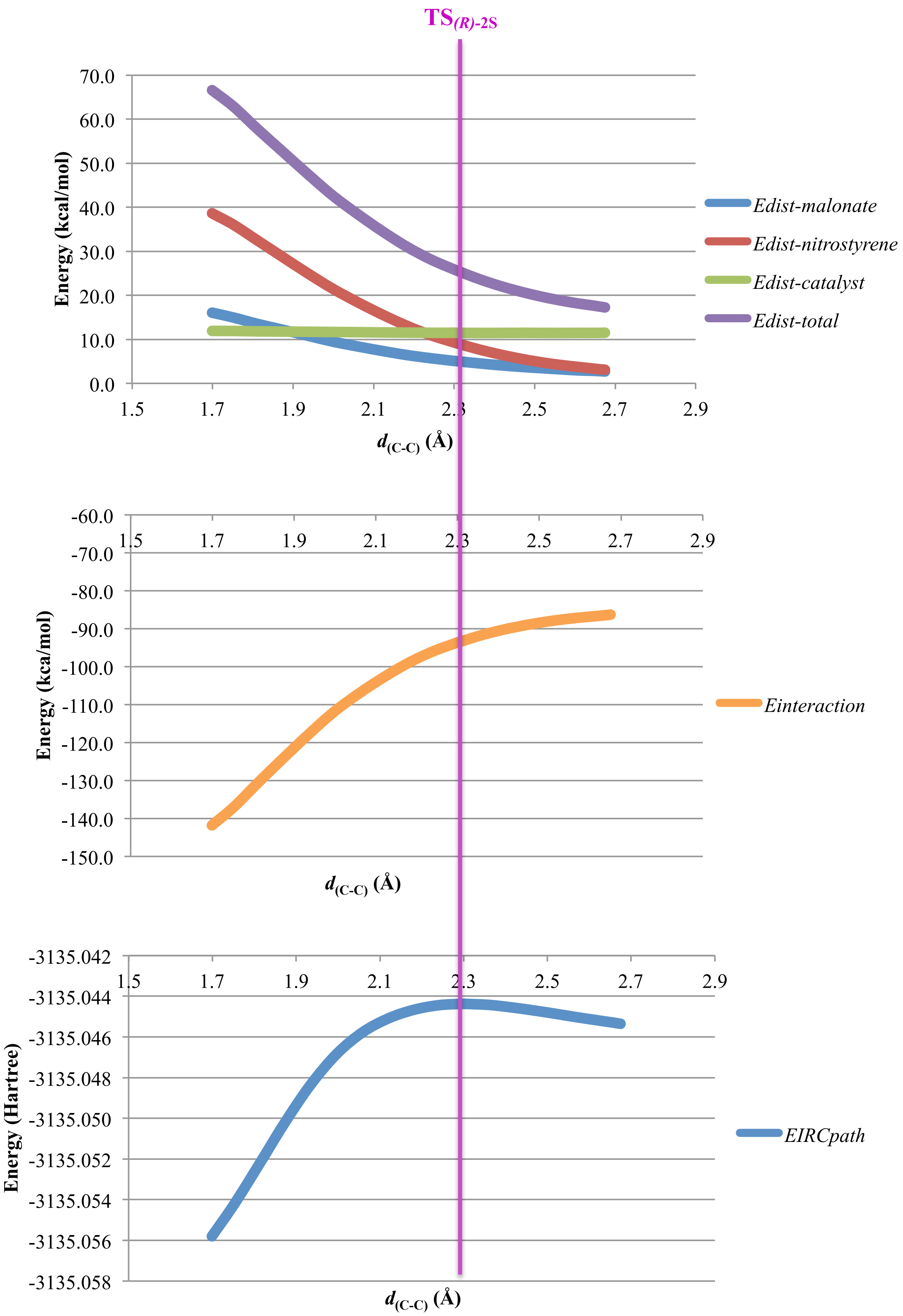

Figure S1.18 | Distortion/interaction analysis along the IRC for ***(R)*-TS$_{2S}$** [B3LYP-D3/6-311+G(d,p)-SMD(Toluene)//B3LYP-D3/6-31G(d)-SMD(Toluene)].

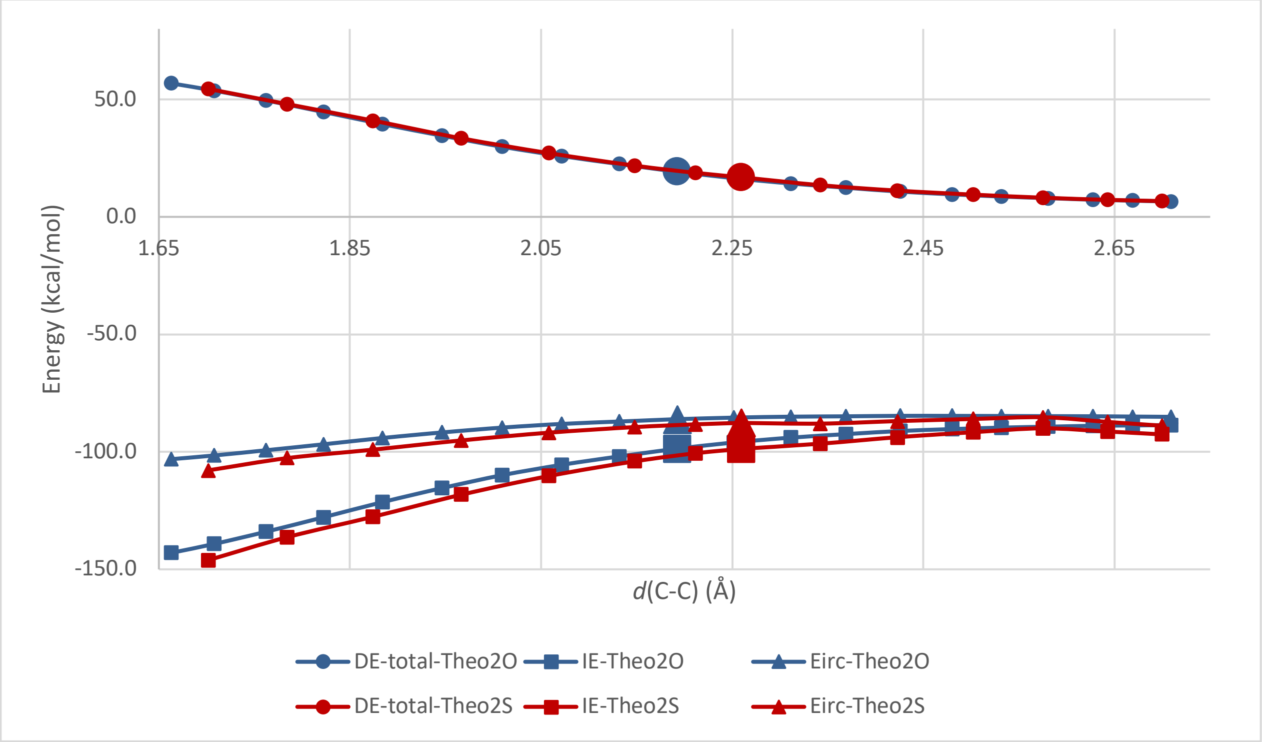

Figure S1.19 | Distortion/interaction diagram along the IRC for **TS$_{\mathbf{Theo2S}}$** vs **TS$_{\mathbf{Theo2O}}$** [B3LYP-D3/6-311+G(d,p)-SMD(Toluene)//B3LYP-D3/6-31G(d)-SMD(Toluene)].

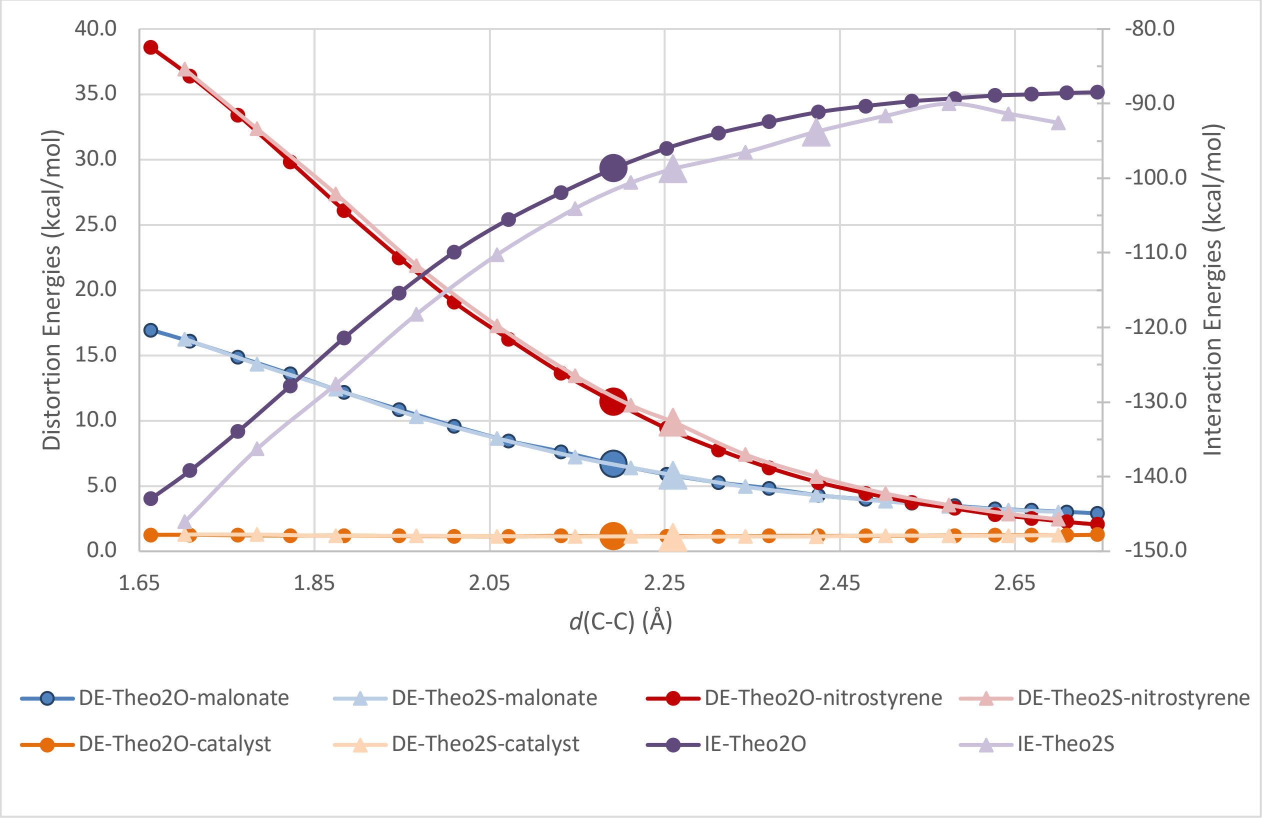

Figure S1.20 | Individual distortion (DE) and interaction (IE) energies along the IRC for **TS$_{\mathbf{Theo2S}}$** vs **TS$_{\mathbf{Theo2O}}$** [B3LYP-D3/6-311+G(d,p)-SMD(Toluene)//B3LYP-D3/6-31G(d)-SMD(Toluene)].

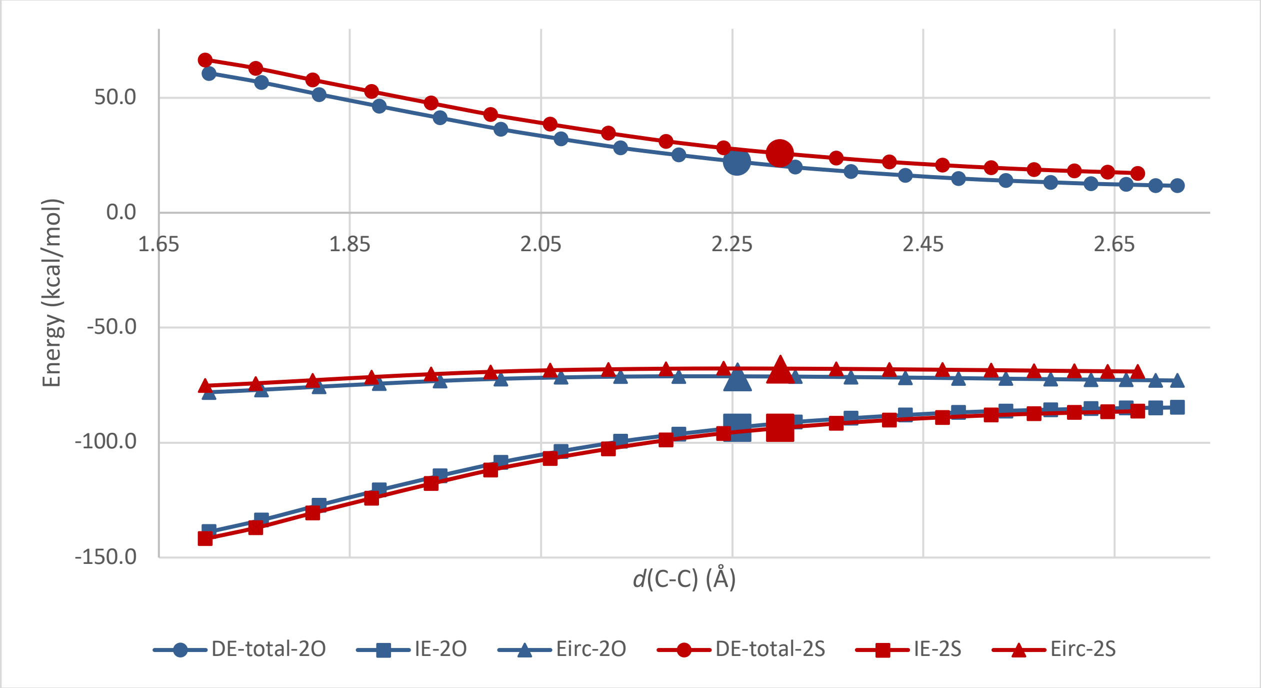

Figure S1.21 | Distortion/interaction diagram along the IRC for ***(R)*-TS$_{2S}$** vs ***(R)*-TS$_{2O}$** [B3LYP-D3/6-311+G(d,p)-SMD(Toluene)//B3LYP-D3/6-31G(d)-SMD(Toluene)] (Larger markers indicate the location of TS)

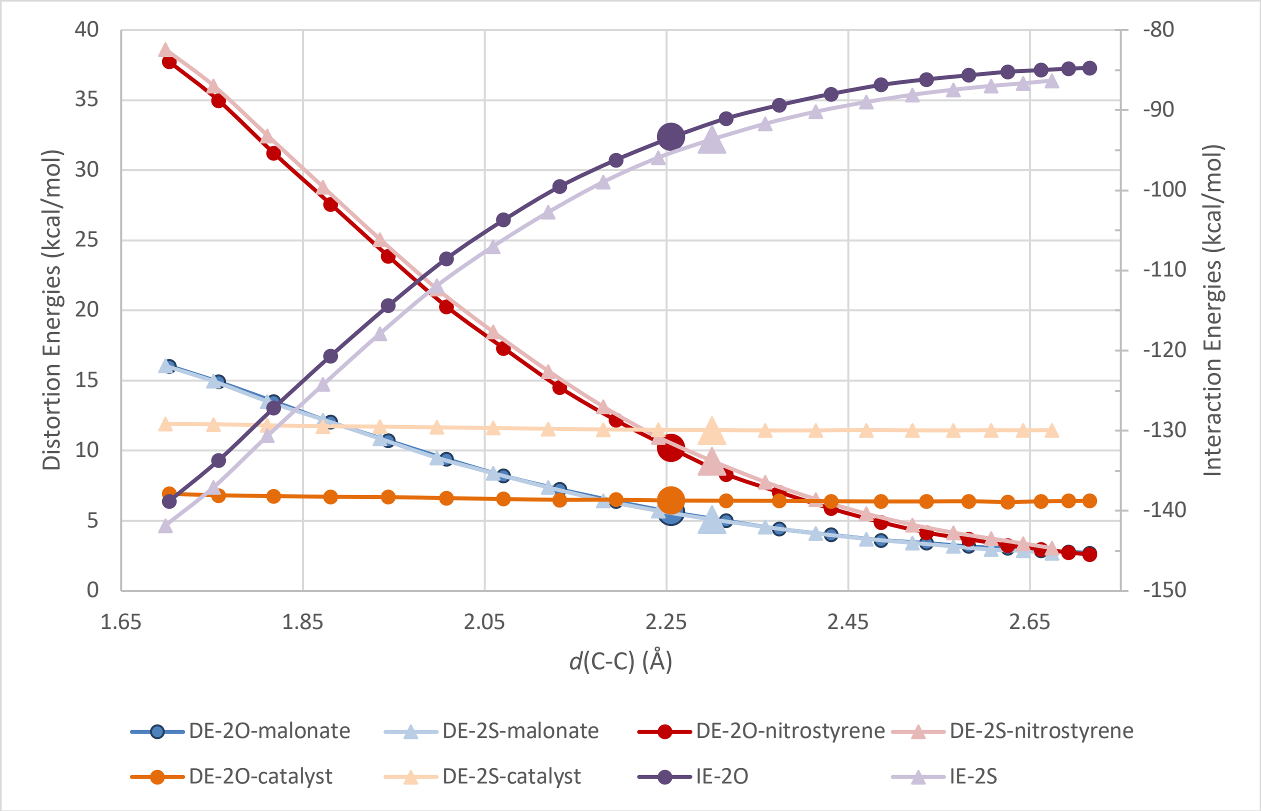

Figure S1.22 | Individual distortion (DE) and interaction (IE) energies along the IRC for ***(R)*-TS$_{2S}$** vs ***(R)*-TS$_{2O}$** [B3LYP-D3/6-311+G(d,p)-SMD(Toluene)//B3LYP-D3/6-31G(d)-SMD(Toluene)]. (Larger markers indicate the location of TS)

Table S1.11 | Distortion/Interaction Analysis Along the IRC for **TS$_{Theo1S\text{-}BMA}$**

$d_{(C\text{-}C)}$ (Å)	E_{dist}-malonate	E_{dist}-styrene	E_{dist}-catalyst	E_{dist}-total	E_{int}	$E_{IRCpath}$*
2.763	2.3	2.3	1.1	5.7	-87.4	-1811.849761
2.734	2.4	2.5	1.0	5.9	-87.4	-1811.849640
2.701	2.5	2.8	1.0	6.2	-87.6	-1811.849500
2.664	2.6	3.0	1.0	6.6	-87.8	-1811.849338
2.623	2.7	3.4	1.0	7.1	-88.1	-1811.849152
2.580	2.9	3.8	0.9	7.6	-88.4	-1811.848943
2.534	3.1	4.3	1.0	8.4	-88.9	-1811.848715
2.485	3.4	5.0	0.9	9.3	-89.6	-1811.848476
2.434	3.7	5.8	0.9	10.4	-90.4	-1811.848242
2.379	4.1	6.9	0.9	11.9	-91.7	-1811.848035
2.325	4.6	8.1	0.9	13.6	-93.2	-1811.847883
2.269	5.2	9.7	0.9	15.9	-95.3	-1811.847824
2.212	5.9	11.4	0.9	18.3	-97.6	-1811.847895
2.155	6.8	13.5	0.9	21.2	-100.6	-1811.848130
2.097	7.6	16.0	0.9	24.6	-104.1	-1811.848557
2.039	8.6	18.6	0.9	28.1	-108.1	-1811.849264
1.979	9.7	21.5	0.9	32.2	-112.8	-1811.850363
1.919	11.0	25.0	0.9	36.9	-118.5	-1811.851879
1.860	12.3	28.5	0.9	41.7	-124.4	-1811.853700
1.802	13.6	31.8	0.9	46.4	-130.4	-1811.855633
1.745	14.8	35.1	1.0	50.9	-136.0	-1811.857439
1.695	15.9	37.7	1.0	54.6	-140.7	-1811.858877
1.658	16.6	39.1	1.0	56.8	-143.5	-1811.859810

*Given in Hartree at B3LYP-D3/6-31G(d)-SMD(Toluene)

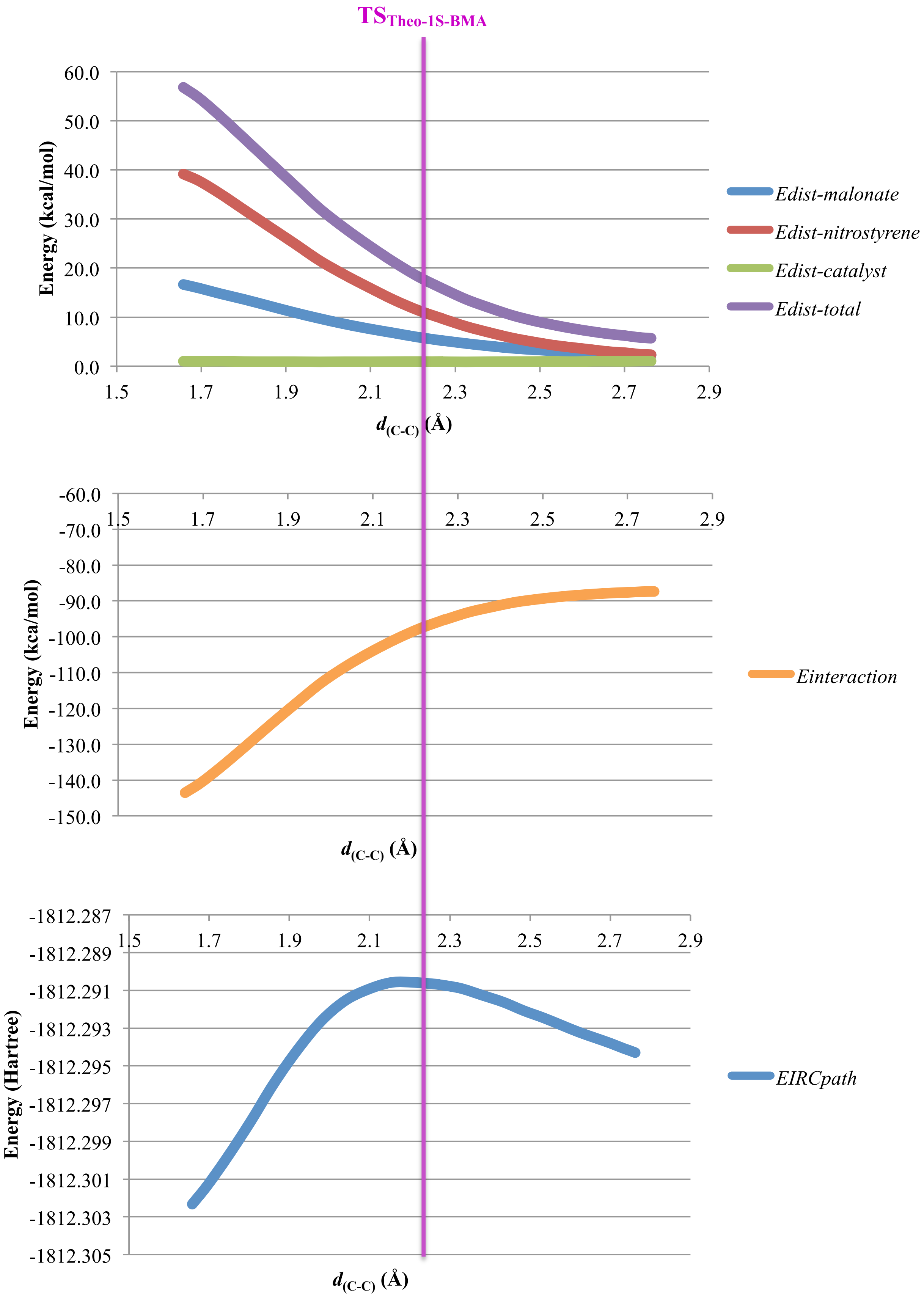

Figure S1.23 | Distortion/interaction analysis along the IRC for **$TS_{Theo1S\text{-}BMA}$** [B3LYP-D3/6-311+G(d,p)-SMD(Toluene)//B3LYP-D3/6-31G(d)-SMD(Toluene)].

Table S1.12 | Distortion/Interaction Analysis Along the IRC for **$TS_{Theo1S\text{-}BMB}$**

$d_{(C\text{-}C)}$ (Å)	E_{dist}-malonate	E_{dist}-styrene	E_{dist}-catalyst	E_{dist}-total	E_{int}	$E_{IRCpath}$*
2.669	1.7	3.2	1.9	6.8	-84.2	-1811.843019
2.642	1.8	3.5	1.8	7.2	-84.5	-1811.842967
2.610	2.0	3.8	1.8	7.6	-84.9	-1811.842906
2.574	2.1	4.2	1.8	8.1	-85.3	-1811.842836
2.531	2.3	4.8	1.8	9.0	-86.0	-1811.842760
2.486	2.6	5.5	1.9	10.1	-87.0	-1811.842687
2.439	2.9	6.2	1.9	11.0	-87.8	-1811.842627
2.389	3.3	7.3	1.9	12.5	-89.3	-1811.842602
2.339	3.8	8.4	1.9	14.1	-90.7	-1811.842637
2.286	4.3	9.8	1.9	16.0	-92.7	-1811.842762
2.232	5.0	11.6	2.0	18.6	-95.3	-1811.843013
2.178	5.7	13.5	2.1	21.3	-98.4	-1811.843420
2.123	6.5	15.9	2.2	24.6	-102.0	-1811.844006
2.068	7.5	18.3	2.3	28.1	-106.0	-1811.844808
2.012	8.5	21.1	2.4	32.0	-110.6	-1811.845927
1.956	9.7	24.1	2.5	35.2	-115.8	-1811.847422
1.899	10.9	27.5	2.6	41.0	-121.8	-1811.849259
1.843	12.2	30.7	2.7	45.6	-127.7	-1811.851304
1.788	13.4	33.9	2.8	50.2	-133.7	-1811.853375
1.735	14.5	36.9	3.0	54.4	-139.1	-1811.855261
1.689	15.5	39.2	3.1	57.8	-143.5	-1811.856759
1.654	16.2	40.6	3.2	60.0	-146.4	-1811.857776

*Given in Hartree at B3LYP-D3/6-31G(d)-SMD(Toluene)

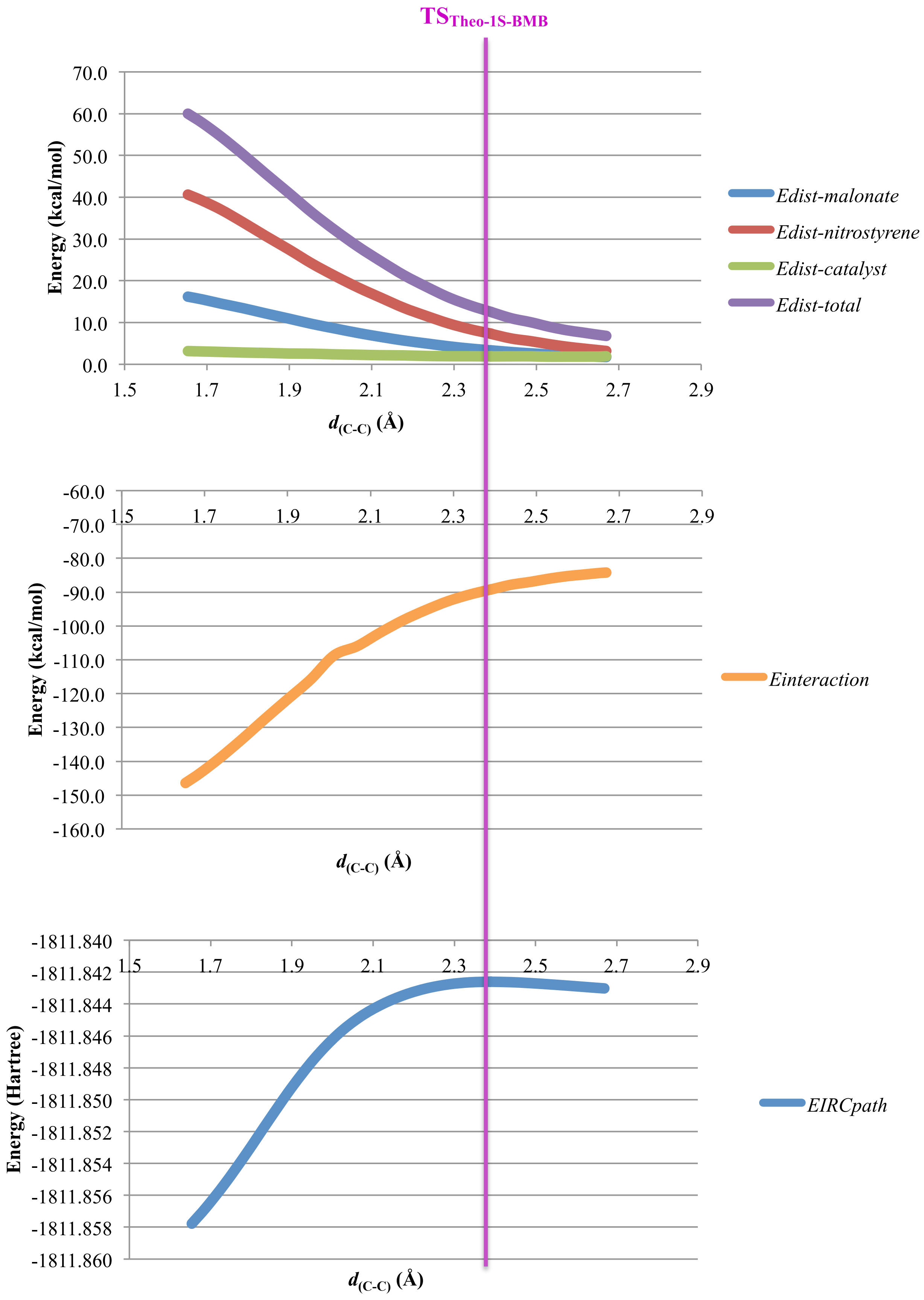

Figure S1.24 | Distortion/interaction analysis along the IRC for **TS$_{\mathbf{Theo1S\text{-}BMB}}$** [B3LYP-D3/6-311+G(d,p)-SMD(Toluene)//B3LYP-D3/6-31G(d)-SMD(Toluene)].

Table S1.13 | Distortion/Interaction Analysis Along the IRC for ***(R)*-TS$_{1S\text{-}BMA}$**

$d_{(C\text{-}C)}$ (Å)	E_{dist}-malonate	E_{dist}-styrene	E_{dist}-catalyst	E_{dist}-total	E_{int}	$E_{IRCpath}$*
2.670	2.7	3.0	17.6	23.3	-87.7	-2832.538195
2.633	2.8	3.4	17.6	23.7	-88.1	-2832.538023
2.589	3.0	3.8	17.6	24.5	-88.6	-2832.537816
2.542	3.3	4.3	17.6	25.2	-89.1	-2832.537573
2.492	3.5	5.0	17.6	26.2	-89.8	-2832.537304
2.436	3.9	6.0	17.6	27.6	-91.0	-2832.537027
2.379	4.4	7.3	17.7	29.4	-92.5	-2832.536770
2.321	4.9	8.7	17.7	31.3	-94.2	-2832.536576
2.260	5.7	10.7	17.8	34.1	-96.9	-2832.536500
2.199	6.6	12.9	17.8	37.3	-100.1	-2832.536593
2.137	7.5	15.4	17.9	40.8	-103.7	-2832.536901
2.075	8.5	18.4	17.9	44.8	-108.1	-2832.537470
2.012	9.7	21.6	18.0	49.3	-113.2	-2832.538433
1.948	11.2	25.2	18.0	54.4	-119.3	-2832.539884
1.885	12.7	29.2	18.1	60.0	-126.0	-2832.541766
1.822	14.2	33.0	18.2	65.4	-132.9	-2832.543871
1.762	15.6	36.6	18.3	70.6	-139.4	-2832.545925
1.708	16.8	39.6	18.3	74.8	-144.8	-2832.547638

*Given in Hartree at B3LYP-D3/6-31G(d)-SMD(Toluene)

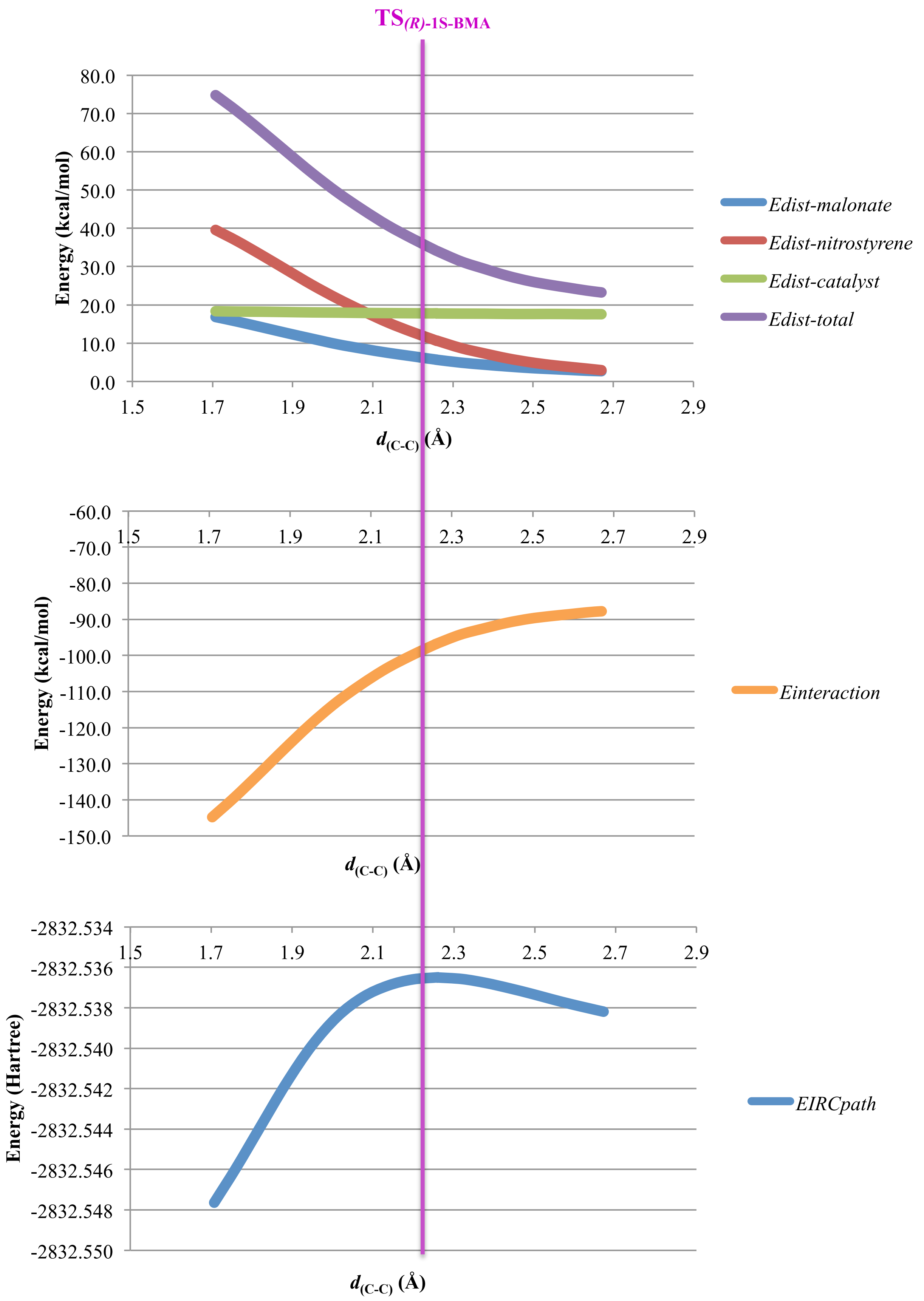

Figure S1.25 | Distortion/interaction analysis along the IRC for ***(R)*-TS$_{\mathbf{1S\text{-}BMA}}$** [B3LYP-D3/6-311+G(d,p)-SMD(Toluene)//B3LYP-D3/6-31G(d)-SMD(Toluene)].

Table S1.14 | Distortion/Interaction Analysis Along the IRC for ***(R)*-TS$_{1S\text{-}BMB}$**

$d_{(C\text{-}C)}$ (Å)	E_{dist}-malonate	E_{dist}-styrene	E_{dist}-catalyst	E_{dist}-total	E_{int}	$E_{IRCpath}$*
2.774	1.7	2.0	9.3	13.0	-88.4	-2832.559002
2.741	1.8	2.3	9.3	13.3	-88.6	-2832.558819
2.705	1.9	2.5	9.3	13.7	-88.7	-2832.558613
2.665	2.0	2.8	9.3	14.1	-89.0	-2832.558381
2.621	2.2	3.2	9.4	14.7	-89.4	-2832.558120
2.575	2.4	3.6	9.4	15.4	-89.7	-2832.557832
2.527	2.7	4.1	9.4	16.2	-90.3	-2832.557523
2.476	3.0	4.9	9.5	17.3	-91.1	-2832.557206
2.423	3.4	5.7	9.5	18.6	-92.2	-2832.556898
2.366	3.8	7.0	9.6	20.4	-93.7	-2832.556629
2.310	4.4	8.4	9.6	22.4	-95.4	-2832.556433
2.252	5.0	10.1	9.7	24.8	-97.8	-2832.556359
2.194	5.8	12.1	9.8	27.7	-100.6	-2832.556446
2.135	6.6	14.3	9.9	30.7	-103.8	-2832.556729
2.076	7.6	17.0	10.0	34.7	-108.0	-2832.557251
2.015	8.7	20.0	10.1	38.8	-112.7	-2832.558138
1.954	9.9	23.3	10.2	43.4	-118.3	-2832.559486
1.893	11.2	26.9	10.4	48.6	-124.6	-2832.561259
1.833	12.6	30.6	10.5	53.7	-131.1	-2832.563284
1.774	13.9	34.2	10.7	58.8	-137.6	-2832.565320
1.719	15.1	37.1	10.8	63.0	-142.9	-2832.567102
1.673	16.2	39.3	10.9	66.5	-147.2	-2832.568392
1.644	16.8	40.1	11.1	68.0	-149.3	-2832.569200

*Given in Hartree at B3LYP-D3/6-31G(d)-SMD(Toluene)

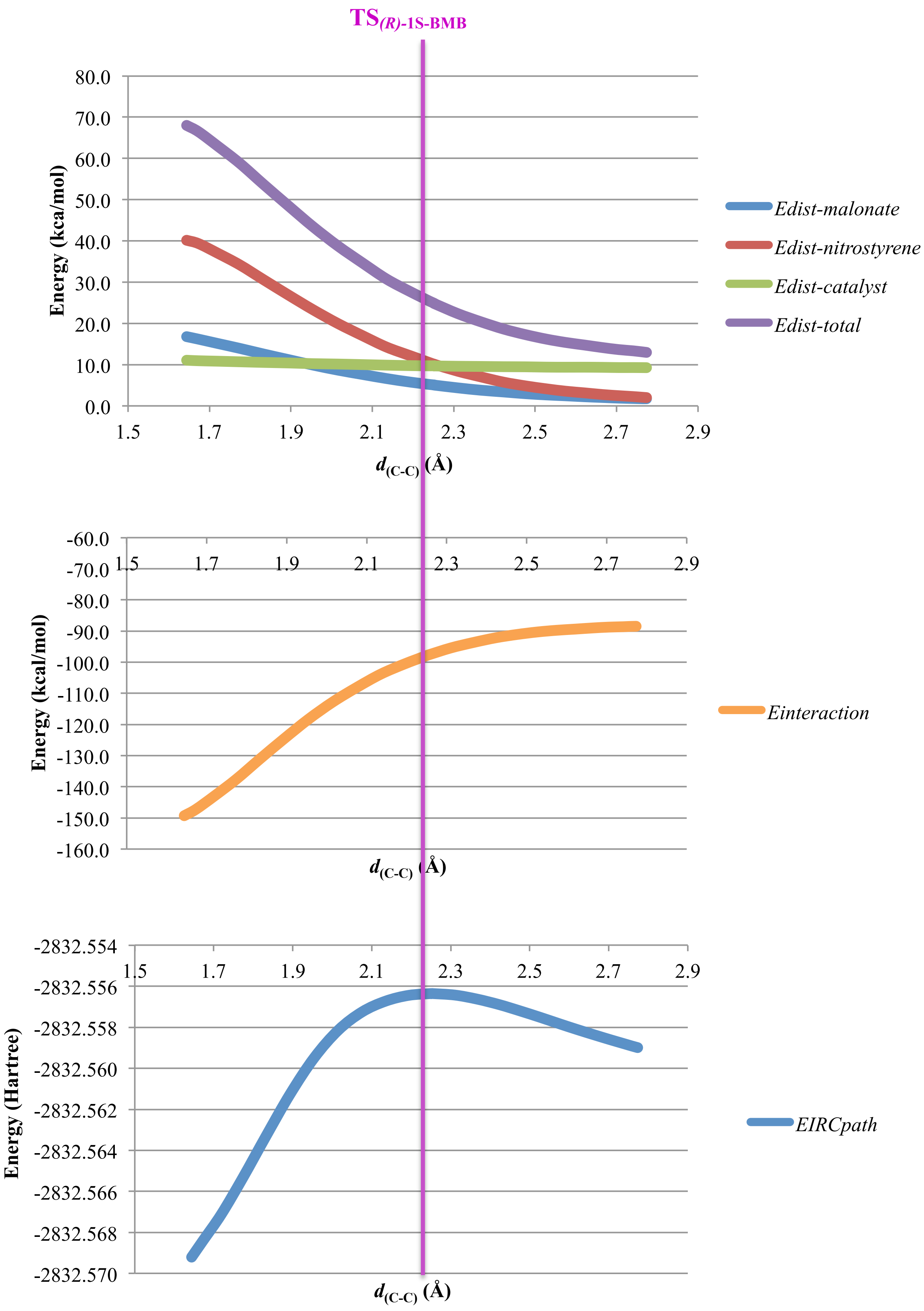

Figure S1.26 | Distortion/interaction analysis along the IRC for ***(R)*-TS$_{\mathbf{1S\text{-}BMB}}$** [B3LYP-D3/6-311+G(d,p)-SMD(Toluene)//B3LYP-D3/6-31G(d)-SMD(Toluene)].

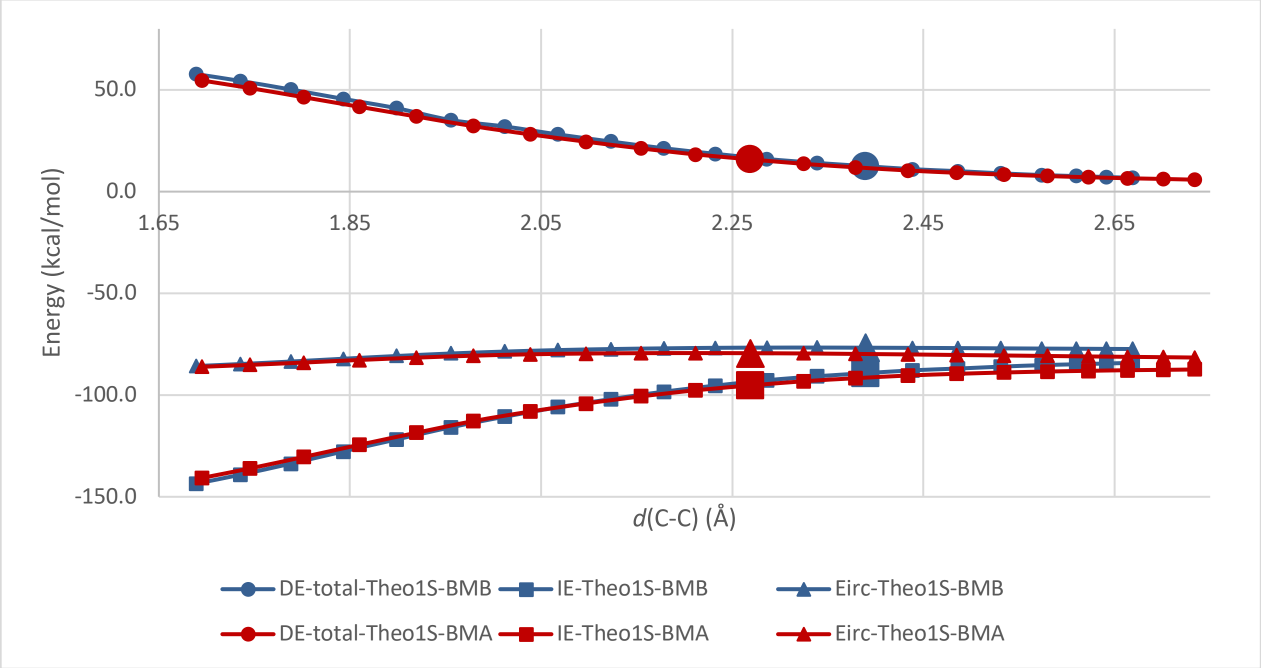

Figure S1.27 | Distortion/interaction diagram along the IRC for **TS**$_{\mathbf{Theo1S\text{-}BMA}}$ vs **TS**$_{\mathbf{Theo1S\text{-}BMB}}$ [B3LYP-D3/6-311+G(d,p)-SMD(Toluene)//B3LYP-D3/6-31G(d)-SMD(Toluene)]. (Larger markers indicate the location of TS)

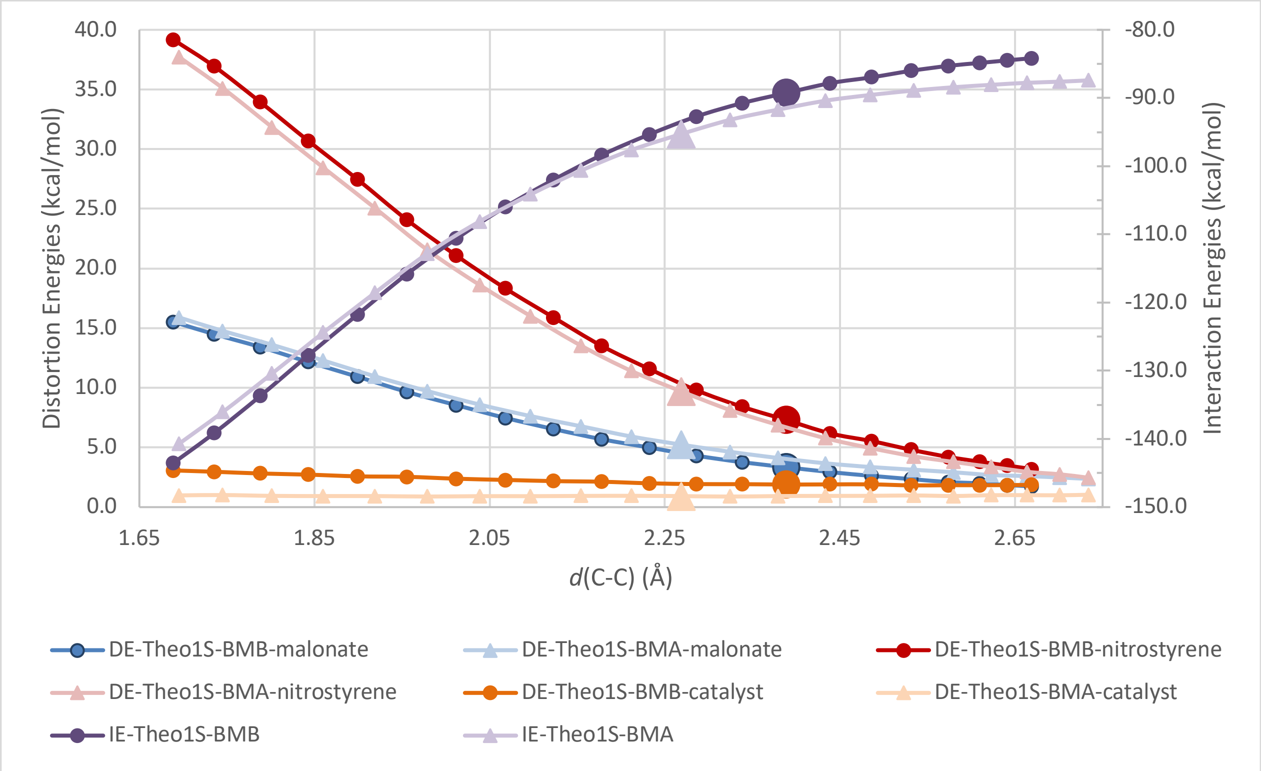

Figure S1.28 | Individual distortion (DE) and interaction (IE) energies along the IRC for **TS**$_{\mathbf{Theo1S\text{-}BMA}}$ vs **TS**$_{\mathbf{Theo1S\text{-}BMB}}$ [B3LYP-D3/6-311+G(d,p)-SMD(Toluene)//B3LYP-D3/6-31G(d)-SMD(Toluene)]. (Larger markers indicate the location of TS)

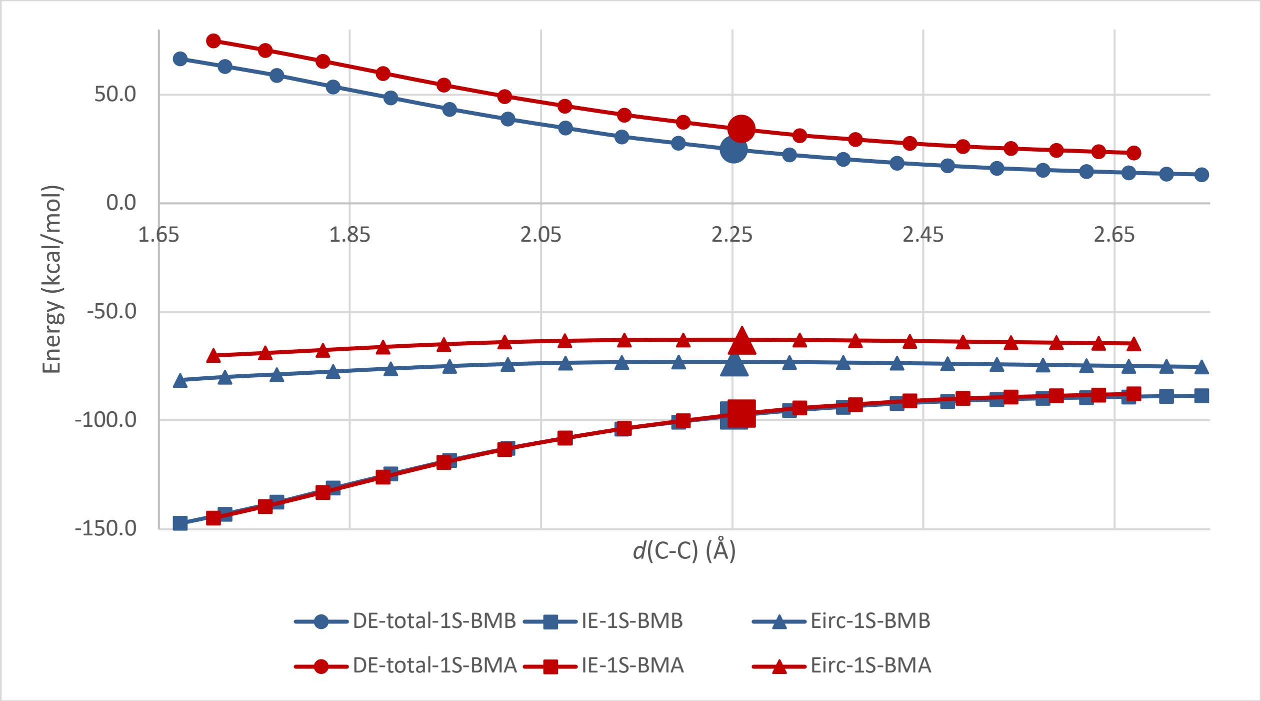

Figure S1.29 | Distortion/interaction diagram along the IRC for ***(R)*-TS$_{1S\text{-}BMA}$** vs ***(R)*-TS$_{1S\text{-}BMB}$** [B3LYP-D3/6-311+G(d,p)-SMD(Toluene)//B3LYP-D3/6-31G(d)-SMD(Toluene)]. (Larger markers indicate the location of TS)

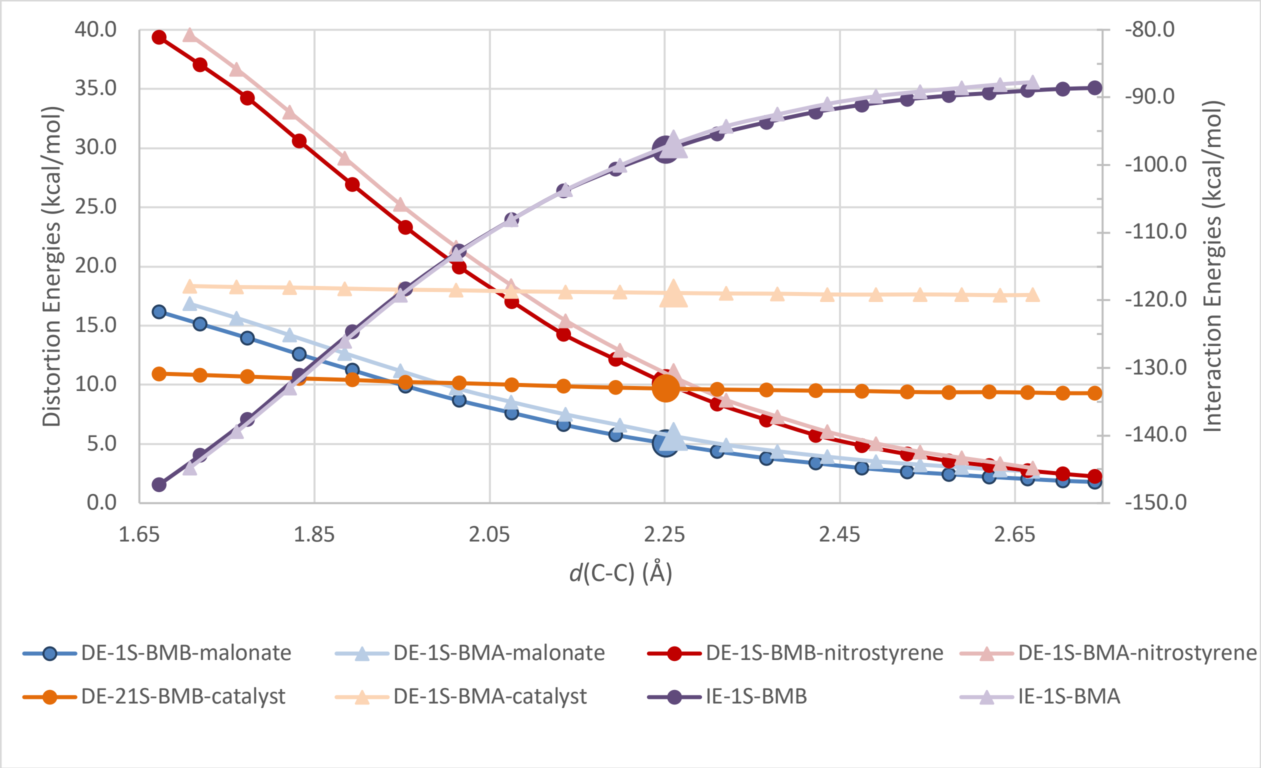

Figure S1.30 | Individual distortion (DE) and interaction (IE) energies along the IRC for ***(R)*-TS$_{1S\text{-}BMA}$** vs ***(R)*-TS$_{1S\text{-}BMB}$** [B3LYP-D3/6-311+G(d,p)-SMD(Toluene)//B3LYP-D3/6-31G(d)-SMD(Toluene)]. (Larger markers indicate the location of TS)

1.3. NATURAL ENERGY DECOMPOSITION ANALYSIS

Natural energy decomposition analysis[14-17] involves partitioning the energy of molecular interactions into various components. It examines electrical interaction (EL), charge transfer (CT), and core repulsion (CORE) contributions within self-consistent field (SCF) wavefunctions. The electrical term consists of classical electrostatic (ES) and polarization interactions (POL+SE), both explained by electrodynamics. SE signifies the linear response self energy (energy penalty) of polarization. CORE contribution arises mainly from intermolecular exchange interactions (EX) and deformation (DEF); DEF refers to the energy required to alter a fragment's wavefunction in the presence of other fragments. In density functional theory (DFT), EX is replaced with the exchange-correlation interaction (XC). The total interaction energy is then given by the sum of EL, CT, and CORE terms.

Natural energy decomposition analysis was performed using NBO 7.0[18] to analyze the trends in interaction energies. Stabilizing interactions in ***(R)*-TS$_{2O}$**, ***(R)*-TS$_{2S}$**, and ***(R)*-TS$_{1S\text{-}BMB}$** are shown in Figure S.1.31, Figure S1.32 and Figure S1.33 respectively. Analysis results are summarized in Tables S.15 and S.16. Components of total interaction energy between the two fragments (catalyst and TS) evaluated at the B3LYP-D3/6-311+G(d,p)(gas)//B3LYP-D3/6-31G(d)-SMD(Toluene) level are given in Table S1.17.

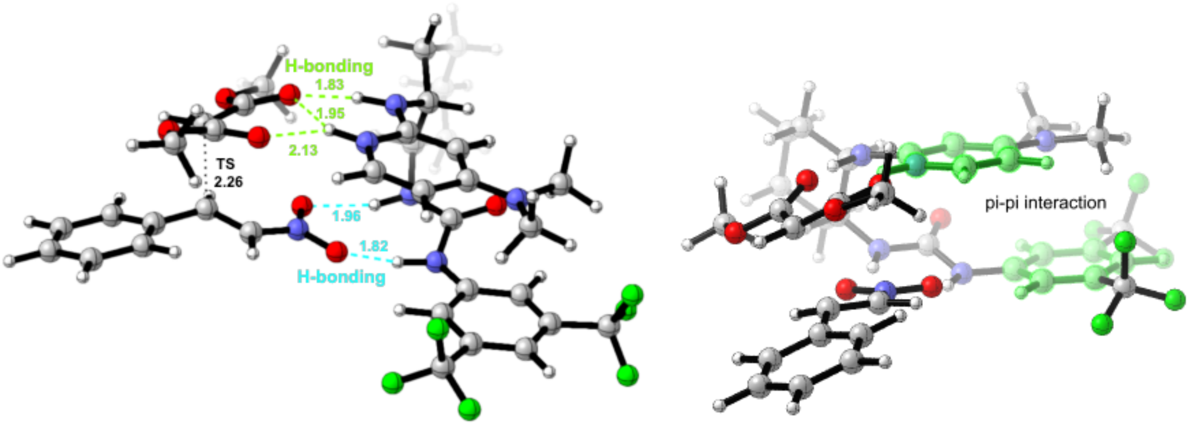

Figure S1.31 | Stabilizing interactions in ***(R)*-TS$_{2O}$** [B3LYP-D3/6-311+G(d,p)-SMD(Toluene)//B3LYP-D3/6-31G(d)-SMD(Toluene)].

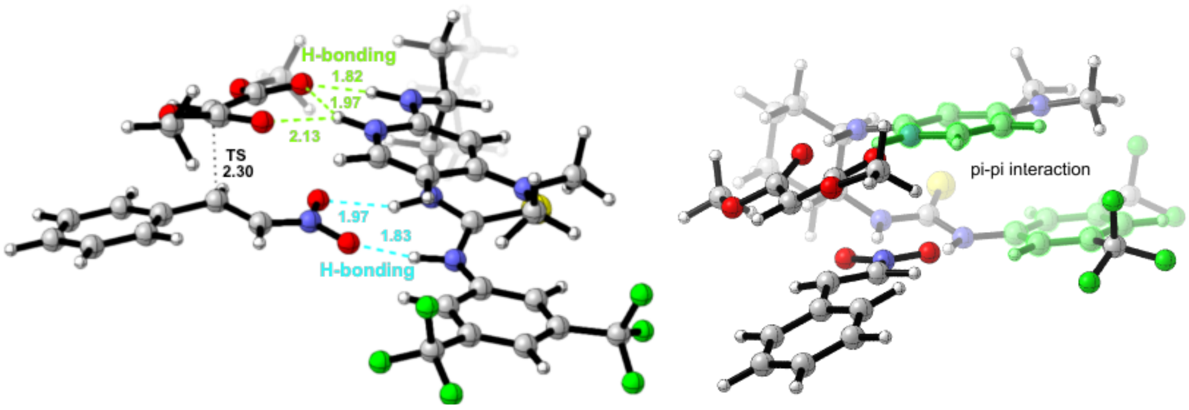

Figure S1.32 | Stabilizing interactions in ***(R)*-TS$_{2S}$** [B3LYP-D3/6-311+G(d,p)-SMD(Toluene)//B3LYP-D3/6-31G(d)-SMD(Toluene)].

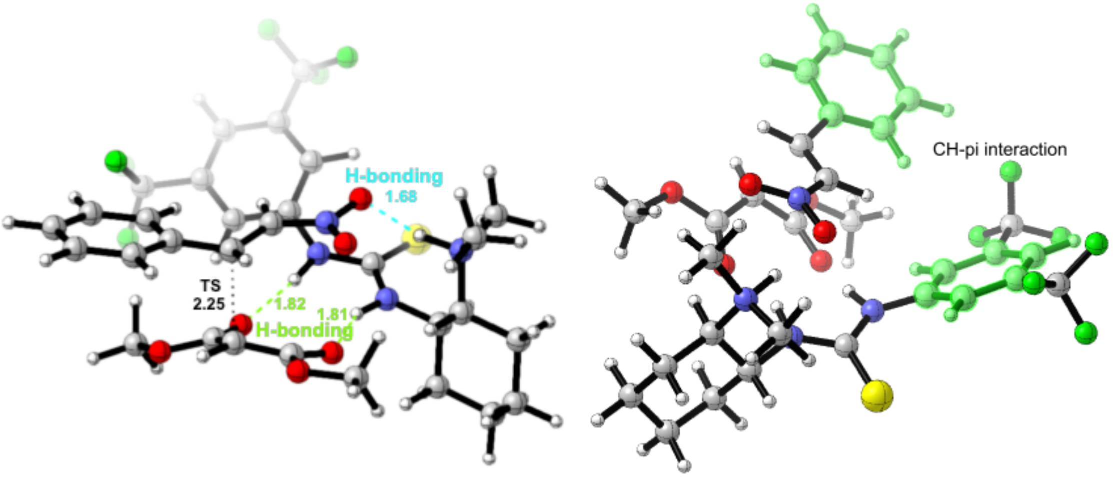

Figure S1.33 | Stabilizing interactions in ***(R)*-TS$_{1S\text{-}BMB}$** [B3LYP-D3/6-311+G(d,p)-SMD(Toluene)//B3LYP-D3/6-31G(d)-SMD(Toluene)].

Table S1.15 | Natural Energy Decomposition Analysis for Theozymes [B3LYP-D3/6-311+G(d,p)(gas)//B3LYP-D3/6-31G(d)-SMD(Toluene)]

	***Theo*-TS$_{1S}$**	***Theo*-TS$_{2S}$**	***Theo*-TS$_{2O}$**	***Theo*-TS$_{1O}$**	***Theo*-TS$_{1S}$ BMB**	***Theo*-TS$_{2S}$ BMB**
Electrical (ES+POL+SE)	-171.5	-171.8	-167.5	-164.3	-153.5	-161.2
Charge Transfer (CT)	-80.8	-87.8	-82.7	-70.3	-85.4	-81.8
(ES+POL+SE+CT)	-252.3	-259.6	-250.2	-234.6	-238.9	-243.0
Core (XC+DEF-SE)	113.3	124.2	117.8	98.9	112.8	111.8
Total Interaction (E)	-138.9	-135.5	-132.4	-135.6	-126.1	-131.2

Table S1.16 | Natural Energy Decomposition Analysis for Full Systems [B3LYP-D3/6-311+G(d,p)(gas)//B3LYP-D3/6-31G(d)-SMD(Toluene)]

	***(R)*-TS$_{1S}$ BMB**	***(R)*-TS$_{2S}$ BMA**	***(R)*-TS$_{2O}$ BMA**	***(S)*-TS$_{1S}$ BMB**	***(S)*-TS$_{2S}$ BMA**	***(S)*-TS$_{2O}$ BMA**	***(R)*-TS$_{1S}$ BMA**	***(R)*-TS$_{2S}$ BMB**	***(R)*-TS$_{2O}$ BMB**
Electrical (ES+POL+SE)	-174.6	-169.8	-165.5	-170.8	-166.0	-161.3	-176.5	-163.9	-159.8
Charge Transfer (CT)	-106.5	-89.1	-85.0	-108.9	-86.8	-80.7	-86.4	-106.0	-100.1
(ES+POL+SE+CT)	-281.0	-258.9	-250.5	-279.8	-252.8	-242.0	-262.9	-269.9	-259.9
Core (XC+DEF-SE)	142.1	124.8	118.4	142.1	120.3	111.6	121.7	140.4	132.5
Total Interaction (E)	-138.9	-134.0	-132.1	-137.7	-132.5	-130.3	-141.2	-129.5	-127.4

Table S1.17 | Components of Total Interaction Energy between the Catalysts and Transition States [B3LYP-D3/6-311+G(d,p) (gas)//B3LYP-D3/6-31G(d)-SMD(Toluene)]

TS_{Theo1O}

```
Natural Energy Decomposition Analysis (Summary):

                                                               Component
                    Energy(wfn)        Energy(wfn)            (kcal/mol)
 -------------------------------------------------------------------------------
 C19H32N4O7    -1489.3004537(scf) -1489.1884607(loc)      CT =  -70.277
                                                          ES = -131.597
                                                         POL =  -70.058
                                                          XC =  -37.682
 1. C6H18N3O(+  -478.7917653(def)  -478.8966268(rel) DEF(SE) =   65.802( 22.111)
 2. C13H14NO6( -1010.0152872(def) -1010.1876627(rel) DEF(SE) =  108.167( 15.227)
                                                               ---------
                                                           E = -135.645


 Electrical (ES+POL+SE) :   -164.318
   Charge Transfer (CT) :    -70.277
       Core (XC+DEF-SE) :     98.949
                        ------------
  Total Interaction (E) :   -135.645
```

TS_{Theo1S}

```
Natural Energy Decomposition Analysis (Summary):

                                                               Component
                    Energy(wfn)        Energy(wfn)            (kcal/mol)
 -------------------------------------------------------------------------------
 C19H32N4O6S   -1812.2589089(scf) -1812.1302127(loc)      CT =  -80.758
                                                          ES = -138.058
                                                         POL =  -71.911
                                                          XC =  -38.258
 1. C6H18N3S(+  -801.7358937(def)  -801.8488255(rel) DEF(SE) =   70.866( 23.330)
 2. C13H14NO6( -1009.9987432(def) -1010.1886753(rel) DEF(SE) =  119.184( 15.144)
                                                               ---------
                                                           E = -138.936


 Electrical (ES+POL+SE) :   -171.496
   Charge Transfer (CT) :    -80.758
       Core (XC+DEF-SE) :    113.318
                        ------------
  Total Interaction (E) :   -138.936
```

$TS_{Theo1S-BMB}$

```
Natural Energy Decomposition Analysis (Summary):

                                                               Component
                    Energy(wfn)        Energy(wfn)            (kcal/mol)
 -------------------------------------------------------------------------------
 C19H32N4O6S   -1812.2571784(scf) -1812.1211102(loc)      CT =  -85.384
                                                          ES = -125.085
                                                         POL =  -61.610
                                                          XC =  -34.547
 1. C6H18N3S(+  -801.7533343(def)  -801.8664636(rel) DEF(SE) =   70.990( 22.508)
 2. C13H14NO6( -1010.0152037(def) -1010.1897292(rel) DEF(SE) =  109.516( 10.670)
                                                               ---------
                                                           E = -126.120


 Electrical (ES+POL+SE) :   -153.517
   Charge Transfer (CT) :    -85.384
       Core (XC+DEF-SE) :    112.781
                        ------------
  Total Interaction (E) :   -126.120
```

TS_{Theo2O}

```
Natural Energy Decomposition Analysis (Summary):

                                                                      Component
                    Energy(wfn)        Energy(wfn)          (kcal/mol)
 -------------------------------------------------------------------------------
 C22H31N5O7    -1657.8338148(scf) -1657.7020262(loc)      CT =  -82.699
                                                          ES = -133.028
                                                         POL =  -74.608
                                                          XC =  -38.409
 1. C9H17N4O(+  -647.3146743(def)  -647.4350638(rel) DEF(SE) =   75.546( 24.915)
 2. C13H14NO6( -1009.9952549(def) -1010.1877857(rel) DEF(SE) =  120.815( 15.249)
                                                              ---------
                                                           E = -132.383


 Electrical (ES+POL+SE) :   -167.472
   Charge Transfer (CT) :    -82.699
       Core (XC+DEF-SE) :    117.788
                        ------------
  Total Interaction (E) :   -132.383
```

TS_{Theo2S}

```
Natural Energy Decomposition Analysis (Summary):

                                                                      Component
                    Energy(wfn)        Energy(wfn)          (kcal/mol)
 -------------------------------------------------------------------------------
 C22H31N5O6S   -1980.7931436(scf) -1980.6532033(loc)      CT =  -87.814
                                                          ES = -136.147
                                                         POL =  -77.636
                                                          XC =  -38.713
 1. C9H17N4S(+  -970.2640387(def)  -970.3893436(rel) DEF(SE) =   78.630( 26.193)
 2. C13H14NO6( -1009.9867869(def) -1010.1879446(rel) DEF(SE) =  126.228( 15.776)
                                                              ---------
                                                           E = -135.451


 Electrical (ES+POL+SE) :   -171.814
   Charge Transfer (CT) :    -87.814
       Core (XC+DEF-SE) :    124.176
                        ------------
  Total Interaction (E) :   -135.451
```

$TS_{Theo2S-BMB}$

```
Natural Energy Decomposition Analysis (Summary):

                                                                      Component
                    Energy(wfn)        Energy(wfn)          (kcal/mol)
 -------------------------------------------------------------------------------
 C22H31N5O6S   -1980.7892955(scf) -1980.6588896(loc)      CT =  -81.831
                                                          ES = -127.448
                                                         POL =  -74.139
                                                          XC =  -34.836
 1. C9H17N4S(+  -970.2761206(def)  -970.3967122(rel) DEF(SE) =   75.672( 26.868)
 2. C13H14NO6( -1010.0060050(def) -1010.1835401(rel) DEF(SE) =  111.405( 13.540)
                                                              ---------
                                                           E = -131.177


 Electrical (ES+POL+SE) :   -161.179
   Charge Transfer (CT) :    -81.831
       Core (XC+DEF-SE) :    111.833
                        ------------
  Total Interaction (E) :   -131.177
```

***(R)*-TS$_{1S\text{-}BMB}$**

```
Natural Energy Decomposition Analysis (Summary):

                                                                   Component
                   Energy(wfn)        Energy(wfn)          (kcal/mol)
 -------------------------------------------------------------------------------
 C30H36F6N4O6S -2833.2658378(scf) -2833.0961657(loc)      CT = -106.471
                                                          ES = -134.150
                                                         POL =  -88.978
                                                          XC =  -50.847
 1. C17H22F6N3 -1822.7014663(def) -1822.8565977(rel) DEF(SE) =   97.346( 30.611)
 2. C13H14NO6( -1009.9580921(def) -1010.1878760(rel) DEF(SE) =  144.192( 17.959)
                                                               ---------
                                                           E = -138.908


 Electrical (ES+POL+SE) :   -174.558
   Charge Transfer (CT) :   -106.471
       Core (XC+DEF-SE) :    142.121
                        ------------
  Total Interaction (E) :   -138.908
```

***(R)*-TS$_{2S}$**

```
Natural Energy Decomposition Analysis (Summary):

                                                                   Component
                   Energy(wfn)        Energy(wfn)          (kcal/mol)
 -------------------------------------------------------------------------------
 C35H40F6N6O6S -3135.8315871(scf) -3135.6895747(loc)      CT =  -89.114
                                                          ES = -130.426
                                                         POL =  -86.646
                                                          XC =  -40.459
 1. C22H26F6N5 -2125.2973212(def) -2125.4324045(rel) DEF(SE) =   84.766( 30.861)
 2. C13H14NO6( -1009.9818517(def) -1010.1855720(rel) DEF(SE) =  127.836( 16.439)
                                                               ---------
                                                           E = -134.043


 Electrical (ES+POL+SE) :   -169.772
   Charge Transfer (CT) :    -89.114
       Core (XC+DEF-SE) :    124.843
                        ------------
  Total Interaction (E) :   -134.043
```

***(R)*-TS$_{2O}$**

```
Natural Energy Decomposition Analysis (Summary):

                                                                   Component
                   Energy(wfn)        Energy(wfn)          (kcal/mol)
 -------------------------------------------------------------------------------
 C35H40F6N6O7  -2812.8839614(scf) -2812.7484412(loc)      CT =  -85.040
                                                          ES = -128.361
                                                         POL =  -81.487
                                                          XC =  -38.123
 1. C22H26F6N5 -1802.3603419(def) -1802.4879997(rel) DEF(SE) =   80.106( 29.276)
 2. C13H14NO6( -1009.9929310(def) -1010.1853896(rel) DEF(SE) =  120.770( 15.097)
                                                               ---------
                                                           E = -132.136


 Electrical (ES+POL+SE) :   -165.475
   Charge Transfer (CT) :    -85.040
       Core (XC+DEF-SE) :    118.379
                        ------------
  Total Interaction (E) :   -132.136
```

<table>
<tr>
<td>(S)-TS$_{1S-}$
BMB</td>
<td><pre>
Natural Energy Decomposition Analysis (Summary):

                                                               Component
                   Energy(wfn)   Energy(wfn)   (kcal/mol)

 C30H36F6N4O6S -2833.2611313(scf) -2833.0875449(loc)   CT = -108.927
                                                          ES = -133.237
                                                         POL = -83.348
                                                          XC = -48.804
 1. C17H22F6N3 -1822.7031492(def) -1822.8564548(rel) DEF(SE) =   96.201(29.524)
 2. C13H14NO6(-1009.9614707(def) -1010.1852296(rel) DEF(SE) = 140.411(16.226)

                                                           E = -137.705

 Electrical (ES+POL+SE) :   -170.835
   Charge Transfer (CT) :   -108.927
       Core (XC+DEF-SE) :   142.057

  Total Interaction (E) :   -137.705
</pre></td>
</tr>
<tr>
<td>(S)-TS$_{2S}$</td>
<td><pre>
Natural Energy Decomposition Analysis (Summary):

                                                               Component
                   Energy(wfn)   Energy(wfn)   (kcal/mol)

 C35H40F6N6O6S -3135.8270895(scf) -3135.6887817(loc)   CT = -86.789
                                                          ES = -127.929
                                                         POL = -83.918
                                                          XC = -38.863
 1. C22H26F6N5 -2125.3026413(def) -2125.4323438(rel) DEF(SE) =   81.390(30.006)
 2. C13H14NO6(-1009.9866066(def) -1010.1835622(rel) DEF(SE) = 123.591(15.856)

                                                           E = -132.520

 Electrical (ES+POL+SE) :   -165.985
   Charge Transfer (CT) :   -86.789
       Core (XC+DEF-SE) :   120.255

  Total Interaction (E) :   -132.520
</pre></td>
</tr>
<tr>
<td>(S)-TS$_{2O}$</td>
<td><pre>
Natural Energy Decomposition Analysis (Summary):

                                                               Component
                   Energy(wfn)   Energy(wfn)   (kcal/mol)

 C35H40F6N6O7 -2812.8788038(scf) -2812.7502217(loc)   CT = -80.686
                                                          ES = -125.402
                                                         POL = -78.860
                                                          XC = -36.297
 1. C22H26F6N5 -1802.3674082(def) -1802.4880685(rel) DEF(SE) =   75.715(28.345)
 2. C13H14NO6(-1009.9994568(def) -1010.1830356(rel) DEF(SE) = 115.197(14.622)

                                                           E = -130.334

 Electrical (ES+POL+SE) :   -161.295
   Charge Transfer (CT) :   -80.686
       Core (XC+DEF-SE) :   111.648

  Total Interaction (E) :   -130.334
</pre></td>
</tr>
</table>

***(R)*-TS$_{1S}$-BMA**

```
Natural Energy Decomposition Analysis (Summary):

                                                               Component
                   Energy(wfn)        Energy(wfn)         (kcal/mol)
 -------------------------------------------------------------------------------
 C30H36F6N4O6S -2833.2501289(scf) -2833.1124603(loc)      CT =  -86.388
                                                          ES = -137.920
                                                         POL =  -84.144
                                                          XC =  -39.781
 1. C17H22F6N3 -1822.7126428(def) -1822.8421934(rel) DEF(SE) =   81.294( 29.310)
 2. C13H14NO6( -1009.9825410(def) -1010.1829684(rel) DEF(SE) =  125.770( 16.255)
                                                               ---------
                                                           E = -141.169


 Electrical (ES+POL+SE) :   -176.499
   Charge Transfer (CT) :    -86.388
       Core (XC+DEF-SE) :    121.719
                          ------------
  Total Interaction (E) :   -141.169
```

***(R)*-TS$_{2S}$-BMB**

```
Natural Energy Decomposition Analysis (Summary):

                                                               Component
                   Energy(wfn)        Energy(wfn)         (kcal/mol)
 -------------------------------------------------------------------------------
 C35H40F6N6O6S -3135.8083236(scf) -3135.6394163(loc)      CT = -105.991
                                                          ES = -125.148
                                                         POL =  -85.225
                                                          XC =  -49.956
 1. C13H14NO6( -1009.9671137(def) -1010.1898670(rel) DEF(SE) =  139.780( 15.452)
 2. C22H26F6N5 -2125.2574433(def) -2125.4121018(rel) DEF(SE) =   97.050( 31.058)
                                                               ---------
                                                           E = -129.490


 Electrical (ES+POL+SE) :   -163.862
   Charge Transfer (CT) :   -105.991
       Core (XC+DEF-SE) :    140.363
                          ------------
  Total Interaction (E) :   -129.490
```

***(R)*-TS$_{2O}$-BMB**

```
Natural Energy Decomposition Analysis (Summary):

                                                               Component
                   Energy(wfn)        Energy(wfn)         (kcal/mol)
 -------------------------------------------------------------------------------
 C35H40F6N6O7  -2812.8558010(scf) -2812.6962774(loc)      CT = -100.103
                                                          ES = -122.788
                                                         POL =  -81.312
                                                          XC =  -46.415
 1. C13H14NO6( -1009.9818843(def) -1010.1890621(rel) DEF(SE) =  130.006( 14.179)
 2. C22H26F6N5 -1802.3151713(def) -1802.4637335(rel) DEF(SE) =   93.224( 30.113)
                                                               ---------
                                                           E = -127.388


 Electrical (ES+POL+SE) :   -159.808
   Charge Transfer (CT) :   -100.103
       Core (XC+DEF-SE) :    132.523
                          ------------
  Total Interaction (E) :   -127.388
```

1.4. CATALYST CONFORMER LIBRARIES

Table S1.18 | Absolute Energies and Energy Corrections for Conformer Library of **2O** [B3LYP-D3/6-31G(d)-SMD(Toluene)].

Conformer	SCF Energy	zero point correction	Enthalpy correction	Free energy Correction	Quasiharmonic correction	Gibbs Free Energy (Hartree)	Relative Gibbs Free Energy (kcal/mol)
Cat 2O_conf1	-1801.576147	0.467899	0.499984	0.40472	0.411644	-1801.171427	0.00
Cat 2O_conf2	-1801.576611	0.468017	0.500027	0.405237	0.411763	-1801.171374	0.03
Cat 2O_conf3	-1801.576612	0.468017	0.500027	0.405246	0.411765	-1801.171366	0.04
Cat 2O_conf4	-1801.576372	0.468128	0.500078	0.405165	0.411991	-1801.171207	0.14
Cat 2O_conf5	-1801.576371	0.468128	0.500077	0.405188	0.411989	-1801.171183	0.15
Cat 2O_conf6	-1801.576371	0.468129	0.500078	0.405193	0.411992	-1801.171178	0.16
Cat 2O_conf7	-1801.576370	0.468124	0.500074	0.405209	0.411984	-1801.171161	0.17
Cat 2O_conf8	-1801.574444	0.468019	0.500099	0.404057	0.411721	-1801.170387	0.65
Cat 2O_conf9	-1801.574555	0.468025	0.500099	0.404174	0.41172	-1801.170381	0.66
Cat 2O_conf10	-1801.566257	0.467214	0.499985	0.398443	0.410564	-1801.167814	2.27
Cat 2O_conf11	-1801.566257	0.467218	0.499985	0.398481	0.410563	-1801.167776	2.29
Cat 2O_conf12	-1801.570294	0.467644	0.499943	0.402701	0.411401	-1801.167593	2.41
Cat 2O_conf13	-1801.572715	0.468334	0.500208	0.405379	0.412484	-1801.167336	2.57
Cat 2O_conf14	-1801.572715	0.468339	0.500209	0.405404	0.41249	-1801.167311	2.58
Cat 2O_conf15	-1801.566332	0.467228	0.499979	0.399102	0.410561	-1801.16723	2.63
Cat 2O_conf16	-1801.566172	0.467219	0.499972	0.399054	0.410534	-1801.167118	2.70
Cat 2O_conf17	-1801.571423	0.468052	0.500060	0.405050	0.411963	-1801.166373	3.17
Cat 2O_conf18	-1801.570507	0.468358	0.500255	0.405206	0.412172	-1801.165301	3.84
Cat 2O_conf19	-1801.570507	0.46836	0.500257	0.405212	0.412176	-1801.165295	3.85
Cat 2O_conf20	-1801.564162	0.467331	0.499995	0.400029	0.410611	-1801.164133	4.58
Cat 2O_conf21	-1801.568194	0.468439	0.500227	0.404761	0.412454	-1801.163433	5.02
Cat 2O_conf22	-1801.565339	0.467394	0.499689	0.402711	0.402711	-1801.162628	5.52
Cat 2O_conf23	-1801.565554	0.467419	0.499665	0.403144	0.411081	-1801.16241	5.66
Cat 2O_conf24	-1801.567783	0.468214	0.499285	0.405742	0.412972	-1801.162041	5.89
Cat 2O_conf25	-1801.565245	0.467545	0.499763	0.403455	0.411241	-1801.16179	6.05
Cat 2O_conf26	-1801.564265	0.46683	0.499225	0.402644	0.410413	-1801.161621	6.15
Cat 2O_conf27	-1801.565304	0.467653	0.499828	0.403749	0.411364	-1801.161555	6.19
Cat 2O_conf28	-1801.565305	0.467655	0.49983	0.403759	0.411367	-1801.161546	6.20
Cat 2O_conf29	-1801.566197	0.468455	0.500258	0.405283	0.412738	-1801.160914	6.60
Cat 2O_conf30	-1801.564617	0.467733	0.499864	0.403923	0.411544	-1801.160694	6.74
Cat 2O_conf31	-1801.564617	0.467734	0.499861	0.40395	0.411548	-1801.160667	6.75

Cat 2O_conf32	-1801.563986	0.467631	0.499771	0.403558	0.41145	-1801.160428	6.90
Cat 2O_conf33	-1801.563987	0.467633	0.499771	0.403564	0.411453	-1801.160423	6.91
Cat 2O_conf34	-1801.564016	0.467712	0.499841	0.403799	0.411538	-1801.160217	7.03
Cat 2O_conf35	-1801.563705	0.468394	0.500313	0.403532	0.412587	-1801.160173	7.06
Cat 2O_conf36	-1801.563704	0.468402	0.500316	0.403573	0.412598	-1801.160131	7.09
Cat 2O_conf37	-1801.563783	0.468381	0.500296	0.403836	0.412526	-1801.159947	7.20
Cat 2O_conf38	-1801.563783	0.468383	0.500298	0.403845	0.412529	-1801.159938	7.21
Cat 2O_conf39	-1801.557177	0.466727	0.499535	0.397351	0.410028	-1801.159826	7.28
Cat 2O_conf40	-1801.557517	0.466548	0.499444	0.398025	0.409628	-1801.159492	7.49
Cat 2O_conf41	-1801.557517	0.466548	0.499444	0.398056	0.409622	-1801.159461	7.51
Cat 2O_conf42	-1801.557517	0.466552	0.499444	0.398068	0.409631	-1801.159449	7.52
Cat 2O_conf43	-1801.557188	0.466701	0.49953	0.397872	0.409947	-1801.159316	7.60
Cat 2O_conf44	-1801.557476	0.466575	0.49947	0.398323	0.409634	-1801.159153	7.70
Cat 2O_conf45	-1801.557476	0.46658	0.499471	0.398349	0.409639	-1801.159127	7.72
Cat 2O_conf46	-1801.557476	0.466585	0.499474	0.398366	0.409642	-1801.15911	7.73
Cat 2O_conf47	-1801.560754	0.467312	0.499696	0.401862	0.410973	-1801.158892	7.87
Cat 2O_conf48	-1801.562205	0.467997	0.500142	0.403441	0.411672	-1801.158764	7.95
Cat 2O_conf49	-1801.561031	0.467683	0.499915	0.40268	0.411387	-1801.158351	8.21
Cat 2O_conf50	-1801.554901	0.466948	0.499642	0.398262	0.409885	-1801.156639	9.28
Cat 2O_conf51	-1801.557070	0.466619	0.498447	0.400445	0.410375	-1801.156625	9.29
Cat 2O_conf52	-1801.555529	0.467403	0.499933	0.399727	0.410866	-1801.155802	9.80
Cat 2O_conf53	-1801.555527	0.46737	0.49991	0.399783	0.410808	-1801.155744	9.84
Cat 2O_conf54	-1801.555666	0.467475	0.499964	0.400291	0.410948	-1801.155375	10.07
Cat 2O_conf55	-1801.554446	0.46683	0.498498	0.400986	0.410566	-1801.15346	11.27
Cat 2O_conf56	-1801.549577	0.46641	0.499397	0.396233	0.409405	-1801.153344	11.35
Cat 2O_conf57	-1801.549618	0.46653	0.499462	0.396879	0.409538	-1801.152739	11.73
Cat 2O_conf58	-1801.548285	0.466708	0.499563	0.397058	0.409723	-1801.151227	12.68
Cat 2O_conf59	-1801.550366	0.466656	0.498597	0.399284	0.410468	-1801.151082	12.77
Cat 2O_conf60	-1801.548301	0.466759	0.49959	0.397469	0.409772	-1801.150832	12.92
Cat 2O_conf61	-1801.548349	0.466786	0.499598	0.397717	0.409778	-1801.150632	13.05
Cat 2O_conf62	-1801.548343	0.466494	0.498522	0.398703	0.410244	-1801.14964	13.67

Table S1.19 | Absolute Energies and Energy Corrections for Conformer Library of **2S** [B3LYP-D3/6-31G(d)-SMD(Toluene)].

**Conformer**	**SCF Energy**	**zero point correction**	**Enthalpy correction**	**Free energy Correction**	**Quasiharmonic correction**	**Gibbs Free Energy (Hartree)**	**Relative Gibbs Free Energy (kcal/mol)**
Cat 2S_conf1	-2124.529741	0.465226	0.497577	0.401716	0.408748	-2124.128025	0.00
Cat 2S_conf2	-2124.529741	0.465233	0.49758	0.401745	0.408753	-2124.127996	0.02
Cat 2S_conf3	-2124.52799	0.465398	0.498005	0.400355	0.408747	-2124.127635	0.24
Cat 2S_conf4	-2124.525455	0.465562	0.498106	0.400445	0.408834	-2124.12501	1.89
Cat 2S_conf5	-2124.521481	0.465007	0.497937	0.39705	0.408045	-2124.124431	2.26
Cat 2S_conf6	-2124.521690	0.465252	0.498075	0.397821	0.408343	-2124.123869	2.61
Cat 2S_conf7	-2124.52169	0.465246	0.49807	0.397839	0.408326	-2124.123851	2.62
Cat 2S_conf8	-2124.525977	0.465608	0.497945	0.402204	0.409398	-2124.123773	2.67
Cat 2S_conf9	-2124.526358	0.466092	0.498328	0.402596	0.409534	-2124.123762	2.68
Cat 2S_conf10	-2124.525977	0.46562	0.497948	0.402249	0.409418	-2124.123728	2.70
Cat 2S_conf11	-2124.521534	0.465239	0.498062	0.398019	0.408303	-2124.123515	2.83
Cat 2S_conf12	-2124.521534	0.465243	0.498063	0.398033	0.408307	-2124.123501	2.84
Cat 2S_conf13	-2124.526060	0.465768	0.497988	0.402664	0.409636	-2124.123396	2.90
Cat 2S_conf14	-2124.526063	0.465768	0.497987	0.402668	0.409635	-2124.123395	2.91
Cat 2S_conf15	-2124.520049	0.465131	0.498047	0.396888	0.40823	-2124.123161	3.05
Cat 2S_conf16	-2124.51991	0.465135	0.498026	0.397445	0.408202	-2124.122465	3.49
Cat 2S_conf17	-2124.51991	0.465142	0.498027	0.397561	0.408203	-2124.122349	3.56
Cat 2S_conf18	-2124.522204	0.465649	0.498137	0.400137	0.409063	-2124.122067	3.74
Cat 2S_conf19	-2124.523425	0.465164	0.496881	0.401396	0.409681	-2124.122029	3.76
Cat 2S_conf20	-2124.521794	0.465843	0.498248	0.399799	0.409381	-2124.121995	3.78
Cat 2S_conf21	-2124.522190	0.465581	0.498096	0.400209	0.408932	-2124.121981	3.79
Cat 2S_conf22	-2124.523535	0.463870	0.494347	0.401920	0.409384	-2124.121615	4.02
Cat 2S_conf23	-2124.521741	0.465492	0.49811	0.400328	0.40871	-2124.121413	4.15
Cat 2S_conf24	-2124.52192	0.46585	0.498213	0.400529	0.409362	-2124.121391	4.16
Cat 2S_conf25	-2124.521485	0.465757	0.498124	0.400236	0.409407	-2124.121249	4.25
Cat 2S_conf26	-2124.520967	0.464742	0.496014	0.400195	0.409288	-2124.120772	4.55
Cat 2S_conf27	-2124.521532	0.465921	0.498199	0.40103	0.409585	-2124.120502	4.72
Cat 2S_conf28	-2124.521526	0.465944	0.498211	0.401149	0.40961	-2124.120377	4.80
Cat 2S_conf29	-2124.517723	0.464762	0.497586	0.398313	0.407755	-2124.11941	5.41
Cat 2S_conf30	-2124.519150	0.465173	0.497765	0.399763	0.408509	-2124.119387	5.42
Cat 2S_conf31	-2124.519133	0.465156	0.497748	0.399836	0.408437	-2124.119297	5.48
Cat 2S_conf32	-2124.520417	0.465747	0.498032	0.401187	0.40934	-2124.11923	5.52
Cat 2S_conf33	-2124.516464	0.464783	0.497592	0.397395	0.408028	-2124.119069	5.62
Cat 2S_conf34	-2124.51776	0.464823	0.497615	0.398742	0.407827	-2124.119018	5.65
Cat 2S_conf35	-2124.517872	0.465213	0.497899	0.398938	0.408539	-2124.118934	5.70
Cat 2S_conf36	-2124.517872	0.465214	0.4979	0.39895	0.408539	-2124.118922	5.71
Cat 2S_conf37	-2124.517934	0.465269	0.497938	0.399043	0.40861	-2124.118891	5.73

Cat 2S_conf38	-2124.517934	0.465272	0.497939	0.399055	0.408611	-2124.118879	5.74
Cat 2S_conf39	-2124.516277	0.465096	0.49796	0.39795	0.408302	-2124.118327	6.09
Cat 2S_conf40	-2124.516596	0.465229	0.497972	0.398463	0.408611	-2124.118133	6.21
Cat 2S_conf41	-2124.518662	0.465853	0.498152	0.401895	0.40953	-2124.116767	7.06
Cat 2S_conf42	-2124.516369	0.466009	0.498308	0.399897	0.409938	-2124.116472	7.25
Cat 2S_conf43	-2124.516369	0.466009	0.498309	0.399914	0.409938	-2124.116455	7.26
Cat 2S_conf44	-2124.516369	0.466009	0.498309	0.399934	0.409937	-2124.116435	7.27
Cat 2S_conf45	-2124.516369	0.466009	0.498309	0.399938	0.409938	-2124.116431	7.28
Cat 2S_conf46	-2124.516255	0.465993	0.498293	0.39993	0.40994	-2124.116325	7.34
Cat 2S_conf47	-2124.510638	0.464142	0.497228	0.394545	0.406908	-2124.116093	7.49
Cat 2S_conf48	-2124.515287	0.465246	0.497911	0.399217	0.408665	-2124.11607	7.50
Cat 2S_conf49	-2124.510532	0.464248	0.497345	0.394567	0.407088	-2124.115965	7.57
Cat 2S_conf50	-2124.510847	0.464297	0.497358	0.395096	0.407129	-2124.115751	7.70
Cat 2S_conf51	-2124.515238	0.465272	0.497915	0.39966	0.408638	-2124.115578	7.81
Cat 2S_conf52	-2124.516304	0.466027	0.498291	0.400799	0.409932	-2124.115505	7.86
Cat 2S_conf53	-2124.516305	0.466033	0.498294	0.400826	0.409943	-2124.115479	7.87
Cat 2S_conf54	-2124.516305	0.466035	0.498295	0.400833	0.409946	-2124.115472	7.88
Cat 2S_conf55	-2124.511356	0.464604	0.497634	0.395979	0.407805	-2124.115377	7.94
Cat 2S_conf56	-2124.517611	0.465793	0.497286	0.40245	0.410287	-2124.115161	8.07
Cat 2S_conf57	-2124.517777	0.465993	0.498188	0.402657	0.409714	-2124.11512	8.10
Cat 2S_conf58	-2124.515632	0.465716	0.498225	0.400533	0.409189	-2124.115099	8.11
Cat 2S_conf59	-2124.517412	0.46653	0.498669	0.402606	0.410447	-2124.114806	8.30
Cat 2S_conf60	-2124.515091	0.465328	0.497897	0.400348	0.408559	-2124.114743	8.33
Cat 2S_conf61	-2124.515091	0.465327	0.497896	0.40035	0.408557	-2124.114741	8.34
Cat 2S_conf62	-2124.511536	0.464905	0.497828	0.396867	0.408156	-2124.114669	8.38
Cat 2S_conf63	-2124.509953	0.464631	0.49768	0.395302	0.407803	-2124.114651	8.39
Cat 2S_conf64	-2124.511409	0.464882	0.497811	0.396808	0.408106	-2124.114601	8.42
Cat 2S_conf65	-2124.515107	0.465363	0.497929	0.400618	0.408582	-2124.114489	8.49
Cat 2S_conf66	-2124.515107	0.465364	0.497929	0.400626	0.408584	-2124.114481	8.50
Cat 2S_conf67	-2124.510077	0.464716	0.497732	0.395701	0.407908	-2124.114376	8.56
Cat 2S_conf68	-2124.510077	0.464721	0.497732	0.395742	0.407909	-2124.114335	8.59
Cat 2S_conf69	-2124.507952	0.464216	0.497385	0.393811	0.407303	-2124.114141	8.71
Cat 2S_conf70	-2124.511602	0.464969	0.497825	0.397527	0.408134	-2124.114075	8.75
Cat 2S_conf71	-2124.517395	0.466805	0.498846	0.403346	0.410789	-2124.114049	8.77
Cat 2S_conf72	-2124.511476	0.464956	0.497811	0.397588	0.408089	-2124.113888	8.87
Cat 2S_conf73	-2124.511476	0.464958	0.497814	0.397597	0.408094	-2124.113879	8.88
Cat 2S_conf74	-2124.509861	0.464647	0.497633	0.396308	0.40793	-2124.113553	9.08
Cat 2S_conf75	-2124.507994	0.464269	0.497402	0.394465	0.407352	-2124.113529	9.10
Cat 2S_conf76	-2124.50809	0.464261	0.497387	0.394727	0.407328	-2124.113363	9.20
Cat 2S_conf77	-2124.50809	0.464263	0.497388	0.394737	0.40733	-2124.113353	9.21
Cat 2S_conf78	-2124.509755	0.46468	0.497637	0.396908	0.407924	-2124.112847	9.52
Cat 2S_conf79	-2124.509755	0.464683	0.497638	0.396916	0.407926	-2124.112839	9.53
Cat 2S_conf80	-2124.515105	0.465435	0.497815	0.402313	0.408956	-2124.112792	9.56

Cat 2S_conf81	-2124.509873	0.464747	0.497654	0.397306	0.407996	-2124.112567	9.70
Cat 2S_conf82	-2124.508584	0.464244	0.49731	0.396399	0.407288	-2124.112185	9.94
Cat 2S_conf83	-2124.509812	0.465177	0.497914	0.397811	0.408537	-2124.112001	10.06
Cat 2S_conf84	-2124.515835	0.466243	0.497616	0.40386	0.410886	-2124.111975	10.07
Cat 2S_conf85	-2124.508596	0.464902	0.497795	0.397316	0.408103	-2124.11128	10.51
Cat 2S_conf86	-2124.51234	0.465364	0.497779	0.401263	0.409148	-2124.111077	10.64
Cat 2S_conf87	-2124.505621	0.464438	0.497526	0.394665	0.407559	-2124.110956	10.71
Cat 2S_conf88	-2124.505581	0.464749	0.497742	0.394859	0.407873	-2124.110722	10.86
Cat 2S_conf89	-2124.508568	0.464964	0.497823	0.397943	0.408128	-2124.110625	10.92
Cat 2S_conf90	-2124.50801	0.464996	0.497883	0.397425	0.408238	-2124.110585	10.94
Cat 2S_conf91	-2124.50816	0.464952	0.497843	0.397656	0.40814	-2124.110504	10.99
Cat 2S_conf92	-2124.50816	0.464951	0.497841	0.397659	0.408138	-2124.110501	11.00
Cat 2S_conf93	-2124.507019	0.464441	0.496654	0.396536	0.408417	-2124.110483	11.01
Cat 2S_conf94	-2124.506871	0.464465	0.496682	0.396552	0.408423	-2124.110319	11.11
Cat 2S_conf95	-2124.505473	0.464793	0.49777	0.395387	0.40787	-2124.110086	11.26
Cat 2S_conf96	-2124.502031	0.464177	0.497452	0.393506	0.406991	-2124.108525	12.24
Cat 2S_conf97	-2124.502143	0.464328	0.497534	0.394264	0.407115	-2124.107879	12.64
Cat 2S_conf98	-2124.502143	0.464328	0.497534	0.394273	0.407116	-2124.10787	12.65
Cat 2S_conf99	-2124.507601	0.464559	0.495677	0.400053	0.409621	-2124.107548	12.85

Table S1.20 | Absolute Energies and Energy Corrections for Conformer Library of **1S** [B3LYP-D3/6-31G(d)-SMD(Toluene)].

Conformer	SCF Energy	zero point correction	Enthalpy correction	Free energy Correction	Quasiharmonic correction	Gibbs Free Energy (Hartree)	Relative Gibbs Free Energy (kcal/mol)
Cat 1S_conf1	-1822.042246	0.378871	0.405402	0.319642	0.327797	-1821.7226	0.00
Cat 1S_conf2	-1822.041915	0.378595	0.405219	0.320123	0.327407	-1821.72179	0.51
Cat 1S_conf3	-1822.043754	0.379221	0.405586	0.322268	0.328296	-1821.72149	0.70
Cat 1S_conf4	-1822.041883	0.379428	0.405762	0.320839	0.328539	-1821.72104	0.98
Cat 1S_conf5	-1822.043663	0.379208	0.405562	0.322681	0.328237	-1821.72098	1.02
Cat 1S_conf6	-1822.041337	0.379226	0.405561	0.320475	0.328427	-1821.72086	1.09
Cat 1S_conf7	-1822.04177	0.379451	0.405761	0.321209	0.328546	-1821.72056	1.28
Cat 1S_conf8	-1822.041749	0.379432	0.405745	0.321262	0.328523	-1821.72049	1.33
Cat 1S_conf9	-1822.039655	0.378712	0.405334	0.319626	0.32781	-1821.72003	1.62
Cat 1S_conf10	-1822.038608	0.378697	0.405371	0.319751	0.327594	-1821.71886	2.35
Cat 1S_conf11	-1822.04031	0.379156	0.405553	0.321599	0.328132	-1821.71871	2.44
Cat 1S_conf12	-1822.038608	0.378703	0.405372	0.319913	0.327596	-1821.7187	2.45
Cat 1S_conf13	-1822.039569	0.379103	0.405565	0.321255	0.328042	-1821.71831	2.69
Cat 1S_conf14	-1822.038745	0.378808	0.405427	0.320485	0.327715	-1821.71826	2.73
Cat 1S_conf15	-1822.03883	0.378845	0.405444	0.32083	0.327657	-1821.718	2.89
Cat 1S_conf16	-1822.037789	0.379123	0.405594	0.320005	0.328033	-1821.71778	3.02
Cat 1S_conf17	-1822.037849	0.379238	0.405663	0.320153	0.328218	-1821.7177	3.08
Cat 1S_conf18	-1822.037888	0.379214	0.405658	0.320465	0.328148	-1821.71742	3.25
Cat 1S_conf19	-1822.034075	0.3778	0.404759	0.316912	0.326296	-1821.71716	3.41
Cat 1S_conf20	-1822.038829	0.378986	0.405509	0.321857	0.327792	-1821.71697	3.53
Cat 1S_conf21	-1822.037269	0.378701	0.405342	0.320584	0.327644	-1821.71669	3.71
Cat 1S_conf22	-1822.037196	0.37878	0.405412	0.32058	0.327751	-1821.71662	3.76
Cat 1S_conf23	-1822.034851	0.377994	0.404845	0.318485	0.32644	-1821.71637	3.91
Cat 1S_conf24	-1822.034852	0.377995	0.404846	0.318493	0.326441	-1821.71636	3.92
Cat 1S_conf25	-1822.033931	0.378341	0.40512	0.318355	0.327522	-1821.71558	4.41
Cat 1S_conf26	-1822.033082	0.378007	0.404943	0.317588	0.326587	-1821.71549	4.46
Cat 1S_conf27	-1822.033822	0.378364	0.40514	0.318476	0.327525	-1821.71535	4.55
Cat 1S_conf28	-1822.033822	0.378369	0.405142	0.318507	0.327529	-1821.71532	4.57
Cat 1S_conf29	-1822.03356	0.378134	0.405015	0.318404	0.326657	-1821.71516	4.67
Cat 1S_conf30	-1822.032159	0.378509	0.405337	0.317143	0.327705	-1821.71502	4.76
Cat 1S_conf31	-1822.035208	0.379009	0.405534	0.320445	0.328137	-1821.71476	4.92
Cat 1S_conf32	-1822.035149	0.378992	0.405523	0.320495	0.328093	-1821.71465	4.99
Cat 1S_conf33	-1822.032527	0.378078	0.404908	0.318648	0.327237	-1821.71388	5.48
Cat 1S_conf34	-1822.031625	0.378176	0.404948	0.318252	0.326884	-1821.71337	5.79
Cat 1S_conf35	-1822.031625	0.378175	0.404947	0.318256	0.326882	-1821.71337	5.80
Cat 1S_conf36	-1822.034122	0.379641	0.405863	0.320811	0.329048	-1821.71331	5.83
Cat 1S_conf37	-1822.032982	0.378804	0.405414	0.319766	0.327928	-1821.71322	5.89

Cat 1S_conf38	-1822.033081	0.378866	0.40545	0.319957	0.327982	-1821.71312	5.95
Cat 1S_conf39	-1822.033411	0.379452	0.405785	0.320579	0.328714	-1821.71283	6.13
Cat 1S_conf40	-1822.033339	0.379414	0.40574	0.320918	0.328647	-1821.71242	6.39
Cat 1S_conf41	-1822.033061	0.37879	0.405332	0.320702	0.327964	-1821.71236	6.43
Cat 1S_conf42	-1822.030665	0.378162	0.405037	0.318398	0.326903	-1821.71227	6.49
Cat 1S_conf43	-1822.030941	0.378266	0.405046	0.318694	0.326911	-1821.71225	6.50
Cat 1S_conf44	-1822.030441	0.378363	0.405158	0.318399	0.326988	-1821.71204	6.63
Cat 1S_conf45	-1822.029489	0.377871	0.404841	0.317567	0.326483	-1821.71192	6.70
Cat 1S_conf46	-1822.029931	0.37828	0.405119	0.318197	0.32678	-1821.71173	6.82
Cat 1S_conf47	-1822.030391	0.378414	0.405177	0.318775	0.327019	-1821.71162	6.90
Cat 1S_conf48	-1822.02954	0.378142	0.404964	0.318864	0.326916	-1821.71068	7.48
Cat 1S_conf49	-1822.029415	0.378215	0.405027	0.318819	0.326979	-1821.7106	7.54
Cat 1S_conf50	-1822.027686	0.378274	0.405162	0.317225	0.327201	-1821.71046	7.62
Cat 1S_conf51	-1822.027686	0.37828	0.405163	0.317308	0.327208	-1821.71038	7.67
Cat 1S_conf52	-1822.032629	0.379726	0.406008	0.322379	0.329015	-1821.71025	7.75
Cat 1S_conf53	-1822.029852	0.378465	0.405194	0.319818	0.327507	-1821.71003	7.89
Cat 1S_conf54	-1822.029952	0.378499	0.405161	0.319955	0.32754	-1821.71	7.91
Cat 1S_conf55	-1822.029952	0.3785	0.405161	0.319962	0.327542	-1821.70999	7.92
Cat 1S_conf56	-1822.028068	0.378326	0.405131	0.31867	0.327071	-1821.7094	8.29
Cat 1S_conf57	-1822.027799	0.37878	0.405442	0.318797	0.327829	-1821.709	8.54
Cat 1S_conf58	-1822.028145	0.37856	0.405315	0.319166	0.327616	-1821.70898	8.55
Cat 1S_conf59	-1822.028062	0.37851	0.405248	0.319167	0.327302	-1821.7089	8.60
Cat 1S_conf60	-1822.025939	0.377889	0.40486	0.317053	0.326597	-1821.70889	8.61
Cat 1S_conf61	-1822.028046	0.378512	0.405272	0.319245	0.327565	-1821.7088	8.66
Cat 1S_conf62	-1822.026386	0.378122	0.40498	0.317811	0.326925	-1821.70858	8.80
Cat 1S_conf63	-1822.02648	0.378202	0.40504	0.31797	0.327033	-1821.70851	8.84
Cat 1S_conf64	-1822.027712	0.378514	0.405231	0.319857	0.327465	-1821.70786	9.26
Cat 1S_conf65	-1822.027995	0.378112	0.404086	0.32036	0.327584	-1821.70764	9.39
Cat 1S_conf66	-1822.024534	0.377857	0.404717	0.317291	0.326678	-1821.70724	9.64
Cat 1S_conf67	-1822.023775	0.377629	0.404609	0.316787	0.326369	-1821.70699	9.80
Cat 1S_conf68	-1822.025313	0.378687	0.405349	0.319101	0.327777	-1821.70621	10.29
Cat 1S_conf69	-1822.02397	0.378244	0.405043	0.318542	0.327138	-1821.70543	10.78
Cat 1S_conf70	-1822.02132	0.378274	0.405149	0.317529	0.327022	-1821.70379	11.81

1.5. CARTESIAN COORDINATES, ABSOLUTE ENERGIES AND CORRECTIONS OF OPTIMIZED STRUCTURES

SCF Energy* is given at B3LYP-D3/6-311+G(d,p)-SMD(Toluene).

**Name**	2-aminopyridine
**Absolute Energies (Hartree)**	There are 0 positive frequencies below 100 cm^-1.<br><br>Zero-point correction: 0.133784 hartree<br>Enthalpy correction: 0.141245 hartree<br>Free Energy correction: 0.106233 hartree<br>Quasiharmonic Free Energy correction: 0.106233 hartree<br><br>SCF Energy: -342.976150 hartree<br>SCF Energy*: -343.0733747 hartree<br>SCF Energy + ZPVE: -342.842366 hartree<br>Enthalpy: -342.834905 hartree<br>Free Energy: -342.869917 hartree<br><br>Free Energy with quasiharmonic correction: -342.869917 hartree (correction: 0.00 kcal/mol)<br>--<br>Low frequencies before projection:<br>-92.5225<br>150.0309<br>234.5663<br><br>Low frequencies after projection:<br>-92.5156<br>150.0152<br>234.5641<br>--
**Cartesian Coordinates**	N -0.1695718 1.6924981 0.0000000<br>C 0.2647762 0.3973981 0.0000000<br>C 1.2876822 -2.1621639 0.0000000<br>C -0.6307858 -0.6993009 0.0000000<br>N 1.6048262 0.2321361 0.0000000<br>C 2.0815042 -1.0164649 0.0000000<br>C -0.0996368 -1.9815419 0.0000000<br>C -1.5544418 2.1023301 0.0000000<br>H 0.5585192 2.3922171 0.0000000<br>H 1.7359412 -3.1503239 0.0000000<br>H -1.7037248 -0.5417799 0.0000000<br>H 3.1679242 -1.1020359 0.0000000<br>H -0.7673288 -2.8398009 0.0000000<br>H -2.0902918 1.7413511 0.8897500<br>H -1.5950998 3.1941301 0.0000000<br>H -2.0902918 1.7413511 -0.8897500

**Name**	trimethylamine
**Absolute Energies (Hartree)**	There are 0 positive frequencies below 100 cm^-1.<br><br>Zero-point correction: 0.121278 hartree<br>Enthalpy correction: 0.127552 hartree<br>Free Energy correction: 0.097086 hartree<br>Quasiharmonic Free Energy correction: 0.097086 hartree<br><br>SCF Energy: -174.484843 hartree<br>SCF Energy*: -174.5389023 hartree<br>SCF Energy + ZPVE: -174.363565 hartree<br>Enthalpy: -174.357291 hartree<br>Free Energy: -174.387757 hartree<br><br>Free Energy with quasiharmonic correction: -174.387757 hartree (correction: 0.00 kcal/mol)<br><br>---<br>Low frequencies before projection:<br>260.7105<br>284.2561<br>285.0669<br>Low frequencies after projection:<br>260.7083<br>284.2556<br>285.0648<br>---
**Cartesian Coordinates**	N -0.0000063 -0.0000795 -0.4802938<br>C 1.2926147 -0.5062175 -0.0413238<br>C -1.0847283 -0.8663145 -0.0413238<br>C -0.2079233 1.3725015 -0.0414238<br>H 1.4475057 -1.5191925 -0.4306138<br>H -0.9325413 -1.8797255 -0.4305138<br>H -2.0393773 -0.4937555 -0.4305438<br>H -1.1615613 1.7475125 -0.4307538<br>H 0.5921457 2.0129655 -0.4304738<br>H -0.2244283 1.4804045 1.0624962<br>H -1.1700523 -0.9344405 1.0625762<br>H 1.3942437 -0.5461115 1.0625862<br>H 2.0941087 0.1324535 -0.4303938

Name	urea
**Absolute Energies (Hartree)**	There are 1 positive frequencies below 100 cm^-1.<br><br>Zero-point correction: 0.118619 hartree<br>Enthalpy correction: 0.126444 hartree<br>Free Energy correction: 0.091142 hartree<br>Quasiharmonic Free Energy correction: 0.091317 hartree<br><br>SCF Energy: -303.894816 hartree<br>SCF Energy*: -303.9974178 hartree<br>SCF Energy + ZPVE: -303.776197 hartree<br>Enthalpy: -303.768372 hartree<br>Free Energy: -303.803674 hartree<br><br>Free Energy with quasiharmonic correction: -303.803499 hartree (correction: 0.11 kcal/mol)<br><br>---<br>Low frequencies before projection:<br>-0.0008<br>-0.0007<br>96.9967<br>Low frequencies after projection:<br>-245.6919<br>-78.384<br>82.8304<br>---
**Cartesian Coordinates**	N -0.0000000 1.1554300 -0.4679207<br>C -0.0000000 -0.0000000 0.2808093<br>O -0.0000000 -0.0000000 1.5136593<br>N -0.0000000 -1.1554300 -0.4679207<br>C -0.0008000 2.4725600 0.1345693<br>C 0.0008000 -2.4725600 0.1345693<br>H -0.0015700 1.0948400 -1.4761007<br>H 0.0015700 -1.0948400 -1.4761007<br>H -0.8947000 3.0432300 -0.1492307<br>H 0.8882300 3.0470400 -0.1565607<br>H 0.0038200 2.3439900 1.2180093<br>H -0.8882300 -3.0470400 -0.1565607<br>H 0.8947000 -3.0432300 -0.1492307<br>H -0.0038200 -2.3439900 1.2180093

**Name**	thiourea
**Absolute Energies (Hartree)**	There are 2 positive frequencies below 100 cm^-1.<br><br>Zero-point correction: 0.118103 hartree<br>Enthalpy correction: 0.127091 hartree<br>Free Energy correction: 0.089209 hartree<br>Quasiharmonic Free Energy correction: 0.089380 hartree<br><br>SCF Energy: -626.855723 hartree<br>SCF Energy*: -626.9559716 hartree<br>SCF Energy + ZPVE: -626.737620 hartree<br>Enthalpy: -626.728632 hartree<br>Free Energy: -626.766514 hartree<br><br>Free Energy with quasiharmonic correction: -626.766343 hartree (correction: 0.11 kcal/mol)<br><br>--<br>Low frequencies before projection:<br>89.7716<br>94.9840<br>162.6444<br>Low frequencies after projection:<br>87.5981<br>94.924<br>162.6444<br>--
**Cartesian Coordinates**	N 0.0000000 1.1440200 -0.4648864<br>C 0.0000000 0.0000000 0.2664736<br>N 0.0000000 -1.1440200 -0.4648864<br>C -0.0526800 2.4810900 0.1010936<br>C 0.0526800 -2.4810900 0.1010936<br>H -0.1082600 1.0683200 -1.4689564<br>H 0.1082600 -1.0683200 -1.4689564<br>H -0.9801300 2.6471800 0.6612236<br>H 0.0043700 3.1965500 -0.7235664<br>H 0.7889700 2.6477700 0.7796736<br>H -0.0043700 -3.1965500 -0.7235664<br>H 0.9801300 -2.6471800 0.6612236<br>H -0.7889700 -2.6477700 0.7796736<br>S 0.0000000 0.0000000 1.9643636

Name	dimethylmalonate
**Absolute Energies (Hartree)**	There are 3 positive frequencies below 100 cm^-1.<br><br>Zero-point correction: 0.134322 hartree<br>Enthalpy correction: 0.145369 hartree<br>Free Energy correction: 0.100420 hartree<br>Quasiharmonic Free Energy correction: 0.102191 hartree<br><br>SCF Energy: -496.276526 hartree<br>SCF Energy*: -496.4328158 hartree<br>SCF Energy + ZPVE: -496.142204 hartree<br>Enthalpy: -496.131157 hartree<br>Free Energy: -496.176106 hartree<br><br>Free Energy with quasiharmonic correction: -496.174335 hartree (correction: 1.11 kcal/mol)<br><br>--<br>Low frequencies before projection:<br>27.9995<br>62.7470<br>91.8433<br>Low frequencies after projection:<br>26.1732<br>62.7327<br>91.8408<br>--
**Cartesian Coordinates**	C -0.0267053 1.1154721 -0.4317659<br>C -1.4049753 0.7202021 0.0587141<br>O -2.0657053 1.3475621 0.8564741<br>O -1.7982253 -0.4299179 -0.5161759<br>C 1.0440547 0.2355921 0.1942641<br>O 0.8793747 -0.4982079 1.1443541<br>O 2.2175647 0.4061321 -0.4388359<br>C 3.3270347 -0.3423969 0.0993341<br>C -3.0667653 -0.9436779 -0.0663859<br>H 0.1653247 2.1542521 -0.1473859<br>H 0.0343447 1.0404521 -1.5215459<br>H 3.1280147 -1.4164369 0.0443741<br>H 3.5110547 -0.0623269 1.1405641<br>H 4.1841447 -0.0813669 -0.5227959<br>H -3.0363153 -1.1474379 1.0077941<br>H -3.8688853 -0.2301579 -0.2764659<br>H -3.2233353 -1.8677379 -0.6245159

**Name**	*trans-β*-nitrostyrene
**Absolute Energies (Hartree)**	There are 2 positive frequencies below 100 cm^-1.<br><br>Zero-point correction: 0.137302 hartree<br>Enthalpy correction: 0.147292 hartree<br>Free Energy correction: 0.104773 hartree<br>Quasiharmonic Free Energy correction: 0.105644 hartree<br><br>SCF Energy: -514.176975 hartree<br>SCF Energy*: -514.3235866 hartree<br>SCF Energy + ZPVE: -514.039673 hartree<br>Enthalpy: -514.029683 hartree<br>Free Energy: -514.072202 hartree<br><br>Free Energy with quasiharmonic correction: -514.071331 hartree (correction: 0.55 kcal/mol)<br><br>---<br>Low frequencies before projection:<br>43.2186<br>91.3679<br>111.4276<br>Low frequencies after projection:<br>43.1245<br>91.359<br>111.4276<br>---
**Cartesian Coordinates**	C -2.2780362 -0.4213273 0.0000067<br>C -1.2869172 0.4844127 0.0000567<br>N -3.6621372 -0.0047073 0.0000967<br>C 0.1447128 0.2075537 0.0000167<br>C 2.9253138 -0.2041763 0.0000067<br>C 1.0352528 1.2982037 0.0001967<br>C 0.6773638 -1.0980963 -0.0001733<br>C 2.0525938 -1.2987063 -0.0001833<br>C 2.4133328 1.0944737 0.0001967<br>O -4.5017462 -0.9138083 -0.0000933<br>O -3.9423972 1.1989527 -0.0002233<br>H -2.1803762 -1.4976073 -0.0000233<br>H -1.5814672 1.5313027 0.0001167<br>H 3.9996038 -0.3667053 0.0000167<br>H 0.6368528 2.3096837 0.0003267<br>H 0.0128738 -1.9568063 -0.0003533<br>H 2.4491538 -2.3101263 -0.0003333<br>H 3.0860228 1.9474837 0.0003467

Name	**1S** catalyst
**Absolute Energies (Hartree)**	There are 9 positive frequencies below 100 cm^-1.<br><br>Zero-point correction: 0.378460 hartree<br>Enthalpy correction: 0.405175 hartree<br>Free Energy correction: 0.322804 hartree<br>Quasiharmonic Free Energy correction: 0.330111 hartree<br><br>SCF Energy: -1822.058907 hartree<br>SCF Energy*: -1822.509175 hartree<br>SCF Energy + ZPVE: -1821.680447 hartree<br>Enthalpy: -1821.653732 hartree<br>Free Energy: -1821.736103 hartree<br><br>Free Energy with quasiharmonic correction: -1821.728796 hartree (correction: 4.59 kcal/mol)<br><br>---<br>Low frequencies before projection:<br>13.2792<br>15.2039<br>16.9870<br>Low frequencies after projection:<br>13.0811<br>15.0304<br>16.8849<br>---
**Cartesian Coordinates**	N 0.5941729 -1.3231853 0.0918040<br>N -1.3679771 -2.3297053 -0.6237160<br>N 1.2312229 1.3524447 -0.4116660<br>C 2.0136329 -0.9735653 0.0770840<br>C 3.6644429 0.9076747 0.2714540<br>C 4.3159929 -1.4283453 1.0158440<br>C 4.5165829 0.0760547 1.2431640<br>C 2.8304029 -1.8084653 1.0782740<br>C 2.1693129 0.5333547 0.3767140<br>C 0.0143029 -2.2958853 -0.6275660<br>C -2.3131671 -1.3174253 -0.3851360<br>C -4.3563571 0.5906747 -0.0031260<br>C -2.0274271 0.0533447 -0.4767660<br>C -3.6309871 -1.7221953 -0.1074260<br>C -4.6332471 -0.7765543 0.0713640<br>C -3.0502071 0.9831947 -0.2759860<br>C -2.6953171 2.4445047 -0.3439460<br>C -6.0293071 -1.2226843 0.4175140<br>F -1.9056671 2.7185147 -1.4124560<br>F -2.0060371 2.8374847 0.7553140<br>F -3.7824071 3.2372947 -0.4349260<br>F -6.2818471 -2.4805243 -0.0058960

	F	-6.2365271	-1.2069643	1.7558740
	F	-6.9617571	-0.4127943	-0.1312860
	H	2.3843029	-1.1922653	-0.9294260
	H	4.0102129	0.7364947	-0.7568560
	H	3.7935829	1.9762147	0.4774540
	H	4.7141829	-1.7039453	0.0288240
	H	4.8814629	-2.0076753	1.7557740
	H	4.2327429	0.3276447	2.2756640
	H	5.5754229	0.3420937	1.1351740
	H	2.6887829	-2.8690953	0.8496040
	H	2.4374229	-1.6337753	2.0900940
	H	1.8669629	0.6822947	1.4251640
	H	-5.1417071	1.3233157	0.1354140
	H	-1.0288571	0.4114247	-0.7094760
	H	-3.8660871	-2.7798053	-0.0408160
	H	-1.7375771	-3.1930053	-1.0027660
	H	0.0065029	-0.7175453	0.6508040
	S	0.8568629	-3.4764553	-1.5045060
	C	1.4571929	1.3462547	-1.8551660
	C	1.1119029	2.7166047	0.0963340
	H	1.4987729	0.3212147	-2.2342160
	H	0.6132229	1.8460147	-2.3430260
	H	2.3817529	1.8661547	-2.1622160
	H	2.0290729	3.3190747	-0.0308360
	H	0.3009029	3.2269647	-0.4281160
	H	0.8611329	2.6955547	1.1626140

Name	**1O** catalyst
**Absolute Energies (Hartree)**	There are 8 positive frequencies below 100 cm^-1.<br><br>Zero-point correction: 0.380018 hartree<br>Enthalpy correction: 0.406742 hartree<br>Free Energy correction: 0.323240 hartree<br>Quasiharmonic Free Energy correction: 0.331838 hartree<br><br>SCF Energy: -1499.092935 hartree<br>SCF Energy*: -1499.547319 hartree<br>SCF Energy + ZPVE: -1498.712917 hartree<br>Enthalpy: -1498.686193 hartree<br>Free Energy: -1498.769695 hartree<br><br>Free Energy with quasiharmonic correction: -1498.761097 hartree (correction: 5.40 kcal/mol)<br><br>---<br>Low frequencies before projection:<br>12.7496<br>15.1273<br>18.0701<br>Low frequencies after projection:<br>12.2258<br>14.9288<br>17.8319<br>---
**Cartesian Coordinates**	N 1.0162674 -0.5765719 -0.7051856<br>N -1.2753326 -0.6726319 -0.4742156<br>N 2.5585374 -0.2407519 1.8582744<br>C 2.3391874 0.0404281 -0.6869256<br>C 4.5737674 0.2034171 0.4921244<br>C 4.4765674 0.3847471 -2.0275756<br>C 5.3035674 -0.0882829 -0.8258056<br>C 3.0772474 -0.2419719 -2.0081956<br>C 3.1704574 -0.4359019 0.5387344<br>C -0.1427626 0.1371681 -0.5854056<br>C -2.6104226 -0.2784809 -0.3479856<br>C -5.3518526 0.3463991 -0.0801356<br>C -3.0300626 1.0609291 -0.2941756<br>C -3.5766726 -1.2953409 -0.2658856<br>C -4.9252926 -0.9801009 -0.1341956<br>C -4.3878826 1.3492891 -0.1621056<br>C -4.8085216 2.7897791 -0.0341156<br>C -5.9430026 -2.0879909 -0.1215356<br>O -0.1869526 1.3655481 -0.5588456<br>F -4.7533216 3.2104691 1.2530444<br>F -6.0797416 2.9847191 -0.4539356<br>F -4.0150316 3.6166591 -0.7494556

	F	-7.0404026	-1.7581609	0.5942944
	F	-6.3675326	-2.3897609	-1.3727556
	F	-5.4438526	-3.2308209	0.4049544
	H	2.1650274	1.1161681	-0.6009956
	H	4.4844174	1.2914871	0.6132444
	H	5.1461274	-0.1648929	1.3507344
	H	4.3819174	1.4797581	-1.9949456
	H	4.9845174	0.1398571	-2.9685856
	H	5.4840274	-1.1701329	-0.9123756
	H	6.2883974	0.3951671	-0.8236956
	H	2.4674274	0.1303281	-2.8400956
	H	3.1631274	-1.3319319	-2.1394256
	H	3.3098674	-1.5219719	0.4275244
	H	-6.4023126	0.5888991	0.0233044
	H	-2.2994016	1.8534991	-0.3586256
	H	-3.2711626	-2.3372209	-0.2989356
	H	-1.1304426	-1.6735019	-0.5118556
	H	0.9858874	-1.5875319	-0.6862856
	C	2.0664974	1.1011581	2.1474844
	C	1.6235474	-1.2659819	2.2936244
	H	1.4911574	-1.1955119	3.3813744
	H	0.6184174	-1.1993019	1.8387144
	H	2.0316274	-2.2600619	2.0757844
	H	1.1363374	1.3633281	1.6157544
	H	1.8754974	1.1863181	3.2244044
	H	2.8225384	1.8492881	1.8908844

Name	**2O** catalyst
**Absolute Energies (Hartree)**	There are 12 positive frequencies below 100 cm^-1.<br><br>Zero-point correction: 0.467859 hartree<br>Enthalpy correction: 0.499950 hartree<br>Free Energy correction: 0.407378 hartree<br>Quasiharmonic Free Energy correction: 0.414682 hartree<br><br>SCF Energy: -1801.592017 hartree<br>SCF Energy*: -1802.120998 hartree<br>SCF Energy + ZPVE: -1801.124158 hartree<br>Enthalpy: -1801.092067 hartree<br>Free Energy: -1801.184639 hartree<br><br>Free Energy with quasiharmonic correction: -1801.177335 hartree (correction: 4.58 kcal/mol)<br><br>---<br>Low frequencies before projection:<br>18.1893<br>24.0357<br>30.0747<br>Low frequencies after projection:<br>18.1271<br>24.0342<br>30.0178<br>---
**Cartesian Coordinates**	N 2.0826120 0.3028981 -1.7053681<br>N -0.2290280 0.2586481 -1.7854081<br>N 2.3409920 -0.0712819 1.1550719<br>N 1.4034310 -2.0057419 0.2312319<br>N -2.3464990 -1.0015409 1.8933719<br>C 3.3127120 0.6992171 -1.0181581<br>C 4.7670920 0.3578671 1.0219419<br>C 5.8103320 1.0202771 -1.2001381<br>C 6.0067120 0.2588171 0.1195319<br>C 4.5380120 0.5648371 -1.9307181<br>C 3.5096110 -0.1235129 0.2849619<br>C 0.9128720 0.9764281 -1.4267081<br>C -1.5476180 0.5346791 -1.4165381<br>C -4.2396280 0.9001391 -0.6413581<br>C -1.9586680 1.7407691 -0.8276681<br>C -2.5024690 -0.4736609 -1.6314981<br>C -3.8246590 -0.2869109 -1.2448081<br>C -3.2907280 1.9006491 -0.4467681<br>C -3.6815480 3.1563291 0.2826719<br>C -4.8269790 -1.3774009 -1.4988381<br>C 1.2423610 -0.8986619 0.9845019<br>C -0.9227290 -2.5524719 0.6507819

	C	0.0211610	-0.5489919	1.5888419
	C	0.3254310	-2.7993819	0.1094619
	C	-1.1127290	-1.3594719	1.4049819
	C	-2.4668880	0.1633791	2.7579919
	C	-3.4390690	-1.9645509	1.8967619
	O	0.8740320	2.1018381	-0.9348581
	F	-3.4474480	3.0458191	1.6185619
	F	-4.9964580	3.4391291	0.1454219
	F	-2.9872070	4.2333291	-0.1419981
	F	-5.8180890	-1.3754799	-0.5777681
	F	-5.4167790	-1.2605799	-2.7109481
	F	-4.2543690	-2.6085509	-1.4641181
	H	3.1824520	1.7516971	-0.7477681
	H	4.6220620	1.4025671	1.3372319
	H	4.8984510	-0.2357129	1.9338919
	H	5.7343420	2.0968071	-0.9888881
	H	6.6843020	0.8881671	-1.8496581
	H	6.2037010	-0.8004929	-0.1002681
	H	6.8882120	0.6389671	0.6503119
	H	4.3771620	1.1524171	-2.8419981
	H	4.6437410	-0.4857929	-2.2403981
	H	3.6431610	-1.1739529	0.0087619
	H	-5.2677780	1.0369091	-0.3299781
	H	-1.2373880	2.5266091	-0.6606981
	H	-2.2003490	-1.4204209	-2.0671581
	H	-1.7253890	-3.2572109	0.4796519
	H	-0.0300880	0.3683281	2.1618219
	H	0.4777610	-3.7036019	-0.4800481
	H	-2.0595180	1.0528491	2.2704219
	H	-3.5230680	0.3582091	2.9518119
	H	-1.9543280	0.0243391	3.7224619
	H	-3.5852790	-2.3960809	0.9047319
	H	-3.2738290	-2.7850609	2.6118619
	H	-4.3654490	-1.4525509	2.1641119
	H	2.1108520	0.8590481	1.4844219
	H	-0.0780690	-0.6893019	-2.1068681
	H	1.9965610	-0.7035919	-1.8101881

Name	**2S** catalyst
**Absolute Energies (Hartree)**	There are 11 positive frequencies below 100 cm^-1.<br><br>Zero-point correction: 0.465190 hartree<br>Enthalpy correction: 0.497495 hartree<br>Free Energy correction: 0.405041 hartree<br>Quasiharmonic Free Energy correction: 0.411704 hartree<br><br>SCF Energy: -2124.546631 hartree<br>SCF Energy*: -2125.073501 hartree<br>SCF Energy + ZPVE: -2124.081441 hartree<br>Enthalpy: -2124.049136 hartree<br>Free Energy: -2124.141590 hartree<br><br>Free Energy with quasiharmonic correction: -2124.134927 hartree (correction: 4.18 kcal/mol)<br><br>--<br>Low frequencies before projection:<br>19.6016<br>31.6083<br>34.3484<br>Low frequencies after projection:<br>19.3896<br>31.577<br>34.342<br>--
**Cartesian Coordinates**	N 2.1352727 1.5847790 -0.2377369<br>N 0.6907727 3.3294190 0.0795531<br>N 1.4575317 -1.1492910 0.2739231<br>N 0.2443727 -0.1758610 -1.4447869<br>N -3.2108083 -2.2943700 -0.3204269<br>C 3.2149127 0.6465580 0.0645131<br>C 3.8312417 -1.7979620 0.0935931<br>C 5.6188727 -0.0370820 -0.2968769<br>C 5.1777217 -1.4809320 -0.5742869<br>C 4.5236627 0.9683580 -0.6785469<br>C 2.7509127 -0.7841420 -0.3100869<br>C 1.9721927 2.8059990 0.2836831<br>C -0.4902873 2.6218490 0.4217331<br>C -2.8671973 1.2857400 1.1075931<br>C -0.4797373 1.6098690 1.3894231<br>C -1.6967573 2.9639090 -0.2012969<br>C -2.8702573 2.2976900 0.1477231<br>C -1.6609773 0.9497190 1.7168731<br>C -1.6347373 -0.0922010 2.8005031<br>C -4.1508473 2.5732000 -0.5884769

	C	0.2765317	-1.0772510	-0.4419369
	C	-2.0880073	-0.7021410	-1.7838469
	C	-0.8342683	-1.8530710	-0.0747869
	C	-0.9245273	-0.0290310	-2.0910269
	C	-2.0704183	-1.6413410	-0.7144369
	C	-3.1185883	-3.4137800	0.6066931
	C	-4.4596883	-2.0722100	-1.0368869
	F	-0.4786273	-0.7976210	2.8049031
	F	-2.6494573	-0.9811410	2.6749531
	F	-1.7535273	0.4600390	4.0310131
	F	-4.1206773	3.7389000	-1.2667469
	F	-4.4195573	1.5954300	-1.4977869
	F	-5.2133873	2.6134300	0.2473331
	H	3.4009027	0.6919280	1.1462831
	H	3.9465117	-1.7807020	1.1878531
	H	3.4946817	-2.8055620	-0.1805269
	H	5.8511327	0.0753480	0.7720031
	H	6.5421427	0.1892180	-0.8437969
	H	5.0787117	-1.6269820	-1.6593169
	H	5.9395617	-2.1903720	-0.2284269
	H	4.8308227	1.9888780	-0.4372869
	H	4.3316927	0.9288480	-1.7602469
	H	2.6370427	-0.8123220	-1.4012169
	H	-3.7796473	0.7582300	1.3587831
	H	0.4462427	1.3280290	1.8745431
	H	-1.7061473	3.7315290	-0.9674769
	H	-2.9868773	-0.4875000	-2.3438669
	H	-0.7344983	-2.5573310	0.7409031
	H	-0.9225373	0.7007890	-2.8986469
	H	-2.4851083	-4.2217810	0.2121831
	H	-2.7128683	-3.0938200	1.5714731
	H	-4.1166183	-3.8186500	0.7827231
	H	-5.2716983	-2.5667800	-0.5009769
	H	-4.6955773	-1.0037500	-1.0843869
	H	-4.4333883	-2.4670500	-2.0637969
	H	1.4830717	-1.8486710	1.0038531
	H	0.6434437	4.3302490	0.2363031
	H	1.3814527	1.1943890	-0.8252369
	S	3.1358927	3.6983780	1.1242731

Name	TS_{Theo1S} / $TS_{Theo1S-BMA}$
**Absolute Energies (Hartree)**	There are 13 positive frequencies below 100 cm^-1.<br><br>Zero-point correction: 0.520018 hartree<br>Enthalpy correction: 0.555948 hartree<br>Free Energy correction: 0.454647 hartree<br>Quasiharmonic Free Energy correction: 0.461640 hartree<br><br>SCF Energy: -1811.847824 hartree<br>SCF Energy*: -1812.290689 hartree<br>SCF Energy + ZPVE: -1811.327806 hartree<br>Enthalpy: -1811.291876 hartree<br>Free Energy: -1811.393176 hartree<br><br>Free Energy with quasiharmonic correction: -1811.386183 hartree (correction: 4.39 kcal/mol)<br><br>---<br>Low frequencies before projection:<br>-199.0560<br>24.0863<br>28.4525<br><br>Low frequencies after projection:<br>-199.0547<br>23.5872<br>28.2981<br>---
**Cartesian Coordinates**	N -0.7336137 2.6048677 0.1673510<br>N -4.2204337 -0.1479323 -1.3758890<br>C -4.7932137 -0.9881723 -0.4798090<br>N -3.9929747 -2.0024723 -0.0754390<br>N -0.6264337 -0.9474123 -1.2727590<br>O -1.3743837 -0.1792223 -1.9619990<br>O -1.1136347 -1.9118823 -0.6194590<br>C 0.7252863 -0.7239323 -1.3018590<br>C 1.6049353 -1.4902223 -0.5370190<br>C 1.7233263 -0.5605423 1.5288410<br>C 0.3164963 -0.5580123 1.8321310<br>O -0.5066737 0.3113077 1.5192510<br>O -0.0681347 -1.6795423 2.4821010<br>C -1.4851047 -1.8193423 2.6739410<br>C 2.3925863 0.6636777 1.1608310<br>O 1.8880763 1.6556077 0.6268710<br>O 3.7275063 0.5890867 1.3981910<br>C 4.5032063 1.7120867 0.9648110<br>C -4.3786147 -3.0143823 0.8877010<br>C -4.8886437 0.9898777 -1.9731490<br>C -2.1989137 2.3206577 0.2113510

	H	2.3251663	-1.3173523	2.0153910
	H	-5.2840047	-3.5396623	0.5671410
	H	-3.5567647	-3.7317423	0.9637710
	H	-4.5729147	-2.5846223	1.8784610
	H	-3.0226947	-2.0061123	-0.3952890
	H	-3.2560837	-0.3270723	-1.6627590
	H	-5.7715337	0.6850477	-2.5465090
	H	-4.1777037	1.4767077	-2.6474590
	H	-5.2176237	1.7095677	-1.2144290
	H	-1.6285747	-2.8260123	3.0704610
	H	1.0135263	0.1094077	-1.9212690
	H	-2.7442037	3.1929677	-0.1564090
	H	-2.4796037	2.0975577	1.2402810
	H	-1.8567037	-1.0784423	3.3896310
	H	-2.0096047	-1.6955123	1.7252210
	H	-0.2583137	1.7420677	0.5284710
	H	-2.3968237	1.4490377	-0.4103590
	H	4.2415863	2.6105267	1.5342610
	H	5.5433363	1.4393767	1.1516310
	H	4.3536663	1.9087167	-0.1002190
	H	1.1801753	-2.3764923	-0.0784690
	C	3.0492953	-1.5463433	-0.8234990
	C	5.8187953	-1.7267433	-1.2946790
	C	3.7114663	-0.5748533	-1.5945590
	C	3.8033153	-2.6077233	-0.2936190
	C	5.1728253	-2.6993333	-0.5271290
	C	5.0819863	-0.6660233	-1.8274790
	H	3.1591463	0.2634967	-2.0064390
	H	3.3044953	-3.3619433	0.3102210
	H	5.7368153	-3.5292233	-0.1101890
	H	5.5766463	0.0939267	-2.4266190
	H	6.8876253	-1.7958733	-1.4779090
	C	-0.2610137	2.8580977	-1.2239190
	C	-0.3490837	3.7037777	1.0965110
	H	-0.6574337	3.4265677	2.1061510
	H	-0.8490737	4.6257977	0.7899510
	H	0.7339663	3.8166977	1.0639010
	H	-0.7228037	3.7774377	-1.5930490
	H	0.8240363	2.9521477	-1.2026190
	H	-0.5569437	2.0086677	-1.8414190
	S	-6.3890137	-0.7806113	0.0888510

<table>
<tr><td>Name</td><td>TS_{Theo1S-BMB}</td></tr>
<tr><td>Absolute Energies (Hartree)</td><td>There are 13 positive frequencies below 100 cm^-1.

Zero-point correction: 0.519067 hartree
Enthalpy correction: 0.555379 hartree
Free Energy correction: 0.452169 hartree
Quasiharmonic Free Energy correction: 0.460520 hartree

SCF Energy: -1811.842602 hartree
SCF Energy*: -1812.286383 hartree
SCF Energy + ZPVE: -1811.323535 hartree
Enthalpy: -1811.287223 hartree
Free Energy: -1811.390433 hartree

Free Energy with quasiharmonic correction: -1811.382083 hartree (correction: 5.24 kcal/mol)

Low frequencies before projection:
-140.0585
15.3547
23.5914

Low frequencies after projection:
-140.058
11.1581
23.4145
---</td></tr>
<tr><td>Cartesian Coordinates</td><td>N -1.2896740 2.1894570 -0.7687481
C -2.3499740 2.3009870 0.0555819
S -3.9104940 2.7641370 -0.4897681
N -2.0886040 1.9912370 1.3470619
C -3.0585440 2.0464170 2.4199619
C -3.5005040 -1.0759630 0.5189219
N -3.4841640 -1.6095930 -0.8784081
O -1.1796140 -0.7132330 -1.9882481
N -0.2317040 -1.2164530 -1.2930181
O -0.4099640 -2.2769130 -0.6460581
C 0.9805560 -0.5698030 -1.3256281
C 2.0646160 -1.0400830 -0.6016281
C 3.4387060 -0.5808220 -0.8325581
C 3.7399260 0.6118880 -1.5150181
C 5.0616660 1.0061380 -1.7058381
C 6.1090860 0.2197880 -1.2190081
C 5.8243660 -0.9665920 -0.5374681
C 4.5032060 -1.3600020 -0.3446081
C 1.8668960 -0.1881630 1.6218919
C 0.6057960 -0.8047230 1.9183319
O -0.5245840 -0.3382730 1.7517519</td></tr>
</table>

	C	1.9724260	1.2001170	1.2750319
	O	1.0947660	1.9384270	0.8167419
	H	-3.4868140	3.0492370	2.5099419
	H	-2.5388940	-1.2847430	0.9893019
	H	-0.3960340	1.9475170	-0.3387781
	H	-1.2373540	1.4565370	1.5278119
	H	2.7551560	-0.6699920	2.0079519
	H	0.9707160	0.3237870	-1.9284881
	H	1.9510760	-2.0161530	-0.1436681
	H	4.2833560	-2.2782620	0.1945519
	H	6.6327260	-1.5835520	-0.1545581
	H	7.1397060	0.5300680	-1.3683281
	H	5.2750560	1.9321080	-2.2330681
	H	2.9401160	1.2449680	-1.8851081
	C	-0.4080840	-2.8244930	2.6143519
	H	-0.9154240	-3.0050330	1.6614819
	H	-1.0916440	-2.3111930	3.2988219
	C	3.4851660	3.0043980	1.0363919
	H	2.9311960	3.7073880	1.6676819
	H	3.1945160	3.1641780	-0.0058081
	O	0.7858960	-2.0686130	2.4054119
	O	3.2474460	1.6531980	1.4435719
	H	-0.0857140	-3.7723230	3.0517019
	H	4.5593560	3.1612880	1.1527419
	H	-2.5740340	-1.2908830	-1.3168881
	C	-3.4703140	-3.0986330	-0.9048781
	H	-3.3729040	-3.4309330	-1.9403981
	H	-4.4028740	-3.4726930	-0.4756681
	H	-2.6101640	-3.4436630	-0.3327481
	H	-4.5003840	-1.4182130	-2.7216781
	C	-4.5894240	-1.0385530	-1.7016781
	H	-4.4996140	0.0505070	-1.6940581
	H	-5.5481440	-1.3380330	-1.2711781
	C	-1.3194640	2.4501370	-2.1919681
	H	-1.9313340	1.7155770	-2.7246181
	H	-0.2933240	2.3822770	-2.5642881
	H	-1.7094340	3.4511570	-2.4046281
	H	-2.5379640	1.7949870	3.3481419
	H	-4.3225840	-1.5450530	1.0654019
	H	-3.8902340	1.3426170	2.2798719
	H	-3.6535140	0.0011070	0.4680719

Name	TS_{Theo1O} / $TS_{Theo1O-BMA}$
Absolute Energies (Hartree)	There are 14 positive frequencies below 100 cm^-1.<br><br>Zero-point correction: 0.522141 hartree<br>Enthalpy correction: 0.557920 hartree<br>Free Energy correction: 0.456328 hartree<br>Quasiharmonic Free Energy correction: 0.463821 hartree<br><br>SCF Energy: -1488.884752 hartree<br>SCF Energy*: -1489.329038 hartree<br>SCF Energy + ZPVE: -1488.362611 hartree<br>Enthalpy: -1488.326832 hartree<br>Free Energy: -1488.428424 hartree<br><br>Free Energy with quasiharmonic correction: -1488.420931 hartree (correction: 4.70 kcal/mol)<br><br>---<br>Low frequencies before projection:<br>-215.0816<br>17.8071<br>27.1520<br><br>Low frequencies after projection:<br>-215.0815<br>17.4356<br>27.0581<br>---
Cartesian Coordinates	N -0.8516139 2.3919046 -0.8721263<br>N -4.2223029 -1.9684964 -1.0046263<br>C -4.7651229 -1.1589664 -0.0338063<br>O -5.9804629 -1.0143964 0.1298337<br>N -3.8194029 -0.5071264 0.7347237<br>N -0.6526429 -1.3385154 -0.6378263<br>O -1.4360929 -0.9839354 -1.5798263<br>O -1.1146029 -1.8502954 0.4194537<br>C 0.6953271 -1.1832654 -0.8288063<br>C 1.6166471 -1.5166654 0.1679837<br>C 1.8412071 0.2154346 1.5536837<br>C 0.4644371 0.3243946 1.9719137<br>O -0.4398729 0.9559146 1.4126637<br>O 0.2351771 -0.4194154 3.0794237<br>C -1.1337629 -0.5450654 3.4863637<br>C 2.4103571 1.1728246 0.6371837<br>O 1.8121261 1.8133646 -0.2311563<br>O 3.7596061 1.2463556 0.7687937<br>C 4.4359861 2.0782356 -0.1804563<br>C -4.2236929 0.2098136 1.9256737<br>C -5.0713229 -2.5179664 -2.0412763

	C	-2.3013239	2.1075236	-0.6526363
	H	2.5224371	-0.2318254	2.2663537
	H	-5.1459629	0.7580836	1.7196937
	H	-4.4114929	-0.4490964	2.7882037
	H	-3.4355129	0.9178336	2.1976237
	H	-2.8726329	-0.8808264	0.7113337
	H	-3.2417729	-1.8147364	-1.2322363
	H	-5.9819229	-2.9224564	-1.5925763
	H	-4.5364829	-3.3275364	-2.5478963
	H	-5.3713129	-1.7728764	-2.7953163
	H	-1.1141029	-1.1479154	4.3964237
	H	0.9520471	-0.7648554	-1.7883263
	H	-2.8925239	2.7444336	-1.3151263
	H	-2.5390139	2.3155436	0.3896637
	H	-1.5737729	0.4339136	3.6957737
	H	-1.7099029	-1.0532064	2.7100437
	H	-0.3213739	1.7808646	-0.2088063
	H	-2.4857529	1.0550436	-0.8611463
	H	4.1542661	3.1287256	-0.0506263
	H	5.5005461	1.9478856	0.0215337
	H	4.2096161	1.7735356	-1.2060563
	H	1.2205371	-2.0978654	0.9941037
	C	3.0430271	-1.7371254	-0.1416763
	C	5.7738671	-2.2004044	-0.6457063
	C	3.6705171	-1.1786844	-1.2689463
	C	3.8128571	-2.5260644	0.7296737
	C	5.1633671	-2.7587044	0.4799337
	C	5.0215971	-1.4092044	-1.5177263
	H	3.1060071	-0.5463854	-1.9467863
	H	3.3420271	-2.9574354	1.6097737
	H	5.7397271	-3.3758644	1.1638737
	H	5.4893771	-0.9685544	-2.3942563
	H	6.8274571	-2.3800144	-0.8418863
	C	-0.4312639	2.0584046	-2.2645863
	C	-0.4970139	3.7909946	-0.5018263
	H	-0.7617539	3.9483946	0.5451837
	H	-1.0534539	4.4820346	-1.1397363
	H	0.5770161	3.9196446	-0.6308363
	H	-0.9589039	2.7180546	-2.9581763
	H	0.6459161	2.1996446	-2.3397263
	H	-0.6949329	1.0169446	-2.4543563

Name	TS_{Theo2O} / $TS_{Theo2O-BMA}$
**Absolute Energies (Hartree)**	There are 15 positive frequencies below 100 cm^-1.<br><br>Zero-point correction: 0.533055 hartree<br>Enthalpy correction: 0.570781 hartree<br>Free Energy correction: 0.463445 hartree<br>Quasiharmonic Free Energy correction: 0.472907 hartree<br><br>SCF Energy: -1657.376817 hartree<br>SCF Energy*: -1657.863959 hartree<br>SCF Energy + ZPVE: -1656.843762 hartree<br>Enthalpy: -1656.806036 hartree<br>Free Energy: -1656.913372 hartree<br><br>Free Energy with quasiharmonic correction: -1656.903909 hartree (correction: 5.94 kcal/mol)<br><br>---<br>Low frequencies before projection:<br>-218.9657<br>14.0058<br>17.4771<br><br>Low frequencies after projection:<br>-218.9656<br>13.8851<br>16.7371<br>---
**Cartesian Coordinates**	N -2.3345428 0.9493901 1.5416895<br>C -1.9108618 1.9832201 0.8092995<br>C -0.8370018 4.0629701 -0.7433005<br>C -2.7729118 2.8968611 0.1444995<br>N -0.5658518 2.1879501 0.6819795<br>C -0.0324418 3.1768901 -0.0748905<br>C -2.2354418 3.9080701 -0.6188805<br>N -3.9847228 -0.2037489 -1.4031105<br>C -4.4412128 -1.4167289 -0.9264205<br>O -5.5961628 -1.8150089 -1.0992705<br>N -3.5122828 -2.1006289 -0.1654305<br>N -0.2668228 -0.5061699 -1.0706005<br>O -1.1038228 0.3114001 -1.5578705<br>O -0.6434528 -1.5881599 -0.5257905<br>C 1.0645372 -0.2049199 -1.1711905<br>C 2.0450472 -1.0756299 -0.6790005<br>C 2.3492572 -0.7302999 1.4642495<br>C 1.0152372 -0.9572699 1.9591095<br>O 0.1416172 -0.0914699 2.1107095<br>O 0.7752072 -2.2565399 2.2291695<br>C -0.5853528 -2.5802799 2.5542895

	C	2.8685972	0.6224401	1.4150695
	O	2.2221682	1.6511701	1.2150195
	O	4.2199272	0.6391691	1.5277495
	C	4.8440782	1.9186991	1.3629395
	C	-3.7944228	-3.4742589	0.2094395
	C	-4.7665628	0.5079911	-2.3956505
	C	-3.7337528	0.6795111	1.8345495
	H	3.0680272	-1.5181009	1.6528795
	H	-4.7856328	-3.5359589	0.6656095
	H	-3.7741828	-4.1682389	-0.6444705
	H	-3.0475928	-3.8020889	0.9395695
	H	-2.5301028	-1.8735499	-0.3284205
	H	-2.9732028	-0.0667389	-1.4382805
	H	-4.7655528	0.0159611	-3.3802305
	H	-4.3526118	1.5145611	-2.5116005
	H	-5.8048928	0.5854711	-2.0630105
	H	-0.6111128	-3.6669299	2.6519095
	H	1.2579272	0.7454801	-1.6415605
	H	-4.1553918	1.4489111	2.4938495
	H	-3.7899928	-0.2873789	2.3348095
	H	-0.8832128	-2.1133599	3.4989895
	H	-1.2532728	-2.2522799	1.7536895
	H	-1.6198928	0.3136001	1.8963695
	H	0.0894482	1.5361801	1.1478595
	H	1.0492082	3.1940801	-0.0864605
	H	-0.4027518	4.8523001	-1.3442905
	H	-2.9017318	4.5926711	-1.1359305
	H	-3.8434918	2.7666011	0.2287695
	H	-4.3187528	0.6186711	0.9136795
	H	4.5362382	2.6080991	2.1557495
	H	5.9174482	1.7307691	1.4225795
	H	4.5944382	2.3569191	0.3925095
	H	1.7008572	-2.0793399	-0.4523505
	C	3.4484672	-0.9736509	-1.1326905
	C	6.1359272	-0.8675609	-1.9643305
	C	4.0052772	0.2246191	-1.6118505
	C	4.2665572	-2.1139309	-1.0723705
	C	5.5958772	-2.0638909	-1.4865305
	C	5.3350372	0.2757791	-2.0240705
	H	3.4028782	1.1267891	-1.6469205
	H	3.8508072	-3.0457009	-0.6962505
	H	6.2102772	-2.9588309	-1.4367205
	H	5.7479882	1.2115391	-2.3915905
	H	7.1726372	-0.8260909	-2.2872505

Name	TS_{Theo2S} / $TS_{Theo2S\text{-}BMA}$
**Absolute Energies (Hartree)**	There are 15 positive frequencies below 100 cm^-1.<br><br>Zero-point correction: 0.530874 hartree<br>Enthalpy correction: 0.568716 hartree<br>Free Energy correction: 0.461941 hartree<br>Quasiharmonic Free Energy correction: 0.470780 hartree<br><br>SCF Energy: -1980.340601 hartree<br>SCF Energy*: -1980.825624 hartree<br>SCF Energy + ZPVE: -1979.809727 hartree<br>Enthalpy: -1979.771885 hartree<br>Free Energy: -1979.878660 hartree<br><br>Free Energy with quasiharmonic correction: -1979.869822 hartree (correction: 5.55 kcal/mol)<br><br>---<br>Low frequencies before projection:<br>-196.7964<br>15.2068<br>23.5843<br><br>Low frequencies after projection:<br>-196.7961<br>15.0754<br>23.523<br>---
**Cartesian Coordinates**	N -2.3669848 0.8444552 1.5127078<br>C -1.9492748 1.9117152 0.8223378<br>C -0.8926038 4.0370152 -0.6782122<br>C -2.8176338 2.8627952 0.2241278<br>N -0.6058048 2.1052742 0.6663778<br>C -0.0803138 3.1164942 -0.0671622<br>C -2.2884538 3.8978052 -0.5149922<br>N -3.8904748 -0.1135438 -1.5081622<br>C -4.4102058 -1.2076338 -0.9088822<br>N -3.4940058 -2.0442248 -0.3706922<br>N -0.2304458 -0.5289058 -1.1870122<br>O -1.0471848 0.3163652 -1.6641222<br>O -0.6359658 -1.6229558 -0.6888422<br>C 1.1065952 -0.2409258 -1.2569422<br>C 2.0673842 -1.1352758 -0.7817322<br>C 2.3506142 -0.8057868 1.4353678<br>C 1.0100742 -1.0567858 1.8844978<br>O 0.1195052 -0.2051558 2.0274378<br>O 0.7748942 -2.3654658 2.1238478<br>C -0.5914658 -2.7048058 2.4100478<br>C 2.8514252 0.5500932 1.3827678

	O	2.1905852	1.5749542	1.2058078
	O	4.2059752	0.5816932	1.4712678
	C	4.8135752	1.8702732	1.3017778
	C	-3.8103458	-3.2933838	0.2942878
	C	-4.6669448	0.9075562	-2.1826622
	C	-3.7530848	0.6084362	1.8851978
	H	3.0741942	-1.5942268	1.5980178
	H	-2.8667768	-3.7694648	0.5731078
	H	-4.4061658	-3.1285538	1.1993678
	H	-4.3713868	-3.9711938	-0.3593622
	H	-2.5028358	-1.8362748	-0.5220622
	H	-2.8708148	-0.0226948	-1.5450622
	H	-5.2310548	0.4978262	-3.0289422
	H	-3.9691948	1.6631562	-2.5523622
	H	-5.3810548	1.3855662	-1.5028522
	H	-0.6161768	-3.7940258	2.4737978
	H	1.3171552	0.7222942	-1.6934822
	H	-4.1139148	1.3740962	2.5831878
	H	-3.8059748	-0.3660238	2.3723578
	H	-0.9132958	-2.2680348	3.3611378
	H	-1.2435058	-2.3545548	1.6063578
	H	-1.6498348	0.2007352	1.8470278
	H	0.0532952	1.4369442	1.1024478
	H	1.0007062	3.1249242	-0.1087522
	H	-0.4651038	4.8437342	-1.2608022
	H	-2.9600238	4.6129452	-0.9811222
	H	-3.8871538	2.7493562	0.3422878
	H	-4.4061548	0.5891262	1.0094678
	H	4.5255162	2.5469032	2.1128778
	H	5.8900652	1.6925622	1.3261778
	H	4.5299752	2.3176432	0.3451678
	H	1.7117542	-2.1330458	-0.5494422
	C	3.4810842	-1.0402268	-1.1877522
	C	6.1944142	-0.9577478	-1.9309422
	C	4.0609352	0.1527832	-1.6549322
	C	4.2880842	-2.1877368	-1.0952022
	C	5.6306142	-2.1490678	-1.4654922
	C	5.4042452	0.1916822	-2.0229422
	H	3.4683252	1.0599132	-1.7166822
	H	3.8526442	-3.1136068	-0.7269522
	H	6.2370742	-3.0477078	-1.3910522
	H	5.8364152	1.1227222	-2.3798422
	H	7.2418042	-0.9247678	-2.2185522
	S	-6.0973158	-1.4860338	-0.7966422

<table>
<tr><td>Name</td><td>TS_{Theo2S-BMB}</td></tr>
<tr><td>Absolute Energies (Hartree)</td><td>There are 14 positive frequencies below 100 cm^-1.

Zero-point correction: 0.530947 hartree
Enthalpy correction: 0.568708 hartree
Free Energy correction: 0.462337 hartree
Quasiharmonic Free Energy correction: 0.471240 hartree

SCF Energy: -1980.338373 hartree
SCF Energy*: -1980.822654 hartree
SCF Energy + ZPVE: -1979.807426 hartree
Enthalpy: -1979.769665 hartree
Free Energy: -1979.876036 hartree

Free Energy with quasiharmonic correction: -1979.867132 hartree (correction: 5.59 kcal/mol)

Low frequencies before projection:
-195.1545
14.4215
25.7304

Low frequencies after projection:
-195.1544
13.8205
25.7192
--</td></tr>
<tr><td>Cartesian Coordinates</td><td>C 2.5294288 2.0941628 0.3777414
S 4.0890988 2.6760718 0.7571214
N 1.7990488 1.3169728 1.2274414
N 1.9308088 2.3298528 -0.8084186
H 1.0578688 1.8397828 -1.0072686
C 2.1408488 1.1516628 2.6297114
H 0.8058488 1.2456628 1.0034014
H 3.1260788 0.6889218 2.7332714
N 2.6544988 -0.9161672 -1.1088186
C 3.1025188 -0.4149882 -2.3981386
H 2.2298688 0.0004228 -2.8978586
H 3.8507188 0.3752718 -2.2757086
H 3.5258288 -1.2182182 -3.0141186
H -3.1431812 0.4867238 -1.6423986
C -1.9893412 1.5854138 -0.1773286
C -2.1716312 0.5661138 -1.1741386
O -0.9694812 1.8184728 0.4752714
C -1.0517512 0.0380528 -1.9098186
O 0.1357788 0.3731328 -1.8326386
C -2.5971912 -1.1939662 0.2728914
C -1.6611412 -1.1469972 1.3024714</td></tr>
</table>

	C	-4.0256412	-0.9421462	0.5066114
	H	-2.3549022	-1.8294462	-0.5710986
	N	-0.3777412	-1.5684072	1.0935414
	H	-1.8261112	-0.7226772	2.2801314
	O	-0.0001722	-2.0329672	-0.0168586
	O	0.4486888	-1.4884172	2.0638714
	C	-4.9630812	-1.4809162	-0.3930086
	C	-4.4963412	-0.1708262	1.5845914
	C	-5.8611912	0.0387038	1.7633614
	C	-6.3280012	-1.2720362	-0.2129386
	C	-6.7822412	-0.5117152	0.8679014
	H	-4.6090622	-2.0645162	-1.2393786
	H	-3.7940212	0.2788638	2.2789914
	H	-6.2076712	0.6383538	2.6007714
	H	-7.0373722	-1.7004652	-0.9157786
	H	-7.8466812	-0.3447752	1.0090514
	H	2.1634988	2.1097328	3.1656014
	H	1.3885088	0.4977228	3.0741914
	C	2.5033998	3.1188428	-1.8779186
	H	3.4653388	2.7165818	-2.2167686
	H	1.7965298	3.1073628	-2.7123586
	H	2.6702498	4.1553528	-1.5641886
	C	3.4884188	-1.3726682	-0.1708986
	C	5.1121478	-2.3187082	1.9226314
	C	4.8919088	-1.5105482	-0.3564886
	N	2.9873878	-1.7221482	1.0478414
	C	3.7564578	-2.1785482	2.0668514
	C	5.6769578	-1.9717082	0.6756414
	H	5.3281488	-1.2385582	-1.3083086
	H	3.2151478	-2.4102982	2.9765414
	H	6.7484478	-2.0643182	0.5250214
	H	5.7188378	-2.6787782	2.7441314
	O	-3.1542512	2.2435538	0.0814914
	O	-1.4469212	-0.9449472	-2.7633586
	C	-3.1093302	3.1737338	1.1695414
	H	-2.4307302	4.0041938	0.9489914
	H	-4.1305502	3.5427438	1.2830014
	H	-2.7781412	2.6892938	2.0935414
	C	-0.3941122	-1.6203972	-3.4615386
	H	-0.8850922	-2.3963072	-4.0524086
	H	0.1446288	-0.9350772	-4.1240086
	H	0.3076978	-2.0744672	-2.7556986
	H	1.9693778	-1.6280572	1.2542914
	H	1.6630588	-0.8165472	-0.8977986

Name	*(R)*-TS$_{1S}$ / *(R)*-TS$_{1S\text{-}BMA}$
**Absolute Energies (Hartree)**	There are 19 positive frequencies below 100 cm^-1.<br><br>Zero-point correction: 0.656952 hartree<br>Enthalpy correction: 0.704172 hartree<br>Free Energy correction: 0.574147 hartree<br>Quasiharmonic Free Energy correction: 0.589607 hartree<br><br>SCF Energy: -2832.536500 hartree<br>SCF Energy*: -2833.281461 hartree<br>SCF Energy + ZPVE: -2831.879548 hartree<br>Enthalpy: -2831.832328 hartree<br>Free Energy: -2831.962353 hartree<br><br>Free Energy with quasiharmonic correction: -2831.946892 hartree (correction: 9.70 kcal/mol)<br><br>--<br>Low frequencies before projection:<br>-210.5053<br>10.3118<br>12.6339<br><br>Low frequencies after projection:<br>-210.5051<br>10.2138<br>11.8619<br>--
**Cartesian Coordinates**	N 1.4216686 1.4614743 -0.7384483<br>N 3.0508276 -0.0810557 -0.6753483<br>N -0.3667414 1.8623253 1.6984817<br>C 0.5996086 2.6537853 -0.5388283<br>C -0.6003204 4.2801653 0.9796417<br>C 0.2030196 5.0552553 -1.2870983<br>C -0.0213804 5.4499753 0.1720617<br>C 1.1406296 3.8502243 -1.3507383<br>C 0.3033086 3.0319753 0.9399517<br>C 2.7036686 1.2189643 -0.3799283<br>C 4.2590876 -0.7603067 -0.4700383<br>C 6.5949976 -2.2995067 -0.1345783<br>C 5.5206876 -0.1779867 -0.6542483<br>C 4.1756076 -2.1218457 -0.1381883<br>C 5.3350576 -2.8767767 0.0202217<br>C 6.6681876 -0.9494167 -0.4746583<br>C 8.0144776 -0.2921077 -0.6144383<br>C 5.2246366 -4.3180067 0.4350017<br>F 8.9738676 -1.1748877 -0.9782083<br>F 8.4215286 0.2673723 0.5503417<br>F 8.0043186 0.6953223 -1.5382483

	F	6.2613066	-5.0572567	-0.0216783
	F	5.2167566	-4.4516867	1.7845717
	F	4.0872566	-4.8922557	-0.0183383
	H	0.9093286	0.7063043	-1.2060983
	H	2.2449576	-0.7064357	-0.8019483
	H	-1.1281414	1.4865653	1.0900817
	H	-0.3653214	2.3753153	-0.9698483
	H	-1.5866804	4.0126053	0.5798617
	H	0.6286396	5.8914853	-1.8544683
	H	0.9329196	5.7656443	0.6162217
	H	1.2730286	3.5049743	-2.3826283
	H	1.2378386	3.2248243	1.4705117
	H	7.4945376	-2.8904467	-0.0122383
	H	5.6034986	0.8594733	-0.9443683
	H	3.2021276	-2.5856457	-0.0119783
	H	-0.7369004	4.6102753	2.0125517
	H	-0.7622504	4.8085253	-1.7532883
	H	-0.7062004	6.3029253	0.2495417
	H	2.1292396	4.1266943	-0.9744883
	N	-0.3660824	-1.4329447	-0.8920683
	C	-1.4319724	-2.2244047	-0.5878783
	C	-2.6934524	-1.9770847	-1.1376083
	C	-3.7721424	-2.9765137	-1.0526583
	C	-5.8707634	-4.8487737	-0.9819583
	C	-3.8463034	-3.9321437	-0.0236583
	C	-4.7779424	-2.9686537	-2.0353583
	C	-5.8149534	-3.8978437	-2.0040783
	C	-4.8842034	-4.8600837	0.0076817
	O	0.7589776	-1.7006257	-0.3553883
	O	-0.4791924	-0.4546147	-1.6923783
	H	-1.2080424	-2.9960447	0.1317117
	H	-2.7139424	-1.3343947	-2.0105183
	H	-3.0990034	-3.9444837	0.7629817
	H	-4.7402424	-2.2229137	-2.8256083
	H	-6.5791934	-3.8802127	-2.7763383
	H	-4.9261334	-5.5923837	0.8094817
	H	-6.6794734	-5.5740527	-0.9544683
	C	-3.6983524	-0.2956437	-0.0111683
	C	-3.1787714	0.8873163	-0.6552583
	C	-3.1934214	2.1388463	-2.6615983
	C	-3.2798524	-0.6397437	1.3236217
	C	-3.7405024	-2.0316537	3.1782417
	O	-2.3553614	1.6977853	-0.2141483
	O	-3.7182914	1.0496863	-1.8890383
	O	-2.2233624	-0.3190947	1.8701217
	O	-4.1559224	-1.4960437	1.9164317
	H	-4.6863424	-0.6082237	-0.3202283
	H	-3.7482714	2.1249163	-3.6014583
	H	-3.3431514	3.0946463	-2.1502183
	H	-2.1258314	1.9936253	-2.8540583

	H	-3.6731024	-1.2444337	3.9367617
	H	-4.5086724	-2.7548337	3.4581417
	H	-2.7668324	-2.5252347	3.0980217
	C	-1.0489114	2.2900153	2.9623317
	H	-0.3217014	2.7750953	3.6171417
	H	-1.8706314	2.9642153	2.7330317
	H	-1.4544514	1.3955153	3.4309717
	C	0.5746286	0.7352853	2.0278417
	H	1.5011786	1.1542343	2.4218517
	H	0.0928376	0.0966253	2.7647017
	H	0.7717876	0.1429143	1.1417617
	S	3.7126786	2.3255243	0.4196317

Name	*(S)*-TS_{1S} / *(S)*-$TS_{1S\text{-}BMA}$
**Absolute Energies (Hartree)**	There are 19 positive frequencies below 100 cm^-1.<br><br>Zero-point correction: 0.657532 hartree<br>Enthalpy correction: 0.704590 hartree<br>Free Energy correction: 0.575187 hartree<br>Quasiharmonic Free Energy correction: 0.590325 hartree<br><br>SCF Energy: -2832.531482 hartree<br>SCF Energy*: -2833.275755 hartree<br>SCF Energy + ZPVE: -2831.873950 hartree<br>Enthalpy: -2831.826892 hartree<br>Free Energy: -2831.956295 hartree<br><br>Free Energy with quasiharmonic correction: -2831.941157 hartree (correction: 9.50 kcal/mol)<br><br>---<br>Low frequencies before projection:<br>-248.1100<br>10.1968<br>13.4514<br><br>Low frequencies after projection:<br>-248.11<br>10.1397<br>13.3994<br>---
**Cartesian Coordinates**	N 1.3674285 1.6123936 -0.8124060<br>N 2.8706495 -0.0262964 -0.5740960<br>N -0.4104615 2.1479926 1.6387740<br>C 0.5807785 2.8315026 -0.6518660<br>C -0.6408425 4.5117326 0.7719340<br>C 0.2544575 5.2121926 -1.4903260<br>C -0.0393935 5.6549226 -0.0579260<br>C 1.1806075 3.9970536 -1.4691860<br>C 0.2656675 3.2648326 0.8094440<br>C 2.6085185 1.3089936 -0.3662460<br>C 4.0211195 -0.7863754 -0.3418860<br>C 6.2213805 -2.5003244 0.0679440<br>C 5.3295895 -0.2908744 -0.4373860<br>C 3.8254105 -2.1480454 -0.0595260<br>C 4.9172905 -2.9885254 0.1384040<br>C 6.4070105 -1.1491844 -0.2231560<br>C 7.8057695 -0.6210634 -0.3945260<br>C 4.6771315 -4.4252354 0.5113640<br>F 7.8984295 0.6906366 -0.0864260<br>F 8.2356895 -0.7535434 -1.6741660<br>F 8.6943105 -1.2836334 0.3825840

	F	4.5230815	-4.5758754	1.8504740
	F	3.5572315	-4.9183754	-0.0647860
	F	5.7060025	-5.2232544	0.1463740
	H	0.9208195	0.9026126	-1.4032560
	H	2.0256195	-0.5885264	-0.7291460
	H	-1.1846315	1.7417616	1.0684540
	H	-0.3815115	2.5751126	-1.1017260
	H	-1.6172225	4.2259316	0.3553440
	H	0.7146765	6.0275326	-2.0609560
	H	0.8915965	5.9940426	0.4172040
	H	1.3617175	3.6199436	-2.4821160
	H	1.1943975	3.4878836	1.3386840
	H	7.0687415	-3.1554044	0.2299940
	H	5.5015995	0.7495056	-0.6719460
	H	2.8153905	-2.5425164	0.0012040
	H	-0.8120425	4.8838226	1.7842940
	H	-0.6878825	4.9577226	-1.9977860
	H	-0.7326335	6.5043326	-0.0404960
	H	2.1529975	4.2694236	-1.0487860
	N	-0.3611695	-1.1369474	-1.4766560
	C	-1.5790695	-1.7011684	-1.6654960
	C	-2.3356095	-2.1731984	-0.5687460
	C	-3.4144285	-3.1665894	-0.7620160
	C	-5.4460485	-5.0892004	-1.0523860
	C	-4.1872185	-3.2197594	-1.9354560
	C	-3.6882885	-4.0821094	0.2668440
	C	-4.6909285	-5.0397494	0.1207040
	C	-5.1925585	-4.1726694	-2.0777060
	O	0.1842295	-0.4877274	-2.4320860
	O	0.2642805	-1.2634874	-0.3687560
	H	-1.7568195	-2.2935084	0.3415540
	H	-1.9687395	-1.5775684	-2.6637660
	H	-4.0147795	-2.5009694	-2.7307060
	H	-5.7815385	-4.2005304	-2.9905860
	H	-6.2293175	-5.8336004	-1.1674960
	H	-4.8831275	-5.7458794	0.9240540
	H	-3.1085485	-4.0386084	1.1844240
	C	-3.0301205	0.5415716	-0.6356860
	C	-3.5223705	-0.5451794	0.1844940
	C	-3.2426305	-0.5691094	1.6125640
	C	-3.7498795	-1.7014794	3.6257540
	C	-2.9547015	1.3638716	-2.8479160
	O	-2.3913305	0.0721516	2.2210640
	O	-4.0223995	-1.4930694	2.2327440
	O	-2.1396415	1.3519916	-0.3641160
	O	-3.5833805	0.5174106	-1.8662060
	H	-3.5034615	1.1877406	-3.7744460
	H	-1.9029015	1.0848316	-2.9687860
	H	-3.0277015	2.4172116	-2.5617060
	H	-4.4307695	-2.4935494	3.9420240

	H	-3.9378005	-0.7905294	4.2028640	
	H	-2.7113195	-2.0112684	3.7798440	
	H	-4.4808105	-0.9484594	-0.1132260	
	C	0.5022995	0.9979426	1.9739740	
	C	-1.0186715	2.6633326	2.9080440	
	H	-1.8525625	3.3246916	2.6834840	
	H	-1.3961015	1.8092716	3.4646240	
	H	-0.2517625	3.1902126	3.4803340	
	H	0.0373995	0.4222226	2.7714540	
	H	1.4696785	1.3892736	2.2889340	
	H	0.5991895	0.3601826	1.1020740	
	S	3.6429385	2.3871746	0.4397440	

Name	*(R)*-**TS$_{1S-BMB}$**
**Absolute Energies (Hartree)**	There are 17 positive frequencies below 100 cm^-1.<br><br>Zero-point correction: 0.656862 hartree<br>Enthalpy correction: 0.703658 hartree<br>Free Energy correction: 0.577547 hartree<br>Quasiharmonic Free Energy correction: 0.589803 hartree<br><br>SCF Energy: -2832.556359 hartree<br>SCF Energy*: -2833.297671 hartree<br>SCF Energy + ZPVE: -2831.899497 hartree<br>Enthalpy: -2831.852701 hartree<br>Free Energy: -2831.978812 hartree<br><br>Free Energy with quasiharmonic correction: -2831.966556 hartree (correction: 7.69 kcal/mol)<br><br>---<br>Low frequencies before projection:<br>-215.5799<br>9.3444<br>15.1512<br><br>Low frequencies after projection:<br>-215.5798<br>7.5499<br>14.7045<br>---
**Cartesian Coordinates**	C -2.3423464 -3.0894713 -0.0329361<br>C -2.0585874 -1.9459013 0.7275339<br>C -3.1203774 -1.1246123 1.1460139<br>C -4.4315874 -1.4567623 0.8187439<br>C -4.7257164 -2.6083023 0.0882339<br>C -3.6659764 -3.4108323 -0.3299761<br>N -0.7678474 -1.4999113 1.0258839<br>C 0.4247526 -2.1744503 1.0566739<br>S 0.5805236 -3.8616103 1.2158039<br>C -5.5539474 -0.5275433 1.1792039<br>C -3.9570064 -4.6783423 -1.0877361<br>N 1.4730426 -1.3250403 0.9848139<br>C 2.8804226 -1.6579903 1.1177439<br>C 3.6667226 -1.1551693 -0.1153061<br>C 5.1611126 -1.4710793 0.0139439<br>C 5.7319426 -0.8423883 1.2959139<br>C 4.9653926 -1.3163193 2.5357539<br>C 3.4674226 -1.0248293 2.3950239<br>N 3.0655126 -1.6746493 -1.4179461<br>O 0.4621826 -1.0111203 -1.8599161

	N	0.4029726	0.2694897	-1.8789761
	O	1.3967916	0.9429897	-2.2601561
	C	-0.7683884	0.8578887	-1.4901861
	C	-0.8892584	2.2495487	-1.4550461
	C	-2.1960484	2.9232787	-1.3545961
	C	-3.3568284	2.2817377	-0.8866961
	C	-4.5585384	2.9797277	-0.7818761
	C	-4.6252494	4.3273777	-1.1428161
	C	-3.4811594	4.9752777	-1.6154661
	C	-2.2800094	4.2793587	-1.7168661
	C	0.0106106	3.0014987	0.4674239
	C	1.3555816	2.5010397	0.3366139
	O	1.7962716	1.4084597	0.7052239
	C	-0.9282484	2.4084287	1.3881439
	O	-0.9830284	1.2281087	1.7479639
	H	2.9592036	-2.7444803	1.1953039
	H	3.2929626	0.0579107	2.3586239
	H	2.9077926	-1.4158103	3.2516039
	H	5.3577226	-0.8285793	3.4358739
	H	5.1183126	-2.3968993	2.6681539
	H	5.6665226	0.2522407	1.2216939
	H	6.7962226	-1.0918183	1.3777339
	H	5.3114436	-2.5576493	0.0506939
	H	5.7099926	-1.0942283	-0.8553461
	H	3.5178726	-0.0729393	-0.1794561
	H	-0.7125974	-0.4895813	1.1994239
	H	1.2869626	-0.3175903	0.9200439
	H	-0.1167794	4.0598287	0.2790739
	H	-1.5244674	0.1577887	-1.1738661
	H	-0.1141084	2.7997987	-1.9774261
	H	-1.3870794	4.7880387	-2.0725361
	H	-3.5244904	6.0225977	-1.9020161
	H	-5.5637094	4.8685967	-1.0575861
	H	-5.4404084	2.4673767	-0.4128761
	H	-3.3305084	1.2365177	-0.5956061
	H	-2.9026974	-0.2303723	1.7183839
	H	-5.7493364	-2.8643433	-0.1587361
	H	-1.5383764	-3.7125313	-0.3956561
	F	-5.2193584	0.3442767	2.1587539
	F	-6.6570174	-1.1908233	1.5868939
	F	-5.9320174	0.2214367	0.1057239
	F	-2.9498264	-5.0159823	-1.9234161
	F	-4.1429754	-5.7289723	-0.2530561
	F	-5.0818564	-4.5723423	-1.8342861
	C	3.4599816	2.9442907	-0.6383161
	H	3.3944616	2.0941307	-1.3240161
	H	4.0230016	2.6558207	0.2554439
	C	-2.9294684	2.8112477	2.5963539
	H	-2.5504684	2.3142377	3.4943239
	H	-3.5514384	2.1150777	2.0302139

	O	2.1483206	3.4025997	-0.2998761
	O	-1.8537794	3.3123987	1.7902339
	H	3.9498306	3.7875607	-1.1297661
	H	-3.5190294	3.6880477	2.8702939
	H	2.0569326	-1.3551603	-1.4548661
	C	3.6956526	-1.0583293	-2.6261961
	H	3.0261426	-1.2229193	-3.4709561
	H	4.6637526	-1.5238893	-2.8144561
	H	3.8000726	0.0131007	-2.4662661
	H	2.5112936	-3.3895703	-2.4988861
	C	3.0158536	-3.1624293	-1.5577361
	H	2.4409036	-3.5904803	-0.7360561
	H	4.0294736	-3.5650893	-1.5757361

<table>
<tr><td>Name</td><td>(S)-TS_{1S-BMB}</td></tr>
<tr><td>Absolute Energies (Hartree)</td><td>There are 17 positive frequencies below 100 cm^-1.

Zero-point correction: 0.657518 hartree
Enthalpy correction: 0.704055 hartree
Free Energy correction: 0.578755 hartree
Quasiharmonic Free Energy correction: 0.590755 hartree

SCF Energy: -2832.552243 hartree
SCF Energy*: -2833.298289 hartree
SCF Energy + ZPVE: -2831.894725 hartree
Enthalpy: -2831.848188 hartree
Free Energy: -2831.973488 hartree

Free Energy with quasiharmonic correction: -2831.961488 hartree (correction: 7.53 kcal/mol)

Low frequencies before projection:
-217.5554
12.0224
14.0037

Low frequencies after projection:
-217.5553
11.572
13.797
---</td></tr>
<tr><td>Cartesian Coordinates</td><td>C -2.6403616 3.6936146 -1.8385913
C -2.4536726 2.4189646 -1.2754313
C -3.5629626 1.7602146 -0.7202813
C -4.8277826 2.3451546 -0.7444913
C -5.0018216 3.6073046 -1.3145513
C -3.9028316 4.2804546 -1.8577413
C -1.1374426 1.7547146 -1.2363113
C -0.1978926 1.9453746 -2.2593013
N 0.8951474 1.1368936 -2.3529213
O 1.7890174 1.3670136 -3.2102913
O 1.0024374 0.1375736 -1.5523213
C -0.3194026 2.6933046 0.6143187
C -1.1689626 2.1171246 1.6330387
O -1.0665626 0.9974846 2.1474987
C 1.0941774 2.3850436 0.5523287
O 1.7228074 1.4815636 1.1098987
N 3.4508574 -1.0027964 -1.3236613
C 4.3598974 -0.2207164 -2.2191313
C 3.8974474 -0.9700664 0.1349187
C 2.9398974 -1.7605164 1.0516187
C 3.3732474 -1.5793564 2.5204487</td></tr>
</table>

	C	4.8233674	-2.0239564	2.7404787
	C	5.7723874	-1.2833464	1.7919087
	C	5.3418474	-1.4496164	0.3249587
	N	1.5675674	-1.3273164	0.8643087
	C	0.4906374	-2.1283454	0.7032587
	N	-0.6817926	-1.4313154	0.8194487
	C	-1.9882726	-1.8404854	0.5168987
	S	0.5815464	-3.8057154	0.4242287
	C	3.2083874	-2.3622364	-1.8963613
	H	2.9861164	-2.8234964	0.8037087
	H	3.2514574	-0.5228164	2.7950887
	H	2.6860674	-2.1569364	3.1477587
	H	5.1145074	-1.8510964	3.7831587
	H	4.9022364	-3.1060164	2.5638387
	H	5.7808674	-0.2134564	2.0438787
	H	6.8002274	-1.6453874	1.9099987
	H	5.4193164	-2.5071564	0.0407487
	H	6.0232974	-0.8871064	-0.3206613
	H	3.8186074	0.0906836	0.3992287
	H	-0.6302426	-0.5159854	1.2874187
	H	1.4096574	-0.3199364	0.9861487
	H	2.8760274	-2.2301364	-2.9279513
	H	4.1334264	-2.9408764	-1.8770013
	H	2.4276764	-2.8710164	-1.3303313
	H	3.7928774	0.0447236	-3.1108213
	H	4.6693674	0.6914536	-1.7076813
	H	5.2340874	-0.8210064	-2.4735013
	H	-0.5733916	3.7024546	0.3169287
	H	2.5119974	-0.5088964	-1.3731613
	H	-1.1187626	0.7775246	-0.7680513
	H	-0.1930526	2.7716246	-2.9534813
	H	-3.4314026	0.7814946	-0.2689213
	H	-5.6693726	1.8141256	-0.3116713
	H	-5.9858116	4.0681956	-1.3313213
	H	-4.0305716	5.2675346	-2.2941313
	H	-1.7916716	4.2351446	-2.2469413
	C	-3.0205826	-1.3750154	1.3413287
	C	-4.3487226	-1.6657454	1.0295187
	C	-4.6748836	-2.4326654	-0.0860113
	C	-3.6381436	-2.8959554	-0.8988213
	C	-2.3065836	-2.5992554	-0.6188113
	H	-2.7770626	-0.7732254	2.2093087
	C	-5.4305126	-1.0621544	1.8769687
	H	-5.7076036	-2.6572044	-0.3232313
	C	-3.9655336	-3.7629254	-2.0843613
	H	-1.5209336	-2.9372854	-1.2799713
	F	-5.6609726	0.2392256	1.5294687
	F	-6.6082926	-1.7071444	1.7560587
	F	-5.1027926	-1.0442054	3.1874887
	F	-3.0464836	-3.6479354	-3.0687813

	F	-4.0176936	-5.0734954	-1.7469713
	F	-5.1702036	-3.4512554	-2.6181213
	O	1.7046674	3.2742236	-0.2765413
	O	-2.1960626	2.9420046	1.9398987
	C	3.0791874	3.0441236	-0.5986413
	H	3.1474674	2.2908836	-1.3895213
	H	3.6493474	2.7341736	0.2810787
	H	3.4601984	3.9951936	-0.9769213
	C	-3.2078526	2.4115146	2.8101587
	H	-3.8864126	3.2436246	3.0057487
	H	-2.7723926	2.0496046	3.7457887
	H	-3.7556026	1.6012446	2.3234487

Name	*(R)*-TS_{1O} / *(R)*-TS_{1O-BMA}
**Absolute Energies (Hartree)**	There are 17 positive frequencies below 100 cm^-1.<br><br>Zero-point correction: 0.659331 hartree<br>Enthalpy correction: 0.705916 hartree<br>Free Energy correction: 0.578985 hartree<br>Quasiharmonic Free Energy correction: 0.592574 hartree<br><br>SCF Energy: -2509.592600 hartree<br>SCF Energy*: -2510.337638 hartree<br>SCF Energy + ZPVE: -2508.933269 hartree<br>Enthalpy: -2508.886684 hartree<br>Free Energy: -2509.013615 hartree<br><br>Free Energy with quasiharmonic correction: -2509.000026 hartree (correction: 8.53 kcal/mol)<br><br>---<br>Low frequencies before projection:<br>-226.0789<br>13.0665<br>16.1224<br><br>Low frequencies after projection:<br>-226.0789<br>13.0174<br>15.9228<br>---
**Cartesian Coordinates**	C 3.2905544 -4.5772142 0.0438220<br>C 3.2738344 -3.3300942 -0.6049680<br>C 4.4846334 -2.8213242 -1.1065180<br>C 5.6688744 -3.5422232 -0.9719080<br>C 5.6693344 -4.7832032 -0.3297580<br>C 4.4742444 -5.2988742 0.1782420<br>C 1.9940634 -2.6064142 -0.7175880<br>C 1.7857834 -1.6301642 -1.6996080<br>N 0.5380234 -1.1254942 -1.9022280<br>O -0.4623266 -1.6267342 -1.2948780<br>O 0.3788534 -0.1456942 -2.6939780<br>C 1.9299834 -1.6005642 1.2654420<br>C 2.8534134 -0.5362142 0.9675620<br>C 0.5225634 -1.3116842 1.4808520<br>O -0.0428766 -0.2127542 1.4675920<br>O 2.5960834 0.5109558 0.3706920<br>N 0.5352724 2.4488058 1.4034420<br>C 1.6836524 2.4952458 2.3614420<br>C 0.8281724 3.1709958 0.0928620<br>C -0.3874176 3.1274758 -0.8601680<br>C 0.0247924 3.7179158 -2.2220580

	C	0.5475724	5.1524758	-2.0750780
	C	1.7013324	5.2216658	-1.0672680
	C	1.3068924	4.6133658	0.2899220
	N	-0.9174466	1.7840248	-0.9918380
	C	-2.2394066	1.5080448	-0.7410480
	N	-2.5899466	0.2177348	-1.0961780
	C	-3.7641066	-0.4520952	-0.7573080
	C	-4.9380966	0.1773348	-0.3092080
	C	-6.0457166	-0.5969562	0.0359220
	C	-6.0279066	-1.9889662	-0.0439080
	C	-4.8623366	-2.6026952	-0.5020880
	C	-3.7489566	-1.8546852	-0.8697480
	C	-4.7457056	-4.1004652	-0.5034480
	F	-5.9496856	-4.7130162	-0.5239680
	C	-7.3120066	0.1016838	0.4504320
	F	-7.0656566	1.2491638	1.1227220
	F	-4.0386456	-4.5572152	-1.5611080
	F	-4.1007856	-4.5452052	0.6101720
	F	-8.0739266	0.4355138	-0.6205480
	F	-8.0835566	-0.6736062	1.2483920
	C	-0.7193276	2.8652248	2.1045520
	H	2.5614234	-1.0997142	-2.2283680
	H	1.1139134	-3.1589242	-0.4084580
	H	-1.8138066	-0.4012452	-1.3621980
	H	-0.3918666	1.1323758	-1.5824880
	H	-1.1893476	3.7451948	-0.4469880
	H	-0.8446276	3.6841948	-2.8875480
	H	0.7965224	3.0763158	-2.6701180
	H	0.8708524	5.5371558	-3.0495680
	H	-0.2718376	5.8013458	-1.7342980
	H	2.5669024	4.6729858	-1.4643880
	H	2.0241024	6.2589958	-0.9204780
	H	2.1664624	4.6496458	0.9665920
	H	0.5062924	5.2137258	0.7427720
	H	1.6341324	2.5704558	-0.3388680
	H	1.6969724	3.4592558	2.8712220
	H	1.5392934	1.6926258	3.0869620
	H	2.6093024	2.3244058	1.8171020
	H	-0.6398876	3.9168958	2.3857820
	H	-0.8169876	2.2487448	2.9995820
	H	-1.5858076	2.7091948	1.4642920
	H	0.4209734	1.4337858	1.1639020
	H	2.3175534	-2.4568342	1.8023220
	H	2.3622644	-4.9768842	0.4452120
	H	4.4645744	-6.2634742	0.6785120
	H	6.5944944	-5.3436532	-0.2254580
	H	6.5948934	-3.1335432	-1.3676880
	H	4.5055534	-1.8547142	-1.5995180
	H	-2.8511266	-2.3537552	-1.2201580
	H	-6.8908366	-2.5762562	0.2440120

	H	-4.9651066	1.2528648	-0.2143180
	O	4.1296434	-0.8682742	1.3115520
	O	-0.1655066	-2.4471742	1.7417220
	C	5.1496734	0.0376568	0.8801520
	H	6.0968734	-0.4645032	1.0857820
	H	5.1009934	0.9800368	1.4378720
	H	5.0629534	0.2574968	-0.1878980
	C	-1.5886966	-2.3023652	1.8691920
	H	-1.8470066	-1.8967652	2.8539120
	H	-2.0079156	-3.3029952	1.7534720
	H	-1.9760266	-1.6381252	1.0975920
	O	-3.0123176	2.3197748	-0.2183780

Name	*(R)*-TS$_{2S}$ / *(R)*-TS$_{2S-BMA}$
**Absolute Energies (Hartree)**	There are 20 positive frequencies below 100 cm^-1.<br><br>Zero-point correction: 0.742911 hartree<br>Enthalpy correction: 0.795697 hartree<br>Free Energy correction: 0.657372 hartree<br>Quasiharmonic Free Energy correction: 0.670502 hartree<br><br>SCF Energy: -3135.044371 hartree<br>SCF Energy*: -3135.862452 hartree<br>SCF Energy + ZPVE: -3134.301460 hartree<br>Enthalpy: -3134.248674 hartree<br>Free Energy: -3134.386999 hartree<br><br>Free Energy with quasiharmonic correction: -3134.373869 hartree (correction: 8.24 kcal/mol)<br><br>---<br>Low frequencies before projection:<br>-180.6366<br>11.6274<br>20.9814<br><br>Low frequencies after projection:<br>-180.6364<br>11.3469<br>20.9614<br>---
**Cartesian Coordinates**	N 1.0316227 2.0587045 -1.5746388<br>N 2.3110617 0.2100835 -1.6920388<br>N -0.0043073 2.2215945 1.1188612<br>N -0.2595383 -0.0019255 1.7098112<br>N 3.8252417 -0.6259965 2.0307312<br>C 0.5052727 3.3204045 -1.0438388<br>C -0.0322773 4.6602945 1.0479612<br>C 0.2876727 5.8298245 -1.1723988<br>C 0.4811727 5.9243645 0.3472112<br>C 1.0065527 4.5969745 -1.7367388<br>C 0.6342327 3.3847545 0.5059612<br>C 2.2972727 1.5757635 -1.5111188<br>C 3.2872417 -0.7613965 -1.4359588<br>C 5.0463817 -2.8835465 -0.8017088<br>C 4.6733317 -0.5453965 -1.3909588<br>C 2.7988617 -2.0585365 -1.1874288<br>C 3.6710617 -3.0960265 -0.8805788<br>C 5.5237717 -1.6002965 -1.0588288<br>C 6.9887617 -1.3324165 -0.8602388<br>C 3.0991617 -4.4325465 -0.5043588<br>C 0.5825827 1.0515545 1.4646112

	C	1.5483517	-1.4875355	2.1445512
	C	1.9593927	0.8478235	1.6113312
	C	0.2148617	-1.2332455	2.0243312
	C	2.4841917	-0.4197465	1.9335512
	C	4.7449117	0.5070135	1.9540612
	C	4.3625717	-1.8921365	2.5228012
	F	7.7571717	-2.3638865	-1.2764988
	F	7.2747317	-1.1586665	0.4647812
	F	7.4122017	-0.2233465	-1.4963188
	F	3.9697217	-5.4418465	-0.7174088
	F	2.7759917	-4.4764765	0.8228012
	F	1.9656717	-4.7180065	-1.1784688
	H	4.2147717	-2.0074065	3.6062212
	H	0.2783427	1.3764545	-1.6936388
	H	1.3704517	-0.1998055	-1.6914488
	H	-1.0185273	2.1766245	1.0080712
	H	-0.5688673	3.2450545	-1.2530588
	H	-1.1190373	4.5774445	0.8948812
	H	0.6657927	6.7346045	-1.6632488
	H	1.5497327	6.0564545	0.5684812
	H	0.8362227	4.4978445	-2.8153188
	H	1.6946527	3.3966745	0.7641812
	H	5.7225917	-3.6905565	-0.5457688
	H	5.0743817	0.4370635	-1.5866488
	H	1.7317617	-2.2485955	-1.2121188
	H	1.8673617	-2.4926565	2.3770712
	H	2.6156727	1.6809535	1.4216912
	H	-0.5511683	-1.9833855	2.1670812
	H	5.7668917	0.1324035	1.9173412
	H	5.4313417	-1.9217065	2.3075112
	H	0.1294627	4.7118745	2.1307612
	H	-0.7880673	5.7733845	-1.3986988
	H	-0.0367173	6.8039845	0.7487412
	H	2.0836427	4.6987935	-1.5889288
	H	4.6388627	1.1731535	2.8222012
	H	3.9071217	-2.7401965	2.0110112
	H	4.5743627	1.0836735	1.0411612
	N	-1.3630483	-0.5670855	-1.3492888
	C	-2.4046483	-1.4365455	-1.1761988
	C	-3.7289983	-1.0272055	-1.3114188
	C	-4.8268783	-1.9933045	-1.4744388
	C	-6.9786483	-3.7690445	-1.8388888
	C	-4.7442383	-3.3231645	-1.0244088
	C	-6.0125383	-1.5695145	-2.1003688
	C	-7.0768483	-2.4485645	-2.2848188
	C	-5.8093783	-4.2010345	-1.2071388
	O	-0.1850983	-1.0221155	-1.2014188
	O	-1.5459883	0.6432745	-1.6687388
	H	-2.0890383	-2.4286755	-0.8959288
	H	-3.8850783	-0.0509055	-1.7570588

	H	-3.8495883	-3.6717255	-0.5181688
	H	-6.0926083	-0.5400345	-2.4412088
	H	-7.9826583	-2.1038445	-2.7761288
	H	-5.7286683	-5.2248645	-0.8519688
	H	-7.8083183	-4.4564245	-1.9799288
	C	-4.4274983	-0.1271655	0.6867512
	C	-3.7392373	1.1314045	0.6180212
	C	-3.7074973	3.2497845	-0.4272088
	C	-3.9515983	-1.1748755	1.5536612
	C	-4.5224083	-3.2346955	2.5646212
	O	-2.6703173	1.4262445	1.1663712
	O	-4.3960373	2.0198945	-0.1710988
	O	-2.7976283	-1.3381055	1.9531012
	O	-4.9319883	-2.0797845	1.8244112
	H	-5.4785283	-0.1244845	0.4318512
	H	-4.3765473	3.8363645	-1.0596988
	H	-3.5020373	3.7904045	0.5020212
	H	-2.7695273	3.0540145	-0.9557588
	H	-4.1895283	-2.9587855	3.5706812
	H	-5.4048983	-3.8744645	2.6235212
	H	-3.7084683	-3.7633155	2.0583412
	H	-1.2793583	0.1142545	1.6242112
	S	3.6694327	2.5357835	-1.2276388

Name	*(S)*-TS2S / *(S)*-TS2S-BMA
**Absolute Energies (Hartree)**	There are 19 positive frequencies below 100 cm^-1.<br><br>Zero-point correction: 0.743437 hartree<br>Enthalpy correction: 0.795847 hartree<br>Free Energy correction: 0.659753 hartree<br>Quasiharmonic Free Energy correction: 0.671409 hartree<br><br>SCF Energy: -3135.042287 hartree<br>SCF Energy*: -3135.859183 hartree<br>SCF Energy + ZPVE: -3134.298850 hartree<br>Enthalpy: -3134.246440 hartree<br>Free Energy: -3134.382534 hartree<br><br>Free Energy with quasiharmonic correction: -3134.370878 hartree (correction: 7.31 kcal/mol)<br><br>---<br>Low frequencies before projection:<br>-199.9088<br>18.5043<br>19.3072<br><br>Low frequencies after projection:<br>-199.9087<br>17.8348<br>19.0653<br>---
**Cartesian Coordinates**	N 1.2621502 2.3303636 -1.6813773<br>N 2.0206612 0.2131546 -1.6905073<br>N 0.3045302 2.2891436 1.0490027<br>N -0.2553088 0.1816036 1.8099127<br>N 3.6866012 -1.0462254 2.0840427<br>C 0.9762202 3.5783336 -0.9662673<br>C 0.6786802 4.6992336 1.2927727<br>C 1.2647092 6.0796636 -0.7470073<br>C 1.4347592 5.9281936 0.7707527<br>C 1.7319002 4.8147146 -1.4795973<br>C 1.1125002 3.4091436 0.5748427<br>C 2.3582612 1.5400146 -1.5434273<br>C 2.7359812 -0.9653454 -1.4528173<br>C 3.9429922 -3.4437154 -0.8394573<br>C 4.1302012 -1.1050654 -1.5056973<br>C 1.9534122 -2.0861754 -1.1141273<br>C 2.5548322 -3.3018654 -0.8164373<br>C 4.7096822 -2.3335954 -1.1837373<br>C 6.2062022 -2.4373844 -1.0966673<br>C 1.7119422 -4.4524154 -0.3463973<br>C 0.7273312 1.0815136 1.4892127

	C	1.3150622	-1.5206364	2.3482027
	C	2.0618812	0.6840746	1.6401727
	C	0.0333412	-1.0773764	2.2211727
	C	2.3939612	-0.6299454	2.0293927
	C	4.7724212	-0.0856754	1.8998227
	C	4.0319622	-2.3882554	2.5460227
	F	6.6350622	-2.2313044	0.1834227
	F	6.8396022	-1.5309744	-1.8664173
	F	6.6547122	-3.6623544	-1.4514573
	F	1.6949022	-4.5306254	1.0171327
	F	0.4255722	-4.3517864	-0.7394173
	F	2.1842232	-5.6401154	-0.7840873
	H	3.9391022	-2.4855554	3.6371227
	H	0.4161112	1.8555836	-2.0088473
	H	1.0073112	0.0585236	-1.6204173
	H	-0.6993098	2.3640236	0.8871927
	H	-0.0912298	3.7459236	-1.1630173
	H	-0.4017898	4.8404136	1.1392427
	H	1.8280792	6.9477346	-1.1099373
	H	2.5034192	5.8239446	1.0065427
	H	1.5670202	4.8993536	-2.5601073
	H	2.1608802	3.2140346	0.8037227
	H	4.4104422	-4.3906054	-0.5956573
	H	4.7506712	-0.2627654	-1.7706673
	H	0.8740122	-1.9882164	-1.0629173
	H	1.4846722	-2.5371264	2.6682627
	H	2.8354612	1.3811546	1.3641127
	H	-0.8337078	-1.6875464	2.4349627
	H	5.7228812	-0.6171744	1.9069127
	H	5.0624522	-2.5998554	2.2593827
	H	0.8289202	4.5706436	2.3709727
	H	0.2055192	6.2726736	-0.9755373
	H	1.0820492	6.8273236	1.2907027
	H	2.8049302	4.6704646	-1.3289773
	H	4.7811412	0.6670546	2.7004227
	H	3.4034422	-3.1408854	2.0676827
	H	4.6822712	0.4254846	0.9368827
	N	-1.5250188	0.2222136	-1.9876873
	C	-2.7746388	-0.3081374	-2.1115473
	C	-3.2852388	-1.1504374	-1.1174273
	C	-4.4394078	-2.0327474	-1.3507973
	C	-6.6018378	-3.7892984	-1.7315173
	C	-5.4564978	-1.7266684	-2.2725773
	C	-4.5320378	-3.2288174	-0.6188873
	C	-5.5998778	-4.1029684	-0.8113473
	C	-6.5267878	-2.5969784	-2.4589673
	O	-1.1306488	1.0899636	-2.8276973
	O	-0.7427088	-0.1527564	-1.0513473
	H	-2.5513678	-1.5107974	-0.4046273
	H	-3.3340188	0.0781426	-2.9493773

	H	-5.4179888	-0.7971084	-2.8329073
	H	-7.3067478	-2.3454284	-3.1725173
	H	-7.4379778	-4.4670384	-1.8808073
	H	-5.6502768	-5.0268384	-0.2415073
	H	-3.7531178	-3.4725074	0.0988827
	C	-3.4672488	1.3710526	0.2941327
	C	-4.2227388	0.1592826	0.4756027
	C	-4.0004088	-0.6850874	1.6297227
	C	-4.9301778	-2.4315274	2.9243827
	C	-3.1820898	3.2360826	-1.1456473
	O	-2.9589088	-0.8039474	2.2746827
	O	-5.0930178	-1.4491074	1.8941627
	O	-2.4605688	1.7325826	0.9151927
	O	-3.9602788	2.1020726	-0.7325973
	H	-3.8006298	3.7645026	-1.8732973
	H	-2.2561898	2.9021526	-1.6256873
	H	-2.9560098	3.8910026	-0.2994373
	H	-5.8676878	-2.9898384	2.9480727
	H	-4.7488478	-1.9566274	3.8938727
	H	-4.0967878	-3.1051674	2.7007627
	H	-5.2186788	0.1558126	0.0533127
	H	-1.2438988	0.4317836	1.6762827
	S	3.9146712	2.1302646	-1.2098673

Name	*(R)*-**TS$_{2O}$** / *(R)*-**TS$_{2O\text{-}BMA}$**
**Absolute Energies (Hartree)**	There are 20 positive frequencies below 100 cm^-1.<br><br>Zero-point correction: 0.745391 hartree<br>Enthalpy correction: 0.797764 hartree<br>Free Energy correction: 0.659838 hartree<br>Quasiharmonic Free Energy correction: 0.673552 hartree<br><br>SCF Energy: -2812.095922 hartree<br>SCF Energy*: -2812.913986 hartree<br>SCF Energy + ZPVE: -2811.350531 hartree<br>Enthalpy: -2811.298158 hartree<br>Free Energy: -2811.436084 hartree<br><br>Free Energy with quasiharmonic correction: -2811.422370 hartree (correction: 8.61 kcal/mol)<br><br>---<br>Low frequencies before projection:<br>-198.1273<br>11.8346<br>20.8518<br><br>Low frequencies after projection:<br>-198.1273<br>10.9111<br>20.817<br>---
**Cartesian Coordinates**	N 0.9487668 1.9923156 -1.6263015<br>N 2.1771168 0.0692156 -1.7493315<br>N -0.0147832 2.2552956 1.0866185<br>N -0.3074832 0.0263056 1.6828285<br>N 3.7738968 -0.6129354 2.0093585<br>C 0.5833268 3.2993156 -1.0921215<br>C 0.1407868 4.7048656 0.9688285<br>C 0.7936568 5.8139056 -1.2199215<br>C 0.8631468 5.8903056 0.3123685<br>C 1.3342068 4.4737356 -1.7391215<br>C 0.6932468 3.3664856 0.4571885<br>C 2.1882968 1.4355956 -1.4702415<br>C 3.1849968 -0.8402154 -1.4416715<br>C 5.1021868 -2.8075754 -0.7828315<br>C 4.5489568 -0.5062254 -1.3722815<br>C 2.7974968 -2.1684854 -1.1906015<br>C 3.7495868 -3.1308854 -0.8704215<br>C 5.4785468 -1.4874754 -1.0336615<br>C 6.9231568 -1.1229454 -0.8440015<br>C 3.2823868 -4.5064954 -0.4913415<br>C 0.5428068 1.0724956 1.4414385

	C	1.4901568	-1.4713844	2.1355285
	C	1.9153868	0.8575356	1.5988685
	C	0.1585668	-1.2073544	2.0059885
	C	2.4322068	-0.4104244	1.9204785
	C	4.6852568	0.5216446	1.8773485
	C	4.3187868	-1.8685454	2.5181185
	O	3.1938068	2.0596346	-1.1159615
	F	7.7544468	-2.0219054	-1.4183015
	F	7.2521668	-1.1038254	0.4829885
	F	7.2321168	0.0920546	-1.3370615
	F	4.2427968	-5.4414254	-0.6544515
	F	2.9133768	-4.5584054	0.8235685
	F	2.2033168	-4.8993844	-1.2007015
	H	4.1637368	-1.9731654	3.6015185
	H	0.1540168	1.3622056	-1.7506515
	H	1.2413468	-0.3483144	-1.7621615
	H	-1.0249632	2.2321456	0.9411485
	H	-0.4810232	3.4065156	-1.3380715
	H	-0.9353232	4.7539156	0.7420885
	H	1.3561268	6.6426456	-1.6669615
	H	1.9167768	5.8858356	0.6265785
	H	1.2268668	4.4038156	-2.8279415
	H	1.7514568	3.3055356	0.7160085
	H	5.8396768	-3.5579054	-0.5233715
	H	4.8603468	0.5108646	-1.5584415
	H	1.7470368	-2.4366544	-1.2253315
	H	1.8036368	-2.4786544	2.3688185
	H	2.5801068	1.6810146	1.4068785
	H	-0.6133932	-1.9517244	2.1471585
	H	5.7083468	0.1522846	1.8341085
	H	5.3893468	-1.8917954	2.3113985
	H	0.2362768	4.7407056	2.0601785
	H	-0.2521732	5.9342856	-1.5403415
	H	0.4315768	6.8332756	0.6698085
	H	2.4010168	4.3778056	-1.5134215
	H	4.5929168	1.2160046	2.7248685
	H	3.8734268	-2.7256154	2.0117685
	H	4.4886868	1.0655546	0.9491085
	N	-1.4554632	-0.6922344	-1.3627915
	C	-2.5179932	-1.5206544	-1.1314915
	C	-3.8349932	-1.0771044	-1.2614115
	C	-4.9591232	-2.0242444	-1.3754015
	C	-7.1595132	-3.7584044	-1.6431415
	C	-4.9057732	-3.3350344	-0.8699115
	C	-6.1399332	-1.5979144	-2.0080415
	C	-7.2284832	-2.4561144	-2.1443615
	C	-5.9947732	-4.1925844	-1.0049015
	O	-0.2889132	-1.1738144	-1.2137215
	O	-1.6146232	0.5076756	-1.7363315
	H	-2.2272332	-2.5059144	-0.8042915

	H	-3.9672232	-0.1280144	-1.7695115
	H	-4.0141532	-3.6860044	-0.3601315
	H	-6.1976032	-0.5821444	-2.3919815
	H	-8.1302232	-2.1092144	-2.6417115
	H	-5.9354332	-5.2025344	-0.6080415
	H	-8.0077332	-4.4296444	-1.7468115
	C	-4.4717932	-0.0706744	0.6536985
	C	-3.7490132	1.1667756	0.5257185
	C	-3.6268832	3.2044556	-0.6579815
	C	-4.0269732	-1.0775544	1.5871085
	C	-4.6594632	-3.0523844	2.7219585
	O	-2.6787232	1.4602556	1.0700485
	O	-4.3713032	2.0276556	-0.3177215
	O	-2.8765832	-1.2543844	1.9891685
	O	-5.0353832	-1.9290744	1.9173685
	H	-5.5264032	-0.0425544	0.4144885
	H	-4.2671932	3.7727056	-1.3352415
	H	-3.3997732	3.8016056	0.2308885
	H	-2.6982232	2.9245556	-1.1643815
	H	-4.3097532	-2.7294344	3.7080685
	H	-5.5626232	-3.6569444	2.8229985
	H	-3.8676332	-3.6366544	2.2426185
	H	-1.3256332	0.1501556	1.5892485

Name	*(S)*-**TS2O** / *(S)*-**TS2O-BMA**
**Absolute Energies (Hartree)**	There are 19 positive frequencies below 100 cm^-1.<br><br>Zero-point correction: 0.745516 hartree<br>Enthalpy correction: 0.797789 hartree<br>Free Energy correction: 0.660620 hartree<br>Quasiharmonic Free Energy correction: 0.673807 hartree<br><br>SCF Energy: -2812.093162 hartree<br>SCF Energy*: -2812.909777 hartree<br>SCF Energy + ZPVE: -2811.347646 hartree<br>Enthalpy: -2811.295373 hartree<br>Free Energy: -2811.432542 hartree<br><br>Free Energy with quasiharmonic correction: -2811.419354 hartree (correction: 8.28 kcal/mol)<br><br>--<br>Low frequencies before projection:<br>-215.7466<br>15.5374<br>18.0781<br><br>Low frequencies after projection:<br>-215.7465<br>15.3767<br>17.8544<br>--
**Cartesian Coordinates**	N 1.0901971 2.2537324 -1.6951252<br>N 1.8985671 0.1207024 -1.7617152<br>N 0.2220771 2.3079924 1.0679248<br>N -0.2668429 0.1561224 1.7848848<br>N 3.7295761 -0.8983086 2.0100548<br>C 0.8867781 3.5228224 -1.0078352<br>C 0.6622481 4.7285224 1.2107748<br>C 1.4281491 5.9919124 -0.8543052<br>C 1.5239991 5.8810724 0.6742648<br>C 1.7754881 4.6609124 -1.5358752<br>C 1.0330481 3.3987624 0.5354648<br>C 2.1824071 1.4614324 -1.4883152<br>C 2.7236561 -0.9591586 -1.4654952<br>C 4.2575351 -3.2433486 -0.8370452<br>C 4.1274161 -0.8968886 -1.4847352<br>C 2.1001061 -2.1750476 -1.1352552<br>C 2.8641251 -3.2956086 -0.8271452<br>C 4.8685461 -2.0327486 -1.1646152<br>C 6.3670461 -1.9574896 -1.0918352<br>C 2.1803151 -4.5473976 -0.3587352<br>C 0.6761171 1.0998124 1.4784648

	C	1.3783261	-1.4903876	2.2864248
	C	2.0243571	0.7550724	1.6226148
	C	0.0780461	-1.0974276	2.1718648
	C	2.4161261	-0.5484086	1.9793248
	C	4.7547371	0.1164314	1.7754148
	C	4.1557761	-2.1990586	2.5183448
	O	3.2753471	1.8685114	-1.0805152
	F	6.7979461	-1.9516996	0.2052848
	F	6.8686161	-0.8491296	-1.6705952
	F	6.9575761	-3.0264896	-1.6729652
	F	2.0642251	-4.5692076	1.0032148
	F	0.9292051	-4.6715976	-0.8478952
	F	2.8654751	-5.6618686	-0.6953252
	H	4.0665661	-2.2620786	3.6122648
	H	0.2438671	1.7929624	-2.0353852
	H	0.8981771	-0.0985876	-1.7178052
	H	-0.7800519	2.3604334	0.8908848
	H	-0.1604719	3.7847724	-1.2116352
	H	-0.4016119	4.9391824	1.0242348
	H	2.0916191	6.7852124	-1.2198852
	H	2.5716691	5.7059714	0.9585048
	H	1.6467281	4.7319224	-2.6221552
	H	2.0817281	3.1870224	0.7448048
	H	4.8490851	-4.1182886	-0.5935852
	H	4.6196971	0.0326414	-1.7297352
	H	1.0164861	-2.2251776	-1.1008652
	H	1.5919561	-2.5080876	2.5764848
	H	2.7657571	1.4890414	1.3624448
	H	-0.7615039	-1.7482266	2.3757148
	H	5.7252871	-0.3701896	1.6950448
	H	5.1979261	-2.3576196	2.2400048
	H	0.7787381	4.6238924	2.2957648
	H	0.4045291	6.2855324	-1.1313452
	H	1.2223091	6.8235124	1.1475848
	H	2.8226481	4.3953114	-1.3525452
	H	4.7916671	0.8502514	2.5932948
	H	3.5755561	-3.0056286	2.0679248
	H	4.5653471	0.6426314	0.8352948
	N	-1.6605729	0.0894634	-1.9739652
	C	-2.9278229	-0.4029266	-2.0581352
	C	-3.4343339	-1.2343756	-1.0469852
	C	-4.6091739	-2.0982756	-1.2692952
	C	-6.8107549	-3.8109646	-1.6306252
	C	-5.6399139	-1.7605156	-2.1638452
	C	-4.7084149	-3.3028956	-0.5535452
	C	-5.7957749	-4.1552756	-0.7361552
	C	-6.7293239	-2.6092046	-2.3413052
	O	-1.2778429	0.9591134	-2.8206452
	O	-0.8525329	-0.3208466	-1.0764752
	H	-2.6801439	-1.6317966	-0.3756052

	H	-3.5072029	0.0131444	-2.8674352
	H	-5.5963839	-0.8232756	-2.7109452
	H	-7.5188739	-2.3330746	-3.0350552
	H	-7.6614949	-4.4719146	-1.7727052
	H	-5.8508449	-5.0864656	-0.1786652
	H	-3.9201949	-3.5705156	0.1452548
	C	-3.5460729	1.2678044	0.3396348
	C	-4.2797129	0.0426444	0.5481048
	C	-4.0040739	-0.7892356	1.7040148
	C	-4.8667039	-2.5338456	3.0461748
	C	-3.3230719	3.1247344	-1.1162952
	O	-2.9356239	-0.8968666	2.3037648
	O	-5.0794039	-1.5569556	2.0192548
	O	-2.5164629	1.6339434	0.9159448
	O	-4.0880529	1.9961144	-0.6615552
	H	-3.9643919	3.6406644	-1.8329552
	H	-2.4115319	2.7824634	-1.6174852
	H	-3.0730219	3.7930334	-0.2873752
	H	-5.7994739	-3.0964556	3.1133048
	H	-4.6454639	-2.0531356	4.0043648
	H	-4.0401249	-3.2038556	2.7889448
	H	-5.3000029	0.0441544	0.1876448
	H	-1.2652229	0.3664434	1.6563448

Name	*(R)*-**TS2S-BMB**
**Absolute Energies (Hartree)**	There are 20 positive frequencies below 100 cm^-1.<br><br>Zero-point correction: 0.741929 hartree<br>Enthalpy correction: 0.794993 hartree<br>Free Energy correction: 0.653484 hartree<br>Quasiharmonic Free Energy correction: 0.669248 hartree<br><br>SCF Energy: -3135.030363 hartree<br>SCF Energy*: -3135.849405 hartree<br>SCF Energy + ZPVE: -3134.288434 hartree<br>Enthalpy: -3134.235370 hartree<br>Free Energy: -3134.376879 hartree<br><br>Free Energy with quasiharmonic correction: -3134.361115 hartree (correction: 9.89 kcal/mol)<br><br>--<br>Low frequencies before projection:<br>-196.4118<br>7.4345<br>11.9580<br><br>Low frequencies after projection:<br>-196.4118<br>6.862<br>11.6076<br>--
**Cartesian Coordinates**	C 2.8108154 2.9911164 -0.3188191<br>C 2.8876354 1.9540164 0.6210509<br>C 4.1408354 1.4045074 0.9374309<br>C 5.2915254 1.8968274 0.3271009<br>C 5.2284454 2.9475374 -0.5893391<br>C 3.9793754 3.4828074 -0.9003691<br>N 1.7598054 1.3514164 1.1873109<br>C 0.5616054 1.9157864 1.5499309<br>C 6.6372654 1.3179874 0.6574409<br>C 3.8870644 4.6336674 -1.8654291<br>N -0.3738046 0.9722664 1.7930509<br>C -1.6858946 1.1916664 2.3834709<br>C -2.7521146 0.3813764 1.6102009<br>C -4.1302046 0.5715954 2.2561809<br>C -4.1039646 0.1741754 3.7401409<br>C -3.0530646 0.9763454 4.5148809<br>C -1.6713246 0.8108864 3.8740209<br>N -2.6574846 0.7549764 0.1817409<br>O -0.1454546 0.8593064 -1.4230391<br>N 0.2169754 -0.3298536 -1.6562191<br>O -0.6388846 -1.1825936 -2.0765491

	C	1.5256254	-0.6772936	-1.4872791
	C	1.9550754	-1.9964536	-1.6326791
	C	3.3790554	-2.3420936	-1.7724691
	C	4.4117554	-1.4447926	-1.4472491
	C	5.7471654	-1.8206526	-1.5752391
	C	6.0766864	-3.0995626	-2.0309191
	C	5.0603964	-4.0000026	-2.3624691
	C	3.7262964	-3.6246236	-2.2316791
	C	1.5427964	-3.0696336	0.3458609
	C	0.1526764	-2.7290036	0.4964709
	O	-0.3227546	-1.7058536	0.9948109
	C	2.5661964	-2.4434436	1.1472309
	O	2.5459454	-1.3209136	1.6611709
	H	-1.9168746	2.2548264	2.2754309
	H	-1.3381346	-0.2326136	3.9667609
	H	-0.9248546	1.4326764	4.3800209
	H	-3.0238446	0.6589054	5.5642909
	H	-3.3310846	2.0399954	4.5120309
	H	-3.8781546	-0.8987346	3.8229509
	H	-5.1013746	0.3213454	4.1721109
	H	-4.4334046	1.6237054	2.1657709
	H	-4.8746346	-0.0359246	1.7320209
	H	-2.4839146	-0.6827236	1.6863509
	H	1.8690354	0.3454164	1.3619009
	H	-0.1241646	-0.0057636	1.6179109
	H	1.7530164	-4.0788336	0.0152409
	H	-1.6933946	0.7148564	-0.1576391
	H	2.1448254	0.1412764	-1.1617591
	H	1.2496664	-2.6807036	-2.0905191
	H	2.9369464	-4.3308236	-2.4777191
	H	5.3083964	-4.9958226	-2.7198791
	H	7.1187564	-3.3917726	-2.1289091
	H	6.5274154	-1.1122126	-1.3154891
	H	4.1781754	-0.4493826	-1.0871191
	H	4.1947354	0.5802074	1.6385509
	H	6.1272454	3.3244874	-1.0636391
	H	1.8468654	3.3863164	-0.6065191
	F	6.5535954	0.1753474	1.3781209
	F	7.4025154	2.1781574	1.3667009
	F	7.3408454	1.0234974	-0.4690491
	F	4.9044844	4.6257674	-2.7599791
	F	2.7333444	4.6161664	-2.5690191
	F	3.9397444	5.8278574	-1.2286491
	C	-2.0421736	-3.3596136	-0.0960491
	H	-2.1955936	-2.4502036	-0.6832591
	H	-2.4560936	-3.2213636	0.9085809
	C	4.8006064	-2.6731726	1.9034409
	H	4.5591654	-2.4015126	2.9358609
	H	5.1663354	-1.7927426	1.3726609
	O	-0.6501836	-3.6857536	-0.0422491

	O	3.6614364	-3.2381136	1.2398409
	H	-2.5250236	-4.2104936	-0.5817891
	H	5.5620064	-3.4552726	1.8880109
	C	-3.5764446	0.5930254	-0.8072491
	C	-5.2089246	0.2823754	-3.0726391
	C	-4.9280046	0.9535754	-0.7492191
	N	-3.1012946	0.0891854	-1.9914691
	C	-3.8827046	-0.0358746	-3.0903191
	C	-5.7829746	0.7845454	-1.8587891
	H	-5.2839746	1.4308154	0.1470409
	H	-3.3826646	-0.4183946	-3.9728091
	H	-5.7891846	0.1622354	-3.9759991
	N	-7.0991746	1.1151454	-1.7940591
	C	-7.6551046	1.6616554	-0.5604391
	H	-7.2046446	2.6308554	-0.3089191
	H	-7.4960946	0.9742054	0.2792809
	H	-8.7288246	1.8053754	-0.6824291
	C	-7.9497846	1.0095354	-2.9767891
	H	-7.6058646	1.6701354	-3.7827591
	H	-8.9663046	1.2993154	-2.7107391
	H	-7.9809046	-0.0197146	-3.3536491
	H	-2.1224246	-0.2618036	-2.0394991
	S	0.3029754	3.5850564	1.7263809

Name	*(R)*-**TS$_{2O-BMB}$**
**Absolute Energies (Hartree)**	There are 22 positive frequencies below 100 cm^-1.<br><br>Zero-point correction: 0.744299 hartree<br>Enthalpy correction: 0.797047 hartree<br>Free Energy correction: 0.656641 hartree<br>Quasiharmonic Free Energy correction: 0.671778 hartree<br><br>SCF Energy: -2812.076292 hartree<br>SCF Energy*: -2812.896011 hartree<br>SCF Energy + ZPVE: -2811.331993 hartree<br>Enthalpy: -2811.279245 hartree<br>Free Energy: -2811.419651 hartree<br><br>Free Energy with quasiharmonic correction: -2811.404515 hartree (correction: 9.50 kcal/mol)<br><br>---<br>Low frequencies before projection:<br>-198.5665<br>9.4283<br>12.9013<br><br>Low frequencies after projection:<br>-198.5663<br>9.3259<br>12.5613<br>---
**Cartesian Coordinates**	C 2.8628920 3.4934266 -0.0301821<br>C 2.8775230 2.1653466 0.4355379<br>C 4.0673430 1.4207176 0.3130879<br>C 5.1956430 1.9866176 -0.2713521<br>C 5.1901820 3.3013476 -0.7378821<br>C 4.0121320 4.0355876 -0.6026721<br>N 1.7853330 1.5213466 0.9946379<br>C 0.5373730 2.0625266 1.2891079<br>C 6.4158030 1.1380676 -0.4787621<br>C 3.9958120 5.4783276 -1.0289621<br>N -0.3478870 1.1023066 1.6904879<br>C -1.6654170 1.4232866 2.2074679<br>C -2.7465770 0.5650856 1.5084979<br>C -4.1352770 0.8877156 2.0732479<br>C -4.1723470 0.6719656 3.5939579<br>C -3.1108670 1.5200256 4.3028679<br>C -1.7186770 1.2265366 3.7336479<br>N -2.5911570 0.7393566 0.0479779<br>O -0.0417170 0.5063566 -1.4944821<br>N 0.2596830 -0.7179634 -1.5648921<br>O -0.6435270 -1.5783134 -1.8556821

	C	1.5540330	-1.1065834	-1.3678721
	C	1.8967530	-2.4581934	-1.3130821
	C	3.2813930	-2.9397734	-1.4467021
	C	4.3959230	-2.0840524	-1.4114721
	C	5.6880030	-2.5974924	-1.5131621
	C	5.8910340	-3.9715224	-1.6558221
	C	4.7909740	-4.8326624	-1.7073021
	C	3.5018540	-4.3203624	-1.6032121
	C	1.5185030	-3.1341934	0.8378779
	C	0.1307730	-2.7616434	0.9363179
	O	-0.3367370	-1.6710534	1.2675579
	C	2.5619030	-2.3528034	1.4529579
	O	2.5489930	-1.1449634	1.7077679
	H	-1.8518880	2.4738066	1.9641479
	H	-1.4329870	0.1897166	3.9622179
	H	-0.9617870	1.8729166	4.1918279
	H	-3.1271470	1.3313256	5.3833179
	H	-3.3447180	2.5854456	4.1632779
	H	-3.9957570	-0.3910544	3.8124079
	H	-5.1748670	0.9096656	3.9704379
	H	-4.3875270	1.9335356	1.8486279
	H	-4.8907970	0.2539456	1.5979179
	H	-2.5242970	-0.4892134	1.7317579
	H	1.9200530	0.5298566	1.2031279
	H	-0.0728470	0.1197266	1.6835779
	H	1.7233340	-4.1888634	0.7074779
	H	-1.6218070	0.5924366	-0.2435121
	H	2.2212230	-0.2858734	-1.1592121
	H	1.1238230	-3.1529734	-1.6218021
	H	2.6472940	-4.9923334	-1.6286921
	H	4.9394040	-5.9026524	-1.8260121
	H	6.8996640	-4.3686524	-1.7331121
	H	6.5323130	-1.9174524	-1.4817621
	H	4.2642130	-1.0120624	-1.3193021
	H	4.0927330	0.4040476	0.6897579
	H	6.0719820	3.7371676	-1.1911721
	H	1.9587320	4.0773466	0.0607379
	F	6.3776530	0.4997676	-1.6826121
	F	6.5410930	0.1659576	0.4583379
	F	7.5572130	1.8590076	-0.4643521
	F	4.8631620	5.7183176	-2.0417521
	F	2.7734720	5.8772366	-1.4450521
	F	4.3503020	6.3049676	-0.0134121
	C	-2.0672870	-3.4580034	0.4380879
	H	-2.2078570	-2.6490534	-0.2844421
	H	-2.4845670	-3.1604634	1.4061179
	C	4.8270530	-2.4064124	2.1508079
	H	4.6144530	-1.8986224	3.0966079
	H	5.1800030	-1.6750424	1.4199279
	O	-0.6792160	-3.7863334	0.5502579

	O	3.6709830	-3.1053224	1.6695579
	H	-2.5576460	-4.3690034	0.0875279
	H	5.5891330	-3.1742424	2.2970179
	C	-3.4919770	0.4990556	-0.9410421
	C	-5.0849470	-0.0151744	-3.1970421
	C	-4.8156270	0.9543956	-0.9807721
	N	-3.0236570	-0.1950944	-2.0271021
	C	-3.7849770	-0.4224444	-3.1233121
	C	-5.6510570	0.6932656	-2.0866221
	H	-5.1579770	1.5748756	-0.1711521
	H	-3.2932970	-0.9615044	-3.9250121
	H	-5.6517670	-0.2291444	-4.0917721
	N	-6.9397770	1.1241156	-2.1177221
	C	-7.4947870	1.8570456	-0.9846121
	H	-7.0056880	2.8313356	-0.8514621
	H	-7.3872070	1.2830156	-0.0566521
	H	-8.5582070	2.0273156	-1.1541521
	C	-7.7483870	0.9553456	-3.3222821
	H	-7.2972270	1.4617856	-4.1848921
	H	-8.7339070	1.3894146	-3.1531621
	H	-7.8832570	-0.1050644	-3.5674521
	H	-2.0682270	-0.6107234	-1.9908921
	O	0.2659920	3.2612466	1.2089379

Name	*(S)*-TS$_{2O\text{-}BMB}$
**Absolute Energies (Hartree)**	There are 22 positive frequencies below 100 cm^-1.<br><br>Zero-point correction: 0.745312 hartree<br>Enthalpy correction: 0.797789 hartree<br>Free Energy correction: 0.659676 hartree<br>Quasiharmonic Free Energy correction: 0.673312 hartree<br><br>SCF Energy: -2812.071925 hartree<br>SCF Energy*: -2812.901185 hartree<br>SCF Energy + ZPVE: -2811.326613 hartree<br>Enthalpy: -2811.274136 hartree<br>Free Energy: -2811.412249 hartree<br><br>Free Energy with quasiharmonic correction: -2811.398613 hartree (correction: 8.56 kcal/mol)<br><br>---<br>Low frequencies before projection:<br>-192.2984<br>13.2430<br>20.0062<br><br>Low frequencies after projection:<br>-192.2982<br>12.5053<br>19.8391<br>---
**Cartesian Coordinates**	C -3.5692931 -1.0127897 1.5334885<br>C -3.5188131 -2.0334397 0.5262785<br>O -2.6266931 -0.3687807 2.0068685<br>C -2.3189531 -2.7956807 0.2592985<br>O -1.2119531 -2.7069807 0.7867685<br>C -3.6572431 -0.7225497 -1.3564515<br>C -2.7579821 0.3431293 -1.3094615<br>C -5.1103131 -0.4911797 -1.3441815<br>N -1.4328131 0.1620093 -1.5878215<br>O -0.9507231 -0.9898807 -1.8013915<br>O -0.6915821 1.1947393 -1.6442215<br>C -5.9556731 -1.4749097 -1.8882615<br>C -5.6926321 0.6677603 -0.7995915<br>C -7.0748621 0.8377903 -0.8127915<br>C -7.3373631 -1.3033397 -1.9011715<br>C -7.9027031 -0.1444197 -1.3624515<br>O -4.8528231 -0.7236597 1.8789485<br>O -2.5588331 -3.7085207 -0.7276715<br>C -1.4164831 -4.4327507 -1.1946515<br>C -5.0349021 0.3980203 2.7519985

	H	-4.6915921	1.3277403	2.2874285
	H	-4.4999421	0.2549603	3.6958685
	H	-6.1101221	0.4534203	2.9327385
	H	-4.4517531	-2.5240497	0.2852585
	H	-3.0142621	1.3673093	-1.0902015
	H	-5.0710121	1.4428703	-0.3652815
	H	-7.5071521	1.7403303	-0.3891215
	H	-8.9808431	-0.0084597	-1.3692915
	H	-7.9732531	-2.0729897	-2.3303915
	H	-5.5166031	-2.3812397	-2.2984515
	H	-3.3182431	-1.6155797	-1.8690315
	H	-0.9234131	-4.9699807	-0.3783215
	H	-0.7022331	-3.7526807	-1.6692015
	H	-1.7978931	-5.1431307	-1.9314315
	N	0.9678969	-1.2880307	1.8870785
	N	-0.0555921	0.7434193	1.6159385
	N	1.8239369	-1.4062907	-0.8796115
	N	2.0043779	0.7695593	-1.5958315
	N	6.0501379	1.0103183	-0.8011515
	C	1.9644569	-2.2943607	1.5270785
	C	3.0368469	-3.6394107	-0.3943915
	C	4.2382169	-3.3080417	1.8392785
	C	4.3926969	-3.5613817	0.3318885
	C	3.3604569	-2.0807007	2.1272085
	C	1.9585469	-2.5919907	-0.0108815
	C	1.1524169	0.0596393	1.6990485
	C	-0.2492921	2.0674993	1.2437485
	C	-0.8635621	4.6703293	0.3136485
	C	0.7780979	2.9436993	0.8497085
	C	-1.5757121	2.5288793	1.2099285
	C	-1.8691321	3.8034993	0.7402285
	C	0.4553479	4.2180193	0.3873885
	C	1.5509779	5.0984793	-0.1423715
	C	-3.3142021	4.1835203	0.6112685
	C	2.6307369	-0.3406607	-1.0963715
	C	3.9954679	2.0670883	-1.5542615
	C	4.0060669	-0.2677817	-0.8699115
	C	2.6623679	1.9355893	-1.8035415
	C	4.7181879	0.9291683	-1.0661715
	C	6.7537679	2.2830883	-0.9287215
	C	6.7502069	-0.1403717	-0.2425915
	O	2.2500579	0.6165893	1.6257185
	F	1.6454979	5.0139293	-1.5029215
	F	2.7703379	4.7710493	0.3402085
	F	1.3410079	6.4047893	0.1362085
	F	-4.0567621	3.7506503	1.6614885
	F	-3.5018321	5.5141803	0.5050585
	F	-3.8804221	3.6200703	-0.4975115
	H	1.8043479	2.6131693	0.8954185
	H	-1.0970921	5.6615693	-0.0556915

	H	6.3490279	3.0405083	-0.2451015
	H	7.8062479	2.1332783	-0.6863515
	H	6.6964179	2.6683783	-1.9536515
	H	6.6869369	-1.0048317	-0.9154815
	H	7.8038169	0.1079083	-0.1122115
	H	6.3392569	-0.4276117	0.7345385
	H	4.4628779	3.0290183	-1.7064215
	H	2.0494879	2.7586293	-2.1476015
	H	4.4934169	-1.1417717	-0.4832215
	H	0.9955269	-3.0731107	-0.1858515
	H	3.8124569	-1.1712217	1.7304985
	H	5.0294569	-2.7832817	-0.1031615
	H	4.9310969	-4.5028017	0.1631685
	H	5.2263369	-3.1808617	2.2991985
	H	3.7869169	-4.1942117	2.3101185
	H	3.1740669	-3.6287407	-1.4818415
	H	1.5860269	-3.2237707	1.9737385
	H	3.2522869	-1.9322307	3.2082585
	H	2.5978169	-4.6161107	-0.1501515
	H	-2.3705621	1.8652993	1.5293885
	H	0.8525069	-1.2495707	-1.1571715
	H	0.9680779	0.7866793	-1.6915015
	H	0.0186969	-1.6285607	1.7291185
	H	-0.9161031	0.1971293	1.7119885

Name	Complex of ***(R)*-TS$_{2S}$**
**Absolute Energies (Hartree)**	There are 20 positive frequencies below 100 cm^-1.<br><br>Zero-point correction: 0.745321 hartree<br>Enthalpy correction: 0.798281 hartree<br>Free Energy correction: 0.661102 hartree<br>Quasiharmonic Free Energy correction: 0.672538 hartree<br><br>SCF Energy: -3135.045960 hartree<br>SCF Energy*: -3135.865413 hartree<br>SCF Energy + ZPVE: -3134.300639 hartree<br>Enthalpy: -3134.247679 hartree<br>Free Energy: -3134.384858 hartree<br><br>Free Energy with quasiharmonic correction: -3134.373423 hartree (correction: 7.18 kcal/mol)<br><br>--<br>Low frequencies before projection:<br>18.2564<br>22.7370<br>32.1389<br>Low frequencies after projection:<br>18.1434<br>22.5466<br>31.5624<br>--

Cartesian Coordinates

N	1.0519895	2.0873316	-1.5822617
N	2.3192995	0.2274916	-1.7151117
N	0.0380395	2.1922826	1.1264983
N	-0.2121505	-0.0455874	1.6650883
N	3.8720295	-0.6515484	2.0393783
C	0.5289495	3.3369826	-1.0155317
C	0.0205895	4.6334826	1.1091183
C	0.3223905	5.8496426	-1.0882817
C	0.5329305	5.9101226	0.4307383
C	1.0285095	4.6257716	-1.6870617
C	0.6751395	3.3659316	0.5341283
C	2.3132295	1.5916416	-1.5122917
C	3.2868695	-0.7536684	-1.4559617
C	5.0275695	-2.8890884	-0.8249617
C	4.6739495	-0.5480184	-1.4124817
C	2.7873495	-2.0452684	-1.2062817
C	3.6504395	-3.0908884	-0.9013017
C	5.5159295	-1.6100284	-1.0816317
C	6.9842695	-1.3545384	-0.8879717
C	3.0719285	-4.4250684	-0.5259417

	C	0.6272295	1.0186216	1.4606683
	C	1.5963995	-1.5208784	2.1319583
	C	2.0031295	0.8219516	1.6296383
	C	0.2630995	-1.2738774	1.9853983
	C	2.5298795	-0.4479584	1.9393083
	C	4.7889695	0.4839316	1.9718883
	C	4.4109095	-1.9202984	2.5222983
	F	7.7414095	-2.3928184	-1.3072417
	F	7.2761795	-1.1824184	0.4352083
	F	7.4137795	-0.2494584	-1.5268917
	F	3.9193285	-5.4421484	-0.7882517
	F	2.7973085	-4.4873184	0.8103683
	F	1.9107285	-4.6819684	-1.1640617
	H	4.2630895	-2.0446484	3.6047183
	H	0.3038695	1.4174326	-1.7569117
	H	1.3802295	-0.1746484	-1.7287117
	H	-0.9730005	2.1326326	0.9824983
	H	-0.5467905	3.2672926	-1.2179417
	H	-1.0679405	4.5591626	0.9657883
	H	0.6997605	6.7627316	-1.5640517
	H	1.6044505	6.0326816	0.6432483
	H	0.8451095	4.5507916	-2.7654917
	H	1.7381795	3.3687916	0.7805083
	H	5.6974395	-3.7021684	-0.5713517
	H	5.0831595	0.4307116	-1.6094417
	H	1.7184395	-2.2259784	-1.2282217
	H	1.9165695	-2.5245184	2.3690783
	H	2.6568595	1.6631816	1.4690383
	H	-0.5029205	-2.0286174	2.1039283
	H	5.8119995	0.1122116	1.9333983
	H	5.4797995	-1.9471084	2.3071383
	H	0.1940195	4.6605426	2.1909583
	H	-0.7558895	5.8029026	-1.3038917
	H	0.0233905	6.7831226	0.8565583
	H	2.1078595	4.7187716	-1.5492217
	H	4.6814695	1.1443716	2.8442583
	H	3.9564595	-2.7650484	2.0039583
	H	4.6171695	1.0664016	1.0628083
	N	-1.4143405	-0.5216474	-1.5127817
	C	-2.4767905	-1.4224574	-1.3464917
	C	-3.7623505	-1.0525274	-1.5838417
	C	-4.9130605	-1.9431174	-1.5631717
	C	-7.2034105	-3.5720074	-1.5447017
	C	-4.8221205	-3.3061174	-1.2188417
	C	-6.1749005	-1.4164574	-1.9013017
	C	-7.3086905	-2.2220374	-1.8919217
	C	-5.9568615	-4.1097974	-1.2098417
	O	-0.2696805	-0.9471174	-1.2136717
	O	-1.5867205	0.6246426	-1.9710717
	H	-2.1480705	-2.3793774	-0.9738617

	H	-3.9362905	-0.0324874	-1.9106217
	H	-3.8629905	-3.7389074	-0.9540417
	H	-6.2569505	-0.3626774	-2.1549617
	H	-8.2749605	-1.7987164	-2.1511017
	H	-5.8714115	-5.1583774	-0.9386217
	H	-8.0884315	-4.2022774	-1.5342117
	C	-4.5084805	-0.0425274	0.9762783
	C	-3.7370205	1.0902526	0.6533483
	C	-3.7053805	3.2257226	-0.3666017
	C	-3.9273205	-1.2086174	1.5459483
	C	-4.3845205	-3.3985474	2.3258283
	O	-2.5170905	1.2546326	0.8557483
	O	-4.4546905	2.0726826	0.0205183
	O	-2.7298905	-1.4439974	1.7653083
	O	-4.8784805	-2.1659474	1.8063283
	H	-5.5724205	-0.0266474	0.7872783
	H	-4.4081005	3.8698526	-0.9005417
	H	-3.3127105	3.7582626	0.5075183
	H	-2.8715705	2.9566226	-1.0249817
	H	-3.9237305	-3.2617474	3.3109483
	H	-5.2543215	-4.0542574	2.4110983
	H	-3.6435505	-3.8512174	1.6581183
	H	-1.2313805	0.0593926	1.5152183
	S	3.6883095	2.5342116	-1.2031117

<table>
<tr><td>Name</td><td>Complex of (S)-TS_{2S}</td></tr>
<tr><td>Absolute Energies (Hartree)</td><td>There are 19 positive frequencies below 100 cm^-1.

Zero-point correction: 0.744723 hartree
Enthalpy correction: 0.797872 hartree
Free Energy correction: 0.659246 hartree
Quasiharmonic Free Energy correction: 0.672097 hartree

SCF Energy: -3135.046018 hartree
SCF Energy*: -3135.865413 hartree
SCF Energy + ZPVE: -3134.301295 hartree
Enthalpy: -3134.248146 hartree
Free Energy: -3134.386772 hartree

Free Energy with quasiharmonic correction: -3134.373921 hartree (correction: 8.06 kcal/mol)

Low frequencies before projection:
18.9714
22.2214
28.1256
Low frequencies after projection:
14.6809
21.739
27.8548
---</td></tr>
<tr><td>Cartesian Coordinates</td><td>N 1.0853419 2.2527057 -1.5958168
N 2.1950519 0.2952657 -1.7146768
N 0.0474619 2.1362857 1.1010132
N -0.2598781 -0.0988943 1.5707332
N 3.7825819 -0.8254043 2.0993832
C 0.6027319 3.4620557 -0.9177568
C 0.0933519 4.5734257 1.3041232
C 0.5138119 5.9794357 -0.7583968
C 0.6744019 5.8871557 0.7651232
C 1.1797119 4.7829357 -1.4511868
C 0.7225719 3.3419857 0.6295232
C 2.2938519 1.6509257 -1.4707468
C 3.0863019 -0.7578443 -1.4713868
C 4.6710419 -3.0154843 -0.8541568
C 4.4851519 -0.6544343 -1.4335068
C 2.4960019 -2.0135143 -1.2334768
C 3.2825919 -3.1183143 -0.9301968
C 5.2496219 -1.7743543 -1.1073368
C 6.7331119 -1.6232543 -0.9167968
C 2.6219219 -4.4194043 -0.5718668</td></tr>
</table>

	C	0.6090819	0.9497657	1.4340432
	C	1.4804719	-1.6191043	2.1334332
	C	1.9715119	0.7168057	1.6660032
	C	0.1635719	-1.3359343	1.9226132
	C	2.4504919	-0.5737443	1.9734932
	C	4.7386619	0.2791957	2.0648832
	C	4.2714319	-2.1275543	2.5431232
	F	7.0385019	-1.4591243	0.4039432
	F	7.2393219	-0.5578943	-1.5670168
	F	7.4131119	-2.7169843	-1.3271068
	F	2.4574919	-4.5380043	0.7783332
	F	1.3950319	-4.5455943	-1.1184568
	F	3.3560329	-5.4849443	-0.9575468
	H	4.1305119	-2.2752143	3.6235632
	H	0.3304019	1.6716757	-1.9579468
	H	1.2291519	-0.0348343	-1.7417568
	H	-0.9391481	2.0657857	0.8324732
	H	-0.4718281	3.4620157	-1.1403468
	H	-0.9924181	4.5527757	1.1261032
	H	0.9488519	6.9135257	-1.1336568
	H	1.7421019	5.9452057	1.0205832
	H	1.0256119	4.8182157	-2.5361968
	H	1.7801819	3.2929357	0.8927532
	H	5.2810219	-3.8755743	-0.6037068
	H	4.9643319	0.2925057	-1.6274568
	H	1.4168019	-2.1166143	-1.2596268
	H	1.7606719	-2.6278943	2.3956432
	H	2.6547319	1.5403957	1.5403732
	H	-0.6285481	-2.0668243	2.0180332
	H	5.7499719	-0.1250643	2.0668032
	H	5.3355219	-2.1953243	2.3136132
	H	0.2352919	4.4922057	2.3878832
	H	-0.5574281	6.0060957	-1.0092368
	H	0.1843219	6.7364457	1.2566132
	H	2.2585119	4.8056357	-1.2781168
	H	4.6200219	0.9438357	2.9323132
	H	3.7736019	-2.9363043	2.0075432
	H	4.6181519	0.8663357	1.1500132
	N	-1.4164381	-0.2139943	-2.1395468
	C	-2.7065281	-0.7783443	-2.2791168
	C	-3.1754081	-1.6552143	-1.3637768
	C	-4.4468381	-2.3538143	-1.4108668
	C	-6.8608581	-3.7863543	-1.3847068
	C	-5.3915981	-2.1826743	-2.4440868
	C	-4.7359281	-3.2604043	-0.3720768
	C	-5.9322181	-3.9700043	-0.3582268
	C	-6.5860781	-2.8916243	-2.4266468
	O	-1.1316181	0.7476857	-2.8827468
	O	-0.5912981	-0.6961743	-1.3399268
	H	-2.5383881	-1.8606143	-0.5083368

	H	-3.2397781	-0.3797643	-3.1295268
	H	-5.1945181	-1.4887443	-3.2554368
	H	-7.3092981	-2.7491043	-3.2245868
	H	-7.7979881	-4.3361143	-1.3758268
	H	-6.1427381	-4.6592543	0.4543932
	H	-4.0126681	-3.3904943	0.4263032
	C	-3.6115081	1.1900157	0.1493032
	C	-4.4623381	0.3119657	0.8387732
	C	-3.9877681	-0.7348843	1.6651732
	C	-4.6496281	-2.4442943	3.1734132
	C	-3.4887081	3.0157557	-1.3609168
	O	-2.8162981	-1.0961543	1.8834532
	O	-5.0260581	-1.4217743	2.2546432
	O	-2.3576281	1.2157157	0.1482732
	O	-4.2945681	2.0937557	-0.6275268
	H	-4.1866081	3.6040457	-1.9622768
	H	-2.7763481	2.5010157	-2.0144968
	H	-2.9358681	3.6865857	-0.6915868
	H	-5.5856981	-2.8838843	3.5269832
	H	-4.0899181	-2.0336343	4.0217432
	H	-4.0341081	-3.2183643	2.7017032
	H	-5.5315281	0.4388857	0.7342932
	H	-1.2657381	0.0132957	1.3565832
	S	3.7186519	2.4668557	-1.0513868

**Name**	Product of ***(S)*-TS2S**
**Absolute Energies (Hartree)**	There are 19 positive frequencies below 100 cm^-1.<br><br>Zero-point correction: 0.745323 hartree<br>Enthalpy correction: 0.797989 hartree<br>Free Energy correction: 0.659612 hartree<br>Quasiharmonic Free Energy correction: 0.673443 hartree<br><br>SCF Energy: -3135.058770 hartree<br>SCF Energy*: -3135.875089 hartree<br>SCF Energy + ZPVE: -3134.313447 hartree<br>Enthalpy: -3134.260781 hartree<br>Free Energy: -3134.399158 hartree<br><br>Free Energy with quasiharmonic correction: -3134.385328 hartree (correction: 8.68 kcal/mol)<br><br>---<br>Low frequencies before projection:<br>10.3343<br>20.1594<br>22.6166<br>Low frequencies after projection:<br>9.5568<br>20.1461<br>22.5877<br>---
**Cartesian Coordinates**	N 1.2373660 2.3987142 -1.7696100<br>N 1.9526150 0.2723632 -1.7096500<br>N 0.1992860 2.3471642 0.9476100<br>N -0.3875750 0.2678542 1.7641900<br>N 3.5322640 -0.9655168 2.1904400<br>C 0.9349260 3.6392342 -1.0535700<br>C 0.5676560 4.7570542 1.1984100<br>C 1.2342570 6.1415542 -0.8131900<br>C 1.3478370 5.9844342 0.7091400<br>C 1.7161060 4.8745442 -1.5319400<br>C 1.0211060 3.4671342 0.4917300<br>C 2.3115150 1.5906532 -1.5696000<br>C 2.6240740 -0.9144868 -1.4114700<br>C 3.7209230 -3.4144768 -0.6836500<br>C 4.0131440 -1.1058968 -1.4196500<br>C 1.7913840 -1.9950258 -1.0552300<br>C 2.3378640 -3.2194968 -0.6990700<br>C 4.5371640 -2.3450468 -1.0435600<br>C 6.0257340 -2.5005278 -0.9152200<br>C 1.4419530 -4.3222058 -0.2138000<br>C 0.6071050 1.1545442 1.4411500<br>C 1.1545740 -1.4169258 2.4148400

	C	1.9333350	0.7598832	1.6480600
	C	-0.1199860	-0.9787558	2.2258300
	C	2.2462440	-0.5424368	2.0916200
	C	4.6307550	-0.0224768	1.9877100
	C	3.8551340	-2.3050468	2.6752600
	F	6.4342840	-2.2566378	0.3658900
	F	6.7116940	-1.6511878	-1.7049400
	F	6.4370830	-3.7550178	-1.2075800
	F	1.4546930	-4.4104658	1.1495400
	F	0.1511830	-4.1472458	-0.5731100
	F	1.8319130	-5.5329858	-0.6690400
	H	3.7398340	-2.3872868	3.7653500
	H	0.3902550	1.9296142	-2.1244200
	H	0.9231550	0.1509242	-1.6459500
	H	-0.7853840	2.3993242	0.7034200
	H	-0.1250040	3.8159942	-1.2848900
	H	-0.5062140	4.9036642	1.0056300
	H	1.8173970	7.0062542	-1.1523600
	H	2.4064970	5.8731832	0.9834800
	H	1.5907560	4.9635542	-2.6172900
	H	2.0610060	3.2682932	0.7529500
	H	4.1470330	-4.3689568	-0.3966800
	H	4.6713350	-0.2954078	-1.6940300
	H	0.7167540	-1.8468558	-1.0354600
	H	1.3108740	-2.4207858	2.7777900
	H	2.7181750	1.4389332	1.3581500
	H	-0.9937360	-1.5841248	2.4261300
	H	5.5764640	-0.5584778	2.0508700
	H	4.8883440	-2.5309478	2.4114200
	H	0.6778660	4.6244842	2.2811100
	H	0.1855970	6.3442942	-1.0792500
	H	0.9812270	6.8835042	1.2197600
	H	2.7820760	4.7192232	-1.3442300
	H	4.6209850	0.7691432	2.7495200
	H	3.2276640	-3.0569468	2.1937100
	H	4.5710250	0.4409532	0.9982900
	N	-1.5221750	0.3557252	-2.0136100
	C	-2.7221050	-0.1967748	-2.0515200
	C	-3.2107460	-0.9743948	-0.8706700
	C	-3.8684760	-2.2979338	-1.2188900
	C	-5.0313570	-4.7769938	-1.8572300
	C	-5.0633160	-2.3590538	-1.9493100
	C	-3.2636970	-3.4951848	-0.8146100
	C	-3.8393470	-4.7268938	-1.1329700
	C	-5.6411470	-3.5880738	-2.2666900
	O	-1.1285050	1.1932552	-2.9152500
	O	-0.6911450	0.0745542	-1.0438900
	H	-2.3338260	-1.1718648	-0.2544600
	H	-3.3572950	0.1072752	-2.8708100
	H	-5.5445260	-1.4400738	-2.2768900

	H	-6.5669770	-3.6179828	-2.8354000
	H	-5.4814670	-5.7347138	-2.1048600
	H	-3.3526970	-5.6457748	-0.8162500
	H	-2.3272070	-3.4639648	-0.2631500
	C	-3.5977250	1.3156252	0.0550800
	C	-4.1730550	-0.0872538	0.0389700
	C	-4.2237560	-0.7040338	1.4223300
	C	-5.5969460	-1.7550638	3.0219300
	C	-3.4655940	3.2866152	-1.2382200
	O	-3.2481760	-0.8869148	2.1274800
	O	-5.4658560	-1.0685638	1.7578900
	O	-2.6663350	1.6846952	0.7546000
	O	-4.1835150	2.0865262	-0.8580900
	H	-4.1400340	3.8268662	-1.9027600
	H	-2.5573640	2.9934752	-1.7750400
	H	-3.2263440	3.8927452	-0.3613500
	H	-6.6584560	-1.9841728	3.1187300
	H	-5.2652860	-1.1127838	3.8422500
	H	-5.0050660	-2.6744838	3.0154100
	H	-5.1714450	-0.0516038	-0.3966400
	H	-1.3575750	0.5062452	1.5448700
	S	3.8669450	2.1561332	-1.1715400

Name	Product of ***(R)*-TS$_{2S}$**
**Absolute Energies (Hartree)**	There are 20 positive frequencies below 100 cm^-1.<br><br>Zero-point correction: 0.745407 hartree<br>Enthalpy correction: 0.798054 hartree<br>Free Energy correction: 0.660309 hartree<br>Quasiharmonic Free Energy correction: 0.673460 hartree<br><br>SCF Energy: -3135.061950 hartree<br>SCF Energy*: -3135.880445 hartree<br>SCF Energy + ZPVE: -3134.316543 hartree<br>Enthalpy: -3134.263896 hartree<br>Free Energy: -3134.401641 hartree<br><br>Free Energy with quasiharmonic correction: -3134.388490 hartree (correction: 8.25 kcal/mol)<br><br>--<br>Low frequencies before projection:<br>12.5061<br>20.1960<br>27.4576<br>Low frequencies after projection:<br>12.3478<br>20.1537<br>27.3387<br>--
**Cartesian Coordinates**	N 1.0175765 2.0204575 -1.5414903<br>N 2.3109365 0.1868065 -1.6539903<br>N -0.0754735 2.2698975 1.1233197<br>N -0.3494735 0.0317075 1.6482197<br>N 3.7172365 -0.6131535 2.0775797<br>C 0.4923375 3.2990775 -1.0621503<br>C -0.0928725 4.7047975 0.9744297<br>C 0.2812975 5.8050375 -1.2731903<br>C 0.4398775 5.9457075 0.2469397<br>C 1.0099075 4.5541675 -1.7825703<br>C 0.5832775 3.4129375 0.4873197<br>C 2.2865065 1.5488265 -1.4727603<br>C 3.2888465 -0.7767535 -1.3870603<br>C 5.0506555 -2.8924245 -0.7272103<br>C 4.6728665 -0.5522135 -1.3069203<br>C 2.8049055 -2.0813635 -1.1615603<br>C 3.6788255 -3.1134135 -0.8416803<br>C 5.5236755 -1.6033745 -0.9632803<br>C 6.9798755 -1.3245245 -0.7248403<br>C 3.1046455 -4.4542835 -0.4871803

	C	0.5006865	1.0858175	1.4471497
	C	1.4371555	-1.4703835	2.1001797
	C	1.8697565	0.8760065	1.6333497
	C	0.1103955	-1.2119025	1.9370697
	C	2.3817165	-0.4007935	1.9431597
	C	4.6451765	0.5150865	2.0315597
	C	4.2375055	-1.8898735	2.5615597
	F	7.7691755	-2.3488345	-1.1205403
	F	7.2295455	-1.1502445	0.6085197
	F	7.4131365	-0.2109445	-1.3467903
	F	3.9993345	-5.4547435	-0.6371603
	F	2.7044355	-4.4904335	0.8205397
	F	2.0164845	-4.7633035	-1.2217503
	H	4.0524755	-2.0256035	3.6368097
	H	0.2559365	1.3308075	-1.6211603
	H	1.3622965	-0.2306225	-1.6563203
	H	-1.0779835	2.2199675	0.9497297
	H	-0.5768025	3.2230975	-1.2899303
	H	-1.1763025	4.6195375	0.7979597
	H	0.6730485	6.6939875	-1.7820503
	H	1.5035385	6.0816265	0.4883097
	H	0.8627975	4.4209775	-2.8609203
	H	1.6364675	3.4278765	0.7732297
	H	5.7265255	-3.6960745	-0.4602603
	H	5.0704665	0.4352455	-1.4841803
	H	1.7384755	-2.2740635	-1.2115703
	H	1.7474255	-2.4837535	2.3074297
	H	2.5333165	1.7104165	1.4775797
	H	-0.6579745	-1.9673925	2.0270597
	H	5.6651665	0.1342055	2.0062797
	H	5.3129155	-1.9155145	2.3826497
	H	0.0426175	4.7904875	2.0587297
	H	-0.7893725	5.7451575	-1.5224403
	H	-0.0840515	6.8385775	0.6097697
	H	2.0835075	4.6599565	-1.6154403
	H	4.5287865	1.1695065	2.9072697
	H	3.8009555	-2.7281535	2.0181697
	H	4.4932765	1.1043365	1.1233797
	N	-1.2887935	-0.6333625	-1.2288303
	C	-2.2802145	-1.4747315	-1.0144203
	C	-3.7074345	-1.0530415	-1.1198503
	C	-4.6235245	-2.2450215	-1.3569503
	C	-6.3859845	-4.3989705	-1.7785903
	C	-4.5615245	-3.3907915	-0.5496403
	C	-5.5827245	-2.1961015	-2.3754203
	C	-6.4570145	-3.2638005	-2.5876903
	C	-5.4342245	-4.4588115	-0.7582003
	O	-0.0639345	-1.0565025	-1.1767203
	O	-1.5029835	0.6230675	-1.4777503
	H	-1.9834045	-2.4929325	-0.8175503

	H	-3.8059435	-0.3550515	-1.9583803
	H	-3.8230245	-3.4527115	0.2454497
	H	-5.6422445	-1.3146115	-3.0097303
	H	-7.1906045	-3.2085805	-3.3878303
	H	-5.3689355	-5.3392915	-0.1241703
	H	-7.0646755	-5.2316305	-1.9430803
	C	-4.2686435	-0.2164415	0.1234897
	C	-3.6613235	1.1723385	0.1717597
	C	-3.6108625	3.2320485	-0.9823303
	C	-4.0699635	-0.9570215	1.4257197
	C	-5.1570545	-2.1508615	3.1436697
	O	-2.8731535	1.5788385	1.0128997
	O	-4.1841535	1.9253385	-0.7959503
	O	-2.9914145	-1.2046915	1.9351397
	O	-5.2391945	-1.3575515	1.9422997
	H	-5.3387835	-0.0907615	-0.0556503
	H	-4.2538225	3.7341385	-1.7061603
	H	-3.5897025	3.7874785	-0.0411103
	H	-2.6026125	3.1229885	-1.3857803
	H	-4.6498045	-1.5955515	3.9371697
	H	-6.1895445	-2.3628005	3.4225697
	H	-4.6165345	-3.0816015	2.9488397
	H	-1.3553935	0.1456375	1.5097297
	S	3.6517065	2.5233965	-1.1758003

Name	$TS_{Theo2S-BMA-R1}$
**Absolute Energies (Hartree)**	There are 15 positive frequencies below 100 cm^-1.<br><br>Zero-point correction: 0.530877 hartree<br>Enthalpy correction: 0.568814 hartree<br>Free Energy correction: 0.461539 hartree<br>Quasiharmonic Free Energy correction: 0.470818 hartree<br><br>SCF Energy: -1980.330375 hartree<br>SCF Energy*: -1980.816138 hartree<br>SCF Energy + ZPVE: -1979.799498 hartree<br>Enthalpy: -1979.761561 hartree<br>Free Energy: -1979.868836 hartree<br><br>Free Energy with quasiharmonic correction: -1979.859557 hartree (correction: 5.82 kcal/mol)<br><br>---<br>Low frequencies before projection:<br>-325.8289<br>22.3402<br>23.7138<br><br>Low frequencies after projection:<br>-325.8289<br>22.3146<br>23.5716<br>---
**Cartesian Coordinates**	N 4.6412355 -0.7644143 -0.8955031<br>C 5.1950555 0.4245857 -0.5618331<br>N 5.2670755 0.6631257 0.7688669<br>N -2.9904845 0.7670957 -1.0565431<br>O 3.5384855 -0.6934343 2.6362369<br>N 2.6978655 -1.3373143 1.9353569<br>O 3.0887355 -2.2175343 1.0995969<br>C 1.3728355 -1.0665043 2.0687169<br>C -0.3128045 -1.8565243 -1.4422631<br>C 0.7477555 -1.1822043 -0.7110831<br>C 0.7761855 0.2622457 -0.6569431<br>O -0.1786245 1.0303757 -0.8444931<br>O 1.9785455 0.7092457 -0.2640131<br>C 2.1216655 2.1227657 -0.0556831<br>O -1.3636045 -1.3747043 -1.8600131<br>O -0.0412145 -3.1799543 -1.5629431<br>C -1.0531645 -3.9693143 -2.2058331<br>C -3.2361245 2.0025157 -0.5285731<br>N -2.1929645 2.7554957 -0.1557431<br>C 5.8876855 1.8308057 1.3591669<br>C 0.4172155 -1.7202843 1.2541569

	C	4.2450055	-1.1196943	-2.2428631
	C	-1.0244945	-1.4608643	1.4900169
	C	-3.7770645	-1.0393243	1.9207969
	C	-1.5022745	-0.2097343	1.9173969
	C	-1.9562645	-2.4869143	1.2598169
	C	-3.3185345	-2.2805243	1.4732469
	C	-2.8628045	-0.0063943	2.1411969
	H	3.4775155	-0.4399643	-2.6362731
	H	3.8417055	-2.1362243	-2.2147031
	H	5.1009755	-1.0954543	-2.9233231
	H	4.2205555	-1.3177843	-0.1451231
	H	4.7913155	0.0240857	1.4125869
	H	5.3993855	2.7613357	1.0433769
	H	5.8051755	1.7357157	2.4455969
	H	6.9457155	1.9045057	1.0847469
	H	-0.6783345	-4.9939943	-2.1902431
	H	-2.0022745	-3.9050743	-1.6646931
	H	-1.2074845	-3.6400443	-3.2380331
	H	-2.0119245	0.4409757	-1.1568331
	H	-1.2617345	2.3643257	-0.3031331
	H	0.6575455	-2.7464543	0.9896669
	H	1.1545755	-0.2349543	2.7209069
	H	-0.8081845	0.6104657	2.0693869
	H	-3.2115145	0.9648957	2.4814169
	H	-4.8376245	-0.8757643	2.0907969
	H	-4.0209345	-3.0906843	1.2958369
	H	-1.6016545	-3.4550243	0.9155069
	H	3.1574655	2.2629057	0.2469769
	H	1.9213155	2.6709057	-0.9802531
	H	1.4431055	2.4653057	0.7330369
	H	1.7203255	-1.6584043	-0.7421831
	C	-2.3171145	4.0864357	0.4128469
	H	-2.8717145	4.0699557	1.3592069
	H	-1.3114645	4.4620757	0.6086269
	H	-2.8161045	4.7766557	-0.2781131
	C	-3.9710645	-0.0874843	-1.4342731
	H	-3.6135845	-1.0335243	-1.8165531
	C	-5.2934845	0.2612057	-1.3135531
	H	-6.0704445	-0.4280143	-1.6198631
	C	-5.5953145	1.5299757	-0.7790931
	H	-6.6313345	1.8368657	-0.6682731
	C	-4.5929645	2.3927757	-0.3877831
	H	-4.8245945	3.3653557	0.0268669
	S	5.7492455	1.5495057	-1.7253331

Name	$TS_{Theo2S-BMA-R11}$
**Absolute Energies (Hartree)**	There are 15 positive frequencies below 100 cm^-1.<br><br>Zero-point correction: 0.530767 hartree<br>Enthalpy correction: 0.568723 hartree<br>Free Energy correction: 0.459856 hartree<br>Quasiharmonic Free Energy correction: 0.471086 hartree<br><br>SCF Energy: -1980.327170 hartree<br>SCF Energy*: -1980.813687 hartree<br>SCF Energy + ZPVE: -1979.796403 hartree<br>Enthalpy: -1979.758447 hartree<br>Free Energy: -1979.867314 hartree<br><br>Free Energy with quasiharmonic correction: -1979.856084 hartree (correction: 7.05 kcal/mol)<br><br>---<br>Low frequencies before projection:<br>-318.6023<br>13.6509<br>17.9959<br><br>Low frequencies after projection:<br>-318.6022<br>10.3087<br>17.5476<br>---
**Cartesian Coordinates**	N 4.7006503 -0.5337554 -0.7086528<br>C 5.1653703 0.7146646 -0.4693328<br>N 5.1748503 1.0759946 0.8354672<br>N -3.1445197 0.2440736 -1.3848428<br>O 3.4899703 -0.2387054 2.7758072<br>N 2.7145503 -0.9924254 2.1104272<br>O 3.1862703 -1.9149754 1.3677172<br>C 1.3716903 -0.7934564 2.1791972<br>C -0.1069397 -2.0068464 -1.3101428<br>C 0.8618503 -1.1833464 -0.6035728<br>C 0.7912403 0.2576136 -0.6884828<br>O -0.1872697 0.9393736 -1.0241428<br>O 1.9325703 0.8193036 -0.2561928<br>C 1.9788003 2.2517636 -0.1890228<br>O -1.1718697 -1.6617964 -1.8162328<br>O 0.2773703 -3.3086264 -1.2916128<br>C -0.6364297 -4.2358064 -1.8963128<br>C -3.4623997 1.4371936 -0.8896028<br>C -4.7935197 1.9140236 -0.7199228<br>N -2.4530297 2.2684036 -0.4876328<br>C -2.6597197 3.4975236 0.0484972

	C	-3.9292197	3.9801536	0.2276872
	C	-5.0087597	3.1576836	-0.1720128
	C	5.7056703	2.3274946	1.3346472
	C	0.4851403	-1.5686064	1.3949372
	C	4.3768703	-1.0361354	-2.0283328
	C	-4.1326697	-0.7514864	-1.7678128
	C	-0.9757697	-1.3720364	1.5614172
	C	-3.7582097	-1.0713764	1.8839672
	C	-1.5329897	-0.1192664	1.8717672
	C	-1.8435897	-2.4630464	1.3888972
	C	-3.2206597	-2.3166564	1.5503572
	C	-2.9082697	0.0265636	2.0412972
	H	3.5743103	-0.4552454	-2.5022228
	H	4.0491103	-2.0740754	-1.9190228
	H	5.2509903	-1.0058154	-2.6850328
	H	4.2945703	-1.0471154	0.0772072
	H	4.7261503	0.4629846	1.5222372
	H	5.1851003	3.1938846	0.9079472
	H	5.5764603	2.3320446	2.4206872
	H	6.7706003	2.4348046	1.0996872
	H	-0.1839597	-5.2201064	-1.7649928
	H	-1.6137797	-4.1988164	-1.4052728
	H	-0.7646597	-4.0184164	-2.9611728
	H	-3.5848097	-1.6381764	-2.0829328
	H	-4.7756697	-1.0172464	-0.9208328
	H	-4.7552297	-0.3990464	-2.5987028
	H	-2.1638997	-0.0585364	-1.4265728
	H	-1.4862797	1.9196436	-0.6018628
	H	-1.7619397	4.0429836	0.3149872
	H	-4.0883297	4.9610236	0.6578272
	H	-5.6211897	1.2865836	-1.0238628
	H	-6.0276097	3.5118236	-0.0450228
	H	0.7907803	-2.5974864	1.2262972
	H	1.0833703	0.0757036	2.7499172
	H	-0.8867697	0.7463136	1.9790072
	H	-3.3193497	1.0009436	2.2914772
	H	-4.8302897	-0.9560064	2.0200272
	H	-3.8727797	-3.1765264	1.4223272
	H	-1.4267997	-3.4341564	1.1345972
	H	2.9689603	2.4868946	0.1964872
	H	1.8465803	2.6889036	-1.1823528
	H	1.2055903	2.6314936	0.4880172
	H	1.8650203	-1.5880464	-0.5433928
	S	5.6887203	1.7653146	-1.7137128

Name	TS$_{Theo2S\text{-}BMA\text{-}R12}$
Absolute Energies (Hartree)	There are 14 positive frequencies below 100 cm^-1.<br><br>Zero-point correction: 0.530957 hartree<br>Enthalpy correction: 0.568812 hartree<br>Free Energy correction: 0.461725 hartree<br>Quasiharmonic Free Energy correction: 0.470795 hartree<br><br>SCF Energy: -1980.328609 hartree<br>SCF Energy*: -1980.813130 hartree<br>SCF Energy + ZPVE: -1979.797652 hartree<br>Enthalpy: -1979.759797 hartree<br>Free Energy: -1979.866884 hartree<br><br>Free Energy with quasiharmonic correction: -1979.857815 hartree (correction: 5.69 kcal/mol)<br><br>---<br>Low frequencies before projection:<br>-351.3753<br>20.7607<br>22.0468<br><br>Low frequencies after projection:<br>-351.3753<br>20.3758<br>21.8607<br>---
**Cartesian Coordinates**	N 2.7928525 2.1100209 -0.1240606<br>C 4.0060725 1.5804209 0.0225294<br>C 6.4719025 0.2774399 0.4052094<br>C 5.1877625 2.0869509 -0.5882906<br>N 4.1396225 0.4566309 0.7894194<br>C 5.3186125 -0.1838791 0.9835894<br>C 6.3872525 1.4403499 -0.3944506<br>N -6.2339575 -0.4692161 -0.8670906<br>C -6.2634475 0.2588439 0.2709394<br>N -5.0651075 0.3503339 0.9049494<br>N -2.7675085 -1.2614371 -1.1537506<br>O -3.5873475 -0.8167771 -2.0162206<br>O -3.1812285 -1.7274771 -0.0441006<br>C -1.4359385 -1.2179371 -1.4234006<br>C -0.4895885 -1.6357681 -0.4536206<br>C -0.5717175 -0.3753181 1.1506294<br>C -0.7942475 0.9263419 0.5276694<br>O 0.0505325 1.7075319 0.0949894<br>O -2.1137575 1.1343829 0.3613694<br>C -2.5093275 2.2526429 -0.4490206<br>C 0.6637425 -0.6366781 1.8524694

	O	1.6920725	0.0516419	1.8332194
	O	0.6135715	-1.8098681	2.5213994
	C	1.8235215	-2.2214881	3.1734194
	C	-4.8721175	0.9914729	2.1895194
	C	-7.3700175	-0.6561861	-1.7447106
	C	2.5194235	3.2734709	-0.9534506
	H	-1.4608475	-0.8239371	1.5777794
	H	-3.7997375	0.9892629	2.3998294
	H	-5.2289775	2.0252739	2.1647394
	H	-5.4041875	0.4735439	2.9989894
	H	-4.3327375	-0.2963171	0.6051594
	H	-5.3169475	-0.7563961	-1.2227706
	H	-8.1958375	-1.1549261	-1.2259006
	H	-7.0437085	-1.2827261	-2.5800506
	H	-7.7484775	0.2963639	-2.1369306
	H	-3.5933675	2.1778929	-0.5247906
	H	-1.1815475	-0.7268381	-2.3503006
	H	3.0475535	4.1606909	-0.5854306
	H	1.4462335	3.4535919	-0.9073406
	H	-2.2182465	3.1949229	0.0249194
	H	-2.0509875	2.1852329	-1.4396306
	H	1.9704725	1.6464619	0.2801194
	H	3.2816125	0.0874609	1.2332894
	H	5.2691325	-1.0655891	1.6108294
	H	7.4108225	-0.2383001	0.5630994
	H	7.2824525	1.8318399	-0.8686806
	H	5.1264035	2.9756009	-1.2027606
	H	2.8034235	3.0982209	-1.9987406
	H	2.1562815	-1.4689281	3.8943194
	H	1.5765015	-3.1517781	3.6870594
	H	2.6152515	-2.4005691	2.4386194
	H	-0.8068485	-2.4620681	0.1765294
	C	0.9391315	-1.6897881	-0.8602706
	C	3.6402815	-1.8700991	-1.6367706
	C	1.5202625	-0.6985081	-1.6692706
	C	1.7440915	-2.7539981	-0.4217406
	C	3.0821515	-2.8460491	-0.8078606
	C	2.8542225	-0.7956291	-2.0615906
	H	0.9284025	0.1571419	-1.9762706
	H	1.3094515	-3.5201081	0.2143894
	H	3.6830815	-3.6875791	-0.4725206
	H	3.2843725	-0.0266591	-2.6976406
	H	4.6798815	-1.9416091	-1.9448006
	S	-7.6806475	1.0184539	0.8532394

Name	$TS_{Theo2S-BMA-R13}$
**Absolute Energies (Hartree)**	There are 14 positive frequencies below 100 cm^-1.<br><br>Zero-point correction: 0.531049 hartree<br>Enthalpy correction: 0.568918 hartree<br>Free Energy correction: 0.461415 hartree<br>Quasiharmonic Free Energy correction: 0.470853 hartree<br><br>SCF Energy: -1980.330926 hartree<br>SCF Energy*: -1980.815986 hartree<br>SCF Energy + ZPVE: -1979.799877 hartree<br>Enthalpy: -1979.762008 hartree<br>Free Energy: -1979.869511 hartree<br><br>Free Energy with quasiharmonic correction: -1979.860073 hartree (correction: 5.92 kcal/mol)<br><br>---<br>Low frequencies before projection:<br>-350.7330<br>14.1279<br>21.1119<br><br>Low frequencies after projection:<br>-350.733<br>12.2114<br>20.9127<br>---
**Cartesian Coordinates**	N 4.0899308 0.0704192 1.1180271<br>C 3.9357108 1.3152892 0.6552671<br>C 3.4121928 3.8913802 -0.3347029<br>C 5.0074818 2.1652692 0.2745971<br>N 2.6682118 1.8038702 0.5121471<br>C 2.3983318 3.0453002 0.0382771<br>C 4.7387518 3.4276592 -0.2093929<br>N -5.7672292 0.4255842 -0.8688129<br>C -5.7190092 0.4176942 0.4852771<br>N -4.8960192 -0.5172358 1.0116671<br>N -2.7897002 -1.3188668 -1.6930229<br>O -3.6331402 -0.7396568 -2.4447229<br>O -3.1755902 -2.0667068 -0.7357129<br>C -1.4622002 -1.1251078 -1.9087929<br>C -0.5045602 -1.6705278 -1.0164329<br>C -0.7385002 -0.7871178 0.8088371<br>C 0.4499098 -1.1859788 1.5289371<br>O 1.4672408 -0.5087788 1.7218271<br>O 0.3755798 -2.4733288 1.9317071<br>C 1.5466588 -3.0096388 2.5654771<br>C -0.9427692 0.6174422 0.4731171

	O	-0.0818782	1.4874812	0.3407071
	O	-2.2383392	0.8403522	0.1931771
	C	-2.5814382	2.1178032	-0.3673729
	C	-4.5106092	-0.5715268	2.4055771
	C	-6.6179892	1.2978252	-1.6520729
	C	5.3730608	-0.5995408	1.2229671
	H	-1.6450402	-1.3283078	1.0531071
	H	-5.3880602	-0.6485658	3.0542971
	H	-3.9393992	0.3160832	2.7084671
	H	-3.8868102	-1.4597268	2.5441771
	H	-6.4578192	1.0570242	-2.7069429
	H	-7.6757392	1.1503452	-1.4077429
	H	-6.3832582	2.3566542	-1.4868229
	H	1.2823488	-4.0287888	2.8513871
	H	-1.2340092	-0.4079978	-2.6826629
	H	6.0392208	-0.0891418	1.9288071
	H	5.1947498	-1.6119008	1.5884871
	H	1.8150898	-2.4261498	3.4509571
	H	2.3904488	-3.0243898	1.8680971
	H	3.2406608	-0.4443098	1.3555271
	H	1.8542408	1.2086502	0.7496671
	H	1.3441518	3.2797512	-0.0241429
	H	3.1873328	4.8804902	-0.7136829
	H	5.5644728	4.0713692	-0.4982529
	H	6.0249918	1.8089082	0.3704171
	H	5.8707398	-0.6675318	0.2481071
	H	-2.4569682	2.9096622	0.3771271
	H	-3.6269382	2.0309732	-0.6592729
	H	-1.9570182	2.3337422	-1.2391929
	H	-0.7676002	-2.6341378	-0.5889229
	C	0.9355698	-1.5213788	-1.3494629
	C	3.6760398	-1.2968808	-1.9536829
	C	1.4535108	-0.3375188	-1.9008429
	C	1.8198498	-2.5801298	-1.0838329
	C	3.1775198	-2.4717998	-1.3862329
	C	2.8093208	-0.2303298	-2.2059829
	H	0.7951608	0.5074712	-2.0716929
	H	1.4324388	-3.4967388	-0.6468429
	H	3.8431888	-3.3077608	-1.1868029
	H	3.1913308	0.6915302	-2.6366829
	H	4.7318098	-1.2122408	-2.1977929
	H	-5.1083992	-0.1628258	-1.3880229
	H	-4.3329602	-1.0751768	0.3645771
	S	-6.6182582	1.5117152	1.4457571

Name	$TS_{Theo2S-BMA-R2}$
**Absolute Energies (Hartree)**	There are 15 positive frequencies below 100 cm^-1.<br><br>Zero-point correction: 0.530521 hartree<br>Enthalpy correction: 0.568614 hartree<br>Free Energy correction: 0.459115 hartree<br>Quasiharmonic Free Energy correction: 0.470328 hartree<br><br>SCF Energy: -1980.334321 hartree<br>SCF Energy*: -1980.818524 hartree<br>SCF Energy + ZPVE: -1979.803800 hartree<br>Enthalpy: -1979.765707 hartree<br>Free Energy: -1979.875206 hartree<br><br>Free Energy with quasiharmonic correction: -1979.863993 hartree (correction: 7.04 kcal/mol)<br><br>--<br>Low frequencies before projection:<br>-199.1696<br>7.7297<br>16.5002<br><br>Low frequencies after projection:<br>-199.1695<br>5.1837<br>15.8102<br>--
**Cartesian Coordinates**	N -3.6667408 -0.8421708 1.5610926<br>C -4.4990308 -1.3285108 0.6103126<br>N -3.9022808 -2.1831008 -0.2600074<br>N -1.1615908 1.9272292 -0.6901174<br>H -2.6671208 -0.9835008 1.4056326<br>H -2.9894008 -2.5543808 0.0128926<br>H -0.3873808 1.4096492 -1.1448874<br>O -1.2426708 -3.2412408 0.5882226<br>N -0.4799608 -2.2334508 0.5996526<br>O -0.9641508 -1.0571008 0.5496426<br>C 0.8752792 -2.4129708 0.6748926<br>C 0.6416592 -0.8765808 -2.0641874<br>C 1.9800192 -0.7316208 -1.5328774<br>C 2.5398492 0.5828992 -1.3607274<br>O 1.9104992 1.6108892 -1.0807574<br>O 3.8915292 0.5827592 -1.4349674<br>C 4.5418292 1.8188092 -1.1151474<br>H 5.6110792 1.6032192 -1.1445674<br>H 4.2585292 2.1672992 -0.1176574<br>H 4.2929992 2.5920592 -1.8493174<br>O -0.1779008 0.0116192 -2.3141474

	O	0.3442792	-2.1836808	-2.2616974
	C	-1.0100508	-2.4611108	-2.6486474
	H	-1.0861208	-3.5488408	-2.6822074
	H	-1.2312908	-2.0330108	-3.6312174
	H	-1.7071608	-2.0535708	-1.9125774
	H	1.0363992	3.0555892	-0.4898174
	C	-0.9535008	3.1513692	-0.1260174
	N	0.2912592	3.6525392	-0.1318074
	C	-4.6039308	-2.8814208	-1.3198574
	H	-5.3864908	-3.5446908	-0.9306574
	H	-3.8692308	-3.4792308	-1.8670674
	H	-5.0749108	-2.1744308	-2.0095874
	C	1.7270792	-1.3093208	0.6256926
	H	1.1856292	-3.4465508	0.6704726
	C	-4.0684408	0.0499092	2.6271826
	H	-4.8682608	-0.3929708	3.2291526
	H	-4.4280208	1.0147692	2.2490926
	H	-3.1954308	0.2218592	3.2633826
	C	-2.3765508	1.3260592	-0.7419474
	H	-2.3735908	0.3485192	-1.2025574
	H	2.6724092	-1.5315108	-1.7603474
	H	1.2565492	-0.3509708	0.8164526
	C	3.1493492	-1.4097908	0.9986026
	C	5.8616592	-1.5038508	1.7376426
	C	3.9148692	-2.5653408	0.7659426
	C	3.7701492	-0.3019808	1.5990526
	C	5.1128592	-0.3487908	1.9704526
	C	5.2574392	-2.6103508	1.1325426
	H	3.4623992	-3.4246708	0.2793326
	H	3.1889892	0.6001992	1.7733526
	H	5.5742092	0.5163192	2.4394426
	H	5.8367892	-3.5097508	0.9420826
	H	6.9096992	-1.5426208	2.0220126
	C	0.6418092	4.9390892	0.4417526
	H	0.0986092	5.7573192	-0.0461774
	H	0.4389892	4.9753492	1.5199726
	H	1.7106092	5.0960092	0.2854526
	C	-2.0800208	3.8131192	0.4237426
	H	-1.9518608	4.7841492	0.8845826
	C	-3.3210708	3.2097892	0.3658126
	H	-4.1797508	3.7217892	0.7907426
	C	-3.4892008	1.9426292	-0.2255774
	H	-4.4515408	1.4449192	-0.2557874
	S	-6.1406508	-0.8746408	0.4966326

Name	$TS_{Theo2S\text{-}BMA\text{-}R21}$
**Absolute Energies (Hartree)**	There are 14 positive frequencies below 100 cm^-1.<br><br>Zero-point correction: 0.531372 hartree<br>Enthalpy correction: 0.569020 hartree<br>Free Energy correction: 0.463452 hartree<br>Quasiharmonic Free Energy correction: 0.471460 hartree<br><br>SCF Energy: -1980.328152 hartree<br>SCF Energy*: -1980.812391 hartree<br>SCF Energy + ZPVE: -1979.796780 hartree<br>Enthalpy: -1979.759132 hartree<br>Free Energy: -1979.864700 hartree<br><br>Free Energy with quasiharmonic correction: -1979.856692 hartree (correction: 5.02 kcal/mol)<br><br>---<br>Low frequencies before projection:<br>-223.3907<br>21.5222<br>25.1294<br><br>Low frequencies after projection:<br>-223.3907<br>20.9961<br>25.025<br>---
**Cartesian Coordinates**	N -1.0169978 2.7400171 0.4900697<br>C 0.3126722 2.6108471 0.7988497<br>C 1.2084722 3.5230871 0.1680097<br>N 0.6777922 1.6594071 1.6480697<br>C 2.0680722 1.3675071 1.9783197<br>N 4.3707922 -1.7130329 -0.4881403<br>C 4.8414622 -0.4514429 -0.6275703<br>N 3.9153922 0.4366071 -1.0722603<br>N 0.9002222 -1.5382929 -1.2141603<br>O 1.7672822 -2.3035029 -1.7304003<br>O 1.2600922 -0.5289029 -0.5202003<br>C -0.4239578 -1.7895029 -1.4127203<br>C -1.3895678 -0.9685029 -0.8095203<br>C -1.6678078 -1.6129329 1.2422397<br>C -2.5053778 -0.5380229 1.7198497<br>O -2.1275778 0.6078271 2.0137297<br>O -3.8146278 -0.8637029 1.7185397<br>C -4.7350778 0.1971771 2.0111197<br>C -0.3177178 -1.7624129 1.7844697<br>O 0.2549422 -1.0214429 2.5766897<br>O 0.2615322 -2.8817129 1.2907397

	C	1.6561722	-3.0423129	1.6000897
	C	4.1817422	1.8361771	-1.3241103
	C	5.1757122	-2.8393229	-0.0608503
	C	-1.5049978	3.6755671	-0.3696003
	H	-2.1765078	-2.5412229	1.0115197
	H	4.9746122	1.9614771	-2.0690603
	H	3.2605822	2.2860071	-1.7058603
	H	4.4959322	2.3686171	-0.4164303
	H	2.9416322	0.1239071	-1.0769103
	H	3.4512722	-1.9210429	-0.8871403
	H	5.5929322	-2.6715429	0.9375597
	H	6.0109722	-3.0307729	-0.7458603
	H	4.5271222	-3.7197229	-0.0346503
	H	-5.7280678	-0.2431229	1.9097497
	H	-0.6335578	-2.6934529	-1.9641103
	H	-4.5871178	0.5720771	3.0283497
	H	-4.6200678	1.0214571	1.3014897
	H	-1.6635378	2.0861571	0.9418297
	H	-0.0268678	0.9910471	1.9721897
	H	2.0641622	0.5073871	2.6439397
	H	2.2666222	3.4485571	0.3801497
	H	-2.5784478	3.6573571	-0.5141803
	H	1.8060522	-3.1536529	2.6783197
	H	1.9652322	-3.9490529	1.0785497
	H	2.2229622	-2.1800529	1.2393497
	H	-1.0222078	0.0030071	-0.4972603
	C	-2.7930378	-0.9859629	-1.2763003
	C	-5.4787678	-0.9326329	-2.1118603
	C	-3.4608278	-2.1745129	-1.6129503
	C	-3.4952878	0.2260771	-1.3632803
	C	-4.8255678	0.2558071	-1.7810603
	C	-4.7903078	-2.1468029	-2.0269203
	H	-2.9410878	-3.1249129	-1.5291303
	H	-2.9833478	1.1507671	-1.1076403
	H	-5.3514878	1.2047871	-1.8481803
	H	-5.2946878	-3.0757929	-2.2786703
	H	-6.5164878	-0.9151529	-2.4333603
	C	-0.6646278	4.5582071	-0.9897203
	C	0.7205022	4.4665771	-0.7036203
	H	2.6398622	1.1070671	1.0833997
	H	2.5370822	2.2195371	2.4827297
	H	-1.0556778	5.2982471	-1.6766103
	H	1.4129022	5.1522671	-1.1828703
	S	6.4485022	-0.0061629	-0.2667403

Name	$TS_{Theo2S-BMA-R22}$
**Absolute Energies (Hartree)**	There are 14 positive frequencies below 100 cm^-1.<br><br>Zero-point correction: 0.530947 hartree<br>Enthalpy correction: 0.568655 hartree<br>Free Energy correction: 0.462072 hartree<br>Quasiharmonic Free Energy correction: 0.471111 hartree<br><br>SCF Energy: -1980.339346 hartree<br>SCF Energy*: -1980.822801 hartree<br>SCF Energy + ZPVE: -1979.808399 hartree<br>Enthalpy: -1979.770691 hartree<br>Free Energy: -1979.877274 hartree<br><br>Free Energy with quasiharmonic correction: -1979.868235 hartree (correction: 5.67 kcal/mol)<br><br>---<br>Low frequencies before projection:<br>-206.7433<br>15.5804<br>19.7342<br><br>Low frequencies after projection:<br>-206.7432<br>15.5066<br>19.6121<br>---
**Cartesian Coordinates**	N -0.0551576 2.7737915 0.1363420<br>C 1.2764034 2.6557415 0.0797920<br>C 4.0559034 2.2177925 0.0701920<br>C 2.1318024 3.5107715 -0.6644480<br>N 1.8677334 1.6408815 0.7742920<br>C 3.2010334 1.4056215 0.7721120<br>C 3.4933024 3.2864925 -0.6579280<br>N 3.9008234 -2.0634275 0.4259820<br>C 4.4948434 -1.3280475 -0.5548680<br>N 3.6258334 -0.8309575 -1.4647980<br>N 0.4472034 -2.0426785 -0.3943280<br>O 1.2000544 -3.0553685 -0.3243680<br>O 0.9455034 -0.8685585 -0.3481380<br>C -0.9011666 -2.2136985 -0.5366380<br>C -1.7582666 -1.1111695 -0.5605380<br>C -2.1690866 -0.5292595 1.5724720<br>C -2.9623866 0.6646105 1.3643720<br>O -2.5483566 1.7816405 1.0500620<br>O -4.2882166 0.4024905 1.4712020<br>C -5.1671566 1.4899305 1.1549120<br>C -0.8288366 -0.4296385 2.0829020

	O	-0.1508866	0.6022115	2.2180420
	O	-0.3350766	-1.6482685	2.4010420
	C	1.0433434	-1.6946285	2.7961220
	C	4.0036834	-0.0368675	-2.6140480
	C	4.6397844	-2.7970975	1.4373220
	C	-0.7953476	3.8046915	-0.5706180
	H	-2.7100566	-1.4320195	1.8244120
	H	4.7011034	-0.5825675	-3.2578580
	H	3.0935134	0.1849915	-3.1785480
	H	4.4814834	0.9056125	-2.3220680
	H	2.6317434	-0.9088485	-1.2419180
	H	2.9575944	-2.4035485	0.2209320
	H	5.2928744	-3.5643575	1.0022020
	H	3.9112044	-3.2800875	2.0951220
	H	5.2640834	-2.1221875	2.0298220
	H	-6.1757366	1.0770405	1.2130020
	H	-1.2155256	-3.2463185	-0.5400880
	H	-0.5222376	4.8105915	-0.2265980
	H	-1.8523076	3.6362305	-0.3666280
	H	-5.0510966	2.3066805	1.8747620
	H	-4.9752466	1.8698905	0.1479520
	H	-0.6057366	2.1508315	0.7366820
	H	1.2406034	1.0205115	1.3179420
	H	3.5221234	0.5404225	1.3380520
	H	5.1163534	2.0016925	0.0559220
	H	4.1404224	3.9443325	-1.2310280
	H	1.7038124	4.3309115	-1.2261180
	H	-0.6279976	3.7457515	-1.6532480
	H	1.2220234	-1.0596385	3.6691320
	H	1.2399644	-2.7392385	3.0409320
	H	1.6852934	-1.3763985	1.9698220
	H	-1.2848766	-0.1496785	-0.7308880
	C	-3.1514366	-1.2264195	-1.0275880
	C	-5.8094566	-1.3450995	-1.9385680
	C	-3.9255856	-2.3823495	-0.8275280
	C	-3.7356766	-0.1303695	-1.6841180
	C	-5.0514366	-0.1903095	-2.1407980
	C	-5.2412056	-2.4395195	-1.2790780
	H	-3.5035056	-3.2318895	-0.2984280
	H	-3.1508566	0.7742005	-1.8279380
	H	-5.4848566	0.6653705	-2.6517680
	H	-5.8282556	-3.3385295	-1.1119480
	H	-6.8367566	-1.3931905	-2.2893680
	S	6.1683434	-1.0094975	-0.6010180

Name	$TS_{Theo2S\text{-}BMA\text{-}R23}$
**Absolute Energies (Hartree)**	There are 14 positive frequencies below 100 cm^-1.<br><br>Zero-point correction: 0.531218 hartree<br>Enthalpy correction: 0.568834 hartree<br>Free Energy correction: 0.463037 hartree<br>Quasiharmonic Free Energy correction: 0.471515 hartree<br><br>SCF Energy: -1980.339082 hartree<br>SCF Energy*: -1980.822264 hartree<br>SCF Energy + ZPVE: -1979.807864 hartree<br>Enthalpy: -1979.770248 hartree<br>Free Energy: -1979.876045 hartree<br><br>Free Energy with quasiharmonic correction: -1979.867567 hartree (correction: 5.32 kcal/mol)<br><br>---<br>Low frequencies before projection:<br>-206.9554<br>18.9440<br>19.8544<br><br>Low frequencies after projection:<br>-206.9552<br>18.2579<br>19.764<br>---
**Cartesian Coordinates**	N 3.2215460 -0.4759471 -1.6759712<br>C 4.1373760 -1.1307171 -0.9261412<br>N 3.6011560 -1.9873771 -0.0191212<br>N 2.4372370 1.0531529 1.5791588<br>H 2.2408560 -0.5912671 -1.4128012<br>H 2.6285260 -2.2665371 -0.1704512<br>H 1.6505970 0.5805439 2.0289188<br>O 0.8550350 -2.9158661 -0.6052612<br>N 0.1058260 -1.8969661 -0.5980012<br>O 0.6058760 -0.7293861 -0.4810412<br>C -1.2466040 -2.0484661 -0.7106612<br>C -0.9694240 -0.4781861 2.0241788<br>C -2.2845340 -0.2838151 1.4676488<br>C -2.7658130 1.0637749 1.2397988<br>O -2.0859930 2.0287449 0.8838388<br>O -4.1137330 1.1448149 1.3558988<br>C -4.6979730 2.4030459 0.9945888<br>H -5.7767230 2.2593759 1.0759888<br>H -4.4324630 2.6814349 -0.0294012<br>H -4.3696230 3.1951949 1.6750388<br>O -0.1438140 0.4025739 2.3148888

	O	-0.6975340	-1.7885961	2.2109188
	C	0.6482460	-2.1074561	2.5963488
	H	0.6833650	-3.1956161	2.6605188
	H	0.8934160	-1.6626561	3.5660088
	H	1.3520860	-1.7559661	1.8363488
	H	0.0944570	1.6841539	0.9294688
	C	2.1293270	1.9321229	0.6229688
	C	3.0836970	2.6392829	-0.1563112
	N	0.8083570	2.1716139	0.3632988
	C	0.3797170	3.0230039	-0.5997612
	H	-0.6966130	3.1009239	-0.6788412
	C	1.2766570	3.7119239	-1.3757012
	H	0.9281180	4.3918639	-2.1431912
	C	2.6539270	3.5075529	-1.1345512
	H	3.3904780	4.0350129	-1.7336112
	C	4.3879650	-2.8369071	0.8528188
	H	5.0143750	-3.5423671	0.2924088
	H	3.6920650	-3.3999471	1.4823088
	H	5.0468060	-2.2410871	1.4919888
	C	-2.0738040	-0.9238251	-0.6696612
	H	-1.5801650	-3.0745351	-0.7338212
	H	4.1379970	2.4632029	0.0135588
	C	3.5379860	0.3936029	-2.7893012
	H	4.0693660	-0.1472471	-3.5809712
	H	4.1638970	1.2370929	-2.4803212
	H	2.5953270	0.7776529	-3.1890312
	C	3.7930770	0.7644529	2.0192488
	H	4.4277460	0.4302929	1.1934688
	H	3.7390660	-0.0376771	2.7569488
	H	4.2523970	1.6420529	2.4916388
	H	-3.0209840	-1.0379151	1.7132488
	H	-1.5846440	0.0206549	-0.8803112
	C	-3.4997540	-1.0014251	-1.0341112
	C	-6.2191240	-1.0520041	-1.7494212
	C	-4.2849840	-2.1397451	-0.7818712
	C	-4.1046640	0.1117649	-1.6406612
	C	-5.4510640	0.0865859	-2.0000912
	C	-5.6310640	-2.1634541	-1.1374912
	H	-3.8450250	-3.0014651	-0.2879812
	H	-3.5085530	1.0014049	-1.8275812
	H	-5.9003030	0.9559859	-2.4727112
	H	-6.2258850	-3.0494541	-0.9324612
	H	-7.2701740	-1.0737041	-2.0242712
	S	5.8170860	-0.8572781	-1.0705712

Name	$TS_{Theo2S-BMA-R31}$
**Absolute Energies (Hartree)**	There are 15 positive frequencies below 100 cm^-1.<br><br>Zero-point correction: 0.531100 hartree<br>Enthalpy correction: 0.568811 hartree<br>Free Energy correction: 0.462404 hartree<br>Quasiharmonic Free Energy correction: 0.471276 hartree<br><br>SCF Energy: -1980.340240 hartree<br>SCF Energy*: -1980.825063 hartree<br>SCF Energy + ZPVE: -1979.809140 hartree<br>Enthalpy: -1979.771429 hartree<br>Free Energy: -1979.877836 hartree<br><br>Free Energy with quasiharmonic correction: -1979.868964 hartree (correction: 5.57 kcal/mol)<br><br>---<br>Low frequencies before projection:<br>-199.7602<br>17.6121<br>24.2939<br><br>Low frequencies after projection:<br>-199.76<br>16.5118<br>24.1568<br>---
**Cartesian Coordinates**	N -2.1076399 1.2486807 1.2381797<br>C -1.6601499 2.2266607 0.3947297<br>N -0.3448799 2.4324397 0.2920297<br>N -3.9183399 -0.5373593 -1.6197803<br>C -4.4694299 -1.3688393 -0.7098703<br>N -3.5717399 -1.9816493 0.1060497<br>N -0.2827899 -0.6177303 -0.9935203<br>O -1.0996099 0.1449107 -1.5930003<br>O -0.6909399 -1.6053303 -0.3100903<br>C 1.0565701 -0.3645103 -1.1307003<br>C 2.0180201 -1.1974903 -0.5567303<br>C 2.3961801 -0.5634703 1.5810697<br>C 1.0583701 -0.6712503 2.0852797<br>O 0.2131601 0.2368897 2.1062297<br>O 0.7709901 -1.9152703 2.5307697<br>C -0.5973499 -2.1426903 2.8896597<br>C 2.9727301 0.7385397 1.3201097<br>O 2.3741301 1.7602997 0.9853497<br>O 4.3278001 0.7015797 1.4062197<br>C 5.0067001 1.9143797 1.0589397<br>C -3.9242709 -3.0247593 1.0503197

	C	-4.6733399	0.2530207	-2.5685503
	C	-3.4208999	0.9695607	1.4358197
	H	3.0809201	-1.3578203	1.8493797
	H	-4.4190709	-3.8718093	0.5603397
	H	-3.0022809	-3.3767093	1.5210497
	H	-4.6014609	-2.6490393	1.8250497
	H	-2.5794299	-1.8676293	-0.1130103
	H	-2.9057299	-0.3924393	-1.5977103
	H	-5.2587099	-0.3815693	-3.2429803
	H	-3.9591599	0.8369307	-3.1558903
	H	-5.3678199	0.9362707	-2.0660303
	H	-0.6625709	-3.2023803	3.1427597
	H	1.2691101	0.5106297	-1.7231103
	H	-0.8844699	-1.5346703	3.7542497
	H	-1.2500899	-1.9131993	2.0437197
	H	-1.3864399	0.6722307	1.7014797
	H	0.3071901	1.8432397	0.8198497
	H	4.7602101	2.7151397	1.7638897
	H	6.0713801	1.6801097	1.1125197
	H	4.7453301	2.2382997	0.0476897
	H	1.6523501	-2.1431503	-0.1714903
	C	3.4176201	-1.1886903	-1.0197103
	C	6.1011201	-1.2609003	-1.8648903
	C	4.0023201	-0.0755503	-1.6491903
	C	4.2062591	-2.3338403	-0.8161503
	C	5.5334691	-2.3721603	-1.2368803
	C	5.3301801	-0.1132403	-2.0677303
	H	3.4256301	0.8325897	-1.7927103
	H	3.7684691	-3.1980403	-0.3224203
	H	6.1250791	-3.2689403	-1.0741003
	H	5.7659801	0.7570297	-2.5512803
	H	7.1366701	-1.2882003	-2.1928703
	C	0.2385801	3.2954897	-0.7195103
	H	-0.0617599	4.3410497	-0.5808303
	H	-0.0495399	2.9699397	-1.7269603
	H	1.3205101	3.2260297	-0.6135003
	C	-2.6470899	2.9615807	-0.3167703
	H	-2.3342399	3.7319407	-1.0091203
	C	-3.9810599	2.6866707	-0.1149403
	H	-4.7279299	3.2527007	-0.6637503
	C	-4.3941899	1.6778307	0.7827797
	H	-5.4374999	1.4277207	0.9230497
	H	-3.6263699	0.1537007	2.1162897
	S	-6.1547399	-1.5937593	-0.5590703

**Name**	**$TS_{Theo2S\text{-}BMA\text{-}R32}$**
**Absolute Energies (Hartree)**	There are 14 positive frequencies below 100 cm^-1.<br><br>Zero-point correction: 0.531625 hartree<br>Enthalpy correction: 0.569147 hartree<br>Free Energy correction: 0.464171 hartree<br>Quasiharmonic Free Energy correction: 0.472016 hartree<br><br>SCF Energy: -1980.332838 hartree<br>SCF Energy*: -1980.818032 hartree<br>SCF Energy + ZPVE: -1979.801213 hartree<br>Enthalpy: -1979.763691 hartree<br>Free Energy: -1979.868667 hartree<br><br>Free Energy with quasiharmonic correction: -1979.860822 hartree (correction: 4.92 kcal/mol)<br><br>---<br>Low frequencies before projection:<br>-234.9935<br>18.2653<br>35.9064<br><br>Low frequencies after projection:<br>-234.9929<br>17.7622<br>35.301<br>---
**Cartesian Coordinates**	N 3.5480347 -1.9799344 0.5331532<br>C 4.4894547 -1.0888534 0.9281532<br>N 3.9885147 0.0850266 1.3822732<br>N 0.4729537 2.6316056 -0.6851568<br>H 2.5610347 -1.7381944 0.6587432<br>H 2.9710447 0.1859656 1.4402432<br>H -0.4437863 2.2454946 -0.9347768<br>O 1.1597347 0.5918556 1.4663832<br>N 0.3103647 -0.2813144 1.0989732<br>O 0.6808747 -1.4253244 0.6924432<br>C -1.0117653 0.0401346 1.1677132<br>C -2.4997453 0.3193246 -1.5993568<br>C -2.3670153 -1.0700654 -1.2465868<br>C -1.2054453 -1.8219754 -1.7199868<br>O -0.2620053 -1.3971044 -2.3749868<br>O -1.2887153 -3.1179454 -1.3319868<br>C -0.1198243 -3.9066044 -1.6021668<br>H -0.3154143 -4.8846044 -1.1587268<br>H 0.7580557 -3.4454844 -1.1415268<br>H 0.0466457 -4.0069044 -2.6792368<br>O -1.5690063 1.1146046 -1.7909968

	O	-3.7870653	0.7329446	-1.5904568
	C	-4.0029663	2.1385646	-1.7690468
	H	-5.0829863	2.2788936	-1.7044068
	H	-3.6349463	2.4703346	-2.7447068
	H	-3.5046563	2.7177946	-0.9845668
	H	0.4271947	0.4649456	-1.9464068
	C	1.5694437	1.9217456	-1.0899468
	N	1.3807147	0.8098656	-1.7942068
	C	4.7893437	1.1365566	1.9740432
	H	4.1116837	1.9419666	2.2699932
	H	5.5262337	1.5282566	1.2637632
	H	5.3358447	0.7854466	2.8577732
	C	-2.0141653	-0.9044954	0.8714832
	H	-1.1973353	1.0466446	1.5078732
	C	3.8425757	-3.2877244	-0.0160068
	H	4.4422557	-3.8896934	0.6757432
	H	4.3934347	-3.2146934	-0.9610768
	H	2.8906057	-3.7949544	-0.1966268
	C	0.5455137	3.7613056	0.0651232
	H	-0.4070163	4.2063646	0.3270432
	H	-3.2870753	-1.6368554	-1.1725668
	H	-1.6855453	-1.9390554	0.8771132
	C	-3.3988953	-0.6812354	1.3462332
	C	-6.0448453	-0.3486464	2.2488632
	C	-4.2063953	-1.7939154	1.6351332
	C	-3.9477453	0.6023346	1.5064532
	C	-5.2561953	0.7666836	1.9554032
	C	-5.5149053	-1.6307364	2.0858532
	H	-3.7979153	-2.7931154	1.5041732
	H	-3.3536263	1.4794846	1.2684632
	H	-5.6631363	1.7675636	2.0734732
	H	-6.1204453	-2.5045164	2.3109532
	H	-7.0652053	-0.2192864	2.5993932
	C	2.8451437	2.4380056	-0.7226168
	H	3.7347437	1.8972956	-1.0153168
	C	2.9237737	3.5884456	0.0236332
	H	3.9001537	3.9696556	0.3069932
	C	1.7566737	4.2760756	0.4379332
	H	1.8092137	5.1794456	1.0326232
	C	2.4708747	-0.0548944	-2.2241368
	H	3.0441247	-0.4245444	-1.3695168
	H	2.0229747	-0.9054944	-2.7336768
	H	3.1422647	0.4694456	-2.9138168
	S	6.1642947	-1.4019134	0.8209832

Name	$TS_{Theo2S\text{-}BMA\text{-}R33}$
**Absolute Energies (Hartree)**	There are 15 positive frequencies below 100 cm^-1.<br><br>Zero-point correction: 0.531327 hartree<br>Enthalpy correction: 0.569052 hartree<br>Free Energy correction: 0.462492 hartree<br>Quasiharmonic Free Energy correction: 0.471636 hartree<br><br>SCF Energy: -1980.338190 hartree<br>SCF Energy*: -1980.821636 hartree<br>SCF Energy + ZPVE: -1979.806863 hartree<br>Enthalpy: -1979.769138 hartree<br>Free Energy: -1979.875698 hartree<br><br>Free Energy with quasiharmonic correction: -1979.866555 hartree (correction: 5.74 kcal/mol)<br><br>--<br>Low frequencies before projection:<br>-226.3478<br>15.7715<br>22.0256<br><br>Low frequencies after projection:<br>-226.3476<br>14.8383<br>21.717<br>--
Cartesian Coordinates	N 3.6752692 -1.5769590 -0.0658886<br>C 4.6240692 -0.9299490 0.6567714<br>N 4.1462892 -0.2833490 1.7433914<br>N -0.4991318 2.8587000 -0.5193086<br>H 2.7344592 -1.6293190 0.3294514<br>H 3.1311592 -0.1603990 1.8024514<br>H -1.1858018 2.2335400 -0.9387786<br>O 1.3814492 0.4574210 1.5630214<br>N 0.5597392 -0.4843700 1.3332614<br>O 0.9583002 -1.6837900 1.2540814<br>C -0.7614408 -0.1562200 1.2041114<br>C -2.3386608 -0.2981910 -1.6545886<br>C -1.9223508 -1.5736000 -1.1384586<br>C -0.5776898 -2.0713700 -1.3857686<br>O 0.4051392 -1.4211500 -1.7342586<br>O -0.5301498 -3.4007700 -1.1282986<br>C 0.7650102 -4.0125600 -1.1500286<br>H 0.5943302 -5.0676100 -0.9268786<br>H 1.4045202 -3.5661190 -0.3851486<br>H 1.2303102 -3.9124600 -2.1349186<br>O -1.6132208 0.6873100 -1.8636686

	O	-3.6775708	-0.2261710	-1.8180386
	C	-4.2058708	1.0501190	-2.2009386
	H	-5.2842808	0.9061390	-2.2816086
	H	-3.7925908	1.3732290	-3.1611986
	H	-3.9906908	1.8085890	-1.4418286
	H	0.3018092	0.7129800	-1.5506686
	C	0.7949182	2.5294400	-0.6834086
	C	1.8833382	3.3274310	-0.2615186
	N	1.0797092	1.3395700	-1.2848786
	C	2.3429092	0.8888610	-1.4711086
	H	2.4100692	-0.0830890	-1.9351886
	C	3.4236792	1.6364610	-1.0736286
	H	4.4288492	1.2538810	-1.2025186
	C	3.1735382	2.8748910	-0.4548886
	H	4.0066682	3.4847210	-0.1177386
	C	4.9519892	0.5448510	2.6164214
	H	4.3060592	0.9015210	3.4240314
	H	5.3790192	1.4093120	2.0908314
	H	5.7802892	-0.0262880	3.0464814
	C	-1.7516208	-1.1234600	0.9759814
	H	-0.9458408	0.9015700	1.2963414
	H	1.6906882	4.2773510	0.2201414
	C	3.9274202	-2.3207690	-1.2833686
	H	4.6447102	-1.7880390	-1.9129386
	H	2.9799902	-2.4185690	-1.8172886
	H	4.3362402	-3.3210090	-1.0851786
	C	-0.9435018	4.0985200	0.0906114
	H	-0.5911018	4.1822700	1.1253814
	H	-2.0354418	4.0987000	0.0976314
	H	-0.6021218	4.9776300	-0.4710186
	H	-2.6958598	-2.3292110	-1.0792586
	H	-1.4470598	-2.1501500	1.1545114
	C	-3.1711308	-0.8214410	1.2689514
	C	-5.8922008	-0.3351710	1.8082514
	C	-4.0491598	-1.8795210	1.5549214
	C	-3.6851508	0.4866690	1.2533614
	C	-5.0311308	0.7268290	1.5206814
	C	-5.3950208	-1.6403010	1.8251614
	H	-3.6674598	-2.8975910	1.5627314
	H	-3.0334608	1.3234290	1.0204814
	H	-5.4096408	1.7455990	1.5046714
	H	-6.0559398	-2.4731920	2.0493014
	H	-6.9416608	-0.1467520	2.0175114
	S	6.2720192	-0.8886280	0.2032714

Name	TS$_{Theo2S-BMA-S1}$
**Absolute Energies (Hartree)**	There are 14 positive frequencies below 100 cm^-1.<br><br>Zero-point correction: 0.531218 hartree<br>Enthalpy correction: 0.568835 hartree<br>Free Energy correction: 0.463035 hartree<br>Quasiharmonic Free Energy correction: 0.471516 hartree<br><br>SCF Energy: -1980.339082 hartree<br>SCF Energy*: -1980.822264 hartree<br>SCF Energy + ZPVE: -1979.807864 hartree<br>Enthalpy: -1979.770247 hartree<br>Free Energy: -1979.876047 hartree<br><br>Free Energy with quasiharmonic correction: -1979.867567 hartree (correction: 5.32 kcal/mol)<br><br>---<br>Low frequencies before projection:<br>-206.9715<br>18.9477<br>19.8237<br><br>Low frequencies after projection:<br>-206.9714<br>18.2616<br>19.7337<br>---
**Cartesian Coordinates**	N -3.2216717 -0.4758673 -1.6758842<br>C -4.1374917 -1.1306773 -0.9260842<br>N -3.6012517 -1.9874073 -0.0191342<br>N -2.4371817 1.0531827 1.5789758<br>O -0.8551317 -2.9158573 -0.6054142<br>N -0.1059217 -1.8969573 -0.5981042<br>O -0.6059617 -0.7293873 -0.4810342<br>C 1.2464983 -2.0484573 -0.7108242<br>C 0.9693283 -0.4784073 2.0241558<br>C 2.2844583 -0.2840883 1.4676458<br>C 2.7658583 1.0634817 1.2399658<br>O 2.0861383 2.0285517 0.8840758<br>O 4.1137783 1.1443917 1.3561158<br>C 4.6981483 2.4026117 0.9949758<br>O 0.1437783 0.4023927 2.3149058<br>O 0.6973483 -1.7888073 2.2107858<br>C -0.6484617 -2.1076073 2.5961858<br>C -2.1291317 1.9322527 0.6229058<br>C -3.0834117 2.6395827 -0.1563442<br>N -0.8081317 2.1716727 0.3633558<br>C -0.3793617 3.0231427 -0.5995742

	C	-1.2762117	3.7122327	-1.3754842
	C	-2.6535017	3.5079327	-1.1344542
	C	-4.3880517	-2.8369973	0.8527658
	C	2.0737083	-0.9238283	-0.6697142
	C	-3.5381417	0.3938227	-2.7891142
	C	-3.7930717	0.7645727	2.0189658
	C	3.4996583	-1.0014083	-1.0341942
	C	6.2190183	-1.0519283	-1.7495342
	C	4.2848983	-2.1397383	-0.7820442
	C	4.1045583	0.1118317	-1.6406742
	C	5.4509583	0.0866717	-2.0001242
	C	5.6309683	-2.1634283	-1.1376742
	H	-4.1639617	1.2373327	-2.4800042
	H	-2.5954917	0.7778227	-3.1888842
	H	-4.0696217	-0.1469173	-3.5807842
	H	-2.2409917	-0.5911273	-1.4126942
	H	-2.6286317	-2.2665673	-0.1705242
	H	-5.0468317	-2.2412073	1.4920258
	H	-3.6921317	-3.4001173	1.4821558
	H	-5.0145217	-3.5423773	0.2923158
	H	-0.6836417	-3.1957673	2.6603258
	H	-1.3522617	-1.7560573	1.8361658
	H	-0.8936317	-1.6628073	3.5658458
	H	-3.7391817	-0.0376773	2.7565458
	H	-4.4277517	0.4305927	1.1931058
	H	-4.2523117	1.6421427	2.4914658
	H	-1.6506017	0.5804727	2.0287358
	H	-0.0943117	1.6840827	0.9295058
	H	0.6969683	3.1010027	-0.6785642
	H	-0.9275607	4.3922427	-2.1428642
	H	-4.1377317	2.4635527	0.0134458
	H	-3.3899817	4.0355127	-1.7335042
	H	1.5845783	0.0206817	-0.8802542
	H	1.5800583	-3.0745283	-0.7341042
	H	3.8449383	-3.0014883	-0.2881942
	H	6.2257983	-3.0494383	-0.9327142
	H	7.2700683	-1.0736083	-2.0243942
	H	5.9001883	0.9561117	-2.4726842
	H	3.5084483	1.0014817	-1.8275142
	H	5.7768883	2.2588217	1.0763758
	H	4.3698683	3.1947017	1.6755158
	H	4.4326883	2.6811417	-0.0289842
	H	3.0208483	-1.0382683	1.7131958
	S	-5.8172017	-0.8572173	-1.0704342

Name	TS$_{Theo2S-BMA-S11}$
**Absolute Energies (Hartree)**	There are 14 positive frequencies below 100 cm^-1.<br><br>Zero-point correction: 0.530946 hartree<br>Enthalpy correction: 0.568654 hartree<br>Free Energy correction: 0.462060 hartree<br>Quasiharmonic Free Energy correction: 0.471110 hartree<br><br>SCF Energy: -1980.339346 hartree<br>SCF Energy*: -1980.822801 hartree<br>SCF Energy + ZPVE: -1979.808400 hartree<br>Enthalpy: -1979.770692 hartree<br>Free Energy: -1979.877286 hartree<br><br>Free Energy with quasiharmonic correction: -1979.868236 hartree (correction: 5.68 kcal/mol)<br><br>---<br>Low frequencies before projection:<br>-206.7323<br>15.4575<br>19.7028<br><br>Low frequencies after projection:<br>-206.7322<br>15.3773<br>19.5803<br>---
**Cartesian Coordinates**	N -3.6258006 -0.8312002 -1.4647905<br>C -4.4948206 -1.3281602 -0.5548105<br>N -3.9008106 -2.0633202 0.4262195<br>N -1.8676906 1.6410708 0.7742195<br>H -2.6317006 -0.9088992 -1.2418405<br>H -2.9576006 -2.4035102 0.2212295<br>H -1.2405706 1.0208208 1.3180395<br>O -1.2001096 -3.0554992 -0.3240205<br>N -0.4472506 -2.0428192 -0.3940505<br>O -0.9455306 -0.8686892 -0.3479105<br>C 0.9011194 -2.2138592 -0.5363805<br>C 0.8288894 -0.4294292 2.0829095<br>C 2.1691194 -0.5291192 1.5724495<br>C 2.9624194 0.6647208 1.3641595<br>O 2.5483894 1.7816908 1.0496395<br>O 4.2882494 0.4026308 1.4710395<br>C 5.1671894 1.4900218 1.1545895<br>H 6.1757594 1.0771318 1.2126295<br>H 4.9752094 1.8699018 0.1476095<br>H 5.0511894 2.3068318 1.8743795<br>O 0.1509594 0.6024508 2.2179595

	O	0.3351494	-1.6480192	2.4012395
	C	-1.0432606	-1.6943292	2.7963595
	H	-1.2398906	-2.7389392	3.0411695
	H	-1.2219006	-1.0593392	3.6693695
	H	-1.6852206	-1.3760892	1.9700595
	H	0.6057594	2.1511508	0.7366895
	C	-1.2763906	2.6559808	0.0797695
	N	0.0551394	2.7742108	0.1364995
	C	-4.6398006	-2.7968502	1.4376395
	H	-5.2924496	-3.5645702	1.0026595
	H	-3.9112496	-3.2792502	2.0958995
	H	-5.2645706	-2.1219102	2.0296195
	C	1.7582194	-1.1113492	-0.5604605
	H	1.2154604	-3.2464892	-0.5397505
	C	-4.0036606	-0.0373602	-2.6142205
	H	-4.7011406	-0.5831802	-3.2578505
	H	-4.4813706	0.9052298	-2.3224505
	H	-3.0935006	0.1842998	-3.1788205
	C	-3.2009506	1.4056398	0.7718695
	H	-3.5220206	0.5404198	1.3377995
	H	2.7100894	-1.4318392	1.8244995
	H	1.2848294	-0.1498792	-0.7309105
	C	3.1513694	-1.2266592	-1.0275405
	C	5.8093794	-1.3454382	-1.9385705
	C	3.9255094	-2.3825792	-0.8274105
	C	3.7356194	-0.1306692	-1.6841905
	C	5.0513694	-0.1906682	-2.1408905
	C	5.2411294	-2.4397982	-1.2789805
	H	3.5034404	-3.2320692	-0.2982305
	H	3.1507994	0.7739008	-1.8280605
	H	5.4847894	0.6649618	-2.6519305
	H	5.8281704	-3.3388082	-1.1117905
	H	6.8366694	-1.3935782	-2.2893805
	C	0.7952894	3.8051508	-0.5704605
	H	0.5218394	4.8110408	-0.2267005
	H	0.6282394	3.7459808	-1.6531305
	H	1.8522294	3.6369808	-0.3661505
	C	-2.1318006	3.5108908	-0.6645905
	H	-1.7038406	4.3310708	-1.2262305
	C	-3.4932706	3.2864298	-0.6582705
	H	-4.1404006	3.9441698	-1.2314805
	C	-4.0558306	2.2176698	0.0697895
	H	-5.1162506	2.0014198	0.0553695
	S	-6.1683306	-1.0097002	-0.6011205

Name	$TS_{Theo2S\text{-}BMA\text{-}S12}$
**Absolute Energies (Hartree)**	There are 14 positive frequencies below 100 cm^-1.<br><br>Zero-point correction: 0.531372 hartree<br>Enthalpy correction: 0.569020 hartree<br>Free Energy correction: 0.463452 hartree<br>Quasiharmonic Free Energy correction: 0.471460 hartree<br><br>SCF Energy: -1980.328152 hartree<br>SCF Energy*: -1980.812391 hartree<br>SCF Energy + ZPVE: -1979.796780 hartree<br>Enthalpy: -1979.759132 hartree<br>Free Energy: -1979.864700 hartree<br><br>Free Energy with quasiharmonic correction: -1979.856692 hartree (correction: 5.02 kcal/mol)<br><br>---<br>Low frequencies before projection:<br>-223.3907<br>21.5222<br>25.1294<br><br>Low frequencies after projection:<br>-223.3907<br>20.9961<br>25.025<br>---
**Cartesian Coordinates**	N -1.0169978 2.7400171 -0.4900697<br>C 0.3126722 2.6108471 -0.7988497<br>C 1.2084722 3.5230871 -0.1680097<br>N 0.6777922 1.6594071 -1.6480697<br>C 2.0680722 1.3675071 -1.9783197<br>N 4.3707922 -1.7130329 0.4881403<br>C 4.8414622 -0.4514429 0.6275703<br>N 3.9153922 0.4366071 1.0722603<br>N 0.9002222 -1.5382929 1.2141603<br>O 1.7672822 -2.3035029 1.7304003<br>O 1.2600922 -0.5289029 0.5202003<br>C -0.4239578 -1.7895029 1.4127203<br>C -1.3895678 -0.9685029 0.8095203<br>C -1.6678078 -1.6129329 -1.2422397<br>C -2.5053778 -0.5380229 -1.7198497<br>O -2.1275778 0.6078271 -2.0137297<br>O -3.8146278 -0.8637029 -1.7185397<br>C -4.7350778 0.1971771 -2.0111197<br>C -0.3177178 -1.7624129 -1.7844697<br>O 0.2549422 -1.0214429 -2.5766897<br>O 0.2615322 -2.8817129 -1.2907397

	C	1.6561722	-3.0423129	-1.6000897
	C	4.1817422	1.8361771	1.3241103
	C	5.1757122	-2.8393229	0.0608503
	C	-1.5049978	3.6755671	0.3696003
	H	-2.1765078	-2.5412229	-1.0115197
	H	4.9746122	1.9614771	2.0690603
	H	3.2605822	2.2860071	1.7058603
	H	4.4959322	2.3686171	0.4164303
	H	2.9416322	0.1239071	1.0769103
	H	3.4512722	-1.9210429	0.8871403
	H	5.5929322	-2.6715429	-0.9375597
	H	6.0109722	-3.0307729	0.7458603
	H	4.5271222	-3.7197229	0.0346503
	H	-5.7280678	-0.2431229	-1.9097497
	H	-0.6335578	-2.6934529	1.9641103
	H	-4.5871178	0.5720771	-3.0283497
	H	-4.6200678	1.0214571	-1.3014897
	H	-1.6635378	2.0861571	-0.9418297
	H	-0.0268678	0.9910471	-1.9721897
	H	2.0641622	0.5073871	-2.6439397
	H	2.2666222	3.4485571	-0.3801497
	H	-2.5784478	3.6573571	0.5141803
	H	1.8060522	-3.1536529	-2.6783197
	H	1.9652322	-3.9490529	-1.0785497
	H	2.2229622	-2.1800529	-1.2393497
	H	-1.0222078	0.0030071	0.4972603
	C	-2.7930378	-0.9859629	1.2763003
	C	-5.4787678	-0.9326329	2.1118603
	C	-3.4608278	-2.1745129	1.6129503
	C	-3.4952878	0.2260771	1.3632803
	C	-4.8255678	0.2558071	1.7810603
	C	-4.7903078	-2.1468029	2.0269203
	H	-2.9410878	-3.1249129	1.5291303
	H	-2.9833478	1.1507671	1.1076403
	H	-5.3514878	1.2047871	1.8481803
	H	-5.2946878	-3.0757929	2.2786703
	H	-6.5164878	-0.9151529	2.4333603
	C	-0.6646278	4.5582071	0.9897203
	C	0.7205022	4.4665771	0.7036203
	H	2.6398622	1.1070671	-1.0833997
	H	2.5370822	2.2195371	-2.4827297
	H	-1.0556778	5.2982471	1.6766103
	H	1.4129022	5.1522671	1.1828703
	S	6.4485022	-0.0061629	0.2667403

Name	$TS_{Theo2S-BMA-S13}$
**Absolute Energies (Hartree)**	There are 15 positive frequencies below 100 cm^-1.<br><br>Zero-point correction: 0.530521 hartree<br>Enthalpy correction: 0.568614 hartree<br>Free Energy correction: 0.459115 hartree<br>Quasiharmonic Free Energy correction: 0.470328 hartree<br><br>SCF Energy: -1980.334321 hartree<br>SCF Energy*: -1980.818524 hartree<br>SCF Energy + ZPVE: -1979.803800 hartree<br>Enthalpy: -1979.765707 hartree<br>Free Energy: -1979.875206 hartree<br><br>Free Energy with quasiharmonic correction: -1979.863993 hartree (correction: 7.04 kcal/mol)<br><br>---<br>Low frequencies before projection:<br>-199.1696<br>7.7297<br>16.5002<br><br>Low frequencies after projection:<br>-199.1695<br>5.1837<br>15.8102<br>---
**Cartesian Coordinates**	N -3.6667408 -0.8421708 -1.5610926<br>C -4.4990308 -1.3285108 -0.6103126<br>N -3.9022808 -2.1831008 0.2600074<br>N -1.1615908 1.9272292 0.6901174<br>H -2.6671208 -0.9835008 -1.4056326<br>H -2.9894008 -2.5543808 -0.0128926<br>H -0.3873808 1.4096492 1.1448874<br>O -1.2426708 -3.2412408 -0.5882226<br>N -0.4799608 -2.2334508 -0.5996526<br>O -0.9641508 -1.0571008 -0.5496426<br>C 0.8752792 -2.4129708 -0.6748926<br>C 0.6416592 -0.8765808 2.0641874<br>C 1.9800192 -0.7316208 1.5328774<br>C 2.5398492 0.5828992 1.3607274<br>O 1.9104992 1.6108892 1.0807574<br>O 3.8915292 0.5827592 1.4349674<br>C 4.5418292 1.8188092 1.1151474<br>H 5.6110792 1.6032192 1.1445674<br>H 4.2585292 2.1672992 0.1176574<br>H 4.2929992 2.5920592 1.8493174<br>O -0.1779008 0.0116192 2.3141474

	O	0.3442792	-2.1836808	2.2616974
	C	-1.0100508	-2.4611108	2.6486474
	H	-1.0861208	-3.5488408	2.6822074
	H	-1.2312908	-2.0330108	3.6312174
	H	-1.7071608	-2.0535708	1.9125774
	H	1.0363992	3.0555892	0.4898174
	C	-0.9535008	3.1513692	0.1260174
	N	0.2912592	3.6525392	0.1318074
	C	-4.6039308	-2.8814208	1.3198574
	H	-5.3864908	-3.5446908	0.9306574
	H	-3.8692308	-3.4792308	1.8670674
	H	-5.0749108	-2.1744308	2.0095874
	C	1.7270792	-1.3093208	-0.6256926
	H	1.1856292	-3.4465508	-0.6704726
	C	-4.0684408	0.0499092	-2.6271826
	H	-4.8682608	-0.3929708	-3.2291526
	H	-4.4280208	1.0147692	-2.2490926
	H	-3.1954308	0.2218592	-3.2633826
	C	-2.3765508	1.3260592	0.7419474
	H	-2.3735908	0.3485192	1.2025574
	H	2.6724092	-1.5315108	1.7603474
	H	1.2565492	-0.3509708	-0.8164526
	C	3.1493492	-1.4097908	-0.9986026
	C	5.8616592	-1.5038508	-1.7376426
	C	3.9148692	-2.5653408	-0.7659426
	C	3.7701492	-0.3019808	-1.5990526
	C	5.1128592	-0.3487908	-1.9704526
	C	5.2574392	-2.6103508	-1.1325426
	H	3.4623992	-3.4246708	-0.2793326
	H	3.1889892	0.6001992	-1.7733526
	H	5.5742092	0.5163192	-2.4394426
	H	5.8367892	-3.5097508	-0.9420826
	H	6.9096992	-1.5426208	-2.0220126
	C	0.6418092	4.9390892	-0.4417526
	H	0.0986092	5.7573192	0.0461774
	H	0.4389892	4.9753492	-1.5199726
	H	1.7106092	5.0960092	-0.2854526
	C	-2.0800208	3.8131192	-0.4237426
	H	-1.9518608	4.7841492	-0.8845826
	C	-3.3210708	3.2097892	-0.3658126
	H	-4.1797508	3.7217892	-0.7907426
	C	-3.4892008	1.9426292	0.2255774
	H	-4.4515408	1.4449192	0.2557874
	S	-6.1406508	-0.8746408	-0.4966326

Name	$TS_{Theo2S\text{-}BMA\text{-}S2}$
**Absolute Energies (Hartree)**	There are 14 positive frequencies below 100 cm^-1.<br><br>Zero-point correction: 0.531049 hartree<br>Enthalpy correction: 0.568918 hartree<br>Free Energy correction: 0.461415 hartree<br>Quasiharmonic Free Energy correction: 0.470853 hartree<br><br>SCF Energy: -1980.330926 hartree<br>SCF Energy*: -1980.815986 hartree<br>SCF Energy + ZPVE: -1979.799877 hartree<br>Enthalpy: -1979.762008 hartree<br>Free Energy: -1979.869511 hartree<br><br>Free Energy with quasiharmonic correction: -1979.860073 hartree (correction: 5.92 kcal/mol)<br><br>---<br>Low frequencies before projection:<br>-350.7330<br>14.1279<br>21.1119<br><br>Low frequencies after projection:<br>-350.733<br>12.2114<br>20.9127<br>---
**Cartesian Coordinates**	N 4.0899308 0.0704192 -1.1180271<br>C 3.9357108 1.3152892 -0.6552671<br>C 3.4121928 3.8913802 0.3347029<br>C 5.0074818 2.1652692 -0.2745971<br>N 2.6682118 1.8038702 -0.5121471<br>C 2.3983318 3.0453002 -0.0382771<br>C 4.7387518 3.4276592 0.2093929<br>N -5.7672292 0.4255842 0.8688129<br>C -5.7190092 0.4176942 -0.4852771<br>N -4.8960192 -0.5172358 -1.0116671<br>N -2.7897002 -1.3188668 1.6930229<br>O -3.6331402 -0.7396568 2.4447229<br>O -3.1755902 -2.0667068 0.7357129<br>C -1.4622002 -1.1251078 1.9087929<br>C -0.5045602 -1.6705278 1.0164329<br>C -0.7385002 -0.7871178 -0.8088371<br>C 0.4499098 -1.1859788 -1.5289371<br>O 1.4672408 -0.5087788 -1.7218271<br>O 0.3755798 -2.4733288 -1.9317071<br>C 1.5466588 -3.0096388 -2.5654771<br>C -0.9427692 0.6174422 -0.4731171

	O	-0.0818782	1.4874812	-0.3407071
	O	-2.2383392	0.8403522	-0.1931771
	C	-2.5814382	2.1178032	0.3673729
	C	-4.5106092	-0.5715268	-2.4055771
	C	-6.6179892	1.2978252	1.6520729
	C	5.3730608	-0.5995408	-1.2229671
	H	-1.6450402	-1.3283078	-1.0531071
	H	-5.3880602	-0.6485658	-3.0542971
	H	-3.9393992	0.3160832	-2.7084671
	H	-3.8868102	-1.4597268	-2.5441771
	H	-6.4578192	1.0570242	2.7069429
	H	-7.6757392	1.1503452	1.4077429
	H	-6.3832582	2.3566542	1.4868229
	H	1.2823488	-4.0287888	-2.8513871
	H	-1.2340092	-0.4079978	2.6826629
	H	6.0392208	-0.0891418	-1.9288071
	H	5.1947498	-1.6119008	-1.5884871
	H	1.8150898	-2.4261498	-3.4509571
	H	2.3904488	-3.0243898	-1.8680971
	H	3.2406608	-0.4443098	-1.3555271
	H	1.8542408	1.2086502	-0.7496671
	H	1.3441518	3.2797512	0.0241429
	H	3.1873328	4.8804902	0.7136829
	H	5.5644728	4.0713692	0.4982529
	H	6.0249918	1.8089082	-0.3704171
	H	5.8707398	-0.6675318	-0.2481071
	H	-2.4569682	2.9096622	-0.3771271
	H	-3.6269382	2.0309732	0.6592729
	H	-1.9570182	2.3337422	1.2391929
	H	-0.7676002	-2.6341378	0.5889229
	C	0.9355698	-1.5213788	1.3494629
	C	3.6760398	-1.2968808	1.9536829
	C	1.4535108	-0.3375188	1.9008429
	C	1.8198498	-2.5801298	1.0838329
	C	3.1775198	-2.4717998	1.3862329
	C	2.8093208	-0.2303298	2.2059829
	H	0.7951608	0.5074712	2.0716929
	H	1.4324388	-3.4967388	0.6468429
	H	3.8431888	-3.3077608	1.1868029
	H	3.1913308	0.6915302	2.6366829
	H	4.7318098	-1.2122408	2.1977929
	H	-5.1083992	-0.1628258	1.3880229
	H	-4.3329602	-1.0751768	-0.3645771
	S	-6.6182582	1.5117152	-1.4457571

Name	$TS_{Theo2S-BMA-S21}$
**Absolute Energies (Hartree)**	There are 14 positive frequencies below 100 cm^-1.<br><br>Zero-point correction: 0.530959 hartree<br>Enthalpy correction: 0.568813 hartree<br>Free Energy correction: 0.461739 hartree<br>Quasiharmonic Free Energy correction: 0.470799 hartree<br><br>SCF Energy: -1980.328609 hartree<br>SCF Energy*: -1980.813130 hartree<br>SCF Energy + ZPVE: -1979.797650 hartree<br>Enthalpy: -1979.759796 hartree<br>Free Energy: -1979.866870 hartree<br><br>Free Energy with quasiharmonic correction: -1979.857810 hartree (correction: 5.69 kcal/mol)<br><br>---<br>Low frequencies before projection:<br>-351.4267<br>20.7935<br>22.0425<br><br>Low frequencies after projection:<br>-351.4267<br>20.4149<br>21.8468<br>---
**Cartesian Coordinates**	N 5.0650602 0.3506067 0.9048408<br>C 6.2634402 0.2588967 0.2709208<br>N 6.2339202 -0.4691833 -0.8670792<br>N -4.1396498 0.4566087 0.7896408<br>O 3.5873802 -0.8168623 -2.0162092<br>N 2.7675202 -1.2614523 -1.1537192<br>O 3.1812402 -1.7274123 -0.0440292<br>C 1.4359602 -1.2179723 -1.4233792<br>C -0.6636998 -0.6365823 1.8524408<br>C 0.5717402 -0.3752423 1.1505808<br>C 0.7942502 0.9263977 0.5275508<br>O -0.0505598 1.7075277 0.0948308<br>O 2.1137502 1.1344577 0.3612408<br>C 2.5092902 2.2526977 -0.4491992<br>O -1.6920198 0.0517477 1.8332508<br>O -0.6135398 -1.8098023 2.5213308<br>C -1.8234398 -2.2213423 3.1734908<br>C -4.0061298 1.5802987 0.0226008<br>N -2.7929398 2.1099687 -0.1239992<br>C 7.3700802 -0.6566233 -1.7444692<br>C 0.4896002 -1.6357123 -0.4535692

	C	4.8721102	0.9918067	2.1893908
	C	-0.9391298	-1.6896823	-0.8602392
	C	-3.6402798	-1.8698413	-1.6368092
	C	-1.5201798	-0.6983923	-1.6692792
	C	-1.7441598	-2.7538323	-0.4217092
	C	-3.0822198	-2.8458013	-0.8078492
	C	-2.8541398	-0.7954313	-2.0616392
	H	5.2290602	2.0255767	2.1645808
	H	3.7997402	0.9896977	2.3996908
	H	5.4041402	0.4738567	2.9988808
	H	4.3326602	-0.2960133	0.6050908
	H	5.3169202	-0.7564033	-1.2227392
	H	7.7490002	0.2957667	-2.1366292
	H	7.0437002	-1.2830533	-2.5798592
	H	8.1956002	-1.1556933	-1.2254792
	H	-1.5763898	-3.1515923	3.6872008
	H	-2.6152398	-2.4004813	2.4387808
	H	-2.1561298	-1.4687113	3.8943508
	H	-3.2816398	0.0875287	1.2335908
	H	-1.9705498	1.6464677	0.2802408
	H	0.8068202	-2.4620223	0.1766108
	H	1.1815802	-0.7269323	-2.3503092
	H	-0.9282598	0.1572177	-1.9762892
	H	-3.2842098	-0.0264513	-2.6977192
	H	-4.6798798	-1.9412913	-1.9448592
	H	-3.6832108	-3.6872813	-0.4724992
	H	-1.3096008	-3.5199523	0.2144508
	H	3.5933502	2.1780377	-0.5248492
	H	2.2180712	3.1949877	0.0246208
	H	2.0510602	2.1851477	-1.4398492
	H	1.4608902	-0.8238123	1.5777608
	C	-2.5195188	3.2732487	-0.9536292
	H	-2.8034488	3.0977387	-1.9988892
	H	-1.4463388	3.4534377	-0.9074892
	H	-3.0477188	4.1605187	-0.5858392
	C	-5.3186098	-0.1839513	0.9838508
	H	-5.2691098	-1.0655513	1.6112208
	C	-6.4718998	0.2772187	0.4053308
	H	-7.4107898	-0.2385613	0.5632408
	C	-6.3872698	1.4399887	-0.3945192
	H	-7.2824698	1.8313387	-0.8688792
	C	-5.1878098	2.0866387	-0.5883792
	H	-5.1264788	2.9751887	-1.2030092
	S	7.6807202	1.0183067	0.8532908

Name	$TS_{Theo2S-BMA-S22}$
**Absolute Energies (Hartree)**	There are 15 positive frequencies below 100 cm^-1.<br><br>Zero-point correction: 0.530766 hartree<br>Enthalpy correction: 0.568723 hartree<br>Free Energy correction: 0.459850 hartree<br>Quasiharmonic Free Energy correction: 0.471086 hartree<br><br>SCF Energy: -1980.327170 hartree<br>SCF Energy*: -1980.813687 hartree<br>SCF Energy + ZPVE: -1979.796404 hartree<br>Enthalpy: -1979.758447 hartree<br>Free Energy: -1979.867320 hartree<br><br>Free Energy with quasiharmonic correction: -1979.856084 hartree (correction: 7.05 kcal/mol)<br><br>--<br>Low frequencies before projection:<br>-318.6222<br>13.6183<br>17.9772<br><br>Low frequencies after projection:<br>-318.6222<br>10.2724<br>17.5208<br>--
**Cartesian Coordinates**	N -4.7007008 -0.5338817 -0.7084235<br>C -5.1654218 0.7145683 -0.4692335<br>N -5.1747818 1.0760783 0.8355165<br>N 3.1443382 0.2441783 -1.3850135<br>O -3.4897608 -0.2384417 2.7758565<br>N -2.7143608 -0.9922417 2.1105265<br>O -3.1861208 -1.9148917 1.3679465<br>C -1.3715108 -0.7932617 2.1792065<br>C 0.1069992 -2.0070817 -1.3100535<br>C -0.8617808 -1.1835317 -0.6035235<br>C -0.7912018 0.2574283 -0.6886635<br>O 0.1872782 0.9391483 -1.0245335<br>O -1.9325018 0.8191583 -0.2563635<br>C -1.9787218 2.2516283 -0.1893535<br>O 1.1718792 -1.6620617 -1.8162535<br>O -0.2772708 -3.3088817 -1.2913335<br>C 0.6365092 -4.2361117 -1.8959835<br>C 3.4621782 1.4372983 -0.8897335<br>C 4.7932682 1.9141783 -0.7200035<br>N 2.4527682 2.2684683 -0.4877535<br>C 2.6594082 3.4975683 0.0484565

	C	3.9288882	3.9802183	0.2276965
	C	5.0084582	3.1578083	-0.1720235
	C	-5.7055618	2.3276483	1.3345565
	C	-0.4849808	-1.5685017	1.3950065
	C	-4.3770008	-1.0364317	-2.0280535
	C	4.1325492	-0.7513317	-1.7679835
	C	0.9759292	-1.3719117	1.5614365
	C	3.7583692	-1.0712617	1.8839565
	C	1.5331592	-0.1191317	1.8716965
	C	1.8437492	-2.4629417	1.3889865
	C	3.2208192	-2.3165517	1.5504165
	C	2.9084492	0.0266983	2.0412165
	H	-3.5744708	-0.4555917	-2.5020835
	H	-4.0492208	-2.0743517	-1.9186335
	H	-5.2511708	-1.0062017	-2.6847035
	H	-4.2945308	-1.0471217	0.0774665
	H	-4.7260318	0.4631583	1.5223265
	H	-5.1849918	3.1939883	0.9077365
	H	-5.5763018	2.3323383	2.4205865
	H	-6.7705018	2.4349473	1.0996265
	H	0.1840592	-5.2204017	-1.7645235
	H	1.6138892	-4.1990417	-1.4050135
	H	0.7646692	-4.0188417	-2.9608735
	H	3.5847392	-1.6380717	-2.0830435
	H	4.7755992	-1.0170117	-0.9210235
	H	4.7550492	-0.3988717	-2.5989135
	H	2.1637392	-0.0584717	-1.4267435
	H	1.4860382	1.9196883	-0.6020735
	H	1.7616082	4.0429883	0.3149365
	H	4.0879582	4.9610783	0.6578965
	H	5.6209682	1.2867683	-1.0239635
	H	6.0272982	3.5119783	-0.0450035
	H	-0.7906208	-2.5974117	1.2265165
	H	-1.0831608	0.0759683	2.7498065
	H	0.8869482	0.7464683	1.9788765
	H	3.3195282	1.0010983	2.2913265
	H	4.8304592	-0.9558817	2.0199965
	H	3.8729292	-3.1764417	1.4224465
	H	1.4269492	-3.4340717	1.1347465
	H	-2.9688818	2.4867983	0.1961365
	H	-1.8465018	2.6886583	-1.1827235
	H	-1.2055118	2.6314283	0.4876465
	H	-1.8649308	-1.5882317	-0.5432435
	S	-5.6889218	1.7650383	-1.7137035

Name	$TS_{Theo2S-BMA-S23}$
**Absolute Energies (Hartree)**	There are 15 positive frequencies below 100 cm^-1.<br><br>Zero-point correction: 0.530877 hartree<br>Enthalpy correction: 0.568814 hartree<br>Free Energy correction: 0.461542 hartree<br>Quasiharmonic Free Energy correction: 0.470820 hartree<br><br>SCF Energy: -1980.330375 hartree<br>SCF Energy*: -1980.816138 hartree<br>SCF Energy + ZPVE: -1979.799498 hartree<br>Enthalpy: -1979.761561 hartree<br>Free Energy: -1979.868833 hartree<br><br>Free Energy with quasiharmonic correction: -1979.859556 hartree (correction: 5.82 kcal/mol)<br><br>---<br>Low frequencies before projection:<br>-325.8378<br>22.3392<br>23.7276<br><br>Low frequencies after projection:<br>-325.8377<br>22.3134<br>23.5857<br>---
**Cartesian Coordinates**	N -4.6412651 -0.7644253 -0.8954926<br>C -5.1950851 0.4245847 -0.5618226<br>N -5.2670651 0.6631447 0.7688674<br>N 2.9904939 0.7671177 -1.0565426<br>O -3.5384751 -0.6934153 2.6362374<br>N -2.6978551 -1.3373043 1.9353574<br>O -3.0887341 -2.2175143 1.0996074<br>C -1.3728251 -1.0664943 2.0687074<br>C 0.3128249 -1.8565233 -1.4422626<br>C -0.7477451 -1.1822043 -0.7110826<br>C -0.7761851 0.2622457 -0.6569426<br>O 0.1786339 1.0303767 -0.8444626<br>O -1.9785461 0.7092357 -0.2640326<br>C -2.1216761 2.1227657 -0.0557026<br>O 1.3636249 -1.3746933 -1.8600126<br>O 0.0412459 -3.1799533 -1.5629526<br>C 1.0531959 -3.9693033 -2.2058326<br>C 3.2361239 2.0025377 -0.5285626<br>N 2.1929639 2.7555067 -0.1557126<br>C -5.8876761 1.8308247 1.3591774<br>C -0.4172151 -1.7202843 1.2541474

	C	-4.2450851	-1.1197253	-2.2428526
	C	1.0245049	-1.4608933	1.4900074
	C	3.7770849	-1.0393823	1.9207774
	C	1.5022949	-0.2097533	1.9173774
	C	1.9562659	-2.4869533	1.2598174
	C	3.3185259	-2.2805723	1.4732374
	C	2.8628249	-0.0064423	2.1411774
	H	-3.4776451	-0.4399753	-2.6363326
	H	-3.8417351	-2.1362453	-2.2146826
	H	-5.1010951	-1.0955453	-2.9232726
	H	-4.2205651	-1.3177853	-0.1451126
	H	-4.7913151	0.0241047	1.4125974
	H	-5.3993961	2.7613547	1.0433374
	H	-5.8051261	1.7357647	2.4456074
	H	-6.9457161	1.9045037	1.0847874
	H	0.6783759	-4.9939833	-2.1902426
	H	2.0022959	-3.9050633	-1.6646826
	H	1.2075259	-3.6400333	-3.2380326
	H	2.0119349	0.4409867	-1.1568126
	H	1.2617339	2.3643267	-0.3030826
	H	-0.6575541	-2.7464543	0.9896574
	H	-1.1545551	-0.2349543	2.7209074
	H	0.8082249	0.6104467	2.0693674
	H	3.2115539	0.9648577	2.4813874
	H	4.8376349	-0.8758223	2.0907774
	H	4.0209259	-3.0907523	1.2958274
	H	1.6016359	-3.4550533	0.9155174
	H	-3.1574861	2.2629057	0.2469174
	H	-1.9212761	2.6709157	-0.9802526
	H	-1.4431461	2.4652957	0.7330574
	H	-1.7203151	-1.6584043	-0.7421926
	C	2.3171129	4.0864367	0.4128974
	H	2.8717329	4.0699577	1.3592474
	H	1.3114629	4.4620667	0.6086874
	H	2.8160829	4.7766777	-0.2780726
	C	3.9710749	-0.0874423	-1.4342826
	H	3.6135949	-1.0334823	-1.8165726
	C	5.2934849	0.2612477	-1.3135826
	H	6.0704549	-0.4279713	-1.6199026
	C	5.5953139	1.5300187	-0.7791226
	H	6.6313339	1.8369187	-0.6683026
	C	4.5929639	2.3928077	-0.3877926
	H	4.8245939	3.3653877	0.0268574
	S	-5.7492961	1.5494847	-1.7253326

<table>
<tr><td>Name</td><td>TS_{Theo2S-BMA-S3}</td></tr>
<tr><td>Absolute Energies (Hartree)</td><td>There are 15 positive frequencies below 100 cm^-1.

Zero-point correction: 0.531327 hartree
Enthalpy correction: 0.569051 hartree
Free Energy correction: 0.462495 hartree
Quasiharmonic Free Energy correction: 0.471637 hartree

SCF Energy: -1980.338190 hartree
SCF Energy*: -1980.821635 hartree
SCF Energy + ZPVE: -1979.806863 hartree
Enthalpy: -1979.769139 hartree
Free Energy: -1979.875695 hartree

Free Energy with quasiharmonic correction: -1979.866554 hartree (correction: 5.74 kcal/mol)

Low frequencies before projection:
-226.3458
15.7820
22.0278

Low frequencies after projection:
-226.3455
14.8471
21.7216
---</td></tr>
<tr><td>Cartesian Coordinates</td><td>N 0.4991484 2.8587008 -0.5192986
C -0.7949006 2.5294508 -0.6833886
C -3.4236706 1.6364998 -1.0735786
C -1.8833116 3.3274608 -0.2614886
N -1.0797006 1.3395908 -1.2848486
C -2.3429006 0.8888908 -1.4710786
C -3.1735116 2.8749198 -0.4548486
N -4.1462906 -0.2834002 1.7434114
C -4.6240606 -0.9299602 0.6567514
N -3.6752506 -1.5769502 -0.0659086
N -0.5597506 -0.4843792 1.3332614
O -1.3814706 0.4574108 1.5630214
O -0.9583106 -1.6837992 1.2541014
C 0.7614194 -0.1562292 1.2041114
C 1.7516094 -1.1234592 0.9759814
C 1.9223294 -1.5736092 -1.1384586
C 2.3386494 -0.2982092 -1.6545886
O 1.6132194 0.6873008 -1.8636686
O 3.6775594 -0.2262082 -1.8180586
C 4.2058694 1.0500818 -2.2009786
C 0.5776694 -2.0713692 -1.3857686</td></tr>
</table>

	O	-0.4051706	-1.4211492	-1.7342286
	O	0.5301204	-3.4007792	-1.1282986
	C	-0.7650396	-4.0125592	-1.1500186
	C	-3.9273806	-2.3207102	-1.2834186
	C	-4.9520006	0.5447798	2.6164514
	C	0.9435384	4.0985308	0.0906014
	H	2.6958394	-2.3292292	-1.0792586
	H	-4.6446706	-1.7879702	-1.9129786
	H	-2.9799506	-2.4184902	-1.8173386
	H	-4.3361996	-3.3209602	-1.0852786
	H	-4.3061006	0.9013698	3.4241214
	H	-5.7803606	-0.0263402	3.0464114
	H	-5.3789406	1.4093098	2.0908814
	H	5.2842694	0.9060818	-2.2816886
	H	0.9458294	0.9015708	1.2963314
	H	0.6021584	4.9776408	-0.4710386
	H	2.0354784	4.0987008	0.0976214
	H	3.7925494	1.3732118	-3.1612086
	H	3.9907294	1.8085518	-1.4418386
	H	1.1858094	2.2335608	-0.9388186
	H	-0.3018006	0.7129908	-1.5506486
	H	-2.4100706	-0.0830492	-1.9351586
	H	-4.4288406	1.2539198	-1.2024686
	H	-4.0066316	3.4847598	-0.1176886
	H	-1.6906416	4.2773808	0.2201614
	H	0.5911384	4.1823008	1.1253714
	H	-1.2303496	-3.9124592	-2.1349086
	H	-0.5943696	-5.0676192	-0.9268686
	H	-1.4045496	-3.5661192	-0.3851386
	H	1.4470494	-2.1501492	1.1545214
	C	3.1711194	-0.8214492	1.2689514
	C	5.8921894	-0.3351682	1.8082514
	C	3.6851494	0.4866718	1.2533314
	C	4.0491394	-1.8795182	1.5549514
	C	5.3950094	-1.6402982	1.8251914
	C	5.0311194	0.7268218	1.5206514
	H	3.0334594	1.3234308	1.0204314
	H	3.6674394	-2.8975882	1.5627714
	H	6.0559194	-2.4731882	2.0493514
	H	5.4096294	1.7456018	1.5046114
	H	6.9416494	-0.1467482	2.0175014
	H	-3.1311606	-0.1604502	1.8024814
	H	-2.7344506	-1.6293302	0.3294414
	S	-6.2720006	-0.8886002	0.2032214

Name	$TS_{Theo2S\text{-}BMA\text{-}S31}$
**Absolute Energies (Hartree)**	There are 14 positive frequencies below 100 cm^-1.<br><br>Zero-point correction: 0.531625 hartree<br>Enthalpy correction: 0.569147 hartree<br>Free Energy correction: 0.464171 hartree<br>Quasiharmonic Free Energy correction: 0.472016 hartree<br><br>SCF Energy: -1980.332838 hartree<br>SCF Energy*: -1980.818032 hartree<br>SCF Energy + ZPVE: -1979.801213 hartree<br>Enthalpy: -1979.763691 hartree<br>Free Energy: -1979.868667 hartree<br><br>Free Energy with quasiharmonic correction: -1979.860822 hartree (correction: 4.92 kcal/mol)<br><br>---<br>Low frequencies before projection:<br>-234.9935<br>18.2653<br>35.9064<br><br>Low frequencies after projection:<br>-234.9929<br>17.7622<br>35.301<br>---
**Cartesian Coordinates**	N 3.5480347 -1.9799344 -0.5331532<br>C 4.4894547 -1.0888534 -0.9281532<br>N 3.9885147 0.0850266 -1.3822732<br>N 0.4729537 2.6316056 0.6851568<br>H 2.5610347 -1.7381944 -0.6587432<br>H 2.9710447 0.1859656 -1.4402432<br>H -0.4437863 2.2454946 0.9347768<br>O 1.1597347 0.5918556 -1.4663832<br>N 0.3103647 -0.2813144 -1.0989732<br>O 0.6808747 -1.4253244 -0.6924432<br>C -1.0117653 0.0401346 -1.1677132<br>C -2.4997453 0.3193246 1.5993568<br>C -2.3670153 -1.0700654 1.2465868<br>C -1.2054453 -1.8219754 1.7199868<br>O -0.2620053 -1.3971044 2.3749868<br>O -1.2887153 -3.1179454 1.3319868<br>C -0.1198243 -3.9066044 1.6021668<br>H -0.3154143 -4.8846044 1.1587268<br>H 0.7580557 -3.4454844 1.1415268<br>H 0.0466457 -4.0069044 2.6792368<br>O -1.5690063 1.1146046 1.7909968

	O	-3.7870653	0.7329446	1.5904568
	C	-4.0029663	2.1385646	1.7690468
	H	-5.0829863	2.2788936	1.7044068
	H	-3.6349463	2.4703346	2.7447068
	H	-3.5046563	2.7177946	0.9845668
	H	0.4271947	0.4649456	1.9464068
	C	1.5694437	1.9217456	1.0899468
	N	1.3807147	0.8098656	1.7942068
	C	4.7893437	1.1365566	-1.9740432
	H	4.1116837	1.9419666	-2.2699932
	H	5.5262337	1.5282566	-1.2637632
	H	5.3358447	0.7854466	-2.8577732
	C	-2.0141653	-0.9044954	-0.8714832
	H	-1.1973353	1.0466446	-1.5078732
	C	3.8425757	-3.2877244	0.0160068
	H	4.4422557	-3.8896934	-0.6757432
	H	4.3934347	-3.2146934	0.9610768
	H	2.8906057	-3.7949544	0.1966268
	C	0.5455137	3.7613056	-0.0651232
	H	-0.4070163	4.2063646	-0.3270432
	H	-3.2870753	-1.6368554	1.1725668
	H	-1.6855453	-1.9390554	-0.8771132
	C	-3.3988953	-0.6812354	-1.3462332
	C	-6.0448453	-0.3486464	-2.2488632
	C	-4.2063953	-1.7939154	-1.6351332
	C	-3.9477453	0.6023346	-1.5064532
	C	-5.2561953	0.7666836	-1.9554032
	C	-5.5149053	-1.6307364	-2.0858532
	H	-3.7979153	-2.7931154	-1.5041732
	H	-3.3536263	1.4794846	-1.2684632
	H	-5.6631363	1.7675636	-2.0734732
	H	-6.1204453	-2.5045164	-2.3109532
	H	-7.0652053	-0.2192864	-2.5993932
	C	2.8451437	2.4380056	0.7226168
	H	3.7347437	1.8972956	1.0153168
	C	2.9237737	3.5884456	-0.0236332
	H	3.9001537	3.9696556	-0.3069932
	C	1.7566737	4.2760756	-0.4379332
	H	1.8092137	5.1794456	-1.0326232
	C	2.4708747	-0.0548944	2.2241368
	H	3.0441247	-0.4245444	1.3695168
	H	2.0229747	-0.9054944	2.7336768
	H	3.1422647	0.4694456	2.9138168
	S	6.1642947	-1.4019134	-0.8209832

Name	$TS_{Theo2S-BMA-S32}$
**Absolute Energies (Hartree)**	There are 15 positive frequencies below 100 cm^-1.<br><br>Zero-point correction: 0.531100 hartree<br>Enthalpy correction: 0.568810 hartree<br>Free Energy correction: 0.462399 hartree<br>Quasiharmonic Free Energy correction: 0.471277 hartree<br><br>SCF Energy: -1980.340240 hartree<br>SCF Energy*: -1980.825063 hartree<br>SCF Energy + ZPVE: -1979.809140 hartree<br>Enthalpy: -1979.771430 hartree<br>Free Energy: -1979.877841 hartree<br><br>Free Energy with quasiharmonic correction: -1979.868963 hartree (correction: 5.57 kcal/mol)<br><br>---<br>Low frequencies before projection:<br>-199.7538<br>17.5458<br>24.3211<br><br>Low frequencies after projection:<br>-199.7537<br>16.3965<br>24.1839<br>---
**Cartesian Coordinates**	N 3.5714718 -1.9817977 0.1061435<br>C 4.4692118 -1.3690777 -0.7098065<br>N 3.9181918 -0.5375177 -1.6196765<br>N 0.3452318 2.4325523 0.2915735<br>H 2.5791718 -1.8675777 -0.1128165<br>H 2.9055918 -0.3925777 -1.5976865<br>H -0.3069982 1.8436223 0.8194835<br>O 1.0993618 0.1447723 -1.5934365<br>N 0.2825918 -0.6176277 -0.9935665<br>O 0.6908218 -1.6048077 -0.3095665<br>C -1.0567882 -0.3646177 -1.1308665<br>C -2.9725682 0.7392223 1.3198135<br>C -2.3961982 -0.5627877 1.5811235<br>C -1.0584282 -0.6706577 2.0854735<br>O -0.2131882 0.2374523 2.1066435<br>O -0.7712082 -1.9147177 2.5309435<br>C 0.5970518 -2.1422177 2.8900935<br>H 0.6621518 -3.2019077 3.1432435<br>H 1.2499818 -1.9128077 2.0442835<br>H 0.8840518 -1.5341877 3.7547235<br>O -2.3738082 1.7608423 0.9848835

	O	-4.3276482	0.7024423	1.4057935
	C	-5.0063682	1.9152323	1.0581435
	H	-6.0710782	1.6810923	1.1116235
	H	-4.7598982	2.7161323	1.7629435
	H	-4.7448182	2.2389023	0.0468535
	H	1.3863918	0.6722923	1.7012535
	C	1.6604618	2.2265923	0.3944235
	C	2.6475818	2.9613423	-0.3170065
	N	2.1077218	1.2485823	1.2379535
	C	3.4209218	0.9693323	1.4358035
	H	3.6262118	0.1534923	2.1163335
	C	4.3943918	1.6774623	0.7828635
	H	5.4376518	1.4272623	0.9232935
	C	3.9814918	2.6862923	-0.1149865
	H	4.7285018	3.2522023	-0.6637265
	C	4.6732518	0.2527323	-2.5685065
	H	3.9591318	0.8368623	-3.1556965
	H	5.3679818	0.9357723	-2.0660365
	H	5.2583718	-0.3819477	-3.2430665
	C	-2.0181282	-1.1974677	-0.5565265
	H	-1.2694282	0.5102323	-1.7236865
	H	2.3349118	3.7317023	-1.0094465
	C	3.9239118	-3.0249177	1.0504235
	H	4.6012918	-2.6492877	1.8250335
	H	3.0019018	-3.3766277	1.5213235
	H	4.4184418	-3.8721277	0.5604335
	C	-0.2379982	3.2957023	-0.7200065
	H	0.0502118	2.9701623	-1.7274265
	H	-1.3199482	3.2263523	-0.6141365
	H	0.0624218	4.3412323	-0.5812465
	H	-3.0810582	-1.3570077	1.8495335
	H	-1.6523482	-2.1429377	-0.1709465
	C	-3.4177582	-1.1889477	-1.0194165
	C	-6.1013082	-1.2617177	-1.8644165
	C	-4.2062682	-2.3341277	-0.8154465
	C	-4.0026182	-0.0760777	-1.6492165
	C	-5.3305082	-0.1140477	-2.0676565
	C	-5.5334882	-2.3727277	-1.2360865
	H	-3.7683482	-3.1981277	-0.3214765
	H	-3.4260482	0.8320723	-1.7930665
	H	-5.7664282	0.7560223	-2.5514465
	H	-6.1249982	-3.2695277	-1.0729865
	H	-7.1368682	-1.2892477	-2.1923165
	S	6.1545018	-1.5942077	-0.5590565

Name	$TS_{Theo2S-BMA-S33}$
**Absolute Energies (Hartree)**	There are 15 positive frequencies below 100 cm^-1.<br><br>Zero-point correction: 0.530874 hartree<br>Enthalpy correction: 0.568716 hartree<br>Free Energy correction: 0.461941 hartree<br>Quasiharmonic Free Energy correction: 0.470780 hartree<br><br>SCF Energy: -1980.340601 hartree<br>SCF Energy*: -1980.825624 hartree<br>SCF Energy + ZPVE: -1979.809727 hartree<br>Enthalpy: -1979.771885 hartree<br>Free Energy: -1979.878660 hartree<br><br>Free Energy with quasiharmonic correction: -1979.869822 hartree (correction: 5.55 kcal/mol)<br><br>---<br>Low frequencies before projection:<br>-196.7964<br>15.2068<br>23.5843<br><br>Low frequencies after projection:<br>-196.7961<br>15.0754<br>23.523<br>---
**Cartesian Coordinates**	N -2.3669848 0.8444552 -1.5127078<br>C -1.9492748 1.9117152 -0.8223378<br>C -0.8926038 4.0370152 0.6782122<br>C -2.8176338 2.8627952 -0.2241278<br>N -0.6058048 2.1052742 -0.6663778<br>C -0.0803138 3.1164942 0.0671622<br>C -2.2884538 3.8978052 0.5149922<br>N -3.8904748 -0.1135438 1.5081622<br>C -4.4102058 -1.2076338 0.9088822<br>N -3.4940058 -2.0442248 0.3706922<br>N -0.2304458 -0.5289058 1.1870122<br>O -1.0471848 0.3163652 1.6641222<br>O -0.6359658 -1.6229558 0.6888422<br>C 1.1065952 -0.2409258 1.2569422<br>C 2.0673842 -1.1352758 0.7817322<br>C 2.3506142 -0.8057868 -1.4353678<br>C 1.0100742 -1.0567858 -1.8844978<br>O 0.1195052 -0.2051558 -2.0274378<br>O 0.7748942 -2.3654658 -2.1238478<br>C -0.5914658 -2.7048058 -2.4100478<br>C 2.8514252 0.5500932 -1.3827678

	O	2.1905852	1.5749542	-1.2058078
	O	4.2059752	0.5816932	-1.4712678
	C	4.8135752	1.8702732	-1.3017778
	C	-3.8103458	-3.2933838	-0.2942878
	C	-4.6669448	0.9075562	2.1826622
	C	-3.7530848	0.6084362	-1.8851978
	H	3.0741942	-1.5942268	-1.5980178
	H	-2.8667768	-3.7694648	-0.5731078
	H	-4.4061658	-3.1285538	-1.1993678
	H	-4.3713868	-3.9711938	0.3593622
	H	-2.5028358	-1.8362748	0.5220622
	H	-2.8708148	-0.0226948	1.5450622
	H	-5.2310548	0.4978262	3.0289422
	H	-3.9691948	1.6631562	2.5523622
	H	-5.3810548	1.3855662	1.5028522
	H	-0.6161768	-3.7940258	-2.4737978
	H	1.3171552	0.7222942	1.6934822
	H	-4.1139148	1.3740962	-2.5831878
	H	-3.8059748	-0.3660238	-2.3723578
	H	-0.9132958	-2.2680348	-3.3611378
	H	-1.2435058	-2.3545548	-1.6063578
	H	-1.6498348	0.2007352	-1.8470278
	H	0.0532952	1.4369442	-1.1024478
	H	1.0007062	3.1249242	0.1087522
	H	-0.4651038	4.8437342	1.2608022
	H	-2.9600238	4.6129452	0.9811222
	H	-3.8871538	2.7493562	-0.3422878
	H	-4.4061548	0.5891262	-1.0094678
	H	4.5255162	2.5469032	-2.1128778
	H	5.8900652	1.6925622	-1.3261778
	H	4.5299752	2.3176432	-0.3451678
	H	1.7117542	-2.1330458	0.5494422
	C	3.4810842	-1.0402268	1.1877522
	C	6.1944142	-0.9577478	1.9309422
	C	4.0609352	0.1527832	1.6549322
	C	4.2880842	-2.1877368	1.0952022
	C	5.6306142	-2.1490678	1.4654922
	C	5.4042452	0.1916822	2.0229422
	H	3.4683252	1.0599132	1.7166822
	H	3.8526442	-3.1136068	0.7269522
	H	6.2370742	-3.0477078	1.3910522
	H	5.8364152	1.1227222	2.3798422
	H	7.2418042	-0.9247678	2.2185522
	S	-6.0973158	-1.4860338	0.7966422

Name	$TS_{Theo1S-BMA-R1}$
Absolute Energies (Hartree)	There are 13 positive frequencies below 100 cm^-1.<br><br>Zero-point correction: 0.519874 hartree<br>Enthalpy correction: 0.556007 hartree<br>Free Energy correction: 0.453221 hartree<br>Quasiharmonic Free Energy correction: 0.461516 hartree<br><br>SCF Energy: -1811.838558 hartree<br>SCF Energy*: -1812.282065 hartree<br>SCF Energy + ZPVE: -1811.318684 hartree<br>Enthalpy: -1811.282551 hartree<br>Free Energy: -1811.385337 hartree<br><br>Free Energy with quasiharmonic correction: -1811.377043 hartree (correction: 5.20 kcal/mol)<br><br>---<br>Low frequencies before projection:<br>-322.0715<br>17.5193<br>24.6286<br><br>Low frequencies after projection:<br>-322.0713<br>17.2392<br>24.4857<br>---
Cartesian Coordinates	N 2.8060030 2.2530813 -0.4851300<br>N -5.1520780 -0.6575557 -0.7128300<br>C -5.0809680 0.1476143 0.3740500<br>N -4.2384080 -0.2840667 1.3403300<br>N -2.1560190 -2.4671467 -0.4658700<br>O -3.0686690 -2.5710367 -1.3415800<br>O -2.4400490 -2.4939067 0.7760700<br>C -0.8652790 -2.3084977 -0.8638200<br>C 0.1551710 -2.0782677 0.0888700<br>C -0.2228580 -0.2684977 1.0225900<br>C -0.5198880 0.6032623 -0.0990100<br>O 0.2714430 1.3017223 -0.7413000<br>O -1.8081280 0.4712633 -0.4636200<br>C -2.2188470 1.1191433 -1.6770500<br>C 0.9597920 -0.0412487 1.8295400<br>O 1.9126420 0.6992013 1.5806000<br>O 0.9546520 -0.8344887 2.9292500<br>C 2.1240020 -0.7576787 3.7583200<br>C -3.8242780 0.5161433 2.4734000<br>C -6.0309080 -0.4337357 -1.8422800<br>C 2.3404940 3.4932913 0.2021100

	H -1.0914380 -0.6457477 1.5490100<br>H -4.6875180 0.8447143 3.0594100<br>H -3.1818780 -0.1057867 3.1039800<br>H -3.2641670 1.4085133 2.1626600<br>H -3.6668580 -1.1078567 1.1369700<br>H -4.5035880 -1.4470557 -0.7866200<br>H -7.0840480 -0.4354047 -1.5393800<br>H -5.8635380 -1.2444357 -2.5571500<br>H -5.8287780 0.5257143 -2.3340700<br>H -3.2356280 0.7722333 -1.8547400<br>H -0.7402490 -2.2036477 -1.9306900<br>H 3.0212940 4.3141213 -0.0342800<br>H 2.3225230 3.3005013 1.2747100<br>H -2.2037570 2.2064033 -1.5561600<br>H -1.5645880 0.8349223 -2.5065800<br>H 2.1057530 1.5285113 -0.2106000<br>H 1.3310240 3.7155913 -0.1448300<br>H 2.2353720 0.2448813 4.1833500<br>H 1.9660220 -1.4867587 4.5547600<br>H 3.0238720 -1.0059187 3.1877300<br>H 0.0246610 -2.5797877 1.0434600<br>C 1.5614910 -1.9882187 -0.3747500<br>C 4.2530510 -1.8714397 -1.2014500<br>C 1.9093920 -1.4003987 -1.6035600<br>C 2.5893810 -2.4889387 0.4413400<br>C 3.9219610 -2.4316897 0.0344400<br>C 3.2406320 -1.3555787 -2.0170600<br>H 1.1375020 -0.9726687 -2.2343700<br>H 2.3340910 -2.9343887 1.3991800<br>H 4.6997610 -2.8357697 0.6769500<br>H 3.4887120 -0.9264397 -2.9848300<br>H 5.2885310 -1.8427297 -1.5304600<br>C 2.7621730 2.3936313 -1.9686700<br>C 4.1492530 1.8194003 -0.0041600<br>H 4.1023730 1.6842003 1.0749300<br>H 4.8846730 2.5820503 -0.2709200<br>H 4.3960520 0.8678203 -0.4765900<br>H 3.4378530 3.1968703 -2.2711200<br>H 3.0741030 1.4488313 -2.4148100<br>S -5.9807170 1.5972343 0.5052900<br>H 1.7374230 2.6159213 -2.2629600

Name	$TS_{Theo1S-BMA-R2}$
**Absolute Energies (Hartree)**	There are 13 positive frequencies below 100 cm^-1.<br><br>Zero-point correction: 0.519673 hartree<br>Enthalpy correction: 0.555844 hartree<br>Free Energy correction: 0.452612 hartree<br>Quasiharmonic Free Energy correction: 0.461101 hartree<br><br>SCF Energy: -1811.841175 hartree<br>SCF Energy*: -1812.284944 hartree<br>SCF Energy + ZPVE: -1811.321502 hartree<br>Enthalpy: -1811.285331 hartree<br>Free Energy: -1811.388563 hartree<br><br>Free Energy with quasiharmonic correction: -1811.380074 hartree (correction: 5.33 kcal/mol)<br><br>---<br>Low frequencies before projection:<br>-201.3540<br>19.3339<br>20.6982<br><br>Low frequencies after projection:<br>-201.3538<br>18.8205<br>20.3419<br>---
**Cartesian Coordinates**	N 0.5230085 2.9032588 -0.1493619<br>N 4.2297885 -1.8909122 0.2954581<br>C 4.7582385 -0.9198822 -0.4856219<br>N 3.9153885 -0.4593822 -1.4438719<br>N 0.7545385 -2.1183112 -0.4582519<br>O 1.5803385 -3.0602112 -0.2945119<br>O 1.1576585 -0.9392212 -0.7172719<br>C -0.5864215 -2.3758612 -0.3634219<br>C -1.5149415 -1.3445112 -0.5088319<br>C -1.6478515 -0.2432112 1.4501781<br>C -2.4144615 0.9027288 1.0334481<br>O -1.9859015 1.9063088 0.4455781<br>O -3.7394515 0.7422688 1.2635081<br>C -4.5983815 1.7638988 0.7422381<br>C -0.2612115 -0.0753412 1.8356381<br>O 0.4239485 0.9468188 1.7563281<br>O 0.2445185 -1.2351412 2.3192681<br>C 1.6510585 -1.2257012 2.6088381<br>C 4.2635485 0.5592478 -2.4114019<br>C 4.9318785 -2.5359022 1.3865281<br>C -0.2494515 3.6812688 -1.1578619

	H	-2.1893315	-1.0345512	1.9517481
	H	5.1357085	0.2613378	-3.0023619
	H	3.4096585	0.6891578	-3.0833119
	H	4.5001485	1.5198778	-1.9357119
	H	2.9348785	-0.7359012	-1.3684219
	H	3.3194485	-2.2788022	0.0363581
	H	5.8507785	-3.0263822	1.0449281
	H	4.2621485	-3.2885322	1.8119481
	H	5.2056185	-1.8167722	2.1667181
	H	-5.6150615	1.4185188	0.9371381
	H	-0.8220015	-3.3937612	-0.0926419
	H	0.4169285	4.3951888	-1.6472819
	H	-1.0648015	4.1982588	-0.6521519
	H	-4.4209815	2.7186388	1.2484781
	H	-4.4471915	1.8962488	-0.3326219
	H	-0.1474715	2.2428088	0.3094981
	H	-0.6635815	2.9863488	-1.8891319
	H	1.8772285	-0.5260812	3.4197781
	H	1.8918685	-2.2454512	2.9122381
	H	2.2216785	-0.9465912	1.7205181
	H	-1.1432515	-0.4414012	-0.9803019
	C	-2.9491315	-1.6246012	-0.7062819
	C	-5.6943815	-2.0740812	-1.1250919
	C	-3.6008715	-2.7040612	-0.0855019
	C	-3.6999615	-0.7717012	-1.5316319
	C	-5.0592315	-0.9963712	-1.7447019
	C	-4.9597215	-2.9251112	-0.2935019
	H	-3.0460315	-3.3597412	0.5796081
	H	-3.2090415	0.0744788	-2.0059319
	H	-5.6219815	-0.3286412	-2.3917419
	H	-5.4498715	-3.7610912	0.1981681
	H	-6.7547615	-2.2496112	-1.2849919
	C	1.6043985	2.0887588	-0.7822719
	C	1.0586585	3.7744988	0.9363881
	H	0.2231385	4.2942788	1.4087081
	H	1.7587485	4.4942888	0.5060181
	H	1.5560685	3.1390688	1.6673481
	H	2.3224485	2.7632988	-1.2530819
	H	2.0766285	1.4836888	-0.0118619
	H	1.1543185	1.4267888	-1.5209519
	S	6.3423185	-0.3185022	-0.2807019

Name	$TS_{Theo1S-BMA-R3}$
**Absolute Energies (Hartree)**	There are 13 positive frequencies below 100 cm^-1.<br><br>Zero-point correction: 0.520018 hartree<br>Enthalpy correction: 0.555948 hartree<br>Free Energy correction: 0.454647 hartree<br>Quasiharmonic Free Energy correction: 0.461641 hartree<br><br>SCF Energy: -1811.847824 hartree<br>SCF Energy*: -1812.290689 hartree<br>SCF Energy + ZPVE: -1811.327806 hartree<br>Enthalpy: -1811.291876 hartree<br>Free Energy: -1811.393176 hartree<br><br>Free Energy with quasiharmonic correction: -1811.386183 hartree (correction: 4.39 kcal/mol)<br><br>---<br>Low frequencies before projection:<br>-199.0591<br>24.0861<br>28.4521<br><br>Low frequencies after projection:<br>-199.0577<br>23.5869<br>28.2977<br>---
**Cartesian Coordinates**	N -0.7336137 2.6048677 0.1673510<br>N -4.2204337 -0.1479323 -1.3758890<br>C -4.7932137 -0.9881723 -0.4798090<br>N -3.9929747 -2.0024723 -0.0754390<br>N -0.6264337 -0.9474123 -1.2727590<br>O -1.3743837 -0.1792223 -1.9619990<br>O -1.1136347 -1.9118823 -0.6194590<br>C 0.7252863 -0.7239323 -1.3018590<br>C 1.6049353 -1.4902223 -0.5370190<br>C 1.7233263 -0.5605423 1.5288410<br>C 0.3164963 -0.5580123 1.8321310<br>O -0.5066737 0.3113077 1.5192510<br>O -0.0681347 -1.6795423 2.4821010<br>C -1.4851047 -1.8193423 2.6739410<br>C 2.3925863 0.6636777 1.1608310<br>O 1.8880763 1.6556077 0.6268710<br>O 3.7275063 0.5890867 1.3981910<br>C 4.5032063 1.7120867 0.9648110<br>C -4.3786147 -3.0143823 0.8877010<br>C -4.8886437 0.9898777 -1.9731490<br>C -2.1989137 2.3206577 0.2113510

	H 2.3251663 -1.3173523 2.0153910<br>H -5.2840047 -3.5396623 0.5671410<br>H -3.5567647 -3.7317423 0.9637710<br>H -4.5729147 -2.5846223 1.8784610<br>H -3.0226947 -2.0061123 -0.3952890<br>H -3.2560837 -0.3270723 -1.6627590<br>H -5.7715337 0.6850477 -2.5465090<br>H -4.1777037 1.4767077 -2.6474590<br>H -5.2176237 1.7095677 -1.2144290<br>H -1.6285747 -2.8260123 3.0704610<br>H 1.0135263 0.1094077 -1.9212690<br>H -2.7442037 3.1929677 -0.1564090<br>H -2.4796037 2.0975577 1.2402810<br>H -1.8567037 -1.0784423 3.3896310<br>H -2.0096047 -1.6955123 1.7252210<br>H -0.2583137 1.7420677 0.5284710<br>H -2.3968237 1.4490377 -0.4103590<br>H 4.2415863 2.6105267 1.5342610<br>H 5.5433363 1.4393767 1.1516310<br>H 4.3536663 1.9087167 -0.1002190<br>H 1.1801753 -2.3764923 -0.0784690<br>C 3.0492953 -1.5463433 -0.8234990<br>C 5.8187953 -1.7267433 -1.2946790<br>C 3.7114663 -0.5748533 -1.5945590<br>C 3.8033153 -2.6077233 -0.2936190<br>C 5.1728253 -2.6993333 -0.5271290<br>C 5.0819863 -0.6660233 -1.8274790<br>H 3.1591463 0.2634967 -2.0064390<br>H 3.3044953 -3.3619433 0.3102210<br>H 5.7368153 -3.5292233 -0.1101890<br>H 5.5766463 0.0939267 -2.4266190<br>H 6.8876253 -1.7958733 -1.4779090<br>C -0.2610137 2.8580977 -1.2239190<br>C -0.3490837 3.7037777 1.0965110<br>H -0.6574337 3.4265677 2.1061510<br>H -0.8490737 4.6257977 0.7899510<br>H 0.7339663 3.8166977 1.0639010<br>H -0.7228037 3.7774377 -1.5930490<br>H 0.8240363 2.9521477 -1.2026190<br>H -0.5569437 2.0086677 -1.8414190<br>S -6.3890137 -0.7806113 0.0888510

Name	$TS_{Theo1S-BMB-R1}$
**Absolute Energies (Hartree)**	There are 14 positive frequencies below 100 cm^-1.<br><br>Zero-point correction: 0.519061 hartree<br>Enthalpy correction: 0.555211 hartree<br>Free Energy correction: 0.453264 hartree<br>Quasiharmonic Free Energy correction: 0.460511 hartree<br><br>SCF Energy: -1811.837508 hartree<br>SCF Energy*: -1812.281316 hartree<br>SCF Energy + ZPVE: -1811.318447 hartree<br>Enthalpy: -1811.282297 hartree<br>Free Energy: -1811.384244 hartree<br><br>Free Energy with quasiharmonic correction: -1811.376997 hartree (correction: 4.55 kcal/mol)<br><br>---<br>Low frequencies before projection:<br>-168.3297<br>27.2208<br>27.6984<br><br>Low frequencies after projection:<br>-168.3292<br>26.9391<br>27.3426<br>---
**Cartesian Coordinates**	N 4.6455568 0.1895515 -0.8525918<br>C 4.6881568 -1.1056185 -0.4601718<br>S 6.1249668 -1.8377985 0.1144382<br>N 3.5121968 -1.7668695 -0.5393818<br>C 3.3228068 -3.1395995 -0.1192418<br>C -4.6897632 -0.1967205 -1.5070118<br>N -5.3671332 -0.3656105 -0.1863718<br>O -3.5167532 -0.4684405 1.7810582<br>N -2.6705232 0.4080695 1.3654182<br>O -3.0524342 1.3371595 0.6005282<br>C -1.3691032 0.2856595 1.7419382<br>C -0.3993432 1.1198905 1.1800682<br>C 1.0118968 1.0422105 1.5586382<br>C 1.6195068 -0.1624495 1.9584682<br>C 2.9680368 -0.1917395 2.3003482<br>C 3.7349368 0.9748215 2.2444382<br>C 3.1472458 2.1754305 1.8370282<br>C 1.7994658 2.2071905 1.4886082<br>C -0.3696632 0.4789905 -1.0530918<br>C -0.3966232 -0.9425295 -0.8375218<br>O 0.5439868 -1.7168595 -0.6944618

	C	0.8282968	1.1503405	-1.4725518
	O	1.9457268	0.6608105	-1.6393518
	H	3.9591478	-3.8237485	-0.6913318
	H	-4.1729932	0.7611495	-1.5056718
	H	3.7420468	0.5663215	-1.1289918
	H	2.6696968	-1.2591295	-0.8084618
	H	-1.3152132	0.9758695	-1.2242518
	H	-1.1595232	-0.5862805	2.3424882
	H	-0.7414642	2.0723605	0.7925682
	H	1.3416158	3.1365005	1.1601282
	H	3.7418958	3.0835415	1.7837382
	H	3.4340168	-1.1300495	2.5845582
	H	1.0464868	-1.0829895	1.9574182
	C	-1.8362532	-2.7265005	-0.2276218
	H	-1.2658232	-2.8952305	0.6903282
	H	-1.4989522	-3.4392905	-0.9872018
	C	1.7637758	3.2559805	-2.0099518
	H	2.1737158	2.9117505	-2.9648718
	H	2.5435258	3.1925805	-1.2446518
	O	-1.6947832	-1.3782105	-0.6840418
	O	0.6121758	2.4892305	-1.6354318
	H	-2.9007732	-2.8637205	-0.0267818
	H	1.4135758	4.2861305	-2.1023018
	H	-4.6162032	-0.3914405	0.5701882
	C	-6.2270932	0.8057185	0.1464182
	H	-6.6926132	0.6336385	1.1189482
	H	-6.9937132	0.9249385	-0.6229018
	H	-5.5847442	1.6849485	0.1993982
	H	-6.4985432	-1.7737115	0.9084682
	C	-6.1116932	-1.6526715	-0.1052818
	H	-5.4263332	-2.4702505	-0.3364818
	H	-6.9354732	-1.6435515	-0.8230318
	H	2.2736778	-3.3872495	-0.2870918
	H	-5.4422032	-0.2351005	-2.2985518
	H	3.5629768	-3.2749595	0.9430582
	H	-3.9536032	-0.9908805	-1.6306318
	C	5.7673168	1.1009315	-0.7964018
	H	4.7906268	0.9384115	2.4964182
	H	5.4328858	2.0656215	-1.1901118
	H	6.1298568	1.2415615	0.2292982
	H	6.6075368	0.7418915	-1.4006018

Name	$TS_{Theo1S\text{-}BMB\text{-}R1\mathit{conf}}$
**Absolute Energies (Hartree)**	There are 14 positive frequencies below 100 cm^-1.<br><br>Zero-point correction: 0.519052 hartree<br>Enthalpy correction: 0.555208 hartree<br>Free Energy correction: 0.453232 hartree<br>Quasiharmonic Free Energy correction: 0.460496 hartree<br><br>SCF Energy: -1811.837508 hartree<br>SCF Energy*: -1812.281316 hartree<br>SCF Energy + ZPVE: -1811.318456 hartree<br>Enthalpy: -1811.282300 hartree<br>Free Energy: -1811.384276 hartree<br><br>Free Energy with quasiharmonic correction: -1811.377012 hartree (correction: 4.56 kcal/mol)<br><br>---<br>Low frequencies before projection:<br>-168.3559<br>27.1468<br>27.6026<br><br>Low frequencies after projection:<br>-168.3554<br>26.8743<br>27.1747<br>---
**Cartesian Coordinates**	N 4.6456160 0.1898028 -0.8523582<br>C 4.6882260 -1.1054472 -0.4601982<br>S 6.1249860 -1.8376762 0.1144818<br>N 3.5122960 -1.7667172 -0.5396882<br>C 3.3228860 -3.1395372 -0.1198682<br>C -4.6902240 -0.1957572 -1.5067182<br>N -5.3674240 -0.3653772 -0.1860782<br>O -3.5165940 -0.4691772 1.7808718<br>N -2.6703840 0.4074528 1.3654318<br>O -3.0523540 1.3368328 0.6009118<br>C -1.3689340 0.2848928 1.7417918<br>C -0.3992040 1.1193128 1.1801518<br>C 1.0120460 1.0416028 1.5586618<br>C 1.6197260 -0.1630772 1.9583318<br>C 2.9682660 -0.1923372 2.3001918<br>C 3.7351060 0.9742628 2.2444218<br>C 3.1473460 2.1749028 1.8371618<br>C 1.7995660 2.2066328 1.4887718<br>C -0.3695540 0.4791328 -1.0531682<br>C -0.3964840 -0.9424372 -0.8379582<br>O 0.5441360 -1.7167472 -0.6948682

	C	0.8283960	1.1506128	-1.4724682
	O	1.9458560	0.6611528	-1.6392482
	H	3.9594060	-3.8235272	-0.6919682
	H	-4.1738440	0.7623228	-1.5051082
	H	3.7421360	0.5665528	-1.1288682
	H	2.6698160	-1.2588972	-0.8086482
	H	-1.3151240	0.9760028	-1.2242482
	H	-1.1593140	-0.5872672	2.3420118
	H	-0.7413640	2.0719028	0.7929918
	H	1.3416660	3.1359628	1.1604118
	H	3.7419460	3.0830428	1.7839818
	H	3.4343160	-1.1306672	2.5842618
	H	1.0467460	-1.0836372	1.9571818
	C	-1.8362240	-2.7265672	-0.2286882
	H	-1.2649940	-2.8957772	0.6886718
	H	-1.4999040	-3.4392772	-0.9887882
	C	1.7638160	3.2563628	-2.0095582
	H	2.1738260	2.9122528	-2.9644882
	H	2.5435260	3.1929028	-1.2442282
	O	-1.6946540	-1.3782072	-0.6848782
	O	0.6122260	2.4895128	-1.6351782
	H	-2.9006040	-2.8634272	-0.0268782
	H	1.4135760	4.2865028	-2.1017982
	H	-4.6163640	-0.3916672	0.5703418
	C	-6.2272740	0.8058028	0.1475318
	H	-6.6926340	0.6331828	1.1200418
	H	-6.9940240	0.9255028	-0.6215882
	H	-5.5848740	1.6849828	0.2009018
	H	-6.4985540	-1.7741872	0.9081518
	C	-6.1120540	-1.6524372	-0.1056482
	H	-5.4268740	-2.4699172	-0.3377182
	H	-6.9361040	-1.6427072	-0.8230882
	H	2.2738160	-3.3872172	-0.2880182
	H	-5.4427040	-0.2342072	-2.2982182
	H	3.5628260	-3.2751172	0.9424518
	H	-3.9537440	-0.9895772	-1.6306182
	C	5.7673060	1.1012438	-0.7959182
	H	4.7907960	0.9378728	2.4963918
	H	5.4327760	2.0660438	-1.1892682
	H	6.1298760	1.2415338	0.2298118
	H	6.6075360	0.7424938	-1.4002882

Name	$TS_{Theo1S-BMB-R2}$
**Absolute Energies (Hartree)**	There are 15 positive frequencies below 100 cm^-1.<br><br>Zero-point correction: 0.518888 hartree<br>Enthalpy correction: 0.555161 hartree<br>Free Energy correction: 0.449516 hartree<br>Quasiharmonic Free Energy correction: 0.457317 hartree<br><br>SCF Energy: -1811.837855 hartree<br>SCF Energy*: -1812.281167 hartree<br>SCF Energy + ZPVE: -1811.318967 hartree<br>Enthalpy: -1811.282694 hartree<br>Free Energy: -1811.388339 hartree<br><br>Free Energy with quasiharmonic correction: -1811.380538 hartree (correction: 4.90 kcal/mol)<br><br>---<br>Low frequencies before projection:<br>-189.4181<br>20.5627<br>26.5149<br><br>Low frequencies after projection:<br>-189.418<br>20.4544<br>25.8971<br>---
**Cartesian Coordinates**	N -2.4606236 -2.2069568 0.6608852<br>C -2.0472936 -2.3125768 -0.6309048<br>S -3.1592536 -2.4226768 -1.9301648<br>N -0.7126336 -2.2944468 -0.8063548<br>C -0.0566636 -2.2051668 -2.0941948<br>C -4.5103736 3.1299532 -0.4569248<br>N -3.6513136 1.9154332 -0.5242648<br>O -1.1647836 2.8460432 0.4169052<br>N -0.4572036 1.8135132 0.1430952<br>O -1.0246436 0.7056632 -0.0808448<br>C 0.8983064 1.9397332 0.0727752<br>C 1.6840464 0.8415042 -0.2757048<br>C 3.0974164 0.9909742 -0.6511948<br>C 3.9363264 1.9568842 -0.0679048<br>C 5.2716264 2.0564542 -0.4480248<br>C 5.7950164 1.1958542 -1.4185448<br>C 4.9720364 0.2324942 -2.0048948<br>C 3.6363464 0.1279342 -1.6201748<br>C 1.9826964 -0.4549658 1.6291952<br>C 2.6071164 -1.5953558 0.9928252<br>O 2.0419964 -2.4710558 0.3392252

	C	0.6429664	-0.5678068	2.1502252
	O	-0.0948236	-1.5555668	2.1114652
	H	-0.4062026	-2.9914368	-2.7699648
	H	-1.7324336	-2.0475068	1.3549152
	H	-0.0985836	-2.2477768	0.0087252
	H	2.6313364	0.2688642	2.1048952
	H	1.2717064	2.9189842	0.3311452
	H	1.1568564	0.0035242	-0.7167848
	H	2.9980664	-0.6273458	-2.0703448
	H	5.3703464	-0.4414758	-2.7586448
	H	5.9096864	2.8027342	0.0177552
	H	3.5456964	2.6143342	0.7038752
	C	4.6772564	-2.5727658	0.4051952
	H	4.4138064	-2.5755758	-0.6559148
	H	4.4661274	-3.5618358	0.8253152
	O	3.9620364	-1.5538458	1.1141952
	O	0.2496064	0.5969332	2.7371952
	H	5.7344164	-2.3294458	0.5280452
	H	-2.7176336	2.1776232	-0.1228848
	C	-4.1799336	0.7947732	0.3081452
	H	-3.4430136	-0.0073668	0.2973652
	H	-5.1273336	0.4542632	-0.1144548
	H	-4.3291836	1.1594232	1.3266452
	H	-2.7857036	0.5855532	-1.9189448
	C	-3.4067336	1.4804532	-1.9336448
	H	-2.8987836	2.2908132	-2.4602448
	H	-4.3659136	1.2605432	-2.4073748
	H	1.0135964	-2.3275668	-1.9166648
	H	-5.4961236	2.8963332	-0.8654748
	H	-0.2410136	-1.2361668	-2.5765348
	H	-4.0406436	3.9252732	-1.0387748
	C	-3.7691226	-2.6085268	1.1386252
	H	6.8384264	1.2749942	-1.7116248
	H	-3.8227436	-2.3695868	2.2049452
	H	-4.5673436	-2.0780168	0.6112852
	H	-3.9440226	-3.6845768	1.0077952
	H	-4.5994336	3.4419032	0.5855952
	C	-1.1192636	0.6413132	3.1516552
	H	-1.3278336	-0.1194868	3.9110452
	H	-1.7771936	0.4859432	2.2909852
	H	-1.2690636	1.6398632	3.5664352

Name	$TS_{Theo1S-BMB-R3}$
Absolute Energies (Hartree)	There are 13 positive frequencies below 100 cm^-1.<br><br>Zero-point correction: 0.519067 hartree<br>Enthalpy correction: 0.555379 hartree<br>Free Energy correction: 0.452169 hartree<br>Quasiharmonic Free Energy correction: 0.460520 hartree<br><br>SCF Energy: -1811.842602 hartree<br>SCF Energy*: -1812.286383 hartree<br>SCF Energy + ZPVE: -1811.323535 hartree<br>Enthalpy: -1811.287223 hartree<br>Free Energy: -1811.390433 hartree<br><br>Free Energy with quasiharmonic correction: -1811.382083 hartree (correction: 5.24 kcal/mol)<br><br>---<br>Low frequencies before projection:<br>-140.0585<br>15.3547<br>23.5914<br><br>Low frequencies after projection:<br>-140.058<br>11.1581<br>23.4145<br>---
Cartesian Coordinates	N -1.2896740 2.1894570 -0.7687481<br>C -2.3499740 2.3009870 0.0555819<br>S -3.9104940 2.7641370 -0.4897681<br>N -2.0886040 1.9912370 1.3470619<br>C -3.0585440 2.0464170 2.4199619<br>C -3.5005040 -1.0759630 0.5189219<br>N -3.4841640 -1.6095930 -0.8784081<br>O -1.1796140 -0.7132330 -1.9882481<br>N -0.2317040 -1.2164530 -1.2930181<br>O -0.4099640 -2.2769130 -0.6460581<br>C 0.9805560 -0.5698030 -1.3256281<br>C 2.0646160 -1.0400830 -0.6016281<br>C 3.4387060 -0.5808220 -0.8325581<br>C 3.7399260 0.6118880 -1.5150181<br>C 5.0616660 1.0061380 -1.7058381<br>C 6.1090860 0.2197880 -1.2190081<br>C 5.8243660 -0.9665920 -0.5374681<br>C 4.5032060 -1.3600020 -0.3446081<br>C 1.8668960 -0.1881630 1.6218919<br>C 0.6057960 -0.8047230 1.9183319<br>O -0.5245840 -0.3382730 1.7517519

	C	1.9724260	1.2001170	1.2750319
	O	1.0947660	1.9384270	0.8167419
	H	-3.4868140	3.0492370	2.5099419
	H	-2.5388940	-1.2847430	0.9893019
	H	-0.3960340	1.9475170	-0.3387781
	H	-1.2373540	1.4565370	1.5278119
	H	2.7551560	-0.6699920	2.0079519
	H	0.9707160	0.3237870	-1.9284881
	H	1.9510760	-2.0161530	-0.1436681
	H	4.2833560	-2.2782620	0.1945519
	H	6.6327260	-1.5835520	-0.1545581
	H	7.1397060	0.5300680	-1.3683281
	H	5.2750560	1.9321080	-2.2330681
	H	2.9401160	1.2449680	-1.8851081
	C	-0.4080840	-2.8244930	2.6143519
	H	-0.9154240	-3.0050330	1.6614819
	H	-1.0916440	-2.3111930	3.2988219
	C	3.4851660	3.0043980	1.0363919
	H	2.9311960	3.7073880	1.6676819
	H	3.1945160	3.1641780	-0.0058081
	O	0.7858960	-2.0686130	2.4054119
	O	3.2474460	1.6531980	1.4435719
	H	-0.0857140	-3.7723230	3.0517019
	H	4.5593560	3.1612880	1.1527419
	H	-2.5740340	-1.2908830	-1.3168881
	C	-3.4703140	-3.0986330	-0.9048781
	H	-3.3729040	-3.4309330	-1.9403981
	H	-4.4028740	-3.4726930	-0.4756681
	H	-2.6101640	-3.4436630	-0.3327481
	H	-4.5003840	-1.4182130	-2.7216781
	C	-4.5894240	-1.0385530	-1.7016781
	H	-4.4996140	0.0505070	-1.6940581
	H	-5.5481440	-1.3380330	-1.2711781
	C	-1.3194640	2.4501370	-2.1919681
	H	-1.9313340	1.7155770	-2.7246181
	H	-0.2933240	2.3822770	-2.5642881
	H	-1.7094340	3.4511570	-2.4046281
	H	-2.5379640	1.7949870	3.3481419
	H	-4.3225840	-1.5450530	1.0654019
	H	-3.8902340	1.3426170	2.2798719
	H	-3.6535140	0.0011070	0.4680719

Name	$TS_{Theo1S\text{-}BMB\text{-}S1}$
**Absolute Energies (Hartree)**	There are 15 positive frequencies below 100 cm^-1.<br><br>Zero-point correction: 0.518888 hartree<br>Enthalpy correction: 0.555161 hartree<br>Free Energy correction: 0.449516 hartree<br>Quasiharmonic Free Energy correction: 0.457317 hartree<br><br>SCF Energy: -1811.837855 hartree<br>SCF Energy*: -1812.281167 hartree<br>SCF Energy + ZPVE: -1811.318967 hartree<br>Enthalpy: -1811.282694 hartree<br>Free Energy: -1811.388339 hartree<br><br>Free Energy with quasiharmonic correction: -1811.380538 hartree (correction: 4.90 kcal/mol)<br><br>---<br>Low frequencies before projection:<br>-189.4181<br>20.5627<br>26.5149<br><br>Low frequencies after projection:<br>-189.418<br>20.4544<br>25.8971<br>---
**Cartesian Coordinates**	N -2.4606236 -2.2069568 -0.6608852<br>C -2.0472936 -2.3125768 0.6309048<br>S -3.1592536 -2.4226768 1.9301648<br>N -0.7126336 -2.2944468 0.8063548<br>C -0.0566636 -2.2051668 2.0941948<br>C -4.5103736 3.1299532 0.4569248<br>N -3.6513136 1.9154332 0.5242648<br>O -1.1647836 2.8460432 -0.4169052<br>N -0.4572036 1.8135132 -0.1430952<br>O -1.0246436 0.7056632 0.0808448<br>C 0.8983064 1.9397332 -0.0727752<br>C 1.6840464 0.8415042 0.2757048<br>C 3.0974164 0.9909742 0.6511948<br>C 3.9363264 1.9568842 0.0679048<br>C 5.2716264 2.0564542 0.4480248<br>C 5.7950164 1.1958542 1.4185448<br>C 4.9720364 0.2324942 2.0048948<br>C 3.6363464 0.1279342 1.6201748<br>C 1.9826964 -0.4549658 -1.6291952<br>C 2.6071164 -1.5953558 -0.9928252<br>O 2.0419964 -2.4710558 -0.3392252

	C	0.6429664	-0.5678068	-2.1502252
	O	-0.0948236	-1.5555668	-2.1114652
	H	-0.4062026	-2.9914368	2.7699648
	H	-1.7324336	-2.0475068	-1.3549152
	H	-0.0985836	-2.2477768	-0.0087252
	H	2.6313364	0.2688642	-2.1048952
	H	1.2717064	2.9189842	-0.3311452
	H	1.1568564	0.0035242	0.7167848
	H	2.9980664	-0.6273458	2.0703448
	H	5.3703464	-0.4414758	2.7586448
	H	5.9096864	2.8027342	-0.0177552
	H	3.5456964	2.6143342	-0.7038752
	C	4.6772564	-2.5727658	-0.4051952
	H	4.4138064	-2.5755758	0.6559148
	H	4.4661274	-3.5618358	-0.8253152
	O	3.9620364	-1.5538458	-1.1141952
	O	0.2496064	0.5969332	-2.7371952
	H	5.7344164	-2.3294458	-0.5280452
	H	-2.7176336	2.1776232	0.1228848
	C	-4.1799336	0.7947732	-0.3081452
	H	-3.4430136	-0.0073668	-0.2973652
	H	-5.1273336	0.4542632	0.1144548
	H	-4.3291836	1.1594232	-1.3266452
	H	-2.7857036	0.5855532	1.9189448
	C	-3.4067336	1.4804532	1.9336448
	H	-2.8987836	2.2908132	2.4602448
	H	-4.3659136	1.2605432	2.4073748
	H	1.0135964	-2.3275668	1.9166648
	H	-5.4961236	2.8963332	0.8654748
	H	-0.2410136	-1.2361668	2.5765348
	H	-4.0406436	3.9252732	1.0387748
	C	-3.7691226	-2.6085268	-1.1386252
	H	6.8384264	1.2749942	1.7116248
	H	-3.8227436	-2.3695868	-2.2049452
	H	-4.5673436	-2.0780168	-0.6112852
	H	-3.9440226	-3.6845768	-1.0077952
	H	-4.5994336	3.4419032	-0.5855952
	C	-1.1192636	0.6413132	-3.1516552
	H	-1.3278336	-0.1194868	-3.9110452
	H	-1.7771936	0.4859432	-2.2909852
	H	-1.2690636	1.6398632	-3.5664352

Name	$TS_{Theo1S\text{-}BMB\text{-}S2}$
Absolute Energies (Hartree)	There are 14 positive frequencies below 100 cm^-1.<br><br>Zero-point correction: 0.519061 hartree<br>Enthalpy correction: 0.555211 hartree<br>Free Energy correction: 0.453264 hartree<br>Quasiharmonic Free Energy correction: 0.460511 hartree<br><br>SCF Energy: -1811.837508 hartree<br>SCF Energy*: -1812.281316 hartree<br>SCF Energy + ZPVE: -1811.318447 hartree<br>Enthalpy: -1811.282297 hartree<br>Free Energy: -1811.384244 hartree<br><br>Free Energy with quasiharmonic correction: -1811.376997 hartree (correction: 4.55 kcal/mol)<br><br>---<br>Low frequencies before projection:<br>-168.3297<br>27.2208<br>27.6984<br><br>Low frequencies after projection:<br>-168.3292<br>26.9391<br>27.3426<br>---
Cartesian Coordinates	N 4.6455568 0.1895515 0.8525918<br>C 4.6881568 -1.1056185 0.4601718<br>S 6.1249668 -1.8377985 -0.1144382<br>N 3.5121968 -1.7668695 0.5393818<br>C 3.3228068 -3.1395995 0.1192418<br>C -4.6897632 -0.1967205 1.5070118<br>N -5.3671332 -0.3656105 0.1863718<br>O -3.5167532 -0.4684405 -1.7810582<br>N -2.6705232 0.4080695 -1.3654182<br>O -3.0524342 1.3371595 -0.6005282<br>C -1.3691032 0.2856595 -1.7419382<br>C -0.3993432 1.1198905 -1.1800682<br>C 1.0118968 1.0422105 -1.5586382<br>C 1.6195068 -0.1624495 -1.9584682<br>C 2.9680368 -0.1917395 -2.3003482<br>C 3.7349368 0.9748215 -2.2444382<br>C 3.1472458 2.1754305 -1.8370282<br>C 1.7994658 2.2071905 -1.4886082<br>C -0.3696632 0.4789905 1.0530918<br>C -0.3966232 -0.9425295 0.8375218<br>O 0.5439868 -1.7168595 0.6944618

	C	0.8282968	1.1503405	1.4725518
	O	1.9457268	0.6608105	1.6393518
	H	3.9591478	-3.8237485	0.6913318
	H	-4.1729932	0.7611495	1.5056718
	H	3.7420468	0.5663215	1.1289918
	H	2.6696968	-1.2591295	0.8084618
	H	-1.3152132	0.9758695	1.2242518
	H	-1.1595232	-0.5862805	-2.3424882
	H	-0.7414642	2.0723605	-0.7925682
	H	1.3416158	3.1365005	-1.1601282
	H	3.7418958	3.0835415	-1.7837382
	H	3.4340168	-1.1300495	-2.5845582
	H	1.0464868	-1.0829895	-1.9574182
	C	-1.8362532	-2.7265005	0.2276218
	H	-1.2658232	-2.8952305	-0.6903282
	H	-1.4989522	-3.4392905	0.9872018
	C	1.7637758	3.2559805	2.0099518
	H	2.1737158	2.9117505	2.9648718
	H	2.5435258	3.1925805	1.2446518
	O	-1.6947832	-1.3782105	0.6840418
	O	0.6121758	2.4892305	1.6354318
	H	-2.9007732	-2.8637205	0.0267818
	H	1.4135758	4.2861305	2.1023018
	H	-4.6162032	-0.3914405	-0.5701882
	C	-6.2270932	0.8057185	-0.1464182
	H	-6.6926132	0.6336385	-1.1189482
	H	-6.9937132	0.9249385	0.6229018
	H	-5.5847442	1.6849485	-0.1993982
	H	-6.4985432	-1.7737115	-0.9084682
	C	-6.1116932	-1.6526715	0.1052818
	H	-5.4263332	-2.4702505	0.3364818
	H	-6.9354732	-1.6435515	0.8230318
	H	2.2736778	-3.3872495	0.2870918
	H	-5.4422032	-0.2351005	2.2985518
	H	3.5629768	-3.2749595	-0.9430582
	H	-3.9536032	-0.9908805	1.6306318
	C	5.7673168	1.1009315	0.7964018
	H	4.7906268	0.9384115	-2.4964182
	H	5.4328858	2.0656215	1.1901118
	H	6.1298568	1.2415615	-0.2292982
	H	6.6075368	0.7418915	1.4006018

Name	TS$_{Theo1S-BMB-S2}$*conf*
**Absolute Energies (Hartree)**	There are 14 positive frequencies below 100 cm^-1.<br><br>Zero-point correction: 0.519052 hartree<br>Enthalpy correction: 0.555208 hartree<br>Free Energy correction: 0.453232 hartree<br>Quasiharmonic Free Energy correction: 0.460496 hartree<br><br>SCF Energy: -1811.837508 hartree<br>SCF Energy*: -1812.281316 hartree<br>SCF Energy + ZPVE: -1811.318456 hartree<br>Enthalpy: -1811.282300 hartree<br>Free Energy: -1811.384276 hartree<br><br>Free Energy with quasiharmonic correction: -1811.377012 hartree (correction: 4.56 kcal/mol)<br><br>--<br>Low frequencies before projection:<br>-168.3559<br>27.1468<br>27.6026<br><br>Low frequencies after projection:<br>-168.3554<br>26.8743<br>27.1747<br>--
**Cartesian Coordinates**	N 4.6456160 0.1898028 0.8523582<br>C 4.6882260 -1.1054472 0.4601982<br>S 6.1249860 -1.8376762 -0.1144818<br>N 3.5122960 -1.7667172 0.5396882<br>C 3.3228860 -3.1395372 0.1198682<br>C -4.6902240 -0.1957572 1.5067182<br>N -5.3674240 -0.3653772 0.1860782<br>O -3.5165940 -0.4691772 -1.7808718<br>N -2.6703840 0.4074528 -1.3654318<br>O -3.0523540 1.3368328 -0.6009118<br>C -1.3689340 0.2848928 -1.7417918<br>C -0.3992040 1.1193128 -1.1801518<br>C 1.0120460 1.0416028 -1.5586618<br>C 1.6197260 -0.1630772 -1.9583318<br>C 2.9682660 -0.1923372 -2.3001918<br>C 3.7351060 0.9742628 -2.2444218<br>C 3.1473460 2.1749028 -1.8371618<br>C 1.7995660 2.2066328 -1.4887718<br>C -0.3695540 0.4791328 1.0531682<br>C -0.3964840 -0.9424372 0.8379582<br>O 0.5441360 -1.7167472 0.6948682

	C	0.8283960	1.1506128	1.4724682
	O	1.9458560	0.6611528	1.6392482
	H	3.9594060	-3.8235272	0.6919682
	H	-4.1738440	0.7623228	1.5051082
	H	3.7421360	0.5665528	1.1288682
	H	2.6698160	-1.2588972	0.8086482
	H	-1.3151240	0.9760028	1.2242482
	H	-1.1593140	-0.5872672	-2.3420118
	H	-0.7413640	2.0719028	-0.7929918
	H	1.3416660	3.1359628	-1.1604118
	H	3.7419460	3.0830428	-1.7839818
	H	3.4343160	-1.1306672	-2.5842618
	H	1.0467460	-1.0836372	-1.9571818
	C	-1.8362240	-2.7265672	0.2286882
	H	-1.2649940	-2.8957772	-0.6886718
	H	-1.4999040	-3.4392772	0.9887882
	C	1.7638160	3.2563628	2.0095582
	H	2.1738260	2.9122528	2.9644882
	H	2.5435260	3.1929028	1.2442282
	O	-1.6946540	-1.3782072	0.6848782
	O	0.6122260	2.4895128	1.6351782
	H	-2.9006040	-2.8634272	0.0268782
	H	1.4135760	4.2865028	2.1017982
	H	-4.6163640	-0.3916672	-0.5703418
	C	-6.2272740	0.8058028	-0.1475318
	H	-6.6926340	0.6331828	-1.1200418
	H	-6.9940240	0.9255028	0.6215882
	H	-5.5848740	1.6849828	-0.2009018
	H	-6.4985540	-1.7741872	-0.9081518
	C	-6.1120540	-1.6524372	0.1056482
	H	-5.4268740	-2.4699172	0.3377182
	H	-6.9361040	-1.6427072	0.8230882
	H	2.2738160	-3.3872172	0.2880182
	H	-5.4427040	-0.2342072	2.2982182
	H	3.5628260	-3.2751172	-0.9424518
	H	-3.9537440	-0.9895772	1.6306182
	C	5.7673060	1.1012438	0.7959182
	H	4.7907960	0.9378728	-2.4963918
	H	5.4327760	2.0660438	1.1892682
	H	6.1298760	1.2415338	-0.2298118
	H	6.6075360	0.7424938	1.4002882

Name	$TS_{Theo1S-BMB-S3}$
Absolute Energies (Hartree)	There are 13 positive frequencies below 100 cm^-1.<br><br>Zero-point correction: 0.519067 hartree<br>Enthalpy correction: 0.555379 hartree<br>Free Energy correction: 0.452169 hartree<br>Quasiharmonic Free Energy correction: 0.460520 hartree<br><br>SCF Energy: -1811.842602 hartree<br>SCF Energy*: -1812.286383 hartree<br>SCF Energy + ZPVE: -1811.323535 hartree<br>Enthalpy: -1811.287223 hartree<br>Free Energy: -1811.390433 hartree<br><br>Free Energy with quasiharmonic correction: -1811.382083 hartree (correction: 5.24 kcal/mol)<br><br>---<br>Low frequencies before projection:<br>-140.0585<br>15.3547<br>23.5914<br><br>Low frequencies after projection:<br>-140.058<br>11.1581<br>23.4145<br>---
Cartesian Coordinates	N -1.2896740 2.1894570 0.7687481<br>C -2.3499740 2.3009870 -0.0555819<br>S -3.9104940 2.7641370 0.4897681<br>N -2.0886040 1.9912370 -1.3470619<br>C -3.0585440 2.0464170 -2.4199619<br>C -3.5005040 -1.0759630 -0.5189219<br>N -3.4841640 -1.6095930 0.8784081<br>O -1.1796140 -0.7132330 1.9882481<br>N -0.2317040 -1.2164530 1.2930181<br>O -0.4099640 -2.2769130 0.6460581<br>C 0.9805560 -0.5698030 1.3256281<br>C 2.0646160 -1.0400830 0.6016281<br>C 3.4387060 -0.5808220 0.8325581<br>C 3.7399260 0.6118880 1.5150181<br>C 5.0616660 1.0061380 1.7058381<br>C 6.1090860 0.2197880 1.2190081<br>C 5.8243660 -0.9665920 0.5374681<br>C 4.5032060 -1.3600020 0.3446081<br>C 1.8668960 -0.1881630 -1.6218919<br>C 0.6057960 -0.8047230 -1.9183319<br>O -0.5245840 -0.3382730 -1.7517519

	C	1.9724260	1.2001170	-1.2750319
	O	1.0947660	1.9384270	-0.8167419
	H	-3.4868140	3.0492370	-2.5099419
	H	-2.5388940	-1.2847430	-0.9893019
	H	-0.3960340	1.9475170	0.3387781
	H	-1.2373540	1.4565370	-1.5278119
	H	2.7551560	-0.6699920	-2.0079519
	H	0.9707160	0.3237870	1.9284881
	H	1.9510760	-2.0161530	0.1436681
	H	4.2833560	-2.2782620	-0.1945519
	H	6.6327260	-1.5835520	0.1545581
	H	7.1397060	0.5300680	1.3683281
	H	5.2750560	1.9321080	2.2330681
	H	2.9401160	1.2449680	1.8851081
	C	-0.4080840	-2.8244930	-2.6143519
	H	-0.9154240	-3.0050330	-1.6614819
	H	-1.0916440	-2.3111930	-3.2988219
	C	3.4851660	3.0043980	-1.0363919
	H	2.9311960	3.7073880	-1.6676819
	H	3.1945160	3.1641780	0.0058081
	O	0.7858960	-2.0686130	-2.4054119
	O	3.2474460	1.6531980	-1.4435719
	H	-0.0857140	-3.7723230	-3.0517019
	H	4.5593560	3.1612880	-1.1527419
	H	-2.5740340	-1.2908830	1.3168881
	C	-3.4703140	-3.0986330	0.9048781
	H	-3.3729040	-3.4309330	1.9403981
	H	-4.4028740	-3.4726930	0.4756681
	H	-2.6101640	-3.4436630	0.3327481
	H	-4.5003840	-1.4182130	2.7216781
	C	-4.5894240	-1.0385530	1.7016781
	H	-4.4996140	0.0505070	1.6940581
	H	-5.5481440	-1.3380330	1.2711781
	C	-1.3194640	2.4501370	2.1919681
	H	-1.9313340	1.7155770	2.7246181
	H	-0.2933240	2.3822770	2.5642881
	H	-1.7094340	3.4511570	2.4046281
	H	-2.5379640	1.7949870	-3.3481419
	H	-4.3225840	-1.5450530	-1.0654019
	H	-3.8902340	1.3426170	-2.2798719
	H	-3.6535140	0.0011070	-0.4680719

<table>
<tr><td>Name</td><td>TS_{Theo1S-BMA-S13}</td></tr>
<tr><td>Absolute Energies (Hartree)</td><td>There are 13 positive frequencies below 100 cm^-1.

Zero-point correction: 0.519673 hartree
Enthalpy correction: 0.555844 hartree
Free Energy correction: 0.452612 hartree
Quasiharmonic Free Energy correction: 0.461101 hartree

SCF Energy: -1811.841175 hartree
SCF Energy*: -1812.284944 hartree
SCF Energy + ZPVE: -1811.321502 hartree
Enthalpy: -1811.285331 hartree
Free Energy: -1811.388563 hartree

Free Energy with quasiharmonic correction: -1811.380074 hartree (correction: 5.33 kcal/mol)

Low frequencies before projection:
-201.3540
19.3339
20.6982

Low frequencies after projection:
-201.3538
18.8205
20.3419
---</td></tr>
<tr><td>Cartesian Coordinates</td><td>N 0.5230085 2.9032588 0.1493619
N 4.2297885 -1.8909122 -0.2954581
C 4.7582385 -0.9198822 0.4856219
N 3.9153885 -0.4593822 1.4438719
N 0.7545385 -2.1183112 0.4582519
O 1.5803385 -3.0602112 0.2945119
O 1.1576585 -0.9392212 0.7172719
C -0.5864215 -2.3758612 0.3634219
C -1.5149415 -1.3445112 0.5088319
C -1.6478515 -0.2432112 -1.4501781
C -2.4144615 0.9027288 -1.0334481
O -1.9859015 1.9063088 -0.4455781
O -3.7394515 0.7422688 -1.2635081
C -4.5983815 1.7638988 -0.7422381
C -0.2612115 -0.0753412 -1.8356381
O 0.4239485 0.9468188 -1.7563281
O 0.2445185 -1.2351412 -2.3192681
C 1.6510585 -1.2257012 -2.6088381
C 4.2635485 0.5592478 2.4114019
C 4.9318785 -2.5359022 -1.3865281
C -0.2494515 3.6812688 1.1578619</td></tr>
</table>

	H	-2.1893315	-1.0345512	-1.9517481
	H	5.1357085	0.2613378	3.0023619
	H	3.4096585	0.6891578	3.0833119
	H	4.5001485	1.5198778	1.9357119
	H	2.9348785	-0.7359012	1.3684219
	H	3.3194485	-2.2788022	-0.0363581
	H	5.8507785	-3.0263822	-1.0449281
	H	4.2621485	-3.2885322	-1.8119481
	H	5.2056185	-1.8167722	-2.1667181
	H	-5.6150615	1.4185188	-0.9371381
	H	-0.8220015	-3.3937612	0.0926419
	H	0.4169285	4.3951888	1.6472819
	H	-1.0648015	4.1982588	0.6521519
	H	-4.4209815	2.7186388	-1.2484781
	H	-4.4471915	1.8962488	0.3326219
	H	-0.1474715	2.2428088	-0.3094981
	H	-0.6635815	2.9863488	1.8891319
	H	1.8772285	-0.5260812	-3.4197781
	H	1.8918685	-2.2454512	-2.9122381
	H	2.2216785	-0.9465912	-1.7205181
	H	-1.1432515	-0.4414012	0.9803019
	C	-2.9491315	-1.6246012	0.7062819
	C	-5.6943815	-2.0740812	1.1250919
	C	-3.6008715	-2.7040612	0.0855019
	C	-3.6999615	-0.7717012	1.5316319
	C	-5.0592315	-0.9963712	1.7447019
	C	-4.9597215	-2.9251112	0.2935019
	H	-3.0460315	-3.3597412	-0.5796081
	H	-3.2090415	0.0744788	2.0059319
	H	-5.6219815	-0.3286412	2.3917419
	H	-5.4498715	-3.7610912	-0.1981681
	H	-6.7547615	-2.2496112	1.2849919
	C	1.6043985	2.0887588	0.7822719
	C	1.0586585	3.7744988	-0.9363881
	H	0.2231385	4.2942788	-1.4087081
	H	1.7587485	4.4942888	-0.5060181
	H	1.5560685	3.1390688	-1.6673481
	H	2.3224485	2.7632988	1.2530819
	H	2.0766285	1.4836888	0.0118619
	H	1.1543185	1.4267888	1.5209519
	S	6.3423185	-0.3185022	0.2807019

Name	$TS_{Theo1S\text{-}BMA\text{-}S23}$
**Absolute Energies (Hartree)**	There are 13 positive frequencies below 100 cm^-1.<br><br>Zero-point correction: 0.519874 hartree<br>Enthalpy correction: 0.556007 hartree<br>Free Energy correction: 0.453221 hartree<br>Quasiharmonic Free Energy correction: 0.461516 hartree<br><br>SCF Energy: -1811.838558 hartree<br>SCF Energy*: -1812.282065 hartree<br>SCF Energy + ZPVE: -1811.318684 hartree<br>Enthalpy: -1811.282551 hartree<br>Free Energy: -1811.385337 hartree<br><br>Free Energy with quasiharmonic correction: -1811.377043 hartree (correction: 5.20 kcal/mol)<br><br>---<br>Low frequencies before projection:<br>-322.0715<br>17.5193<br>24.6286<br><br>Low frequencies after projection:<br>-322.0713<br>17.2392<br>24.4857<br>---
**Cartesian Coordinates**	N 2.8060030 2.2530813 0.4851300<br>N -5.1520780 -0.6575557 0.7128300<br>C -5.0809680 0.1476143 -0.3740500<br>N -4.2384080 -0.2840667 -1.3403300<br>N -2.1560190 -2.4671467 0.4658700<br>O -3.0686690 -2.5710367 1.3415800<br>O -2.4400490 -2.4939067 -0.7760700<br>C -0.8652790 -2.3084977 0.8638200<br>C 0.1551710 -2.0782677 -0.0888700<br>C -0.2228580 -0.2684977 -1.0225900<br>C -0.5198880 0.6032623 0.0990100<br>O 0.2714430 1.3017223 0.7413000<br>O -1.8081280 0.4712633 0.4636200<br>C -2.2188470 1.1191433 1.6770500<br>C 0.9597920 -0.0412487 -1.8295400<br>O 1.9126420 0.6992013 -1.5806000<br>O 0.9546520 -0.8344887 -2.9292500<br>C 2.1240020 -0.7576787 -3.7583200<br>C -3.8242780 0.5161433 -2.4734000<br>C -6.0309080 -0.4337357 1.8422800<br>C 2.3404940 3.4932913 -0.2021100

	H	-1.0914380	-0.6457477	-1.5490100
	H	-4.6875180	0.8447143	-3.0594100
	H	-3.1818780	-0.1057867	-3.1039800
	H	-3.2641670	1.4085133	-2.1626600
	H	-3.6668580	-1.1078567	-1.1369700
	H	-4.5035880	-1.4470557	0.7866200
	H	-7.0840480	-0.4354047	1.5393800
	H	-5.8635380	-1.2444357	2.5571500
	H	-5.8287780	0.5257143	2.3340700
	H	-3.2356280	0.7722333	1.8547400
	H	-0.7402490	-2.2036477	1.9306900
	H	3.0212940	4.3141213	0.0342800
	H	2.3225230	3.3005013	-1.2747100
	H	-2.2037570	2.2064033	1.5561600
	H	-1.5645880	0.8349223	2.5065800
	H	2.1057530	1.5285113	0.2106000
	H	1.3310240	3.7155913	0.1448300
	H	2.2353720	0.2448813	-4.1833500
	H	1.9660220	-1.4867587	-4.5547600
	H	3.0238720	-1.0059187	-3.1877300
	H	0.0246610	-2.5797877	-1.0434600
	C	1.5614910	-1.9882187	0.3747500
	C	4.2530510	-1.8714397	1.2014500
	C	1.9093920	-1.4003987	1.6035600
	C	2.5893810	-2.4889387	-0.4413400
	C	3.9219610	-2.4316897	-0.0344400
	C	3.2406320	-1.3555787	2.0170600
	H	1.1375020	-0.9726687	2.2343700
	H	2.3340910	-2.9343887	-1.3991800
	H	4.6997610	-2.8357697	-0.6769500
	H	3.4887120	-0.9264397	2.9848300
	H	5.2885310	-1.8427297	1.5304600
	C	2.7621730	2.3936313	1.9686700
	C	4.1492530	1.8194003	0.0041600
	H	4.1023730	1.6842003	-1.0749300
	H	4.8846730	2.5820503	0.2709200
	H	4.3960520	0.8678203	0.4765900
	H	3.4378530	3.1968703	2.2711200
	H	3.0741030	1.4488313	2.4148100
	S	-5.9807170	1.5972343	-0.5052900
	H	1.7374230	2.6159213	2.2629600

Name	$TS_{Theo1S-BMA-S33}$
Absolute Energies (Hartree)	There are 13 positive frequencies below 100 cm^-1.<br><br>Zero-point correction: 0.520018 hartree<br>Enthalpy correction: 0.555948 hartree<br>Free Energy correction: 0.454647 hartree<br>Quasiharmonic Free Energy correction: 0.461641 hartree<br><br>SCF Energy: -1811.847824 hartree<br>SCF Energy*: -1812.290689 hartree<br>SCF Energy + ZPVE: -1811.327806 hartree<br>Enthalpy: -1811.291876 hartree<br>Free Energy: -1811.393176 hartree<br><br>Free Energy with quasiharmonic correction: -1811.386183 hartree (correction: 4.39 kcal/mol)<br><br>---<br>Low frequencies before projection:<br>-199.0591<br>24.0861<br>28.4521<br><br>Low frequencies after projection:<br>-199.0577<br>23.5869<br>28.2977<br>---
Cartesian Coordinates	N -0.7336137 2.6048677 -0.1673510<br>N -4.2204337 -0.1479323 1.3758890<br>C -4.7932137 -0.9881723 0.4798090<br>N -3.9929747 -2.0024723 0.0754390<br>N -0.6264337 -0.9474123 1.2727590<br>O -1.3743837 -0.1792223 1.9619990<br>O -1.1136347 -1.9118823 0.6194590<br>C 0.7252863 -0.7239323 1.3018590<br>C 1.6049353 -1.4902223 0.5370190<br>C 1.7233263 -0.5605423 -1.5288410<br>C 0.3164963 -0.5580123 -1.8321310<br>O -0.5066737 0.3113077 -1.5192510<br>O -0.0681347 -1.6795423 -2.4821010<br>C -1.4851047 -1.8193423 -2.6739410<br>C 2.3925863 0.6636777 -1.1608310<br>O 1.8880763 1.6556077 -0.6268710<br>O 3.7275063 0.5890867 -1.3981910<br>C 4.5032063 1.7120867 -0.9648110<br>C -4.3786147 -3.0143823 -0.8877010<br>C -4.8886437 0.9898777 1.9731490<br>C -2.1989137 2.3206577 -0.2113510

	H	2.3251663	-1.3173523	-2.0153910
	H	-5.2840047	-3.5396623	-0.5671410
	H	-3.5567647	-3.7317423	-0.9637710
	H	-4.5729147	-2.5846223	-1.8784610
	H	-3.0226947	-2.0061123	0.3952890
	H	-3.2560837	-0.3270723	1.6627590
	H	-5.7715337	0.6850477	2.5465090
	H	-4.1777037	1.4767077	2.6474590
	H	-5.2176237	1.7095677	1.2144290
	H	-1.6285747	-2.8260123	-3.0704610
	H	1.0135263	0.1094077	1.9212690
	H	-2.7442037	3.1929677	0.1564090
	H	-2.4796037	2.0975577	-1.2402810
	H	-1.8567037	-1.0784423	-3.3896310
	H	-2.0096047	-1.6955123	-1.7252210
	H	-0.2583137	1.7420677	-0.5284710
	H	-2.3968237	1.4490377	0.4103590
	H	4.2415863	2.6105267	-1.5342610
	H	5.5433363	1.4393767	-1.1516310
	H	4.3536663	1.9087167	0.1002190
	H	1.1801753	-2.3764923	0.0784690
	C	3.0492953	-1.5463433	0.8234990
	C	5.8187953	-1.7267433	1.2946790
	C	3.7114663	-0.5748533	1.5945590
	C	3.8033153	-2.6077233	0.2936190
	C	5.1728253	-2.6993333	0.5271290
	C	5.0819863	-0.6660233	1.8274790
	H	3.1591463	0.2634967	2.0064390
	H	3.3044953	-3.3619433	-0.3102210
	H	5.7368153	-3.5292233	0.1101890
	H	5.5766463	0.0939267	2.4266190
	H	6.8876253	-1.7958733	1.4779090
	C	-0.2610137	2.8580977	1.2239190
	C	-0.3490837	3.7037777	-1.0965110
	H	-0.6574337	3.4265677	-2.1061510
	H	-0.8490737	4.6257977	-0.7899510
	H	0.7339663	3.8166977	-1.0639010
	H	-0.7228037	3.7774377	1.5930490
	H	0.8240363	2.9521477	1.2026190
	H	-0.5569437	2.0086677	1.8414190
	S	-6.3890137	-0.7806113	-0.0888510

Name	$TS_{Theo2S-BMB-R1}$
**Absolute Energies (Hartree)**	There are 15 positive frequencies below 100 cm^-1.<br><br>Zero-point correction: 0.529708 hartree<br>Enthalpy correction: 0.567971 hartree<br>Free Energy correction: 0.459581 hartree<br>Quasiharmonic Free Energy correction: 0.469016 hartree<br><br>SCF Energy: -1980.332588 hartree<br>SCF Energy + ZPVE: -1979.802880 hartree<br>Enthalpy: -1979.764617 hartree<br>Free Energy: -1979.873007 hartree<br><br>Free Energy with quasiharmonic correction: -1979.863572 hartree (correction: 5.92 kcal/mol)<br><br>---<br>Low frequencies before projection:<br>-144.7729<br>14.0876<br>23.4218<br><br>Low frequencies after projection:<br>-144.7727<br>13.885<br>23.3408<br>---
**Cartesian Coordinates**	C -4.9863290 1.3575775 -0.1712902<br>S -6.3672490 2.0290375 0.5851598<br>N -5.0338890 0.1887775 -0.8531102<br>N -3.7673990 1.9393175 -0.1146702<br>H -2.9624390 1.4485775 -0.5039102<br>C -6.2197700 -0.6259225 -1.0025002<br>H -4.1549200 -0.1842925 -1.2042302<br>H -6.6089800 -0.9647025 -0.0347002<br>N 4.1966610 0.4648555 -1.0187802<br>C 4.0227610 0.8882655 -2.3983002<br>H 2.9526110 1.0142455 -2.5642002<br>H 4.5168410 1.8494555 -2.5765502<br>H 4.4134810 0.1482655 -3.1083702<br>H 0.8751200 -0.9154735 -1.3375702<br>C -1.2731700 -0.8838535 -1.5892002<br>C -0.0302500 -0.3816135 -1.0834102<br>O -2.3520400 -0.2916535 -1.6538702<br>C 0.0996210 0.9669765 -0.6117002<br>O -0.7767590 1.7678265 -0.3044202<br>C -0.0700100 -1.4005535 1.0752798<br>C 0.8452500 -0.6702035 1.8303298<br>C -1.4971200 -1.4026735 -1.3868898

	H	0.3119000	-2.2480035	0.5189898
	N	2.1742000	-0.7027045	1.5299298
	H	0.5836900	0.0210765	2.6169798
	O	2.6127700	-1.3913045	0.5617098
	O	2.9830300	0.0029755	2.2261098
	C	-2.2602300	-2.5485235	-1.0915898
	C	-2.1418800	-0.2829435	1.9462498
	C	-3.5034900	-0.3230125	2.2283198
	C	-3.6218900	-2.5867325	1.3807698
	C	-4.2460500	-1.4738925	1.9514598
	H	-1.7732900	-3.4069435	0.6368998
	H	-1.5845190	0.6325565	2.1123898
	H	-4.0006290	0.5541475	2.6302198
	H	-4.1978700	-3.4804925	1.1559398
	H	-5.3113600	-1.4905525	2.1619998
	H	-7.0212000	-0.0793715	-1.5113602
	H	-5.9496600	-1.5002225	-1.6025902
	C	-3.4829890	3.1567975	0.6160898
	H	-3.6887790	3.0452675	1.6887998
	H	-2.4249390	3.3805865	0.4731998
	H	-4.0901890	3.9913475	0.2495998
	C	5.3830710	0.3329245	-0.4163002
	C	7.7632600	0.0124645	1.0396798
	C	6.6368210	0.5730845	-1.0390802
	N	5.3951800	-0.0366555	0.8985198
	C	6.5348500	-0.2002255	1.6126998
	C	7.7978010	0.4121845	-0.3142302
	H	6.6621410	0.8649845	-2.0811602
	H	6.3926200	-0.4990855	2.6447498
	H	8.7537610	0.5891945	-0.7984402
	H	8.6695200	-0.1221655	1.6169098
	O	-1.1574300	-2.1848935	-1.9895302
	O	1.4320310	1.2797655	-0.4290102
	C	-2.3614700	-2.7849835	-2.4833102
	H	-2.7393100	-2.2484025	-3.3596902
	H	-2.0891500	-3.8059535	-2.7590102
	H	-3.1375800	-2.7998025	-1.7119802
	C	1.6936510	2.5351155	0.2074198
	H	2.7788010	2.5960755	0.3042598
	H	1.3196410	3.3676455	-0.3965302
	H	1.2276410	2.5772165	1.1962398
	H	4.4768500	-0.1500945	1.3931998
	H	3.3786510	0.0793155	-0.5455802

Name	TS$_{Theo2S-BMB-R1\textit{conf}}$
**Absolute Energies (Hartree)**	There are 15 positive frequencies below 100 cm^-1.<br><br>Zero-point correction: 0.529706 hartree<br>Enthalpy correction: 0.567970 hartree<br>Free Energy correction: 0.459583 hartree<br>Quasiharmonic Free Energy correction: 0.469011 hartree<br><br>SCF Energy: -1980.332588 hartree<br>SCF Energy + ZPVE: -1979.802882 hartree<br>Enthalpy: -1979.764618 hartree<br>Free Energy: -1979.873005 hartree<br><br>Free Energy with quasiharmonic correction: -1979.863576 hartree (correction: 5.92 kcal/mol)<br><br>---<br>Low frequencies before projection:<br>-144.5303<br>14.1635<br>23.4424<br><br>Low frequencies after projection:<br>-144.5301<br>13.9708<br>23.3613<br>---
**Cartesian Coordinates**	C -4.9860328 1.3576911 -0.1716185<br>S -6.3668628 2.0294111 0.5847715<br>N -5.0336528 0.1886811 -0.8530485<br>N -3.7670828 1.9394311 -0.1153085<br>H -2.9621428 1.4485911 -0.5044885<br>C -6.2195728 -0.6259989 -1.0022285<br>H -4.1547128 -0.1845289 -1.2040885<br>H -6.6087128 -0.9646389 -0.0343485<br>N 4.1967172 0.4645601 -1.0187485<br>C 4.0228972 0.8872601 -2.3984985<br>H 2.9527772 1.0134701 -2.5644285<br>H 4.5172572 1.8482201 -2.5772585<br>H 4.4133972 0.1467801 -3.1081785<br>H 0.8750272 -0.9162499 -1.3372885<br>C -1.2732028 -0.8848989 -1.5890785<br>C -0.0303628 -0.3822199 -1.0835985<br>O -2.3521428 -0.2928789 -1.6541885<br>C 0.0994172 0.9666401 -0.6126885<br>O -0.7770028 1.7677101 -0.3060685<br>C -0.0701828 -1.3999499 1.0760315<br>C 0.8449872 -0.6690399 1.8306115<br>C -1.4973028 -1.4019889 1.3874915

	H	0.3118272	-2.2476199	0.5201515
	N	2.1739672	-0.7016899	1.5302915
	H	0.5833672	0.0227201	2.6168115
	O	2.6126172	-1.3908599	0.5625315
	O	2.9827272	0.0044201	2.2261015
	C	-2.2603328	-2.5479989	1.0925915
	C	-2.1421728	-0.2820789	1.9464015
	C	-3.5037928	-0.3221289	2.2284015
	C	-3.6220028	-2.5861789	1.3817015
	C	-4.2462628	-1.4731589	1.9519315
	H	-1.7733228	-3.4065589	0.6382415
	H	-1.5848728	0.6335211	2.1122415
	H	-4.0010328	0.5551611	2.6299115
	H	-4.1979228	-3.4800589	1.1571715
	H	-5.3115828	-1.4897989	2.1624215
	H	-7.0210328	-0.0794989	-1.5110885
	H	-5.9495428	-1.5003889	-1.6022085
	C	-3.4825518	3.1571411	0.6150215
	H	-3.6880418	3.0459111	1.6878215
	H	-2.4245618	3.3809411	0.4717815
	H	-4.0898918	3.9915311	0.2484515
	C	5.3831072	0.3327701	-0.4161985
	C	7.7632172	0.0126901	1.0399915
	C	6.6368972	0.5725001	-1.0390485
	N	5.3951372	-0.0362299	0.8987915
	C	6.5347672	-0.1996099	1.6130815
	C	7.7978372	0.4118101	-0.3140885
	H	6.6622672	0.8638801	-2.0812785
	H	6.3924772	-0.4979999	2.6452515
	H	8.7538372	0.5884801	-0.7983485
	H	8.6694372	-0.1217799	1.6173115
	O	-1.1572928	-2.1861989	-1.9885885
	O	1.4317972	1.2795701	-0.4299885
	C	-2.3612228	-2.7866889	-2.4821485
	H	-2.7390628	-2.2506189	-3.3588385
	H	-2.0887528	-3.8077689	-2.7572985
	H	-3.1373928	-2.8012089	-1.7108785
	C	1.6933272	2.5353301	0.2056715
	H	2.7784572	2.5962601	0.3028015
	H	1.3195682	3.3674801	-0.3989585
	H	1.2270272	2.5781401	1.1943215
	H	4.4767672	-0.1493199	1.3934615
	H	3.3787072	0.0791301	-0.5454585

Name	$TS_{Theo2S-BMB-R12}$
**Absolute Energies (Hartree)**	There are 15 positive frequencies below 100 cm^-1.<br><br>Zero-point correction: 0.529707 hartree<br>Enthalpy correction: 0.567969 hartree<br>Free Energy correction: 0.459602 hartree<br>Quasiharmonic Free Energy correction: 0.469013 hartree<br><br>SCF Energy: -1980.332588 hartree<br>SCF Energy + ZPVE: -1979.802881 hartree<br>Enthalpy: -1979.764619 hartree<br>Free Energy: -1979.872986 hartree<br><br>Free Energy with quasiharmonic correction: -1979.863575 hartree (correction: 5.91 kcal/mol)<br><br>---<br>Low frequencies before projection:<br>-144.4105<br>14.2913<br>23.4440<br><br>Low frequencies after projection:<br>-144.4102<br>14.1115<br>23.3628<br>---
**Cartesian Coordinates**	C -4.9859422 1.3576873 -0.1720557<br>S -6.3665612 2.0294373 0.5847043<br>N -5.0337222 0.1886373 -0.8533957<br>N -3.7669712 1.9394373 -0.1161557<br>H -2.9621122 1.4483773 -0.5052257<br>C -6.2196122 -0.6261827 -1.0020857<br>H -4.1548222 -0.1845927 -1.2044957<br>H -6.6083822 -0.9647627 -0.0340357<br>N 4.1968778 0.4632563 -1.0190057<br>C 4.0230278 0.8854563 -2.3989057<br>H 2.9528478 1.0108263 -2.5650857<br>H 4.5167278 1.8467163 -2.5778457<br>H 4.4141978 0.1450463 -3.1082957<br>H 0.8747578 -0.9169637 -1.3372457<br>C -1.2734722 -0.8853427 -1.5888957<br>C -0.0305222 -0.3826437 -1.0837357<br>O -2.3522922 -0.2931427 -1.6542157<br>C 0.0995078 0.9663563 -0.6133057<br>O -0.7767522 1.7676473 -0.3068057<br>C -0.0703222 -1.3994537 1.0765443<br>C 0.8448778 -0.6682037 1.8307343<br>C -1.4974322 -1.4012827 1.3880043

	H	0.3116778	-2.2473637	0.5210143
	N	2.1738678	-0.7010737	1.5304743
	H	0.5832978	0.0239663	2.6165943
	O	2.6125078	-1.3907537	0.5630543
	O	2.9826478	0.0053063	2.2259843
	C	-2.2605122	-2.5474027	1.0936843
	C	-2.1422222	-0.2810727	1.9463843
	C	-3.5038422	-0.3209227	2.2284743
	C	-3.6221722	-2.5853927	1.3828943
	C	-4.2463622	-1.4720727	1.9526243
	H	-1.7735522	-3.4061927	0.6397243
	H	-1.5848822	0.6345873	2.1117243
	H	-4.0010322	0.5565873	2.6295443
	H	-4.1981322	-3.4793627	1.1588443
	H	-5.3116622	-1.4885727	2.1632143
	H	-7.0212922	-0.0798127	-1.5107357
	H	-5.9496922	-1.5006127	-1.6020657
	C	-3.4822012	3.1571273	0.6141143
	H	-3.6871112	3.0458273	1.6870143
	H	-2.4243012	3.3810673	0.4703443
	H	-4.0898212	3.9914673	0.2478943
	C	5.3832378	0.3321963	-0.4162557
	C	7.7632778	0.0135863	1.0403843
	C	6.6370278	0.5720163	-1.0390757
	N	5.3952278	-0.0360937	0.8989343
	C	6.5348278	-0.1987737	1.6134443
	C	7.7979278	0.4120263	-0.3138957
	H	6.6624378	0.8629163	-2.0814357
	H	6.3924978	-0.4966237	2.6457743
	H	8.7539378	0.5887563	-0.7981357
	H	8.6694678	-0.1203137	1.6178743
	O	-1.1578122	-2.1868327	-1.9878757
	O	1.4319678	1.2791463	-0.4309057
	C	-2.3618922	-2.7873227	-2.4810657
	H	-2.7396722	-2.2516327	-3.3580057
	H	-2.0896422	-3.8086127	-2.7557057
	H	-3.1380222	-2.8013027	-1.7097457
	C	1.6937588	2.5351063	0.2042343
	H	2.7789288	2.5960763	0.3008843
	H	1.3197388	3.3670663	-0.4004957
	H	1.2278788	2.5782363	1.1930643
	H	4.4768278	-0.1490337	1.3935743
	H	3.3789578	0.0777763	-0.5455657

Name	$TS_{Theo2S\text{-}BMB\text{-}R2}$
**Absolute Energies (Hartree)**	There are 14 positive frequencies below 100 cm^-1.<br><br>Zero-point correction: 0.530436 hartree<br>Enthalpy correction: 0.568045 hartree<br>Free Energy correction: 0.462347 hartree<br>Quasiharmonic Free Energy correction: 0.470712 hartree<br><br>SCF Energy: -1980.331241 hartree<br>SCF Energy + ZPVE: -1979.800805 hartree<br>Enthalpy: -1979.763196 hartree<br>Free Energy: -1979.868894 hartree<br><br>Free Energy with quasiharmonic correction: -1979.860529 hartree (correction: 5.25 kcal/mol)<br><br>---<br>Low frequencies before projection:<br>-132.2547<br>15.0253<br>27.7611<br><br>Low frequencies after projection:<br>-132.2539<br>14.7902<br>27.7206<br>---
**Cartesian Coordinates**	C -0.1583210 2.5383186 -0.2804211<br>S -1.3608810 3.1523786 0.7850989<br>N -0.4302310 1.8609386 -1.4092211<br>N 1.1588490 2.7167596 -0.0185911<br>H 1.8158690 2.3402396 -0.7020211<br>C -1.7539410 1.4929586 -1.8654111<br>H 0.3373590 1.3686496 -1.8715611<br>H -2.1333810 0.6151586 -1.3328811<br>N -4.1590210 1.4058586 0.5993289<br>C -4.9876410 2.6002186 0.5608689<br>H -4.3147010 3.4587186 0.5582389<br>H -5.6000210 2.6327186 -0.3482711<br>H -5.6440410 2.6645786 1.4370889<br>H 2.7126890 -2.0946804 -1.2391211<br>C 0.8359290 -1.3813004 -2.0301211<br>C 2.1537990 -1.1976804 -1.4684811<br>O 0.1726390 -0.5970314 -2.7026611<br>C 2.9131790 -0.0077004 -1.7208911<br>O 2.4796490 1.1227896 -1.9923911<br>C 1.6614090 -0.8854604 0.8392289<br>C 0.7911490 -1.9052404 1.1884689<br>C 3.0538890 -0.8694804 1.3094189

	H	1.2208090	0.0736496	0.5921889
	N	-0.5595910	-1.8051114	0.9705789
	H	1.0877790	-2.8603204	1.5947489
	O	-1.0470310	-0.7394214	0.4620489
	O	-1.3065110	-2.7633314	1.2943289
	C	3.6716490	0.3674696	1.5540889
	C	3.7975390	-2.0444304	1.5198189
	C	5.1139890	-1.9787704	1.9664989
	C	4.9875190	0.4332696	2.0086989
	C	5.7149390	-0.7402004	2.2146389
	H	3.1059290	1.2783096	1.3910389
	H	3.3505300	-3.0115104	1.3081689
	H	5.6772290	-2.8959804	2.1164689
	H	5.4448290	1.4008296	2.1986989
	H	6.7432990	-0.6928404	2.5626089
	H	-2.4467610	2.3276086	-1.7356711
	H	-1.6813510	1.2366386	-2.9232911
	C	1.6805590	3.5495296	1.0490089
	H	1.4183690	3.1522096	2.0354489
	H	2.7695590	3.5730896	0.9498189
	H	1.2996690	4.5749396	0.9851589
	C	-4.6526710	0.1652186	0.5562789
	C	-5.5434610	-2.5001614	0.4433789
	C	-6.0435210	-0.1259814	0.4906589
	N	-3.7942810	-0.8972414	0.5645389
	C	-4.2097210	-2.1890014	0.5133689
	C	-6.4686310	-1.4349814	0.4345189
	H	-6.7541510	0.6898986	0.4868089
	H	-3.4020210	-2.9096714	0.5431989
	H	-7.5333910	-1.6442424	0.3847389
	H	-5.8630200	-3.5340914	0.4028489
	O	0.3564690	-2.6230204	-1.7036811
	O	4.2477090	-0.2167504	-1.5498611
	C	-0.9998710	-2.8679014	-2.0819811
	H	-1.1264010	-2.8077714	-3.1679711
	H	-1.2278200	-3.8761014	-1.7303511
	H	-1.6661210	-2.1414814	-1.6055811
	C	5.0799290	0.9429696	-1.6447211
	H	6.0892390	0.5991896	-1.4104111
	H	5.0500090	1.3671796	-2.6538111
	H	4.7752390	1.7120496	-0.9285611
	H	-2.7545010	-0.7545814	0.5999889
	H	-3.1461910	1.5791386	0.6458089

Name	TS$_{Theo2S\text{-}BMB\text{-}R2\textit{conf}}$
Absolute Energies (Hartree)	There are 14 positive frequencies below 100 cm^-1.<br><br>Zero-point correction: 0.530183 hartree<br>Enthalpy correction: 0.568182 hartree<br>Free Energy correction: 0.461477 hartree<br>Quasiharmonic Free Energy correction: 0.469989 hartree<br><br>SCF Energy: -1980.338783 hartree<br>SCF Energy + ZPVE: -1979.808600 hartree<br>Enthalpy: -1979.770601 hartree<br>Free Energy: -1979.877306 hartree<br><br>Free Energy with quasiharmonic correction: -1979.868794 hartree (correction: 5.34 kcal/mol)<br><br>---<br>Low frequencies before projection:<br>-203.4883<br>19.0971<br>26.0536<br><br>Low frequencies after projection:<br>-203.4878<br>19.021<br>25.9703<br>---
Cartesian Coordinates	C -2.1302816 2.2842534 0.8601860<br>S -3.5720806 2.8747444 1.5616760<br>N -1.4407716 1.2336934 1.3708260<br>N -1.6120516 2.7934234 -0.2795840<br>H -0.7259816 2.4043424 -0.5935140<br>C -1.7238016 0.6206234 2.6545760<br>H -0.5453816 1.0172024 0.9412460<br>H -1.5950416 1.3288734 3.4828960<br>N -3.1269626 -0.0734566 -1.1291740<br>C -3.8914816 0.9364944 -1.8434140<br>H -3.1870116 1.5155034 -2.4407040<br>H -4.6256426 0.4776244 -2.5160840<br>H -4.4000016 1.6123444 -1.1461540<br>H 2.8165274 -0.1763686 -1.7776640<br>C 0.6681674 -0.1989476 -1.6569440<br>C 1.9770974 0.2219114 -1.2233840<br>O -0.3963326 0.4245624 -1.5522140<br>C 2.1405484 1.5213314 -0.6115940<br>O 1.3106584 2.1220614 0.0749160<br>C 2.4213174 -1.0733486 0.6120660<br>C 1.8329064 -2.3235786 0.4102460<br>C 3.8806774 -0.9065496 0.6776260

	H	1.8376974	-0.3634786	1.1862960
	N	0.4800164	-2.4590276	0.5232260
	H	2.3432264	-3.2142486	0.0770260
	O	-0.2162226	-1.4220676	0.8283560
	O	-0.0871836	-3.5611676	0.3220060
	C	4.4088874	0.1175904	1.4817260
	C	4.7696474	-1.7213596	-0.0459640
	C	6.1445774	-1.5228906	0.0460060
	C	5.7851874	0.3123804	1.5777760
	C	6.6578574	-0.5079006	0.8599360
	H	3.7286884	0.7601304	2.0347660
	H	4.3812764	-2.4991896	-0.6973940
	H	6.8192164	-2.1579806	-0.5218140
	H	6.1752484	1.1060994	2.2091860
	H	7.7315474	-0.3552706	0.9284960
	H	-2.7516326	0.2479934	2.6920860
	H	-1.0346326	-0.2168176	2.7769060
	C	-2.1058406	3.9773734	-0.9510440
	H	-2.0411706	4.8666434	-0.3120240
	H	-1.4926406	4.1334434	-1.8431540
	H	-3.1516206	3.8588734	-1.2517140
	C	-3.6752226	-0.9883256	-0.3283740
	C	-4.6194536	-3.0527456	1.3329860
	C	-5.0732226	-1.1941656	-0.1689740
	N	-2.8436526	-1.8006466	0.3864360
	C	-3.2790136	-2.8092066	1.1799560
	C	-5.5223336	-2.2082656	0.6474460
	H	-5.7659826	-0.5440756	-0.6871640
	H	-2.4863636	-3.3777466	1.6523360
	H	-6.5917636	-2.3581646	0.7644260
	H	-4.9639836	-3.8594056	1.9680760
	O	0.7296374	-1.4229176	-2.2408740
	O	3.3976984	2.0045514	-0.7898840
	C	-0.5085536	-2.0232776	-2.6392640
	H	-1.1600826	-1.3011276	-3.1382140
	H	-0.2403036	-2.8280276	-3.3263940
	H	-1.0177436	-2.4521276	-1.7696140
	C	3.7010894	3.2223704	-0.0973740
	H	4.7609694	3.4076204	-0.2810540
	H	3.1000094	4.0517814	-0.4840240
	H	3.5154994	3.1252004	0.9762960
	H	-1.8094326	-1.6280166	0.4075960
	H	-2.1050026	0.0208534	-1.1608640

Name	$TS_{Theo2S\text{-}BMB\text{-}R3}$
**Absolute Energies (Hartree)**	There are 14 positive frequencies below 100 cm^-1.<br><br>Zero-point correction: 0.530947 hartree<br>Enthalpy correction: 0.568708 hartree<br>Free Energy correction: 0.462338 hartree<br>Quasiharmonic Free Energy correction: 0.471240 hartree<br><br>SCF Energy: -1980.338373 hartree<br>SCF Energy + ZPVE: -1979.807426 hartree<br>Enthalpy: -1979.769665 hartree<br>Free Energy: -1979.876035 hartree<br><br>Free Energy with quasiharmonic correction: -1979.867132 hartree (correction: 5.59 kcal/mol)<br><br>---<br>Low frequencies before projection:<br>-195.1704<br>14.4479<br>25.7278<br><br>Low frequencies after projection:<br>-195.1703<br>13.8664<br>25.7168<br>---
**Cartesian Coordinates**	C 2.5294862 2.0940194 0.3781132<br>S 4.0892462 2.6756294 0.7575532<br>N 1.7990462 1.3167294 1.2276732<br>N 1.9308362 2.3300494 -0.8079568<br>H 1.0578362 1.8401294 -1.0068668<br>C 2.1408662 1.1511694 2.6299132<br>H 0.8058162 1.2456294 1.0036832<br>H 3.1260262 0.6882694 2.7333732<br>N 2.6544462 -0.9159706 -1.1089968<br>C 3.1025062 -0.4144406 -2.3981668<br>H 2.2299262 0.0013294 -2.8977068<br>H 3.8508962 0.3756094 -2.2754968<br>H 3.5256162 -1.2175506 -3.0144468<br>H -3.1431638 0.4870084 -1.6422568<br>C -1.9893738 1.5855584 -0.1770268<br>C -2.1716138 0.5663784 -1.1739768<br>O -0.9695138 1.8186184 0.4755732<br>C -1.0517038 0.0385084 -1.9097468<br>O 0.1357962 0.3736494 -1.8324968<br>C -2.5971238 -1.1939816 0.2726932<br>C -1.6611438 -1.1471816 1.3023432<br>C -4.0256038 -0.9422916 0.5064032

	H -2.3547638 -1.8293016 -0.5713968<br>N -0.3777138 -1.5685316 1.0934232<br>H -1.8262138 -0.7231016 2.2800932<br>O -0.0000438 -2.0327906 -0.0170768<br>O 0.4486062 -1.4888206 2.0638532<br>C -4.9629838 -1.4810916 -0.3932668<br>C -4.4963938 -0.1710816 1.5844232<br>C -5.8612638 0.0383184 1.7631732<br>C -6.3279238 -1.2723216 -0.2132068<br>C -6.7822438 -0.5121116 0.8676632<br>H -4.6088938 -2.0646016 -1.2396668<br>H -3.7941138 0.2786184 2.2788632<br>H -6.2077938 0.6378884 2.6006132<br>H -7.0372438 -1.7007716 -0.9160868<br>H -7.8467038 -0.3452616 1.0088032<br>H 2.1636762 2.1091594 3.1659432<br>H 1.3884462 0.4972694 3.0743332<br>C 2.5035062 3.1191494 -1.8773468<br>H 3.4653962 2.7168594 -2.2162668<br>H 1.7966262 3.1078794 -2.7117768<br>H 2.6704452 4.1555994 -1.5634468<br>C 3.4883462 -1.3726606 -0.1711568<br>C 5.1120362 -2.3191806 1.9222032<br>C 4.8918362 -1.5105706 -0.3567668<br>N 2.9873162 -1.7223406 1.0475232<br>C 3.7563562 -2.1789806 2.0664432<br>C 5.6768662 -1.9719606 0.6752632<br>H 5.3280962 -1.2384306 -1.3085368<br>H 3.2150362 -2.4108706 2.9760932<br>H 6.7483562 -2.0646006 0.5246332<br>H 5.7187162 -2.6794406 2.7436232<br>O -3.1543238 2.2435784 0.0819032<br>O -1.4468138 -0.9443416 -2.7634668<br>C -3.1094638 3.1735684 1.1701132<br>H -2.4310148 4.0041784 0.9496532<br>H -4.1307338 3.5424084 1.2837132<br>H -2.7781238 2.6890184 2.0940032<br>C -0.3939838 -1.6195416 -3.4618568<br>H -0.8849438 -2.3952716 -4.0529968<br>H 0.1447562 -0.9339906 -4.1240968<br>H 0.3078262 -2.0738406 -2.7561668<br>H 1.9693362 -1.6281906 1.2540132<br>H 1.6630062 -0.8163606 -0.8979668

Name	TS$_{Theo2S-BMB-R3}$*conf*
**Absolute Energies (Hartree)**	There are 14 positive frequencies below 100 cm^-1.<br><br>Zero-point correction: 0.530981 hartree<br>Enthalpy correction: 0.568857 hartree<br>Free Energy correction: 0.462257 hartree<br>Quasiharmonic Free Energy correction: 0.471107 hartree<br><br>SCF Energy: -1980.339123 hartree<br>SCF Energy + ZPVE: -1979.808142 hartree<br>Enthalpy: -1979.770266 hartree<br>Free Energy: -1979.876866 hartree<br><br>Free Energy with quasiharmonic correction: -1979.868016 hartree (correction: 5.55 kcal/mol)<br><br>--<br>Low frequencies before projection:<br>-187.6022<br>21.3730<br>25.9468<br><br>Low frequencies after projection:<br>-187.6021<br>21.0103<br>25.8801<br>--
**Cartesian Coordinates**	C -2.6295233 1.9344688 0.5638920<br>S -4.1961533 2.5979188 0.4270820<br>N -1.7821933 1.8091488 -0.4951380<br>N -2.1391333 1.4482388 1.7245920<br>H -1.2364333 0.9726988 1.7045620<br>C -2.0122033 2.4606788 -1.7723980<br>H -0.8059833 1.6377588 -0.2537280<br>H -2.9367433 2.0972688 -2.2288080<br>N -2.6029443 -1.3065912 -0.0448480<br>C -3.0267443 -1.6176312 1.2062720<br>H -2.2374143 -1.6583212 1.9438420<br>H 3.0339767 -0.4127722 1.8318920<br>C 1.8741567 1.2714078 1.1304420<br>C 2.0709867 -0.1163122 1.4397920<br>O 0.8590267 1.7994388 0.6706520<br>C 0.9608367 -0.9865412 1.7304520<br>O -0.2396433 -0.7033312 1.7979220<br>C 2.6332867 -0.8131922 -0.7121580<br>C 1.7893567 -0.1824122 -1.6219580<br>C 4.0721067 -0.5237422 -0.6610280<br>H 2.3281357 -1.7940922 -0.3675480<br>N 0.4976767 -0.5991512 -1.7830980

	H	2.0455467	0.6787178	-2.2189680
	O	0.0299857	-1.5600812	-1.0988680
	O	-0.2280933	-0.0139012	-2.6442980
	C	4.9412957	-1.5029522	-0.1467580
	C	4.6174867	0.6994878	-1.0915480
	C	5.9897567	0.9251778	-1.0258980
	C	6.3137857	-1.2767522	-0.0827780
	C	6.8433067	-0.0612122	-0.5240880
	H	4.5278657	-2.4446522	0.2059620
	H	3.9667567	1.4832978	-1.4652680
	H	6.3946967	1.8761278	-1.3613580
	H	6.9704057	-2.0470622	0.3124420
	H	7.9136767	0.1188468	-0.4725680
	H	-2.0935233	3.5508388	-1.6709380
	H	-1.1728733	2.2101988	-2.4231880
	C	-2.8688333	1.4184888	2.9744120
	H	-3.7966733	0.8403388	2.8889820
	H	-2.2218533	0.9524488	3.7230620
	H	-3.1325633	2.4281188	3.3077720
	C	-3.4444943	-1.1671512	-1.1124480
	C	-4.8282243	-1.3893502	-0.8790680
	N	-2.9227233	-0.8248712	-2.2919080
	H	-5.5261743	-1.2786302	-1.6982980
	O	3.0231367	1.9861278	1.2942620
	O	1.3914757	-2.2625322	1.9403020
	C	2.9678967	3.3506678	0.8625120
	H	2.2655067	3.9279678	1.4725820
	H	3.9805667	3.7392978	0.9866520
	H	2.6603667	3.4242678	-0.1854680
	C	0.3669957	-3.2397612	2.1486320
	H	0.8888057	-4.1889012	2.2867120
	H	-0.2259743	-3.0099112	3.0400920
	H	-0.2927243	-3.3016012	1.2773220
	H	-1.9175033	-0.6129712	-2.3540580
	H	-1.5916343	-1.1904912	-0.2059580
	C	-4.3567643	-1.8309112	1.4605120
	H	-4.6887843	-2.0713212	2.4628320
	C	-5.2644543	-1.7123802	0.3857420
	H	-6.3256843	-1.8668302	0.5570220
	C	-3.7230733	-0.5702912	-3.4772380
	H	-4.2600343	-1.4696712	-3.8022680
	H	-4.4473233	0.2361088	-3.3095080
	H	-3.0437633	-0.2672712	-4.2757480

Name	TS$_{Theo2S-BMB-R31}$
Absolute Energies (Hartree)	There are 14 positive frequencies below 100 cm^-1.<br><br>Zero-point correction: 0.530982 hartree<br>Enthalpy correction: 0.568858 hartree<br>Free Energy correction: 0.462268 hartree<br>Quasiharmonic Free Energy correction: 0.471108 hartree<br><br>SCF Energy: -1980.339123 hartree<br>SCF Energy + ZPVE: -1979.808141 hartree<br>Enthalpy: -1979.770265 hartree<br>Free Energy: -1979.876855 hartree<br><br>Free Energy with quasiharmonic correction: -1979.868015 hartree (correction: 5.55 kcal/mol)<br><br>---<br>Low frequencies before projection:<br>-187.5811<br>21.4327<br>25.9917<br><br>Low frequencies after projection:<br>-187.5809<br>21.0929<br>25.922<br>---
Cartesian Coordinates	C -2.6295608 1.9346234 0.5634808<br>S -4.1961208 2.5981844 0.4264708<br>N -1.7822008 1.8089734 -0.4954992<br>N -2.1392318 1.4486334 1.7243108<br>H -1.2365718 0.9730234 1.7043908<br>C -2.0121008 2.4602534 -1.7728992<br>H -0.8060318 1.6375134 -0.2540092<br>H -2.9365908 2.0967534 -2.2293392<br>N -2.6029418 -1.3065966 -0.0445492<br>C -3.0267718 -1.6172866 1.2066408<br>H -2.2374618 -1.6577766 1.9442408<br>H 3.0339282 -0.4123376 1.8320108<br>C 1.8741882 1.2716824 1.1300708<br>C 2.0709582 -0.1159576 1.4397908<br>O 0.8591392 1.7996124 0.6699808<br>C 0.9607882 -0.9861076 1.7306208<br>O -0.2396918 -0.7028566 1.7979608<br>C 2.6333082 -0.8133976 -0.7119992<br>C 1.7893882 -0.1828476 -1.6219592<br>C 4.0721282 -0.5239186 -0.6608992<br>H 2.3281482 -1.7942176 -0.3671492<br>N 0.4976982 -0.5996076 -1.7829892

	H	2.0455782	0.6781624	-2.2191492
	O	0.0300082	-1.5603766	-1.0985492
	O	-0.2280618	-0.0145366	-2.6443092
	C	4.9413182	-1.5030186	-0.1464192
	C	4.6174882	0.6992314	-1.0916592
	C	5.9897482	0.9249614	-1.0260192
	C	6.3137982	-1.2767686	-0.0824492
	C	6.8433082	-0.0613086	-0.5239892
	H	4.5278972	-2.4446486	0.2064908
	H	3.9667582	1.4829514	-1.4655492
	H	6.3946892	1.8758514	-1.3616692
	H	6.9704272	-2.0469886	0.3129508
	H	7.9136782	0.1187704	-0.4724892
	H	-2.0934308	3.5504334	-1.6716592
	H	-1.1727008	2.2096534	-2.4235592
	C	-2.8689718	1.4192234	2.9741108
	H	-3.7968218	0.8410544	2.8888208
	H	-2.2220118	0.9533834	3.7229108
	H	-3.1326908	2.4289534	3.3071908
	C	-3.4444718	-1.1674456	-1.1122092
	C	-4.8282018	-1.3895756	-0.8787992
	N	-2.9226818	-0.8254666	-2.2917592
	H	-5.5261318	-1.2790956	-1.6980792
	O	3.0231392	1.9864324	1.2939008
	O	1.3913872	-2.2620476	1.9408108
	C	2.9679792	3.3508724	0.8618108
	H	2.2654492	3.9283124	1.4715908
	H	3.9806292	3.7395414	0.9860808
	H	2.6606792	3.4242124	-0.1862492
	C	0.3668872	-3.2392076	2.1493208
	H	0.8886672	-4.1883276	2.2876308
	H	-0.2261028	-3.0091466	3.0407108
	H	-0.2928028	-3.3012366	1.2780008
	H	-1.9174618	-0.6135866	-2.3539392
	H	-1.5916318	-1.1905366	-0.2056692
	C	-4.3568018	-1.8305056	1.4609108
	H	-4.6888428	-2.0706256	2.4632908
	C	-5.2644618	-1.7122656	0.3860908
	H	-6.3257018	-1.8666656	0.5573908
	C	-3.7230118	-0.5711856	-3.4771592
	H	-4.4472718	0.2352344	-3.3096392
	H	-3.0436918	-0.2683566	-4.2757392
	H	-4.2599518	-1.4706556	-3.8019792

Name	$TS_{Theo2S-BMB-S1}$
**Absolute Energies (Hartree)**	There are 14 positive frequencies below 100 cm^-1.<br><br>Zero-point correction: 0.530436 hartree<br>Enthalpy correction: 0.568045 hartree<br>Free Energy correction: 0.462347 hartree<br>Quasiharmonic Free Energy correction: 0.470712 hartree<br><br>SCF Energy: -1980.331241 hartree<br>SCF Energy + ZPVE: -1979.800805 hartree<br>Enthalpy: -1979.763196 hartree<br>Free Energy: -1979.868894 hartree<br><br>Free Energy with quasiharmonic correction: -1979.860529 hartree (correction: 5.25 kcal/mol)<br><br>---<br>Low frequencies before projection:<br>-132.2547<br>15.0253<br>27.7611<br><br>Low frequencies after projection:<br>-132.2539<br>14.7902<br>27.7206<br>---
**Cartesian Coordinates**	C -0.1583210 2.5383186 0.2804211<br>S -1.3608810 3.1523786 -0.7850989<br>N -0.4302310 1.8609386 1.4092211<br>N 1.1588490 2.7167596 0.0185911<br>H 1.8158690 2.3402396 0.7020211<br>C -1.7539410 1.4929586 1.8654111<br>H 0.3373590 1.3686496 1.8715611<br>H -2.1333810 0.6151586 1.3328811<br>N -4.1590210 1.4058586 -0.5993289<br>C -4.9876410 2.6002186 -0.5608689<br>H -4.3147010 3.4587186 -0.5582389<br>H -5.6000210 2.6327186 0.3482711<br>H -5.6440410 2.6645786 -1.4370889<br>H 2.7126890 -2.0946804 1.2391211<br>C 0.8359290 -1.3813004 2.0301211<br>C 2.1537990 -1.1976804 1.4684811<br>O 0.1726390 -0.5970314 2.7026611<br>C 2.9131790 -0.0077004 1.7208911<br>O 2.4796490 1.1227896 1.9923911<br>C 1.6614090 -0.8854604 -0.8392289<br>C 0.7911490 -1.9052404 -1.1884689<br>C 3.0538890 -0.8694804 -1.3094189

	H	1.2208090	0.0736496	-0.5921889
	N	-0.5595910	-1.8051114	-0.9705789
	H	1.0877790	-2.8603204	-1.5947489
	O	-1.0470310	-0.7394214	-0.4620489
	O	-1.3065110	-2.7633314	-1.2943289
	C	3.6716490	0.3674696	-1.5540889
	C	3.7975390	-2.0444304	-1.5198189
	C	5.1139890	-1.9787704	-1.9664989
	C	4.9875190	0.4332696	-2.0086989
	C	5.7149390	-0.7402004	-2.2146389
	H	3.1059290	1.2783096	-1.3910389
	H	3.3505300	-3.0115104	-1.3081689
	H	5.6772290	-2.8959804	-2.1164689
	H	5.4448290	1.4008296	-2.1986989
	H	6.7432990	-0.6928404	-2.5626089
	H	-2.4467610	2.3276086	1.7356711
	H	-1.6813510	1.2366386	2.9232911
	C	1.6805590	3.5495296	-1.0490089
	H	1.4183690	3.1522096	-2.0354489
	H	2.7695590	3.5730896	-0.9498189
	H	1.2996690	4.5749396	-0.9851589
	C	-4.6526710	0.1652186	-0.5562789
	C	-5.5434610	-2.5001614	-0.4433789
	C	-6.0435210	-0.1259814	-0.4906589
	N	-3.7942810	-0.8972414	-0.5645389
	C	-4.2097210	-2.1890014	-0.5133689
	C	-6.4686310	-1.4349814	-0.4345189
	H	-6.7541510	0.6898986	-0.4868089
	H	-3.4020210	-2.9096714	-0.5431989
	H	-7.5333910	-1.6442424	-0.3847389
	H	-5.8630200	-3.5340914	-0.4028489
	O	0.3564690	-2.6230204	1.7036811
	O	4.2477090	-0.2167504	1.5498611
	C	-0.9998710	-2.8679014	2.0819811
	H	-1.1264010	-2.8077714	3.1679711
	H	-1.2278200	-3.8761014	1.7303511
	H	-1.6661210	-2.1414814	1.6055811
	C	5.0799290	0.9429696	1.6447211
	H	6.0892390	0.5991896	1.4104111
	H	5.0500090	1.3671796	2.6538111
	H	4.7752390	1.7120496	0.9285611
	H	-2.7545010	-0.7545814	-0.5999889
	H	-3.1461910	1.5791386	-0.6458089

Name	TS$_{Theo2S-BMB-S13}$
**Absolute Energies (Hartree)**	There are 14 positive frequencies below 100 cm^-1.<br><br>Zero-point correction: 0.530183 hartree<br>Enthalpy correction: 0.568182 hartree<br>Free Energy correction: 0.461477 hartree<br>Quasiharmonic Free Energy correction: 0.469989 hartree<br><br>SCF Energy: -1980.338783 hartree<br>SCF Energy + ZPVE: -1979.808600 hartree<br>Enthalpy: -1979.770601 hartree<br>Free Energy: -1979.877306 hartree<br><br>Free Energy with quasiharmonic correction: -1979.868794 hartree (correction: 5.34 kcal/mol)<br><br>---<br>Low frequencies before projection:<br>-203.4883<br>19.0971<br>26.0536<br><br>Low frequencies after projection:<br>-203.4878<br>19.021<br>25.9703<br>---
**Cartesian Coordinates**	C -2.1302816 2.2842534 -0.8601860<br>S -3.5720806 2.8747444 -1.5616760<br>N -1.4407716 1.2336934 -1.3708260<br>N -1.6120516 2.7934234 0.2795840<br>H -0.7259816 2.4043424 0.5935140<br>C -1.7238016 0.6206234 -2.6545760<br>H -0.5453816 1.0172024 -0.9412460<br>H -1.5950416 1.3288734 -3.4828960<br>N -3.1269626 -0.0734566 1.1291740<br>C -3.8914816 0.9364944 1.8434140<br>H -3.1870116 1.5155034 2.4407040<br>H -4.6256426 0.4776244 2.5160840<br>H -4.4000016 1.6123444 1.1461540<br>H 2.8165274 -0.1763686 1.7776640<br>C 0.6681674 -0.1989476 1.6569440<br>C 1.9770974 0.2219114 1.2233840<br>O -0.3963326 0.4245624 1.5522140<br>C 2.1405484 1.5213314 0.6115940<br>O 1.3106584 2.1220614 -0.0749160<br>C 2.4213174 -1.0733486 -0.6120660<br>C 1.8329064 -2.3235786 -0.4102460<br>C 3.8806774 -0.9065496 -0.6776260

	H	1.8376974	-0.3634786	-1.1862960
	N	0.4800164	-2.4590276	-0.5232260
	H	2.3432264	-3.2142486	-0.0770260
	O	-0.2162226	-1.4220676	-0.8283560
	O	-0.0871836	-3.5611676	-0.3220060
	C	4.4088874	0.1175904	-1.4817260
	C	4.7696474	-1.7213596	0.0459640
	C	6.1445774	-1.5228906	-0.0460060
	C	5.7851874	0.3123804	-1.5777760
	C	6.6578574	-0.5079006	-0.8599360
	H	3.7286884	0.7601304	-2.0347660
	H	4.3812764	-2.4991896	0.6973940
	H	6.8192164	-2.1579806	0.5218140
	H	6.1752484	1.1060994	-2.2091860
	H	7.7315474	-0.3552706	-0.9284960
	H	-2.7516326	0.2479934	-2.6920860
	H	-1.0346326	-0.2168176	-2.7769060
	C	-2.1058406	3.9773734	0.9510440
	H	-2.0411706	4.8666434	0.3120240
	H	-1.4926406	4.1334434	1.8431540
	H	-3.1516206	3.8588734	1.2517140
	C	-3.6752226	-0.9883256	0.3283740
	C	-4.6194536	-3.0527456	-1.3329860
	C	-5.0732226	-1.1941656	0.1689740
	N	-2.8436526	-1.8006466	-0.3864360
	C	-3.2790136	-2.8092066	-1.1799560
	C	-5.5223336	-2.2082656	-0.6474460
	H	-5.7659826	-0.5440756	0.6871640
	H	-2.4863636	-3.3777466	-1.6523360
	H	-6.5917636	-2.3581646	-0.7644260
	H	-4.9639836	-3.8594056	-1.9680760
	O	0.7296374	-1.4229176	2.2408740
	O	3.3976984	2.0045514	0.7898840
	C	-0.5085536	-2.0232776	2.6392640
	H	-1.1600826	-1.3011276	3.1382140
	H	-0.2403036	-2.8280276	3.3263940
	H	-1.0177436	-2.4521276	1.7696140
	C	3.7010894	3.2223704	0.0973740
	H	4.7609694	3.4076204	0.2810540
	H	3.1000094	4.0517814	0.4840240
	H	3.5154994	3.1252004	-0.9762960
	H	-1.8094326	-1.6280166	-0.4075960
	H	-2.1050026	0.0208534	1.1608640

Name	TS$_{Theo2S-BMB-S2}$
**Absolute Energies (Hartree)**	There are 15 positive frequencies below 100 cm^-1.<br><br>Zero-point correction: 0.529708 hartree<br>Enthalpy correction: 0.567971 hartree<br>Free Energy correction: 0.459581 hartree<br>Quasiharmonic Free Energy correction: 0.469016 hartree<br><br>SCF Energy: -1980.332588 hartree<br>SCF Energy + ZPVE: -1979.802880 hartree<br>Enthalpy: -1979.764617 hartree<br>Free Energy: -1979.873007 hartree<br><br>Free Energy with quasiharmonic correction: -1979.863572 hartree (correction: 5.92 kcal/mol)<br><br>---<br>Low frequencies before projection:<br>-144.7729<br>14.0876<br>23.4218<br><br>Low frequencies after projection:<br>-144.7727<br>13.885<br>23.3408<br>---
**Cartesian Coordinates**	C -4.9863290 1.3575775 0.1712902<br>S -6.3672490 2.0290375 -0.5851598<br>N -5.0338890 0.1887775 0.8531102<br>N -3.7673990 1.9393175 0.1146702<br>H -2.9624390 1.4485775 0.5039102<br>C -6.2197700 -0.6259225 1.0025002<br>H -4.1549200 -0.1842925 1.2042302<br>H -6.6089800 -0.9647025 0.0347002<br>N 4.1966610 0.4648555 1.0187802<br>C 4.0227610 0.8882655 2.3983002<br>H 2.9526110 1.0142455 2.5642002<br>H 4.5168410 1.8494555 2.5765502<br>H 4.4134810 0.1482655 3.1083702<br>H 0.8751200 -0.9154735 1.3375702<br>C -1.2731700 -0.8838535 1.5892002<br>C -0.0302500 -0.3816135 1.0834102<br>O -2.3520400 -0.2916535 1.6538702<br>C 0.0996210 0.9669765 0.6117002<br>O -0.7767590 1.7678265 0.3044202<br>C -0.0700100 -1.4005535 -1.0752798<br>C 0.8452500 -0.6702035 -1.8303298<br>C -1.4971200 -1.4026735 -1.3868898

	H	0.3119000	-2.2480035	-0.5189898
	N	2.1742000	-0.7027045	-1.5299298
	H	0.5836900	0.0210765	-2.6169798
	O	2.6127700	-1.3913045	-0.5617098
	O	2.9830300	0.0029755	-2.2261098
	C	-2.2602300	-2.5485235	-1.0915898
	C	-2.1418800	-0.2829435	-1.9462498
	C	-3.5034900	-0.3230125	-2.2283198
	C	-3.6218900	-2.5867325	-1.3807698
	C	-4.2460500	-1.4738925	-1.9514598
	H	-1.7732900	-3.4069435	-0.6368998
	H	-1.5845190	0.6325565	-2.1123898
	H	-4.0006290	0.5541475	-2.6302198
	H	-4.1978700	-3.4804925	-1.1559398
	H	-5.3113600	-1.4905525	-2.1619998
	H	-7.0212000	-0.0793715	1.5113602
	H	-5.9496600	-1.5002225	1.6025902
	C	-3.4829890	3.1567975	-0.6160898
	H	-3.6887790	3.0452675	-1.6887998
	H	-2.4249390	3.3805865	-0.4731998
	H	-4.0901890	3.9913475	-0.2495998
	C	5.3830710	0.3329245	0.4163002
	C	7.7632600	0.0124645	-1.0396798
	C	6.6368210	0.5730845	1.0390802
	N	5.3951800	-0.0366555	-0.8985198
	C	6.5348500	-0.2002255	-1.6126998
	C	7.7978010	0.4121845	0.3142302
	H	6.6621410	0.8649845	2.0811602
	H	6.3926200	-0.4990855	-2.6447498
	H	8.7537610	0.5891945	0.7984402
	H	8.6695200	-0.1221655	-1.6169098
	O	-1.1574300	-2.1848935	1.9895302
	O	1.4320310	1.2797655	0.4290102
	C	-2.3614700	-2.7849835	2.4833102
	H	-2.7393100	-2.2484025	3.3596902
	H	-2.0891500	-3.8059535	2.7590102
	H	-3.1375800	-2.7998025	1.7119802
	C	1.6936510	2.5351155	-0.2074198
	H	2.7788010	2.5960755	-0.3042598
	H	1.3196410	3.3676455	0.3965302
	H	1.2276410	2.5772165	-1.1962398
	H	4.4768500	-0.1500945	-1.3931998
	H	3.3786510	0.0793155	0.5455802

Name	$TS_{Theo2S\text{-}BMB\text{-}S2conf}$
**Absolute Energies (Hartree)**	There are 15 positive frequencies below 100 cm^-1.<br><br>Zero-point correction: 0.530159 hartree<br>Enthalpy correction: 0.568268 hartree<br>Free Energy correction: 0.460609 hartree<br>Quasiharmonic Free Energy correction: 0.469652 hartree<br><br>SCF Energy: -1980.332170 hartree<br>SCF Energy + ZPVE: -1979.802011 hartree<br>Enthalpy: -1979.763902 hartree<br>Free Energy: -1979.871561 hartree<br><br>Free Energy with quasiharmonic correction: -1979.862518 hartree (correction: 5.67 kcal/mol)<br><br>---<br>Low frequencies before projection:<br>-167.2690<br>18.4947<br>26.3455<br><br>Low frequencies after projection:<br>-167.2689<br>18.347<br>26.0313<br>---
**Cartesian Coordinates**	C 4.8741134 -1.5081658 -0.1642591<br>S 6.2660934 -1.9160658 -1.0723491<br>N 4.9275734 -0.6817558 0.9070309<br>N 3.6407534 -1.9671858 -0.4738291<br>H 2.8339134 -1.6145658 0.0406709<br>C 6.1289234 -0.0273758 1.3774209<br>H 4.0444734 -0.4119258 1.3336709<br>H 6.5460934 0.6564442 0.6276809<br>N -4.2920966 -0.3388758 0.5730109<br>C -4.1342266 -1.1642058 1.6364609<br>H -3.1259266 -1.2190558 2.0263809<br>H -0.9545766 0.5110042 1.5045409<br>C 1.1721534 0.2768542 1.8219109<br>C -0.0555666 0.0544642 1.1135409<br>O 2.2224434 -0.3582058 1.7184809<br>C -0.2089266 -1.0496358 0.2070509<br>O 0.6558834 -1.7413058 -0.3187691<br>C 0.1337034 1.7368442 -0.5265091<br>C -0.7466266 1.3574142 -1.5419091<br>C 1.5773234 1.8006642 -0.7489591<br>H -0.2663466 2.3621542 0.2622909<br>N -2.0893166 1.3315342 -1.3235491

	H	-0.4465866	0.9813342	-2.5084691
	O	-2.5665666	1.6667042	-0.1870791
	O	-2.8700166	0.9436742	-2.2478191
	C	2.3372934	2.7499342	-0.0395391
	C	2.2380334	0.9213042	-1.6274591
	C	3.6138834	1.0125642	-1.8122091
	C	3.7136834	2.8418242	-0.2309791
	C	4.3544734	1.9741142	-1.1197891
	H	1.8371634	3.4154042	0.6587909
	H	1.6786634	0.1371442	-2.1262391
	H	4.1218134	0.3108642	-2.4662791
	H	4.2872934	3.5858942	0.3153709
	H	5.4299234	2.0293142	-1.2610391
	H	6.9054334	-0.7557258	1.6331409
	H	5.8679234	0.5444542	2.2732009
	C	3.3530534	-2.8103858	-1.6156691
	H	3.6169734	-2.3190458	-2.5614191
	H	2.2822534	-3.0183458	-1.6033491
	H	3.9124634	-3.7506558	-1.5661591
	C	-5.4801566	-0.1616568	-0.0777391
	C	-6.6082466	-0.8596268	0.4301709
	N	-5.4892566	0.6433232	-1.1445491
	O	1.0772634	1.3565242	2.6518309
	O	-1.5380766	-1.2132958	-0.1217291
	C	2.2725734	1.6851442	3.3719309
	H	2.5854734	0.8566342	4.0155009
	H	2.0188134	2.5559142	3.9800209
	H	3.0879334	1.9321842	2.6853009
	C	-1.8031066	-2.1524758	-1.1701091
	H	-2.8779166	-2.1023458	-1.3497891
	H	-1.5157466	-3.1656958	-0.8720891
	H	-1.2614466	-1.8833258	-2.0812791
	H	-4.5771366	0.9449832	-1.5158891
	H	-3.4928566	0.2570842	0.2780209
	C	-5.1997766	-1.8683168	2.1370809
	H	-5.0697566	-2.5311968	2.9833609
	C	-6.4569466	-1.6936768	1.5171109
	H	-7.3214766	-2.2312368	1.8957109
	H	-7.5694866	-0.7360268	-0.0518391
	C	-6.6735266	0.8958432	-1.9480591
	H	-7.0676366	-0.0250468	-2.3970091
	H	-6.3925366	1.5800132	-2.7502391
	H	-7.4639566	1.3668532	-1.3527391

Name	$TS_{Theo2S\text{-}BMB\text{-}S21}$
**Absolute Energies (Hartree)**	There are 15 positive frequencies below 100 cm^-1.<br><br>Zero-point correction: 0.529707 hartree<br>Enthalpy correction: 0.567969 hartree<br>Free Energy correction: 0.459602 hartree<br>Quasiharmonic Free Energy correction: 0.469013 hartree<br><br>SCF Energy: -1980.332588 hartree<br>SCF Energy + ZPVE: -1979.802881 hartree<br>Enthalpy: -1979.764619 hartree<br>Free Energy: -1979.872986 hartree<br><br>Free Energy with quasiharmonic correction: -1979.863575 hartree (correction: 5.91 kcal/mol)<br><br>---<br>Low frequencies before projection:<br>-144.4105<br>14.2913<br>23.4440<br><br>Low frequencies after projection:<br>-144.4102<br>14.1115<br>23.3628<br>---
**Cartesian Coordinates**	C -4.9859422 1.3576873 0.1720557<br>S -6.3665612 2.0294373 -0.5847043<br>N -5.0337222 0.1886373 0.8533957<br>N -3.7669712 1.9394373 0.1161557<br>H -2.9621122 1.4483773 0.5052257<br>C -6.2196122 -0.6261827 1.0020857<br>H -4.1548222 -0.1845927 1.2044957<br>H -6.6083822 -0.9647627 0.0340357<br>N 4.1968778 0.4632563 1.0190057<br>C 4.0230278 0.8854563 2.3989057<br>H 2.9528478 1.0108263 2.5650857<br>H 4.5167278 1.8467163 2.5778457<br>H 4.4141978 0.1450463 3.1082957<br>H 0.8747578 -0.9169637 1.3372457<br>C -1.2734722 -0.8853427 1.5888957<br>C -0.0305222 -0.3826437 1.0837357<br>O -2.3522922 -0.2931427 1.6542157<br>C 0.0995078 0.9663563 0.6133057<br>O -0.7767522 1.7676473 0.3068057<br>C -0.0703222 -1.3994537 -1.0765443<br>C 0.8448778 -0.6682037 -1.8307343<br>C -1.4974322 -1.4012827 -1.3880043

	H	0.3116778	-2.2473637	-0.5210143
	N	2.1738678	-0.7010737	-1.5304743
	H	0.5832978	0.0239663	-2.6165943
	O	2.6125078	-1.3907537	-0.5630543
	O	2.9826478	0.0053063	-2.2259843
	C	-2.2605122	-2.5474027	-1.0936843
	C	-2.1422222	-0.2810727	-1.9463843
	C	-3.5038422	-0.3209227	-2.2284743
	C	-3.6221722	-2.5853927	-1.3828943
	C	-4.2463622	-1.4720727	-1.9526243
	H	-1.7735522	-3.4061927	-0.6397243
	H	-1.5848822	0.6345873	-2.1117243
	H	-4.0010322	0.5565873	-2.6295443
	H	-4.1981322	-3.4793627	-1.1588443
	H	-5.3116622	-1.4885727	-2.1632143
	H	-7.0212922	-0.0798127	1.5107357
	H	-5.9496922	-1.5006127	1.6020657
	C	-3.4822012	3.1571273	-0.6141143
	H	-3.6871112	3.0458273	-1.6870143
	H	-2.4243012	3.3810673	-0.4703443
	H	-4.0898212	3.9914673	-0.2478943
	C	5.3832378	0.3321963	0.4162557
	C	7.7632778	0.0135863	-1.0403843
	C	6.6370278	0.5720163	1.0390757
	N	5.3952278	-0.0360937	-0.8989343
	C	6.5348278	-0.1987737	-1.6134443
	C	7.7979278	0.4120263	0.3138957
	H	6.6624378	0.8629163	2.0814357
	H	6.3924978	-0.4966237	-2.6457743
	H	8.7539378	0.5887563	0.7981357
	H	8.6694678	-0.1203137	-1.6178743
	O	-1.1578122	-2.1868327	1.9878757
	O	1.4319678	1.2791463	0.4309057
	C	-2.3618922	-2.7873227	2.4810657
	H	-2.7396722	-2.2516327	3.3580057
	H	-2.0896422	-3.8086127	2.7557057
	H	-3.1380222	-2.8013027	1.7097457
	C	1.6937588	2.5351063	-0.2042343
	H	2.7789288	2.5960763	-0.3008843
	H	1.3197388	3.3670663	0.4004957
	H	1.2278788	2.5782363	-1.1930643
	H	4.4768278	-0.1490337	-1.3935743
	H	3.3789578	0.0777763	0.5455657

<table>
<tr><td>Name</td><td>TS$_{Theo2S-BMB-S23}$</td></tr>
<tr><td>Absolute Energies (Hartree)</td><td>There are 15 positive frequencies below 100 cm^-1.

Zero-point correction: 0.529706 hartree
Enthalpy correction: 0.567970 hartree
Free Energy correction: 0.459583 hartree
Quasiharmonic Free Energy correction: 0.469011 hartree

SCF Energy: -1980.332588 hartree
SCF Energy + ZPVE: -1979.802882 hartree
Enthalpy: -1979.764618 hartree
Free Energy: -1979.873005 hartree

Free Energy with quasiharmonic correction: -1979.863576 hartree (correction: 5.92 kcal/mol)

Low frequencies before projection:
-144.5303
14.1635
23.4424

Low frequencies after projection:
-144.5301
13.9708
23.3613
---</td></tr>
<tr><td>Cartesian Coordinates</td><td>C -4.9860328 1.3576911 0.1716185
S -6.3668628 2.0294111 -0.5847715
N -5.0336528 0.1886811 0.8530485
N -3.7670828 1.9394311 0.1153085
H -2.9621428 1.4485911 0.5044885
C -6.2195728 -0.6259989 1.0022285
H -4.1547128 -0.1845289 1.2040885
H -6.6087128 -0.9646389 0.0343485
N 4.1967172 0.4645601 1.0187485
C 4.0228972 0.8872601 2.3984985
H 2.9527772 1.0134701 2.5644285
H 4.5172572 1.8482201 2.5772585
H 4.4133972 0.1467801 3.1081785
H 0.8750272 -0.9162499 1.3372885
C -1.2732028 -0.8848989 1.5890785
C -0.0303628 -0.3822199 1.0835985
O -2.3521428 -0.2928789 1.6541885
C 0.0994172 0.9666401 0.6126885
O -0.7770028 1.7677101 0.3060685
C -0.0701828 -1.3999499 -1.0760315
C 0.8449872 -0.6690399 -1.8306115
C -1.4973028 -1.4019889 -1.3874915</td></tr>
</table>

	H	0.3118272	-2.2476199	-0.5201515
	N	2.1739672	-0.7016899	-1.5302915
	H	0.5833672	0.0227201	-2.6168115
	O	2.6126172	-1.3908599	-0.5625315
	O	2.9827272	0.0044201	-2.2261015
	C	-2.2603328	-2.5479989	-1.0925915
	C	-2.1421728	-0.2820789	-1.9464015
	C	-3.5037928	-0.3221289	-2.2284015
	C	-3.6220028	-2.5861789	-1.3817015
	C	-4.2462628	-1.4731589	-1.9519315
	H	-1.7733228	-3.4065589	-0.6382415
	H	-1.5848728	0.6335211	-2.1122415
	H	-4.0010328	0.5551611	-2.6299115
	H	-4.1979228	-3.4800589	-1.1571715
	H	-5.3115828	-1.4897989	-2.1624215
	H	-7.0210328	-0.0794989	1.5110885
	H	-5.9495428	-1.5003889	1.6022085
	C	-3.4825518	3.1571411	-0.6150215
	H	-3.6880418	3.0459111	-1.6878215
	H	-2.4245618	3.3809411	-0.4717815
	H	-4.0898918	3.9915311	-0.2484515
	C	5.3831072	0.3327701	0.4161985
	C	7.7632172	0.0126901	-1.0399915
	C	6.6368972	0.5725001	1.0390485
	N	5.3951372	-0.0362299	-0.8987915
	C	6.5347672	-0.1996099	-1.6130815
	C	7.7978372	0.4118101	0.3140885
	H	6.6622672	0.8638801	2.0812785
	H	6.3924772	-0.4979999	-2.6452515
	H	8.7538372	0.5884801	0.7983485
	H	8.6694372	-0.1217799	-1.6173115
	O	-1.1572928	-2.1861989	1.9885885
	O	1.4317972	1.2795701	0.4299885
	C	-2.3612228	-2.7866889	2.4821485
	H	-2.7390628	-2.2506189	3.3588385
	H	-2.0887528	-3.8077689	2.7572985
	H	-3.1373928	-2.8012089	1.7108785
	C	1.6933272	2.5353301	-0.2056715
	H	2.7784572	2.5962601	-0.3028015
	H	1.3195682	3.3674801	0.3989585
	H	1.2270272	2.5781401	-1.1943215
	H	4.4767672	-0.1493199	-1.3934615
	H	3.3787072	0.0791301	0.5454585

<table>
<tr><td>Name</td><td>TS$_{Theo2S\text{-}BMB\text{-}S3}$</td></tr>
<tr><td>Absolute Energies (Hartree)</td><td>There are 14 positive frequencies below 100 cm^-1.

Zero-point correction: 0.530947 hartree
Enthalpy correction: 0.568708 hartree
Free Energy correction: 0.462338 hartree
Quasiharmonic Free Energy correction: 0.471240 hartree

SCF Energy: -1980.338373 hartree
SCF Energy + ZPVE: -1979.807426 hartree
Enthalpy: -1979.769665 hartree
Free Energy: -1979.876035 hartree

Free Energy with quasiharmonic correction: -1979.867132 hartree (correction: 5.59 kcal/mol)

Low frequencies before projection:
-195.1704
14.4479
25.7278

Low frequencies after projection:
-195.1703
13.8664
25.7168
---</td></tr>
<tr><td>Cartesian Coordinates</td><td>C 2.5294862 2.0940194 -0.3781132
S 4.0892462 2.6756294 -0.7575532
N 1.7990462 1.3167294 -1.2276732
N 1.9308362 2.3300494 0.8079568
H 1.0578362 1.8401294 1.0068668
C 2.1408662 1.1511694 -2.6299132
H 0.8058162 1.2456294 -1.0036832
H 3.1260262 0.6882694 -2.7333732
N 2.6544462 -0.9159706 1.1089968
C 3.1025062 -0.4144406 2.3981668
H 2.2299262 0.0013294 2.8977068
H 3.8508962 0.3756094 2.2754968
H 3.5256162 -1.2175506 3.0144468
H -3.1431638 0.4870084 1.6422568
C -1.9893738 1.5855584 0.1770268
C -2.1716138 0.5663784 1.1739768
O -0.9695138 1.8186184 -0.4755732
C -1.0517038 0.0385084 1.9097468
O 0.1357962 0.3736494 1.8324968
C -2.5971238 -1.1939816 -0.2726932
C -1.6611438 -1.1471816 -1.3023432
C -4.0256038 -0.9422916 -0.5064032</td></tr>
</table>

	H	-2.3547638	-1.8293016	0.5713968
	N	-0.3777138	-1.5685316	-1.0934232
	H	-1.8262138	-0.7231016	-2.2800932
	O	-0.0000438	-2.0327906	0.0170768
	O	0.4486062	-1.4888206	-2.0638532
	C	-4.9629838	-1.4810916	0.3932668
	C	-4.4963938	-0.1710816	-1.5844232
	C	-5.8612638	0.0383184	-1.7631732
	C	-6.3279238	-1.2723216	0.2132068
	C	-6.7822438	-0.5121116	-0.8676632
	H	-4.6088938	-2.0646016	1.2396668
	H	-3.7941138	0.2786184	-2.2788632
	H	-6.2077938	0.6378884	-2.6006132
	H	-7.0372438	-1.7007716	0.9160868
	H	-7.8467038	-0.3452616	-1.0088032
	H	2.1636762	2.1091594	-3.1659432
	H	1.3884462	0.4972694	-3.0743332
	C	2.5035062	3.1191494	1.8773468
	H	3.4653962	2.7168594	2.2162668
	H	1.7966262	3.1078794	2.7117768
	H	2.6704452	4.1555994	1.5634468
	C	3.4883462	-1.3726606	0.1711568
	C	5.1120362	-2.3191806	-1.9222032
	C	4.8918362	-1.5105706	0.3567668
	N	2.9873162	-1.7223406	-1.0475232
	C	3.7563562	-2.1789806	-2.0664432
	C	5.6768662	-1.9719606	-0.6752632
	H	5.3280962	-1.2384306	1.3085368
	H	3.2150362	-2.4108706	-2.9760932
	H	6.7483562	-2.0646006	-0.5246332
	H	5.7187162	-2.6794406	-2.7436232
	O	-3.1543238	2.2435784	-0.0819032
	O	-1.4468138	-0.9443416	2.7634668
	C	-3.1094638	3.1735684	-1.1701132
	H	-2.4310148	4.0041784	-0.9496532
	H	-4.1307338	3.5424084	-1.2837132
	H	-2.7781238	2.6890184	-2.0940032
	C	-0.3939838	-1.6195416	3.4618568
	H	-0.8849438	-2.3952716	4.0529968
	H	0.1447562	-0.9339906	4.1240968
	H	0.3078262	-2.0738406	2.7561668
	H	1.9693362	-1.6281906	-1.2540132
	H	1.6630062	-0.8163606	0.8979668

Name	TS$_{Theo2S\text{-}BMB\text{-}S3conf}$
**Absolute Energies (Hartree)**	There are 14 positive frequencies below 100 cm^-1.<br><br>Zero-point correction: 0.530981 hartree<br>Enthalpy correction: 0.568857 hartree<br>Free Energy correction: 0.462257 hartree<br>Quasiharmonic Free Energy correction: 0.471107 hartree<br><br>SCF Energy: -1980.339123 hartree<br>SCF Energy + ZPVE: -1979.808142 hartree<br>Enthalpy: -1979.770266 hartree<br>Free Energy: -1979.876866 hartree<br><br>Free Energy with quasiharmonic correction: -1979.868016 hartree (correction: 5.55 kcal/mol)<br><br>---<br>Low frequencies before projection:<br>-187.6022<br>21.3730<br>25.9468<br><br>Low frequencies after projection:<br>-187.6021<br>21.0103<br>25.8801<br>---
**Cartesian Coordinates**	C -2.6295233 1.9344688 -0.5638920<br>S -4.1961533 2.5979188 -0.4270820<br>N -1.7821933 1.8091488 0.4951380<br>N -2.1391333 1.4482388 -1.7245920<br>H -1.2364333 0.9726988 -1.7045620<br>C -2.0122033 2.4606788 1.7723980<br>H -0.8059833 1.6377588 0.2537280<br>H -2.9367433 2.0972688 2.2288080<br>N -2.6029443 -1.3065912 0.0448480<br>C -3.0267443 -1.6176312 -1.2062720<br>H -2.2374143 -1.6583212 -1.9438420<br>H 3.0339767 -0.4127722 -1.8318920<br>C 1.8741567 1.2714078 -1.1304420<br>C 2.0709867 -0.1163122 -1.4397920<br>O 0.8590267 1.7994388 -0.6706520<br>C 0.9608367 -0.9865412 -1.7304520<br>O -0.2396433 -0.7033312 -1.7979220<br>C 2.6332867 -0.8131922 0.7121580<br>C 1.7893567 -0.1824122 1.6219580<br>C 4.0721067 -0.5237422 0.6610280<br>H 2.3281357 -1.7940922 0.3675480<br>N 0.4976767 -0.5991512 1.7830980

	H	2.0455467	0.6787178	2.2189680
	O	0.0299857	-1.5600812	1.0988680
	O	-0.2280933	-0.0139012	2.6442980
	C	4.9412957	-1.5029522	0.1467580
	C	4.6174867	0.6994878	1.0915480
	C	5.9897567	0.9251778	1.0258980
	C	6.3137857	-1.2767522	0.0827780
	C	6.8433067	-0.0612122	0.5240880
	H	4.5278657	-2.4446522	-0.2059620
	H	3.9667567	1.4832978	1.4652680
	H	6.3946967	1.8761278	1.3613580
	H	6.9704057	-2.0470622	-0.3124420
	H	7.9136767	0.1188468	0.4725680
	H	-2.0935233	3.5508388	1.6709380
	H	-1.1728733	2.2101988	2.4231880
	C	-2.8688333	1.4184888	-2.9744120
	H	-3.7966733	0.8403388	-2.8889820
	H	-2.2218533	0.9524488	-3.7230620
	H	-3.1325633	2.4281188	-3.3077720
	C	-3.4444943	-1.1671512	1.1124480
	C	-4.8282243	-1.3893502	0.8790680
	N	-2.9227233	-0.8248712	2.2919080
	H	-5.5261743	-1.2786302	1.6982980
	O	3.0231367	1.9861278	-1.2942620
	O	1.3914757	-2.2625322	-1.9403020
	C	2.9678967	3.3506678	-0.8625120
	H	2.2655067	3.9279678	-1.4725820
	H	3.9805667	3.7392978	-0.9866520
	H	2.6603667	3.4242678	0.1854680
	C	0.3669957	-3.2397612	-2.1486320
	H	0.8888057	-4.1889012	-2.2867120
	H	-0.2259743	-3.0099112	-3.0400920
	H	-0.2927243	-3.3016012	-1.2773220
	H	-1.9175033	-0.6129712	2.3540580
	H	-1.5916343	-1.1904912	0.2059580
	C	-4.3567643	-1.8309112	-1.4605120
	H	-4.6887843	-2.0713212	-2.4628320
	C	-5.2644543	-1.7123802	-0.3857420
	H	-6.3256843	-1.8668302	-0.5570220
	C	-3.7230733	-0.5702912	3.4772380
	H	-4.2600343	-1.4696712	3.8022680
	H	-4.4473233	0.2361088	3.3095080
	H	-3.0437633	-0.2672712	4.2757480

Name	$TS_{Theo2S-BMB-S32}$
**Absolute Energies (Hartree)**	There are 14 positive frequencies below 100 cm^-1.<br><br>Zero-point correction: 0.530982 hartree<br>Enthalpy correction: 0.568858 hartree<br>Free Energy correction: 0.462268 hartree<br>Quasiharmonic Free Energy correction: 0.471108 hartree<br><br>SCF Energy: -1980.339123 hartree<br>SCF Energy + ZPVE: -1979.808141 hartree<br>Enthalpy: -1979.770265 hartree<br>Free Energy: -1979.876855 hartree<br><br>Free Energy with quasiharmonic correction: -1979.868015 hartree (correction: 5.55 kcal/mol)<br><br>---<br>Low frequencies before projection:<br>-187.5811<br>21.4327<br>25.9917<br><br>Low frequencies after projection:<br>-187.5809<br>21.0929<br>25.922<br>---
**Cartesian Coordinates**	C -2.6295608 1.9346234 -0.5634808<br>S -4.1961208 2.5981844 -0.4264708<br>N -1.7822008 1.8089734 0.4954992<br>N -2.1392318 1.4486334 -1.7243108<br>H -1.2365718 0.9730234 -1.7043908<br>C -2.0121008 2.4602534 1.7728992<br>H -0.8060318 1.6375134 0.2540092<br>H -2.9365908 2.0967534 2.2293392<br>N -2.6029418 -1.3065966 0.0445492<br>C -3.0267718 -1.6172866 -1.2066408<br>H -2.2374618 -1.6577766 -1.9442408<br>H 3.0339282 -0.4123376 -1.8320108<br>C 1.8741882 1.2716824 -1.1300708<br>C 2.0709582 -0.1159576 -1.4397908<br>O 0.8591392 1.7996124 -0.6699808<br>C 0.9607882 -0.9861076 -1.7306208<br>O -0.2396918 -0.7028566 -1.7979608<br>C 2.6333082 -0.8133976 0.7119992<br>C 1.7893882 -0.1828476 1.6219592<br>C 4.0721282 -0.5239186 0.6608992<br>H 2.3281482 -1.7942176 0.3671492<br>N 0.4976982 -0.5996076 1.7829892

	H	2.0455782	0.6781624	2.2191492
	O	0.0300082	-1.5603766	1.0985492
	O	-0.2280618	-0.0145366	2.6443092
	C	4.9413182	-1.5030186	0.1464192
	C	4.6174882	0.6992314	1.0916592
	C	5.9897482	0.9249614	1.0260192
	C	6.3137982	-1.2767686	0.0824492
	C	6.8433082	-0.0613086	0.5239892
	H	4.5278972	-2.4446486	-0.2064908
	H	3.9667582	1.4829514	1.4655492
	H	6.3946892	1.8758514	1.3616692
	H	6.9704272	-2.0469886	-0.3129508
	H	7.9136782	0.1187704	0.4724892
	H	-2.0934308	3.5504334	1.6716592
	H	-1.1727008	2.2096534	2.4235592
	C	-2.8689718	1.4192234	-2.9741108
	H	-3.7968218	0.8410544	-2.8888208
	H	-2.2220118	0.9533834	-3.7229108
	H	-3.1326908	2.4289534	-3.3071908
	C	-3.4444718	-1.1674456	1.1122092
	C	-4.8282018	-1.3895756	0.8787992
	N	-2.9226818	-0.8254666	2.2917592
	H	-5.5261318	-1.2790956	1.6980792
	O	3.0231392	1.9864324	-1.2939008
	O	1.3913872	-2.2620476	-1.9408108
	C	2.9679792	3.3508724	-0.8618108
	H	2.2654492	3.9283124	-1.4715908
	H	3.9806292	3.7395414	-0.9860808
	H	2.6606792	3.4242124	0.1862492
	C	0.3668872	-3.2392076	-2.1493208
	H	0.8886672	-4.1883276	-2.2876308
	H	-0.2261028	-3.0091466	-3.0407108
	H	-0.2928028	-3.3012366	-1.2780008
	H	-1.9174618	-0.6135866	2.3539392
	H	-1.5916318	-1.1905366	0.2056692
	C	-4.3568018	-1.8305056	-1.4609108
	H	-4.6888428	-2.0706256	-2.4632908
	C	-5.2644618	-1.7122656	-0.3860908
	H	-6.3257018	-1.8666656	-0.5573908
	C	-3.7230118	-0.5711856	3.4771592
	H	-4.4472718	0.2352344	3.3096392
	H	-3.0436918	-0.2683566	4.2757392
	H	-4.2599518	-1.4706556	3.8019792

Name	$TS_{Theo2O\text{-}BMA\text{-}R1}$
**Absolute Energies (Hartree)**	There are 15 positive frequencies below 100 cm^-1.<br><br>Zero-point correction: 0.532980 hartree<br>Enthalpy correction: 0.570806 hartree<br>Free Energy correction: 0.462911 hartree<br>Quasiharmonic Free Energy correction: 0.472621 hartree<br><br>SCF Energy: -1657.365702 hartree<br>SCF Energy + ZPVE: -1656.832722 hartree<br>Enthalpy: -1656.794896 hartree<br>Free Energy: -1656.902791 hartree<br><br>Free Energy with quasiharmonic correction: -1656.893082 hartree (correction: 6.09 kcal/mol)<br><br>---<br>Low frequencies before projection:<br>-367.8014<br>14.4176<br>24.9302<br><br>Low frequencies after projection:<br>-367.8013<br>13.0826<br>24.1971<br>---
**Cartesian Coordinates**	N -2.3506450 2.8182122 -0.0196386<br>C -3.4531450 2.1014932 -0.2776186<br>C -5.6426650 0.4295632 -0.8201186<br>C -4.7714250 2.5333032 0.0211114<br>N -3.3110650 0.8641332 -0.8379186<br>C -4.3558650 0.0418132 -1.0989986<br>C -5.8394550 1.7041132 -0.2508986<br>N 6.0446750 0.1253112 0.8751314<br>C 5.9987550 0.4398012 -0.4611486<br>O 6.9576050 0.9099012 -1.0842286<br>N 4.7702450 0.1944312 -1.0477786<br>N 2.5988050 -0.7862078 1.4605814<br>O 3.3419350 0.0531322 2.0583814<br>O 3.0907340 -1.6319078 0.6476714<br>C 1.2589350 -0.7588078 1.6890014<br>C 0.3818340 -1.6275578 0.9846614<br>C 0.4052450 -1.1936078 -0.9833386<br>C 0.4705550 0.2558022 -0.9859386<br>O -0.4877950 1.0390222 -0.9576686<br>O 1.7365650 0.6770522 -0.8927786<br>C 1.9728050 2.0677922 -0.6242986<br>C -0.7731460 -1.8502978 -1.5361686

	O	-1.8560560	-1.3369978	-1.8078886
	O	-0.5629460	-3.1815778	-1.6664586
	C	-1.6926360	-3.9501078	-2.1084686
	C	4.6556650	0.1964812	-2.4918586
	C	7.1631050	0.5636112	1.6818214
	C	-2.3692250	4.1413322	0.5812314
	H	1.3541340	-1.6762078	-1.1878586
	H	5.1625450	1.0757912	-2.8972286
	H	5.1010050	-0.6936288	-2.9643886
	H	3.5952250	0.2390712	-2.7578786
	H	4.1433850	-0.4346288	-0.5466886
	H	5.1530550	-0.0319088	1.3483014
	H	8.0983050	0.3746412	1.1483014
	H	7.1692650	-0.0011488	2.6196914
	H	7.1274450	1.6380712	1.9246114
	H	3.0539750	2.1473422	-0.5199886
	H	0.9316050	0.0484522	2.3261814
	H	-2.9092040	4.8627132	-0.0440986
	H	-1.3361250	4.4776222	0.6826214
	H	1.6126950	2.6874622	-1.4512986
	H	1.4760350	2.3645722	0.3044014
	H	-1.4555250	2.4102222	-0.2899286
	H	-2.3621350	0.5046422	-1.0476986
	H	-4.0752850	-0.9142868	-1.5189086
	H	-6.4712450	-0.2343168	-1.0328586
	H	-6.8451150	2.0420732	-0.0181386
	H	-4.9218250	3.5107232	0.4607614
	H	-2.8264550	4.1230732	1.5777714
	H	-2.0057160	-3.6416378	-3.1106786
	H	-1.3524160	-4.9867878	-2.1235186
	H	-2.5330560	-3.8360478	-1.4173286
	H	0.7839240	-2.6159978	0.7777914
	C	-1.0525060	-1.6268478	1.3808514
	C	-3.7765660	-1.6892768	2.0968214
	C	-1.7233550	-0.4511078	1.7570314
	C	-1.7762260	-2.8302778	1.3572014
	C	-3.1249260	-2.8628968	1.7107714
	C	-3.0685550	-0.4854968	2.1210714
	H	-1.1944550	0.4963822	1.7489514
	H	-1.2712360	-3.7447178	1.0584314
	H	-3.6643960	-3.8063168	1.6918514
	H	-3.5710750	0.4329232	2.4129014
	H	-4.8265960	-1.7114768	2.3757914

Name	TS$_{Theo2O-BMA-R2}$
**Absolute Energies (Hartree)**	There are 15 positive frequencies below 100 cm^-1.<br><br>Zero-point correction: 0.533257 hartree<br>Enthalpy correction: 0.570724 hartree<br>Free Energy correction: 0.465071 hartree<br>Quasiharmonic Free Energy correction: 0.473366 hartree<br><br>SCF Energy: -1657.376012 hartree<br>SCF Energy + ZPVE: -1656.842755 hartree<br>Enthalpy: -1656.805288 hartree<br>Free Energy: -1656.910941 hartree<br><br>Free Energy with quasiharmonic correction: -1656.902645 hartree (correction: 5.21 kcal/mol)<br><br>--<br>Low frequencies before projection:<br>-222.1845<br>19.8786<br>20.8489<br><br>Low frequencies after projection:<br>-222.1845<br>19.42<br>20.185<br>--
**Cartesian Coordinates**	N 0.0265631 2.8786587 0.2004728<br>C 1.3420531 2.6669497 0.0811428<br>C 4.0709441 2.0018807 -0.0872972<br>C 2.2301031 3.4821197 -0.6696772<br>N 1.8818241 1.5868497 0.7151128<br>C 3.1870641 1.2402897 0.6361928<br>C 3.5666531 3.1442497 -0.7432172<br>N 3.8729251 -2.0480493 0.4593528<br>C 4.4574851 -1.4255293 -0.6343872<br>O 5.6660751 -1.1778993 -0.7005372<br>N 3.5562951 -1.0755203 -1.6085172<br>N 0.3882551 -2.0831313 -0.3251972<br>O 1.1160351 -3.1122603 -0.2395272<br>O 0.9151941 -0.9198603 -0.3115972<br>C -0.9651249 -2.2203113 -0.4496672<br>C -1.7940449 -1.0914113 -0.4853572<br>C -2.1756759 -0.4516113 1.5776128<br>C -2.9370759 0.7641677 1.3543528<br>O -2.4930159 1.8612887 1.0146828<br>O -4.2682759 0.5420977 1.4746028<br>C -5.1189459 1.6435777 1.1297128<br>C -0.8366959 -0.3735813 2.1060228

	O	-0.1363859	0.6442187	2.2252428
	O	-0.3797249	-1.5951813	2.4566228
	C	1.0003651	-1.6745603	2.8457328
	C	3.9642741	-0.2902993	-2.7518172
	C	4.7237151	-2.7418493	1.4103828
	C	-0.6698069	3.9728087	-0.4525972
	H	-2.7456149	-1.3251523	1.8674328
	H	4.9681441	-0.5935993	-3.0574572
	H	3.2706141	-0.4706503	-3.5795472
	H	3.9846341	0.7905107	-2.5444172
	H	2.5728051	-1.0671403	-1.3420972
	H	2.9563151	-2.4633203	0.2867228
	H	5.1364551	-3.6819493	1.0147428
	H	4.1392451	-2.9695393	2.3077128
	H	5.5618851	-2.0977793	1.6863628
	H	-6.1379559	1.2598767	1.2030328
	H	-1.3102549	-3.2427213	-0.4228872
	H	-0.3180669	4.9486187	-0.0944772
	H	-1.7276769	3.8680387	-0.2132072
	H	-4.9778569	2.4775077	1.8249228
	H	-4.9203059	1.9877377	0.1112328
	H	-0.5499569	2.2446287	0.7634728
	H	1.2347041	0.9966097	1.2676128
	H	3.4551641	0.3263497	1.1515828
	H	5.1044341	1.6915707	-0.1728072
	H	4.2377831	3.7688807	-1.3259072
	H	1.8459931	4.3567897	-1.1786572
	H	-0.5480369	3.9331687	-1.5423572
	H	1.2046451	-1.0174703	3.6963528
	H	1.1640051	-2.7168203	3.1226928
	H	1.6454551	-1.4061303	2.0044228
	H	-1.2930459	-0.1543313	-0.7070572
	C	-3.1901049	-1.1879123	-0.9597372
	C	-5.8487849	-1.2724633	-1.8742272
	C	-3.9996249	-2.3074023	-0.7041172
	C	-3.7386859	-0.1103323	-1.6738772
	C	-5.0550159	-0.1534223	-2.1320572
	C	-5.3155449	-2.3482023	-1.1575272
	H	-3.6040149	-3.1407723	-0.1304472
	H	-3.1261659	0.7674977	-1.8620072
	H	-5.4603859	0.6878277	-2.6881672
	H	-5.9299449	-3.2194833	-0.9469472
	H	-6.8762849	-1.3071233	-2.2261572

Name	$TS_{Theo2O\text{-}BMA\text{-}R22}$
**Absolute Energies (Hartree)**	There are 15 positive frequencies below 100 cm^-1.<br><br>Zero-point correction: 0.533262 hartree<br>Enthalpy correction: 0.570727 hartree<br>Free Energy correction: 0.465090 hartree<br>Quasiharmonic Free Energy correction: 0.473375 hartree<br><br>SCF Energy: -1657.376012 hartree<br>SCF Energy + ZPVE: -1656.842750 hartree<br>Enthalpy: -1656.805285 hartree<br>Free Energy: -1656.910922 hartree<br><br>Free Energy with quasiharmonic correction: -1656.902636 hartree (correction: 5.20 kcal/mol)<br><br>---<br>Low frequencies before projection:<br>-222.2992<br>19.8951<br>20.8677<br><br>Low frequencies after projection:<br>-222.2992<br>19.4853<br>20.2032<br>---
**Cartesian Coordinates**	N -3.5559100 -1.0756402 1.6086100<br>C -4.4576100 -1.4253802 0.6348600<br>O -5.6661700 -1.1778602 0.7018400<br>N -3.8736700 -2.0475202 -0.4594300<br>N -1.8813700 1.5871598 -0.7149000<br>H -2.5725700 -1.0670102 1.3416300<br>H -2.9570000 -2.4629102 -0.2874300<br>H -1.2345200 0.9968298 -1.2675600<br>O -1.1165900 -3.1126502 0.2378200<br>N -0.3887700 -2.0835902 0.3238000<br>O -0.9156700 -0.9202602 0.3101900<br>C 0.9645500 -2.2207792 0.4486200<br>C 0.8368300 -0.3732692 -2.1065100<br>C 2.1756700 -0.4517792 -1.5777200<br>C 2.9374600 0.7637308 -1.3542600<br>O 2.4937100 1.8610408 -1.0148200<br>O 4.2686000 0.5411308 -1.4740800<br>C 5.1195800 1.6423308 -1.1290600<br>H 6.1384700 1.2582908 -1.2021900<br>H 4.9208700 1.9865608 -0.1106200<br>H 4.9789000 2.4763108 -1.8243100<br>O 0.1367700 0.6447008 -2.2257500

	O	0.3797000	-1.5946892	-2.4575300
	C	-1.0002900	-1.6737202	-2.8470400
	H	-1.1640500	-2.7158602	-3.1243800
	H	-1.2042400	-1.0163102	-3.6975000
	H	-1.6455600	-1.4054602	-2.0058000
	H	0.5504900	2.2447308	-0.7641000
	C	-1.3412800	2.6672798	-0.0812300
	N	-0.0258110	2.8788608	-0.2009800
	C	-4.7250800	-2.7411402	-1.4100600
	H	-5.1381400	-3.6809602	-1.0141000
	H	-4.1409500	-2.9692602	-2.3075000
	H	-5.5630400	-2.0967502	-1.6859500
	C	1.7934400	-1.0918292	0.4847900
	H	1.3097100	-3.2431792	0.4218100
	C	-3.9634300	-0.2906102	2.7522300
	H	-4.9662500	-0.5954902	3.0597400
	H	-3.9860300	0.7900798	2.5444200
	H	-3.2680400	-0.4694202	3.5788300
	C	-3.1866200	1.2407198	-0.6354800
	H	-3.4549700	0.3267298	-1.1506600
	H	2.7453900	-1.3254592	-1.8675500
	H	1.2922800	-0.1548392	0.7065500
	C	3.1892900	-1.1883392	0.9597800
	C	5.8475600	-1.2728792	1.8754800
	C	3.9989400	-2.3078092	0.7044900
	C	3.7375300	-0.1107792	1.6742200
	C	5.0536500	-0.1538692	2.1330000
	C	5.3146600	-2.3485992	1.1585000
	H	3.6036100	-3.1411692	0.1306300
	H	3.1249200	0.7670408	1.8621000
	H	5.4587500	0.6873708	2.6893300
	H	5.9291700	-3.2198592	0.9481800
	H	6.8749000	-1.3075382	2.2278800
	C	0.6708090	3.9731708	0.4515800
	H	0.3186690	4.9489008	0.0936100
	H	0.5497290	3.9335908	1.5414100
	H	1.7285390	3.8685508	0.2115300
	C	-2.2290110	3.4826198	0.6698100
	H	-1.8446310	4.3572898	1.1786000
	C	-3.5655610	3.1448998	0.7438100
	H	-4.2364410	3.7696698	1.3266400
	C	-4.0701700	2.0024998	0.0881800
	H	-5.1037000	1.6923798	0.1740000

Name	$TS_{Theo2O\text{-}BMA\text{-}R3}$
Absolute Energies (Hartree)	There are 15 positive frequencies below 100 cm^-1.<br><br>Zero-point correction: 0.533056 hartree<br>Enthalpy correction: 0.570781 hartree<br>Free Energy correction: 0.463450 hartree<br>Quasiharmonic Free Energy correction: 0.472908 hartree<br><br>SCF Energy: -1657.376816 hartree<br>SCF Energy + ZPVE: -1656.843760 hartree<br>Enthalpy: -1656.806035 hartree<br>Free Energy: -1656.913366 hartree<br><br>Free Energy with quasiharmonic correction: -1656.903909 hartree (correction: 5.93 kcal/mol)<br><br>---<br>Low frequencies before projection:<br>-219.0514<br>14.0339<br>17.4980<br><br>Low frequencies after projection:<br>-219.0514<br>13.9129<br>16.7344<br>---
Cartesian Coordinates	N -2.3348622 0.9485345 1.5414505<br>C -1.9116122 1.9824245 0.8088705<br>C -0.8387832 4.0622645 -0.7443695<br>C -2.7741022 2.8956345 0.1440405<br>N -0.5667122 2.1876145 0.6812205<br>C -0.0337822 3.1765845 -0.0759395<br>C -2.2371332 3.9069045 -0.6196195<br>N -3.9846322 -0.2038255 -1.4028295<br>C -4.4409722 -1.4169065 -0.9263195<br>O -5.5958822 -1.8153065 -1.0991695<br>N -3.5119222 -2.1008255 -0.1655195<br>N -0.2664422 -0.5067355 -1.0704495<br>O -1.1034922 0.3104045 -1.5583295<br>O -0.6430322 -1.5886655 -0.5254295<br>C 1.0648678 -0.2052455 -1.1707695<br>C 2.0454978 -1.0756455 -0.6781495<br>C 2.3499378 -0.7290455 1.4645505<br>C 1.0159478 -0.9561755 1.9594105<br>O 0.1420978 -0.0904855 2.1101805<br>O 0.7762178 -2.2553355 2.2301705<br>C -0.5843522 -2.5793055 2.5550305<br>C 2.8688578 0.6238645 1.4147305

	O	2.2221578	1.6522945	1.2140605	
	O	4.2201778	0.6410455	1.5275905	
	C	4.8439678	1.9206755	1.3622005	
	C	-3.7940622	-3.4744755	0.2093205	
	C	-4.7665922	0.5080835	-2.3951595	
	C	-3.7338922	0.6786645	1.8351705	
	H	3.0689778	-1.5164855	1.6536905	
	H	-4.7847422	-3.5360465	0.6666805	
	H	-3.7750422	-4.1682355	-0.6447895	
	H	-3.0464322	-3.8026755	0.9384705	
	H	-2.5297222	-1.8737055	-0.3284795	
	H	-2.9731422	-0.0668255	-1.4380795	
	H	-4.3529422	1.5148235	-2.5106695	
	H	-5.8049722	0.5850835	-2.0625895	
	H	-4.7653222	0.0164535	-3.3799395	
	H	-0.6098422	-3.6659155	2.6531205	
	H	1.2582078	0.7450245	-1.6414295	
	H	-4.1551622	1.4480045	2.4947705	
	H	-3.7898022	-0.2882755	2.3354005	
	H	-0.8826022	-2.1120655	3.4994605	
	H	-1.2521122	-2.2518355	1.7540805	
	H	-1.6200122	0.3131845	1.8965205	
	H	0.0888978	1.5362445	1.1472805	
	H	1.0478578	3.1941445	-0.0877095	
	H	-0.4049232	4.8516245	-1.3455995	
	H	-2.9037732	4.5911645	-1.1366695	
	H	-3.8446122	2.7650345	0.2285305	
	H	-4.3194722	0.6178235	0.9146705	
	H	4.5353378	2.6105755	2.1542705	
	H	5.9173578	1.7331855	1.4226905	
	H	4.5947878	2.3580355	0.3912605	
	H	1.7013378	-2.0792755	-0.4510795	
	C	3.4488378	-0.9738855	-1.1321795	
	C	6.1360778	-0.8681945	-1.9645595	
	C	4.0056378	0.2242455	-1.6117095	
	C	4.2668278	-2.1142345	-1.0718895	
	C	5.5960478	-2.0643945	-1.4864095	
	C	5.3352778	0.2752155	-2.0242995	
	H	3.4032978	1.1264645	-1.6467795	
	H	3.8510878	-3.0459045	-0.6955095	
	H	6.2103778	-2.9593845	-1.4366195	
	H	5.7482278	1.2108755	-2.3920795	
	H	7.1727078	-0.8268845	-2.2877695	

Name	$TS_{Theo20\text{-}BMA\text{-}R3}$*conf*
**Absolute Energies (Hartree)**	There are 15 positive frequencies below 100 cm^-1.<br><br>Zero-point correction: 0.533055 hartree<br>Enthalpy correction: 0.570781 hartree<br>Free Energy correction: 0.463443 hartree<br>Quasiharmonic Free Energy correction: 0.472907 hartree<br><br>SCF Energy: -1657.376816 hartree<br>SCF Energy + ZPVE: -1656.843761 hartree<br>Enthalpy: -1656.806035 hartree<br>Free Energy: -1656.913373 hartree<br><br>Free Energy with quasiharmonic correction: -1656.903910 hartree (correction: 5.94 kcal/mol)<br><br>---<br>Low frequencies before projection:<br>-219.0504<br>14.0155<br>17.5044<br><br>Low frequencies after projection:<br>-219.0503<br>13.8975<br>16.7379<br>---
**Cartesian Coordinates**	N -2.3346149 0.9487389 1.5414837<br>C -1.9113949 1.9826189 0.8088637<br>C -0.8385949 4.0624489 -0.7443863<br>C -2.7738949 2.8957489 0.1439537<br>N -0.5664949 2.1878989 0.6813237<br>C -0.0335849 3.1768689 -0.0758563<br>C -2.2369549 3.9070189 -0.6197163<br>N -3.9847749 -0.2038711 -1.4028663<br>C -4.4410449 -1.4169311 -0.9261863<br>O -5.5959649 -1.8153411 -1.0989363<br>N -3.5119449 -2.1007311 -0.1653463<br>N -0.2665449 -0.5066711 -1.0703363<br>O -1.1036049 0.3104989 -1.5581263<br>O -0.6431349 -1.5886011 -0.5252963<br>C 1.0647651 -0.2052411 -1.1707363<br>C 2.0453751 -1.0756411 -0.6780663<br>C 2.3499051 -0.7289611 1.4645737<br>C 1.0159551 -0.9561811 1.9595337<br>O 0.1421451 -0.0905111 2.1107137<br>O 0.7762451 -2.2554211 2.2299337<br>C -0.5842749 -2.5794711 2.5549037<br>C 2.8687451 0.6239789 1.4147637

	O	2.2219551	1.6523689	1.2142437
	O	4.2200751	0.6412389	1.5274237
	C	4.8437751	1.9208989	1.3619437
	C	-3.7939749	-3.4743411	0.2096837
	C	-4.7667549	0.5077089	-2.3954263
	C	-3.7336349	0.6787089	1.8350937
	H	3.0689951	-1.5163711	1.6536037
	H	-4.7848449	-3.5359711	0.6666137
	H	-3.7744649	-4.1682911	-0.6442663
	H	-3.0466249	-3.8022611	0.9392237
	H	-2.5297549	-1.8735611	-0.3283563
	H	-2.9732749	-0.0669311	-1.4382663
	H	-4.7660749	0.0153989	-3.3798563
	H	-4.3526449	1.5141789	-2.5117463
	H	-5.8049949	0.5854789	-2.0625563
	H	-0.6097549	-3.6661211	2.6527037
	H	1.2581151	0.7450089	-1.6414263
	H	-4.1549649	1.4478689	2.4948737
	H	-3.7895149	-0.2883511	2.3350537
	H	-0.8823949	-2.1124811	3.4994837
	H	-1.2521449	-2.2518111	1.7541237
	H	-1.6197149	0.3134389	1.8965437
	H	0.0891751	1.5366689	1.1474537
	H	1.0480551	3.1945089	-0.0875563
	H	-0.4047449	4.8518089	-1.3456363
	H	-2.9035949	4.5912189	-1.1368363
	H	-3.8444049	2.7650889	0.2283837
	H	-4.3191849	0.6180789	0.9145637
	H	4.5354751	2.6106889	2.1542337
	H	5.9171951	1.7334189	1.4219437
	H	4.5941751	2.3583889	0.3911637
	H	1.7011651	-2.0792411	-0.4509263
	C	3.4487151	-0.9739911	-1.1321363
	C	6.1359451	-0.8685211	-1.9645663
	C	4.0055451	0.2240489	-1.6118463
	C	4.2666651	-2.1143711	-1.0717063
	C	5.5958751	-2.0646311	-1.4862463
	C	5.3351951	0.2749089	-2.0244463
	H	3.4032451	1.1262789	-1.6470563
	H	3.8508951	-3.0459711	-0.6951863
	H	6.2101751	-2.9596411	-1.4363463
	H	5.7481651	1.2104989	-2.3923663
	H	7.1725751	-0.8272911	-2.2877863

Name	$TS_{Theo2O-BMA-R33}$
Absolute Energies (Hartree)	There are 15 positive frequencies below 100 cm^-1.<br><br>Zero-point correction: 0.532947 hartree<br>Enthalpy correction: 0.570669 hartree<br>Free Energy correction: 0.463405 hartree<br>Quasiharmonic Free Energy correction: 0.473040 hartree<br><br>SCF Energy: -1657.373386 hartree<br>SCF Energy + ZPVE: -1656.840439 hartree<br>Enthalpy: -1656.802717 hartree<br>Free Energy: -1656.909981 hartree<br><br>Free Energy with quasiharmonic correction: -1656.900346 hartree (correction: 6.05 kcal/mol)<br><br>---<br>Low frequencies before projection:<br>-226.5666<br>17.6007<br>22.1297<br><br>Low frequencies after projection:<br>-226.5665<br>17.1891<br>21.7741<br>---
**Cartesian Coordinates**	N -4.0857999 -2.0567241 -0.2591723<br>C -5.0074309 -1.0634441 -0.5097423<br>O -6.2248809 -1.2354551 -0.3970023<br>N -4.4518209 0.1545159 -0.8450023<br>N 1.2280671 2.9215699 0.3034677<br>H -3.1364999 -1.9346831 -0.6133323<br>H -3.4745309 0.1553069 -1.1377623<br>H 1.7389771 2.1468199 0.7308777<br>O -1.5935419 0.2599579 -1.5043423<br>N -0.7959909 -0.6750221 -1.1914623<br>O -1.2166899 -1.8435221 -0.9429723<br>C 0.5434391 -0.3922511 -1.1572223<br>C 2.4929091 -0.4718991 1.5871177<br>C 1.7097901 -1.6413201 1.2618977<br>C 0.3214101 -1.6940211 1.6853577<br>O -0.4158609 -0.7252211 1.8758977<br>O -0.1155889 -2.9612011 1.8261677<br>C -1.5178789 -3.0984221 2.1119877<br>H -1.7234289 -4.1686221 2.0528377<br>H -2.1139799 -2.5453621 1.3840577<br>H -1.7436899 -2.7299121 3.1182477<br>O 2.0735681 0.6871999 1.6922077

	O	3.8077991	-0.7585881	1.7068577
	C	4.6826981	0.3595819	1.9085677
	H	5.6889691	-0.0607371	1.9416977
	H	4.4531181	0.8698519	2.8492077
	H	4.6051381	1.0704919	1.0812677
	H	-0.1178219	0.9883189	1.2717977
	C	-0.1090929	2.9159289	0.4484977
	C	-0.9626339	3.9787079	0.0690377
	N	-0.6900029	1.8006089	0.9745577
	C	-2.0297619	1.6453579	1.0912277
	H	-2.3445519	0.6826369	1.4700977
	C	-2.8873629	2.6586069	0.7373277
	H	-3.9554429	2.5182059	0.8340877
	C	-2.3293939	3.8427869	0.2232377
	H	-2.9785939	4.6637669	-0.0674623
	C	-5.3058319	1.1754649	-1.4206023
	H	-4.7238329	2.0948459	-1.5347823
	H	-6.1553619	1.3712649	-0.7600723
	H	-5.7085719	0.8949549	-2.4057923
	C	1.4929301	-1.3847701	-0.8686323
	H	0.7719781	0.6397299	-1.3686623
	H	-0.5345649	4.8851589	-0.3390923
	C	-4.5510489	-3.4200041	-0.0895223
	H	-5.2905089	-3.4702841	0.7150377
	H	-3.6971389	-4.0495831	0.1761577
	H	-5.0182489	-3.8287541	-0.9977723
	C	1.9766561	4.0751299	-0.1628523
	H	1.6866461	4.3503499	-1.1834723
	H	3.0354161	3.8089809	-0.1672223
	H	1.8405651	4.9472099	0.4902677
	H	2.2462601	-2.5823991	1.2920077
	H	1.1258401	-2.4035501	-0.9405523
	C	2.9125191	-1.1945391	-1.2479023
	C	5.6338691	-0.9200071	-1.9230323
	C	3.4792191	0.0765609	-1.4470523
	C	3.7373301	-2.3218481	-1.3915423
	C	5.0827101	-2.1885581	-1.7276923
	C	4.8253991	0.2106319	-1.7828623
	H	2.8703881	0.9680809	-1.3344423
	H	3.3147111	-3.3110091	-1.2328323
	H	5.7014111	-3.0752071	-1.8366823
	H	5.2446581	1.2017029	-1.9373023
	H	6.6829891	-0.8133671	-2.1852123

Name	$TS_{Theo2O\text{-}BMA\text{-}S11}$
**Absolute Energies (Hartree)**	There are 15 positive frequencies below 100 cm^-1.<br><br>Zero-point correction: 0.533262 hartree<br>Enthalpy correction: 0.570727 hartree<br>Free Energy correction: 0.465090 hartree<br>Quasiharmonic Free Energy correction: 0.473375 hartree<br><br>SCF Energy: -1657.376012 hartree<br>SCF Energy + ZPVE: -1656.842750 hartree<br>Enthalpy: -1656.805285 hartree<br>Free Energy: -1656.910922 hartree<br><br>Free Energy with quasiharmonic correction: -1656.902636 hartree (correction: 5.20 kcal/mol)<br><br>---<br>Low frequencies before projection:<br>-222.2992<br>19.8951<br>20.8677<br><br>Low frequencies after projection:<br>-222.2992<br>19.4853<br>20.2032<br>---
**Cartesian Coordinates**	N -3.5559100 -1.0756402 -1.6086100<br>C -4.4576100 -1.4253802 -0.6348600<br>O -5.6661700 -1.1778602 -0.7018400<br>N -3.8736700 -2.0475202 0.4594300<br>N -1.8813700 1.5871598 0.7149000<br>H -2.5725700 -1.0670102 -1.3416300<br>H -2.9570000 -2.4629102 0.2874300<br>H -1.2345200 0.9968298 1.2675600<br>O -1.1165900 -3.1126502 -0.2378200<br>N -0.3887700 -2.0835902 -0.3238000<br>O -0.9156700 -0.9202602 -0.3101900<br>C 0.9645500 -2.2207792 -0.4486200<br>C 0.8368300 -0.3732692 2.1065100<br>C 2.1756700 -0.4517792 1.5777200<br>C 2.9374600 0.7637308 1.3542600<br>O 2.4937100 1.8610408 1.0148200<br>O 4.2686000 0.5411308 1.4740800<br>C 5.1195800 1.6423308 1.1290600<br>H 6.1384700 1.2582908 1.2021900<br>H 4.9208700 1.9865608 0.1106200<br>H 4.9789000 2.4763108 1.8243100<br>O 0.1367700 0.6447008 2.2257500

	O	0.3797000	-1.5946892	2.4575300
	C	-1.0002900	-1.6737202	2.8470400
	H	-1.1640500	-2.7158602	3.1243800
	H	-1.2042400	-1.0163102	3.6975000
	H	-1.6455600	-1.4054602	2.0058000
	H	0.5504900	2.2447308	0.7641000
	C	-1.3412800	2.6672798	0.0812300
	N	-0.0258110	2.8788608	0.2009800
	C	-4.7250800	-2.7411402	1.4100600
	H	-5.1381400	-3.6809602	1.0141000
	H	-4.1409500	-2.9692602	2.3075000
	H	-5.5630400	-2.0967502	1.6859500
	C	1.7934400	-1.0918292	-0.4847900
	H	1.3097100	-3.2431792	-0.4218100
	C	-3.9634300	-0.2906102	-2.7522300
	H	-4.9662500	-0.5954902	-3.0597400
	H	-3.9860300	0.7900798	-2.5444200
	H	-3.2680400	-0.4694202	-3.5788300
	C	-3.1866200	1.2407198	0.6354800
	H	-3.4549700	0.3267298	1.1506600
	H	2.7453900	-1.3254592	1.8675500
	H	1.2922800	-0.1548392	-0.7065500
	C	3.1892900	-1.1883392	-0.9597800
	C	5.8475600	-1.2728792	-1.8754800
	C	3.9989400	-2.3078092	-0.7044900
	C	3.7375300	-0.1107792	-1.6742200
	C	5.0536500	-0.1538692	-2.1330000
	C	5.3146600	-2.3485992	-1.1585000
	H	3.6036100	-3.1411692	-0.1306300
	H	3.1249200	0.7670408	-1.8621000
	H	5.4587500	0.6873708	-2.6893300
	H	5.9291700	-3.2198592	-0.9481800
	H	6.8749000	-1.3075382	-2.2278800
	C	0.6708090	3.9731708	-0.4515800
	H	0.3186690	4.9489008	-0.0936100
	H	0.5497290	3.9335908	-1.5414100
	H	1.7285390	3.8685508	-0.2115300
	C	-2.2290110	3.4826198	-0.6698100
	H	-1.8446310	4.3572898	-1.1786000
	C	-3.5655610	3.1448998	-0.7438100
	H	-4.2364410	3.7696698	-1.3266400
	C	-4.0701700	2.0024998	-0.0881800
	H	-5.1037000	1.6923798	-0.1740000

Name	$TS_{Theo2O-BMA-S13}$
**Absolute Energies (Hartree)**	There are 15 positive frequencies below 100 cm^-1.<br><br>Zero-point correction: 0.533257 hartree<br>Enthalpy correction: 0.570724 hartree<br>Free Energy correction: 0.465071 hartree<br>Quasiharmonic Free Energy correction: 0.473366 hartree<br><br>SCF Energy: -1657.376012 hartree<br>SCF Energy + ZPVE: -1656.842755 hartree<br>Enthalpy: -1656.805288 hartree<br>Free Energy: -1656.910941 hartree<br><br>Free Energy with quasiharmonic correction: -1656.902645 hartree (correction: 5.21 kcal/mol)<br><br>---<br>Low frequencies before projection:<br>-222.1845<br>19.8786<br>20.8489<br><br>Low frequencies after projection:<br>-222.1845<br>19.42<br>20.185<br>---
**Cartesian Coordinates**	N 0.0265631 2.8786587 -0.2004728<br>C 1.3420531 2.6669497 -0.0811428<br>C 4.0709441 2.0018807 0.0872972<br>C 2.2301031 3.4821197 0.6696772<br>N 1.8818241 1.5868497 -0.7151128<br>C 3.1870641 1.2402897 -0.6361928<br>C 3.5666531 3.1442497 0.7432172<br>N 3.8729251 -2.0480493 -0.4593528<br>C 4.4574851 -1.4255293 0.6343872<br>O 5.6660751 -1.1778993 0.7005372<br>N 3.5562951 -1.0755203 1.6085172<br>N 0.3882551 -2.0831313 0.3251972<br>O 1.1160351 -3.1122603 0.2395272<br>O 0.9151941 -0.9198603 0.3115972<br>C -0.9651249 -2.2203113 0.4496672<br>C -1.7940449 -1.0914113 0.4853572<br>C -2.1756759 -0.4516113 -1.5776128<br>C -2.9370759 0.7641677 -1.3543528<br>O -2.4930159 1.8612887 -1.0146828<br>O -4.2682759 0.5420977 -1.4746028<br>C -5.1189459 1.6435777 -1.1297128<br>C -0.8366959 -0.3735813 -2.1060228

	O	-0.1363859	0.6442187	-2.2252428
	O	-0.3797249	-1.5951813	-2.4566228
	C	1.0003651	-1.6745603	-2.8457328
	C	3.9642741	-0.2902993	2.7518172
	C	4.7237151	-2.7418493	-1.4103828
	C	-0.6698069	3.9728087	0.4525972
	H	-2.7456149	-1.3251523	-1.8674328
	H	4.9681441	-0.5935993	3.0574572
	H	3.2706141	-0.4706503	3.5795472
	H	3.9846341	0.7905107	2.5444172
	H	2.5728051	-1.0671403	1.3420972
	H	2.9563151	-2.4633203	-0.2867228
	H	5.1364551	-3.6819493	-1.0147428
	H	4.1392451	-2.9695393	-2.3077128
	H	5.5618851	-2.0977793	-1.6863628
	H	-6.1379559	1.2598767	-1.2030328
	H	-1.3102549	-3.2427213	0.4228872
	H	-0.3180669	4.9486187	0.0944772
	H	-1.7276769	3.8680387	0.2132072
	H	-4.9778569	2.4775077	-1.8249228
	H	-4.9203059	1.9877377	-0.1112328
	H	-0.5499569	2.2446287	-0.7634728
	H	1.2347041	0.9966097	-1.2676128
	H	3.4551641	0.3263497	-1.1515828
	H	5.1044341	1.6915707	0.1728072
	H	4.2377831	3.7688807	1.3259072
	H	1.8459931	4.3567897	1.1786572
	H	-0.5480369	3.9331687	1.5423572
	H	1.2046451	-1.0174703	-3.6963528
	H	1.1640051	-2.7168203	-3.1226928
	H	1.6454551	-1.4061303	-2.0044228
	H	-1.2930459	-0.1543313	0.7070572
	C	-3.1901049	-1.1879123	0.9597372
	C	-5.8487849	-1.2724633	1.8742272
	C	-3.9996249	-2.3074023	0.7041172
	C	-3.7386859	-0.1103323	1.6738772
	C	-5.0550159	-0.1534223	2.1320572
	C	-5.3155449	-2.3482023	1.1575272
	H	-3.6040149	-3.1407723	0.1304472
	H	-3.1261659	0.7674977	1.8620072
	H	-5.4603859	0.6878277	2.6881672
	H	-5.9299449	-3.2194833	0.9469472
	H	-6.8762849	-1.3071233	2.2261572

Name	$TS_{Theo2O\text{-}BMA\text{-}S21}$
**Absolute Energies (Hartree)**	There are 15 positive frequencies below 100 cm^-1.<br><br>Zero-point correction: 0.533334 hartree<br>Enthalpy correction: 0.570859 hartree<br>Free Energy correction: 0.464697 hartree<br>Quasiharmonic Free Energy correction: 0.473568 hartree<br><br>SCF Energy: -1657.363566 hartree<br>SCF Energy + ZPVE: -1656.830232 hartree<br>Enthalpy: -1656.792707 hartree<br>Free Energy: -1656.898869 hartree<br><br>Free Energy with quasiharmonic correction: -1656.889998 hartree (correction: 5.57 kcal/mol)<br><br>---<br>Low frequencies before projection:<br>-353.7371<br>19.5933<br>25.0798<br><br>Low frequencies after projection:<br>-353.7369<br>18.4903<br>25.053<br>---
**Cartesian Coordinates**	N -5.0578205 -0.7113350 -1.1512778<br>C -5.6254595 0.4393850 -0.6508778<br>O -5.9682495 1.3882650 -1.3713078<br>N -5.8140495 0.4371750 0.7123222<br>N 3.9125405 0.2088720 -0.8724578<br>O -3.7695805 -0.7909950 2.3610422<br>N -2.8947005 -1.3378060 1.6232122<br>O -3.2287905 -2.1272160 0.6826122<br>C -1.5790805 -1.0709160 1.8499122<br>C 0.4620895 -1.2281870 -1.5032878<br>C -0.7599805 -0.8209160 -0.8514778<br>C -1.0041195 0.5959140 -0.5911078<br>O -0.1637395 1.4686330 -0.3776478<br>O -2.3262695 0.8307240 -0.5078878<br>C -2.7530095 2.1470540 -0.1116378<br>O 1.4684595 -0.5365370 -1.7071678<br>O 0.4323695 -2.5400470 -1.8333178<br>C 1.6344995 -3.0797270 -2.3993578<br>C 3.7717205 1.4943320 -0.4301678<br>N 2.5485205 2.0179330 -0.3803978<br>C -6.2461595 1.6535750 1.3684422<br>C -0.5816705 -1.6264660 1.0120422

	C	-4.5385405	-0.6962550	-2.5051078
	C	0.8415695	-1.4493570	1.4018222
	C	3.5418195	-1.1986480	2.1636722
	C	1.3246295	-0.2465170	1.9438022
	C	1.7423795	-2.5126670	1.2223622
	C	3.0796095	-2.3912780	1.6020722
	C	2.6592295	-0.1281070	2.3290722
	H	-3.6499405	-0.0561450	-2.6100878
	H	-4.2708005	-1.7188250	-2.7902978
	H	-5.3056205	-0.3290750	-3.1914478
	H	-4.5355305	-1.2847250	-0.4882478
	H	-5.2277305	-0.1846450	1.2716622
	H	-5.4640095	2.4291750	1.4037622
	H	-6.5393595	1.4149460	2.3954722
	H	-7.1067395	2.0740360	0.8421722
	H	1.4032985	-4.1184770	-2.6402678
	H	2.4547095	-3.0401170	-1.6747778
	H	1.9207395	-2.5366670	-3.3049178
	H	3.0514995	-0.2832980	-1.1683778
	H	1.7262905	1.4430230	-0.6025578
	H	-0.8145905	-2.6095960	0.6122422
	H	-1.3956805	-0.3230760	2.6062422
	H	0.6562805	0.6014430	2.0463622
	H	3.0131105	0.8054320	2.7583922
	H	4.5814995	-1.1013380	2.4644322
	H	3.7562285	-3.2321080	1.4711622
	H	1.3819685	-3.4448670	0.7957522
	H	-3.7774595	2.2442850	-0.4695678
	H	-2.1167395	2.9131640	-0.5611878
	H	-2.7150095	2.2297940	0.9792122
	H	-1.6463705	-1.3873060	-1.1131478
	C	2.2659615	3.3502230	0.1298922
	H	2.5895815	3.4594530	1.1726622
	H	1.1865915	3.4883830	0.0816222
	H	2.7532515	4.1230230	-0.4759578
	C	5.1025895	-0.4365380	-0.9359978
	H	5.0574395	-1.4550880	-1.3018178
	C	6.2611405	0.1862410	-0.5517678
	H	7.2088795	-0.3347490	-0.6039878
	C	6.1693205	1.5200910	-0.0920178
	H	7.0683305	2.0433910	0.2203322
	C	4.9582005	2.1709920	-0.0293778
	H	4.8904505	3.1906620	0.3265322

Name	$TS_{Theo2O-BMA-S23}$
**Absolute Energies (Hartree)**	There are 15 positive frequencies below 100 cm^-1.<br><br>Zero-point correction: 0.532980 hartree<br>Enthalpy correction: 0.570806 hartree<br>Free Energy correction: 0.462911 hartree<br>Quasiharmonic Free Energy correction: 0.472621 hartree<br><br>SCF Energy: -1657.365702 hartree<br>SCF Energy + ZPVE: -1656.832722 hartree<br>Enthalpy: -1656.794896 hartree<br>Free Energy: -1656.902791 hartree<br><br>Free Energy with quasiharmonic correction: -1656.893082 hartree (correction: 6.09 kcal/mol)<br><br>---<br>Low frequencies before projection:<br>-367.8014<br>14.4176<br>24.9302<br><br>Low frequencies after projection:<br>-367.8013<br>13.0826<br>24.1971<br>---
**Cartesian Coordinates**	N -2.3506450 2.8182122 0.0196386<br>C -3.4531450 2.1014932 0.2776186<br>C -5.6426650 0.4295632 0.8201186<br>C -4.7714250 2.5333032 -0.0211114<br>N -3.3110650 0.8641332 0.8379186<br>C -4.3558650 0.0418132 1.0989986<br>C -5.8394550 1.7041132 0.2508986<br>N 6.0446750 0.1253112 -0.8751314<br>C 5.9987550 0.4398012 0.4611486<br>O 6.9576050 0.9099012 1.0842286<br>N 4.7702450 0.1944312 1.0477786<br>N 2.5988050 -0.7862078 -1.4605814<br>O 3.3419350 0.0531322 -2.0583814<br>O 3.0907340 -1.6319078 -0.6476714<br>C 1.2589350 -0.7588078 -1.6890014<br>C 0.3818340 -1.6275578 -0.9846614<br>C 0.4052450 -1.1936078 0.9833386<br>C 0.4705550 0.2558022 0.9859386<br>O -0.4877950 1.0390222 0.9576686<br>O 1.7365650 0.6770522 0.8927786<br>C 1.9728050 2.0677922 0.6242986<br>C -0.7731460 -1.8502978 1.5361686

	O	-1.8560560	-1.3369978	1.8078886
	O	-0.5629460	-3.1815778	1.6664586
	C	-1.6926360	-3.9501078	2.1084686
	C	4.6556650	0.1964812	2.4918586
	C	7.1631050	0.5636112	-1.6818214
	C	-2.3692250	4.1413322	-0.5812314
	H	1.3541340	-1.6762078	1.1878586
	H	5.1625450	1.0757912	2.8972286
	H	5.1010050	-0.6936288	2.9643886
	H	3.5952250	0.2390712	2.7578786
	H	4.1433850	-0.4346288	0.5466886
	H	5.1530550	-0.0319088	-1.3483014
	H	8.0983050	0.3746412	-1.1483014
	H	7.1692650	-0.0011488	-2.6196914
	H	7.1274450	1.6380712	-1.9246114
	H	3.0539750	2.1473422	0.5199886
	H	0.9316050	0.0484522	-2.3261814
	H	-2.9092040	4.8627132	0.0440986
	H	-1.3361250	4.4776222	-0.6826214
	H	1.6126950	2.6874622	1.4512986
	H	1.4760350	2.3645722	-0.3044014
	H	-1.4555250	2.4102222	0.2899286
	H	-2.3621350	0.5046422	1.0476986
	H	-4.0752850	-0.9142868	1.5189086
	H	-6.4712450	-0.2343168	1.0328586
	H	-6.8451150	2.0420732	0.0181386
	H	-4.9218250	3.5107232	-0.4607614
	H	-2.8264550	4.1230732	-1.5777714
	H	-2.0057160	-3.6416378	3.1106786
	H	-1.3524160	-4.9867878	2.1235186
	H	-2.5330560	-3.8360478	1.4173286
	H	0.7839240	-2.6159978	-0.7777914
	C	-1.0525060	-1.6268478	-1.3808514
	C	-3.7765660	-1.6892768	-2.0968214
	C	-1.7233550	-0.4511078	-1.7570314
	C	-1.7762260	-2.8302778	-1.3572014
	C	-3.1249260	-2.8628968	-1.7107714
	C	-3.0685550	-0.4854968	-2.1210714
	H	-1.1944550	0.4963822	-1.7489514
	H	-1.2712360	-3.7447178	-1.0584314
	H	-3.6643960	-3.8063168	-1.6918514
	H	-3.5710750	0.4329232	-2.4129014
	H	-4.8265960	-1.7114768	-2.3757914

Name	$TS_{Theo2O\text{-}BMA\text{-}S3}$
Absolute Energies (Hartree)	There are 15 positive frequencies below 100 cm^-1.<br><br>Zero-point correction: 0.532947 hartree<br>Enthalpy correction: 0.570669 hartree<br>Free Energy correction: 0.463405 hartree<br>Quasiharmonic Free Energy correction: 0.473040 hartree<br><br>SCF Energy: -1657.373386 hartree<br>SCF Energy + ZPVE: -1656.840439 hartree<br>Enthalpy: -1656.802717 hartree<br>Free Energy: -1656.909981 hartree<br><br>Free Energy with quasiharmonic correction: -1656.900346 hartree (correction: 6.05 kcal/mol)<br><br>---<br>Low frequencies before projection:<br>-226.5666<br>17.6007<br>22.1297<br><br>Low frequencies after projection:<br>-226.5665<br>17.1891<br>21.7741<br>---
Cartesian Coordinates	N -4.0857999 -2.0567241 0.2591723<br>C -5.0074309 -1.0634441 0.5097423<br>O -6.2248809 -1.2354551 0.3970023<br>N -4.4518209 0.1545159 0.8450023<br>N 1.2280671 2.9215699 -0.3034677<br>H -3.1364999 -1.9346831 0.6133323<br>H -3.4745309 0.1553069 1.1377623<br>H 1.7389771 2.1468199 -0.7308777<br>O -1.5935419 0.2599579 1.5043423<br>N -0.7959909 -0.6750221 1.1914623<br>O -1.2166899 -1.8435221 0.9429723<br>C 0.5434391 -0.3922511 1.1572223<br>C 2.4929091 -0.4718991 -1.5871177<br>C 1.7097901 -1.6413201 -1.2618977<br>C 0.3214101 -1.6940211 -1.6853577<br>O -0.4158609 -0.7252211 -1.8758977<br>O -0.1155889 -2.9612011 -1.8261677<br>C -1.5178789 -3.0984221 -2.1119877<br>H -1.7234289 -4.1686221 -2.0528377<br>H -2.1139799 -2.5453621 -1.3840577<br>H -1.7436899 -2.7299121 -3.1182477<br>O 2.0735681 0.6871999 -1.6922077

	O	3.8077991	-0.7585881	-1.7068577	
	C	4.6826981	0.3595819	-1.9085677	
	H	5.6889691	-0.0607371	-1.9416977	
	H	4.4531181	0.8698519	-2.8492077	
	H	4.6051381	1.0704919	-1.0812677	
	H	-0.1178219	0.9883189	-1.2717977	
	C	-0.1090929	2.9159289	-0.4484977	
	C	-0.9626339	3.9787079	-0.0690377	
	N	-0.6900029	1.8006089	-0.9745577	
	C	-2.0297619	1.6453579	-1.0912277	
	H	-2.3445519	0.6826369	-1.4700977	
	C	-2.8873629	2.6586069	-0.7373277	
	H	-3.9554429	2.5182059	-0.8340877	
	C	-2.3293939	3.8427869	-0.2232377	
	H	-2.9785939	4.6637669	0.0674623	
	C	-5.3058319	1.1754649	1.4206023	
	H	-4.7238329	2.0948459	1.5347823	
	H	-6.1553619	1.3712649	0.7600723	
	H	-5.7085719	0.8949549	2.4057923	
	C	1.4929301	-1.3847701	0.8686323	
	H	0.7719781	0.6397299	1.3686623	
	H	-0.5345649	4.8851589	0.3390923	
	C	-4.5510489	-3.4200041	0.0895223	
	H	-5.2905089	-3.4702841	-0.7150377	
	H	-3.6971389	-4.0495831	-0.1761577	
	H	-5.0182489	-3.8287541	0.9977723	
	C	1.9766561	4.0751299	0.1628523	
	H	1.6866461	4.3503499	1.1834723	
	H	3.0354161	3.8089809	0.1672223	
	H	1.8405651	4.9472099	-0.4902677	
	H	2.2462601	-2.5823991	-1.2920077	
	H	1.1258401	-2.4035501	0.9405523	
	C	2.9125191	-1.1945391	1.2479023	
	C	5.6338691	-0.9200071	1.9230323	
	C	3.4792191	0.0765609	1.4470523	
	C	3.7373301	-2.3218481	1.3915423	
	C	5.0827101	-2.1885581	1.7276923	
	C	4.8253991	0.2106319	1.7828623	
	H	2.8703881	0.9680809	1.3344423	
	H	3.3147111	-3.3110091	1.2328323	
	H	5.7014111	-3.0752071	1.8366823	
	H	5.2446581	1.2017029	1.9373023	
	H	6.6829891	-0.8133671	2.1852123	

Name	$TS_{Theo2O\text{-}BMA\text{-}S33}$
**Absolute Energies (Hartree)**	There are 15 positive frequencies below 100 cm^-1.<br><br>Zero-point correction: 0.533055 hartree<br>Enthalpy correction: 0.570781 hartree<br>Free Energy correction: 0.463443 hartree<br>Quasiharmonic Free Energy correction: 0.472907 hartree<br><br>SCF Energy: -1657.376816 hartree<br>SCF Energy + ZPVE: -1656.843761 hartree<br>Enthalpy: -1656.806035 hartree<br>Free Energy: -1656.913373 hartree<br><br>Free Energy with quasiharmonic correction: -1656.903910 hartree (correction: 5.94 kcal/mol)<br><br>---<br>Low frequencies before projection:<br>-219.0504<br>14.0155<br>17.5044<br><br>Low frequencies after projection:<br>-219.0503<br>13.8975<br>16.7379<br>---
**Cartesian Coordinates**	N -2.3346149 0.9487389 -1.5414837<br>C -1.9113949 1.9826189 -0.8088637<br>C -0.8385949 4.0624489 0.7443863<br>C -2.7738949 2.8957489 -0.1439537<br>N -0.5664949 2.1878989 -0.6813237<br>C -0.0335849 3.1768689 0.0758563<br>C -2.2369549 3.9070189 0.6197163<br>N -3.9847749 -0.2038711 1.4028663<br>C -4.4410449 -1.4169311 0.9261863<br>O -5.5959649 -1.8153411 1.0989363<br>N -3.5119449 -2.1007311 0.1653463<br>N -0.2665449 -0.5066711 1.0703363<br>O -1.1036049 0.3104989 1.5581263<br>O -0.6431349 -1.5886011 0.5252963<br>C 1.0647651 -0.2052411 1.1707363<br>C 2.0453751 -1.0756411 0.6780663<br>C 2.3499051 -0.7289611 -1.4645737<br>C 1.0159551 -0.9561811 -1.9595337<br>O 0.1421451 -0.0905111 -2.1107137<br>O 0.7762451 -2.2554211 -2.2299337<br>C -0.5842749 -2.5794711 -2.5549037<br>C 2.8687451 0.6239789 -1.4147637

	O	2.2219551	1.6523689	-1.2142437
	O	4.2200751	0.6412389	-1.5274237
	C	4.8437751	1.9208989	-1.3619437
	C	-3.7939749	-3.4743411	-0.2096837
	C	-4.7667549	0.5077089	2.3954263
	C	-3.7336349	0.6787089	-1.8350937
	H	3.0689951	-1.5163711	-1.6536037
	H	-4.7848449	-3.5359711	-0.6666137
	H	-3.7744649	-4.1682911	0.6442663
	H	-3.0466249	-3.8022611	-0.9392237
	H	-2.5297549	-1.8735611	0.3283563
	H	-2.9732749	-0.0669311	1.4382663
	H	-4.7660749	0.0153989	3.3798563
	H	-4.3526449	1.5141789	2.5117463
	H	-5.8049949	0.5854789	2.0625563
	H	-0.6097549	-3.6661211	-2.6527037
	H	1.2581151	0.7450089	1.6414263
	H	-4.1549649	1.4478689	-2.4948737
	H	-3.7895149	-0.2883511	-2.3350537
	H	-0.8823949	-2.1124811	-3.4994837
	H	-1.2521449	-2.2518111	-1.7541237
	H	-1.6197149	0.3134389	-1.8965437
	H	0.0891751	1.5366689	-1.1474537
	H	1.0480551	3.1945089	0.0875563
	H	-0.4047449	4.8518089	1.3456363
	H	-2.9035949	4.5912189	1.1368363
	H	-3.8444049	2.7650889	-0.2283837
	H	-4.3191849	0.6180789	-0.9145637
	H	4.5354751	2.6106889	-2.1542337
	H	5.9171951	1.7334189	-1.4219437
	H	4.5941751	2.3583889	-0.3911637
	H	1.7011651	-2.0792411	0.4509263
	C	3.4487151	-0.9739911	1.1321363
	C	6.1359451	-0.8685211	1.9645663
	C	4.0055451	0.2240489	1.6118463
	C	4.2666651	-2.1143711	1.0717063
	C	5.5958751	-2.0646311	1.4862463
	C	5.3351951	0.2749089	2.0244463
	H	3.4032451	1.1262789	1.6470563
	H	3.8508951	-3.0459711	0.6951863
	H	6.2101751	-2.9596411	1.4363463
	H	5.7481651	1.2104989	2.3923663
	H	7.1725751	-0.8272911	2.2877863

Name	$TS_{Theo2O\text{-}BMB\text{-}R1}$
Absolute Energies (Hartree)	There are 15 positive frequencies below 100 cm^-1.<br><br>Zero-point correction: 0.532120 hartree<br>Enthalpy correction: 0.570090 hartree<br>Free Energy correction: 0.462764 hartree<br>Quasiharmonic Free Energy correction: 0.471566 hartree<br><br>SCF Energy: -1657.366857 hartree<br>SCF Energy + ZPVE: -1656.834737 hartree<br>Enthalpy: -1656.796767 hartree<br>Free Energy: -1656.904093 hartree<br><br>Free Energy with quasiharmonic correction: -1656.895291 hartree (correction: 5.52 kcal/mol)<br><br>---<br>Low frequencies before projection:<br>-86.9393<br>16.2282<br>26.8187<br><br>Low frequencies after projection:<br>-86.9293<br>15.5351<br>26.7644<br>---
Cartesian Coordinates	C 3.9653950 2.3879826 0.5343052<br>N 2.7325850 2.9971926 0.4091552<br>N 3.9770350 1.2857626 1.3497752<br>H 3.1027650 0.7875826 1.5055852<br>C 2.5189950 3.9993026 -0.6120948<br>H 1.9259950 2.4904526 0.7601352<br>H 1.6140050 4.5683726 -0.3746748<br>N -4.0517550 -0.4102874 1.0030652<br>C -4.0235340 -0.8413074 2.3909752<br>H -2.9769840 -0.8783774 2.6951952<br>H -4.5453550 -0.1235574 3.0328552<br>H -4.4733040 -1.8335274 2.5238352<br>H -0.5754140 -1.8714774 0.9294952<br>C 1.5753060 -1.8801174 1.2358752<br>C 0.3015160 -1.2447274 1.0169852<br>O 2.6351460 -1.3665974 1.5763552<br>C 0.1016050 0.1431226 1.2716052<br>O 0.9390450 1.0202426 1.4904952<br>C 0.4099060 -1.0470174 -1.4432248<br>C -0.4110150 0.0324126 -1.7390248<br>C 1.8484060 -1.0315974 -1.6768848<br>H -0.0640740 -2.0177074 -1.3701548

	N	-1.7638050	-0.0645174	-1.5725248
	H	-0.0684150	1.0120526	-2.0367148
	O	-2.3011940	-1.1354374	-1.1659748
	O	-2.4892750	0.9582626	-1.8198548
	C	2.5022660	-2.2459874	-1.9610748
	C	2.6094150	0.1505226	-1.6151648
	C	3.9782550	0.1251926	-1.8646148
	C	3.8701160	-2.2693974	-2.2182948
	C	4.6095260	-1.0837274	-2.1738348
	H	1.9269060	-3.1672174	-1.9799048
	H	2.1368750	1.0823526	-1.3254548
	H	4.5562250	1.0394126	-1.7726248
	H	4.3610060	-3.2114374	-2.4476648
	H	5.6794060	-1.1018974	-2.3633848
	H	3.3703550	4.6832226	-0.6360048
	H	2.4052950	3.5738426	-1.6234548
	C	5.1810250	0.4879326	1.4598552
	H	6.0443050	1.1411626	1.6100652
	H	5.0774650	-0.1803274	2.3180752
	H	5.3654550	-0.1275474	0.5676352
	C	-5.1665550	-0.1052774	0.3308652
	C	-7.3734850	0.6081526	-1.2537448
	C	-6.4802950	-0.1762174	0.8657752
	N	-5.0360050	0.3322826	-0.9567948
	C	-6.0906150	0.6753926	-1.7353448
	C	-7.5550850	0.1757326	0.0783652
	H	-6.6188250	-0.5225674	1.8819752
	H	-5.8365550	1.0014626	-2.7372948
	H	-8.5579250	0.1109126	0.4903052
	H	-8.2111850	0.8826826	-1.8826548
	O	4.9778650	2.8279526	-0.0301348
	O	1.4912360	-3.2249574	0.9658952
	O	-1.2347650	0.4786426	1.1782252
	C	-1.5289150	1.8765626	1.2727452
	H	-2.6161150	1.9532126	1.2275952
	H	-1.1586750	2.2950326	2.2132452
	H	-1.0857050	2.4273426	0.4372252
	C	2.7154160	-3.9481974	1.1321752
	H	3.0407960	-3.9366174	2.1782752
	H	2.4979160	-4.9732474	0.8223452
	H	3.5116360	-3.5259174	0.5124652
	H	-4.0704650	0.4585726	-1.3440548
	H	-3.1911750	-0.5235274	0.4638252

<table>
<tr><td>Name</td><td>TS_{Theo2O-BMB-R2}</td></tr>
<tr><td>Absolute Energies (Hartree)</td><td>There are 16 positive frequencies below 100 cm^-1.

Zero-point correction: 0.532300 hartree
Enthalpy correction: 0.570141 hartree
Free Energy correction: 0.462362 hartree
Quasiharmonic Free Energy correction: 0.472205 hartree

SCF Energy: -1657.367980 hartree
SCF Energy + ZPVE: -1656.835680 hartree
Enthalpy: -1656.797839 hartree
Free Energy: -1656.905618 hartree

Free Energy with quasiharmonic correction: -1656.895776 hartree (correction: 6.18 kcal/mol)

Low frequencies before projection:
-156.4162
12.3369
19.8105

Low frequencies after projection:
-156.4158
8.0317
19.7001
---</td></tr>
<tr><td>Cartesian Coordinates</td><td>C -2.4808208 1.8807515 -0.5889663
N -1.5263108 2.4808015 0.2157837
N -2.0372008 0.7775315 -1.2576863
H -1.0406508 0.5746515 -1.3064563
C -1.7282798 3.8425415 0.6720637
H -0.5670708 2.2371415 -0.0161563
H -1.4184998 4.5928115 -0.0725763
N -3.0268508 0.1061615 1.8777037
C -3.6023308 1.2993725 2.4815337
H -2.8061708 1.8135915 3.0208137
H -4.0035308 1.9689725 1.7136537
H -4.3900508 1.0305925 3.1949537
H 3.4063892 -0.2435195 -1.6368363
C 1.4045192 -1.0369195 -1.7354563
C 2.3681392 -0.0076795 -1.4528163
O 0.1794092 -0.9547285 -1.7646563
C 2.0162592 1.3845105 -1.3755663
O 0.8967992 1.8931815 -1.3173463
C 2.4681892 -0.4477895 0.8641937
C 1.6108192 -1.5179995 1.1000037
C 3.9281592 -0.5669295 0.9344937
H 2.0532292 0.5353005 1.0538737</td></tr>
</table>

	N	0.2635592	-1.3170585	1.2314537
	H	1.8920282	-2.5585395	1.0612737
	O	-0.2232008	-0.1536585	1.2974137
	O	-0.4907118	-2.3445885	1.3173937
	C	4.6885092	0.5834505	1.2106337
	C	4.6007582	-1.7844195	0.7190137
	C	5.9888882	-1.8477205	0.7975137
	C	6.0773792	0.5163095	1.2939737
	C	6.7328492	-0.7000005	1.0902237
	H	4.1798792	1.5326705	1.3525037
	H	4.0372782	-2.6748895	0.4590637
	H	6.4946282	-2.7939305	0.6244837
	H	6.6485892	1.4142895	1.5135637
	H	7.8164592	-0.7536105	1.1513837
	H	-2.7881998	3.9956115	0.8821637
	H	-1.1556298	4.0134615	1.5908037
	C	-2.8788308	0.1140315	-2.2281263
	H	-3.0009908	0.6976115	-3.1532763
	H	-2.4150408	-0.8422085	-2.4807963
	H	-3.8741608	-0.0625175	-1.8125063
	C	-3.7258108	-0.7249675	1.1015037
	C	-4.9899418	-2.6060975	-0.5586163
	C	-5.1155408	-0.6131075	0.8272437
	N	-3.0570318	-1.7676385	0.5273437
	C	-3.6498818	-2.6814975	-0.2776163
	C	-5.7243008	-1.5432375	0.0149737
	H	-5.6691708	0.2192125	1.2401837
	H	-2.9903618	-3.4486285	-0.6661963
	H	-6.7846308	-1.4460975	-0.1992663
	H	-5.4575018	-3.3371675	-1.2063963
	O	-3.6626508	2.2600125	-0.6272363
	O	2.0227482	-2.2519195	-1.8668463
	O	3.1437092	2.1584005	-1.2895663
	C	2.9117402	3.5578805	-1.1006563
	H	3.9016302	4.0151805	-1.0394363
	H	2.3521402	3.9843205	-1.9394363
	H	2.3534302	3.7472405	-0.1770063
	C	1.1322982	-3.3716395	-1.9438463
	H	0.5220382	-3.3311185	-2.8524263
	H	1.7726282	-4.2563895	-1.9658063
	H	0.4688482	-3.4035285	-1.0725363
	H	-2.0472018	-1.8958785	0.7617637
	H	-2.0053708	0.0600215	1.7871737

Name	$TS_{Theo20\text{-}BMB\text{-}S1}$
**Absolute Energies (Hartree)**	There are 16 positive frequencies below 100 cm^-1.<br><br>Zero-point correction: 0.532300 hartree<br>Enthalpy correction: 0.570141 hartree<br>Free Energy correction: 0.462362 hartree<br>Quasiharmonic Free Energy correction: 0.472205 hartree<br><br>SCF Energy: -1657.367980 hartree<br>SCF Energy + ZPVE: -1656.835680 hartree<br>Enthalpy: -1656.797839 hartree<br>Free Energy: -1656.905618 hartree<br><br>Free Energy with quasiharmonic correction: -1656.895776 hartree (correction: 6.18 kcal/mol)<br><br>---<br>Low frequencies before projection:<br>-156.4162<br>12.3369<br>19.8105<br><br>Low frequencies after projection:<br>-156.4158<br>8.0317<br>19.7001<br>---
**Cartesian Coordinates**	C -2.4808208 1.8807515 0.5889663<br>N -1.5263108 2.4808015 -0.2157837<br>N -2.0372008 0.7775315 1.2576863<br>H -1.0406508 0.5746515 1.3064563<br>C -1.7282798 3.8425415 -0.6720637<br>H -0.5670708 2.2371415 0.0161563<br>H -1.4184998 4.5928115 0.0725763<br>N -3.0268508 0.1061615 -1.8777037<br>C -3.6023308 1.2993725 -2.4815337<br>H -2.8061708 1.8135915 -3.0208137<br>H -4.0035308 1.9689725 -1.7136537<br>H -4.3900508 1.0305925 -3.1949537<br>H 3.4063892 -0.2435195 1.6368363<br>C 1.4045192 -1.0369195 1.7354563<br>C 2.3681392 -0.0076795 1.4528163<br>O 0.1794092 -0.9547285 1.7646563<br>C 2.0162592 1.3845105 1.3755663<br>O 0.8967992 1.8931815 1.3173463<br>C 2.4681892 -0.4477895 -0.8641937<br>C 1.6108192 -1.5179995 -1.1000037<br>C 3.9281592 -0.5669295 -0.9344937<br>H 2.0532292 0.5353005 -1.0538737

	N	0.2635592	-1.3170585	-1.2314537
	H	1.8920282	-2.5585395	-1.0612737
	O	-0.2232008	-0.1536585	-1.2974137
	O	-0.4907118	-2.3445885	-1.3173937
	C	4.6885092	0.5834505	-1.2106337
	C	4.6007582	-1.7844195	-0.7190137
	C	5.9888882	-1.8477205	-0.7975137
	C	6.0773792	0.5163095	-1.2939737
	C	6.7328492	-0.7000005	-1.0902237
	H	4.1798792	1.5326705	-1.3525037
	H	4.0372782	-2.6748895	-0.4590637
	H	6.4946282	-2.7939305	-0.6244837
	H	6.6485892	1.4142895	-1.5135637
	H	7.8164592	-0.7536105	-1.1513837
	H	-2.7881998	3.9956115	-0.8821637
	H	-1.1556298	4.0134615	-1.5908037
	C	-2.8788308	0.1140315	2.2281263
	H	-3.0009908	0.6976115	3.1532763
	H	-2.4150408	-0.8422085	2.4807963
	H	-3.8741608	-0.0625175	1.8125063
	C	-3.7258108	-0.7249675	-1.1015037
	C	-4.9899418	-2.6060975	0.5586163
	C	-5.1155408	-0.6131075	-0.8272437
	N	-3.0570318	-1.7676385	-0.5273437
	C	-3.6498818	-2.6814975	0.2776163
	C	-5.7243008	-1.5432375	-0.0149737
	H	-5.6691708	0.2192125	-1.2401837
	H	-2.9903618	-3.4486285	0.6661963
	H	-6.7846308	-1.4460975	0.1992663
	H	-5.4575018	-3.3371675	1.2063963
	O	-3.6626508	2.2600125	0.6272363
	O	2.0227482	-2.2519195	1.8668463
	O	3.1437092	2.1584005	1.2895663
	C	2.9117402	3.5578805	1.1006563
	H	3.9016302	4.0151805	1.0394363
	H	2.3521402	3.9843205	1.9394363
	H	2.3534302	3.7472405	0.1770063
	C	1.1322982	-3.3716395	1.9438463
	H	0.5220382	-3.3311185	2.8524263
	H	1.7726282	-4.2563895	1.9658063
	H	0.4688482	-3.4035285	1.0725363
	H	-2.0472018	-1.8958785	-0.7617637
	H	-2.0053708	0.0600215	-1.7871737

Name	$TS_{Theo2O\text{-}BMB\text{-}S2}$
**Absolute Energies (Hartree)**	There are 15 positive frequencies below 100 cm^-1.<br><br>Zero-point correction: 0.532120 hartree<br>Enthalpy correction: 0.570090 hartree<br>Free Energy correction: 0.462764 hartree<br>Quasiharmonic Free Energy correction: 0.471566 hartree<br><br>SCF Energy: -1657.366857 hartree<br>SCF Energy + ZPVE: -1656.834737 hartree<br>Enthalpy: -1656.796767 hartree<br>Free Energy: -1656.904093 hartree<br><br>Free Energy with quasiharmonic correction: -1656.895291 hartree (correction: 5.52 kcal/mol)<br><br>--<br>Low frequencies before projection:<br>-86.9393<br>16.2282<br>26.8187<br><br>Low frequencies after projection:<br>-86.9293<br>15.5351<br>26.7644<br>--
**Cartesian Coordinates**	C 3.9653950 2.3879826 -0.5343052<br>N 2.7325850 2.9971926 -0.4091552<br>N 3.9770350 1.2857626 -1.3497752<br>H 3.1027650 0.7875826 -1.5055852<br>C 2.5189950 3.9993026 0.6120948<br>H 1.9259950 2.4904526 -0.7601352<br>H 1.6140050 4.5683726 0.3746748<br>N -4.0517550 -0.4102874 -1.0030652<br>C -4.0235340 -0.8413074 -2.3909752<br>H -2.9769840 -0.8783774 -2.6951952<br>H -4.5453550 -0.1235574 -3.0328552<br>H -4.4733040 -1.8335274 -2.5238352<br>H -0.5754140 -1.8714774 -0.9294952<br>C 1.5753060 -1.8801174 -1.2358752<br>C 0.3015160 -1.2447274 -1.0169852<br>O 2.6351460 -1.3665974 -1.5763552<br>C 0.1016050 0.1431226 -1.2716052<br>O 0.9390450 1.0202426 -1.4904952<br>C 0.4099060 -1.0470174 1.4432248<br>C -0.4110150 0.0324126 1.7390248<br>C 1.8484060 -1.0315974 1.6768848<br>H -0.0640740 -2.0177074 1.3701548

	N	-1.7638050	-0.0645174	1.5725248
	H	-0.0684150	1.0120526	2.0367148
	O	-2.3011940	-1.1354374	1.1659748
	O	-2.4892750	0.9582626	1.8198548
	C	2.5022660	-2.2459874	1.9610748
	C	2.6094150	0.1505226	1.6151648
	C	3.9782550	0.1251926	1.8646148
	C	3.8701160	-2.2693974	2.2182948
	C	4.6095260	-1.0837274	2.1738348
	H	1.9269060	-3.1672174	1.9799048
	H	2.1368750	1.0823526	1.3254548
	H	4.5562250	1.0394126	1.7726248
	H	4.3610060	-3.2114374	2.4476648
	H	5.6794060	-1.1018974	2.3633848
	H	3.3703550	4.6832226	0.6360048
	H	2.4052950	3.5738426	1.6234548
	C	5.1810250	0.4879326	-1.4598552
	H	6.0443050	1.1411626	-1.6100652
	H	5.0774650	-0.1803274	-2.3180752
	H	5.3654550	-0.1275474	-0.5676352
	C	-5.1665550	-0.1052774	-0.3308652
	C	-7.3734850	0.6081526	1.2537448
	C	-6.4802950	-0.1762174	-0.8657752
	N	-5.0360050	0.3322826	0.9567948
	C	-6.0906150	0.6753926	1.7353448
	C	-7.5550850	0.1757326	-0.0783652
	H	-6.6188250	-0.5225674	-1.8819752
	H	-5.8365550	1.0014626	2.7372948
	H	-8.5579250	0.1109126	-0.4903052
	H	-8.2111850	0.8826826	1.8826548
	O	4.9778650	2.8279526	0.0301348
	O	1.4912360	-3.2249574	-0.9658952
	O	-1.2347650	0.4786426	-1.1782252
	C	-1.5289150	1.8765626	-1.2727452
	H	-2.6161150	1.9532126	-1.2275952
	H	-1.1586750	2.2950326	-2.2132452
	H	-1.0857050	2.4273426	-0.4372252
	C	2.7154160	-3.9481974	-1.1321752
	H	3.0407960	-3.9366174	-2.1782752
	H	2.4979160	-4.9732474	-0.8223452
	H	3.5116360	-3.5259174	-0.5124652
	H	-4.0704650	0.4585726	1.3440548
	H	-3.1911750	-0.5235274	-0.4638252

Name	$TS_{Zhang\ et\ al.,\ 2009}$
**Absolute Energies (Hartree)**	There are 22 positive frequencies below 100 cm^-1.<br><br>Zero-point correction: 0.835524 hartree<br>Enthalpy correction: 0.891691 hartree<br>Free Energy correction: 0.742987 hartree<br>Quasiharmonic Free Energy correction: 0.761339 hartree<br><br>SCF Energy: -3081.229686 hartree<br>SCF Energy*: -3082.100734 hartree<br>SCF Energy + ZPVE: -3080.394162 hartree<br>Enthalpy: -3080.337995 hartree<br>Free Energy: -3080.486699 hartree<br><br>Free Energy with quasiharmonic correction: -3080.468347 hartree (correction: 11.52 kcal/mol)<br><br>---<br>Low frequencies before projection:<br>-228.2008<br>5.5773<br>10.0379<br><br>Low frequencies after projection:<br>-228.2008<br>4.9257<br>9.223<br>---
**Cartesian Coordinates**	N -2.0200780 -1.0611590 2.0389990<br>O -1.0928710 -0.2206740 2.3187370<br>O -1.9386620 -2.2500800 2.4515150<br>C -3.0797810 -0.6192330 1.3018160<br>C -4.0797190 -1.5130190 0.8963390<br>C -3.3587910 -2.6260150 -0.8826110<br>C -2.2458910 -3.3611510 -0.3211510<br>O -1.0548440 -3.0425190 -0.3232790<br>O -2.6728880 -4.5073270 0.2693140<br>C -1.6823170 -5.2239480 1.0127890<br>C -3.1269300 -1.5077430 -1.7627870<br>O -2.1456020 -0.7579700 -1.7523890<br>O -4.1806360 -1.2880000 -2.5839790<br>C -4.1106250 -0.1064840 -3.3961250<br>H -4.2628710 -3.1964590 -1.0532190<br>H -2.1991230 -6.0878900 1.4357090<br>H -3.0143310 0.4198620 1.0206710<br>H -0.8647020 -5.5597430 0.3662300<br>H -1.2842050 -4.5922610 1.8123260<br>H -3.2508890 -0.1483710 -4.0720960

	H	-5.0402100	-0.0922180	-3.9675470
	H	-4.0360220	0.7917350	-2.7794050
	H	-4.1033150	-2.4575600	1.4295430
	C	-5.3780610	-1.0443780	0.3757170
	C	-7.8896170	-0.2497750	-0.6157290
	C	-5.5607280	0.2380750	-0.1698050
	C	-6.4768990	-1.9204260	0.4104500
	C	-7.7208360	-1.5282400	-0.0775210
	C	-6.8052150	0.6298820	-0.6583720
	H	-4.7343700	0.9371310	-0.2157040
	H	-6.3458010	-2.9183030	0.8224110
	H	-8.5586270	-2.2193220	-0.0382550
	H	-6.9266380	1.6265540	-1.0743570
	H	-8.8588780	0.0584370	-0.9984830
	C	4.2631750	-0.4695030	-1.5148290
	C	5.2925780	-2.0230850	-3.6037860
	C	5.4100230	-0.0715250	-2.1821490
	C	3.6000730	-1.6727910	-1.8571570
	C	4.1170870	-2.4593930	-2.9435100
	C	5.9367580	-0.8630700	-3.2363910
	N	3.5301510	-3.6123200	-3.3803440
	C	2.4325780	-4.0041810	-2.7670120
	C	1.8534680	-3.3275160	-1.6665020
	C	2.4345630	-2.1730830	-1.1874250
	C	2.1684420	-0.5753150	2.9172760
	C	1.7045180	-2.3996520	1.2324250
	C	3.4166980	-2.7376750	3.0732290
	C	3.0235600	-3.1159990	1.6311960
	C	3.5563130	-1.2017320	3.2213980
	N	1.2106050	-1.6450750	2.4537300
	H	0.2914310	-1.1622430	2.2607290
	O	5.9663390	1.0973440	-1.7600060
	C	2.3078990	-3.2266290	4.0249980
	C	0.9595400	-2.6216670	3.5728340
	C	1.8622870	-1.4469350	0.0259590
	H	3.8737230	0.2010420	-0.7569150
	H	5.6684490	-2.6360410	-4.4174220
	H	6.8337040	-0.5580400	-3.7628620
	H	1.9658010	-4.9159240	-3.1382340
	H	0.9455870	-3.7178380	-1.2195840
	H	2.1914040	0.1938700	2.1445230
	H	1.7050360	-0.1313710	3.8008100
	H	0.9023490	-3.1054670	1.0125550
	H	4.3675260	-3.2160290	3.3294880
	H	2.8939740	-4.2001390	1.5545130
	H	3.8216780	-2.8499820	0.9342970
	H	3.7914370	-0.9972920	4.2740820
	H	2.5356050	-2.9283180	5.0538450
	H	2.2505750	-4.3199500	4.0086610
	H	0.2573550	-3.3596130	3.1868930

	H	0.4474340	-2.0629330	4.3579900
	H	2.5621110	-0.6695180	0.3124040
	C	7.1121860	1.5988790	-2.4405460
	H	7.3569190	2.5437650	-1.9505730
	H	6.9037770	1.7886440	-3.5016860
	H	7.9687940	0.9173190	-2.3523670
	C	4.6981290	-0.6043110	2.3792970
	H	5.6255120	-1.1270870	2.6503330
	H	4.5378670	-0.8062040	1.3147340
	C	4.8889890	0.9034780	2.5702130
	H	4.0127040	1.4699940	2.2341680
	H	5.7442460	1.2580600	1.9838540
	H	5.0725360	1.1547020	3.6226590
	C	0.5150770	0.5756900	-0.2077430
	N	-0.6574190	1.2163750	-0.5392030
	N	1.4547040	1.4031800	0.2429810
	H	-1.4543250	0.7482660	-0.9720430
	C	-0.4727780	2.5495960	-0.2672280
	C	0.5148550	5.0107210	0.4839100
	C	0.8623990	2.6532160	0.2142630
	C	-1.3133700	3.6565770	-0.3588240
	C	-0.8032670	4.9005190	0.0245870
	C	1.3612470	3.8971900	0.5882000
	H	-1.4357860	5.7780950	-0.0286250
	H	2.3756240	3.9995360	0.9584610
	C	-2.7389760	3.4753190	-0.7584000
	C	1.0601620	6.3675030	0.8162340
	F	-2.8555110	2.6575100	-1.8422140
	F	-3.3612370	4.6308950	-1.0537180
	F	-3.4696550	2.8738350	0.2276660
	F	1.9747670	6.3192150	1.8156330
	F	1.6871010	6.9426760	-0.2436070
	F	0.0919890	7.2329890	1.2019910
	N	0.6172990	-0.7669280	-0.2861130
	H	-0.1864850	-1.3227120	-0.5890930

<table>
<tr><td>Name</td><td>TS$_{\text{Lai et al., 2015}}$</td></tr>
<tr><td>Absolute Energies (Hartree)</td><td>There are 24 positive frequencies below 100 cm^-1.

Zero-point correction: 0.916223 hartree
Enthalpy correction: 0.974661 hartree
Free Energy correction: 0.823086 hartree
Quasiharmonic Free Energy correction: 0.839474 hartree

SCF Energy: -3811.877321 hartree
SCF Energy*: -3812.664852 hartree
SCF Energy + ZPVE: -3810.961098 hartree
Enthalpy: -3810.902660 hartree
Free Energy: -3811.054235 hartree

Free Energy with quasiharmonic correction: -3811.037847 hartree (correction: 10.28 kcal/mol)

Low frequencies before projection:
-154.5979
12.2864
18.5914

Low frequencies after projection:
-154.5976
11.8879
18.5025
--</td></tr>
<tr><td>Cartesian Coordinates</td><td>N 0.9449910 -0.7115020 -1.6837650
O 0.3182190 0.2655810 -1.1401670
O 0.4731910 -1.2918670 -2.6885910
C 2.1601490 -1.0231590 -1.1267830
C 2.9608100 -2.0349490 -1.6305390
C 2.2766030 -4.0012370 -0.4780800
C 0.8803970 -3.7836870 -0.7097020
O 0.1364090 -2.9836240 -0.1293750
O 0.4440420 -4.5587730 -1.7440260
C -0.8721250 -4.2890800 -2.2235210
C 2.9010730 -3.6652550 0.7728200
O 2.5065120 -2.8598650 1.6135760
O 4.0834740 -4.3292110 0.9262360
C 4.8167750 -4.0139440 2.1134720
H 2.7565570 -4.7688030 -1.0714140
H -1.1010660 -5.0851440 -2.9362050
H 2.3746700 -0.4492150 -0.2382620
H -1.6023140 -4.2940930 -1.4100250
H -0.8856930 -3.3174590 -2.7266210
H 4.2780430 -4.3482920 3.0071970
H 5.7640300 -4.5499830 2.0251710
H 5.0026780 -2.9396410 2.1915290</td></tr>
</table>

	H	2.6388370	-2.5044140	-2.5539850
	C	4.3917690	-2.1197150	-1.3087060
	C	7.1451740	-2.3257520	-0.7625290
	C	4.9756860	-1.3684870	-0.2708810
	C	5.2141210	-2.9754900	-2.0623810
	C	6.5762260	-3.0782170	-1.7939330
	C	6.3392830	-1.4711450	-0.0057340
	H	4.3677380	-0.7069560	0.3360390
	H	4.7715410	-3.5684870	-2.8591530
	H	7.1947920	-3.7456510	-2.3880840
	H	6.7730890	-0.8772590	0.7944270
	H	8.2087030	-2.4027060	-0.5525260
	C	-5.7366550	-0.8871970	0.9709550
	C	-7.0222430	-2.4452460	-0.9642010
	C	-7.0942800	-1.1457220	1.0714210
	C	-4.9702830	-1.3965800	-0.1042140
	C	-5.6327910	-2.2002740	-1.0956030
	C	-7.7470580	-1.9348720	0.0881890
	N	-4.9928720	-2.7435850	-2.1717250
	C	-3.6998800	-2.5247020	-2.2809550
	C	-2.9456400	-1.7498510	-1.3662340
	C	-3.5644310	-1.1635940	-0.2810790
	C	-1.3616090	2.7872570	-1.1846210
	C	-2.6683710	1.2283510	0.1734130
	C	-3.7960970	3.2312390	-0.8032430
	C	-3.9470470	2.0796850	0.2150720
	C	-2.3745950	3.8586650	-0.7037410
	N	-2.0432440	1.4355530	-1.2008290
	H	-1.2699830	0.7408530	-1.3540580
	O	-7.7248520	-0.6003420	2.1460510
	C	-3.9580690	2.6423810	-2.2168200
	C	-3.0388410	1.4077660	-2.3340210
	C	-2.7961520	-0.2480010	0.6555140
	H	-5.2869180	-0.2875600	1.7533400
	H	-7.4978620	-3.0553440	-1.7260090
	H	-8.8081020	-2.1436380	0.1601540
	H	-3.2023290	-2.9647770	-3.1434630
	H	-1.8866350	-1.6108710	-1.5625040
	H	-0.4799630	2.6922710	-0.5551550
	H	-1.0190580	2.9742420	-2.2041990
	H	-1.9480120	1.6689240	0.8624650
	H	-4.5521550	4.0013670	-0.6228380
	H	-4.0725690	2.4529320	1.2353500
	H	-4.8345810	1.4870420	-0.0307480
	H	-2.3450080	4.6884240	-1.4240420
	H	-3.7026270	3.3928890	-2.9729020
	H	-4.9956440	2.3408920	-2.3906510
	H	-3.5924740	0.4765120	-2.2617310
	H	-2.4575770	1.3878810	-3.2587730
	C	-9.1150350	-0.8519520	2.3304730

	H	-9.3889990	-0.3325290	3.2510660
	H	-9.3215820	-1.9236550	2.4482470
	H	-9.7140790	-0.4518980	1.5016220
	C	-2.1228530	4.4480210	0.6675310
	H	-2.9429180	5.0754640	1.0216840
	C	-1.0524340	4.2983710	1.4496730
	H	-1.0066680	4.7851650	2.4204110
	H	-0.1800910	3.7139810	1.1711670
	H	-3.3415610	-0.1460950	1.5955760
	N	-1.4935490	-0.8018990	1.0003870
	H	-1.0417400	-1.4574390	0.3672130
	C	-0.7838320	-0.4154490	2.0990350
	N	0.4622480	-0.9143950	2.1763150
	H	0.7816380	-1.5972580	1.4849900
	S	-1.4442640	0.6277550	3.2932680
	C	1.4517730	-0.5260530	3.1659070
	H	2.2485100	-1.2663780	3.1063630
	H	1.0121410	-0.5532830	4.1681250
	C	2.0253130	0.8787690	2.9272140
	C	3.1201670	1.2181770	3.9623120
	C	3.5482390	1.8651560	1.5576720
	N	2.5820100	1.0411190	1.5776000
	S	4.1536460	2.5003430	3.1336320
	S	4.3659050	2.4662890	0.1051050
	C	3.0215520	2.2871480	-1.1674030
	H	2.2211600	1.7109040	-0.7014580
	H	3.4433360	1.7112560	-1.9941020
	C	2.5074230	3.6281820	-1.6142060
	C	1.5213330	6.0908740	-2.3164840
	C	2.9811640	4.2428110	-2.7792710
	C	2.4791080	5.4939050	-3.1352140
	C	1.1063580	5.4000480	-1.1759310
	H	3.7294650	3.7460710	-3.3901640
	H	2.8306440	5.9925850	-4.0344290
	H	0.3531570	5.8200070	-0.5117550
	H	1.1039770	7.0655280	-2.5505760
	N	1.5786480	4.1984230	-0.8267910
	H	2.7042600	1.6192250	4.8890570
	H	3.7452060	0.3481540	4.1926350
	H	1.2048400	1.6017010	3.0140760

1.6. CARTESIAN COORDINATES OF CATALYST CONFORMER LIBRARY

**Name**	**Cat 2O_conf1**
**Cartesian Coordinates**	N 2.90463 0.11404 -1.53438<br>N 0.59201 0.07545 -1.62185<br>N 3.14254 -0.22279 1.33516<br>N 2.19943 -2.15248 0.40280<br>N -1.54973 -1.13492 2.06077<br>C 4.13244 0.51506 -0.84358<br>C 5.57168 0.19485 1.21133<br>C 6.63367 0.81942 -1.01157<br>C 6.81619 0.07244 0.31850<br>C 5.36351 0.36146 -1.74541<br>C 4.31621 -0.28955 0.47287<br>C 1.73444 0.78936 -1.25514<br>C -0.72572 0.34970 -1.24385<br>C -3.41389 0.71718 -0.46383<br>C -1.13752 1.56289 -0.67159<br>C -1.67813 -0.66347 -1.44370<br>C -2.99883 -0.47679 -1.05333<br>C -2.46844 1.72372 -0.28629<br>C -2.86353 2.99422 0.41588<br>C -4.00195 -1.57018 -1.29312<br>C 2.03794 -1.04217 1.15171<br>C -0.13216 -2.68602 0.81279<br>C 0.81815 -0.68764 1.75391<br>C 1.11648 -2.93952 0.27606<br>C -0.31825 -1.49379 1.56803<br>C -1.66817 0.03980 2.91284<br>C -2.64388 -2.09658 2.06978<br>O 1.69571 1.91708 -0.76870<br>F -2.18543 4.06493 -0.04814<br>F -2.61056 2.92163 1.75051<br>F -4.18290 3.25755 0.28722<br>F -3.42366 -2.79894 -1.26804<br>F -4.97597 -1.57085 -0.35378<br>F -4.61182 -1.45232 -2.49383<br>H 4.00489 1.57213 -0.59011<br>H 5.43324 1.24543 1.50940<br>H 5.69202 -0.38526 2.13301<br>H 6.56144 1.89853 -0.81304<br>H 7.51100 0.67541 -1.65348<br>H 7.00403 -0.99120 0.11259<br>H 7.69797 0.45080 0.84945<br>H 5.21265 0.93721 -2.66558<br>H 5.46572 -0.69374 -2.04014<br>H 4.44689 -1.34433 0.21242<br>H -4.44144 0.85519 -0.15172<br>H -0.41770 2.35327 -0.52119

	H	-1.37489	-1.61559	-1.86576
	H	-0.93876	-3.38433	0.63565
	H	0.76987	0.22701	2.33088
	H	1.26575	-3.84367	-0.31397
	H	-2.72325	0.22720	3.11798
	H	-1.14081	-0.08238	3.87113
	H	-1.27617	0.92579	2.40680
	H	-2.79397	-2.52847	1.07896
	H	-2.47683	-2.91604	2.78488
	H	-3.56762	-1.58095	2.33779
	H	2.91065	0.71471	1.64209
	H	0.74442	-0.87778	-1.92533
	H	2.81476	-0.89491	-1.60969

Name	Cat 2O_conf2			
Cartesian Coordinates	N	2.91335	-0.56336	-1.46405
	N	0.61202	-0.60455	-1.61064
	N	3.16046	0.36842	1.28758
	N	2.04701	-1.69484	1.23154
	N	-1.54032	0.21176	2.38904
	C	4.15189	0.05862	-0.99216
	C	5.59901	0.56118	1.02313
	C	6.66517	0.16770	-1.24772
	C	6.82884	0.02789	0.27301
	C	5.37165	-0.50149	-1.73578
	C	4.31192	-0.12423	0.54190
	C	1.75634	0.18756	-1.50352
	C	-0.70646	-0.23288	-1.34005
	C	-3.39595	0.33861	-0.70132
	C	-1.10687	1.08848	-1.08091
	C	-1.66778	-1.25255	-1.29834
	C	-2.99232	-0.96384	-0.98252
	C	-2.43534	1.34911	-0.75656
	C	-2.86560	2.76424	-0.48292
	C	-3.96006	-2.10608	-0.85725
	C	1.99561	-0.36642	1.45679
	C	-0.30275	-1.81831	1.83391
	C	0.82940	0.30267	1.86814
	C	0.90150	-2.37031	1.43828
	C	-0.36670	-0.41271	2.04668
	C	-2.69727	-0.58023	2.78631
	C	-1.54735	1.64040	2.66442
	O	1.72868	1.41527	-1.48537
	F	-3.77176	2.81851	0.52645
	F	-3.45203	3.33428	-1.55995
	F	-1.82809	3.55817	-0.12998
	F	-3.79608	-2.76499	0.32459
	F	-5.24523	-1.70095	-0.90371
	F	-3.78300	-3.02832	-1.82974
	H	4.05845	1.12774	-1.20843
	H	5.51051	1.64615	0.85874
	H	5.70198	0.41025	2.10346
	H	6.63841	1.23463	-1.51266
	H	7.52998	-0.26345	-1.76620
	H	6.96981	-1.03301	0.52551
	H	7.73036	0.55384	0.60965
	H	5.23583	-0.35608	-2.81346
	H	5.42997	-1.58685	-1.56407
	H	4.38287	-1.19645	0.75196
	H	-4.42348	0.55860	-0.43966
	H	-0.37493	1.88109	-1.11006
	H	-1.37811	-2.28083	-1.49239
	H	-1.17399	-2.45139	1.93853

	H	0.87339	1.37232	2.02900
	H	0.95810	-3.44434	1.26146
	H	-3.52524	0.09297	3.01370
	H	-3.02109	-1.24249	1.97920
	H	-2.49417	-1.19095	3.67798
	H	-2.57871	1.98222	2.75707
	H	-1.00388	1.89397	3.58728
	H	-1.09955	2.19572	1.83542
	H	3.00183	1.36343	1.18141
	H	0.76630	-1.60393	-1.63041
	H	2.79274	-1.50204	-1.09446

Name	Cat 2O_conf3			
Cartesian Coordinates	N	2.91341	-0.56160	-1.46470
	N	0.61205	-0.60277	-1.61116
	N	3.16018	0.36725	1.28785
	N	2.04719	-1.69625	1.22978
	N	-1.54081	0.20845	2.38847
	C	4.15194	0.05977	-0.99206
	C	5.59881	0.56034	1.02391
	C	6.66521	0.16903	-1.24719
	C	6.82872	0.02775	0.27342
	C	5.37175	-0.49965	-1.73611
	C	4.31179	-0.12463	0.54187
	C	1.75638	0.18924	-1.50314
	C	-0.70644	-0.23130	-1.34019
	C	-3.39591	0.33955	-0.70076
	C	-1.66773	-1.25103	-1.29929
	C	-1.10690	1.08981	-1.07998
	C	-2.43537	1.35014	-0.75524
	C	-2.99226	-0.96266	-0.98316
	C	-3.96000	-2.10502	-0.85905
	C	-2.86538	2.76512	-0.48048
	C	1.99547	-0.36808	1.45623
	C	-0.30261	-1.82076	1.83171
	C	0.82908	0.30038	1.86812
	C	0.90180	-2.37218	1.43581
	C	-0.36691	-0.41537	2.04580
	C	-2.69713	-0.58431	2.78613
	C	-1.54842	1.63673	2.66560
	O	1.72870	1.41693	-1.48333
	F	-3.78335	-3.02596	-1.83285
	F	-5.24517	-1.69982	-0.90447
	F	-3.79560	-2.76559	0.32185
	F	-3.77315	2.81869	0.52740
	F	-1.82795	3.55805	-0.12481
	F	-3.44960	3.33696	-1.55778
	H	4.05859	1.12910	-1.20726
	H	5.51033	1.64547	0.86055
	H	5.70164	0.40839	2.10411
	H	6.63849	1.23622	-1.51109
	H	7.53008	-0.26161	-1.76600
	H	6.96964	-1.03339	0.52492
	H	7.73022	0.55335	0.61063
	H	5.23612	-0.35315	-2.81366
	H	5.43001	-1.58518	-1.56549
	H	4.38273	-1.19705	0.75087
	H	-4.42340	0.55924	-0.43875
	H	-1.37806	-2.27914	-1.49429
	H	-0.37500	1.88248	-1.10848
	H	-1.17370	-2.45415	1.93569

	H	0.87292	1.36984	2.03029
	H	0.95870	-3.44604	1.25808
	H	-2.49314	-1.19542	3.67733
	H	-3.52524	0.08842	3.01448
	H	-3.02124	-1.24623	1.97887
	H	-1.09868	2.19294	1.83831
	H	-2.58002	1.97848	2.75605
	H	-1.00722	1.88926	3.59010
	H	3.00111	1.36220	1.18178
	H	0.76632	-1.60216	-1.63103
	H	2.79306	-1.50100	-1.09690

Name	Cat 2O_conf4			
Cartesian Coordinates	N	2.90867	-0.28454	-1.52675
	N	0.60082	-0.30901	-1.64807
	N	3.14598	0.12203	1.34257
	N	2.11842	-1.94097	0.91702
	N	-1.57323	-0.38592	2.25993
	C	4.14317	0.25452	-0.95179
	C	5.58248	0.42374	1.12139
	C	6.65200	0.44939	-1.18237
	C	6.82090	0.05156	0.29159
	C	5.36944	-0.14768	-1.78153
	C	4.31090	-0.19637	0.52528
	C	1.74784	0.45488	-1.42657
	C	-0.71579	0.02929	-1.32424
	C	-3.39634	0.52186	-0.58490
	C	-1.10879	1.30718	-0.89287
	C	-1.68054	-0.98512	-1.39793
	C	-3.00083	-0.73436	-1.03421
	C	-2.43175	1.52759	-0.51966
	C	-2.83971	2.89275	-0.03748
	C	-3.98174	-1.86967	-1.11501
	C	2.00895	-0.67391	1.36462
	C	-0.23428	-2.25212	1.42832
	C	0.80946	-0.12936	1.85634
	C	0.99934	-2.68496	0.97855
	C	-0.36173	-0.90531	1.86979
	C	-1.64000	0.96432	2.79967
	C	-2.69556	-1.27963	2.51665
	O	1.72041	1.66268	-1.20324
	F	-3.78713	2.81260	0.93049
	F	-3.35978	3.65105	-1.02903
	F	-1.79730	3.58190	0.48260
	F	-3.51691	-2.96647	-0.45452
	F	-5.17849	-1.55960	-0.57629
	F	-4.19922	-2.26057	-2.39111
	H	4.03752	1.34385	-0.97401
	H	5.47144	1.51873	1.14610
	H	5.69115	0.09043	2.15949
	H	6.60575	1.54543	-1.25831
	H	7.52348	0.13009	-1.76647
	H	6.98143	-1.03434	0.35696
	H	7.71343	0.52756	0.71530
	H	5.22932	0.18145	-2.81740
	H	5.44705	-1.24512	-1.80127
	H	4.40947	-1.28640	0.54029
	H	-4.41720	0.70590	-0.27561
	H	-0.37209	2.09244	-0.81936
	H	-1.39321	-1.98626	-1.70499
	H	-1.07718	-2.92995	1.40861

	H	0.80631	0.89926	2.19403
	H	1.10415	-3.71134	0.62712
	H	-1.14795	1.05138	3.78036
	H	-1.17442	1.67713	2.11365
	H	-2.68583	1.25739	2.90215
	H	-3.57520	-0.68133	2.75965
	H	-2.93709	-1.87325	1.63188
	H	-2.49914	-1.96611	3.35345
	H	2.94728	1.11413	1.39913
	H	0.74889	-1.29821	-1.79879
	H	2.79983	-1.27715	-1.34014

Name	Cat 2O_conf5			
Cartesian Coordinates	N	2.90856	-0.28443	-1.52679
	N	0.60068	-0.30882	-1.64833
	N	3.14596	0.12161	1.34292
	N	2.11861	-1.94133	0.91677
	N	-1.57343	-0.38708	2.25944
	C	4.14301	0.25459	-0.95160
	C	5.58239	0.42344	1.12154
	C	6.65186	0.44968	-1.18223
	C	6.82081	0.05152	0.29164
	C	5.36931	-0.14733	-1.78146
	C	4.31079	-0.19661	0.52535
	C	1.74774	0.45502	-1.42684
	C	-0.71590	0.02962	-1.32440
	C	-3.39625	0.52234	-0.58450
	C	-1.10870	1.30747	-0.89284
	C	-1.68077	-0.98472	-1.39806
	C	-3.00096	-0.73386	-1.03411
	C	-2.43157	1.52794	-0.51926
	C	-2.83897	2.89309	-0.03653
	C	-3.98218	-1.86888	-1.11529
	C	2.00892	-0.67437	1.36462
	C	-0.23410	-2.25292	1.42768
	C	0.80927	-0.13007	1.85622
	C	0.99964	-2.68549	0.97797
	C	-0.36181	-0.90622	1.86937
	C	-1.64043	0.96285	2.79995
	C	-2.69587	-1.28093	2.51520
	O	1.72037	1.66286	-1.20372
	F	-3.78753	2.81303	0.93026
	F	-3.35708	3.65271	-1.02806
	F	-1.79645	3.58087	0.48537
	F	-5.17810	-1.55942	-0.57437
	F	-4.20161	-2.25767	-2.39172
	F	-3.51663	-2.96681	-0.45722
	H	4.03728	1.34392	-0.97360
	H	5.47129	1.51842	1.14647
	H	5.69109	0.08990	2.15956
	H	6.60557	1.54574	-1.25792
	H	7.52334	0.13053	-1.76641
	H	6.98141	-1.03439	0.35674
	H	7.71331	0.52747	0.71544
	H	5.22910	0.18202	-2.81725
	H	5.44699	-1.24476	-1.80144
	H	4.40941	-1.28663	0.54019
	H	-4.41703	0.70639	-0.27497
	H	-0.37190	2.09263	-0.81927
	H	-1.39354	-1.98586	-1.70517
	H	-1.07686	-2.93092	1.40783

	H	0.80585	0.89855	2.19395
	H	1.10466	-3.71180	0.62637
	H	-1.17516	1.67619	2.11428
	H	-2.68633	1.25557	2.90280
	H	-1.14826	1.04943	3.78060
	H	-3.57583	-0.68275	2.75729
	H	-2.93645	-1.87463	1.63023
	H	-2.50016	-1.96736	3.35222
	H	2.94732	1.11370	1.39984
	H	0.74870	-1.29809	-1.79869
	H	2.79968	-1.27704	-1.34022

Name	Cat 2O_conf6			
Cartesian Coordinates	N	2.90849	-0.28430	-1.52665
	N	0.60060	-0.30854	-1.64861
	N	3.14601	0.12143	1.34288
	N	2.11886	-1.94165	0.91668
	N	-1.57335	-0.38746	2.25906
	C	4.14296	0.25475	-0.95159
	C	5.58246	0.42347	1.12150
	C	6.65177	0.45004	-1.18233
	C	6.82084	0.05169	0.29147
	C	5.36922	-0.14698	-1.78158
	C	4.31088	-0.19658	0.52531
	C	1.74769	0.45513	-1.42663
	C	-0.71595	0.02978	-1.32444
	C	-3.39628	0.52247	-0.58434
	C	-1.10872	1.30759	-0.89262
	C	-1.68085	-0.98449	-1.39822
	C	-3.00103	-0.73366	-1.03414
	C	-2.43155	1.52804	-0.51899
	C	-2.83903	2.89313	-0.03615
	C	-3.98229	-1.86864	-1.11539
	C	2.00906	-0.67468	1.36449
	C	-0.23388	-2.25332	1.42743
	C	0.80937	-0.13045	1.85602
	C	0.99992	-2.68585	0.97783
	C	-0.36168	-0.90664	1.86911
	C	-1.64018	0.96230	2.80003
	C	-2.69579	-1.28131	2.51482
	O	1.72034	1.66289	-1.20304
	F	-3.35783	3.65248	-1.02755
	F	-1.79649	3.58123	0.48517
	F	-3.78715	2.81293	0.93107
	F	-5.17803	-1.55936	-0.57400
	F	-4.20210	-2.25705	-2.39186
	F	-3.51654	-2.96681	-0.45780
	H	4.03712	1.34407	-0.97347
	H	5.47131	1.51845	1.14659
	H	5.69124	0.08981	2.15948
	H	6.60537	1.54610	-1.25786
	H	7.52323	0.13106	-1.76663
	H	6.98146	-1.03422	0.35642
	H	7.71336	0.52761	0.71528
	H	5.22895	0.18251	-2.81731
	H	5.44696	-1.24440	-1.80172
	H	4.40957	-1.28659	0.54000
	H	-4.41702	0.70651	-0.27469
	H	-0.37189	2.09272	-0.81897
	H	-1.39369	-1.98560	-1.70554
	H	-1.07659	-2.93138	1.40754

	H	0.80589	0.89817	2.19373
	H	1.10498	-3.71216	0.62626
	H	-1.14765	1.04856	3.78055
	H	-1.17512	1.67588	2.11445
	H	-2.68603	1.25497	2.90336
	H	-3.57591	-0.68310	2.75631
	H	-2.93599	-1.87539	1.63001
	H	-2.50033	-1.96737	3.35220
	H	2.94710	1.11349	1.39953
	H	0.74859	-1.29776	-1.79938
	H	2.79967	-1.27696	-1.34031

Name	Cat 2O_conf7			
Cartesian Coordinates	N	2.90842	-0.28240	-1.52698
	N	0.60049	-0.30744	-1.64783
	N	3.14667	0.12014	1.34307
	N	2.11971	-1.94292	0.91608
	N	-1.57286	-0.38934	2.25827
	C	4.14292	0.25608	-0.95160
	C	5.58302	0.42237	1.12129
	C	6.65170	0.45182	-1.18276
	C	6.82123	0.05173	0.29050
	C	5.36904	-0.14460	-1.78237
	C	4.31128	-0.19699	0.52472
	C	1.74742	0.45640	-1.42570
	C	-0.71617	0.03070	-1.32385
	C	-3.39666	0.52289	-0.58397
	C	-1.10909	1.30826	-0.89148
	C	-1.68101	-0.98361	-1.39826
	C	-3.00125	-0.73303	-1.03428
	C	-2.43200	1.52846	-0.51797
	C	-2.83969	2.89322	-0.03441
	C	-3.98262	-1.86786	-1.11646
	C	2.00976	-0.67615	1.36426
	C	-0.23308	-2.25493	1.42633
	C	0.80996	-0.13215	1.85585
	C	1.00083	-2.68722	0.97677
	C	-0.36104	-0.90842	1.86848
	C	-1.63963	0.95998	2.80039
	C	-2.69538	-1.28330	2.51343
	O	1.71984	1.66394	-1.20084
	F	-1.79713	3.58156	0.48652
	F	-3.78720	2.81220	0.93337
	F	-3.35947	3.65268	-1.02521
	F	-4.20404	-2.25379	-2.39338
	F	-3.51605	-2.96723	-0.46152
	F	-5.17772	-1.55953	-0.57303
	H	4.03699	1.34540	-0.97223
	H	5.47181	1.51732	1.14773
	H	5.69207	0.08749	2.15884
	H	6.60520	1.54796	-1.25701
	H	7.52300	0.13358	-1.76771
	H	6.98203	-1.03423	0.35412
	H	7.71381	0.52727	0.71461
	H	5.22851	0.18602	-2.81771
	H	5.44690	-1.24199	-1.80373
	H	4.40995	-1.28702	0.53807
	H	-4.41746	0.70673	-0.27442
	H	-0.37231	2.09338	-0.81732
	H	-1.39374	-1.98457	-1.70593
	H	-1.07570	-2.93308	1.40622

	H	0.80629	0.89643	2.19365
	H	1.10602	-3.71341	0.62487
	H	-2.68548	1.25243	2.90430
	H	-1.14675	1.04543	3.78079
	H	-1.17490	1.67419	2.11523
	H	-2.50031	-1.96940	3.35086
	H	-3.57567	-0.68515	2.75450
	H	-2.93508	-1.87725	1.62840
	H	2.94739	1.11211	1.40015
	H	0.74858	-1.29665	-1.79865
	H	2.80033	-1.27561	-1.34338

Name	Cat 2O_conf8			
Cartesian Coordinates	N	2.85723	0.80077	-1.31770
	N	0.54134	0.60045	-1.33987
	N	3.07605	-0.67430	1.12659
	N	2.42745	-2.12348	-0.58516
	N	-1.29980	-2.74359	1.27962
	C	4.04459	0.98552	-0.47840
	C	5.44660	-0.00794	1.37294
	C	6.49928	1.53409	-0.35931
	C	6.72168	0.34552	0.58969
	C	5.30274	1.29815	-1.29599
	C	4.27780	-0.26458	0.41215
	C	1.65551	1.28178	-0.83957
	C	-0.78176	0.78773	-0.92072
	C	-3.47849	1.03723	-0.12808
	C	-1.24569	1.98295	-0.34819
	C	-1.68428	-0.27339	-1.10448
	C	-3.01160	-0.14078	-0.71190
	C	-2.57976	2.08657	0.04498
	C	-3.07746	3.39190	0.60693
	C	-3.96495	-1.29339	-0.84711
	C	2.11737	-1.50470	0.57551
	C	0.29724	-3.27549	-0.47557
	C	0.88694	-1.68154	1.23446
	C	1.51963	-2.99709	-1.05692
	C	-0.06594	-2.57092	0.70789
	C	-1.72773	-1.88015	2.36896
	C	-2.10154	-3.91411	0.93753
	O	1.56507	2.23615	-0.07136
	F	-2.10360	4.07782	1.24272
	F	-4.08615	3.20445	1.49031
	F	-3.55806	4.20051	-0.36807
	F	-3.47919	-2.28274	-1.63259
	F	-4.24529	-1.85018	0.36197
	F	-5.14943	-0.90706	-1.36965
	H	3.82431	1.84070	0.16809
	H	5.18516	0.81983	2.04940
	H	5.60522	-0.89502	1.99652
	H	6.31217	2.44042	0.23452
	H	7.40549	1.72478	-0.94665
	H	7.03193	-0.53146	0.00353
	H	7.54057	0.56390	1.28565
	H	5.11904	2.17584	-1.92569
	H	5.51749	0.45611	-1.97041
	H	4.54121	-1.10155	-0.24168
	H	-4.51179	1.13186	0.18430
	H	-0.56284	2.80411	-0.19295
	H	-1.33830	-1.20988	-1.52825
	H	-0.37141	-3.98650	-0.94315

	H	0.70031	-1.13851	2.15321
	H	1.79649	-3.49975	-1.98300
	H	-1.20984	-2.10022	3.31452
	H	-1.54818	-0.83088	2.10893
	H	-2.80088	-2.00144	2.51871
	H	-2.96193	-3.96251	1.60517
	H	-2.48109	-3.86185	-0.08794
	H	-1.52242	-4.83911	1.05258
	H	2.73907	-0.02252	1.82410
	H	0.72599	-0.30967	-1.74511
	H	2.82214	-0.12401	-1.73898

Name	Cat 2O_conf9			
Cartesian Coordinates	N	2.85946	0.77164	-1.33056
	N	0.54290	0.58268	-1.36358
	N	3.06578	-0.67173	1.13399
	N	2.41423	-2.14141	-0.55908
	N	-1.32366	-2.71079	1.30052
	C	4.04463	0.96313	-0.48968
	C	5.43912	-0.01436	1.37613
	C	6.50140	1.50375	-0.37100
	C	6.71708	0.32472	0.59125
	C	5.30598	1.26206	-1.30758
	C	4.27072	-0.27688	0.41640
	C	1.65786	1.26429	-0.86481
	C	-0.78034	0.78216	-0.95068
	C	-3.48220	1.06271	-0.18558
	C	-1.24290	1.99192	-0.40964
	C	-1.68677	-0.27888	-1.11687
	C	-3.01628	-0.13088	-0.73873
	C	-2.58001	2.11105	-0.02951
	C	-3.03561	3.39123	0.61892
	C	-3.97628	-1.27909	-0.86381
	C	2.10378	-1.50395	0.59138
	C	0.27734	-3.28011	-0.43941
	C	0.86992	-1.66351	1.24823
	C	1.50319	-3.01701	-1.02072
	C	-0.08631	-2.55517	0.73158
	C	-2.13240	-3.88038	0.97237
	C	-1.75234	-1.82522	2.37178
	O	1.56742	2.22822	-0.10874
	F	-2.39656	4.46992	0.11636
	F	-2.79821	3.38066	1.95339
	F	-4.36348	3.59381	0.45946
	F	-5.15440	-0.89325	-1.40079
	F	-3.49129	-2.28351	-1.63050
	F	-4.26881	-1.81578	0.35151
	H	3.82497	1.82680	0.14568
	H	5.18015	0.82217	2.04271
	H	5.59272	-0.89495	2.01005
	H	6.31663	2.41724	0.21251
	H	7.40953	1.68450	-0.95851
	H	7.02444	-0.55998	0.01523
	H	7.53578	0.54710	1.28616
	H	5.12690	2.13334	-1.94742
	H	5.51905	0.41180	-1.97214
	H	4.53100	-1.12312	-0.22665
	H	-4.52054	1.17403	0.10275
	H	-0.56113	2.81893	-0.28220
	H	-1.34312	-1.22468	-1.52135
	H	-0.39355	-3.99449	-0.89863

	H	0.68297	-1.10548	2.15790
	H	1.78027	-3.53494	-1.93830
	H	-2.99487	-3.91390	1.63831
	H	-2.50877	-3.83998	-0.05483
	H	-1.55979	-4.80748	1.10201
	H	-2.82763	-1.93489	2.51519
	H	-1.24356	-2.03349	3.32489
	H	-1.56195	-0.78205	2.09544
	H	2.73024	-0.00832	1.82120
	H	0.72484	-0.33430	-1.75433
	H	2.82229	-0.15837	-1.73995

Name	Cat 2O_conf10			
Cartesian Coordinates	N	-0.53855	3.38733	-0.33768
	N	0.68847	1.40869	-0.21850
	N	-3.35767	1.11520	0.56355
	N	-1.80894	-0.40821	-0.28545
	N	-4.82597	-3.34349	-0.58367
	C	-1.82798	2.72548	-0.51072
	C	-2.82300	3.18314	1.80076
	C	-3.32744	4.76861	-0.09598
	C	-3.87443	4.11544	1.18028
	C	-2.86431	3.69794	-1.09555
	C	-2.32002	2.10284	0.83209
	C	0.69671	2.79963	-0.22253
	C	1.81377	0.59293	-0.06225
	C	3.95574	-1.23723	0.15970
	C	3.12345	0.98977	-0.38467
	C	1.60032	-0.71513	0.39811
	C	2.65614	-1.61889	0.48326
	C	4.16903	0.07657	-0.26120
	C	5.57294	0.53529	-0.55204
	C	2.35802	-3.01096	0.96582
	C	-3.07922	-0.15247	0.09843
	C	-2.51385	-2.64308	-0.90813
	C	-4.11368	-1.10170	0.01754
	C	-1.57614	-1.64025	-0.78181
	C	-3.85026	-2.38552	-0.49025
	C	-6.18865	-3.03429	-0.17930
	C	-4.51608	-4.65122	-1.14554
	O	1.72162	3.47607	-0.12858
	F	6.17097	1.03918	0.55461
	F	5.60831	1.50568	-1.49182
	F	6.35492	-0.47991	-0.98922
	F	2.12376	-3.04300	2.29958
	F	1.24627	-3.51303	0.36997
	F	3.37043	-3.86965	0.71863
	H	-1.69841	1.91022	-1.22910
	H	-3.20600	2.70382	2.71069
	H	-1.95308	3.77906	2.10333
	H	-4.08945	5.40350	-0.56318
	H	-2.49318	5.43480	0.16922
	H	-4.16793	4.87920	1.91004
	H	-4.78671	3.55257	0.93461
	H	-3.73140	3.10029	-1.40141
	H	-2.45413	4.15503	-2.00393
	H	-1.47003	1.59866	1.30703
	H	4.77489	-1.94216	0.22301
	H	3.30830	1.99666	-0.72789
	H	0.60110	-1.02281	0.68347
	H	-2.21443	-3.59897	-1.31635

	H	-5.10636	-0.81786	0.34565
	H	-0.55174	-1.83447	-1.09112
	H	-6.61076	-2.21059	-0.77185
	H	-6.81717	-3.91352	-0.32521
	H	-6.23894	-2.75352	0.88131
	H	-5.41322	-5.27107	-1.12599
	H	-4.17586	-4.57683	-2.18723
	H	-3.73807	-5.16409	-0.56574
	H	-4.24390	1.23592	1.03188
	H	-0.20294	0.89987	-0.21901
	H	-0.49317	4.39621	-0.34314

Name	Cat 2O_conf11			
Cartesian Coordinates	N	-0.53808	3.38705	-0.33915
	N	0.68872	1.40828	-0.21937
	N	-3.35718	1.11559	0.56414
	N	-1.80898	-0.40812	-0.28530
	N	-4.82594	-3.34390	-0.57950
	C	-1.82769	2.72534	-0.51118
	C	-2.82185	3.18386	1.80052
	C	-3.32683	4.76874	-0.09659
	C	-3.87342	4.11604	1.18009
	C	-2.86413	3.69770	-1.09597
	C	-2.31931	2.10318	0.83203
	C	0.69702	2.79927	-0.22283
	C	1.81392	0.59250	-0.06272
	C	3.95585	-1.23772	0.15979
	C	3.12371	0.98931	-0.38472
	C	1.60036	-0.71558	0.39754
	C	2.65615	-1.61935	0.48295
	C	4.16925	0.07610	-0.26099
	C	5.57315	0.53510	-0.55141
	C	2.35754	-3.01149	0.96496
	C	-3.07899	-0.15227	0.09936
	C	-2.51433	-2.64310	-0.90707
	C	-4.11349	-1.10154	0.01943
	C	-1.57653	-1.64024	-0.78165
	C	-3.85033	-2.38559	-0.48788
	C	-6.18972	-3.03235	-0.18056
	C	-4.51793	-4.64961	-1.14714
	O	1.72189	3.47558	-0.12776
	F	5.60872	1.50361	-1.49315
	F	6.35626	-0.48042	-0.98579
	F	6.16977	1.04173	0.55475
	F	1.24825	-3.51465	0.36542
	F	3.37144	-3.86951	0.72170
	F	2.11850	-3.04316	2.29789
	H	-1.69847	1.90983	-1.22930
	H	-3.20456	2.70490	2.71076
	H	-1.95178	3.77981	2.10260
	H	-4.08895	5.40354	-0.56374
	H	-2.49244	5.43498	0.16808
	H	-4.16661	4.88006	1.90969
	H	-4.78582	3.55314	0.93491
	H	-3.73139	3.10002	-1.40131
	H	-2.45422	4.15446	-2.00463
	H	-1.46920	1.59906	1.30682
	H	4.77498	-1.94264	0.22338
	H	3.30871	1.99621	-0.72787
	H	0.60109	-1.02330	0.68267
	H	-2.21530	-3.59890	-1.31578

	H	-5.10597	-0.81759	0.34802
	H	-0.55239	-1.83441	-1.09185
	H	-6.61003	-2.21081	-0.77749
	H	-6.81811	-3.91203	-0.32434
	H	-6.24262	-2.74741	0.87873
	H	-3.73577	-5.16270	-0.57334
	H	-5.41401	-5.27085	-1.12314
	H	-4.18420	-4.57236	-2.19081
	H	-4.24306	1.23637	1.03313
	H	-0.20275	0.89956	-0.21978
	H	-0.49287	4.39594	-0.34231

Name	Cat 2O_conf12			
Cartesian Coordinates	N	-2.28978	-0.50418	1.05012
	N	-0.40130	-0.38370	2.46365
	N	-2.69521	1.84688	-0.60716
	N	-0.91725	0.55103	-1.37763
	N	1.75736	3.74228	-0.71424
	C	-3.64398	-0.07140	0.69551
	C	-5.04071	1.20646	-0.97473
	C	-6.03264	-0.78283	0.25213
	C	-6.03196	0.03441	-1.04789
	C	-4.61684	-1.25746	0.60765
	C	-3.62335	0.72282	-0.63270
	C	-1.77593	-0.16933	2.27043
	C	0.57979	-0.87324	1.59287
	C	2.61823	-1.82606	-0.10285
	C	0.35641	-1.98589	0.76985
	C	1.84031	-0.25477	1.57310
	C	2.84196	-0.73545	0.73813
	C	1.36665	-2.43540	-0.07919
	C	1.06761	-3.53722	-1.05854
	C	4.18272	-0.05774	0.68078
	C	-1.34383	1.72854	-0.87578
	C	1.31939	1.47253	-1.49371
	C	-0.48740	2.81712	-0.63463
	C	0.39001	0.46868	-1.68111
	C	0.88189	2.70789	-0.93877
	C	3.18694	3.54668	-0.92192
	C	1.29670	4.94283	-0.03455
	O	-2.43302	0.29525	3.20038
	F	0.61284	-3.02861	-2.23324
	F	0.11868	-4.38317	-0.60455
	F	2.16544	-4.26837	-1.34946
	F	5.17836	-0.87082	1.09545
	F	4.48981	0.31139	-0.59017
	F	4.23244	1.06170	1.43968
	H	-3.98152	0.59961	1.49342
	H	-5.37321	1.91850	-0.20354
	H	-5.01052	1.75366	-1.92372
	H	-6.42059	-0.16065	1.07175
	H	-6.70836	-1.64195	0.16260
	H	-5.75054	-0.61928	-1.88596
	H	-7.03892	0.41045	-1.26591
	H	-4.60989	-1.79322	1.56374
	H	-4.25306	-1.95906	-0.15693
	H	-3.27910	0.05622	-1.42975
	H	3.39552	-2.18248	-0.76877
	H	-0.59874	-2.49593	0.79242
	H	2.02105	0.61843	2.18999
	H	2.35871	1.28317	-1.72372

	H	-0.90355	3.72715	-0.21976
	H	0.71030	-0.49154	-2.07768
	H	3.39553	3.23568	-1.95222
	H	3.70424	4.49179	-0.74984
	H	3.60703	2.79316	-0.24432
	H	0.92551	4.72879	0.97884
	H	2.12276	5.65060	0.04644
	H	0.49012	5.43104	-0.59602
	H	-2.95796	2.61679	-0.00541
	H	-0.07860	0.09420	3.29476
	H	-1.64877	-0.55955	0.25958

Name	Cat 2O_conf13			
Cartesian Coordinates	N	-2.75359	-0.40270	-1.68285
	N	-0.45200	-0.25376	-1.71240
	N	-3.63071	-1.57613	0.84221
	N	-1.83778	-2.98346	0.39088
	N	0.84957	-0.23119	2.13150
	C	-4.09273	-0.12491	-1.14084
	C	-5.58411	-0.07389	0.90421
	C	-6.09918	1.40498	-1.09020
	C	-6.16249	1.27012	0.43838
	C	-4.66336	1.22418	-1.60413
	C	-4.14718	-0.27536	0.40413
	C	-1.65498	0.35447	-1.34424
	C	0.83255	0.11860	-1.30467
	C	3.46239	0.68804	-0.45922
	C	1.83637	-0.85689	-1.37413
	C	1.15899	1.39860	-0.82644
	C	2.46052	1.65731	-0.40404
	C	3.13114	-0.57073	-0.95246
	C	4.13470	-1.68746	-0.91705
	C	2.80050	2.98661	0.21028
	C	-2.25855	-1.82385	0.93345
	C	0.40917	-2.42569	1.12546
	C	-1.40733	-0.91946	1.58113
	C	-0.52728	-3.25629	0.53458
	C	-0.02367	-1.17158	1.63791
	C	2.19231	-0.59389	2.56617
	C	0.33247	1.04857	2.59611
	O	-1.71489	1.46059	-0.81329
	F	4.02052	-2.51029	-1.98236
	F	3.95678	-2.46731	0.18960
	F	5.40493	-1.23891	-0.87551
	F	2.96548	2.86922	1.55976
	F	3.96494	3.47922	-0.26830
	F	1.84759	3.91697	0.01052
	H	-4.72692	-0.91752	-1.56269
	H	-6.21089	-0.89489	0.52293
	H	-5.59339	-0.15290	1.99703
	H	-6.75223	0.64700	-1.54793
	H	-6.48488	2.38250	-1.40362
	H	-5.58812	2.08826	0.89636
	H	-7.19610	1.37487	0.78977
	H	-4.62866	1.26882	-2.69914
	H	-4.02407	2.02729	-1.22663
	H	-3.51227	0.50408	0.83244
	H	4.46871	0.90604	-0.12245
	H	1.59884	-1.85528	-1.72651
	H	0.39539	2.15857	-0.76465
	H	1.44906	-2.72385	1.13877

	H	-1.82922	-0.02815	2.02129
	H	-0.20118	-4.21033	0.12080
	H	2.19343	-1.09377	3.54685
	H	2.68092	-1.24436	1.84141
	H	2.79329	0.31551	2.63512
	H	1.16621	1.73778	2.73164
	H	-0.33242	1.47784	1.84038
	H	-0.21787	0.96673	3.54618
	H	-4.08559	-2.35428	0.37128
	H	-0.51776	-1.21170	-2.03167
	H	-2.57700	-1.39129	-1.81584

Name	Cat 2O_conf14			
Cartesian Coordinates	N	-2.75377	-0.40176	-1.68315
	N	-0.45212	-0.25351	-1.71257
	N	-3.63035	-1.57684	0.84122
	N	-1.83701	-2.98351	0.38950
	N	0.84940	-0.23113	2.13139
	C	-4.09283	-0.12437	-1.14085
	C	-5.58389	-0.07490	0.90452
	C	-6.09939	1.40531	-1.08880
	C	-6.16246	1.26937	0.43969
	C	-4.66364	1.22500	-1.60311
	C	-4.14704	-0.27589	0.40407
	C	-1.65507	0.35504	-1.34448
	C	0.83246	0.11869	-1.30472
	C	3.46230	0.68790	-0.45908
	C	1.15904	1.39872	-0.82661
	C	1.83618	-0.85689	-1.37412
	C	3.13094	-0.57084	-0.95228
	C	2.46056	1.65736	-0.40420
	C	2.80070	2.98672	0.20988
	C	4.13433	-1.68772	-0.91642
	C	-2.25814	-1.82427	0.93257
	C	0.40972	-2.42547	1.12456
	C	-1.40723	-0.91997	1.58077
	C	-0.52646	-3.25608	0.53327
	C	-0.02349	-1.17169	1.63748
	C	2.19204	-0.59339	2.56665
	C	0.33182	1.04837	2.59615
	O	-1.71477	1.46111	-0.81338
	F	1.84836	3.91744	0.00927
	F	3.96568	3.47870	-0.26808
	F	2.96475	2.86968	1.55953
	F	4.01924	-2.51174	-1.98072
	F	5.40463	-1.23934	-0.87623
	F	3.95707	-2.46637	0.19124
	H	-4.72706	-0.91672	-1.56313
	H	-6.21065	-0.89572	0.52281
	H	-5.59298	-0.15463	1.99729
	H	-6.75246	0.64763	-1.54698
	H	-6.48522	2.38304	-1.40144
	H	-5.58809	2.08724	0.89815
	H	-7.19603	1.37381	0.79129
	H	-4.62914	1.27043	-2.69809
	H	-4.02434	2.02790	-1.22517
	H	-3.51216	0.50335	0.83278
	H	4.46865	0.90580	-0.12235
	H	0.39552	2.15876	-0.76489
	H	1.59870	-1.85519	-1.72678
	H	1.44969	-2.72338	1.13780

	H	-1.82942	-0.02907	2.02147
	H	-0.20007	-4.20988	0.11913
	H	2.68068	-1.24475	1.84276
	H	2.79310	0.31605	2.63458
	H	2.19303	-1.09201	3.54798
	H	-0.33320	1.47749	1.84044
	H	-0.21847	0.96625	3.54622
	H	1.16533	1.73787	2.73165
	H	-4.08504	-2.35489	0.36995
	H	-0.51811	-1.21201	-2.03013
	H	-2.57742	-1.39013	-1.81782

Name	Cat 2O_conf15			
Cartesian Coordinates	N	-0.54751	3.39835	-0.31069
	N	0.68465	1.42563	-0.17421
	N	-3.36600	1.10686	0.53951
	N	-1.78608	-0.41561	-0.25233
	N	-4.78174	-3.36464	-0.62288
	C	-1.82967	2.72818	-0.50705
	C	-2.87034	3.18009	1.78598
	C	-3.34816	4.76247	-0.11961
	C	-3.91551	4.10614	1.14602
	C	-2.86150	3.69428	-1.11047
	C	-2.34208	2.10321	0.82701
	C	0.69010	2.81593	-0.19173
	C	1.81179	0.61251	-0.02173
	C	3.95185	-1.22153	0.18321
	C	1.60458	-0.68572	0.46628
	C	3.11652	1.00175	-0.37301
	C	4.16162	0.08748	-0.25607
	C	2.65860	-1.59285	0.53990
	C	2.34120	-2.99646	0.97173
	C	5.56243	0.53844	-0.57249
	C	-3.06846	-0.16245	0.09112
	C	-2.46148	-2.66060	-0.87136
	C	-4.09608	-1.11699	-0.01268
	C	-1.53224	-1.65252	-0.72631
	C	-3.81224	-2.40355	-0.50207
	C	-4.44051	-4.68922	-1.12380
	C	-6.15127	-3.06818	-0.23259
	O	1.71357	3.49517	-0.10325
	F	1.64158	-3.01906	2.13072
	F	3.44660	-3.74961	1.14953
	F	1.56913	-3.62887	0.04878
	F	5.58639	1.50688	-1.51451
	F	6.18267	1.04072	0.52263
	F	6.33117	-0.48181	-1.02150
	H	-1.68298	1.91295	-1.22253
	H	-3.26733	2.69802	2.68840
	H	-2.00986	3.78143	2.10432
	H	-4.10449	5.39371	-0.60079
	H	-2.52202	5.43233	0.16110
	H	-4.22735	4.86805	1.87009
	H	-4.81975	3.53807	0.88303
	H	-3.71965	3.09156	-1.43137
	H	-2.43843	4.15345	-2.01192
	H	-1.49781	1.60560	1.31845
	H	4.77029	-1.92752	0.24295
	H	0.61244	-0.98289	0.78633
	H	3.29766	2.00395	-0.73193
	H	-2.14447	-3.62056	-1.25610

	H	-5.09912	-0.83520	0.28421
	H	-0.49723	-1.84821	-0.99784
	H	-3.70294	-5.18817	-0.48145
	H	-5.33969	-5.30594	-1.14774
	H	-4.03385	-4.64339	-2.14256
	H	-6.22112	-2.80560	0.83207
	H	-6.56515	-2.23587	-0.81803
	H	-6.77489	-3.94586	-0.40628
	H	-4.26722	1.22804	0.97801
	H	-0.20454	0.91287	-0.17384
	H	-0.50487	4.40698	-0.33887

Name	Cat 2O_conf16			
Cartesian Coordinates	N	-0.54795	3.38756	-0.33009
	N	0.68823	1.41489	-0.20251
	N	-3.36029	1.10408	0.56425
	N	-1.80624	-0.41163	-0.28868
	N	-4.81465	-3.35446	-0.60109
	C	-1.83433	2.72052	-0.50583
	C	-2.83417	3.17210	1.80472
	C	-3.34169	4.75781	-0.09105
	C	-3.88802	4.10139	1.18383
	C	-2.87342	3.68981	-1.09111
	C	-2.32620	2.09461	0.83554
	C	0.68953	2.80582	-0.20861
	C	1.81547	0.60315	-0.03892
	C	3.96521	-1.21425	0.21631
	C	1.60164	-0.70986	0.40851
	C	3.12843	1.01019	-0.33239
	C	4.17745	0.10269	-0.19360
	C	2.66159	-1.60636	0.51175
	C	2.37010	-2.99796	0.99972
	C	5.56488	0.53718	-0.58378
	C	-3.07770	-0.16099	0.09478
	C	-2.50458	-2.64555	-0.92226
	C	-4.10944	-1.11274	0.00883
	C	-1.56978	-1.64068	-0.79060
	C	-3.84189	-2.39378	-0.50374
	C	-4.50296	-4.65524	-1.17794
	C	-6.18022	-3.04805	-0.20426
	O	1.71035	3.48795	-0.11077
	F	2.17873	-3.03373	2.34034
	F	3.37078	-3.86054	0.71872
	F	1.23845	-3.49280	0.43733
	F	5.77535	1.84860	-0.33838
	F	6.51837	-0.15880	0.07828
	F	5.79092	0.34525	-1.90726
	H	-1.70048	1.90658	-1.22491
	H	-3.21675	2.69047	2.71361
	H	-1.96671	3.77068	2.10906
	H	-4.10534	5.39045	-0.55862
	H	-2.51014	5.42666	0.17592
	H	-4.18513	4.86338	1.91398
	H	-4.79802	3.53561	0.93637
	H	-3.73791	3.08936	-1.39879
	H	-2.46366	4.14931	-1.99847
	H	-1.47536	1.59258	1.31121
	H	4.78972	-1.91017	0.30558
	H	0.59991	-1.02469	0.67617
	H	3.31570	2.02474	-0.65133
	H	-2.20236	-3.59847	-1.33533

	H	-5.10336	-0.83277	0.33646
	H	-0.54468	-1.83029	-1.10056
	H	-4.16925	-4.56979	-2.22099
	H	-3.71950	-5.17011	-0.60756
	H	-5.39728	-5.27918	-1.15838
	H	-6.60220	-2.22656	-0.80017
	H	-6.80549	-3.92949	-0.35082
	H	-6.23582	-2.76554	0.85549
	H	-4.24747	1.22094	1.03174
	H	-0.20092	0.90234	-0.21107
	H	-0.50676	4.39661	-0.33636

Name	Cat 2O_conf17			
Cartesian Coordinates	N	-3.24942	1.89978	-0.96556
	N	-1.09234	1.08195	-1.34728
	N	-2.47037	0.23118	1.33610
	N	-1.77007	-1.58258	0.04066
	N	1.76425	-1.87041	2.33077
	C	-3.92379	0.63411	-0.66952
	C	-4.65474	-1.00829	1.14994
	C	-6.17278	-0.54593	-0.81737
	C	-6.10943	-0.88131	0.67875
	C	-5.38730	0.73479	-1.12779
	C	-3.84623	0.26710	0.83872
	C	-1.89112	2.14869	-0.95521
	C	0.28774	0.96570	-1.14047
	C	3.03058	0.48326	-0.67502
	C	1.05416	1.88465	-0.40787
	C	0.91889	-0.17161	-1.66913
	C	2.26704	-0.40886	-1.42739
	C	2.40603	1.62739	-0.18226
	C	3.17174	2.57960	0.69376
	C	2.92182	-1.61842	-2.03333
	C	-1.54012	-0.76057	1.08610
	C	0.33095	-2.68998	0.53372
	C	-0.37759	-0.82113	1.87315
	C	-0.83568	-2.52434	-0.18595
	C	0.60320	-1.79252	1.60281
	C	2.80508	-2.81214	1.93728
	C	2.10221	-0.80061	3.26071
	O	-1.44716	3.26876	-0.70731
	F	2.89696	2.36947	2.01172
	F	2.85815	3.86809	0.44190
	F	4.50873	2.44798	0.55165
	F	2.06088	-2.65317	-2.17103
	F	3.42292	-1.36033	-3.26366
	F	3.96035	-2.05951	-1.27774
	H	-3.44727	-0.17011	-1.23597
	H	-4.17263	-1.85564	0.65241
	H	-4.60882	-1.19744	2.22943
	H	-5.75004	-1.38008	-1.39525
	H	-7.21339	-0.43040	-1.14346
	H	-6.61527	-0.08745	1.24847
	H	-6.65218	-1.81156	0.88590
	H	-5.40883	0.96248	-2.19946
	H	-5.86341	1.58379	-0.61237
	H	-4.31376	1.10386	1.37609
	H	4.07891	0.29346	-0.48281
	H	0.58951	2.77887	-0.01867
	H	0.34318	-0.88841	-2.24317
	H	1.03680	-3.45001	0.22959

	H	-0.24582	-0.09736	2.66811
	H	-1.03473	-3.18186	-1.03118
	H	3.22273	-2.58469	0.94812
	H	3.61285	-2.77519	2.66990
	H	2.41804	-3.83732	1.92256
	H	2.18870	0.17200	2.75865
	H	1.34982	-0.71642	4.05452
	H	3.05789	-1.02799	3.73537
	H	-2.30359	0.76601	2.17532
	H	-1.55418	0.18705	-1.46511
	H	-3.74736	2.72462	-0.64883

Name	Cat 2O_conf18			
Cartesian Coordinates	N	2.41599	-2.76693	0.29595
	N	0.48081	-1.62783	-0.39056
	N	2.67159	0.05751	1.21320
	N	2.21816	0.93566	-0.89530
	N	0.12687	4.16290	0.83079
	C	3.43153	-1.86057	-0.22967
	C	5.00997	0.09074	0.28891
	C	5.81906	-1.83078	-1.13156
	C	6.21506	-0.78034	-0.08593
	C	4.66296	-2.69742	-0.61966
	C	3.82181	-0.75547	0.79077
	C	1.05089	-2.64689	0.37018
	C	-0.87187	-1.26542	-0.31544
	C	-3.56883	-0.42541	-0.20083
	C	-1.89812	-2.19334	-0.06625
	C	-1.21583	0.07786	-0.52632
	C	-2.54728	0.48428	-0.46379
	C	-3.22215	-1.76300	-0.00911
	C	-4.30600	-2.78729	0.19406
	C	-2.85188	1.94867	-0.60202
	C	2.11542	1.06724	0.44510
	C	0.99951	3.01487	-1.13712
	C	1.41741	2.11880	1.06081
	C	1.67936	1.92207	-1.63642
	C	0.82608	3.12534	0.27198
	C	-0.54675	5.13018	-0.02919
	C	-0.26613	4.08120	2.23180
	O	0.38727	-3.44147	1.03694
	F	-3.90782	-3.79557	1.00003
	F	-4.68296	-3.35149	-0.98015
	F	-5.41930	-2.24566	0.74106
	F	-2.16639	2.51636	-1.62539
	F	-4.16268	2.19001	-0.81238
	F	-2.49957	2.64011	0.51675
	H	3.04651	-1.37447	-1.12864
	H	4.68546	0.66461	-0.58531
	H	5.27809	0.81397	1.06918
	H	5.51320	-1.32542	-2.05848
	H	6.67544	-2.46723	-1.38478
	H	6.59833	-1.28979	0.81076
	H	7.02947	-0.14957	-0.46238
	H	4.36098	-3.43743	-1.36927
	H	5.00181	-3.26050	0.26447
	H	4.13785	-1.28361	1.70040
	H	-4.60086	-0.10365	-0.14384
	H	-1.65241	-3.23078	0.10357
	H	-0.44564	0.81156	-0.72378
	H	0.57223	3.73354	-1.82247

	H	1.34779	2.14071	2.14143
	H	1.78286	1.80326	-2.71356
	H	-0.99207	5.90726	0.59375
	H	0.16942	5.61464	-0.70183
	H	-1.33831	4.66603	-0.62940
	H	-0.87192	4.95185	2.48549
	H	-0.85748	3.17743	2.43092
	H	0.61050	4.08045	2.89081
	H	2.63476	0.23130	2.20784
	H	1.09915	-0.89357	-0.72952
	H	2.74581	-3.49341	0.91935

Name	Cat 2O_conf19			
Cartesian Coordinates	N	2.41628	-2.76666	0.29602
	N	0.48102	-1.62769	-0.39087
	N	2.67185	0.05770	1.21324
	N	2.21823	0.93603	-0.89511
	N	0.12672	4.16287	0.83158
	C	3.43175	-1.86031	-0.22971
	C	5.01012	0.09105	0.28885
	C	5.81918	-1.83032	-1.13187
	C	6.21523	-0.77993	-0.08618
	C	4.66320	-2.69706	-0.61989
	C	3.82203	-0.75525	0.79072
	C	1.05113	-2.64652	0.37008
	C	-0.87169	-1.26535	-0.31561
	C	-3.56873	-0.42565	-0.20090
	C	-1.89781	-2.19336	-0.06628
	C	-1.21581	0.07786	-0.52670
	C	-2.54731	0.48411	-0.46417
	C	-3.22188	-1.76316	-0.00901
	C	-4.30555	-2.78759	0.19437
	C	-2.85234	1.94836	-0.60283
	C	2.11532	1.06730	0.44531
	C	0.99956	3.01529	-1.13656
	C	1.41707	2.11862	1.06119
	C	1.67947	1.92260	-1.63605
	C	0.82593	3.12541	0.27254
	C	-0.26749	4.08014	2.23222
	C	-0.54699	5.13025	-0.02822
	O	0.38749	-3.44102	1.03693
	F	-3.90730	-3.79540	1.00090
	F	-4.68207	-3.35243	-0.97966
	F	-5.41910	-2.24598	0.74086
	F	-2.16621	2.51623	-1.62563
	F	-4.16306	2.18913	-0.81436
	F	-2.50137	2.64009	0.51621
	H	3.04661	-1.37422	-1.12860
	H	4.68549	0.66500	-0.58528
	H	5.27826	0.81422	1.06917
	H	5.51318	-1.32488	-2.05870
	H	6.67557	-2.46669	-1.38525
	H	6.59861	-1.28945	0.81042
	H	7.02957	-0.14906	-0.46264
	H	4.36113	-3.43705	-1.36947
	H	5.00216	-3.26018	0.26417
	H	4.13812	-1.28337	1.70034
	H	-4.60079	-0.10401	-0.14388
	H	-1.65198	-3.23076	0.10355
	H	-0.44572	0.81159	-0.72444
	H	0.57221	3.73401	-1.82182

	H	1.34717	2.14021	2.14179
	H	1.78301	1.80400	-2.71320
	H	-0.87292	4.95097	2.48616
	H	-0.85956	3.17657	2.43007
	H	0.60860	4.07830	2.89192
	H	-0.99138	5.90779	0.59483
	H	0.16902	5.61410	-0.70144
	H	-1.33923	4.66642	-0.62780
	H	2.63486	0.23122	2.20791
	H	1.09926	-0.89357	-0.73027
	H	2.74595	-3.49241	0.92037

Name	Cat 2O_conf20			
Cartesian Coordinates	N	-0.58659	-3.34284	0.65055
	N	0.67715	-1.41816	0.29784
	N	-3.45639	-1.15830	-0.28605
	N	-1.81047	0.49768	-0.18095
	N	-4.79206	3.48079	-0.47673
	C	-1.84236	-2.62874	0.85574
	C	-3.07139	-3.40797	-1.24328
	C	-3.39344	-4.67127	0.91893
	C	-4.06068	-4.21058	-0.38437
	C	-2.82205	-3.47088	1.68878
	C	-2.46376	-2.20441	-0.50545
	C	0.65564	-2.80144	0.43517
	C	1.83325	-0.63139	0.23564
	C	4.02900	1.13538	0.05138
	C	1.72918	0.69532	0.67167
	C	3.06697	-1.08389	-0.26347
	C	4.14273	-0.20166	-0.33719
	C	2.80629	1.57092	0.55329
	C	2.59756	2.99574	0.98164
	C	5.43992	-0.68006	-0.93297
	C	-3.11984	0.18188	-0.28681
	C	-2.44335	2.83747	-0.32854
	C	-4.14297	1.14144	-0.38969
	C	-1.52293	1.81500	-0.22560
	C	-3.82580	2.50998	-0.39783
	C	-4.40937	4.88023	-0.60938
	C	-6.18655	3.10077	-0.64156
	O	1.66376	-3.50649	0.38075
	F	2.18828	3.07798	2.26954
	F	1.62765	3.59308	0.23682
	F	3.71098	3.74620	0.85992
	F	5.62532	-2.00554	-0.75626
	F	5.48714	-0.45053	-2.26877
	F	6.50577	-0.04397	-0.39289
	H	-1.62891	-1.71595	1.42218
	H	-3.54216	-3.06283	-2.17227
	H	-2.24257	-4.06434	-1.53704
	H	-4.10978	-5.20826	1.55173
	H	-2.59618	-5.39092	0.68047
	H	-4.43139	-5.07118	-0.95354
	H	-4.94154	-3.59797	-0.14148
	H	-3.64844	-2.81115	1.97996
	H	-2.32574	-3.79197	2.61205
	H	-1.66141	-1.79168	-1.12727
	H	4.87193	1.81058	-0.02449
	H	0.80062	1.03693	1.11452
	H	3.17351	-2.11291	-0.57325
	H	-2.09254	3.86049	-0.35592

	H	-5.16801	0.79979	-0.46984
	H	-0.46870	2.07275	-0.18110
	H	-5.30899	5.49631	-0.63974
	H	-3.80679	5.20946	0.24572
	H	-3.83435	5.06601	-1.52743
	H	-6.80679	3.99788	-0.65485
	H	-6.35638	2.55046	-1.57865
	H	-6.52479	2.47078	0.19084
	H	-4.40239	-1.36639	-0.57226
	H	-0.19752	-0.88654	0.27116
	H	-0.56481	-4.34765	0.75003

Name	Cat 2S_conf1			
Cartesian Coordinates	N	2.53127	1.08166	-0.48639
	N	1.06531	2.80908	-0.16435
	N	1.88278	-1.66847	0.00410
	N	0.65447	-0.67085	-1.69206
	N	-2.78664	-2.81049	-0.56632
	C	3.61418	0.15015	-0.17769
	C	4.26227	-2.28685	-0.17880
	C	6.03205	-0.49813	-0.51166
	C	5.61223	-1.94084	-0.82531
	C	4.93048	0.49992	-0.89456
	C	3.17139	-1.28038	-0.57446
	C	2.34596	2.28671	0.06318
	C	-0.11165	2.09085	0.17540
	C	-2.47827	0.74002	0.86223
	C	-0.09190	1.07306	1.13654
	C	-1.32218	2.43213	-0.43933
	C	-2.49085	1.75728	-0.09130
	C	-1.26824	0.40468	1.46460
	C	-1.23552	-0.63698	2.54875
	C	-3.77589	2.03528	-0.81930
	C	0.69479	-1.58315	-0.69948
	C	-1.68521	-1.18350	-2.00493
	C	-0.40977	-2.36917	-0.33657
	C	-0.52465	-0.50858	-2.31667
	C	-1.65340	-2.14495	-0.95637
	C	-2.68557	-3.94029	0.34774
	C	-4.05815	-2.53314	-1.22205
	F	-0.07657	-1.33633	2.55119
	F	-2.24721	-1.52966	2.42301
	F	-1.35641	-0.08250	3.77712
	F	-3.75763	3.21577	-1.47079
	F	-4.03395	1.07469	-1.75037
	F	-4.83619	2.04337	0.01934
	H	3.78120	0.18407	0.90783
	H	4.36686	-2.28715	0.91678
	H	3.94166	-3.29378	-0.47315
	H	6.24398	-0.40403	0.56312
	H	6.96187	-0.25054	-1.03737
	H	5.52651	-2.06397	-1.91427
	H	6.37794	-2.64915	-0.48668
	H	5.21933	1.52086	-0.63326
	H	4.75664	0.47372	-1.97980
	H	3.06030	-1.29811	-1.66643
	H	-3.38687	0.20684	1.11428
	H	0.83867	0.79192	1.61363
	H	-1.34008	3.20690	-1.19777
	H	-2.59227	-0.95439	-2.54531
	H	-0.30330	-3.08320	0.46955

	H	-0.53219	0.23535	-3.11101
	H	-2.01982	-4.72112	-0.04592
	H	-2.31127	-3.62431	1.32678
	H	-3.67525	-4.37663	0.48961
	H	-4.85785	-3.03587	-0.67581
	H	-4.27152	-1.45890	-1.21640
	H	-4.07855	-2.88335	-2.26472
	H	1.91623	-2.38126	0.71980
	H	1.01605	3.80456	0.02195
	H	1.78465	0.68881	-1.08316
	S	3.48036	3.16333	0.95485

Name	Cat 2S_conf2
Cartesian Coordinates	N 2.53134 1.08189 -0.48544<br>N 1.06541 2.80920 -0.16279<br>N 1.88335 -1.66830 0.00452<br>N 0.65469 -0.67048 -1.69130<br>N -2.78545 -2.81232 -0.56715<br>C 3.61448 0.15042 -0.17733<br>C 4.26290 -2.28648 -0.17957<br>C 6.03235 -0.49736 -0.51207<br>C 5.61266 -1.93999 -0.82628<br>C 4.93056 0.50073 -0.89433<br>C 3.17180 -1.28000 -0.57452<br>C 2.34599 2.28667 0.06466<br>C -0.11172 2.09083 0.17616<br>C -2.47893 0.74023 0.86143<br>C -1.32195 2.43264 -0.43889<br>C -0.09254 1.07246 1.13671<br>C -1.26921 0.40430 1.46408<br>C -2.49090 1.75790 -0.09167<br>C -3.77553 2.03624 -0.82025<br>C -1.23733 -0.63772 2.54790<br>C 0.69533 -1.58334 -0.69920<br>C -1.68478 -1.18380 -2.00448<br>C -0.40883 -2.37009 -0.33691<br>C -0.52443 -0.50829 -2.31583<br>C -1.65259 -2.14593 -0.95658<br>C -4.05762 -2.53319 -1.22083<br>C -2.68438 -3.94144 0.34767<br>F -4.03300 1.07625 -1.75197<br>F -3.75696 3.21716 -1.47100<br>F -4.83633 2.04378 0.01783<br>F -1.35846 -0.08354 3.77640<br>F -2.24940 -1.52989 2.42154<br>F -0.07873 -1.33759 2.55067<br>H 3.78173 0.18391 0.90817<br>H 4.36779 -2.28724 0.91598<br>H 3.94240 -3.29335 -0.47429<br>H 6.96206 -0.24941 -1.03781<br>H 6.24444 -0.40371 0.56273<br>H 5.52667 -2.06263 -1.91528<br>H 6.37855 -2.64836 -0.48818<br>H 5.21930 1.52158 -0.63262<br>H 4.75653 0.47497 -1.97955<br>H 3.06034 -1.29736 -1.66645<br>H -3.38779 0.20727 1.11299<br>H -1.33940 3.20782 -1.19693<br>H 0.83781 0.79069 1.61386<br>H -2.59191 -0.95469 -2.54474<br>H -0.30211 -3.08469 0.46868

	H	-0.53228	0.23613	-3.10971
	H	-4.27054	-1.45885	-1.21319
	H	-4.07937	-2.88161	-2.26406
	H	-4.85688	-3.03658	-0.67457
	H	-3.67394	-4.37819	0.48915
	H	-2.01810	-4.72217	-0.04524
	H	-2.31075	-3.62482	1.32678
	H	1.91751	-2.38286	0.71846
	H	1.01588	3.80448	0.02452
	H	1.78469	0.68910	-1.08223
	S	3.48036	3.16290	0.95677

Name	Cat 2S_conf3			
Cartesian Coordinates	N	-2.19864	-0.35741	0.85975
	N	-0.45742	-0.51602	2.39097
	N	-2.53028	1.88956	-0.93431
	N	-0.69614	0.58175	-1.52433
	N	1.92023	3.79113	-0.74096
	C	-3.53692	0.02380	0.39751
	C	-4.83512	1.18047	-1.42184
	C	-5.87402	-0.75870	-0.15304
	C	-5.80095	-0.01271	-1.49341
	C	-4.47768	-1.18751	0.31807
	C	-3.43413	0.74518	-0.96646
	C	-1.78995	-0.26186	2.13605
	C	0.55760	-0.96469	1.52287
	C	2.65876	-1.85916	-0.11201
	C	0.35971	-2.03933	0.64736
	C	1.81226	-0.34257	1.57912
	C	2.84856	-0.79516	0.76985
	C	1.40627	-2.46574	-0.16895
	C	1.15354	-3.53240	-1.19981
	C	4.19149	-0.11744	0.79215
	C	-1.16108	1.77428	-1.09648
	C	1.53978	1.50981	-1.51112
	C	-0.32771	2.87294	-0.82678
	C	0.62886	0.49828	-1.73849
	C	1.06182	2.75631	-1.01726
	C	1.40972	5.00826	-0.12877
	C	3.36113	3.57921	-0.81296
	F	0.78516	-2.97930	-2.38386
	F	0.16394	-4.37301	-0.83295
	F	2.25678	-4.27255	-1.44025
	F	4.57326	0.24983	-0.45817
	F	4.19340	1.00155	1.55219
	F	5.15873	-0.93094	1.26707
	H	-3.93891	0.73202	1.13081
	H	-5.22754	1.92646	-0.71386
	H	-4.75121	1.67639	-2.39519
	H	-6.32342	-0.10226	0.60580
	H	-6.52767	-1.63489	-0.23844
	H	-5.45634	-0.70531	-2.27475
	H	-6.79706	0.33259	-1.79532
	H	-4.52335	-1.66322	1.30363
	H	-4.05292	-1.92137	-0.38176
	H	-3.02582	0.04741	-1.70389
	H	3.46403	-2.19554	-0.75502
	H	-0.59971	-2.54116	0.60798
	H	1.96240	0.50657	2.23596
	H	2.59233	1.32088	-1.66950
	H	-0.77397	3.79259	-0.46857

	H	0.97642	-0.46758	-2.09505
	H	0.95187	4.81655	0.85297
	H	2.22959	5.71464	0.00760
	H	0.65745	5.48674	-0.76823
	H	3.87085	4.51498	-0.57942
	H	3.70274	2.81256	-0.10615
	H	3.66580	3.27584	-1.82164
	H	-2.84055	2.67190	-0.37193
	H	-0.17887	-0.25268	3.32730
	H	-1.49905	-0.42302	0.11679
	S	-2.78215	0.16425	3.44763

Name	Cat 2S_conf4			
Cartesian Coordinates	N	-2.57380	-1.28861	-0.82066
	N	-0.34025	-0.86653	-0.89098
	N	-3.36099	0.96191	0.75234
	N	-2.46392	1.64278	-1.29391
	N	0.54302	3.81087	0.71312
	C	-3.84647	-1.33921	-0.10129
	C	-5.65345	0.06990	0.95524
	C	-6.20830	-2.20960	-0.03024
	C	-6.72167	-0.79092	0.26194
	C	-4.89822	-2.18107	-0.83310
	C	-4.35989	0.10657	0.12881
	C	-1.36917	-1.52193	-0.23843
	C	1.03088	-0.89865	-0.56750
	C	3.78001	-0.87146	0.01867
	C	1.72649	0.31007	-0.50052
	C	1.71913	-2.10380	-0.36519
	C	3.07749	-2.07697	-0.06614
	C	3.09317	0.31515	-0.21196
	C	3.78946	1.64271	-0.11595
	C	3.83049	-3.37053	0.09562
	C	-2.39373	1.65782	0.05826
	C	-0.57805	3.16818	-1.35203
	C	-1.39856	2.35571	0.76570
	C	-1.57031	2.41194	-1.94543
	C	-0.45637	3.13437	0.06850
	C	1.42173	4.70903	-0.02700
	C	0.76156	3.59796	2.13669
	F	5.11952	1.52393	0.06077
	F	3.31210	2.38477	0.91724
	F	3.59359	2.38443	-1.23711
	F	4.80592	-3.26543	1.02728
	F	3.02443	-4.38899	0.45935
	F	4.43620	-3.73676	-1.06086
	H	-3.63727	-1.79849	0.86912
	H	-6.01375	1.09394	1.10494
	H	-5.43417	-0.34420	1.95102
	H	-6.03307	-2.73440	0.91970
	H	-6.96861	-2.78468	-0.57189
	H	-7.00860	-0.30820	-0.68336
	H	-7.62589	-0.83302	0.88081
	H	-4.50750	-3.19342	-0.98360
	H	-5.07890	-1.75193	-1.82961
	H	-4.58193	0.55192	-0.84660
	H	4.83588	-0.86340	0.25905
	H	1.20068	1.24494	-0.66155
	H	1.19329	-3.04574	-0.43479
	H	0.10143	3.73951	-1.97040
	H	-1.39061	2.30121	1.84737

	H	-1.65423	2.40071	-3.03132
	H	2.07106	5.22873	0.67813
	H	2.05895	4.16983	-0.73634
	H	0.84299	5.46318	-0.57389
	H	-0.07233	3.98111	2.74075
	H	0.89143	2.53133	2.35681
	H	1.67168	4.11493	2.44068
	H	-3.16233	0.77650	1.72675
	H	-0.62546	-0.04106	-1.41039
	H	-2.57431	-0.58579	-1.55903
	S	-1.15065	-2.49661	1.12439

Name	Cat 2S_conf5			
Cartesian Coordinates	N	-0.71839	3.27927	-0.24607
	N	0.57226	1.38216	-0.07391
	N	-3.42780	0.85669	0.63245
	N	-1.72230	-0.58048	-0.04723
	N	-4.57053	-3.54020	-1.01153
	C	-1.96646	2.54839	-0.46156
	C	-3.11413	3.01738	1.78287
	C	-3.58565	4.52295	-0.18815
	C	-4.17217	3.87509	1.07302
	C	-3.01288	3.45402	-1.13067
	C	-2.49196	1.94105	0.87894
	C	0.51242	2.74865	-0.03201
	C	1.71135	0.56288	0.05756
	C	3.82719	-1.28346	0.23047
	C	1.56712	-0.61639	0.79554
	C	2.92649	0.82352	-0.59001
	C	3.97229	-0.09090	-0.48170
	C	2.61122	-1.53853	0.85740
	C	2.35907	-2.84663	1.55208
	C	5.29392	0.23118	-1.12681
	C	-3.04666	-0.36206	0.11505
	C	-2.25079	-2.80838	-0.84247
	C	-4.02775	-1.32044	-0.19298
	C	-1.37356	-1.79725	-0.51387
	C	-3.64793	-2.57899	-0.69051
	C	-4.13081	-4.86247	-1.43812
	C	-5.98409	-3.29734	-0.76624
	F	1.56056	-3.64894	0.79897
	F	1.72320	-2.67533	2.73325
	F	3.49565	-3.53235	1.79375
	F	6.08202	0.96582	-0.30844
	F	5.98749	-0.88872	-1.43825
	F	5.13874	0.94190	-2.26620
	H	-1.75203	1.72357	-1.14779
	H	-3.52888	2.53480	2.67527
	H	-2.30046	3.67007	2.12331
	H	-4.34898	5.10654	-0.71575
	H	-2.80251	5.23812	0.10387
	H	-4.54796	4.64105	1.76118
	H	-5.04269	3.26218	0.79397
	H	-3.83086	2.80713	-1.47269
	H	-2.57424	3.90805	-2.02678
	H	-1.64085	1.51069	1.41463
	H	4.63892	-1.99791	0.28483
	H	0.63098	-0.81708	1.30509
	H	3.04520	1.72284	-1.17792
	H	-1.86016	-3.75061	-1.20233
	H	-5.06808	-1.07029	-0.02448

	H	-0.30521	-1.97007	-0.61923
	H	-3.52158	-4.80496	-2.34842
	H	-3.54292	-5.37055	-0.66132
	H	-5.00500	-5.47589	-1.65931
	H	-6.19215	-3.13093	0.30038
	H	-6.33868	-2.42171	-1.32510
	H	-6.56176	-4.16162	-1.09584
	H	-4.41151	1.07941	0.59784
	H	-0.30192	0.83452	-0.06324
	H	-0.75077	4.28553	-0.15857
	S	1.82718	3.77938	0.27610

Name	Cat 2S_conf6
Cartesian Coordinates	N -0.65149 3.34532 -0.14752<br>N 0.59184 1.41365 -0.03357<br>N -3.47836 0.96617 0.32786<br>N -1.74921 -0.48539 -0.26655<br>N -4.52566 -3.62116 -0.77231<br>C -1.89166 2.65826 -0.50690<br>C -3.20926 3.03311 1.65552<br>C -3.49727 4.64733 -0.26009<br>C -4.19708 3.94081 0.90820<br>C -2.86294 3.62040 -1.20924<br>C -2.53257 1.99257 0.74941<br>C 0.55515 2.77543 0.09643<br>C 1.71089 0.56508 0.08450<br>C 3.78318 -1.33446 0.19331<br>C 2.95236 0.84412 -0.50450<br>C 1.52198 -0.65588 0.73807<br>C 2.54497 -1.60400 0.76670<br>C 3.97505 -0.09796 -0.42895<br>C 5.32734 0.22724 -1.00527<br>C 2.24383 -2.94906 1.36437<br>C -3.06902 -0.29908 -0.04629<br>C -2.23620 -2.78723 -0.85074<br>C -4.02657 -1.31525 -0.20326<br>C -1.38245 -1.72121 -0.66551<br>C -3.62739 -2.59981 -0.61222<br>C -5.93738 -3.39484 -0.50285<br>C -4.06855 -4.94094 -1.18731<br>F 6.15996 0.73088 -0.06480<br>F 5.25200 1.14008 -1.99774<br>F 5.93183 -0.87414 -1.51131<br>F 1.43401 -3.67232 0.54604<br>F 3.35543 -3.68417 1.57406<br>F 1.59389 -2.84067 2.54554<br>H -1.63834 1.86662 -1.21756<br>H -3.69736 2.51813 2.49268<br>H -2.41600 3.65305 2.09197<br>H -4.20651 5.27164 -0.81589<br>H -2.73285 5.33242 0.13576<br>H -4.62442 4.67310 1.60304<br>H -5.03903 3.34961 0.51969<br>H -3.65832 3.00665 -1.64861<br>H -2.34189 4.11348 -2.03804<br>H -1.73151 1.51667 1.32817<br>H 4.57758 -2.07008 0.21944<br>H 3.10783 1.77999 -1.02233<br>H 0.56818 -0.86982 1.20734<br>H -1.83073 -3.73996 -1.16363<br>H -5.06600 -1.08382 -0.00484

	H	-0.31807	-1.86276	-0.83455
	H	-6.35288	-2.61761	-1.15853
	H	-6.49163	-4.31729	-0.67885
	H	-6.10535	-3.08970	0.53915
	H	-3.36237	-5.37120	-0.46504
	H	-4.92649	-5.61014	-1.26089
	H	-3.57949	-4.90828	-2.16952
	H	-4.41450	1.03082	0.70185
	H	-0.29187	0.88702	-0.11058
	H	-0.67112	4.34485	0.00044
	S	1.87068	3.75262	0.54547

Name	Cat 2S_conf7			
Cartesian Coordinates	N	-0.65125	3.34575	-0.14593
	N	0.59192	1.41388	-0.03349
	N	-3.47858	0.96646	0.32570
	N	-1.74931	-0.48510	-0.26828
	N	-4.52660	-3.61919	-0.78090
	C	-1.89131	2.65916	-0.50665
	C	-3.21020	3.03222	1.65537
	C	-3.49673	4.64825	-0.25897
	C	-4.19738	3.94080	0.90824
	C	-2.86203	3.62208	-1.20869
	C	-2.53299	1.99248	0.74868
	C	0.55537	2.77550	0.09749
	C	1.71089	0.56509	0.08462
	C	3.78293	-1.33459	0.19338
	C	2.95241	0.84413	-0.50426
	C	1.52174	-0.65589	0.73801
	C	2.54468	-1.60412	0.76664
	C	3.97503	-0.09803	-0.42874
	C	5.32736	0.22713	-1.00498
	C	2.24341	-2.94926	1.36406
	C	-3.06927	-0.29858	-0.04904
	C	-2.23633	-2.78669	-0.85342
	C	-4.02688	-1.31454	-0.20703
	C	-1.38245	-1.72099	-0.66692
	C	-3.62782	-2.59873	-0.61724
	C	-5.93636	-3.39654	-0.49821
	C	-4.06719	-4.94347	-1.17873
	F	5.25188	1.13922	-1.99818
	F	5.93227	-0.87444	-1.51010
	F	6.15962	0.73175	-0.06475
	F	1.59299	-2.84106	2.54497
	F	1.43400	-3.67251	0.54529
	F	3.35500	-3.68429	1.57411
	H	-1.63771	1.86813	-1.21790
	H	-3.69900	2.51643	2.49161
	H	-2.41722	3.65165	2.09302
	H	-4.20553	5.27321	-0.81460
	H	-2.73239	5.33278	0.13800
	H	-4.62496	4.67256	1.60350
	H	-5.03925	3.35014	0.51874
	H	-3.65727	3.00886	-1.64905
	H	-2.34043	4.11582	-2.03675
	H	-1.73236	1.51598	1.32751
	H	4.57729	-2.07026	0.21956
	H	3.10791	1.78005	-1.02199
	H	0.56784	-0.86977	1.20712
	H	-1.83066	-3.73969	-1.16525
	H	-5.06625	-1.08333	-0.00809

	H	-0.31788	-1.86299	-0.83434
	H	-6.09813	-3.10385	0.54855
	H	-6.35544	-2.61159	-1.14195
	H	-6.49197	-4.31649	-0.68282
	H	-4.92601	-5.61057	-1.26081
	H	-3.56442	-4.91864	-2.15398
	H	-3.37233	-5.37101	-0.44369
	H	-4.41529	1.03142	0.69822
	H	-0.29175	0.88731	-0.11128
	H	-0.67057	4.34529	0.00201
	S	1.87108	3.75222	0.54695

Name	Cat 2S_conf8			
Cartesian Coordinates	N	2.24131	-0.74377	0.63997
	N	0.69442	-2.28216	1.42871
	N	2.55169	-0.00116	-2.13312
	N	0.58198	-1.00590	-2.78063
	N	-1.70512	2.22106	-1.29973
	C	3.54584	-0.29707	0.14380
	C	4.74082	1.01989	-1.63403
	C	5.74197	0.81993	0.69224
	C	5.59995	1.70098	-0.55785
	C	4.36857	0.40203	1.23583
	C	3.36084	0.61742	-1.08919
	C	2.00688	-1.96903	1.15123
	C	-0.45855	-1.48357	1.25030
	C	-2.80503	0.02394	0.95363
	C	-1.52855	-1.98664	0.50408
	C	-0.55847	-0.21365	1.83547
	C	-1.72472	0.53091	1.67236
	C	-2.69090	-1.23167	0.36198
	C	-3.82979	-1.78474	-0.45278
	C	-1.83414	1.88091	2.32796
	C	1.16435	0.05237	-2.18275
	C	-1.56564	0.07606	-2.46669
	C	0.44109	1.14968	-1.68294
	C	-0.75073	-0.95366	-2.90992
	C	-0.96356	1.17686	-1.80692
	C	-3.11167	2.35408	-1.65707
	C	-1.02153	3.41619	-0.82238
	F	-3.38842	-2.54260	-1.47977
	F	-4.59505	-0.79613	-0.98115
	F	-4.65022	-2.55998	0.28984
	F	-2.71075	2.68671	1.68427
	F	-2.25236	1.78648	3.60904
	F	-0.63861	2.51928	2.35145
	H	4.08571	-1.19518	-0.17815
	H	5.25145	0.11751	-2.00027
	H	4.59697	1.67670	-2.49877
	H	6.31571	-0.08272	0.43883
	H	6.30941	1.34583	1.46906
	H	5.13182	2.65572	-0.27654
	H	6.58662	1.94505	-0.96921
	H	3.81513	1.28511	1.58682
	H	4.47296	-0.27931	2.08651
	H	2.85464	1.53392	-0.76662
	H	-3.70297	0.61467	0.82742
	H	-1.43469	-2.94599	0.00750
	H	0.26630	0.18014	2.41881
	H	-2.63343	0.00995	-2.62117
	H	0.96904	1.99155	-1.25924

	H	-1.20838	-1.81284	-3.39867
	H	-3.65090	1.42492	-1.46240
	H	-3.56077	3.12957	-1.03407
	H	-3.25483	2.62192	-2.71466
	H	-0.46488	3.92720	-1.62201
	H	-1.75813	4.10666	-0.41110
	H	-0.32593	3.16676	-0.01585
	H	2.88721	-0.90895	-2.44021
	H	0.55184	-3.26400	1.62775
	H	1.44015	-0.19196	0.35051
	S	3.20417	-3.12624	1.46299

Name	Cat 2S_conf9			
Cartesian Coordinates	N	2.35933	-2.58057	0.68873
	N	0.44553	-1.52198	-0.04675
	N	2.89891	0.31829	1.03347
	N	1.99683	0.81805	-1.05304
	N	0.46055	4.45853	0.41181
	C	3.31793	-1.87119	-0.15794
	C	5.00592	0.03542	-0.30855
	C	5.50621	-2.17555	-1.42326
	C	6.10434	-0.95995	-0.70377
	C	4.43472	-2.85137	-0.55829
	C	3.91666	-0.62907	0.55728
	C	1.01743	-2.43481	0.79546
	C	-0.91585	-1.14014	-0.05591
	C	-3.58277	-0.27099	-0.20233
	C	-1.22336	0.21653	0.06076
	C	-1.94642	-2.06927	-0.24462
	C	-3.26625	-1.62847	-0.30536
	C	-2.54943	0.64346	-0.02327
	C	-2.83337	2.11770	0.03345
	C	-4.38223	-2.62852	-0.44468
	C	2.22308	1.21154	0.21934
	C	0.78185	2.88052	-1.42013
	C	1.72888	2.41491	0.74775
	C	1.30142	1.67009	-1.83072
	C	0.97588	3.28429	-0.06857
	C	0.44409	4.69251	1.85017
	C	-0.40382	5.27211	-0.43770
	F	-2.60751	2.71588	-1.16735
	F	-2.03457	2.75343	0.92731
	F	-4.11165	2.38614	0.37000
	F	-4.91288	-2.95417	0.75689
	F	-5.39669	-2.13973	-1.19765
	F	-3.96749	-3.77797	-1.02019
	H	2.81110	-1.53749	-1.06534
	H	4.53504	0.44464	-1.20934
	H	5.42750	0.88129	0.24820
	H	5.05589	-1.85300	-2.37254
	H	6.28955	-2.90046	-1.67412
	H	6.63669	-1.29891	0.19722
	H	6.84677	-0.46415	-1.34053
	H	3.98595	-3.70350	-1.08082
	H	4.90490	-3.24752	0.35544
	H	4.38977	-1.00763	1.47285
	H	-4.61227	0.06092	-0.26328
	H	-0.43645	0.93740	0.23864
	H	-1.71262	-3.12031	-0.34524
	H	0.20328	3.47306	-2.11504
	H	1.93365	2.65513	1.78367

	H	1.13007	1.33219	-2.85089
	H	-0.09858	3.89872	2.38188
	H	1.46189	4.75173	2.25373
	H	-0.04915	5.64363	2.05345
	H	-0.67000	6.18414	0.09834
	H	0.12027	5.56673	-1.35344
	H	-1.32457	4.74375	-0.71178
	H	3.07300	0.66507	1.96654
	H	1.06153	-0.84121	-0.50171
	H	2.74270	-3.23898	1.35483
	S	0.15710	-3.37513	1.92149

Name	Cat 2S_conf10			
Cartesian Coordinates	N	2.24080	-0.74501	0.63842
	N	0.69439	-2.28283	1.42907
	N	2.55170	0.00042	-2.13417
	N	0.58184	-1.00503	-2.78059
	N	-1.70544	2.22102	-1.29784
	C	3.54527	-0.29743	0.14307
	C	4.74085	1.02074	-1.63344
	C	5.74092	0.81969	0.69324
	C	5.59952	1.70132	-0.55653
	C	4.36730	0.40129	1.23590
	C	3.36065	0.61796	-1.08935
	C	2.00671	-1.96998	1.15033
	C	-0.45854	-1.48416	1.25052
	C	-2.80455	0.02394	0.95343
	C	-1.52868	-1.98718	0.50451
	C	-0.55809	-0.21405	1.83536
	C	-1.72411	0.53082	1.67205
	C	-2.69081	-1.23188	0.36218
	C	-3.82966	-1.78484	-0.45270
	C	-1.83324	1.88104	2.32730
	C	1.16420	0.05343	-2.18312
	C	-1.56592	0.07623	-2.46531
	C	0.44091	1.15054	-1.68301
	C	-0.75097	-0.95328	-2.90902
	C	-0.96383	1.17720	-1.80593
	C	-1.02180	3.41659	-0.82158
	C	-3.11194	2.35444	-1.65546
	F	-4.59631	-0.79624	-0.97901
	F	-4.64871	-2.56231	0.28907
	F	-3.38801	-2.54051	-1.48125
	F	-0.63755	2.51905	2.35080
	F	-2.70955	2.68703	1.68336
	F	-2.25165	1.78706	3.60836
	H	4.08556	-1.19516	-0.17921
	H	5.25167	0.11858	-1.99994
	H	4.59732	1.67802	-2.49787
	H	6.31492	-0.08276	0.43973
	H	6.30783	1.34528	1.47065
	H	5.13127	2.65595	-0.27503
	H	6.58639	1.94554	-0.96731
	H	4.47147	-0.28042	2.08632
	H	3.81347	1.28406	1.58706
	H	2.85451	1.53429	-0.76621
	H	-3.70229	0.61490	0.82704
	H	-1.43509	-2.94668	0.00816
	H	0.26681	0.17965	2.41861
	H	-2.63380	0.00959	-2.61894
	H	0.96889	1.99261	-1.25974

	H	-1.20866	-1.81263	-3.39745
	H	-1.75819	4.10654	-0.40907
	H	-0.32484	3.16748	-0.01612
	H	-0.46668	3.92796	-1.62205
	H	-3.65133	1.42522	-1.46157
	H	-3.56103	3.12949	-1.03193
	H	-3.25481	2.62310	-2.71289
	H	2.88723	-0.90739	-2.44133
	H	0.55179	-3.26469	1.62801
	H	1.43957	-0.19267	0.35017
	S	3.20411	-3.12732	1.46125

Name	Cat 2S_conf11			
Cartesian Coordinates	N	-0.68710	3.34354	-0.13338
	N	0.57870	1.42689	-0.01528
	N	-3.48786	0.93044	0.32104
	N	-1.73675	-0.50051	-0.25909
	N	-4.47390	-3.66466	-0.80132
	C	-1.91640	2.64200	-0.50164
	C	-3.25495	3.00015	1.65092
	C	-3.54867	4.61090	-0.26696
	C	-4.24821	3.89564	0.89620
	C	-2.89412	3.59217	-1.21121
	C	-2.55818	1.96837	0.74987
	C	0.52516	2.78791	0.11568
	C	1.70722	0.59138	0.10759
	C	3.80780	-1.27454	0.24270
	C	1.53210	-0.62889	0.76569
	C	2.94749	0.88523	-0.47724
	C	3.98378	-0.03993	-0.38884
	C	2.57009	-1.56058	0.80819
	C	2.28920	-2.90160	1.42468
	C	5.29819	0.24557	-1.06494
	C	-3.06070	-0.32915	-0.05176
	C	-2.19271	-2.80608	-0.85358
	C	-4.00544	-1.35536	-0.22054
	C	-1.35245	-1.73139	-0.65686
	C	-3.58823	-2.63430	-0.62895
	C	-5.89006	-3.45514	-0.54169
	C	-3.99824	-4.97923	-1.21198
	F	1.64602	-2.78653	2.60901
	F	3.41090	-3.62071	1.63581
	F	1.48245	-3.64388	0.62084
	F	6.34117	-0.23372	-0.34831
	F	5.36328	-0.34436	-2.28469
	F	5.50249	1.56516	-1.25314
	H	-1.64851	1.85350	-1.21049
	H	-3.74318	2.47873	2.48399
	H	-2.47310	3.62984	2.09400
	H	-4.26169	5.22604	-0.82815
	H	-2.79618	5.30584	0.13457
	H	-4.68949	4.62262	1.58790
	H	-5.08019	3.29428	0.50178
	H	-3.67837	2.96869	-1.65692
	H	-2.37285	4.09192	-2.03587
	H	-1.75565	1.50227	1.33450
	H	4.61867	-1.99094	0.29319
	H	0.57968	-0.85303	1.23310
	H	3.09469	1.82482	-0.99085
	H	-1.77398	-3.75359	-1.16490
	H	-5.04926	-1.13579	-0.03195

	H	-0.28481	-1.86091	-0.81496
	H	-6.43268	-4.38291	-0.72580
	H	-6.06944	-3.15601	0.50020
	H	-6.30908	-2.67995	-1.19750
	H	-4.84845	-5.65683	-1.29752
	H	-3.49726	-4.94011	-2.18787
	H	-3.29687	-5.40356	-0.48147
	H	-4.42827	0.98409	0.68579
	H	-0.29828	0.88951	-0.09552
	H	-0.71904	4.34283	0.01381
	S	1.82683	3.78041	0.57160

Name	Cat 2S_conf12			
Cartesian Coordinates	N	-0.68697	3.34361	-0.13367
	N	0.57868	1.42685	-0.01571
	N	-3.48793	0.93063	0.32039
	N	-1.73677	-0.50029	-0.25971
	N	-4.47375	-3.66470	-0.80123
	C	-1.91635	2.64221	-0.50189
	C	-3.25492	3.00006	1.65073
	C	-3.54852	4.61116	-0.26687
	C	-4.24813	3.89573	0.89614
	C	-2.89404	3.59254	-1.21131
	C	-2.55821	1.96841	0.74952
	C	0.52514	2.78782	0.11576
	C	1.70715	0.59131	0.10738
	C	3.80771	-1.27463	0.24266
	C	2.94752	0.88521	-0.47723
	C	1.53195	-0.62898	0.76540
	C	2.56992	-1.56067	0.80798
	C	3.98379	-0.03997	-0.38877
	C	5.29826	0.24565	-1.06468
	C	2.28896	-2.90170	1.42443
	C	-3.06067	-0.32903	-0.05221
	C	-2.19260	-2.80598	-0.85378
	C	-4.00536	-1.35533	-0.22071
	C	-1.35240	-1.73120	-0.65735
	C	-3.58812	-2.63428	-0.62905
	C	-3.99807	-4.97937	-1.21159
	C	-5.88974	-3.45549	-0.54041
	F	5.36316	-0.34344	-2.28486
	F	6.34114	-0.23443	-0.34841
	F	5.50290	1.56530	-1.25195
	F	1.48208	-3.64388	0.62060
	F	3.41060	-3.62090	1.63548
	F	1.64585	-2.78659	2.60880
	H	-1.64853	1.85377	-1.21083
	H	-3.74319	2.47854	2.48371
	H	-2.47305	3.62965	2.09393
	H	-4.26146	5.22648	-0.82795
	H	-2.79597	5.30595	0.13482
	H	-4.68942	4.62260	1.58795
	H	-5.08011	3.29445	0.50159
	H	-3.67833	2.96917	-1.65709
	H	-2.37276	4.09240	-2.03590
	H	-1.75571	1.50215	1.33407
	H	4.61857	-1.99104	0.29316
	H	3.09478	1.82486	-0.99071
	H	0.57947	-0.85316	1.23268
	H	-1.77381	-3.75354	-1.16485
	H	-5.04918	-1.13580	-0.03207

	H	-0.28477	-1.86065	-0.81553
	H	-4.84842	-5.65669	-1.29802
	H	-3.49630	-4.94020	-2.18705
	H	-3.29742	-5.40402	-0.48058
	H	-6.06833	-3.15643	0.50164
	H	-6.30945	-2.68038	-1.19586
	H	-6.43232	-4.38336	-0.72412
	H	-4.42820	0.98408	0.68553
	H	-0.29831	0.88952	-0.09607
	H	-0.71905	4.34276	0.01447
	S	1.82671	3.78005	0.57254

Name	Cat 2S_conf13			
Cartesian Coordinates	N	2.17993	0.83960	-0.55583
	N	0.48725	2.34883	-1.06265
	N	2.83866	-0.10747	2.10764
	N	0.91669	0.95564	2.82819
	N	-1.50531	-2.19588	1.39450
	C	3.54871	0.41830	-0.23814
	C	4.97452	-1.00733	1.26881
	C	5.70683	-0.55492	-1.11967
	C	5.72591	-1.55461	0.04587
	C	4.27081	-0.14403	-1.47220
	C	3.53299	-0.60676	0.92050
	C	1.83595	2.08859	-0.92750
	C	-0.62087	1.48310	-1.01800
	C	-2.92695	-0.12558	-0.99691
	C	-0.59628	0.20272	-1.58930
	C	-1.80696	1.94969	-0.43266
	C	-2.94526	1.15047	-0.43430
	C	-1.74084	-0.59193	-1.55536
	C	-1.71479	-1.95254	-2.19735
	C	-4.19899	1.61079	0.25881
	C	1.44897	-0.11951	2.21929
	C	-1.27611	-0.03286	2.51363
	C	0.68360	-1.20110	1.74907
	C	-0.41756	0.95706	2.96375
	C	-0.72103	-1.17049	1.87384
	C	-0.87170	-3.43579	0.96417
	C	-2.91913	-2.24903	1.74336
	F	-2.54885	-2.81721	-1.57236
	F	-0.47400	-2.49600	-2.17158
	F	-2.09639	-1.90579	-3.49250
	F	-5.30333	1.29998	-0.45316
	F	-4.34091	1.00410	1.47046
	F	-4.20870	2.93907	0.48104
	H	4.08519	1.31075	0.10410
	H	5.50011	-0.12475	1.66175
	H	4.94554	-1.74524	2.07784
	H	6.27784	0.34188	-0.84058
	H	6.20170	-0.98236	-1.99967
	H	5.25295	-2.49426	-0.27531
	H	6.75760	-1.80155	0.32316
	H	4.26044	0.61579	-2.26054
	H	3.71011	-1.01320	-1.84549
	H	3.01104	-1.50754	0.57760
	H	-3.81324	-0.74816	-0.98443
	H	0.30045	-0.16481	-2.07301
	H	-1.82949	2.92946	0.03138
	H	-2.34093	0.08754	2.65203
	H	1.18302	-2.06683	1.33777

	H	-0.83474	1.82973	3.46530
	H	-0.34057	-3.94039	1.78482
	H	-1.63575	-4.10794	0.57353
	H	-0.16360	-3.24478	0.15287
	H	-3.39642	-3.03670	1.15792
	H	-3.08167	-2.45354	2.81217
	H	-3.41511	-1.30838	1.49425
	H	3.20026	0.78482	2.43396
	H	0.28743	3.33697	-1.15127
	H	1.44064	0.23208	-0.21723
	S	2.93346	3.34272	-1.22320

Name	Cat 2S_conf14			
Cartesian Coordinates	N	2.17982	0.83914	-0.55618
	N	0.48688	2.34827	-1.06228
	N	2.83921	-0.10659	2.10766
	N	0.91746	0.95687	2.82851
	N	-1.50495	-2.19466	1.39566
	C	3.54872	0.41817	-0.23861
	C	4.97508	-1.00627	1.26893
	C	5.70686	-0.55512	-1.11994
	C	5.72647	-1.55406	0.04621
	C	4.27066	-0.14483	-1.47246
	C	3.53340	-0.60628	0.92061
	C	1.83567	2.08801	-0.92827
	C	-0.62118	1.48245	-1.01783
	C	-2.92727	-0.12621	-0.99722
	C	-0.59636	0.20189	-1.58882
	C	-1.80742	1.94911	-0.43294
	C	-2.94577	1.14987	-0.43477
	C	-1.74093	-0.59268	-1.55520
	C	-1.71490	-1.95348	-2.19678
	C	-4.19958	1.61033	0.25809
	C	1.44954	-0.11835	2.21954
	C	-1.27553	-0.03129	2.51409
	C	0.68397	-1.19987	1.74950
	C	-0.41680	0.95853	2.96410
	C	-0.72062	-1.16914	1.87456
	C	-2.91879	-2.24754	1.74446
	C	-0.87127	-3.43468	0.96565
	F	-2.09795	-1.90740	-3.49148
	F	-2.54792	-2.81830	-1.57055
	F	-0.47382	-2.49640	-2.17204
	F	-4.34148	1.00396	1.46999
	F	-4.20944	2.93865	0.47993
	F	-5.30391	1.29921	-0.45375
	H	4.08526	1.31082	0.10306
	H	5.50049	-0.12334	1.66132
	H	4.94642	-1.74374	2.07837
	H	6.27764	0.34203	-0.84152
	H	6.20170	-0.98297	-1.99975
	H	5.25378	-2.49408	-0.27427
	H	6.75829	-1.80047	0.32348
	H	4.25995	0.61441	-2.26132
	H	3.71006	-1.01439	-1.84501
	H	3.01166	-1.50737	0.57825
	H	-3.81356	-0.74880	-0.98494
	H	0.30062	-0.16584	-2.07191
	H	-1.83006	2.92893	0.03098
	H	-2.34036	0.08937	2.65225
	H	1.18320	-2.06563	1.33798

	H	-0.83382	1.83134	3.46551
	H	-3.08133	-2.45125	2.81342
	H	-3.41471	-1.30705	1.49464
	H	-3.39610	-3.03561	1.15959
	H	-1.63554	-4.10791	0.57731
	H	-0.16469	-3.24425	0.15285
	H	-0.33837	-3.93786	1.78601
	H	3.20115	0.78566	2.43370
	H	0.28703	3.33629	-1.15200
	H	1.44069	0.23230	-0.21595
	S	2.93304	3.34183	-1.22574

Name	Cat 2S_conf15			
Cartesian Coordinates	N	-0.82573	-3.20747	0.85321
	N	0.50616	-1.37618	0.45518
	N	-3.45507	-0.93627	-0.48557
	N	-1.74693	0.60111	-0.05593
	N	-4.43224	3.78839	-0.73434
	C	-2.06692	-2.43298	0.88825
	C	-3.10102	-3.24400	-1.30374
	C	-3.71698	-4.40041	0.85101
	C	-4.22000	-3.95496	-0.52846
	C	-3.16598	-3.20164	1.63751
	C	-2.50636	-2.04233	-0.55328
	C	0.42988	-2.71707	0.70200
	C	1.67916	-0.60026	0.31691
	C	3.83832	1.15901	-0.04636
	C	1.73734	0.60291	1.02006
	C	2.71850	-0.93900	-0.55945
	C	3.79148	-0.06598	-0.71886
	C	2.79918	1.48653	0.81709
	C	2.75081	2.81729	1.51211
	C	4.93944	-0.44905	-1.61442
	C	-3.03709	0.37839	-0.38972
	C	-2.19888	2.98116	-0.18251
	C	-3.96378	1.40895	-0.62263
	C	-1.37497	1.89564	0.02710
	C	-3.56251	2.75204	-0.51904
	C	-3.96977	5.16583	-0.62802
	C	-5.81769	3.51356	-1.08273
	F	2.50435	2.68383	2.83498
	F	1.74606	3.58833	1.01275
	F	3.89469	3.51716	1.37560
	F	5.44649	0.62808	-2.26014
	F	5.95770	-0.99530	-0.91047
	F	4.57571	-1.34994	-2.55226
	H	-1.87390	-1.50909	1.44350
	H	-3.44846	-2.91538	-2.29137
	H	-2.28445	-3.95513	-1.48091
	H	-2.93930	-5.16766	0.72140
	H	-4.52275	-4.87544	1.42264
	H	-4.57924	-4.81544	-1.10486
	H	-5.08178	-3.28417	-0.39923
	H	-3.98175	-2.49241	1.82150
	H	-2.78407	-3.51097	2.61763
	H	-1.61815	-1.70939	-1.10290
	H	4.66306	1.84330	-0.20326
	H	0.94660	0.85051	1.72024
	H	2.67662	-1.86623	-1.11367
	H	-1.79081	3.97854	-0.08785
	H	-4.98141	1.14261	-0.88182

	H	-0.33341	2.07431	0.27752
	H	-4.80230	5.83999	-0.83217
	H	-3.59205	5.38873	0.37840
	H	-3.17203	5.38251	-1.35062
	H	-5.89423	2.95241	-2.02430
	H	-6.32293	2.93500	-0.29748
	H	-6.35341	4.45544	-1.20592
	H	-4.33820	-1.07409	-0.95653
	H	-0.35235	-0.80881	0.38702
	H	-0.85934	-4.19535	1.06305
	S	1.76743	-3.75514	0.85069

Name	Cat 2S_conf16			
Cartesian Coordinates	N	0.86805	-3.20381	-0.86339
	N	-0.49080	-1.39119	-0.47211
	N	3.45765	-0.89302	0.48546
	N	1.73024	0.61792	0.03831
	N	4.35913	3.84620	0.74320
	C	2.09820	-2.41172	-0.89318
	C	3.13373	-3.20484	1.30470
	C	3.77721	-4.35450	-0.84554
	C	4.26689	-3.90008	0.53578
	C	3.21196	-3.16507	-1.63612
	C	2.52493	-2.01300	0.54993
	C	-0.39485	-2.73120	-0.71597
	C	-1.67481	-0.63138	-0.33620
	C	-3.86616	1.09151	0.00882
	C	-2.70848	-0.98076	0.54226
	C	-1.75273	0.56569	-1.04807
	C	-2.83116	1.43108	-0.85509
	C	-3.79706	-0.12558	0.69319
	C	-4.87596	-0.46604	1.68639
	C	-2.81224	2.75116	-1.57160
	C	3.02013	0.41510	0.38545
	C	2.14444	3.00459	0.16780
	C	3.92814	1.45980	0.62820
	C	1.33945	1.90653	-0.04986
	C	3.50766	2.79660	0.51932
	C	5.74169	3.59246	1.11811
	C	3.87370	5.21628	0.64405
	F	-6.08470	-0.00764	1.28811
	F	-4.62657	0.09502	2.89574
	F	-4.98599	-1.79567	1.88457
	F	-1.81674	3.54655	-1.09306
	F	-2.57510	2.60168	-2.89454
	F	-3.96700	3.43308	-1.43576
	H	1.89470	-1.49141	-1.45057
	H	3.47158	-2.86996	2.29357
	H	2.32697	-3.92780	1.47910
	H	4.59275	-4.81791	-1.41283
	H	3.01066	-5.13329	-0.71868
	H	4.63583	-4.75455	1.11499
	H	5.11936	-3.21681	0.41013
	H	4.01805	-2.44417	-1.81750
	H	2.83930	-3.48140	-2.61758
	H	1.62931	-1.69277	1.09510
	H	-4.71146	1.75445	0.14661
	H	-2.65660	-1.90846	1.09539
	H	-0.96722	0.81983	-1.75183
	H	1.72212	3.99556	0.06834
	H	4.94650	1.20887	0.89952

	H	0.29804	2.06899	-0.31197
	H	6.26273	4.54229	1.24302
	H	5.80982	3.03879	2.06489
	H	6.26759	3.01548	0.34558
	H	4.69480	5.90314	0.85212
	H	3.49401	5.43759	-0.36180
	H	3.07121	5.41627	1.36644
	H	4.33868	-1.01633	0.96442
	H	0.35929	-0.81105	-0.40408
	H	0.91650	-4.19161	-1.07076
	S	-1.71734	-3.78834	-0.86511

Name	Cat 2S_conf17			
Cartesian Coordinates	N	0.86856	-3.20412	-0.86263
	N	-0.49056	-1.39143	-0.47257
	N	3.45781	-0.89222	0.48493
	N	1.73028	0.61821	0.03671
	N	4.35932	3.84727	0.73794
	C	2.09856	-2.41177	-0.89319
	C	3.13437	-3.20375	1.30492
	C	3.77796	-4.35421	-0.84488
	C	4.26763	-3.89909	0.53621
	C	3.21235	-3.16527	-1.63593
	C	2.52532	-2.01230	0.54970
	C	-0.39443	-2.73156	-0.71569
	C	-1.67467	-0.63180	-0.33645
	C	-3.86612	1.09084	0.00896
	C	-2.70789	-0.98106	0.54259
	C	-1.75311	0.56498	-1.04873
	C	-2.83159	1.43026	-0.85555
	C	-3.79653	-0.12601	0.69372
	C	-4.87497	-0.46629	1.68748
	C	-2.81312	2.75020	-1.57234
	C	3.02021	0.41574	0.38387
	C	2.14420	3.00501	0.16494
	C	3.92813	1.46070	0.62601
	C	1.33932	1.90673	-0.05203
	C	3.50763	2.79737	0.51588
	C	3.87097	5.21699	0.64853
	C	5.73926	3.59388	1.12265
	F	-4.98454	-1.79585	1.88637
	F	-6.08399	-0.00844	1.28942
	F	-4.62531	0.09544	2.89648
	F	-1.81841	3.54633	-1.09335
	F	-2.57510	2.60061	-2.89509
	F	-3.96838	3.43141	-1.43729
	H	1.89478	-1.49180	-1.45102
	H	3.47222	-2.86841	2.29364
	H	2.32776	-3.92678	1.47966
	H	4.59358	-4.81766	-1.41203
	H	3.01161	-5.13315	-0.71764
	H	4.63682	-4.75322	1.11576
	H	5.11994	-3.21567	0.41019
	H	4.01827	-2.44431	-1.81781
	H	2.83957	-3.48214	-2.61718
	H	1.62961	-1.69210	1.09475
	H	-4.71147	1.75369	0.14689
	H	-2.65560	-1.90857	1.09601
	H	-0.96798	0.81899	-1.75296
	H	1.72156	3.99586	0.06568
	H	4.94640	1.21002	0.89787

	H	0.29776	2.06893	-0.31374
	H	3.07434	5.41376	1.37843
	H	4.69309	5.90427	0.85116
	H	3.48251	5.44089	-0.35324
	H	5.80180	3.04631	2.07349
	H	6.26803	3.01107	0.35664
	H	6.26128	4.54371	1.24338
	H	4.33883	-1.01520	0.96397
	H	0.35945	-0.81115	-0.40480
	H	0.91714	-4.19190	-1.07004
	S	-1.71679	-3.78891	-0.86440

Name	Cat 2S_conf18			
Cartesian Coordinates	N	1.73779	-1.34944	-0.72234
	N	-0.11781	-0.57980	-1.89634
	N	3.43695	0.90580	-0.14605
	N	2.00445	1.55355	1.58147
	N	-0.38783	3.71477	-1.14422
	C	3.15790	-1.55319	-0.42564
	C	5.22797	-0.62232	0.66418
	C	4.88153	-3.13519	0.51020
	C	5.49145	-1.98437	1.32520
	C	3.38305	-2.91197	0.25047
	C	3.72521	-0.40025	0.44392
	C	1.23920	-0.80611	-1.84692
	C	-1.11564	-0.69297	-0.90679
	C	-3.21434	-0.87955	0.95308
	C	-0.91399	-0.29466	0.42261
	C	-2.37536	-1.16635	-1.30168
	C	-3.41366	-1.24357	-0.37839
	C	-1.96026	-0.41146	1.33971
	C	-1.75231	0.04288	2.76286
	C	-4.77637	-1.69212	-0.83867
	C	2.30991	1.61188	0.27067
	C	0.11129	3.00042	1.13858
	C	1.55618	2.33667	-0.66680
	C	0.93102	2.25835	1.97193
	C	0.41463	3.04358	-0.24779
	C	-1.69612	4.18614	-0.71023
	C	-0.15211	3.53964	-2.57094
	F	-0.49149	-0.18944	3.18497
	F	-2.58971	-0.58944	3.61457
	F	-1.98210	1.37061	2.90030
	F	-5.49208	-2.23900	0.16650
	F	-5.49681	-0.65279	-1.32151
	F	-4.69335	-2.60718	-1.82962
	H	3.67291	-1.54744	-1.39054
	H	5.74739	-0.56959	-0.30406
	H	5.61698	0.19567	1.27991
	H	5.40125	-3.21189	-0.45530
	H	5.03000	-4.09153	1.02546
	H	5.05212	-1.98317	2.33320
	H	6.56931	-2.14111	1.45196
	H	2.97416	-3.70901	-0.38151
	H	2.83565	-2.93876	1.20495
	H	3.22536	-0.41438	1.41757
	H	-4.01673	-0.96848	1.67472
	H	0.03257	0.12713	0.73669
	H	-2.53456	-1.48631	-2.32638
	H	-0.75524	3.49589	1.55426
	H	1.83302	2.27642	-1.71114

	H	0.68939	2.19490	3.03048
	H	-2.19440	4.67962	-1.54626
	H	-2.33935	3.36639	-0.35485
	H	-1.60089	4.91991	0.09778
	H	0.84937	3.88856	-2.84604
	H	-0.24030	2.48775	-2.88369
	H	-0.87673	4.13305	-3.13076
	H	3.55482	0.91491	-1.15449
	H	-0.43623	-0.35308	-2.82978
	H	1.09258	-1.59961	0.01734
	S	2.15361	-0.41048	-3.22571

Name	Cat 2S_conf19
Cartesian Coordinates	N 2.11427 0.16656 0.94758<br>N 0.40069 -0.43473 2.38442<br>N 2.94689 -1.76970 -1.04515<br>N 1.26111 -3.18454 -0.37213<br>N -1.69364 -0.80323 -2.23503<br>C 3.46757 0.20802 0.39831<br>C 4.92455 -0.39409 -1.56770<br>C 5.44763 1.66742 -0.17794<br>C 5.50571 1.02820 -1.57307<br>C 4.02011 1.64085 0.38598<br>C 3.48883 -0.41587 -1.02041<br>C 1.75417 -0.40823 2.11377<br>C -0.66466 0.15233 1.67629<br>C -2.84417 1.27243 0.29325<br>C -1.86095 -0.56567 1.53676<br>C -0.57935 1.44798 1.14887<br>C -1.65641 1.98253 0.44193<br>C -2.93839 0.00330 0.86461<br>C -4.23106 -0.76224 0.77653<br>C -1.48967 3.33328 -0.19978<br>C 1.58442 -2.05153 -1.02264<br>C -1.05811 -2.78706 -0.95498<br>C 0.63746 -1.23765 -1.67318<br>C -0.03723 -3.51535 -0.36558<br>C -0.72642 -1.59099 -1.64106<br>C -2.97945 -1.39301 -2.59526<br>C -1.28230 0.36658 -2.99685<br>F -4.01550 -2.09393 0.62254<br>F -4.98804 -0.35610 -0.26689<br>F -4.97648 -0.61645 1.89321<br>F -0.84459 4.19726 0.61336<br>F -0.74618 3.24695 -1.33587<br>F -2.66935 3.88824 -0.54156<br>H 4.08851 -0.40980 1.05396<br>H 5.55019 -1.04763 -0.94206<br>H 4.92349 -0.82420 -2.57517<br>H 6.11117 1.11583 0.50289<br>H 5.81869 2.69845 -0.21372<br>H 4.93312 1.64716 -2.27932<br>H 6.53826 1.00625 -1.94107<br>H 3.99286 2.04186 1.40538<br>H 3.36245 2.27411 -0.22841<br>H 2.87489 0.20799 -1.68149<br>H -3.67635 1.69473 -0.25476<br>H -1.93242 -1.57410 1.92905<br>H 0.31468 2.04190 1.29992<br>H -2.07798 -3.13328 -0.86375<br>H 0.97062 -0.37751 -2.23730

	H	-0.28152	-4.43620	0.16331
	H	-2.88814	-2.13084	-3.40673
	H	-3.44598	-1.87683	-1.73785
	H	-3.65485	-0.59854	-2.91888
	H	-0.68678	1.03952	-2.37412
	H	-0.70010	0.10841	-3.89539
	H	-2.17176	0.91705	-3.31002
	H	3.44072	-2.41493	-0.43489
	H	0.16183	-1.04421	3.15579
	H	1.37324	0.34083	0.27753
	S	2.82022	-1.08549	3.23744

Name	Cat 2S_conf20			
Cartesian Coordinates	N	2.33291	-1.75237	0.78916
	N	0.31378	-1.24863	-0.18147
	N	2.41816	0.31514	-1.42289
	N	1.36059	2.35931	-1.48241
	N	1.64058	2.78976	2.70079
	C	3.11352	-1.87810	-0.45135
	C	4.48682	-0.72024	-2.24499
	C	5.21306	-2.97830	-1.38296
	C	5.68337	-1.62555	-1.93018
	C	4.31835	-2.78635	-0.15323
	C	3.57037	-0.51141	-1.02611
	C	0.98849	-1.61314	0.95118
	C	-1.05304	-0.90676	-0.27730
	C	-3.74423	-0.15854	-0.56364
	C	-1.38833	0.27802	-0.93854
	C	-2.06705	-1.73495	0.21995
	C	-3.39767	-1.34970	0.07894
	C	-2.72846	0.64373	-1.07395
	C	-3.04299	1.92749	-1.79247
	C	-4.48840	-2.26648	0.56362
	C	1.99358	1.45453	-0.71355
	C	0.88789	3.61750	0.53175
	C	2.12151	1.55160	0.67149
	C	0.82740	3.40860	-0.83573
	C	1.55609	2.66106	1.33969
	C	0.91790	3.86253	3.37099
	C	2.17561	1.69010	3.49720
	F	-2.28587	2.95242	-1.33241
	F	-4.33731	2.28838	-1.65665
	F	-2.79150	1.82827	-3.11964
	F	-4.08268	-3.04295	1.59039
	F	-5.57653	-1.57684	0.97640
	F	-4.90692	-3.10123	-0.42025
	H	2.48078	-2.36480	-1.20629
	H	3.89405	-1.17391	-3.05380
	H	4.82217	0.25995	-2.60405
	H	4.65199	-3.51360	-2.16183
	H	6.07064	-3.60843	-1.12091
	H	6.32739	-1.13407	-1.18739
	H	6.29007	-1.76454	-2.83236
	H	3.95382	-3.75005	0.22028
	H	4.90975	-2.32901	0.65460
	H	4.13667	0.01161	-0.24381
	H	-4.78199	0.13708	-0.65477
	H	-0.61037	0.92786	-1.32787
	H	-1.81483	-2.65948	0.72067
	H	0.40582	4.48808	0.95632
	H	2.59233	0.75135	1.22134

	H	0.29886	4.12436	-1.46209
	H	1.24107	4.84339	3.00305
	H	-0.16938	3.78134	3.22956
	H	1.12905	3.82240	4.44036
	H	1.59773	0.76509	3.36303
	H	3.22119	1.48870	3.23170
	H	2.14818	1.96683	4.55191
	H	2.38377	0.51459	-2.41841
	H	0.90679	-0.79521	-0.88739
	H	2.80279	-2.02532	1.64273
	S	0.30090	-1.84580	2.48237

Name	Cat 1S_conf1
Cartesian Coordinates	N 2.61404 -1.45669 0.87591<br>N 0.60842 -0.49516 0.37881<br>N 2.53630 1.50637 0.13167<br>C 3.43181 -0.81798 -0.18013<br>C 4.87227 1.21516 -0.80141<br>C 5.76656 -1.08278 -1.23584<br>C 6.12951 0.34022 -0.79982<br>C 4.71127 -1.67301 -0.29700<br>C 3.76769 0.66615 0.12593<br>C 1.25217 -1.55254 0.95086<br>C -0.76383 -0.27609 0.20665<br>C -3.46658 0.35277 -0.27967<br>C -1.22087 1.04432 0.30824<br>C -1.67182 -1.28655 -0.14386<br>C -3.00671 -0.96238 -0.37368<br>C -2.55698 1.35125 0.05814<br>C -3.01314 2.77565 0.21608<br>C -3.98839 -2.05853 -0.69467<br>F -3.26220 3.08038 1.51134<br>F -4.14482 3.02782 -0.47561<br>F -2.06920 3.64964 -0.21222<br>F -4.97613 -1.62038 -1.51019<br>F -3.39657 -3.10939 -1.30321<br>F -4.58587 -2.53215 0.42308<br>H 2.87633 -0.86980 -1.12670<br>H 4.49773 1.26547 -1.83119<br>H 5.10910 2.24322 -0.50190<br>H 5.38233 -1.06393 -2.26560<br>H 6.65139 -1.72981 -1.24062<br>H 6.57167 0.31625 0.20645<br>H 6.88404 0.77049 -1.46875<br>H 4.42788 -2.68449 -0.61071<br>H 5.15212 -1.76060 0.70764<br>H 4.14813 0.67879 1.15425<br>H -4.50322 0.59254 -0.48119<br>H -0.53053 1.83433 0.58498<br>H -1.33225 -2.30791 -0.24305<br>H 1.21818 0.35098 0.31838<br>H 3.06770 -2.22439 1.35495<br>S 0.53162 -2.85037 1.76439<br>C 2.58570 2.58133 1.12597<br>C 2.12569 2.01915 -1.18322<br>H 1.62318 3.10365 1.14301<br>H 2.76081 2.15663 2.11841<br>H 3.37078 3.32600 0.91437<br>H 1.11439 2.43103 -1.10208<br>H 2.78475 2.81131 -1.56746<br>H 2.09517 1.20446 -1.91241

Name	Cat 1S_conf2			
Cartesian Coordinates	N	1.69331	0.96639	-0.22216
	N	-0.18763	2.20197	-0.73244
	N	2.02337	-0.79632	1.76537
	C	3.09274	0.64428	0.04320
	C	4.49788	-1.13680	1.15678
	C	5.40328	0.15700	-0.83293
	C	5.42885	-1.17502	-0.06689
	C	3.97309	0.57261	-1.21287
	C	3.07260	-0.74538	0.73141
	C	1.19282	2.14566	-0.63214
	C	-1.14360	1.18276	-0.57406
	C	-3.12856	-0.78991	-0.22463
	C	-0.90317	-0.14609	-0.95519
	C	-2.40114	1.51996	-0.04920
	C	-3.37912	0.54206	0.10905
	C	-1.88233	-1.11655	-0.75380
	C	-1.58947	-2.52940	-1.18529
	C	-4.69934	0.90698	0.73659
	F	-1.80457	-2.70228	-2.50840
	F	-2.35633	-3.42714	-0.53327
	F	-0.29186	-2.85934	-0.95766
	F	-4.67493	0.72093	2.07724
	F	-5.01947	2.20160	0.52262
	F	-5.70707	0.14891	0.25232
	H	3.51452	1.40079	0.72089
	H	4.87914	-0.40838	1.88461
	H	4.48163	-2.11201	1.65820
	H	5.85009	0.94381	-0.20827
	H	6.02069	0.08768	-1.73629
	H	5.10520	-1.98396	-0.73777
	H	6.45196	-1.41633	0.24596
	H	3.97317	1.54144	-1.71751
	H	3.53649	-0.15988	-1.90624
	H	2.74634	-1.45955	-0.03840
	H	-3.88610	-1.54977	-0.07738
	H	0.04220	-0.42429	-1.40151
	H	-2.60896	2.54484	0.24086
	H	-0.53820	3.14254	-0.86116
	H	1.09746	0.37968	0.37224
	S	2.09177	3.53050	-0.99503
	C	2.34796	-0.08617	3.00089
	C	1.52688	-2.14591	2.02121
	H	2.68921	0.92795	2.77366
	H	1.44574	-0.00750	3.61633
	H	3.12716	-0.58709	3.59983
	H	1.14934	-2.58263	1.09149
	H	2.28921	-2.82184	2.44698
	H	0.69385	-2.09221	2.73030

Name	Cat 1S_conf3
Cartesian Coordinates	N 1.78979 -1.27565 0.18212<br>N -0.09242 -2.19241 -0.79463<br>N 2.55508 1.15844 -1.04639<br>C 3.19334 -0.86661 0.26850<br>C 2.75484 1.35952 1.43691<br>C 3.27102 -0.72001 2.81118<br>C 3.41039 0.80261 2.71019<br>C 3.84942 -1.38558 1.55888<br>C 3.27682 0.68087 0.15111<br>C 1.27929 -2.21219 -0.63652<br>C -1.00582 -1.15981 -0.50277<br>C -2.90456 0.83146 0.09003<br>C -2.31097 -1.51161 -0.12452<br>C -0.66359 0.19604 -0.61677<br>C -1.61045 1.16976 -0.29391<br>C -3.24769 -0.52099 0.15339<br>C -4.66340 -0.90630 0.49514<br>C -1.17564 2.61019 -0.34603<br>F -5.21695 -0.04033 1.37218<br>F -4.73430 -2.14111 1.03873<br>F -5.45115 -0.91127 -0.60523<br>F -0.27850 2.88444 0.63899<br>F -0.56499 2.90448 -1.51590<br>F -2.20412 3.46794 -0.19523<br>H 3.70025 -1.33472 -0.57493<br>H 2.94360 2.43549 1.38916<br>H 1.66153 1.25332 1.49021<br>H 3.77919 -1.09515 3.70719<br>H 2.21057 -0.99091 2.91851<br>H 2.97240 1.29283 3.58784<br>H 4.47853 1.06383 2.70505<br>H 4.92687 -1.17888 1.49948<br>H 3.73133 -2.47401 1.58899<br>H 4.35736 0.91731 0.06538<br>H -3.62927 1.59735 0.33599<br>H -2.58425 -2.55769 -0.03419<br>H 0.33099 0.50163 -0.94224<br>H -0.46485 -3.03296 -1.21703<br>H 1.13394 -0.67931 0.66878<br>S 2.18731 -3.40096 -1.42694<br>C 2.57624 2.61881 -1.17517<br>C 3.05548 0.54723 -2.28238<br>H 2.08962 2.89622 -2.11368<br>H 2.00837 3.08861 -0.37220<br>H 3.60213 3.03249 -1.18097<br>H 4.13593 0.72614 -2.43919<br>H 2.87917 -0.53032 -2.29224<br>H 2.51441 0.97366 -3.13201

Name	Cat 1S_conf4			
Cartesian Coordinates	N	-2.70393	-1.37140	0.13082
	N	-0.61637	-0.47266	-0.08162
	N	-2.53760	1.60746	0.16759
	C	-3.31560	-0.51498	-0.90102
	C	-4.93730	0.80503	0.57388
	C	-5.73815	-1.29541	-0.58813
	C	-6.12590	0.10983	-0.10820
	C	-4.52424	-1.23547	-1.52741
	C	-3.72524	0.88275	-0.37036
	C	-1.36865	-1.52351	0.37005
	C	0.77053	-0.27547	-0.06022
	C	3.52144	0.33621	-0.10934
	C	1.21128	1.05593	0.00251
	C	1.71930	-1.30137	-0.16870
	C	3.07711	-0.98309	-0.18323
	C	2.57030	1.35228	-0.02417
	C	3.01409	2.78946	-0.02507
	C	4.08498	-2.10174	-0.22699
	F	4.19533	2.95196	0.60933
	F	2.11152	3.59878	0.57750
	F	3.17775	3.25802	-1.28606
	F	5.25070	-1.70610	-0.78887
	F	4.38009	-2.55221	1.01439
	F	3.63234	-3.16080	-0.93360
	H	-2.55998	-0.36609	-1.68150
	H	-4.66930	0.24271	1.47655
	H	-5.21753	1.81492	0.89613
	H	-5.51884	-1.92897	0.28404
	H	-6.57832	-1.77215	-1.10612
	H	-6.45727	0.70860	-0.96898
	H	-6.97369	0.05693	0.58478
	H	-4.21913	-2.23775	-1.85018
	H	-4.80432	-0.68248	-2.43354
	H	-4.04363	1.44056	-1.26039
	H	4.57975	0.56628	-0.12309
	H	0.48702	1.86024	0.07815
	H	1.40063	-2.33126	-0.23935
	H	-1.16522	0.40056	-0.20329
	H	-3.25593	-2.14559	0.47580
	S	-0.80885	-2.88094	1.21132
	C	-2.37923	1.56535	1.62920
	C	-2.45183	2.98583	-0.32181
	H	-1.39052	1.96047	1.88492
	H	-3.13377	2.16341	2.16234
	H	-2.43183	0.53502	1.98426
	H	-1.52110	3.44130	0.03367
	H	-2.43642	2.99311	-1.41583
	H	-3.29050	3.61476	0.02279

Name	Cat 1S_conf5			
Cartesian Coordinates	N	-1.78536	-1.27767	0.17549
	N	0.09218	-2.18468	-0.81924
	N	-2.55748	1.16854	-1.02659
	C	-3.18837	-0.86931	0.27256
	C	-2.74297	1.34520	1.45981
	C	-3.25435	-0.74664	2.81690
	C	-3.39314	0.77699	2.73082
	C	-3.83872	-1.40022	1.56107
	C	-3.27231	0.67937	0.17035
	C	-1.27865	-2.20563	-0.65521
	C	1.00730	-1.15287	-0.52968
	C	2.91742	0.83651	0.03634
	C	0.66049	0.20392	-0.62134
	C	2.32119	-1.50534	-0.18534
	C	3.26314	-0.51559	0.07950
	C	1.61327	1.17580	-0.31203
	C	1.17741	2.61637	-0.35218
	C	4.65342	-0.91364	0.50173
	F	0.55860	2.91850	-1.51585
	F	2.20642	3.47378	-0.20255
	F	0.28674	2.88293	0.64062
	F	5.56511	0.02538	0.16903
	F	4.73145	-1.08426	1.84249
	F	5.03639	-2.07816	-0.06646
	H	-3.69919	-1.32909	-0.57307
	H	-1.64956	1.23673	1.50651
	H	-2.93033	2.42186	1.42329
	H	-3.75848	-1.12985	3.71176
	H	-2.19356	-1.01900	2.91697
	H	-4.46112	1.03902	2.73285
	H	-2.95094	1.25864	3.61107
	H	-3.72108	-2.48894	1.58034
	H	-4.91630	-1.19242	1.50842
	H	-4.35334	0.91643	0.09305
	H	3.65078	1.60171	0.25680
	H	-0.33972	0.51185	-0.92719
	H	2.60244	-2.55174	-0.12477
	H	0.46128	-3.01987	-1.25506
	H	-1.12726	-0.68956	0.66906
	S	-2.19057	-3.38606	-1.45381
	C	-2.58163	2.62992	-1.14263
	C	-3.06304	0.56748	-2.26536
	H	-2.01072	3.09368	-0.33833
	H	-2.09960	2.91617	-2.08085
	H	-3.60819	3.04187	-1.14011
	H	-4.14476	0.74501	-2.41472
	H	-2.52769	1.00312	-3.11393
	H	-2.88423	-0.50950	-2.28626

Name	Cat 1S_conf6			
Cartesian Coordinates	N	-2.74998	-1.35200	-0.27690
	N	-0.66147	-0.45359	-0.08717
	N	-2.44496	1.65798	0.36121
	C	-3.37911	-0.61281	0.83795
	C	-4.65821	0.91678	-0.70597
	C	-5.72766	-1.26749	0.08858
	C	-5.96699	0.18016	-0.36352
	C	-4.68353	-1.33996	1.21631
	C	-3.70357	0.86995	0.49510
	C	-1.41590	-1.49731	-0.53564
	C	0.72767	-0.27920	-0.08504
	C	3.48765	0.28154	0.04694
	C	1.19873	1.03820	-0.18468
	C	1.65243	-1.31943	0.08891
	C	3.01403	-1.02719	0.14524
	C	2.56200	1.31055	-0.11297
	C	3.03409	2.72827	-0.28362
	C	3.99275	-2.14254	0.40366
	F	2.12923	3.61799	0.19239
	F	3.23347	3.03630	-1.58672
	F	4.20147	2.95296	0.35757
	F	4.19476	-2.32300	1.73242
	F	3.56347	-3.32161	-0.09190
	F	5.20272	-1.88429	-0.14281
	H	-2.68776	-0.64385	1.68895
	H	-4.89410	1.95143	-0.97154
	H	-4.17916	0.45347	-1.57754
	H	-6.66604	-1.72072	0.42820
	H	-5.39927	-1.86559	-0.77269
	H	-6.63638	0.19839	-1.23132
	H	-6.47982	0.72775	0.44030
	H	-5.08670	-0.86416	2.11889
	H	-4.45978	-2.38053	1.47889
	H	-4.26166	1.27268	1.35798
	H	4.54896	0.49196	0.09589
	H	0.49579	1.85245	-0.32242
	H	1.31018	-2.34125	0.17139
	H	-1.21894	0.41862	0.05842
	H	-3.28894	-2.12022	-0.65361
	S	-0.86543	-2.84547	-1.40069
	C	-2.00859	2.16209	1.67053
	C	-2.45888	2.74293	-0.62433
	H	-1.01090	2.60402	1.58293
	H	-2.69633	2.92562	2.07112
	H	-1.94927	1.34043	2.38981
	H	-2.66789	2.35099	-1.62116
	H	-3.19121	3.53211	-0.38729
	H	-1.46645	3.20464	-0.64731

Name	Cat 1S_conf7			
Cartesian Coordinates	N	2.70825	-1.37197	-0.14616
	N	0.61624	-0.48240	0.05792
	N	2.53092	1.60700	-0.15143
	C	3.30903	-0.52427	0.89946
	C	4.93601	0.81707	-0.54979
	C	5.73634	-1.29286	0.59514
	C	6.12237	0.11885	0.13315
	C	4.51587	-1.24702	1.52675
	C	3.71741	0.88060	0.38694
	C	1.37489	-1.52733	-0.39579
	C	-0.77034	-0.28433	0.02970
	C	-3.52036	0.33284	0.05290
	C	-1.72269	-1.31042	0.10176
	C	-1.20769	1.04921	-0.00981
	C	-2.56623	1.34826	0.00227
	C	-3.07966	-0.98885	0.10543
	C	-4.09143	-2.09604	0.24567
	C	-3.00667	2.78653	0.01586
	F	-5.27142	-1.77447	-0.33182
	F	-4.35278	-2.36001	1.54948
	F	-3.66491	-3.24908	-0.31040
	F	-2.09245	3.60211	-0.56033
	F	-4.17685	2.96086	-0.63557
	F	-3.18978	3.23773	1.28040
	H	2.54730	-0.38638	1.67607
	H	4.67586	0.26320	-1.45992
	H	5.21486	1.83122	-0.85955
	H	5.52492	-1.91761	-0.28528
	H	6.57462	-1.77236	1.11365
	H	6.44533	0.70945	1.00271
	H	6.97525	0.07658	-0.55429
	H	4.21212	-2.25367	1.83696
	H	4.78784	-0.70263	2.44048
	H	4.02761	1.42980	1.28518
	H	-4.57850	0.56417	0.04246
	H	-1.40713	-2.34310	0.13819
	H	-0.48087	1.85305	-0.06135
	H	1.16269	0.39027	0.19446
	H	3.26469	-2.14270	-0.49184
	S	0.82669	-2.88119	-1.25033
	C	2.38317	1.58056	-1.61454
	C	2.43666	2.97963	0.35247
	H	1.39514	1.97566	-1.87292
	H	3.13973	2.18669	-2.13549
	H	2.44145	0.55434	-1.98051
	H	1.50647	3.43548	-0.00400
	H	2.41419	2.97487	1.44639
	H	3.27508	3.61552	0.02024

Name	Cat 1S_conf8
Cartesian Coordinates	N -2.70775 -1.36327 0.18499<br>N -0.61725 -0.47959 -0.05799<br>N -2.53317 1.61413 0.08621<br>C -3.31356 -0.55247 -0.88686<br>C -4.93565 0.83543 0.52255<br>C -5.73897 -1.31277 -0.54625<br>C -6.12418 0.11384 -0.13202<br>C -4.52200 -1.29813 -1.48362<br>C -3.72141 0.86860 -0.42134<br>C -1.37320 -1.50941 0.43332<br>C 0.76991 -0.28254 -0.03993<br>C 3.52069 0.32667 -0.11205<br>C 1.21071 1.04991 -0.02993<br>C 1.71891 -1.31258 -0.10383<br>C 3.07619 -0.99493 -0.13268<br>C 2.57004 1.34515 -0.06666<br>C 3.01513 2.77963 0.01690<br>C 4.08419 -2.10501 -0.27614<br>F 2.09189 3.62493 -0.50000<br>F 4.17359 2.98674 -0.64654<br>F 3.22154 3.16411 1.29887<br>F 4.33638 -2.37427 -1.58075<br>F 5.26890 -1.78335 0.29180<br>F 3.65931 -3.25487 0.28738<br>H -2.55475 -0.44043 -1.67050<br>H -4.67086 0.31225 1.44929<br>H -5.21427 1.85905 0.79953<br>H -5.52373 -1.90637 0.35453<br>H -6.57873 -1.81058 -1.04469<br>H -6.44969 0.67421 -1.02043<br>H -6.97491 0.09521 0.55911<br>H -4.21807 -2.31469 -1.75936<br>H -4.79838 -0.78671 -2.41479<br>H -4.03690 1.38613 -1.33648<br>H 4.57911 0.55568 -0.13552<br>H 0.48616 1.85699 0.00116<br>H 1.40018 -2.34481 -0.12203<br>H -1.16415 0.38854 -0.21970<br>H -3.26173 -2.12254 0.55884<br>S -0.81921 -2.83377 1.32943<br>C -2.38022 1.64056 1.54870<br>C -2.43995 2.96759 -0.46734<br>H -2.43512 0.62824 1.95217<br>H -1.39196 2.04629 1.78915<br>H -3.13582 2.26398 2.05029<br>H -1.50551 3.43344 -0.13590<br>H -2.42650 2.92349 -1.56047<br>H -3.27409 3.61730 -0.15133

Name	Cat 1S_conf9			
Cartesian Coordinates	N	1.85234	-0.94423	-0.30650
	N	-0.00071	-1.99851	-1.18309
	N	2.39054	-0.37964	2.28544
	C	3.26375	-0.67739	-0.01117
	C	2.84325	1.66416	0.93458
	C	3.49482	1.38840	-1.49940
	C	3.60265	2.27010	-0.25344
	C	4.00062	-0.02189	-1.19027
	C	3.27564	0.21781	1.26104
	C	1.37779	-1.93018	-1.09329
	C	-0.96069	-1.04343	-0.79774
	C	-2.93961	0.81426	-0.04447
	C	-2.21531	-1.48305	-0.35362
	C	-0.71509	0.33470	-0.88949
	C	-1.69195	1.24405	-0.49429
	C	-3.19222	-0.55657	0.00620
	C	-4.51124	-1.04872	0.54309
	C	-1.40571	2.71646	-0.63167
	F	-4.86031	-2.23674	0.00242
	F	-5.51024	-0.17475	0.29466
	F	-4.46280	-1.22419	1.88451
	F	-0.09555	2.99227	-0.41458
	F	-1.69772	3.16695	-1.87231
	F	-2.12618	3.45257	0.24013
	H	3.76076	-1.63070	0.19878
	H	1.76548	1.68281	0.72491
	H	3.00107	2.29983	1.81099
	H	2.44836	1.34253	-1.83149
	H	4.06819	1.81817	-2.32929
	H	4.66294	2.38852	0.01331
	H	3.21293	3.27583	-0.45149
	H	3.91702	-0.67841	-2.06047
	H	5.06639	0.02170	-0.92518
	H	4.32404	0.24334	1.61632
	H	-3.69674	1.52787	0.25640
	H	-2.42434	-2.54574	-0.28630
	H	0.23403	0.69287	-1.26499
	H	-0.33916	-2.85102	-1.61036
	H	1.24593	-0.63137	0.45315
	S	2.31588	-3.06532	-1.92072
	C	2.20712	0.46158	3.47008
	C	2.82611	-1.72079	2.68712
	H	3.16113	0.71907	3.96630
	H	1.68767	1.38745	3.21700
	H	1.58522	-0.07650	4.19150
	H	3.85695	-1.73299	3.08628
	H	2.15511	-2.09635	3.46512
	H	2.77335	-2.41723	1.84707

Name	Cat 1S_conf10			
Cartesian Coordinates	N	2.03952	-1.21950	-0.66438
	N	-0.04477	-2.19451	-1.12123
	N	3.84203	0.71074	-0.04847
	C	1.78469	-0.52389	0.60231
	C	2.91253	0.92097	2.33165
	C	0.98970	-0.59574	2.99168
	C	2.28792	0.07986	3.45580
	C	1.20252	-1.41745	1.71092
	C	3.14005	0.06555	1.07011
	C	1.31759	-2.12213	-1.35618
	C	-0.92434	-1.12674	-0.86047
	C	-2.70162	0.97138	-0.25950
	C	-2.14655	-1.39233	-0.22874
	C	-0.61799	0.18796	-1.22945
	C	-1.49057	1.22303	-0.89921
	C	-3.02699	-0.34983	0.05178
	C	-4.30576	-0.65907	0.78573
	C	-1.04601	2.63047	-1.18810
	F	-4.08737	-0.79791	2.11494
	F	-4.85719	-1.81624	0.35724
	F	-5.22585	0.31620	0.63287
	F	-2.04554	3.52503	-1.07170
	F	-0.06659	3.00563	-0.31922
	F	-0.52293	2.74910	-2.42736
	H	1.08910	0.30960	0.43922
	H	3.86562	1.35135	2.66042
	H	2.24885	1.76191	2.08935
	H	0.59014	-1.24150	3.78229
	H	0.22719	0.17288	2.79877
	H	2.09812	0.70650	4.33546
	H	3.00500	-0.69307	3.76780
	H	1.89908	-2.24598	1.89626
	H	0.25696	-1.86222	1.39191
	H	3.76933	-0.79049	1.35229
	H	-3.37889	1.77996	-0.01624
	H	-2.39995	-2.41052	0.04902
	H	0.30019	0.40000	-1.76493
	H	-0.46550	-3.03764	-1.49274
	H	2.97765	-1.04443	-1.01825
	S	2.02593	-3.17304	-2.47826
	C	5.28163	0.82622	0.16292
	C	3.26684	1.97997	-0.49171
	H	5.69892	-0.15196	0.42301
	H	5.75565	1.16204	-0.76565
	H	5.55392	1.54337	0.95822
	H	2.18757	1.89041	-0.63728
	H	3.44250	2.80859	0.21605
	H	3.71360	2.25718	-1.45229

Name	Cat 1S_conf11			
Cartesian Coordinates	N	-1.67773	1.18144	-0.00536
	N	0.25196	2.28370	-0.66469
	N	-2.07132	-1.23979	1.00448
	C	-3.08515	0.94944	0.29345
	C	-4.56130	-0.74781	1.52643
	C	-5.37054	0.36510	-0.60723
	C	-5.40530	-0.90782	0.24943
	C	-3.93036	0.74468	-0.97496
	C	-3.11799	-0.23280	1.31115
	C	-1.11543	2.31428	-0.43664
	C	1.16776	1.22988	-0.50066
	C	3.07980	-0.81241	-0.15692
	C	2.43580	1.51936	0.02533
	C	0.87725	-0.08909	-0.88503
	C	1.82071	-1.09430	-0.68413
	C	3.38025	0.50716	0.17852
	C	4.75812	0.85735	0.67816
	C	1.45775	-2.52204	-0.99309
	F	5.36153	-0.19631	1.26885
	F	4.72257	1.86641	1.57633
	F	5.55976	1.25914	-0.33509
	F	1.01130	-3.16578	0.11932
	F	0.47169	-2.61143	-1.91280
	F	2.51325	-3.22560	-1.44986
	H	-3.48082	1.83335	0.80770
	H	-4.52530	-1.68817	2.09061
	H	-5.06768	-0.02140	2.17684
	H	-5.96678	0.23002	-1.51733
	H	-5.83107	1.19397	-0.04982
	H	-6.43784	-1.14600	0.53378
	H	-5.04759	-1.75371	-0.34845
	H	-3.48329	-0.03399	-1.60418
	H	-3.90493	1.67326	-1.55375
	H	-2.78850	0.20072	2.26173
	H	3.81033	-1.59917	-0.01473
	H	2.67511	2.53379	0.32788
	H	-0.06517	-0.33075	-1.35889
	H	0.64198	3.20736	-0.80268
	H	-1.12331	0.35721	0.23992
	S	-1.94302	3.77217	-0.69340
	C	-1.51476	-1.86423	2.20455
	C	-2.39830	-2.25916	0.00598
	H	-0.64770	-2.46756	1.91904
	H	-1.17744	-1.09345	2.90456
	H	-2.23393	-2.51924	2.72812
	H	-1.49576	-2.83245	-0.21986
	H	-3.17457	-2.96511	0.34617
	H	-2.73848	-1.79428	-0.92012

Name	Cat 1S_conf12
Cartesian Coordinates	N -2.03947 -1.21958 -0.66451<br>N 0.04494 -2.19414 -1.12178<br>N -3.84194 0.71053 -0.04809<br>C -1.78454 -0.52421 0.60228<br>C -2.91229 0.92023 2.33202<br>C -0.98937 -0.59659 2.99156<br>C -2.28757 0.07889 3.45593<br>C -1.20228 -1.41801 1.71064<br>C -3.13988 0.06510 1.07028<br>C -1.31744 -2.12179 -1.35676<br>C 0.92438 -1.12633 -0.86067<br>C 2.70140 0.97186 -0.25911<br>C 0.61795 0.18842 -1.22943<br>C 2.14650 -1.39188 -0.22883<br>C 3.02683 -0.34933 0.05200<br>C 1.49038 1.22352 -0.89892<br>C 1.04572 2.63092 -1.18769<br>C 4.30559 -0.65917 0.78566<br>F 0.52328 2.74975 -2.42723<br>F 2.04507 3.52563 -1.07060<br>F 0.06580 3.00576 -0.31929<br>F 4.85984 -1.81314 0.35209<br>F 5.22378 0.31871 0.63842<br>F 4.08621 -0.80508 2.11396<br>H -1.08900 0.30933 0.43932<br>H -3.86538 1.35050 2.66096<br>H -2.24867 1.76125 2.08986<br>H -0.22688 0.17209 2.79875<br>H -0.58973 -1.24249 3.78201<br>H -3.00460 -0.69413 3.76784<br>H -2.09770 0.70534 4.33572<br>H -0.25675 -1.86271 1.39145<br>H -1.89880 -2.24660 1.89585<br>H -3.76915 -0.79100 1.35231<br>H 3.37851 1.78048 -0.01555<br>H -0.30020 0.40046 -1.76499<br>H 2.39997 -2.41010 0.04884<br>H 0.46579 -3.03702 -1.49374<br>H -2.97762 -1.04440 -1.01831<br>S -2.02562 -3.17224 -2.47935<br>C -3.26690 1.97995 -0.49096<br>C -5.28154 0.82579 0.16334<br>H -3.71385 2.25748 -1.45137<br>H -3.44247 2.80832 0.21711<br>H -2.18766 1.89051 -0.63677<br>H -5.75562 1.16172 -0.76516<br>H -5.69870 -0.15251 0.42323<br>H -5.55393 1.54273 0.95879

Name	Cat 1S_conf13			
Cartesian Coordinates	N	-1.69169	-1.06358	-0.39819
	N	0.26244	-2.10047	-1.11162
	N	-2.28931	1.50752	0.10188
	C	-3.11332	-0.86820	-0.14185
	C	-4.82999	1.01023	0.14349
	C	-5.00033	-1.10918	1.52554
	C	-5.22829	0.40812	1.50222
	C	-3.53626	-1.44847	1.21926
	C	-3.40913	0.64673	-0.35281
	C	-1.11219	-2.14169	-0.93593
	C	1.19270	-1.11944	-0.72187
	C	3.13586	0.76299	0.06890
	C	0.97395	0.25243	-0.91761
	C	2.41017	-1.53954	-0.16377
	C	3.37006	-0.60491	0.21282
	C	1.93145	1.17506	-0.49574
	C	1.63218	2.64502	-0.61887
	C	4.64522	-1.08057	0.86010
	F	0.85289	2.91534	-1.68942
	F	2.75266	3.38637	-0.72825
	F	0.96447	3.10119	0.47520
	F	5.03877	-2.27622	0.36932
	F	5.65759	-0.20667	0.67487
	F	4.49088	-1.23328	2.19585
	H	-3.67078	-1.41233	-0.91128
	H	-4.94007	2.10156	0.15618
	H	-5.53968	0.64178	-0.61016
	H	-5.28564	-1.52618	2.49860
	H	-5.64774	-1.58750	0.77601
	H	-6.28328	0.63681	1.69828
	H	-4.65558	0.87142	2.31419
	H	-2.88811	-1.06066	2.01417
	H	-3.38152	-2.53215	1.18411
	H	-3.40575	0.78490	-1.43983
	H	3.88253	1.48654	0.37158
	H	0.08127	0.60752	-1.41714
	H	2.60281	-2.59856	-0.02487
	H	0.64175	-3.00746	-1.35217
	H	-1.15793	-0.23157	-0.15339
	S	-1.93771	-3.54839	-1.40390
	C	-2.22474	2.75787	-0.65742
	C	-2.18994	1.78362	1.53605
	H	-1.33216	3.31331	-0.36350
	H	-2.15112	2.54237	-1.72814
	H	-3.10242	3.40767	-0.49299
	H	-2.99178	2.44189	1.91137
	H	-2.21164	0.85535	2.10886
	H	-1.22954	2.27413	1.72483

Name	Cat 1S_conf14			
Cartesian Coordinates	N	-2.03409	-1.23327	-0.65474
	N	0.05867	-2.21387	-1.05475
	N	-3.85198	0.70087	-0.10191
	C	-1.80310	-0.51686	0.60488
	C	-2.96878	0.94935	2.29191
	C	-1.05271	-0.54917	3.00942
	C	-2.36210	0.12730	3.43962
	C	-1.23811	-1.39106	1.73751
	C	-3.16891	0.07478	1.03902
	C	-1.29939	-2.14778	-1.31670
	C	0.93239	-1.13870	-0.80519
	C	2.69258	0.97696	-0.21745
	C	2.14436	-1.38743	-0.14630
	C	0.62774	0.16695	-1.20531
	C	1.49082	1.21169	-0.88005
	C	3.01761	-0.33688	0.12567
	C	4.34249	-0.63456	0.77803
	C	1.04023	2.61258	-1.19014
	F	5.27310	-0.99000	-0.13743
	F	4.24734	-1.65588	1.65891
	F	4.82770	0.43568	1.44325
	F	0.51880	2.71045	-2.43187
	F	0.05750	2.99592	-0.32848
	F	2.03533	3.51353	-1.08500
	H	-1.10716	0.31624	0.44098
	H	-2.30424	1.78974	2.05007
	H	-3.92983	1.37995	2.59633
	H	-0.29032	0.22027	2.81908
	H	-0.66498	-1.18120	3.81686
	H	-3.08168	-0.64414	3.74957
	H	-2.19119	0.76738	4.31347
	H	-0.28512	-1.83685	1.44298
	H	-1.93499	-2.21941	1.92244
	H	-3.80022	-0.77948	1.32194
	H	3.35736	1.79434	0.03100
	H	2.38892	-2.39626	0.17004
	H	-0.28427	0.36598	-1.75604
	H	0.48673	-3.06353	-1.40214
	H	-2.96557	-1.06397	-1.02859
	S	-1.98573	-3.21946	-2.43250
	C	-3.27421	1.96670	-0.55164
	C	-5.29600	0.81235	0.07932
	H	-3.46866	2.80434	0.14038
	H	-2.19174	1.88109	-0.67397
	H	-3.70269	2.22782	-1.52500
	H	-5.75305	1.13162	-0.86348
	H	-5.71387	-0.16377	0.34607
	H	-5.58735	1.54032	0.85788

Name	Cat 1S_conf15
Cartesian Coordinates	N -1.58546 -1.17158 -0.67703<br>N 0.16885 -2.05402 0.52949<br>N -2.28962 1.32043 0.56214<br>C -2.90352 -0.56253 -0.93652<br>C -4.73599 0.96232 -0.06530<br>C -5.32222 -0.93741 -1.61041<br>C -5.77889 -0.11278 -0.40021<br>C -3.96786 -1.60198 -1.33349<br>C -3.35193 0.34793 0.23288<br>C -1.20147 -1.94055 0.36135<br>C 1.17314 -1.10215 0.25633<br>C 3.27234 0.71817 -0.17599<br>C 2.48245 -1.56327 0.05435<br>C 0.91486 0.27646 0.24619<br>C 1.96783 1.16489 0.02119<br>C 3.51767 -0.65556 -0.14869<br>C 4.92859 -1.16321 -0.29206<br>C 1.65524 2.63698 -0.01879<br>F 5.54187 -1.25941 0.91056<br>F 5.68190 -0.34196 -1.05549<br>F 4.96560 -2.39249 -0.85264<br>F 0.95974 2.96227 -1.13686<br>F 0.88569 3.01172 1.03389<br>F 2.76361 3.40327 -0.00030<br>H -2.72893 0.07684 -1.81029<br>H -4.66166 1.65028 -0.91822<br>H -5.05558 1.56163 0.79605<br>H -6.06392 -1.70614 -1.85796<br>H -5.24516 -0.27808 -2.48825<br>H -6.75006 0.35716 -0.59871<br>H -5.91685 -0.77791 0.46376<br>H -4.07377 -2.32639 -0.52184<br>H -3.61143 -2.15179 -2.21290<br>H -3.45671 -0.29990 1.10827<br>H 4.07912 1.41974 -0.34636<br>H 2.68421 -2.62937 0.04994<br>H -0.09464 0.64794 0.42614<br>H 0.42896 -2.81327 1.14681<br>H -0.83327 -0.78239 -1.23064<br>S -2.20639 -2.80978 1.40749<br>C -2.28808 1.71371 1.96898<br>C -2.23259 2.49171 -0.31086<br>H -2.24789 0.82035 2.60020<br>H -1.39399 2.31384 2.16915<br>H -3.17132 2.31014 2.26313<br>H -3.07949 3.18599 -0.17264<br>H -1.31078 3.04178 -0.11198<br>H -2.21412 2.18523 -1.36175

Name	Cat 1S_conf16
Cartesian Coordinates	N 2.74805 -1.22343 -1.31638<br>N 0.68687 -0.36740 -0.80967<br>N 2.60827 1.63970 -0.54251<br>C 3.64081 -0.64480 -0.28038<br>C 4.34197 1.17814 1.28697<br>C 5.09890 -1.20026 1.72572<br>C 4.72985 0.14694 2.35803<br>C 3.98608 -1.69850 0.79332<br>C 3.18045 0.65922 0.40872<br>C 1.38867 -1.43687 -1.28087<br>C -0.66600 -0.22332 -0.48661<br>C -3.34131 0.28698 0.24706<br>C -1.17325 1.08656 -0.48152<br>C -1.50971 -1.27850 -0.10952<br>C -2.83113 -1.01058 0.24455<br>C -2.49300 1.33208 -0.11570<br>C -2.98782 2.75128 -0.05249<br>C -3.70372 -2.14806 0.70691<br>F -4.31270 2.83604 -0.29890<br>F -2.35393 3.55026 -0.94191<br>F -2.77982 3.29250 1.17316<br>F -5.01563 -1.89177 0.50306<br>F -3.40957 -3.30357 0.07494<br>F -3.55034 -2.37609 2.03468<br>H 4.56428 -0.41528 -0.82714<br>H 5.21134 1.40351 0.65439<br>H 4.04694 2.11566 1.76910<br>H 6.02714 -1.08748 1.14715<br>H 5.30028 -1.94867 2.50098<br>H 3.88078 0.00682 3.04216<br>H 5.56131 0.52882 2.96201<br>H 4.29082 -2.62814 0.29782<br>H 3.07756 -1.92886 1.36617<br>H 2.35479 0.40263 1.08680<br>H -4.37362 0.47716 0.51322<br>H -0.53654 1.91256 -0.77932<br>H -1.14091 -2.29417 -0.10784<br>H 1.24166 0.51534 -0.83102<br>H 3.20206 -2.00109 -1.78316<br>S 0.74238 -2.86559 -1.91340<br>C 2.06547 2.82910 0.12007<br>C 3.46734 1.99871 -1.67389<br>H 1.45906 3.39208 -0.59720<br>H 1.42068 2.52561 0.95148<br>H 2.84102 3.50647 0.50955<br>H 2.91366 2.67700 -2.32994<br>H 4.39639 2.50485 -1.36593<br>H 3.71719 1.10722 -2.25262

Name	Cat 1S_conf17			
Cartesian Coordinates	N	2.76174	-1.14122	-1.37307
	N	0.69140	-0.32878	-0.83787
	N	2.58219	1.68389	-0.46685
	C	3.64744	-0.59630	-0.31217
	C	4.31334	1.15843	1.34658
	C	5.11094	-1.22460	1.66974
	C	4.71431	0.08193	2.36686
	C	4.01022	-1.69500	0.70902
	C	3.16431	0.66536	0.43767
	C	1.40491	-1.37619	-1.33789
	C	-0.65937	-0.21036	-0.49652
	C	-3.32161	0.25350	0.31018
	C	-1.18111	1.09279	-0.45321
	C	-1.48329	-1.28371	-0.12712
	C	-2.79830	-1.03827	0.26498
	C	-2.49387	1.31576	-0.05002
	C	-3.04170	2.71682	-0.06866
	C	-3.64581	-2.19181	0.73381
	F	-2.07406	3.63853	0.15268
	F	-3.99590	2.89565	0.87122
	F	-3.60342	3.01911	-1.26258
	F	-4.96363	-1.95932	0.53915
	F	-3.33501	-3.34192	0.10029
	F	-3.47983	-2.41583	2.06081
	H	4.56639	-0.32561	-0.84747
	H	5.18163	1.42894	0.73059
	H	4.00074	2.06601	1.87273
	H	6.03899	-1.06579	1.10174
	H	5.32333	-2.00722	2.40737
	H	3.86449	-0.10816	3.03796
	H	5.53556	0.44779	2.99431
	H	4.33340	-2.59291	0.16865
	H	3.10464	-1.97106	1.26624
	H	2.33939	0.36354	1.09778
	H	-4.34464	0.42786	0.61992
	H	-0.55578	1.93297	-0.73416
	H	-1.10221	-2.29457	-0.15317
	H	1.23797	0.55963	-0.82652
	H	3.22397	-1.89780	-1.86591
	S	0.77379	-2.79648	-2.00298
	C	3.44458	2.11719	-1.56946
	C	2.01293	2.82656	0.25427
	H	2.88459	2.81987	-2.19377
	H	4.36175	2.62219	-1.22642
	H	3.71563	1.26275	-2.19308
	H	1.36034	2.46703	1.05688
	H	2.77282	3.49078	0.69405
	H	1.40822	3.42161	-0.43795

Name	Cat 1S_conf18			
Cartesian Coordinates	N	2.73891	-1.22302	-1.31976
	N	0.68625	-0.36701	-0.77912
	N	2.60870	1.63962	-0.53096
	C	3.65088	-0.64429	-0.30037
	C	4.37437	1.17213	1.26504
	C	5.15185	-1.20425	1.67316
	C	4.78708	0.13609	2.32176
	C	4.02344	-1.70241	0.75987
	C	3.19900	0.65437	0.40439
	C	1.38022	-1.43663	-1.26047
	C	-0.66271	-0.22070	-0.44082
	C	-3.31647	0.30059	0.35984
	C	-1.50301	-1.27345	-0.04906
	C	-1.16368	1.09106	-0.42111
	C	-2.47188	1.34261	-0.01975
	C	-2.81399	-1.00011	0.33782
	C	-3.72329	-2.14614	0.69703
	C	-2.99389	2.75330	-0.05369
	F	-4.66804	-1.77463	1.59202
	F	-3.04395	-3.18455	1.23149
	F	-4.37682	-2.61847	-0.38963
	F	-3.55818	3.04949	-1.24797
	F	-3.93814	2.96255	0.88992
	F	-2.00814	3.66016	0.14830
	H	4.56160	-0.40792	-0.86525
	H	5.23082	1.40552	0.61789
	H	4.08422	2.10520	1.75854
	H	6.06760	-1.08168	1.07691
	H	5.37303	-1.95685	2.43889
	H	3.95136	-0.01360	3.02019
	H	5.62724	0.51863	2.91314
	H	4.32340	-2.62716	0.25240
	H	3.12785	-1.94165	1.34919
	H	2.38577	0.39172	1.09504
	H	-4.33211	0.49845	0.67965
	H	-1.13386	-2.28873	-0.04220
	H	-0.52452	1.91677	-0.71378
	H	1.24147	0.51541	-0.80582
	H	3.18429	-2.00151	-1.79344
	S	0.72351	-2.86682	-1.87911
	C	3.44641	2.00567	-1.67606
	C	2.07705	2.82466	0.14863
	H	2.88126	2.68994	-2.31592
	H	4.38230	2.50788	-1.38278
	H	3.68307	1.11827	-2.26657
	H	2.85914	3.50024	0.52803
	H	1.45665	3.39162	-0.55327
	H	1.44739	2.51561	0.98943

Name	Cat 1S_conf19
Cartesian Coordinates	N -2.35252 -0.03423 -0.48364<br>N -0.10388 0.27243 -0.56353<br>N -4.16859 1.93727 -0.05762<br>C -3.64744 -0.49536 0.01564<br>C -6.05009 0.21072 0.27455<br>C -5.45917 -2.24488 -0.00120<br>C -6.51631 -1.15815 -0.24891<br>C -4.08971 -1.84498 -0.57120<br>C -4.69049 0.58896 -0.34555<br>C -1.14610 -0.47510 -0.04898<br>C 1.28274 0.15318 -0.33683<br>C 4.07184 0.04594 0.00272<br>C 1.94336 -1.08268 -0.33191<br>C 2.02509 1.32917 -0.18518<br>C 3.40973 1.27045 -0.02639<br>C 3.32383 -1.12243 -0.14941<br>C 4.01581 -2.45838 -0.07744<br>C 4.18836 2.55482 0.06196<br>F 5.29276 -2.38456 -0.51864<br>F 3.38302 -3.39405 -0.81872<br>F 4.06462 -2.92466 1.19150<br>F 5.37717 2.38708 0.67851<br>F 4.44698 3.06242 -1.16778<br>F 3.50608 3.50743 0.73895<br>H -3.58483 -0.60402 1.10721<br>H -5.95880 0.17542 1.36820<br>H -6.79428 0.98086 0.03995<br>H -5.35973 -2.41492 1.08042<br>H -5.78184 -3.19682 -0.43918<br>H -6.70859 -1.08036 -1.32892<br>H -7.46803 -1.43531 0.22044<br>H -3.33804 -2.60358 -0.34014<br>H -4.14375 -1.75479 -1.66498<br>H -4.80719 0.55989 -1.43933<br>H 5.14590 0.00135 0.13453<br>H 1.38504 -1.99659 -0.47485<br>H 1.52203 2.29121 -0.17613<br>H -0.36825 1.10195 -1.08282<br>H -2.41974 0.94042 -0.78592<br>S -0.94326 -1.77151 1.01285<br>C -3.99132 2.23759 1.36320<br>C -4.90747 2.99655 -0.73868<br>H -3.46535 3.19320 1.46131<br>H -4.94287 2.31699 1.91564<br>H -3.37850 1.46828 1.84121<br>H -5.92962 3.14403 -0.34781<br>H -4.36807 3.94352 -0.62608<br>H -4.97987 2.76961 -1.80764

Name	Cat 1S_conf20			
Cartesian Coordinates	N	-1.57188	-1.18564	-0.64499
	N	0.16465	-2.04235	0.60372
	N	-2.29428	1.33337	0.52936
	C	-2.88563	-0.58279	-0.93736
	C	-4.72947	0.96402	-0.13265
	C	-5.29289	-0.97044	-1.64269
	C	-5.76877	-0.11671	-0.46043
	C	-3.94508	-1.63055	-1.32582
	C	-3.35170	0.35457	0.20352
	C	-1.20309	-1.93154	0.41550
	C	1.17243	-1.09680	0.31968
	C	3.27802	0.71435	-0.11706
	C	0.91586	0.28195	0.28726
	C	2.48255	-1.56263	0.13617
	C	3.52077	-0.65936	-0.07156
	C	1.97260	1.16590	0.06242
	C	1.66653	2.63913	0.01093
	C	4.91143	-1.17731	-0.32937
	F	1.00182	2.96395	-1.12584
	F	2.77731	3.40092	0.05552
	F	0.87193	3.02042	1.04223
	F	5.85627	-0.30375	0.08068
	F	5.13621	-2.35134	0.30057
	F	5.11830	-1.39215	-1.65013
	H	-2.69844	0.03580	-1.82330
	H	-4.63864	1.63172	-0.99998
	H	-5.06285	1.58371	0.70877
	H	-6.03179	-1.74351	-1.88506
	H	-5.19888	-0.33222	-2.53437
	H	-6.73547	0.34998	-0.68642
	H	-5.92257	-0.76135	0.41630
	H	-4.06696	-2.33365	-0.49783
	H	-3.57479	-2.20317	-2.18468
	H	-3.47176	-0.27305	1.09171
	H	4.08977	1.41322	-0.27455
	H	-0.09451	0.65676	0.45487
	H	2.68465	-2.62838	0.16289
	H	0.41720	-2.78797	1.24048
	H	-0.81167	-0.81022	-1.19719
	S	-2.22364	-2.77584	1.46673
	C	-2.21083	2.47716	-0.37733
	C	-2.32805	1.77063	1.92274
	H	-2.16611	2.13912	-1.41766
	H	-3.05851	3.17781	-0.28263
	H	-1.29275	3.03081	-0.17145
	H	-1.43630	2.37237	2.12810
	H	-3.21535	2.38055	2.17397
	H	-2.31024	0.89744	2.58252

1.7. REFERENCES

1. Okino, T.; Hoashi, Y.; Furukawa, T.; Xu, X.; Takemoto, Y. Enantio- and Diastereoselective Michael Reaction of 1,3-Dicarbonyl Compounds to Nitroolefins Catalyzed by a Bifunctional Thiourea. *J. Am. Chem. Soc.* **2005**, *127*, 119–125.
2. Hamza, A.; Schubert, G.; Soós, T., Pápai I. Theoretical studies on the bifunctionality of chiral thiourea-based organocatalysts: Competing routes to C-C bond formation. *J. Am. Chem. Soc.* **2006**, 128, 13151–13160.
3. Okino, T.; Hoashi, Y.; Takemoto, Y. Enantioselective Michael Reaction of Malonates to Nitroolefins Catalyzed by Bifunctional Organocatalysts. *J. Am. Chem. Soc*. **2003**, *125*, 12672–12673.
4. Işık, M.; Tanyeli, C. Cu-Catalyzed Selective Mono-*N*-pyridylation: Direct Access to 2-AminoDMAP/Sulfonamides as Bifunctional Organocatalysts. *J. Org. Chem.* **2013**, *78*, 1604–1611.
5. Işık, M.; Unver, M. Y.; Tanyeli, C. Modularly Evolved 2-AminoDMAP/Squaramides as Highly Active Bifunctional Organocatalysts in Michael Addition. *J. Org. Chem*. **2015**, *80*, 828–835.
6. Izzo, J.A.; Myshchuk, Y.; Hirschi, J.S., Vetticatt, M.J. Tran- sition state analysis of an enantioselective Michael addition by a bifunctional thiourea organocatalyst. Org. Biomol. Chem. 2019, 17, 3934–3939.
7. *Molecular Operating Environment (MOE),* 2022.02 Chemical Computing Group ULC, 910-1010 Sherbrooke St. W., Montreal, QC H3A 2R7, Canada, **2023**.
8. Chen, I.J.; Foloppe, N. Conformational sampling of druglike molecules with MOE and catalyst: Implications for pharmacophore modeling and virtual screening. *J Chem Inf Model* **2008**, *48*, 1773–1791.
9. Zhang L.; Lee M.M; Lee S.M.; Lee J.; Cheng M.; Jeong B.S.; Park H.G.; Jew S.S. Novel cinchona-aminobenzimidazole bifunctional organocatalysts. *Adv. Synth. Catal.* **2009**, 351, 3063-3066.
10. Lai Q.; Li Y.; Gong Z.; Liu Q.; Wei C.; Song Z. Novel chiral bifunctional l-thiazoline-thiourea derivatives: design and application in enantioselective Michael reactions. *Chirality* **2015**, 27, 979-988.
11. Okino T.; Hoashi Y.; Takemoto Y. Enantioselective Michael reaction of malonates to nitroolefins catalyzed by bifunctional organocatalysts. *J. Am. Chem. Soc.* **2003**,125,12672-12673.

12. Bickelhaupt, F.M.; Houk, K.N. Analyzing Reaction Rates with the Distortion / Interaction - Activation Strain Model. *Angewandte Chemie* **2017**, 129, 10204–10221.

13. Gaussian 16, Revision C.01, Frisch, M. J.; Trucks, G. W.; Schlegel, H. B.; Scuseria, G. E.; Robb, M. A.; Cheeseman, J. R.; Scalmani, G.; Barone, V.; Petersson, G. A.; Nakatsuji, H.; Li, X.; Caricato, M.; Marenich, A. V.; Bloino, J.; Janesko, B. G.; Gomperts, R.; Mennucci, B.; Hratchian, H. P.; Ortiz, J. V.; Izmaylov, A. F.; Sonnenberg, J. L.; Wil- liams-Young, D.; Ding, F.; Lipparini, F.; Egidi, F.; Goings, J.; Peng, B.; Petrone, A.; Henderson, T.; Ranasinghe, D.; Zakrzewski, V. G.; Gao, J.; Rega, N.; Zheng, G.; Liang, W.; Hada, M.; Ehara, M.; Toyota, K.; Fukuda, R.; Hasegawa, J.; Ishida, M.; Nakajima, T.; Honda, Y.; Kitao, O.; Nakai, H.; Vreven, T.; Throssell, K.; Montgomery, J. A., Jr.; Peralta, J. E.; Ogliaro, F.; Bearpark, M. J.; Heyd, J. J.; Brothers, E. N.; Kudin, K. N.; Staroverov, V. N.; Keith, T. A.; Kobayashi, R.; Normand, J.; Raghavachari, K.; Rendell, A. P.; Burant, J. C.; Iyengar, S. S.; Tomasi, J.; Cossi, M.; Millam, J. M.; Klene, M.; Adamo, C.; Cammi, R.; Ochterski, J. W.; Martin, R. L.; Morokuma, K.; Farkas, O.; Foresman, J. B.; Fox, D. J. Gaussian, Inc., Wallingford CT, **2016**.

14. Glendening, E. D.; Streitwieser, A. *J. Chem. Phys.* **1994**, *100*, 2900.

15. Glendening, E. D. *J. Am. Chem. Soc.* **1996***, 118*, 2473.

16. Schenter, G. K.; Glendening, E. D. *J. Phys. Chem. A* **1996**, *199*, 17152.

17. Glendening, E. D. *J. Phys. Chem. A* **2005**, *109*, 11936.

18. NBO 7.0, Glendening, E. D.; Badenhoop, J. K.; Reed, A. E.; Carpenter, J. E.; Bohmann, J. A.; Morales, C. M.; Karafiloglou, P.; Landis, C. R.; Weinhold, F. Theoretical Chemistry Institute, University of Wisconsin, Madison, **2018**.

2. EXPERIMENTAL

2.1. GENERAL

The compound 2-aminoDMAP,[1] which serves as the precatalyst for the two catalysts (**2O**/**S**), was synthesized following our previous work. All the chemicals and solvents used in this work were commercially supplied and used without further purification unless specifically noted. 1D NMR spectra were recorded on a Bruker Spectrospin Avance DPX 400 spectrometer using $CDCl_3$ as the solvent. Chemical shifts values are reported in ppm relative to tetramethylsilane as the internal standard. Spin multiplicities were reported as follows: s (singlet), bs (broad singlet), d (doublet), dd (doublet of doublet), ddd (doublet of doublet of doublet), triplet of doublets (td), triplet (t), doublet of triplets (dt), quartet (q), doublet of quartets (dq), heptet (hept), multiplet (m). HRMS data were acquired on an Agilent Technologies 6530 Accurate- Mass Q-TOF LC/MS. Flash grade silica gel (40–63 μm) was used for flash column chromatography (FCC) purifications. Reactions were monitored by thin layer chromatography (TLC) using precoated silica gel plates (Merck Silica Gel PF-254), visualized by a handheld UV-Vis lamp. All organic extracts were dehydrated over either anhydrous Na_2SO_4 or $MgSO_4$ and concentrated by using rotary evaporator before being subjected to FCC. Optical rotation measurements were acquired using a Rudolph Scientific Autopol III polarimeter by employing a 1 dm cell and reported as follows: $[\alpha]^T$ (*c* in g per 100 mL, solvent). Enantiomeric purities of D chiral adducts were determined on a Thermo-Finnigan HPLC system equipped with analytical chiral columns from Daicel Chiral Technologies (Chiralpak AS-H chiral column (0.46 cmØ × 25 cm), Chiralpak AD chiral column (0.46 cmØ × 25 cm), Chiralpak OJ-H chiral column (0.46 cmØ × 25 cm), and Chiralpak IA chiral column (0.46 cmØ × 25 cm). For enantiomer separations, isocratic mixtures of HPLC grade *n*-hexanes and isopropyl alcohol were used.

2.2. CHARACTERIZATION DATA FOR CHIRAL 2-AMINODMAP/(THIO)UREA COMPOUNDS

Data for 2S:

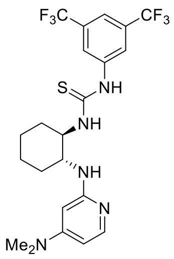

mp: 115-121 ˚C

^{1}H NMR (400 MHz, $CDCl_3$) δ 10.11 (bs, 1H), 8.76 (bs, 1H), 8.00 (s, 2H), 7.46 (s, 1H), 7.38 (d, *J* = 7.5 Hz, 1H), 6.77 (bs, 1H), 6.03 (dd, *J* = 2.4, 7.5Hz, 1H), 5.77 (bs, 1H), 4.47 (bs, 1H), 3.80 (bs, 1H), 2.99 (s, 6H), 2.14 – 2.04 (m, 1H), 2.04 – 1.94 (m, 1H), 1.83 – 1.68 (m, 2H), 1.66 – 1.52 (m, 1H), 1.52 – 1.29 (m, 3H) ppm.

^{13}C NMR (101 MHz, $CDCl_3$) δ 181.5, 156.9, 152.1, 141.2, 135.0, 131.26 (d, *J* = 99.8 Hz), 131.26 (d, *J* = 33.4 Hz), 123.26 (d, *J* = 818.2 Hz), 123.26 (d, *J* = 272.7 Hz), 123.4, 117.4 (d, *J* = 4.0 Hz) 100.0, 87.8, 60.4, 53.8, 39.9, 30.9, 29.7, 29.3, 23.7 ppm.

IR (neat) 2930, 2857, 1609, 1525, 1471, 1377, 1274, 1168, 1126, 884, 700, 680 cm^{-1}.

HRMS calculated for $C_{22}H_{26}F_6N_5S$ $[M + H]^+$ 506.1813, found 506.1800.

$[\alpha]_D^{25}$ = -133.0° (*c* 1.0, $CHCl_3$)

Data for 2O:

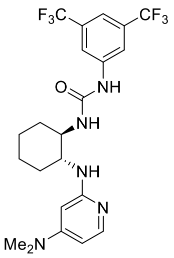

mp: 180-200 ˚C

^{1}H NMR (400 MHz, $CDCl_3$) δ 8.03 (bs, 1H), 7.71 (s, 2H), 7.55 (d, *J* = 6.3 Hz, 1H), 7.28 (s, 1H), 6.59 (bs, 1H), 5.90 (d, *J* = 4.7 Hz, 1H), 5.46 (s, 1H), 4.59 (bs, 1H), 3.62 (bs, 1H), 3.48 (bs, 1H), 2.82 (s, 6H), 2.01 (s, 2H), 1.60 (s, 2H), 1.18 (s, 4H) ppm.

^{13}C NMR (101 MHz, $CDCl_3$) δ 156.9, 153.9, 153.6, 143.7, 139.3, 129.5 (d, *J* = 99.2 Hz), 129.5 (d, *J* = 33.1 Hz), 121.1 (d, *J* = 818.1 Hz), 121.1 (d, *J* = 272.7 Hz), 115.8, 112.5 (d, *J* = 2.1 Hz), 97.8, 86.9, 59.3, 58.8, 36.8, 32.2, 27.5, 22.5, 21.9 ppm.

IR (neat): 2922, 2853, 1610, 1527, 1473, 1389, 1275, 1169, 1125, 878, 702, 681 cm^{-1}.

HRMS: calculated for $C_{22}H_{26}F_6N_5O$ $[M + H]^+$ 490.2042, found 490.2052.

$[\alpha]_D^{25}$ = -35.3° (*c* = 1, $CHCl_3$)

2.3. CHARACTERIZATION DATA FOR CHIRAL CONJUGATE ADDITION PRODUCTS

Data for 5a:

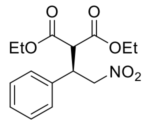

^{1}H NMR (400 MHz, $CDCl_3$) δ 7.36 – 7.05 (m, 5H), 4.86 (dd, J = 13.1, 4.9 Hz, 1H), 4.79 (dd, J = 13.1, 9.1 Hz, 1H), 4.18 – 4.13 (m, 3H), 3.93 (q, J = 7.1 Hz, 2H), 3.75 (d, J = 9.4 Hz, 1H), 1.19 (t, J = 7.2 Hz, 3H), 0.97 (t, J = 7.1 Hz, 3H) ppm.

^{13}C NMR (101 MHz, $CDCl_3$) δ 166.4, 165.8, 135.2, 127.9, 127.3, 127.0, 76.6, 61.1, 60.9, 53.9, 41.9, 12.9, 12.7 ppm.

HPLC (Chiralpak AS-H, 90:10 *n*-hexane/isopropyl alcohol, 1 mL/min, 210 nm): t_{minor} = 19.172, t_{major} = 23.390 min; 94% ee.

$[\alpha]_D^{25}$ = -4.22° (c 1.0, $CHCl_3$).

The absolute configuration was assigned as (*R*) by comparison of the optical rotation with the following literature value.[2] $[\alpha]_D^{25}$ = +7.3° (c 1.07, $CHCl_3$), [95% ee, (*S*)-enantiomer].

Data for 5b:

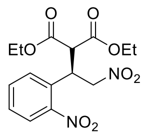

^{1}H NMR (400 MHz, $CDCl_3$) δ 7.87 (dd, J = 8.1, 1.3 Hz, 1H), 7.52 (td, J = 7.6, 1.3 Hz, 1H), 7.44 – 7.32 (m, 2H), 5.09 (dd, J = 13.8, 7.9 Hz, 1H), 4.98 (dd, J = 13.8, 4.3 Hz, 1H), 4.67 (td, J = 8.1, 4.3 Hz, 1H), 4.21 – 4.09 (m, 3H), 4.05 – 3.94 (m, 2H), 1.19 (dd, J = 9.0, 5.2 Hz, 3H), 1.03 (t, J = 7.1 Hz, 3H) ppm.

^{13}C NMR (101 MHz, $CDCl_3$) δ 166.3, 165.6, 149.0, 132.3, 130.3, 128.3, 124.4, 75.2, 61.3, 61.2, 52.6, 36.7, 12.9, 12.7 ppm.

HPLC (Chiralpak AS-H, 98:2 *n*-hexane/isopropyl alcohol, 1 mL/min, 215 nm): t_{major} = 47.570 min, t_{minor} = 64.163; 80% ee.

$[\alpha]_D^{25}$ = -3.48° (c 1.0, $CHCl_3$).

The absolute configuration was assigned as (*R*) by comparison of the optical rotation with the following literature value.[3] $[\alpha]_D^{25}$ = +1.6° (c 0.8, $CHCl_3$), [87% ee, (*S*)-enantiomer].

Data for 5c:

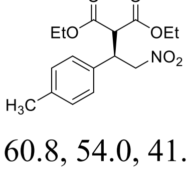

^{1}H NMR (400 MHz, $CDCl_3$) δ 7.04 (s, 4H), 4.83 (dd, J = 13.0, 4.9 Hz, 1H), 4.76 (dd, J = 13.0, 9.2 Hz, 1H), 4.18 – 4.10 (m, 3H), 3.94 (q, J = 7.1 Hz, 2H), 3.73 (d, J = 9.3 Hz, 1H), 2.22 (s, 3H), 1.19 (t, J = 7.2 Hz, 3H), 0.99 (t, J = 7.1 Hz, 3H) ppm.

^{13}C NMR (101 MHz, $CDCl_3$) δ 166.5, 165.9, 137.1, 132.1, 128.6, 126.8, 76.8, 61.1, 60.8, 54.0, 41.6, 20.0, 13.0, 12.7 ppm.

HPLC (Chiralpak AD, 85:15 *n*-hexane/isopropyl alcohol, 1 mL/min, 215 nm): t_{major} = 10.902 min, t_{minor} = 28.823; 91% ee.

$[\alpha]_D^{25}$ = -3.20° (c 1.0, $CHCl_3$).

The absolute configuration was assigned as (*R*) by comparison of the optical rotation with the following literature value.[2] $[\alpha]_D^{25}$ = +6.25° (c 1.36, $CHCl_3$), [95% ee, (*S*)-enantiomer].

Data for 5d:

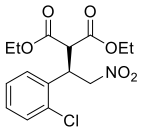

^{1}H NMR (400 MHz, $CDCl_3$) δ 7.38 – 7.29 (m, 1H), 7.22 – 7.12 (m, 3H), 5.03 (dd, *J* = 13.5, 8.5 Hz, 1H), 4.88 (dd, *J* = 13.5, 4.4 Hz, 1H), 4.67 (td, *J* = 8.6, 4.4 Hz, 1H), 4.17 – 4.10 (m, 2H), 4.00 (dd, *J* = 14.3, 7.3 Hz, 3H), 1.17 (t, *J* = 7.1 Hz, 3H), 1.03 (t, *J* = 7.1 Hz, 3H) ppm.
^{13}C NMR (101 MHz, $CDCl_3$) δ 168.4, 167.8, 135.2, 134.8, 131.5, 130.5, 129.9, 128.3, 76.7, 63.1, 63.0, 54.2, 40.5, 14.9, 14.85 ppm.
HPLC (Chiralpak IA, 80:20 *n*-hexane/isopropyl alcohol, 1 mL/min, 215 nm): t_{major} = 6.534 min, t_{minor} = 28.673; 90% ee.
$[\alpha]_D^{25}$= -5.70° (*c* 1.0, $CHCl_3$).
The absolute configuration was assigned as (*R*) by comparison of the optical rotation with the following literature value.[2] $[\alpha]_D^{25}$= +11.1° (*c* 1.13, $CHCl_3$), [92% ee, (*S*)-enantiomer].

Data for 5e:

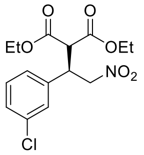

^{1}H NMR (400 MHz, $CDCl_3$) δ 7.23 – 7.14 (m, 3H), 7.12 – 7.03 (m, 1H), 4.85 (dd, *J* = 13.3, 4.8 Hz, 1H), 4.78 (dd, *J* = 13.3, 9.3 Hz, 1H), 4.23 – 4.08 (m, 3H), 3.98 (q, *J* = 7.1 Hz, 2H), 3.72 (d, *J* = 9.1 Hz, 1H), 1.19 (t, *J* = 7.1 Hz, 3H), 1.02 (t, *J* = 7.1 Hz, 3H) ppm.
^{13}C NMR (101 MHz, $CDCl_3$) δ 166.2, 165.6, 137.4, 133.8, 129.2, 127.6, 127.3, 125.3,

76.2, 61.3, 61.1, 53.7, 41.5, 12.9, 12.7 ppm.

HPLC (Chiralpak IA, 80:20 *n*-hexane/isopropyl alcohol, 1 mL/min, 215 nm): t_{major} = 7.419 min, t_{minor} = 12.914; 88% ee.
IR (neat) 2983, 1728, 1554, 1476, 1435, 1369, 1299, 1253, 1231, 1177, 1085, 1024, 860, 789, 695 cm^{-1}.
HRMS (ESI) calcd for $C_{15}H_{17}ClNO_6$ $[M+H]^-$ 342.0744, found 342.0724.

$[\alpha]_D^{25}$= -7.20° (*c* 1.0, $CHCl_3$).

Data for 5f:

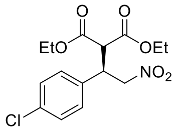

^{1}H NMR (400 MHz, $CDCl_3$) δ 7.25 – 7.19 (m, 2H), 7.15 – 7.09 (m, 2H, 2xAr*H*), 4.84 (dd, *J* = 13.2, 4.8 Hz, 1H), 4.76 (dd, *J* = 13.2, 9.3 Hz, 1H), 4.21 – 4.10 (m, 3H), 3.96 (q, *J* = 7.1 Hz, 2H), 3.71 [d, *J* = 9.3 Hz, 1H], 1.19 (t, *J* = 7.1 Hz, 3H), 1.02 (t, *J* = 7.1 Hz, 3H) ppm.
^{13}C NMR (101 MHz, $CDCl_3$) δ 166.2, 165.6, 133.8, 133.3, 128.4, 128.2, 76.4, 61.3, 61.0, 53.7, 41.3, 12.9, 12.8 ppm.
HPLC (Chiralpak IA, 80:20 *n*-hexane/isopropyl alcohol, 1 mL/min, 254 nm): t_{major} = 11.175 min, t_{minor} = 33.172; 90% ee.
$[\alpha]_D^{25}$= -2.40° (*c* 1.0, $CHCl_3$).
The absolute configuration was assigned as (*R*) by comparison of the optical rotation with the following literature value.[4] $[\alpha]_D^{25}$ = -5.6° (*c* 1.0, CH_2Cl_2), [88% ee, (*R*)-enantiomer].

Data for 5g:

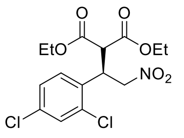

^{1}H NMR (400 MHz, $CDCl_3$) δ 7.36 (s, 1H), 7.18 – 7.10 (m, 2H), 5.01 (dd, J = 13.6, 8.6 Hz, 1H), 4.85 (dd, J = 13.6, 4.3 Hz, 1H), 4.62 (td, J = 8.6, 4.3 Hz, 1H), 4.14 (dd, J = 5.0, 2.2 Hz, 2H), 4.03 (q, J = 7.1 Hz, 2H), 3.96 (d, J = 8.7 Hz, 1H), 1.23 – 1.19 (t, 3H), 1.07 (t, J = 7.1 Hz, 3H).

^{13}C NMR (101 MHz, $CDCl_3$) δ 166.2, 165.6, 133.9, 133.8, 131.5, 129.3, 128.8, 126.6, 74.5, 61.3, 61.2, 52.0, 40.7, 13.0, 12.9.

HPLC (Chiralpak AD, 65:35 *n*-hexane/isopropyl alcohol, 1 mL/min, 215 nm): t_{major} = 4.922 min, t_{minor} = 23.089; 92% ee.

$[\alpha]_D^{25}$ = -11.10° (*c* 1.0, $CHCl_3$).

The absolute configuration was assigned as (*R*) by comparison of the optical rotation with the following literature value.[4] $[\alpha]_D^{25}$ = -6.3° (*c* 1.15, CH_2Cl_2), [94% ee, (*R*)-enantiomer].

Data for 5h:

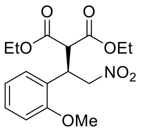

^{1}H NMR (400 MHz, $CDCl_3$) δ 7.21 – 7.15 (m, 1H), 7.07 (dd, J = 7.7, 1.6 Hz, 1H), 6.80 (ddd, J = 7.4, 4.8, 2.6 Hz, 2H), 4.95 (dd, J = 12.9, 9.1 Hz, 1H), 4.80 (dd, J = 12.9, 4.5 Hz, 1H), 4.31 (td, J = 9.6, 4.5 Hz, 1H), 4.20 – 4.05 (m, 3H), 3.87 (q, J = 7.1 Hz, 2H), 3.80 (s, 3H), 1.19 (t, J = 7.1 Hz, 3H), 0.93 (t, J = 7.1 Hz, 3H).

^{13}C NMR (101 MHz, $CDCl_3$) δ 166.9, 166.2, 156.4, 129.9, 128.6, 122.8, 119.8, 110.1, 75.2, 60.9, 60.5, 54.4, 51.7, 39.5, 13.0, 12.7.

HPLC (Chiralpak AD, 90:10 *n*-hexane/isopropyl alcohol, 1 mL/min, 215 nm): t_{major} = 9.792 min, t_{minor} = 13.127; 92% ee.

$[\alpha]_D^{25}$ = -16.30° (*c* 1.0, $CHCl_3$).

The absolute configuration was assigned as (*R*) by comparison of the optical rotation with the following literature value.[5] $[\alpha]_D^{25}$ = -20.3° (*c* 1.0, $CHCl_3$), [96% ee, (*R*)-enantiomer].

Data for 5i:

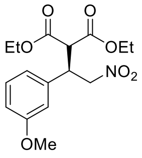

^{1}H NMR (400 MHz, $CDCl_3$) δ 7.16 (dd, J = 16.6, 8.7 Hz, 1H), 6.81 – 6.64 (m, 3H), 4.84 (dd, J = 13.1, 5.0 Hz, 1H), 4.78 (dd, J = 13.1, 8.9 Hz, 1H), 4.21 – 4.10 (m, 3H), 3.96 (q, J = 7.1 Hz, 2H), 3.74 [d, J = 9.2 Hz, 1H], 3.70 (s, 3H), 1.19 (t, J = 7.1 Hz, 3H), 1.00 (t, J = 7.1 Hz, 3H) ppm.

^{13}C NMR (101 MHz, $CDCl_3$) δ 166.5, 165.8, 158.9, 136.8, 129.0, 119.0, 113.1, 112.6, 76.6, 61.1, 60.9, 54.2, 54.0, 41.9, 12.9, 12.8 ppm.

HPLC (Chiralpak AD, 80:20 *n*-hexane/isopropyl alcohol, 1 mL/min, 215 nm): t_{major} = 9.230 min, t_{minor} = 12.091; 88% ee.

IR (neat) 2981, 2936, 2906, 1728, 1602, 1586, 1489, 1456, 1438, 1369, 1292, 1259, 1174, 1154, 1095, 1026,858, 785, 755, 699 cm^{-1}.

HRMS (ESI) calcd for $C_{16}H_{20}NO_7$ $[M+H]^-$ 338.1240, found 338.1199.

$[\alpha]_D^{25}$ = -2.76° (*c* 1.0, $CHCl_3$).

Data for 5j:

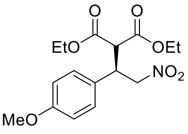

^{1}H NMR (400 MHz, $CDCl_3$) δ 7.12 – 7.05 (m, 2H), 6.81 – 6.72 (m, 2H), 4.82 (dd, *J* = 12.9, 4.9 Hz, 1H), 4.74 (dd, *J* = 12.9, 9.3 Hz, 1H), 4.20 – 4.08 (m, 3H), 3.95 (q, *J* = 7.1 Hz, 2H), 3.72 (d, *J* = 3.8 Hz, 1H), 3.70 (s, 3H,), 1.19 (t, *J* = 7.1 Hz, 3H), 1.00 (t, *J* = 7.1 Hz, 3H) ppm.

^{13}C NMR (101 MHz, $CDCl_3$) δ 166.5, 165.9, 158.4, 128.1, 127.0, 113.3, 76.9, 61.2, 60.8, 54.2, 54.1, 41.3, 13.0, 12.8 ppm.

HPLC (Chiralpak AD, 80:20 *n*-hexane/isopropyl alcohol, 1 mL/min, 215nm): t_{major} = 12.676 min, t_{minor} = 39.547; 94% ee.

$[\alpha]_D^{25}$ = -5.70° (*c* 1.0, $CHCl_3$).

The absolute configuration was assigned as (*R*) by comparison of the optical rotation with the following literature value.[4] $[\alpha]_D^{25}$ = -3.0° (*c* 1.0, CH_2Cl_2), [88% ee, (*R*)-enantiomer].

Data for 5k:

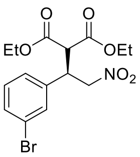

^{1}H NMR (400 MHz, $CDCl_3$) δ 7.41 – 7.28 (m, 2H), 7.18 – 7.07 (m, 2H), 4.85 (dd, *J* = 13.4, 4.8 Hz, 1H), 4.77 (dd, *J* = 13.3, 9.3 Hz, 1H), 4.20 – 4.10 (m, 3H), 3.98 (q, *J* = 7.1 Hz, 2H), 3.71 (d, *J* = 9.1 Hz, 1H), 1.19 (t, *J* = 7.1 Hz, 3H), 1.02 (t, *J* = 7.1 Hz, 3H) ppm.

^{13}C NMR (101 MHz, $CDCl_3$) δ 166.2, 165.6, 137.7, 130.5, 130.2, 129.5, 125.7, 121.9, 76.2, 61.3, 61.1, 53.7, 41.5, 13.0, 12.8 ppm.

HPLC (Chiralpak AD, 80:20 *n*-hexane/isopropyl alcohol, 1 mL/min, 215 nm): t_{major} = 8.934 min, t_{minor} = 15.886; 87% ee.

IR (neat) 2982, 2936, 1727, 1596, 1552, 1474, 1431, 1369, 1299, 1252, 1228, 1177, 1153, 1095, 1075, 1025, 998, 859, 788, 694, 665 cm^{-1}.

HRMS (ESI) calcd for $C_{15}H_{17}BrNO_6$ $[M+H]^-$ 386.0239, found 386.0192.

$[\alpha]_D^{25}$ = -3.70° (*c* 1.0, $CHCl_3$).

Data for 5l:

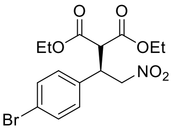

^{1}H NMR (400 MHz, $CDCl_3$) δ 7.45 – 7.33 (m, 2H), 7.13 – 7.01 (m, 2H), 4.84 (dd, *J* = 13.2, 4.8 Hz, 1H), 4.76 (dd, *J* = 13.2, 9.3 Hz, 1H), 4.21 – 4.09 (m, 3H), 3.96 (q, *J* = 7.1 Hz, 2H), 3.71 (d, *J* = 9.3 Hz, 1H), 1.19 (t, *J* = 7.2 Hz, 3H), 1.02 (t, *J* = 7.1 Hz, 3H) ppm.

^{13}C NMR (101 MHz, $CDCl_3$) δ 167.2, 166.6, 135.3, 132.1, 129.8, 122.5, 77.3, 62.3, 62.1, 54.7, 42.4, 14.0, 13.8 ppm.

HPLC (Chiralpak AD, 80:20 *n*-hexane/isopropyl alcohol, 1 mL/min, 215nm): t_{major} = 14.197 min, t_{minor} = 39.377, 92% ee.

$[\alpha]_D^{25}$ = -4.50° (*c* 1.0, $CHCl_3$).

The absolute configuration was assigned as (*R*) by comparison of the optical rotation with the following literature value.[2] $[\alpha]_D^{25}$ = +8.4° (*c* 1.04, $CHCl_3$), [95% ee, (*S*)-enantiomer].

Data for 5m:

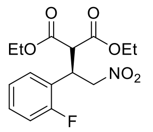

^{1}**H NMR** (400 MHz, $CDCl_3$) δ 7.25 – 7.14 (m, 2H), 7.05 – 6.95 (m, 2H), 4.94 – 4.79 (m, 2H), 4.44 – 4.30 (m, 1H), 4.18 – 4.12 (m, 2H), 3.91 (dt, *J* = 10.0, 7.7 Hz, 3H), 1.19 (dd, *J* = 7.1, 5.8 Hz, 3H), 0.96 (t, *J* = 7.1 Hz, 3H) ppm.

^{13}**C NMR** (101 MHz, $CDCl_3$) δ 166.3, 165.7, 160.0 (d, *J* = 247.1 Hz), 129.7 (d, *J* = 4.1 Hz), 129.2 (d, *J* = 8.6 Hz), 123.5 (d, *J* = 3.3 Hz), 122.1 (d, *J* = 13.0 Hz), 115.1 (d, *J* = 22.1 Hz), 75.3 (d, *J* = 2.4 Hz), 61.2, 60.9, 52.3, 37.6, 12.9, 12.7 ppm.

HPLC (Chiralpak AD, 90:10 *n*-hexane/isopropyl alcohol, 1 mL/min, 215 nm): t_{major} = 11.973 min, t_{minor} = 35.137; 93% ee.
IR (neat) 2984, 2936, 1730, 1616, 1555, 1492, 1457, 1370, 1297, 1234, 1177, 1152, 1108, 1096, 1025, 943,859, 838, 759,687, 610 cm^{-1}.
HRMS (ESI) calcd for $C_{15}H_{17}FNO_6$ $[M+H]^-$ 326.1040, found 326.0994.

$[\alpha]_D^{25}$ = -16.0° (*c* 1.0, $CHCl_3$).

Data for 5n:

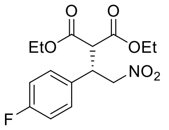

^{1}**H NMR** (400 MHz, $CDCl_3$) δ 7.20 – 7.12 (m, 2H), 7.02 – 6.86 (m, 2H), 4.84 (dd, *J* = 13.1, 4.7 Hz, 1H), 4.75 (dd, *J* = 13.1, 9.4 Hz, 1H), 4.22 – 4.10 (m, 3H), 3.95 (q, *J* = 7.1 Hz, 2H), 3.71 [d, *J* = 9.4 Hz, 1H], 1.19 (t, *J* = 7.1 Hz, 3H), 1.04 – 0.96 (t, 3H) ppm.

^{13}**C NMR** (101 MHz, $CDCl_3$) δ 166.3, 165.7, 161.5 (d, *J* = 247.5 Hz), 131.0 (d, *J* = 2.4 Hz), 128.8 (d, *J* = 8.2 Hz), 114.9 (d, *J* = 21.5 Hz), 76.7, 61.2, 60.95, 53.9, 41.3, 12.9, 12.8 ppm.
HPLC (Chiralpak AS-H, 95:5 *n*-hexane/isopropyl alcohol, 0.8 mL/min, 215nm): t_{minor} = 18.800, t_{major} = 22.093 min; 94% ee.
$[\alpha]_D^{25}$ = -6.0° (*c* 1.0, $CHCl_3$).
The absolute configuration was assigned as (*S*) by comparison of the optical rotation with the following literature value.[6] $[\alpha]_D^{25}$ = -7.20° (*c* 1.0, $CHCl_3$), [92% ee, (*S*)-enantiomer].

Data for 5o:

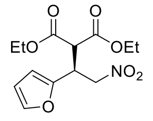

^{1}**H NMR** (400 MHz, $CDCl_3$) δ 7.27 (dd, *J* = 1.8, 0.7 Hz, 1H), 6.22 (dd, *J* = 3.3, 1.9 Hz, 1H), 6.15 (d, *J* = 3.3 Hz, 1H), 4.85 (dd, *J* = 11.5, 6.2 Hz, 1H), 4.81 (dd, *J* = 11.5, 3.3 Hz, 1H), 4.31 (td, *J* = 8.0, 5.2 Hz, 1H), 4.18 – 4.12 (m, 2H), 4.07 (q, *J* = 7.1 Hz, 2H), 3.87 – 3.79 (m, 1H), 1.19 (t, *J* = 7.1 Hz, 3H), 1.13 (t, *J* = 7.1 Hz, 3H) ppm.
^{13}**C NMR** (101 MHz, $CDCl_3$) δ 166.1, 165.8, 148.6, 141.7, 109.5, 107.4, 74.4, 61.1, 52.0, 35.8, 12.9, 12.9 ppm.

HPLC (Chiralpak IA, 80:20 *n*-hexane/isopropyl alcohol, 1 mL/min, 215 nm): t_{major} = 6.238 min, t_{minor} = 7.119; 91% ee.
$[\alpha]_D^{25}$ = +2.62° (*c* 1.0, $CHCl_3$).
The absolute configuration was assigned as (*S*) by comparison of the optical rotation with the following literature value.[2] $[\alpha]_D^{25}$ = -2.8° (*c* 1.22, $CHCl_3$), [95% ee, (*R*)-enantiomer].

Data for 5p:

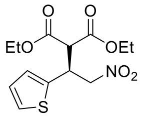

^{1}H NMR (400 MHz, $CDCl_3$) δ 7.16 (dd, J = 5.0, 1.1 Hz, 1H), 6.89 (d, J = 2.5 Hz, 1H), 6.86 (dd, J = 5.0, 3.6 Hz, 1H), 4.87 (dd, J = 12.7, 4.6 Hz, 1H), 4.83 (dd, J = 12.7, 7.6 Hz, 1H), 4.48 (td, J = 8.1, 5.3 Hz, 1H), 4.20 – 4.11 (m, 2H), 4.05 (q, J = 7.1 Hz, 2H), 3.87 – 3.74 (d, 1H), 1.20 (t, J = 5.5 Hz, 3H), 1.09 (t, J = 7.1 Hz, 3H) ppm.

^{13}C NMR (101 MHz, $CDCl_3$) δ 166.9, 166.4, 138.3, 126.7, 126.5, 125.3, 77.8, 61.9, 61.8, 55.3, 38.1, 13.7, 13.5 ppm.

HPLC (Chiralpak AD, 90:10 *n*-hexane/isopropyl alcohol, 1 mL/min, 215 nm) t_{major} = 11.318 min, t_{minor} = 20.136; 88% ee.

$[\alpha]_D^{25}$ = +3.50° (c 1.0, $CHCl_3$).

The absolute configuration was assigned as (S) by comparison of the optical rotation with the following literature value.[4] $[\alpha]_D^{25}$ = +8.7° (c 1.0, CH_2Cl_2), [87% ee, (S)-enantiomer].

Data for 5r:

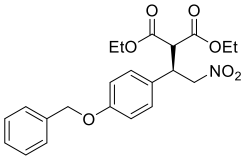

^{1}H NMR (400 MHz, $CDCl_3$) δ 7.36 – 7.22 (m, 5H), 7.11 – 7.05 (m, 2H,), 6.87 – 6.79 [m, 2H), 4.94 (s, 2H), 4.81 (dd, J = 12.9, 4.8 Hz, 1H,), 4.73 (dd, J = 12.9, 9.3 Hz, 1H), 4.20 – 4.08 (m, 3H), 3.93 (q, J = 7.1 Hz, 2H), 3.71 [t, J = 8.4 Hz, 1H], 1.18 (t, J = 7.1 Hz, 3H), 0.98 (t, J = 7.1 Hz, 3H).

^{13}C NMR (101 MHz, $CDCl_3$) δ 166.5, 165.9, 157.6, 135.7, 128.2, 127.6, 127.3, 127.0, 126.5, 114.2, 76.9, 69.0, 61.1, 60.8, 54.1, 41.3, 13.0, 12.8 ppm.

HPLC (Chiralpak AS-H, 97:3 *n*-hexane/isopropyl alcohol, 1 mL/min, 215 nm): t_{major} = 38.747 min, t_{minor} = 43.305; 99% ee.

IR (neat) 2985, 2931, 1727, 1611, 1584, 1554, 1511,1442, 1380, 1290, 1244, 1199, 1177, 1115, 1083, 1060, 1045,1016, 992, 963, 908, 861, 834, 820, 764, 736, 695, 629, 591, 560 cm^{-1}.

HRMS (ESI) calcd for $C_{22}H_{24}NO_7$ $[M+H]^-$ 414.1553, found 414.1498.

$[\alpha]_D^{25}$ = +3.50° (c 1.0, $CHCl_3$).

Data for 5ab:

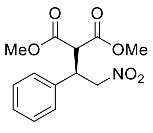

^{1}H NMR (400 MHz, $CDCl_3$) δ 7.29 – 7.12 (m, 5H), 4.89 – 4.77 (m, 2H), 4.18 (td, J = 8.9, 5.3 Hz, 1H), 3.80 (d, J = 9.1 Hz, 1H), 3.69 (s, 3H), 3.49 (s, 3H) ppm.

^{13}C NMR (101 MHz, $CDCl_3$) δ 166.8, 166.2, 135.1, 128.0, 127.4, 126.84, 76.4, 53.8, 52.0, 51.8, 41.9 ppm.

HPLC (Chiralpak AD, 90:10 *n*-hexane/isopropyl alcohol, 1 mL/min, 220 nm) t_{major} = 17.757 min, t_{minor} = 24.360; 92% ee.

$[\alpha]_D^{25}$ = -0.74° (c 1.0, $CHCl_3$).

The absolute configuration was assigned as (R) by comparison of the optical rotation with the following literature value.[4] $[\alpha]_D^{25}$ = -2.5° (c 1.04, CH_2Cl_2), [89% ee, (R)-enantiomer].

Data for 5ac:

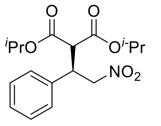

¹H NMR (400 MHz, $CDCl_3$) δ 7.27 – 7.14 (m, 5H), 5.01 (hept, *J* = 6.3 Hz, 1H), 4.86 (dd, *J* = 12.9, 4.6 Hz, 1H), 4.82 – 4.71 (m, 2H), 4.14 (td, *J* = 9.5, 4.6 Hz, 1H), 3.69 (d, *J* = 9.6 Hz, 1H), 1.18 (d, *J* = 6.3 Hz, 6H), 1.00 (d, *J* = 6.3 Hz, 3H), 0.95 (t, *J* = 6.5 Hz, 3H) ppm.

¹³C NMR (101 MHz, $CDCl_3$) δ 166.1, 165.3, 135.3, 129.5, 127.9, 127.3, 127.1, 76.9, 68.9, 68.5, 54.2, 41.9, 29.9, 20.6, 20.4, 20.3 ppm.

HPLC (Chiralpak AD, 95:5 *n*-hexane/isopropyl alcohol, 1 mL/min, 215nm): t_{major} = 19.500 min, t_{minor} = 42.532; 85% ee.

$[\alpha]_D^{25}$ = -6.60° (*c* 1.0, $CHCl_3$).

The absolute configuration was assigned as (*R*) by comparison of the optical rotation with the following literature value.[4] $[\alpha]_D^{25}$ = -6.5° (*c* 1.0, CH_2Cl_2), [89% ee, (*R*)-enantiomer].

Data for 5ad:

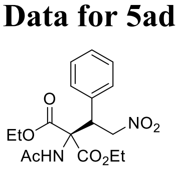

¹H NMR (400 MHz, $CDCl_3$) δ 7.22 (dd, *J* = 6.7, 3.6 Hz, 3H), 7.13 (ddd, *J* = 5.6, 4.7, 3.5 Hz, 2H), 6.64 (s, 1H), 5.52 – 5.36 (m, 1H), 4.72 – 4.54 (m, 2H), 4.28 – 4.16 (m, 2H), 4.14 – 4.05 (m, 1H), 3.98 (dq, *J* = 10.7, 7.2 Hz, 1H), 2.05 (s, 3H), 1.20 (dd, J =7.1, 2.6 Hz, 3H), 1.17 (t, *J* = 7.2 Hz, 3H) ppm.

¹³C NMR (101 MHz, $CDCl_3$) δ 169.1, 165.5, 164.8, 132.9, 127.8, 127.8, 127.7, 75.9, 66.3, 62.6, 61.8, 47.4, 22.1, 12.9, 12.8 ppm.

HPLC (Chiralpak OJ-H, 85:15 *n*-hexane/isopropyl alcohol, 1 mL/min, 215 nm): t_{minor} = 19.695, t_{major} = 27.980 min; 66% ee.

$[\alpha]_D^{25}$ = +21.26° (*c* 1.0, $CHCl_3$).

The absolute configuration was assigned as (*R*) by comparison of the optical rotation with the following literature value.[2] $[\alpha]_D^{25}$ = -42.5° (*c* 1.04, $CHCl_3$), [94% ee, (*S*)-enantiomer].

2.4. GRAM-SCALE FORMAL SYNTHESES OF (R)-BACLOFEN AND ITS (S)-CONFIGURATED FLUORINE ANALOG

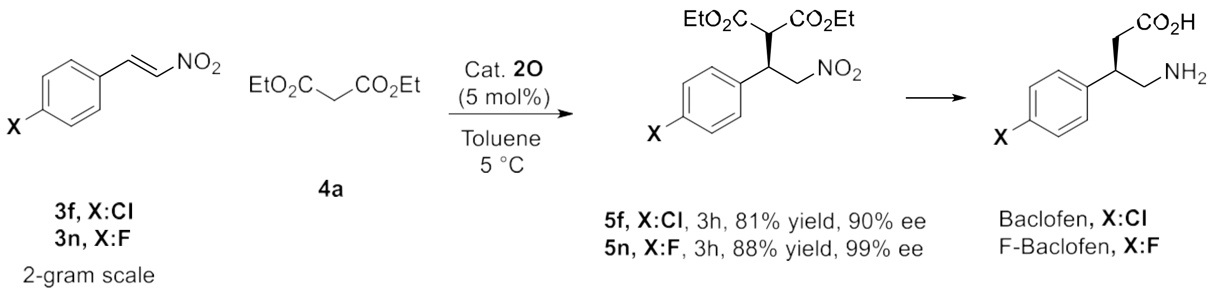

Figure S2.1 | Application of catalyst **2O** on gram-scale formal syntheses of (*R*)-Baclofen and its (*S*)-configurated fluorine analog under the optimized reaction conditions.

2.5. COPIES OF NMR SPECTRA

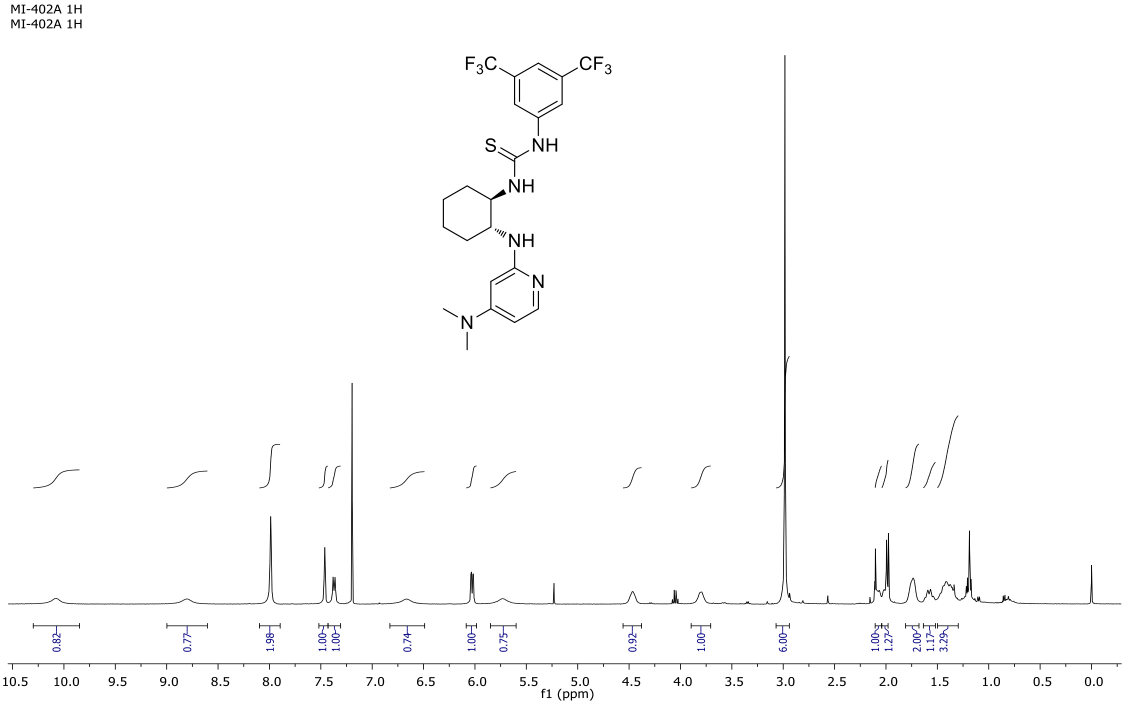

Figure S2.2 | ^{1}H NMR spectrum of 2-aminoDMAP/thiourea (**2S**)

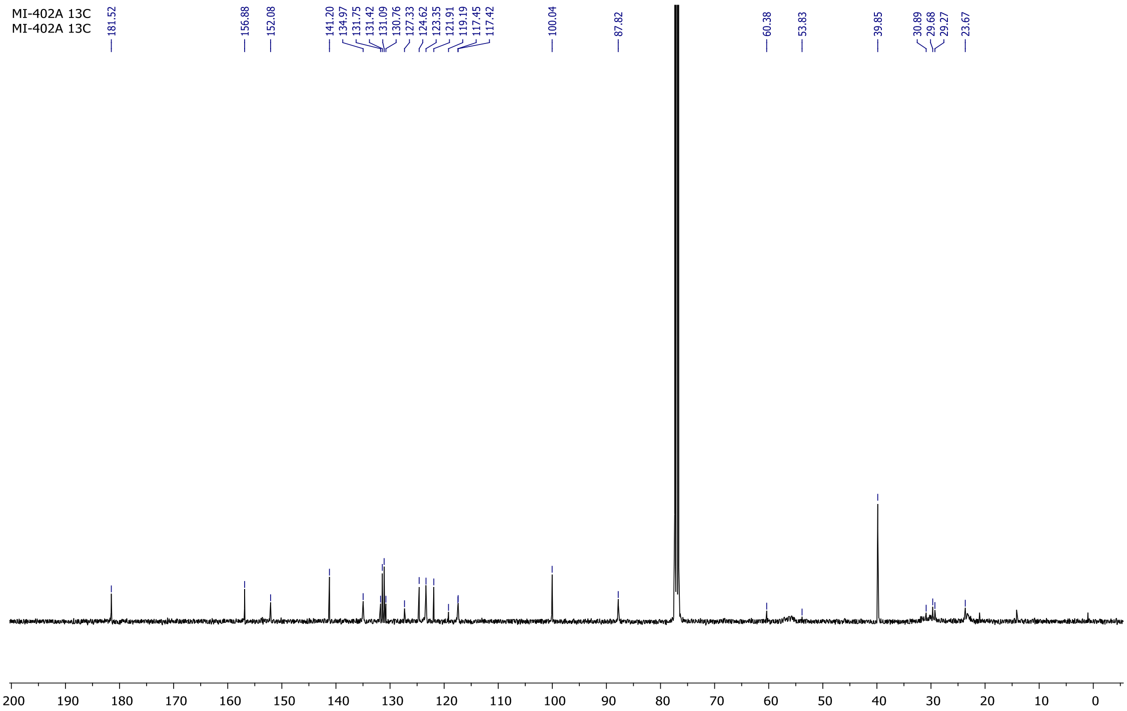

Figure S2.3 | ^{13}C NMR spectrum of 2-aminoDMAP/thiourea (**2S**)

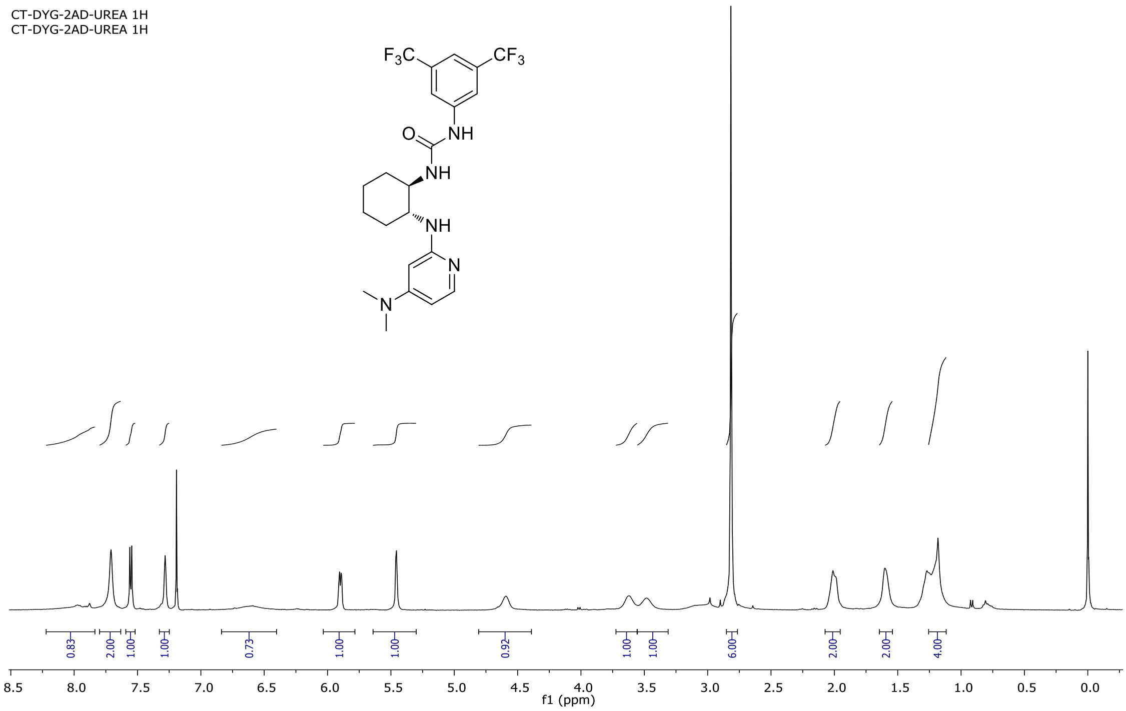

Figure S2.4 | ^{1}H NMR spectrum of 2-aminoDMAP/urea (**2O**)

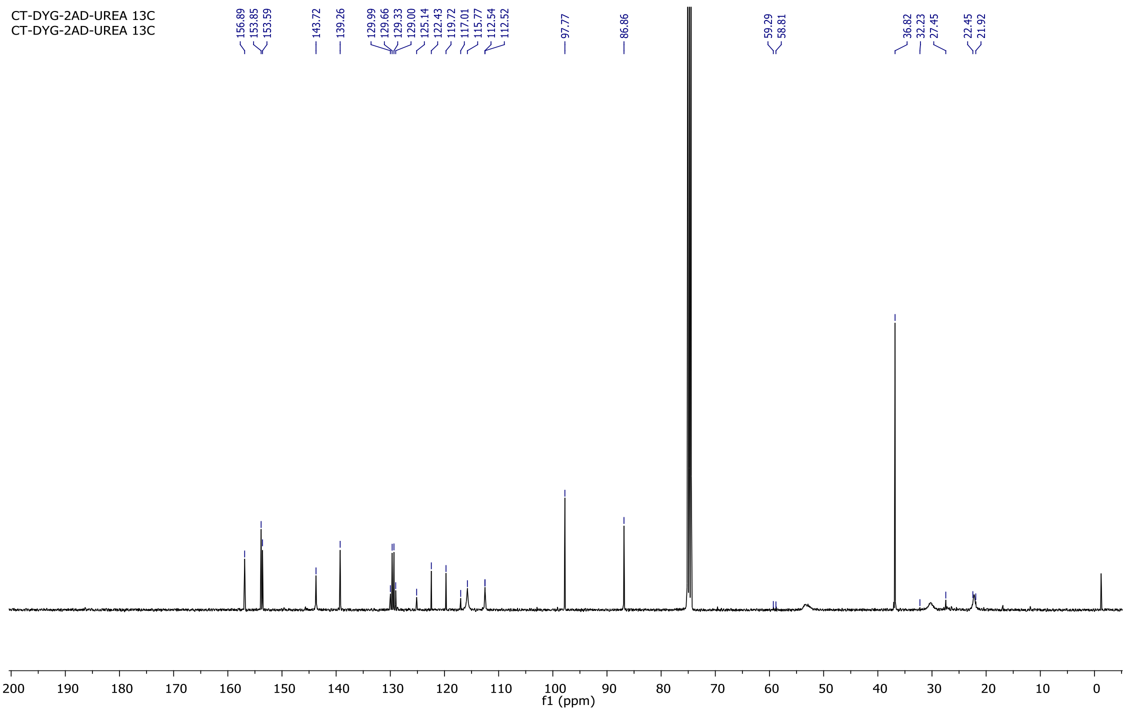

Figure S2.5 | ^{1}H NMR spectrum of 2-aminoDMAP/urea (**2O**)

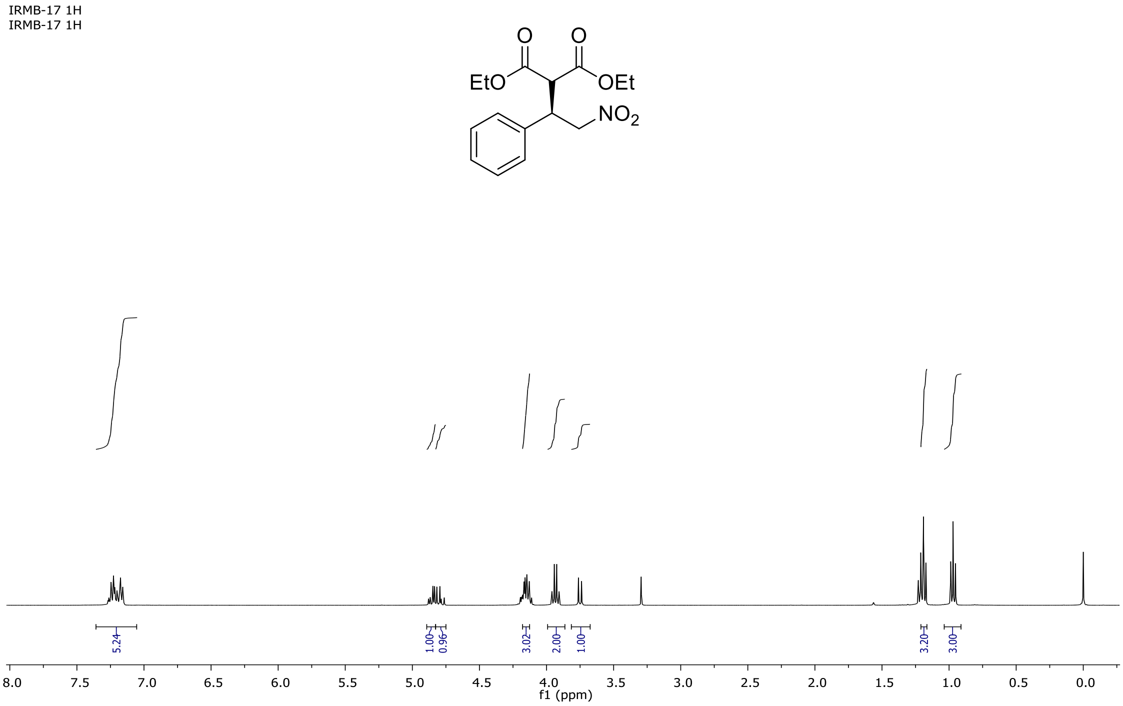

Figure S2.6 | ^{1}H NMR spectrum of chiral adduct **5a**

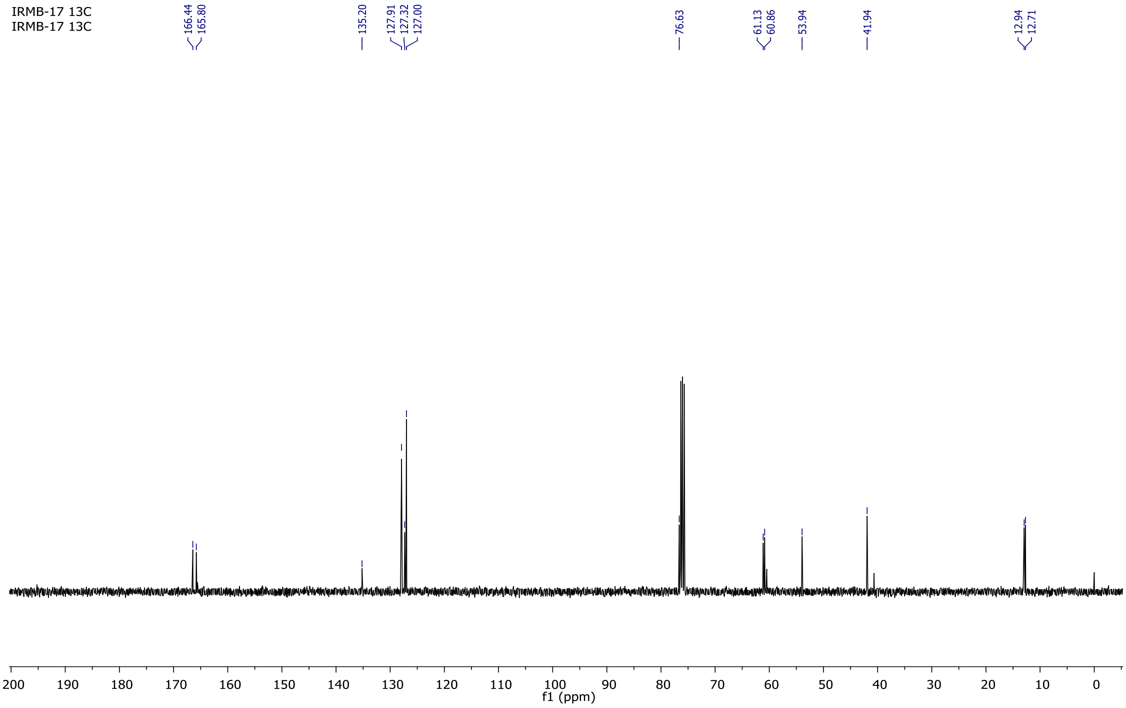

Figure S2.7 | ^{13}C NMR spectrum of chiral adduct **5a**

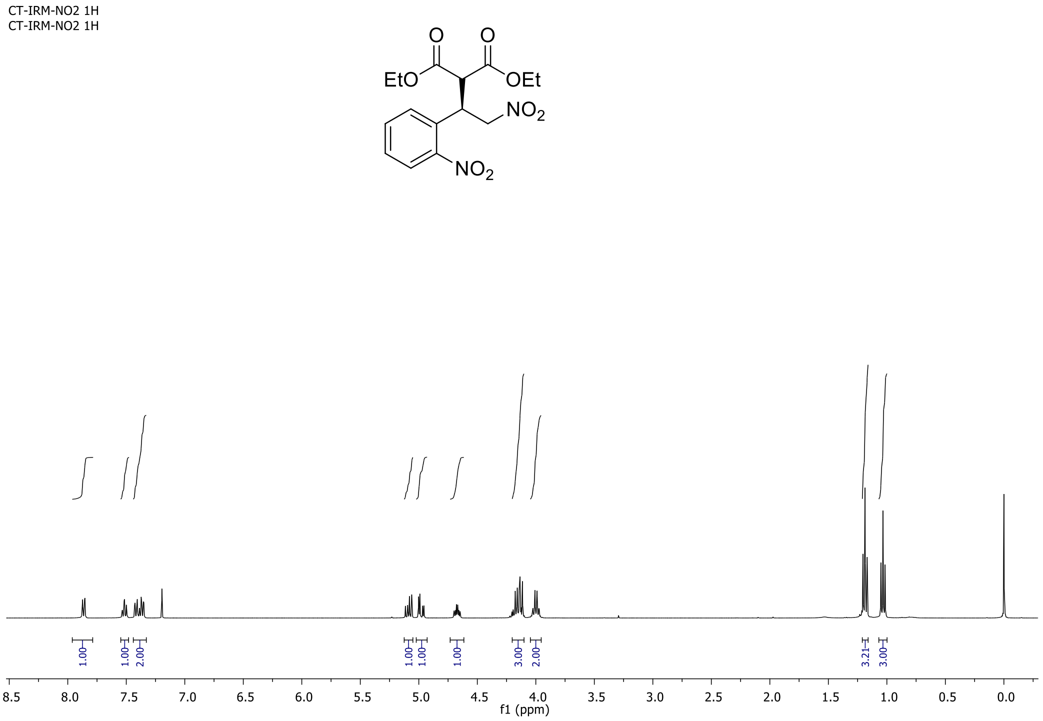

Figure S2.8 | ^{1}H NMR spectrum of chiral adduct **5b**

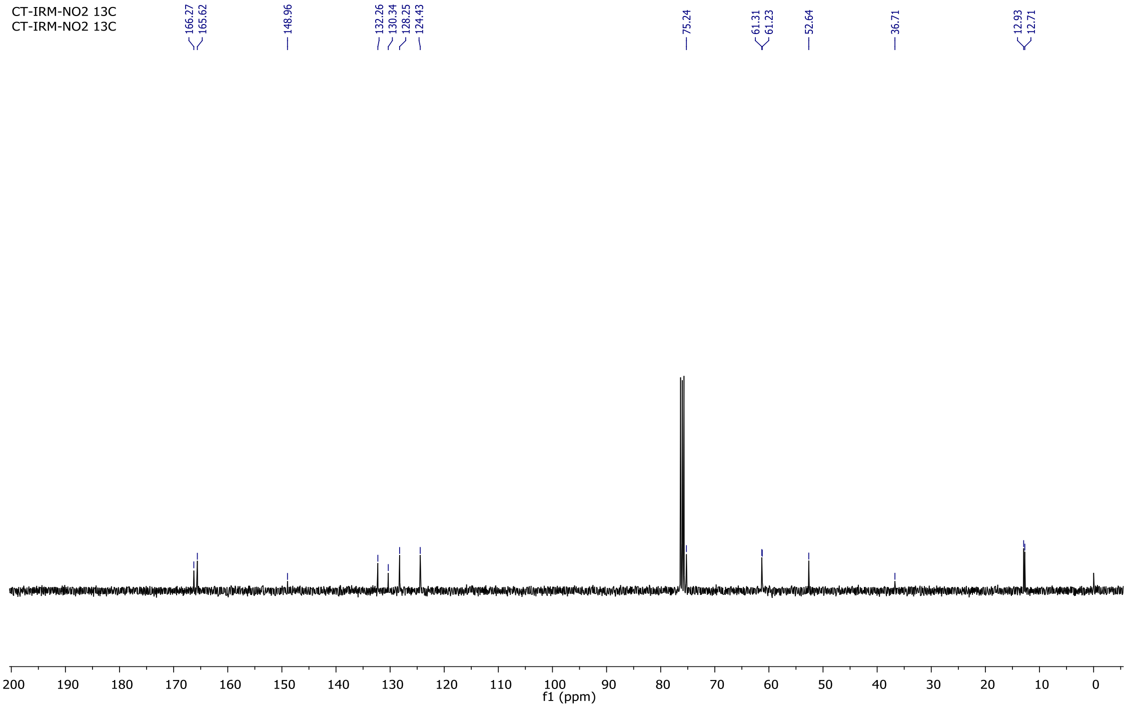

Figure S2.9 | ^{13}C NMR spectrum of chiral adduct **5b**

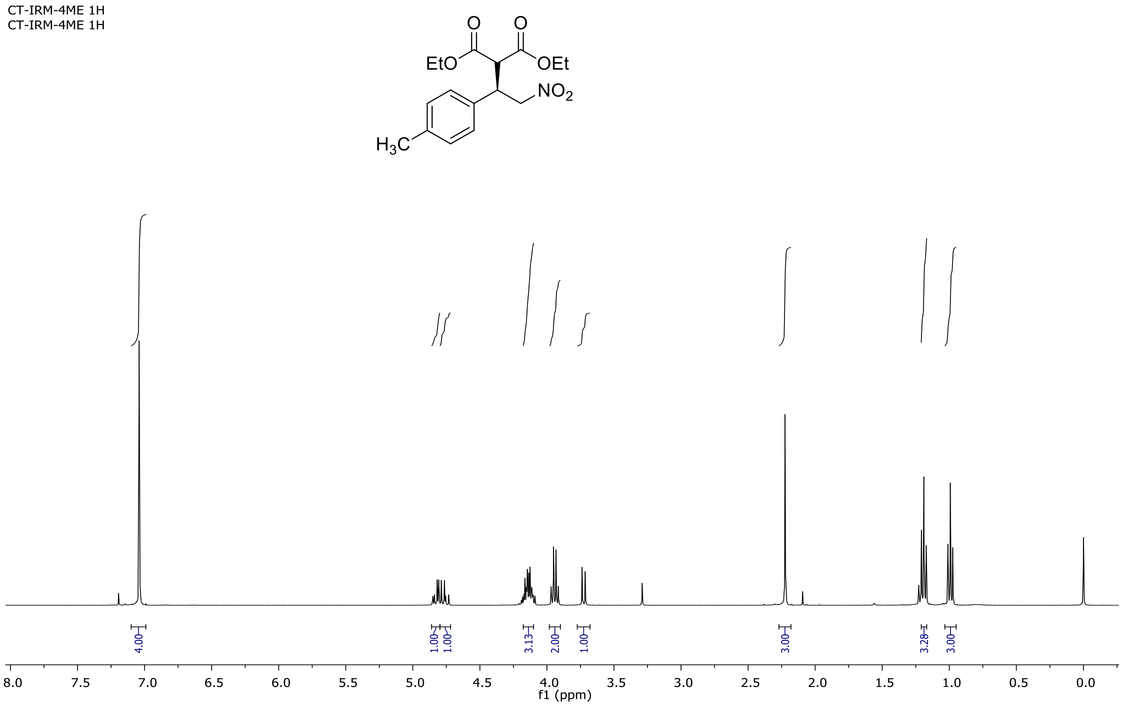

Figure S2.10 | ^{1}H NMR spectrum of chiral adduct **5c**

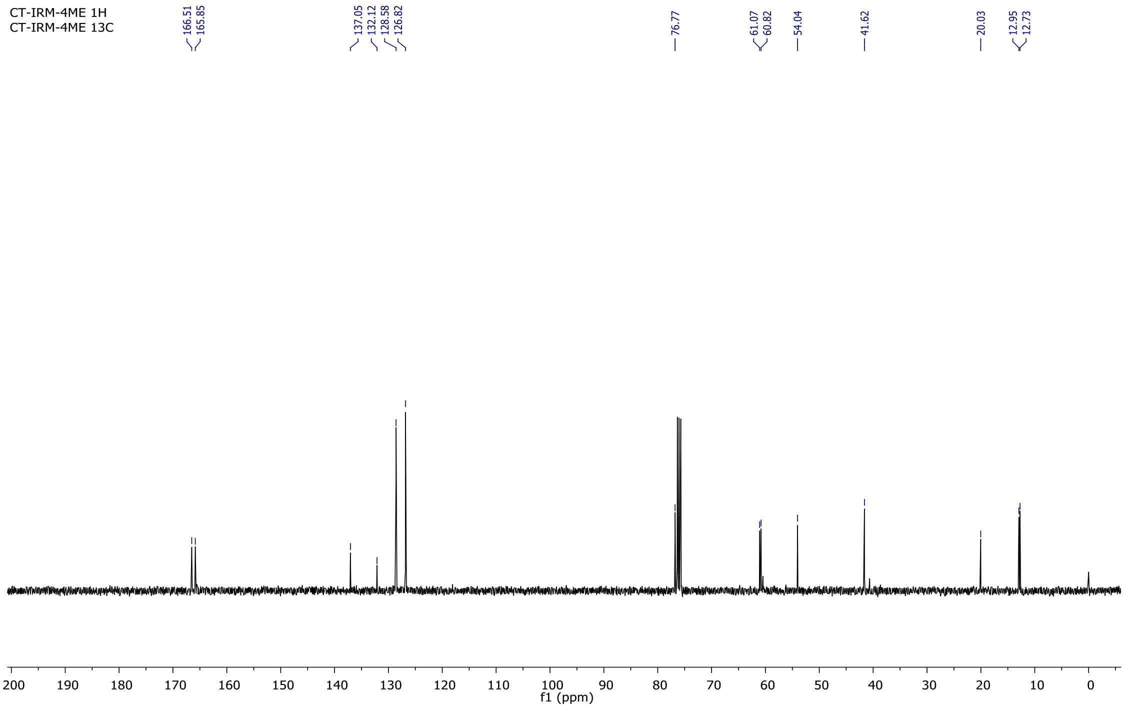

Figure S2.11 | ^{13}C NMR spectrum of chiral adduct **5c**

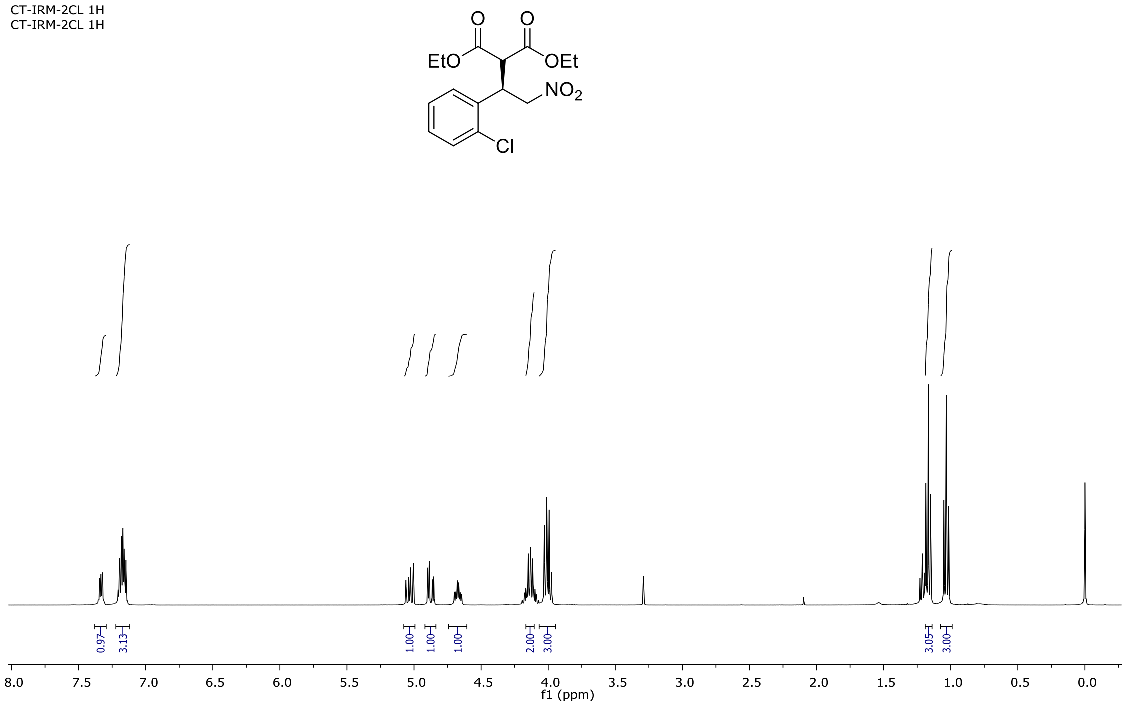

Figure S2.12 | ^{1}H NMR spectrum of chiral adduct **5d**

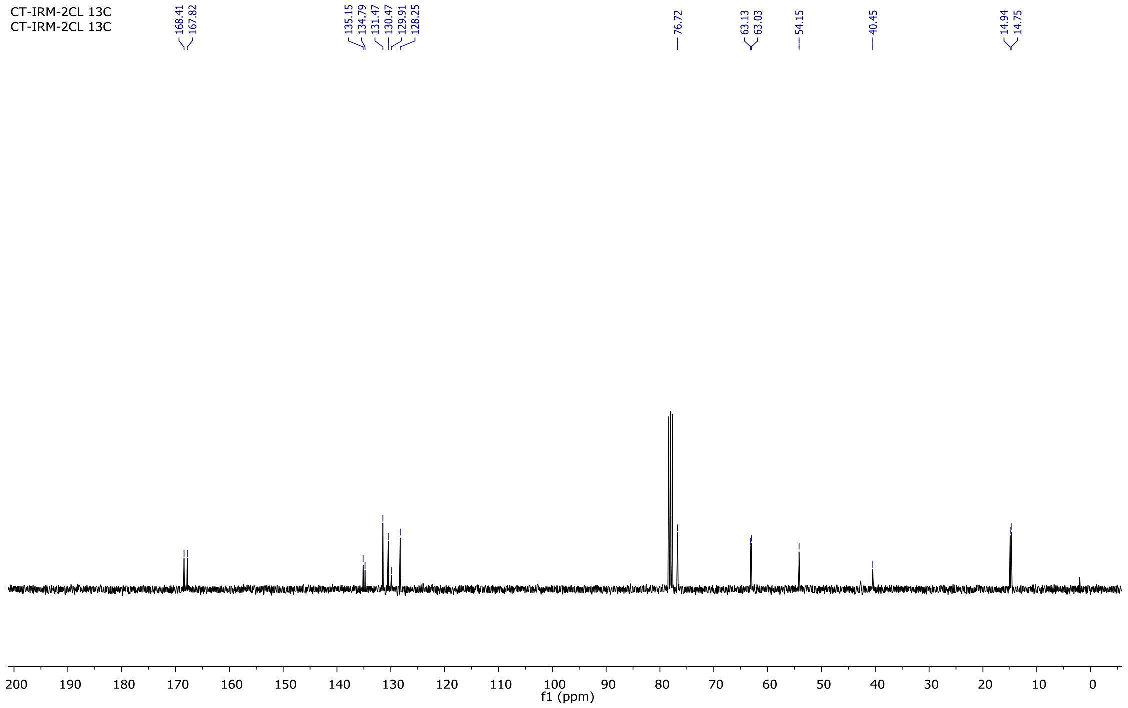

Figure S2.13 | ^{13}C NMR spectrum of chiral adduct **5d**

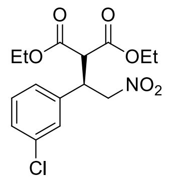

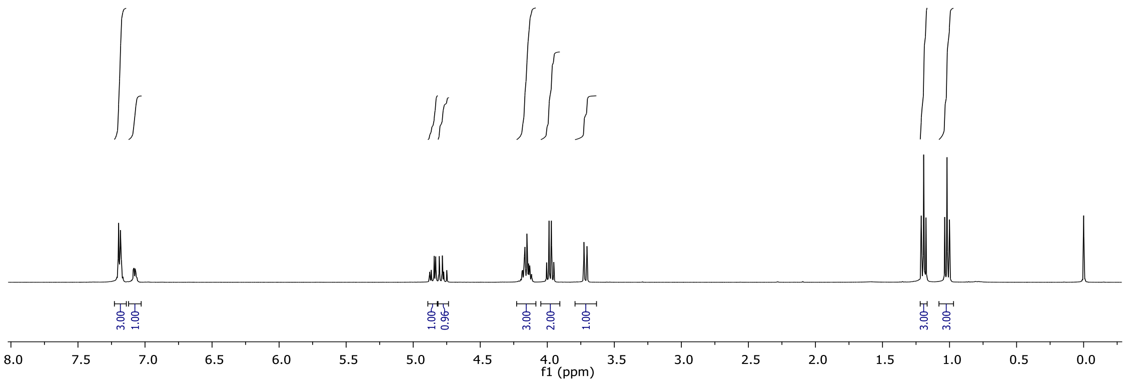

Figure S2.14 | ^{1}H NMR spectrum of chiral adduct **5e**

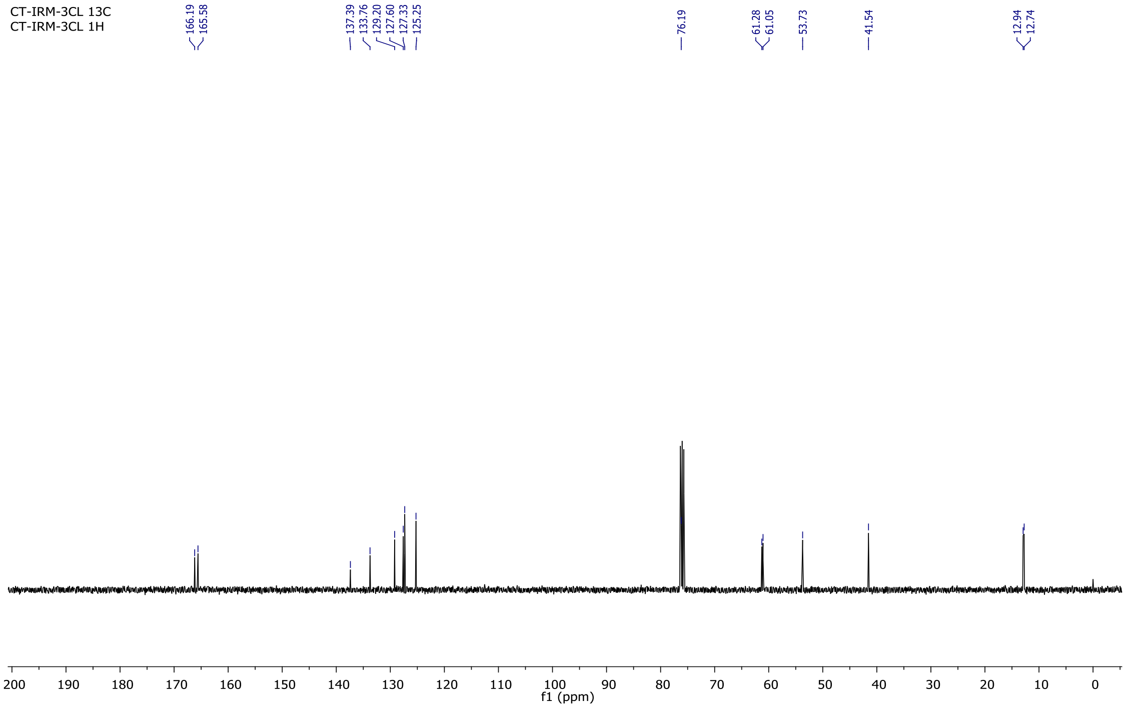

Figure S2.15 | ^{13}C NMR spectrum of chiral adduct **5e**

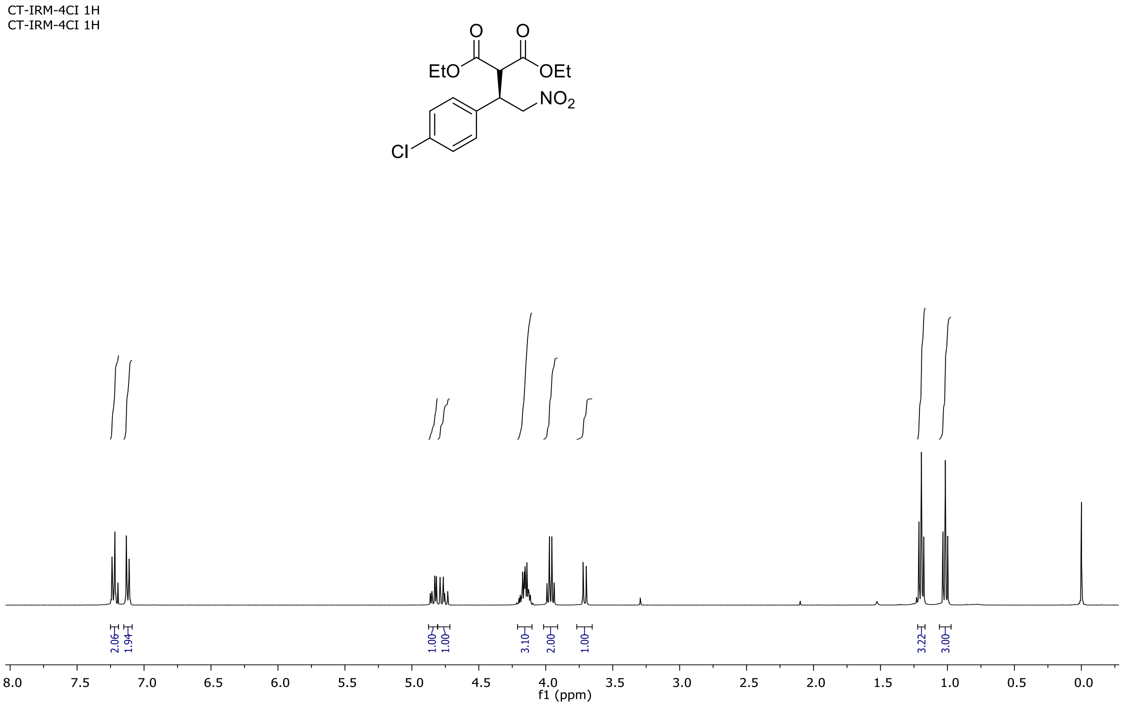

Figure S2.16 | ^{1}H NMR spectrum of chiral adduct **5f**

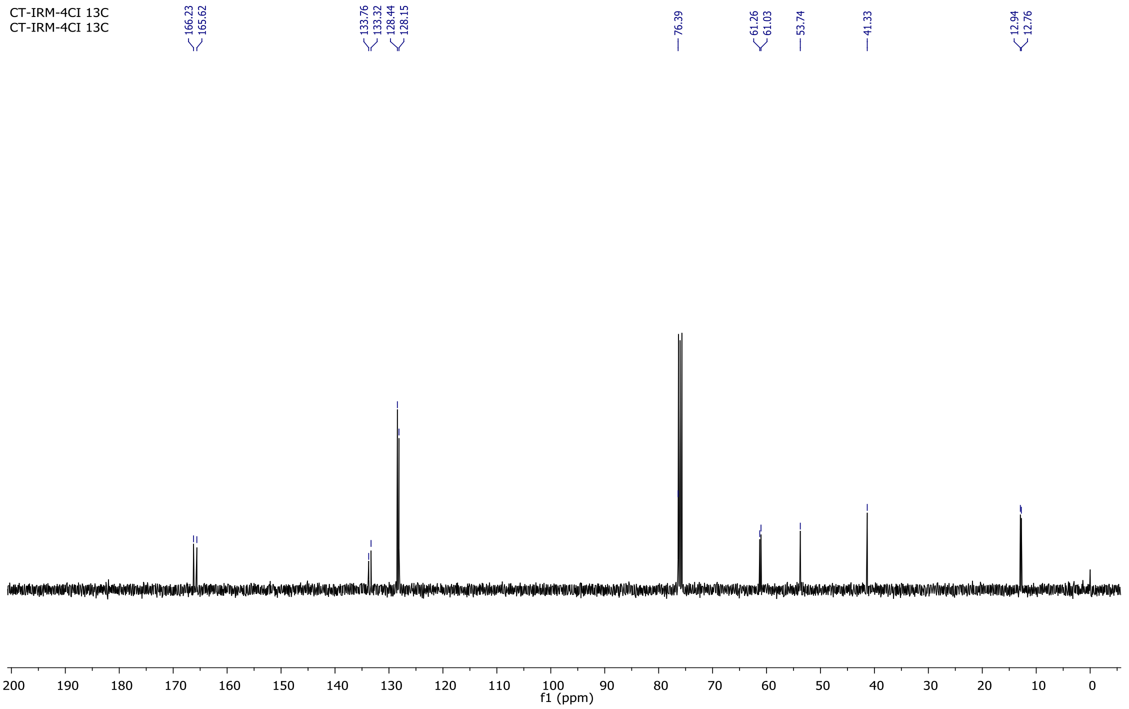

Figure S2.17 | ^{13}C NMR spectrum of chiral adduct **5f**

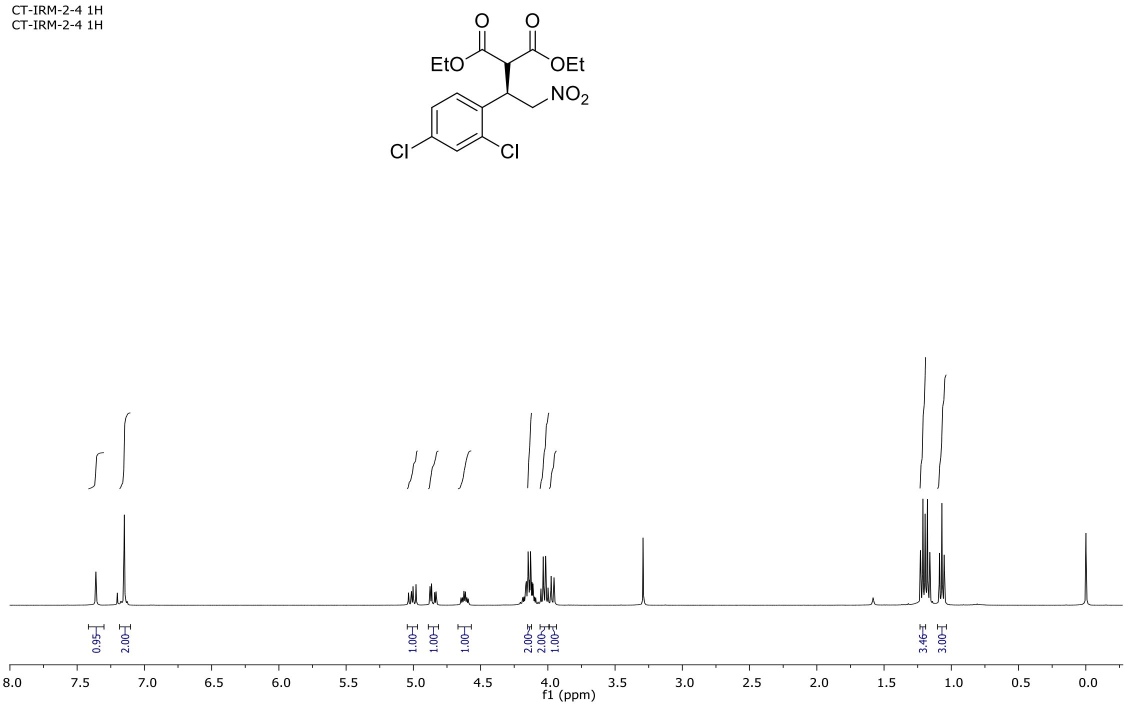

Figure S2.18 | ^{1}H NMR spectrum of chiral adduct **5g**

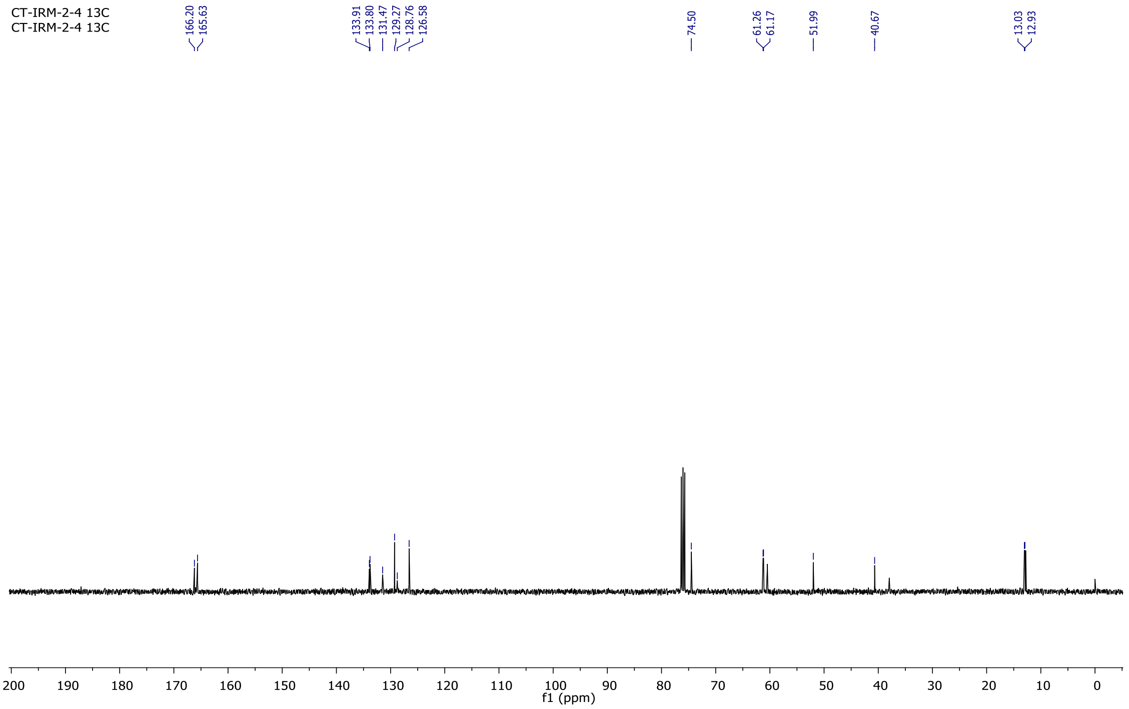

Figure S2.19 | ^{13}C NMR spectrum of chiral adduct **5g**

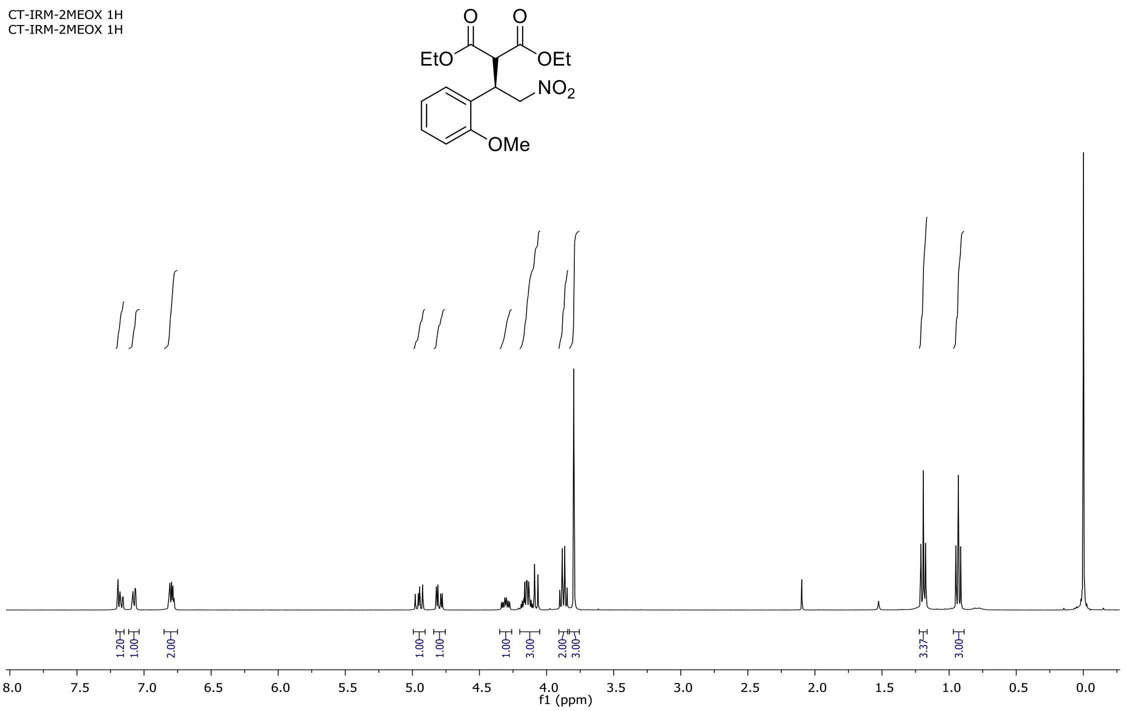

Figure S2.20 | ^{1}H NMR spectrum of chiral adduct **5h**

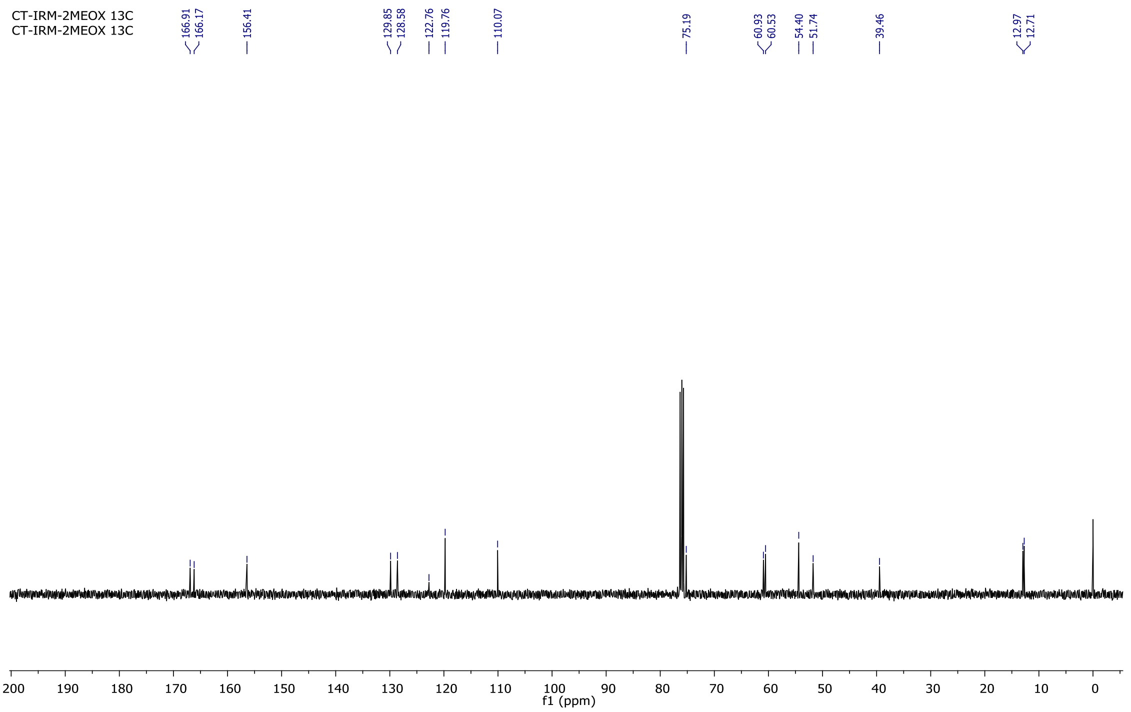

Figure S2.21 | ^{13}C NMR spectrum of chiral adduct **5h**

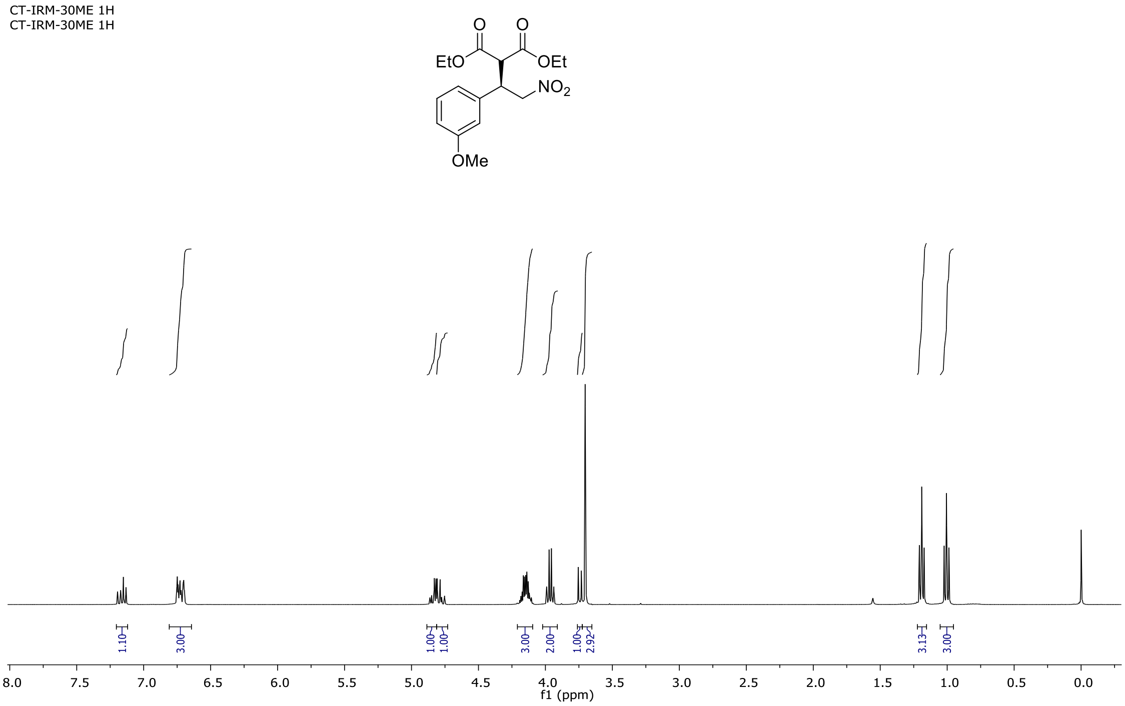

Figure S2.22 | ^{1}H NMR spectrum of chiral adduct **5i**

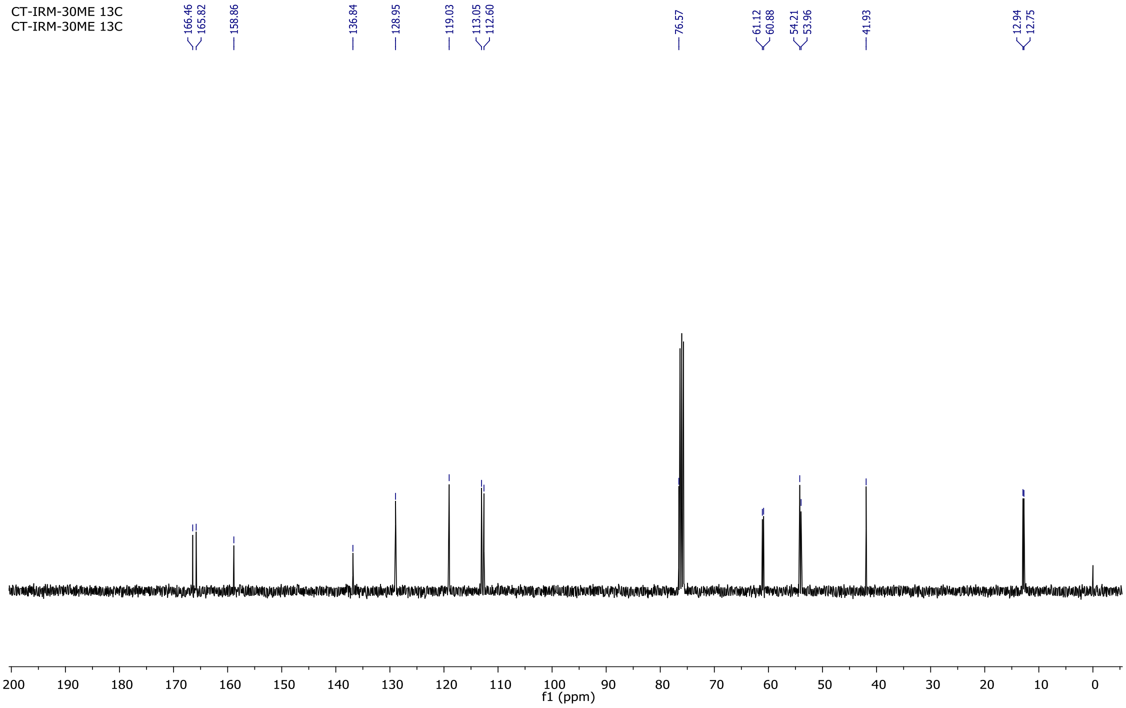

Figure S2.23 | ^{13}C NMR spectrum of chiral adduct **5i**

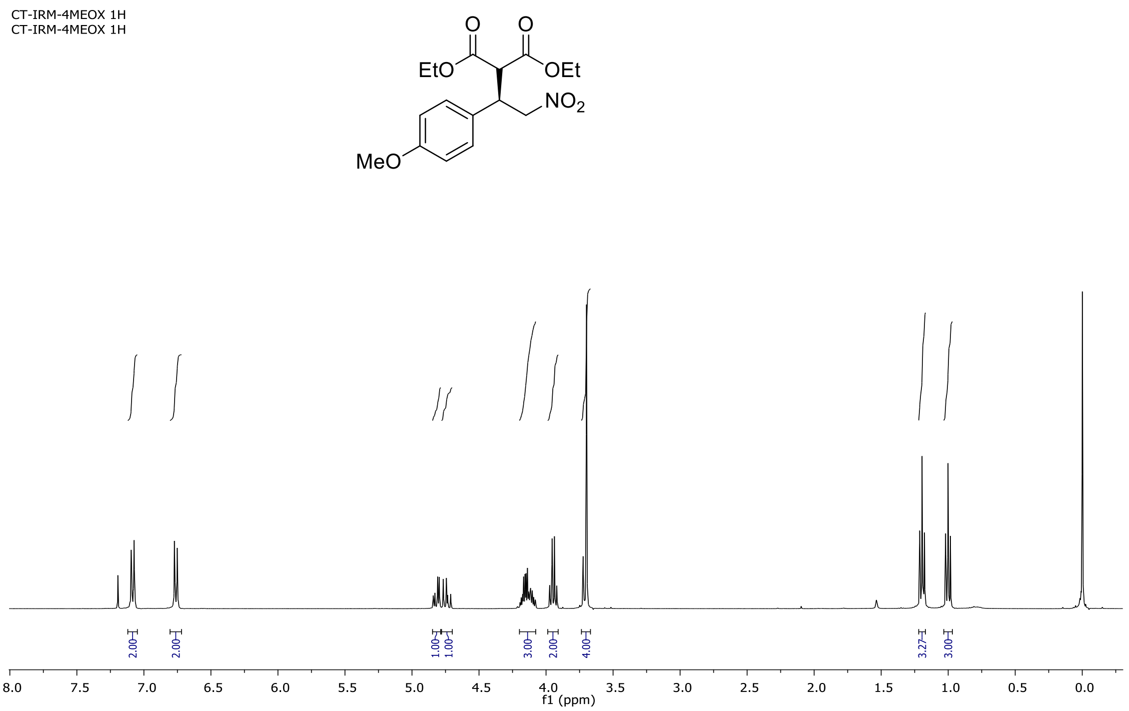

Figure S2.24 | ^{1}H NMR spectrum of chiral adduct **5j**

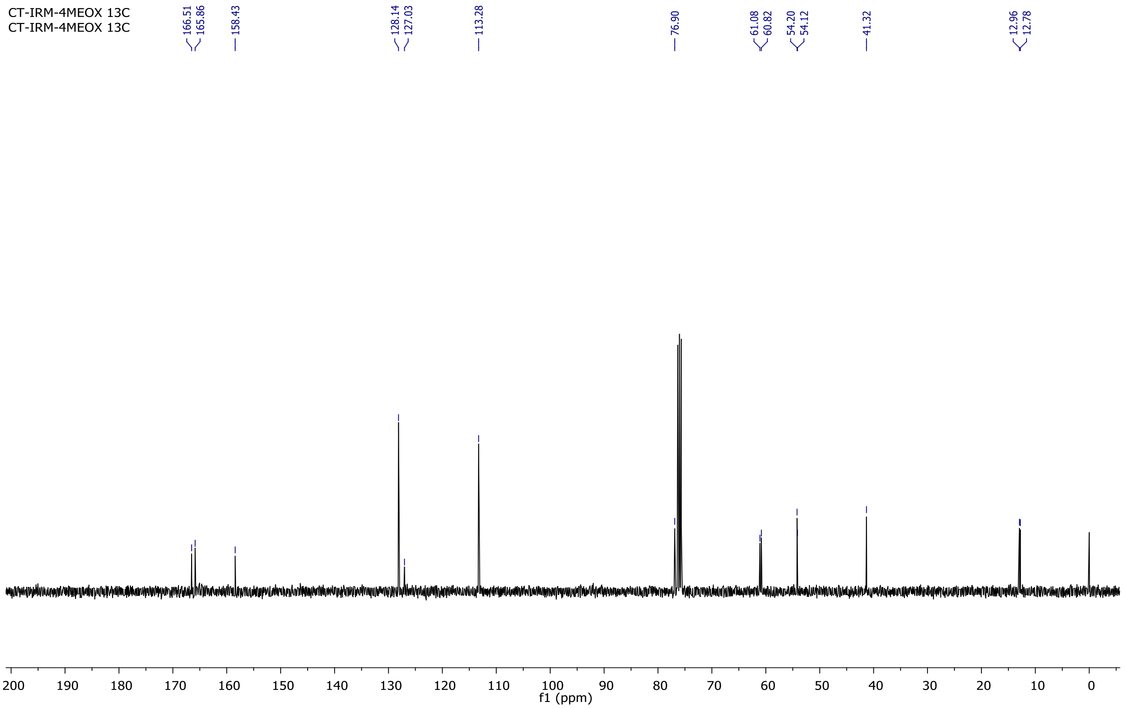

Figure S2.25 | ^{13}C NMR spectrum of chiral adduct **5j**

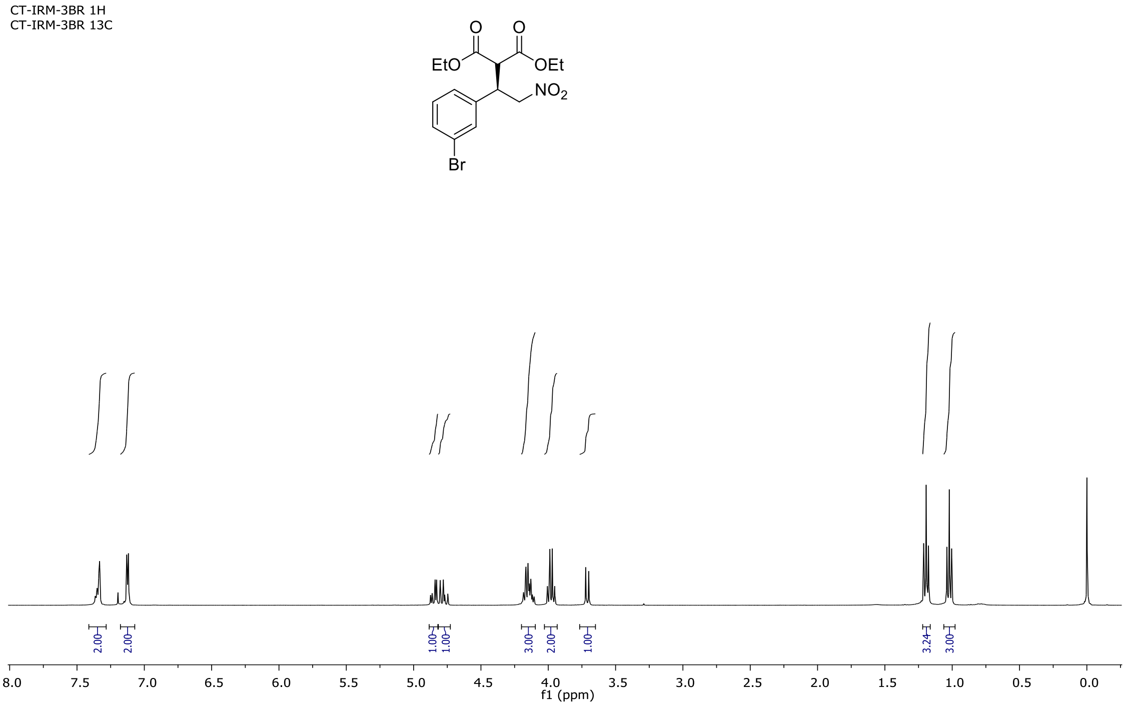

Figure S2.26 | ^{1}H NMR spectrum of chiral adduct **5k**

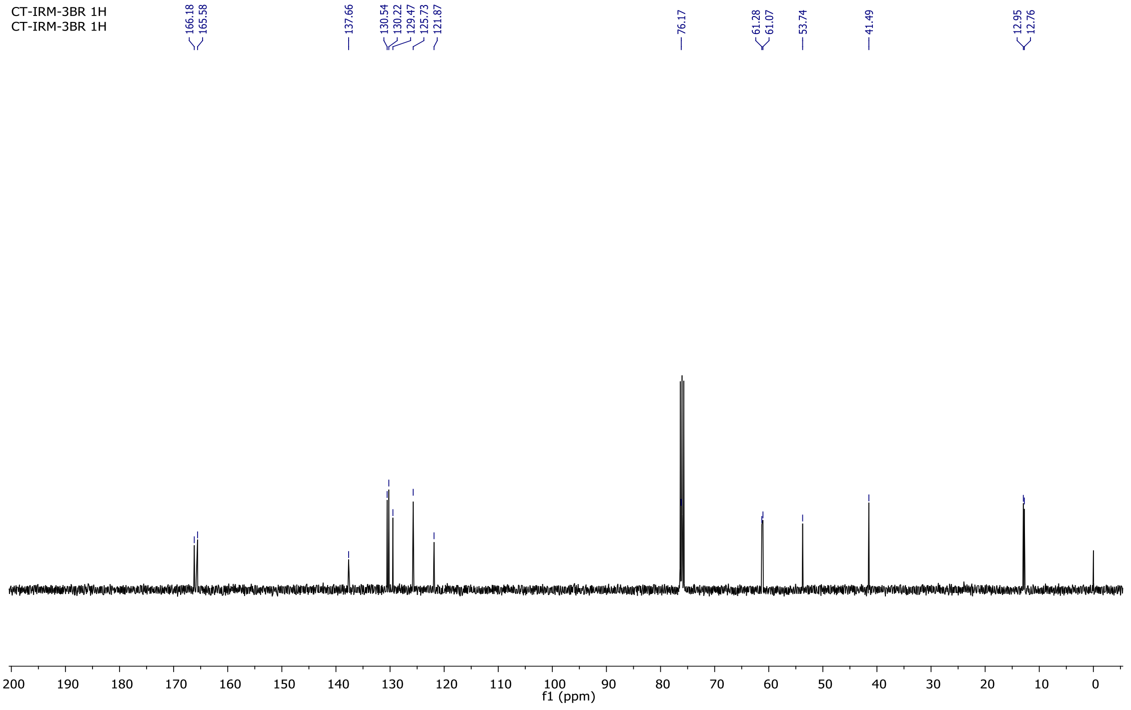

Figure S2.27 | ^{13}C NMR spectrum of chiral adduct **5k**

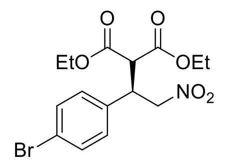

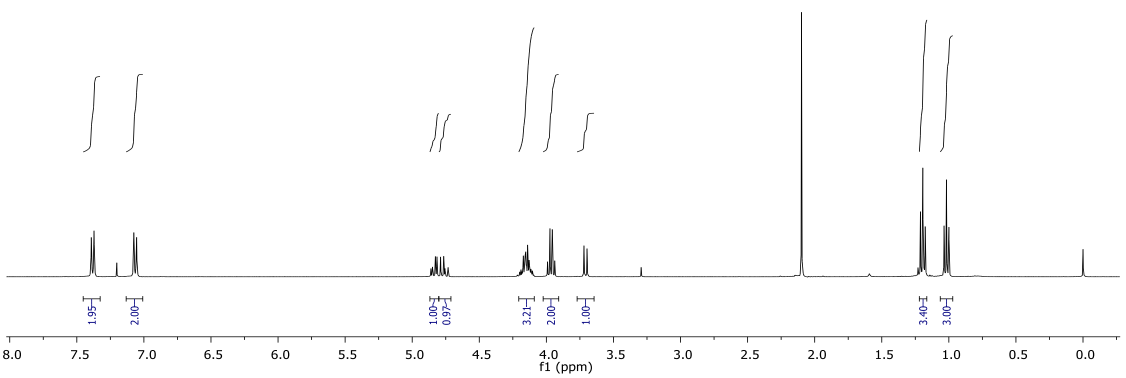

Figure S2.28 | ^{1}H NMR spectrum of chiral adduct **5l**

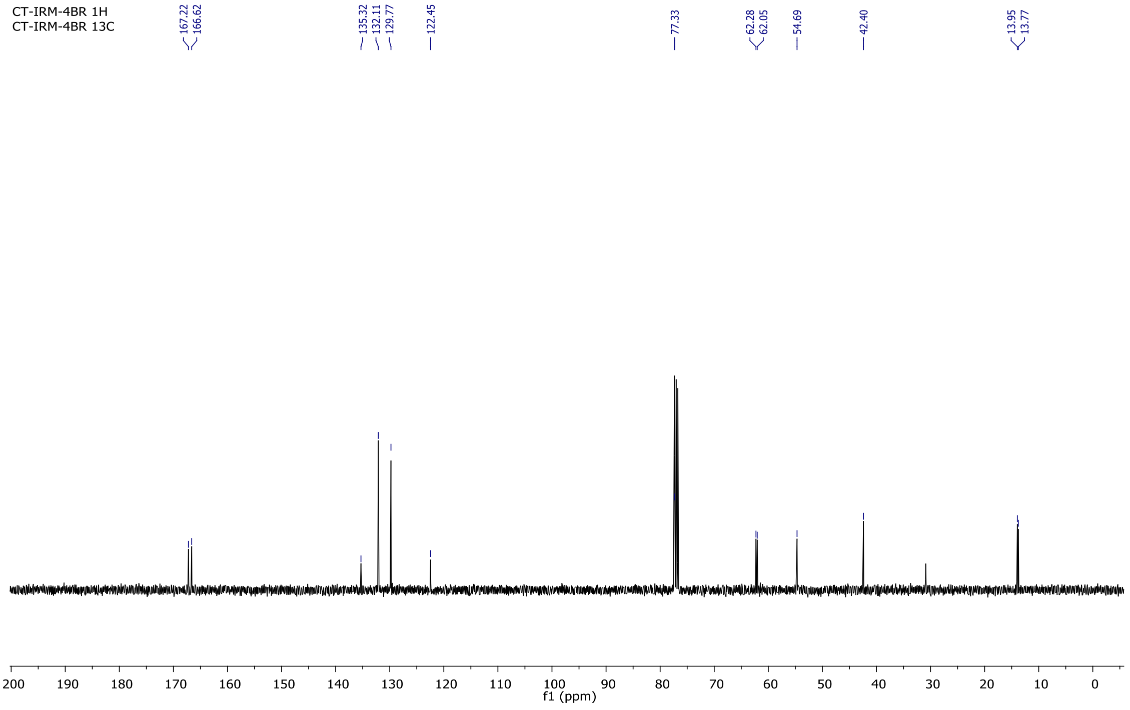

Figure S2.29 | ^{13}C NMR spectrum of chiral adduct **5l**

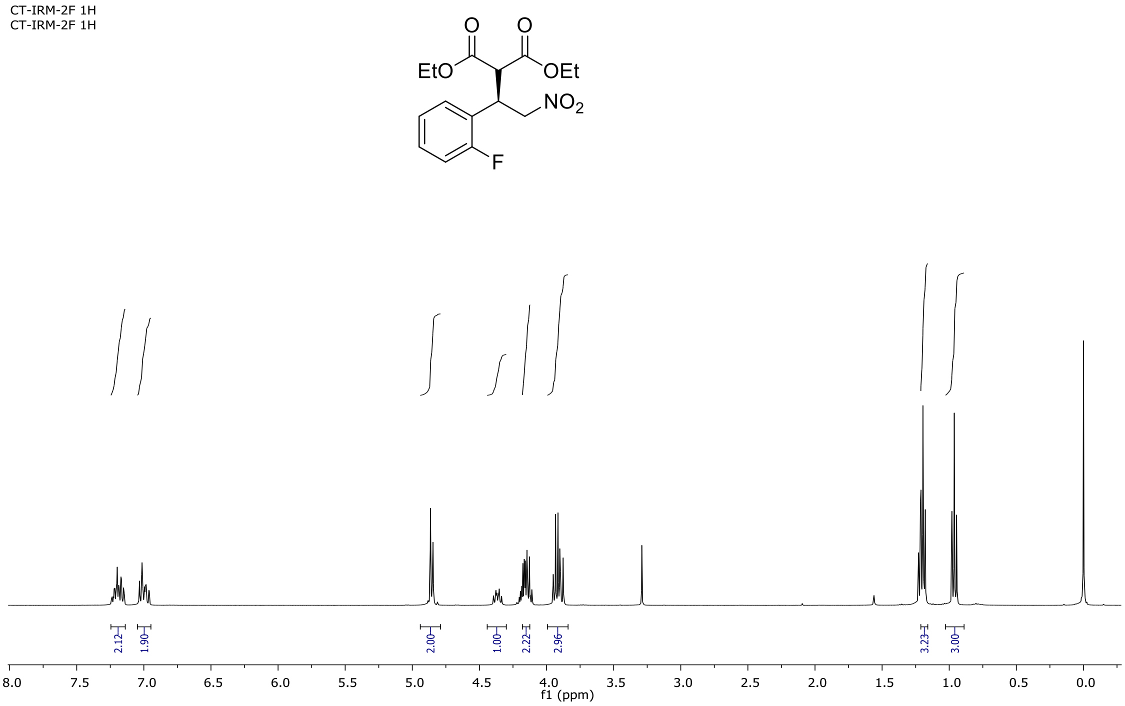

Figure S2.30 | ^{1}H NMR spectrum of chiral adduct **5m**

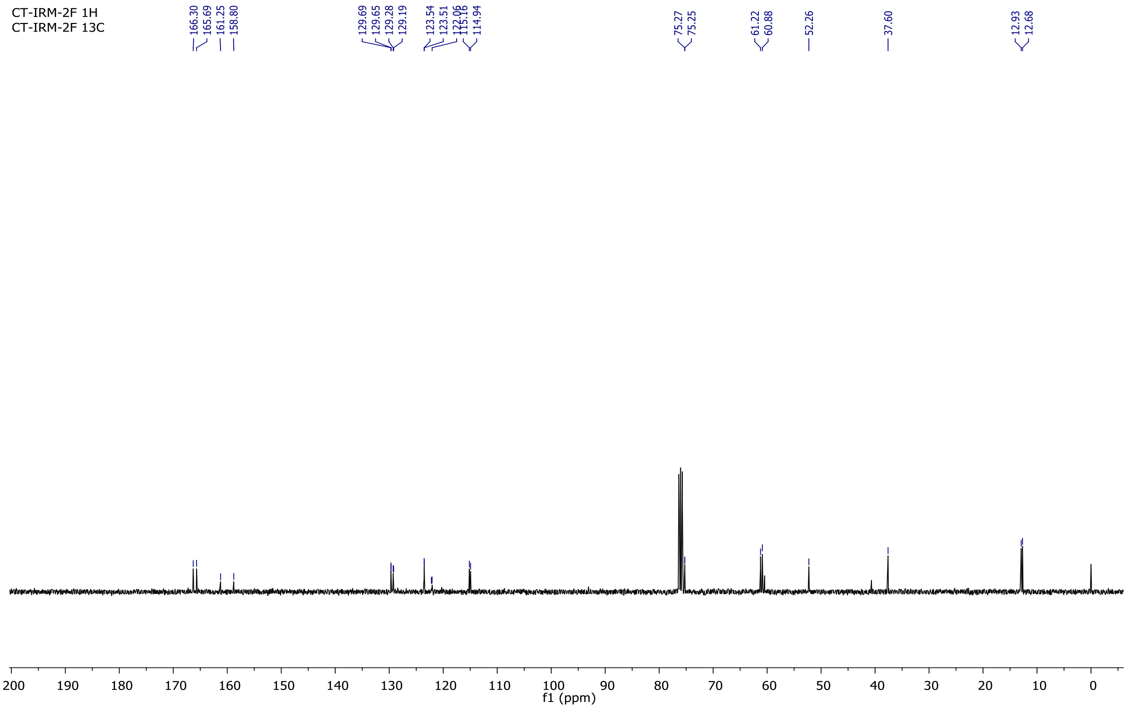

Figure S2.31 | ^{13}C NMR spectrum of chiral adduct **5m**

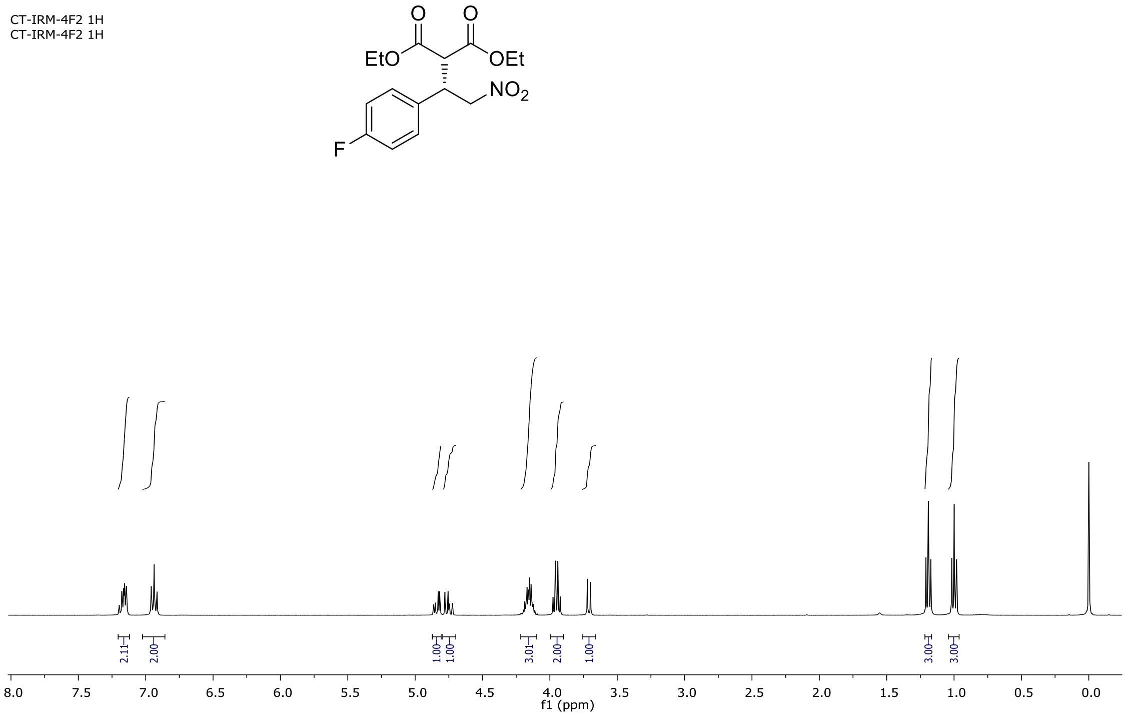

Figure S2.32 | ^{1}H NMR spectrum of chiral adduct **5n**

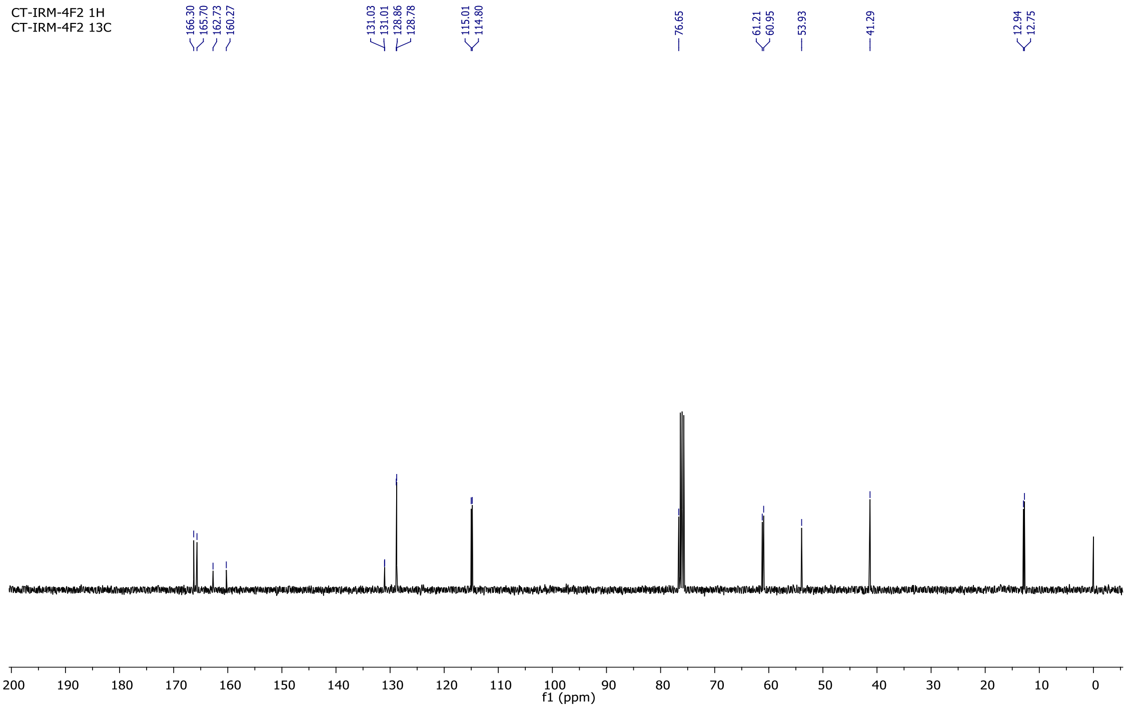

Figure S2.33 | ^{13}C NMR spectrum of chiral adduct **5n**

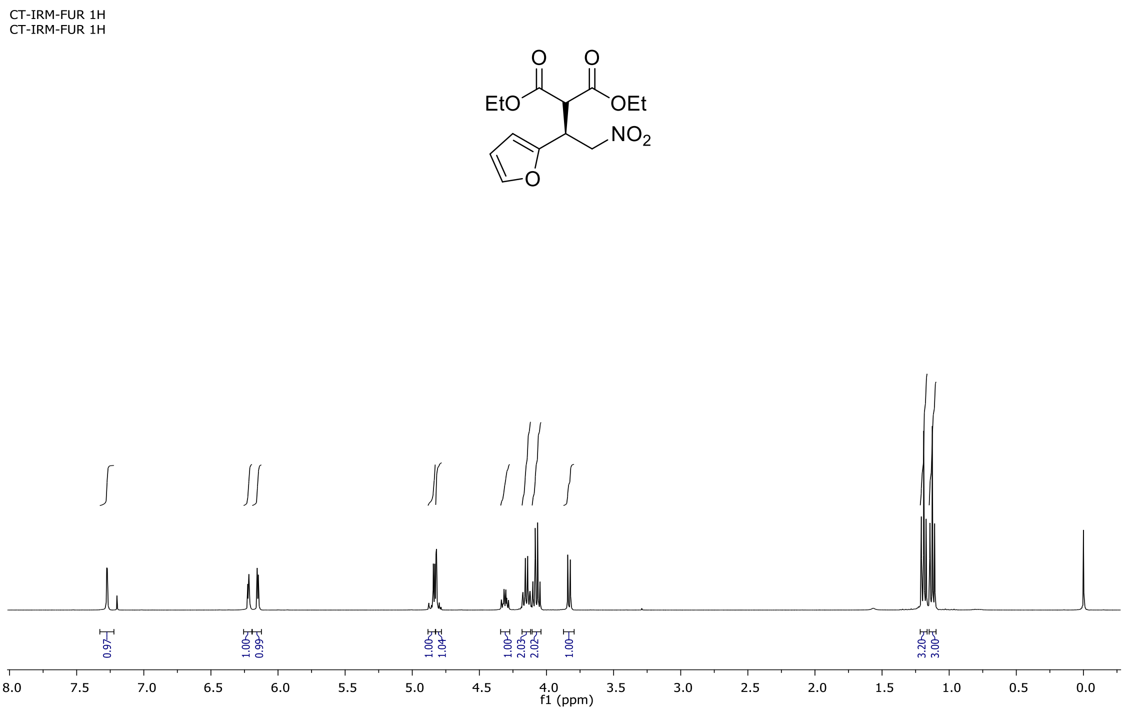

Figure S2.34 | ^{1}H NMR spectrum of chiral adduct **5o**

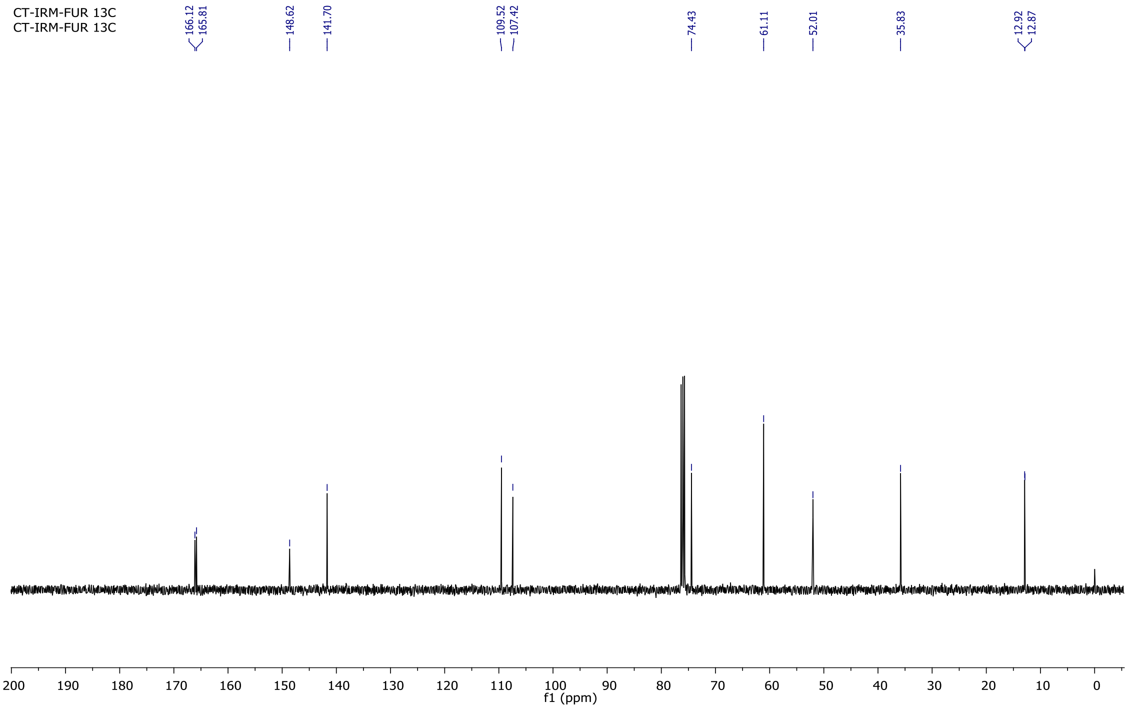

Figure S2.35 | ^{13}C NMR spectrum of chiral adduct **5o**

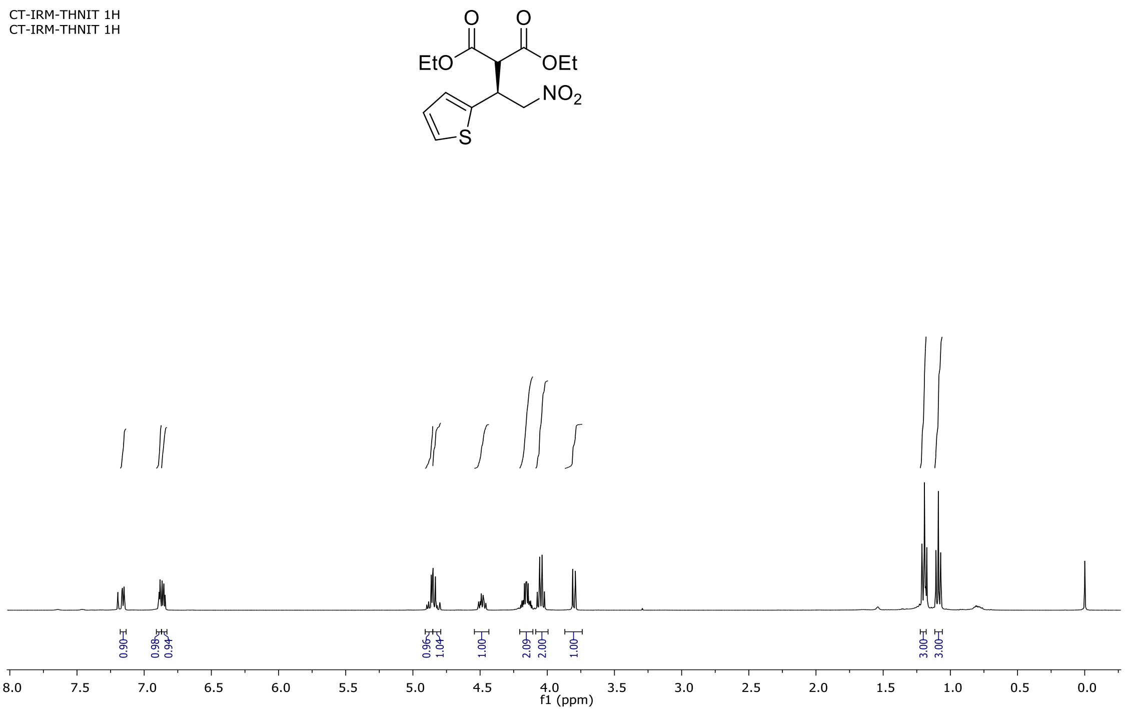

Figure S2.36 | ^{1}H NMR spectrum of chiral adduct **5p**

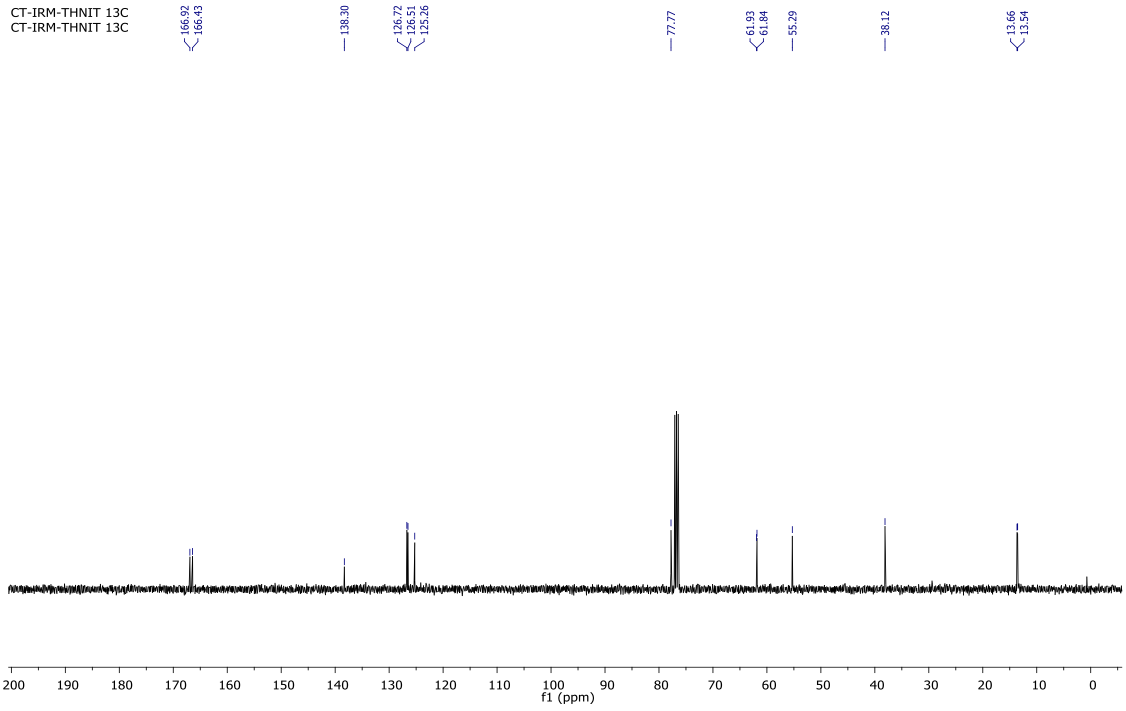

Figure S2.37 | ^{13}C NMR spectrum of chiral adduct **5p**

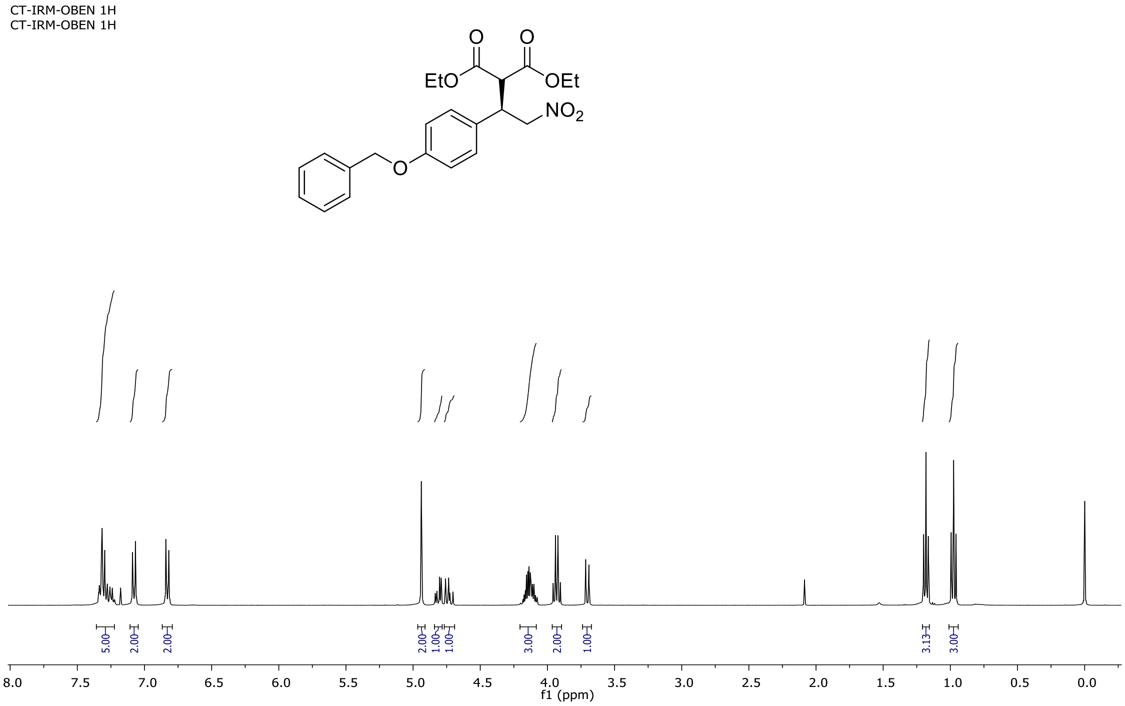

Figure S2.38 | ^{1}H NMR spectrum of chiral adduct **5r**

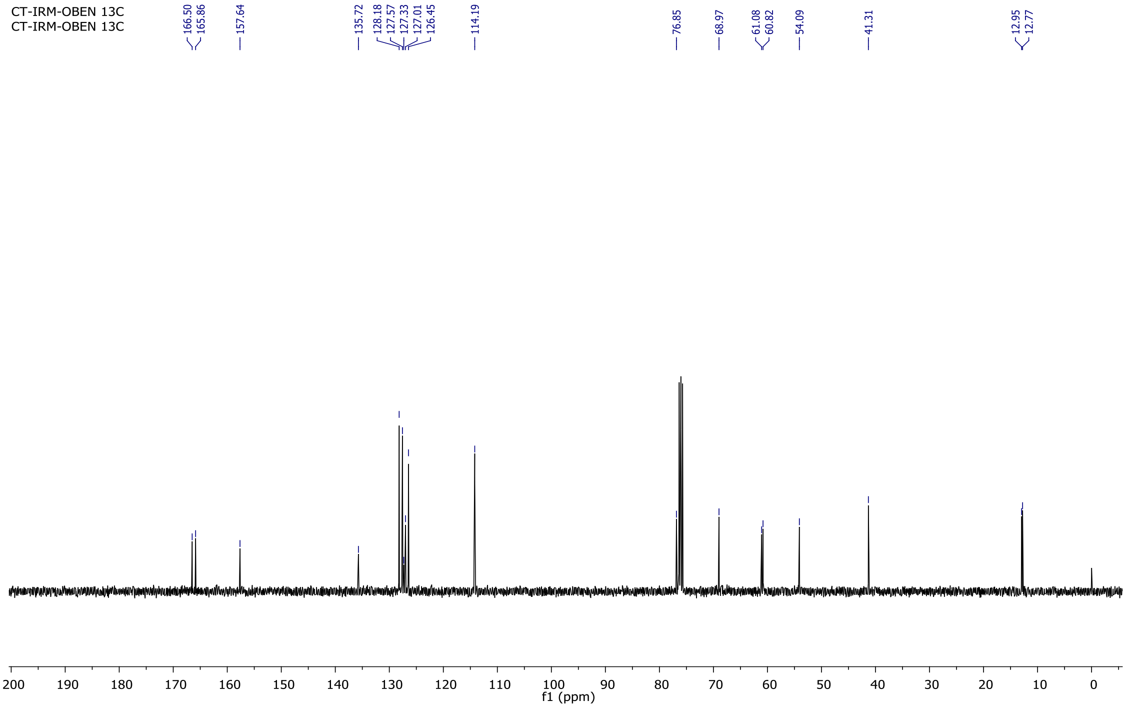

Figure S2.39 | ^{13}C NMR spectrum of chiral adduct **5r**

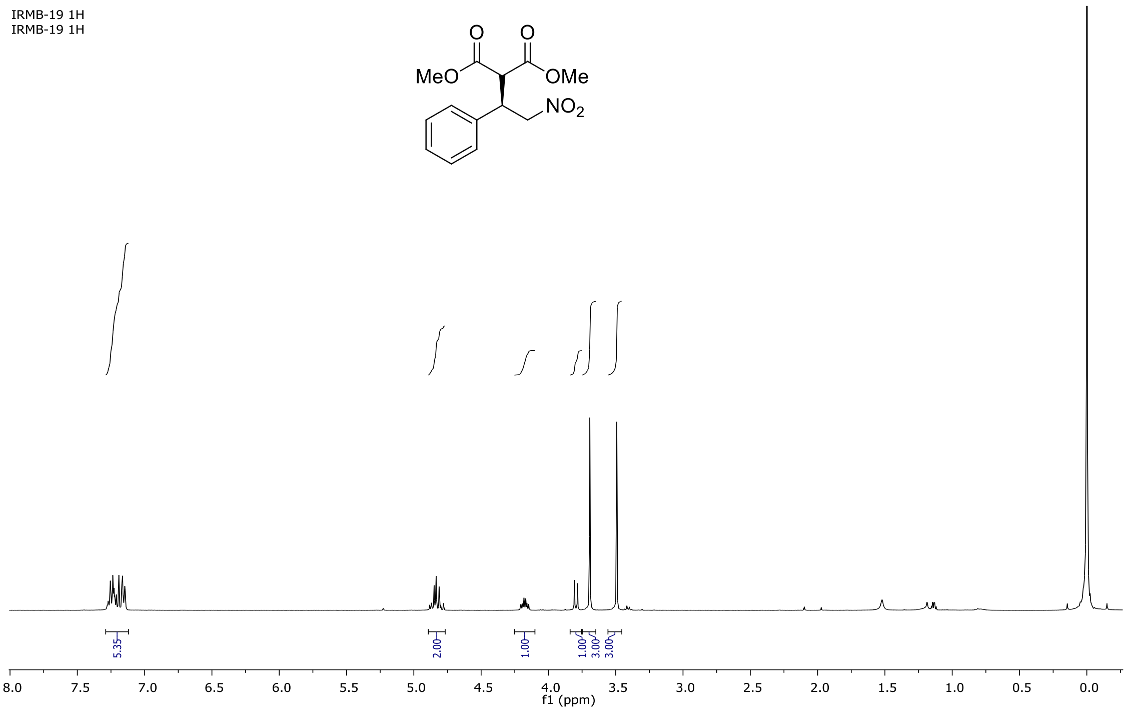

Figure S2.40 | ^{1}H NMR spectrum of chiral adduct **5ab**

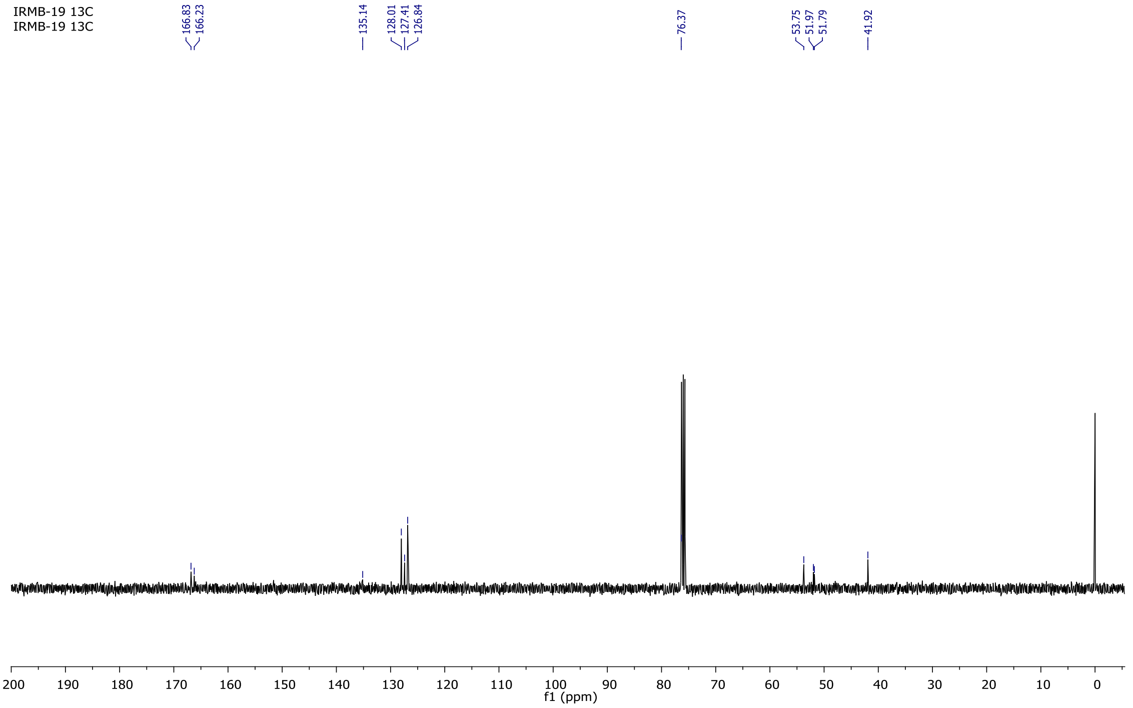

Figure S2.41 | ^{13}C NMR spectrum of chiral adduct **5ab**

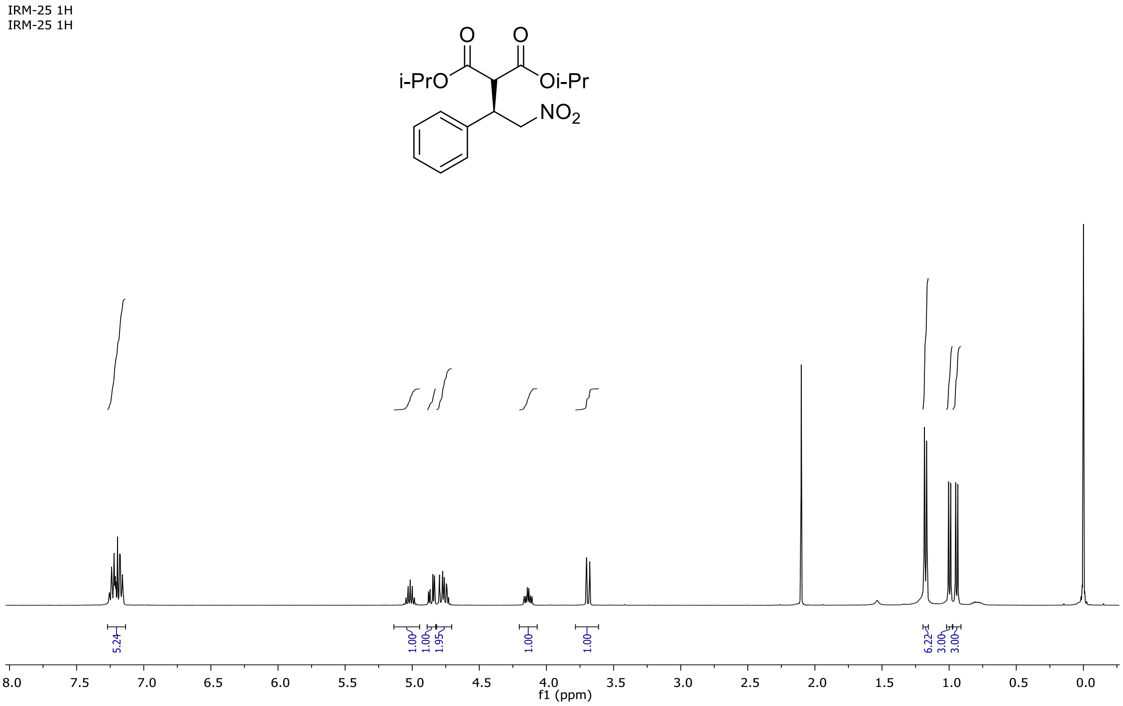

Figure S2.42 | ^{1}H NMR spectrum of chiral adduct **5ac**

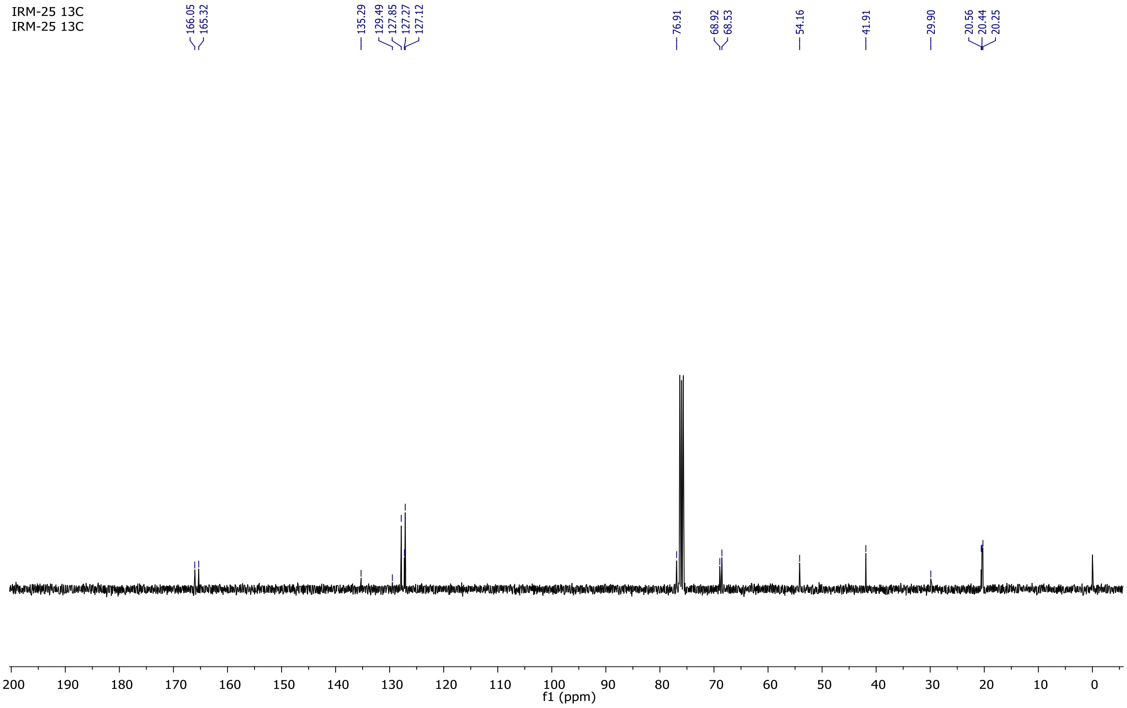

Figure S2.43 | ^{13}C NMR spectrum of chiral adduct **5ac**

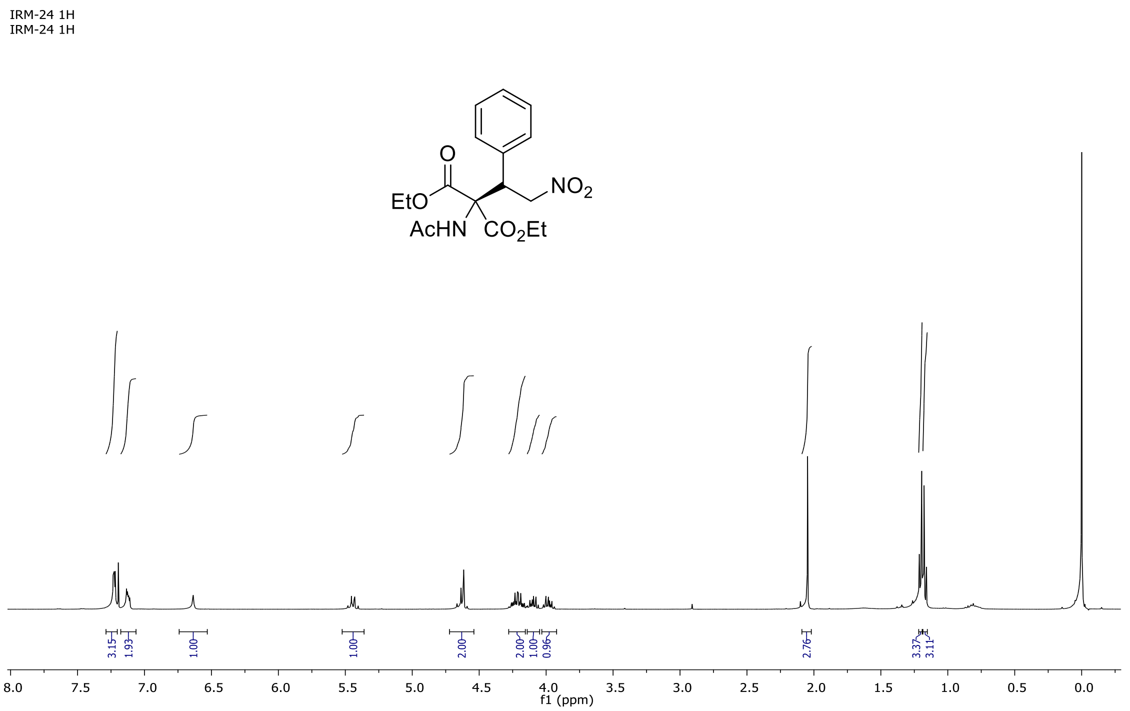

Figure S2.44 | ^{1}H NMR spectrum of chiral adduct **5ad**

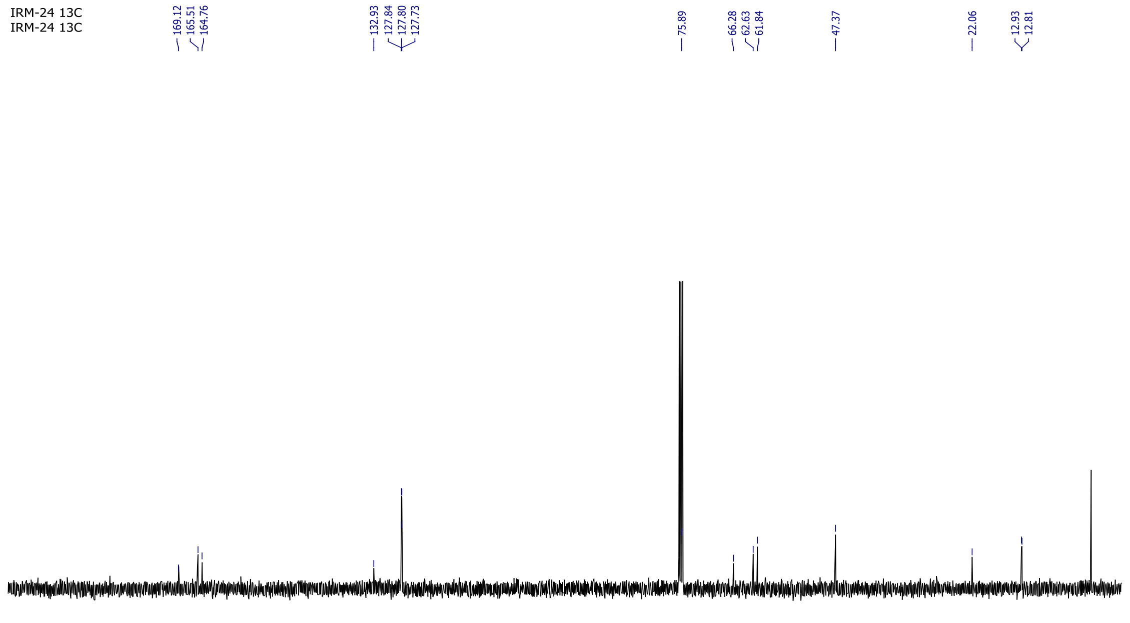

Figure S2.45 | ^{13}C NMR spectrum of chiral adduct **5ad**

2.6. COPIES OF CHIRAL HPLC CHROMATOGRAMS

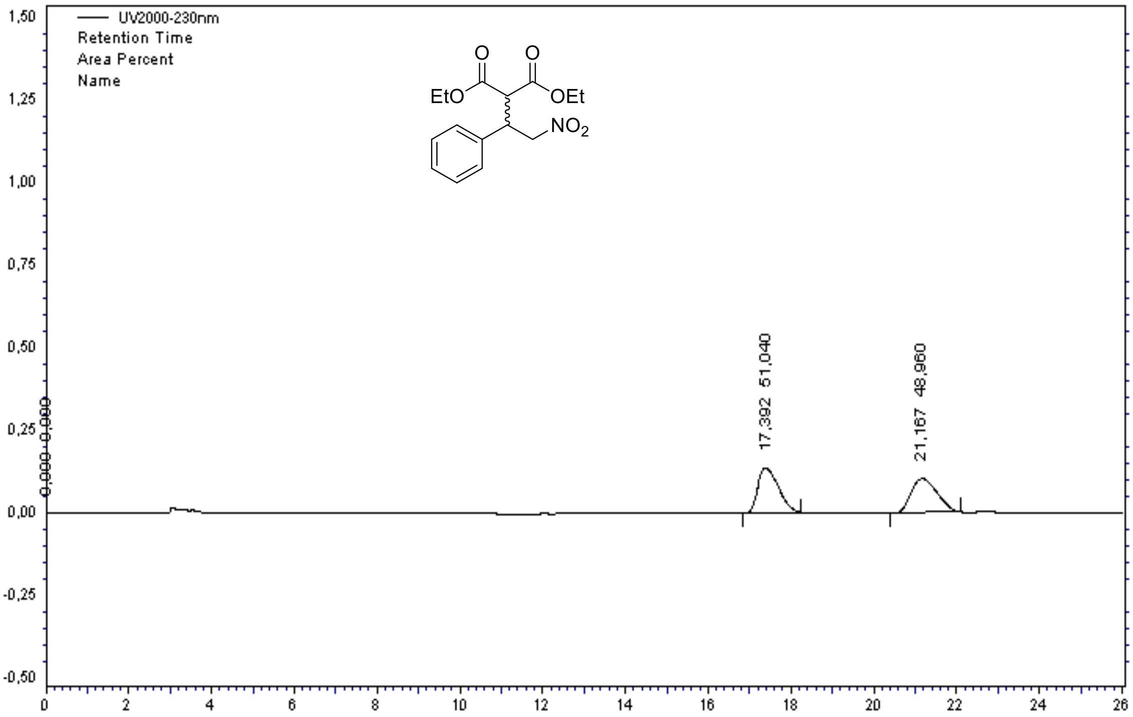

Figure S2.46 | HPLC chromatogram of *rac*-**5a**

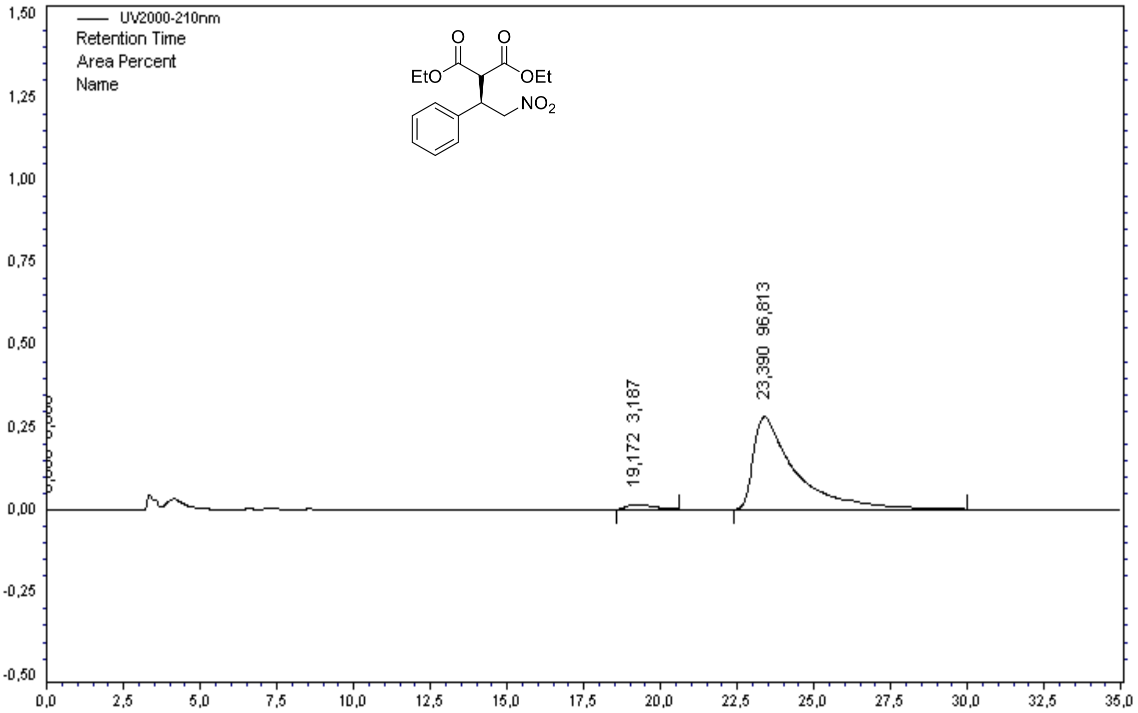

Figure S2.47 | HPLC chromatogram of *enantiomerically enriched adduct* **5a**

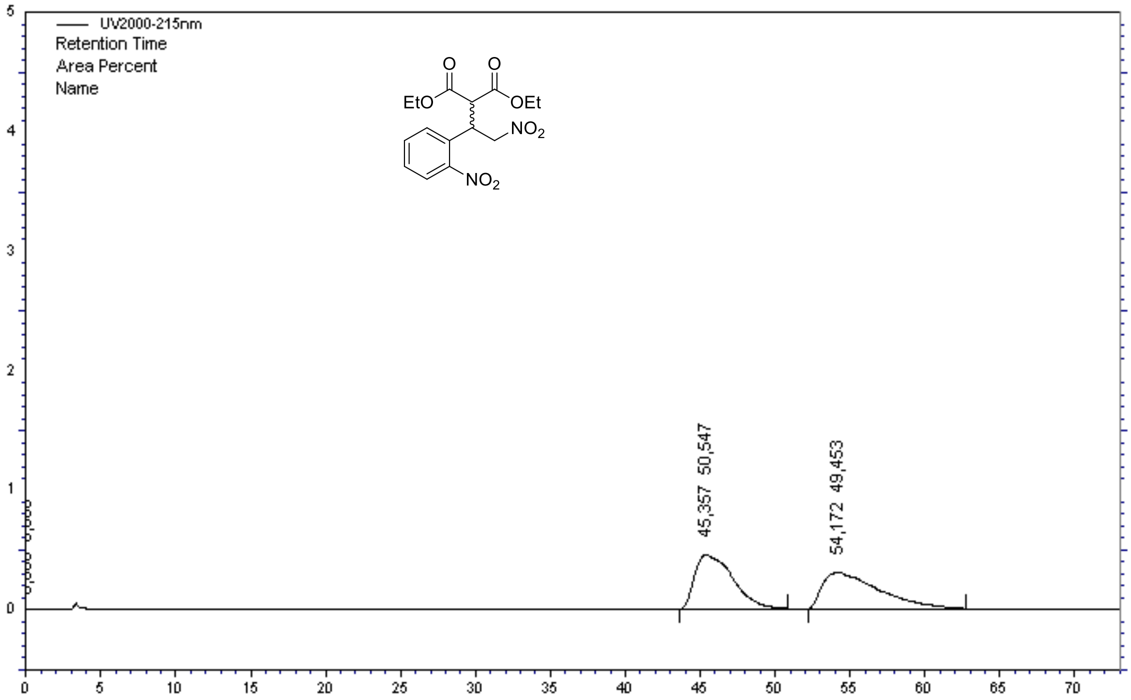

Figure S2.48 | HPLC chromatogram of *rac*-**5b**

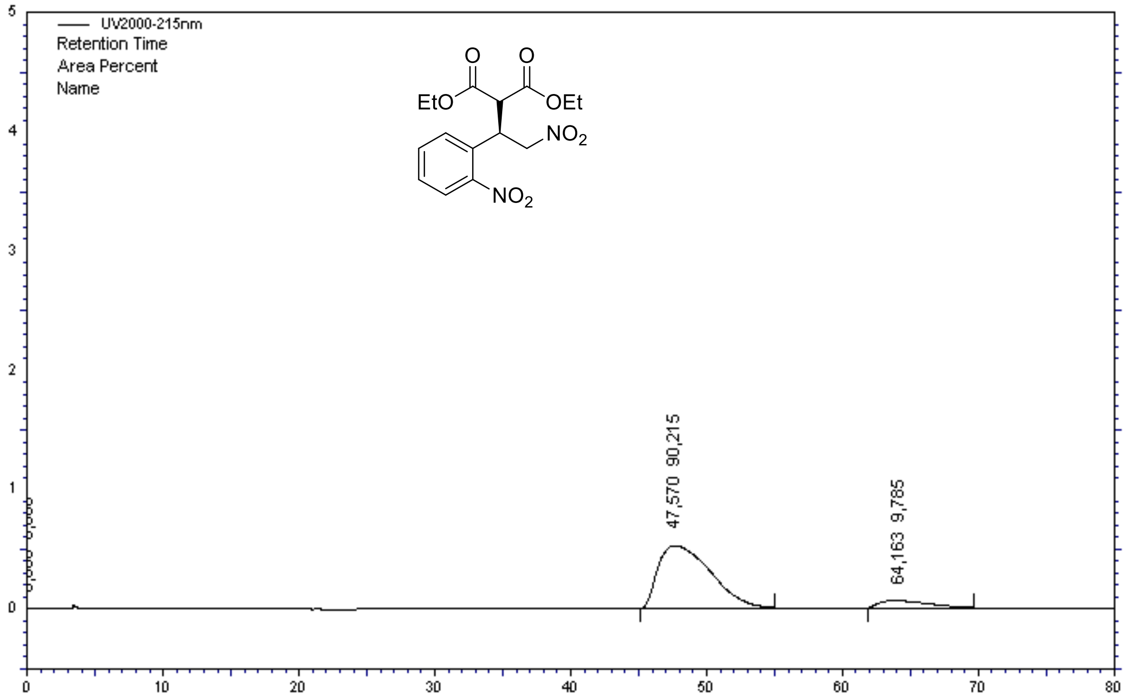

Figure S2.49 | HPLC chromatogram of *enantiomerically enriched adduct* **5b**

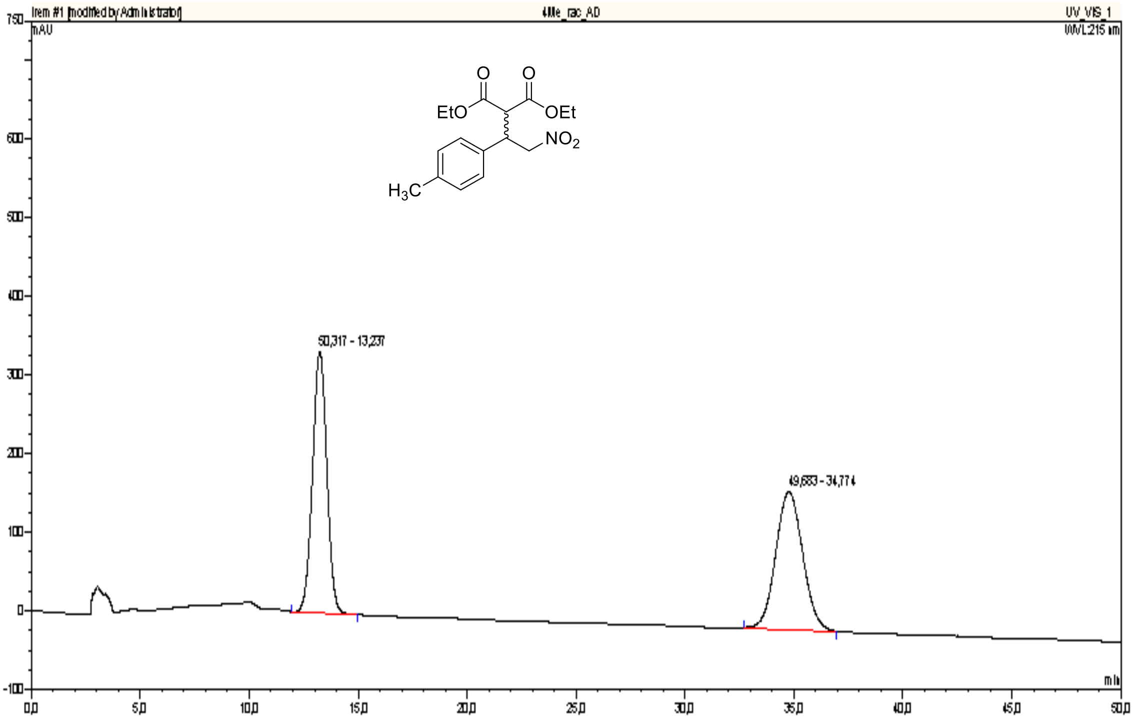

Figure S2.50 | HPLC chromatogram of *rac*-**5c**

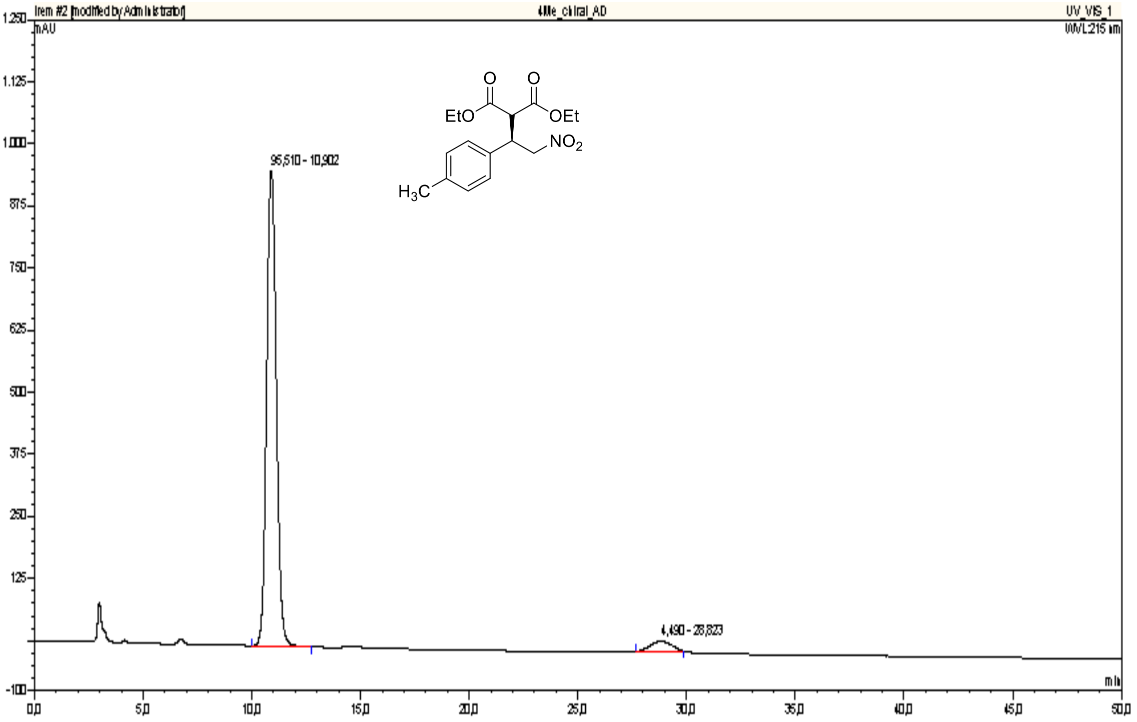

Figure S2.51 | HPLC chromatogram of *enantiomerically enriched adduct* **5c**

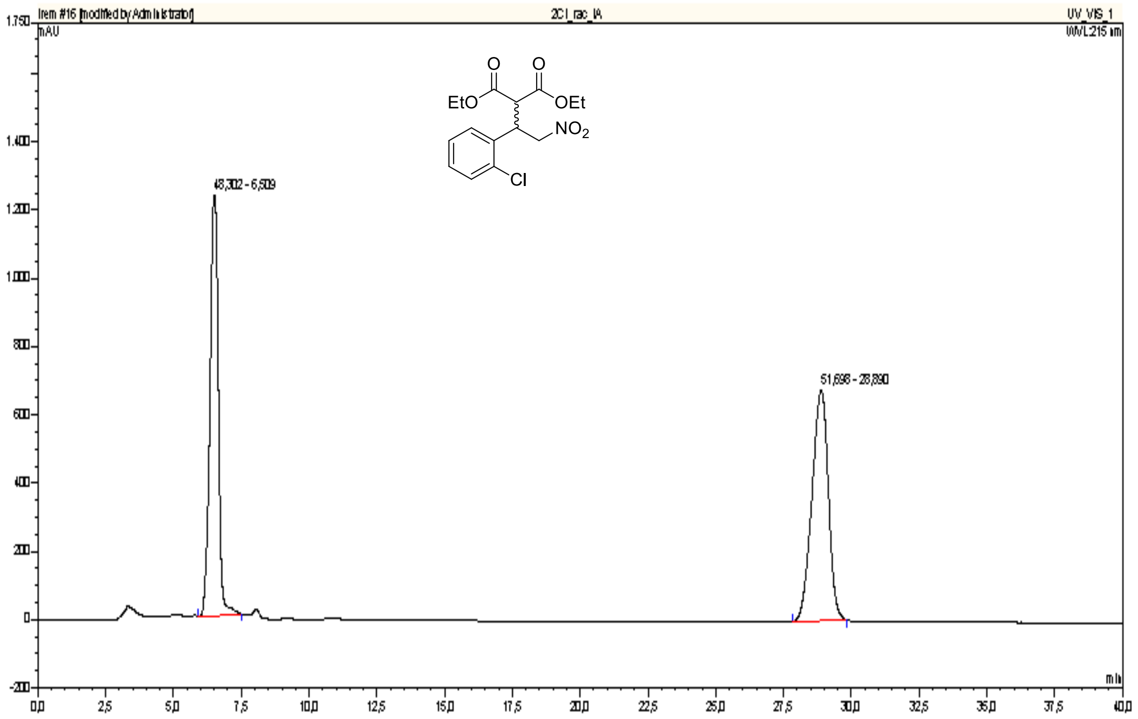

Figure S2.52 | HPLC chromatogram of *rac*-**5d**

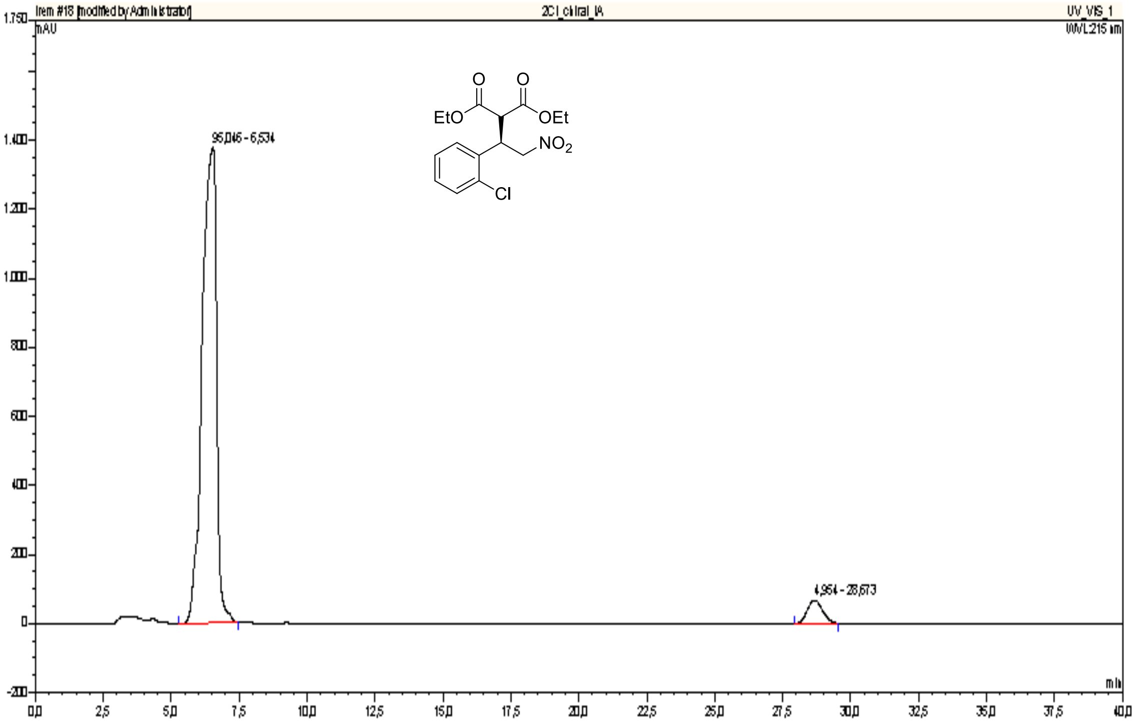

Figure S2.53 | HPLC chromatogram of *enantiomerically enriched adduct* **5d**

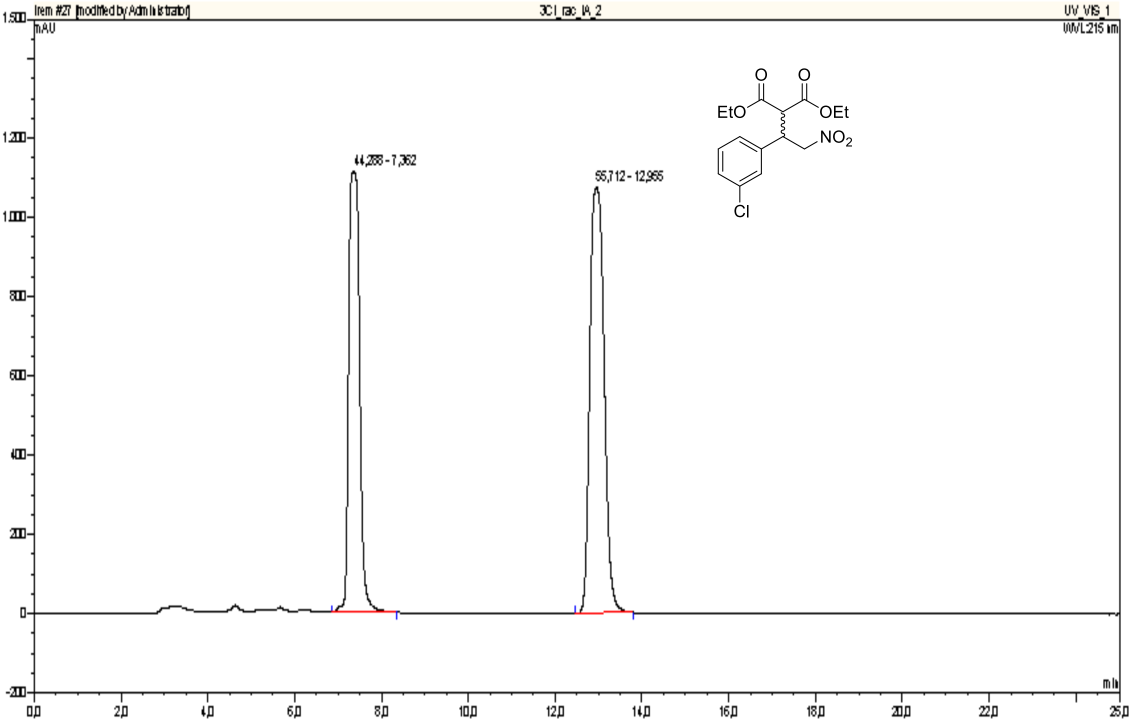

Figure S2.54 | HPLC chromatogram of *rac*-**5e**

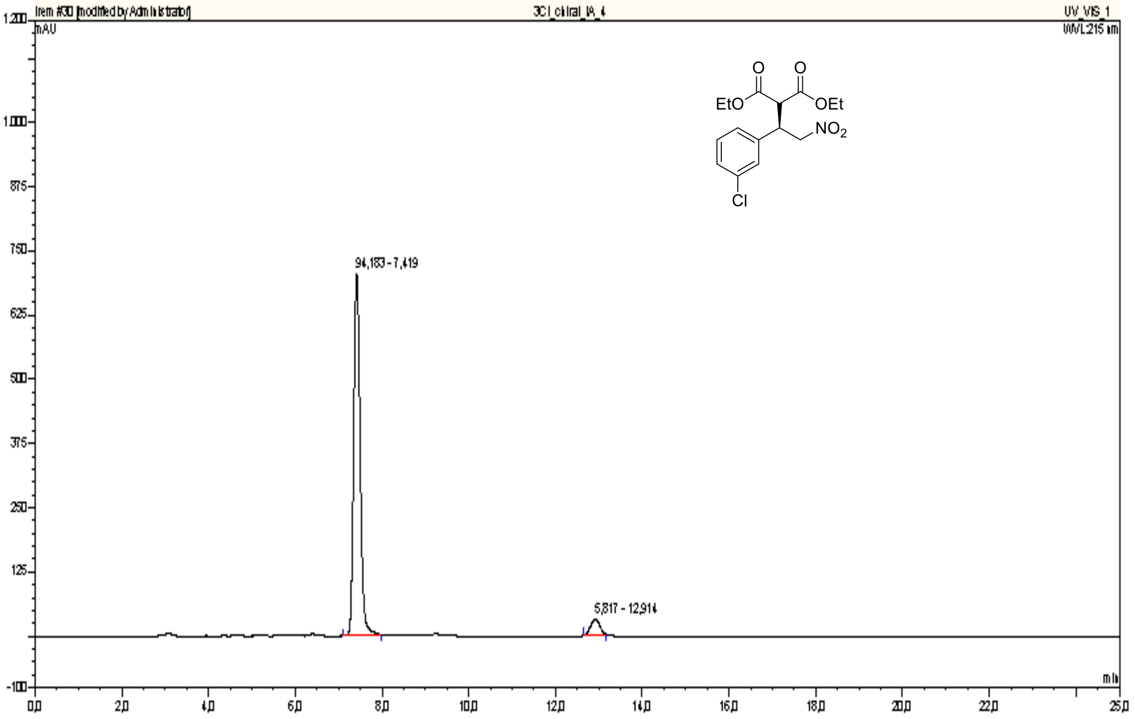

Figure S2.55 | HPLC chromatogram of *enantiomerically enriched adduct* **5e**

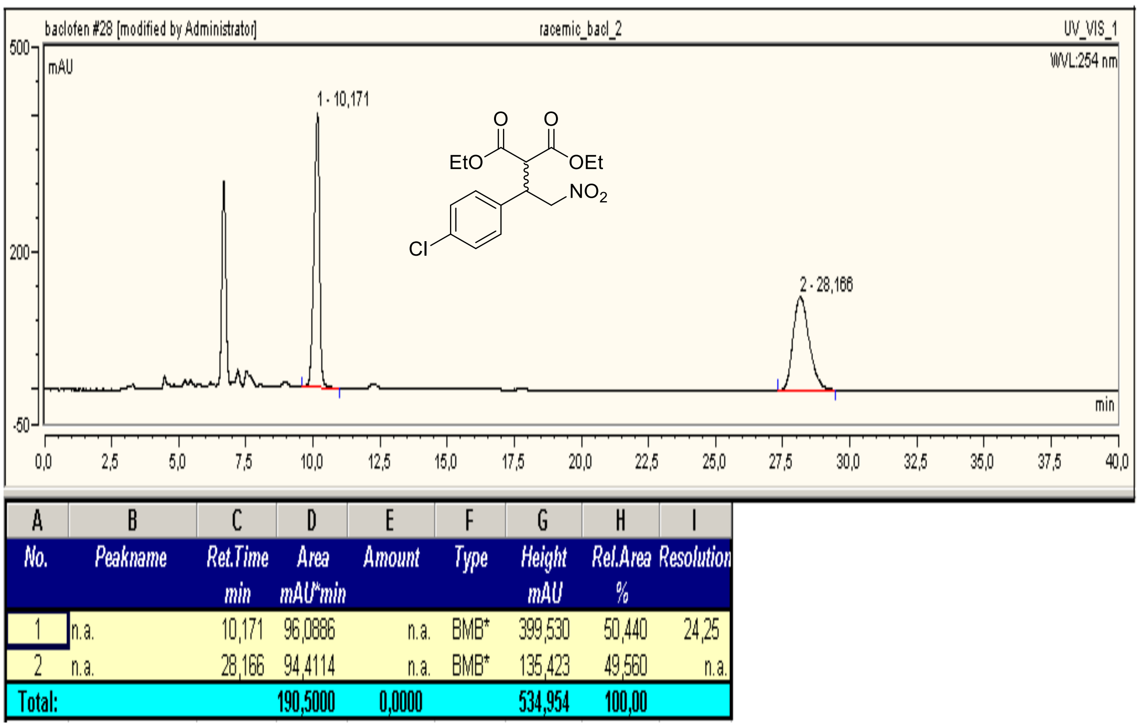

A	B	C	D	E	F	G	H	I
No.	Peakname	Ret.Time min	Area mAU*min	Amount	Type	Height mAU	Rel.Area %	Resolution
1	n.a.	10,171	96,0886	n.a.	BMB*	399,530	50,440	24,25
2	n.a.	28,166	94,4114	n.a.	BMB*	135,423	49,560	n.a.
Total:			190,5000	0,0000		534,954	100,00	

Figure S2.56 | HPLC chromatogram of *rac*-**5f**

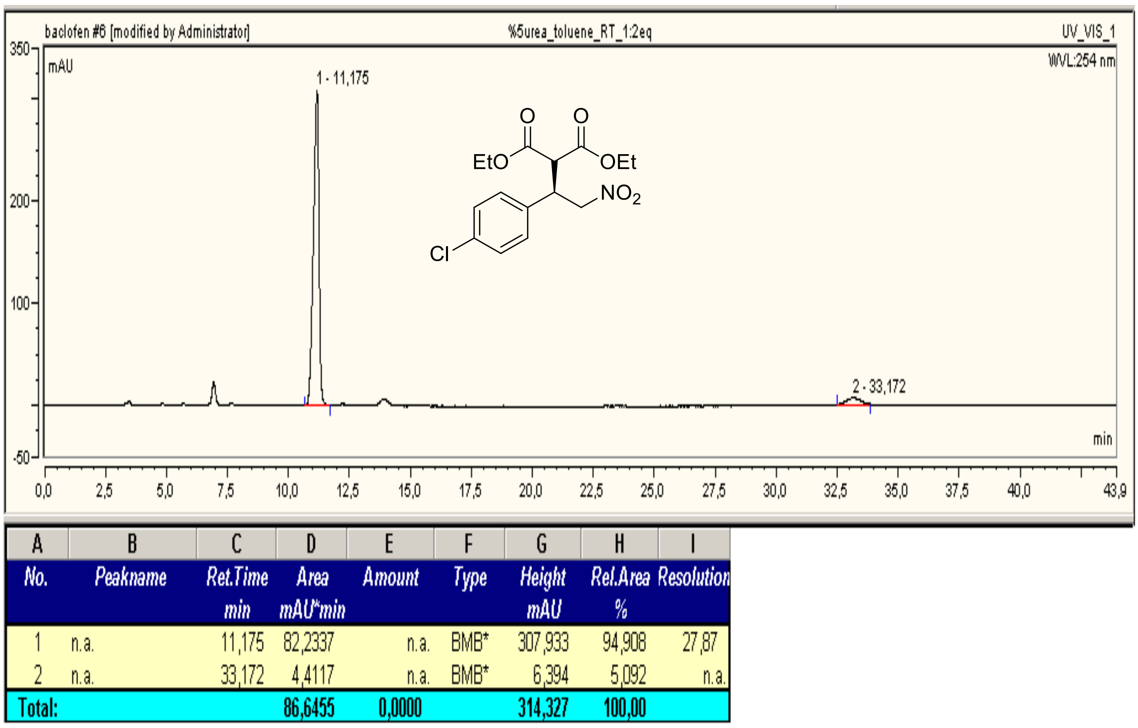

A	B	C	D	E	F	G	H	I
No.	Peakname	Ret.Time min	Area mAU*min	Amount	Type	Height mAU	Rel.Area %	Resolution
1	n.a.	11,175	82,2337	n.a.	BMB*	307,933	94,908	27,87
2	n.a.	33,172	4,4117	n.a.	BMB*	6,394	5,092	n.a.
Total:			86,6455	0,0000		314,327	100,00	

Figure S2.57 | HPLC chromatogram of *enantiomerically enriched adduct* **5f**

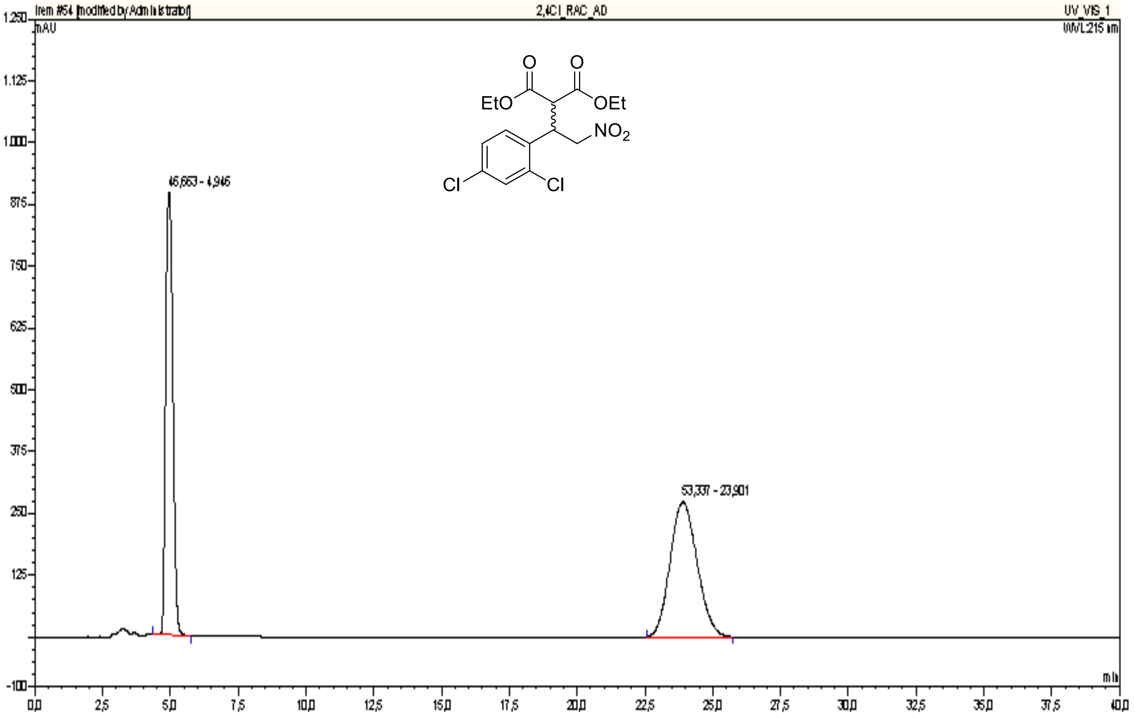

Figure S2.58 | HPLC chromatogram of *rac*-**5g**

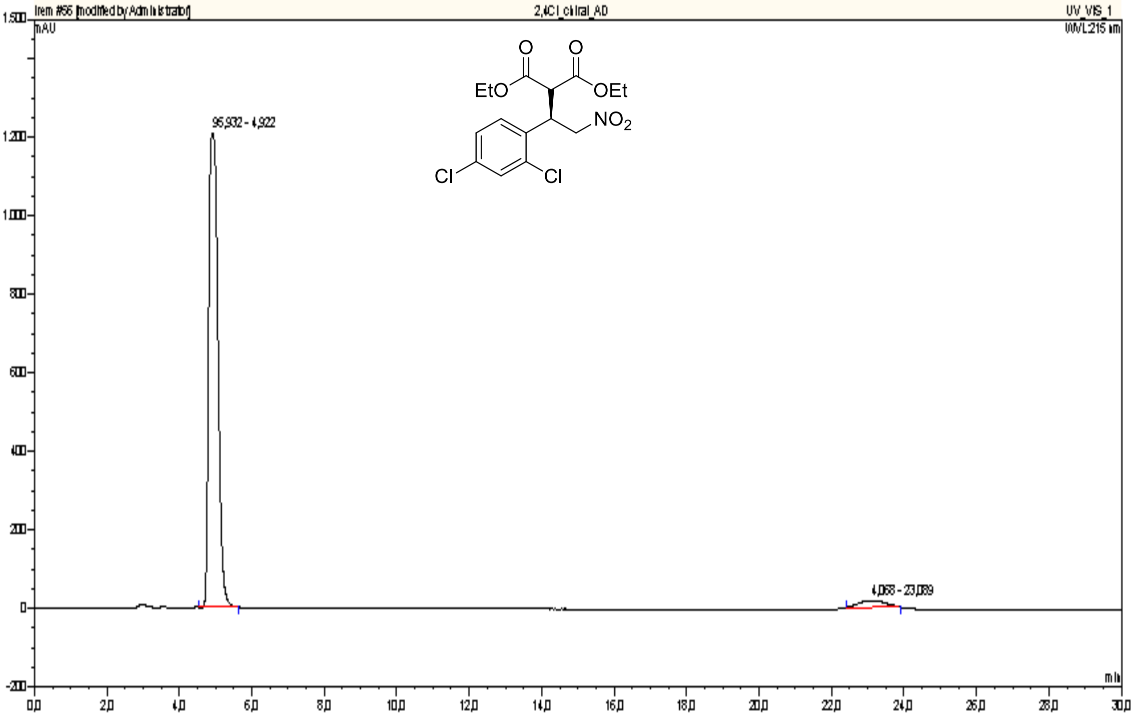

Figure S2.59 | HPLC chromatogram of *enantiomerically enriched adduct* **5g**

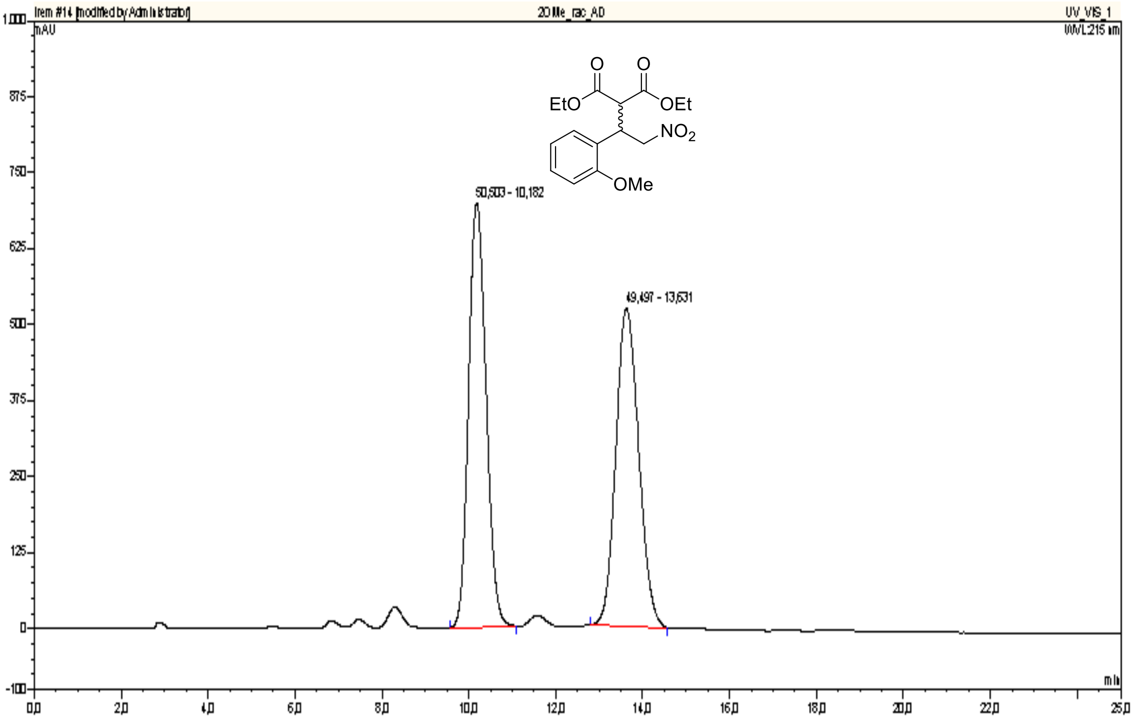

Figure S2.60 | HPLC chromatogram of *rac*-**5h**

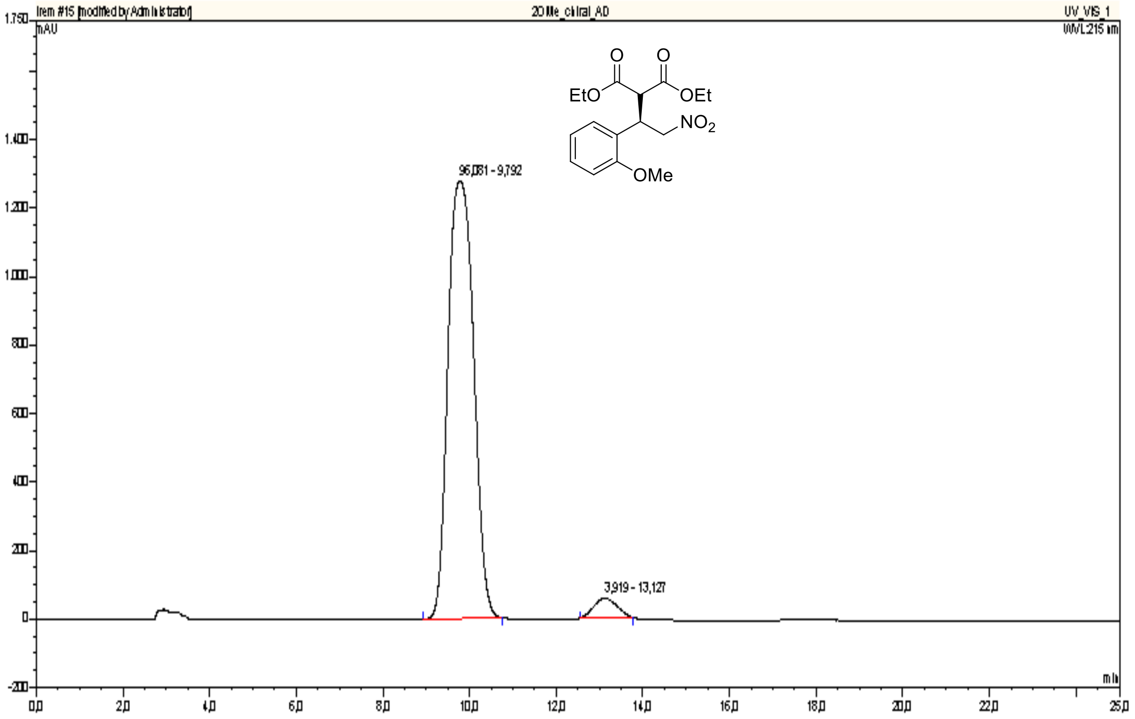

Figure S2.61 | HPLC chromatogram of *enantiomerically enriched adduct* **5h**

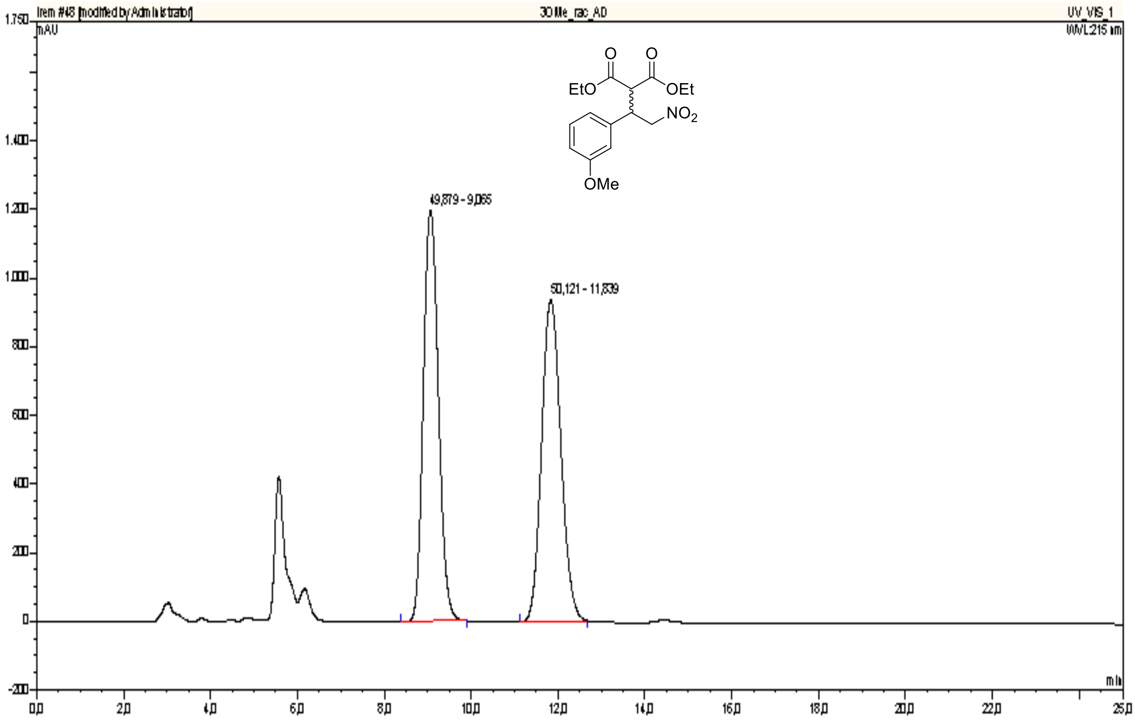

Figure S2.62 | HPLC chromatogram of *rac*-**5i**

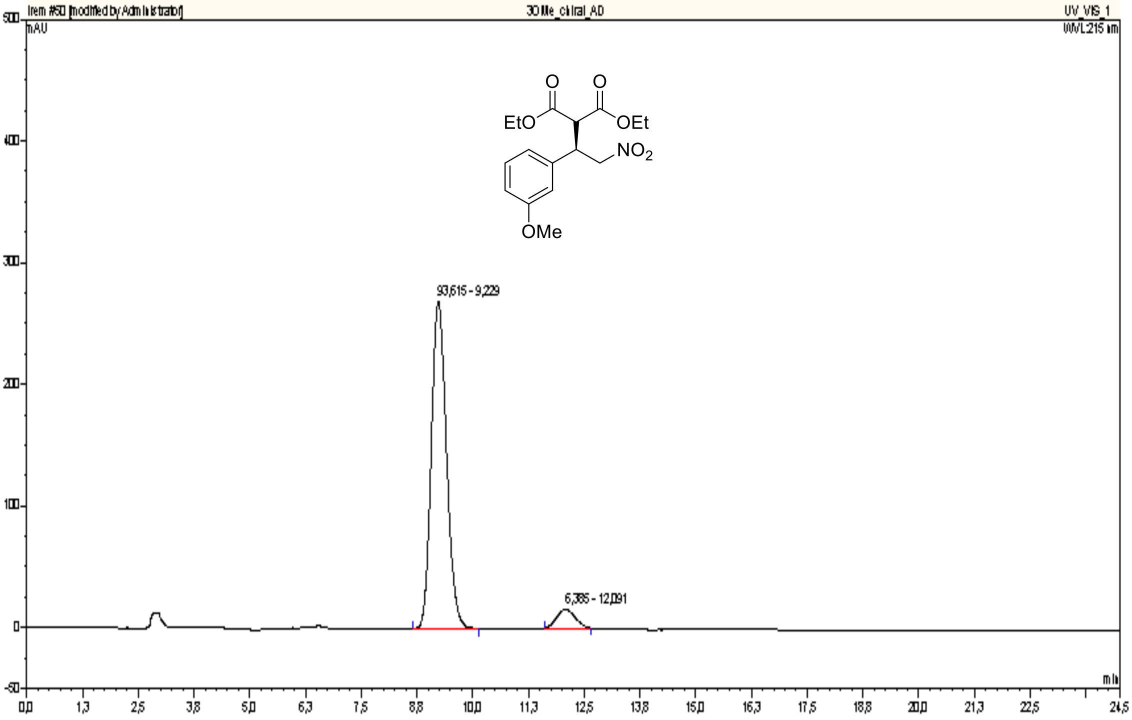

Figure S2.63| HPLC chromatogram of *enantiomerically enriched adduct* **5i**

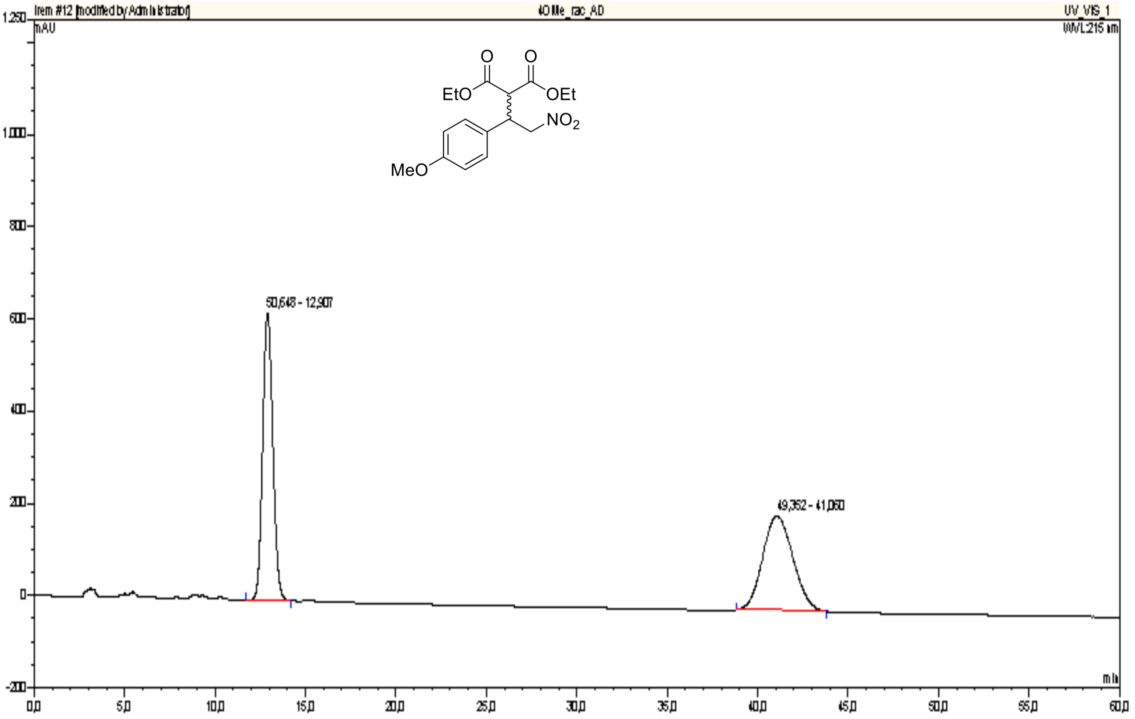

Figure S2.64| HPLC chromatogram of *rac*-**5j**

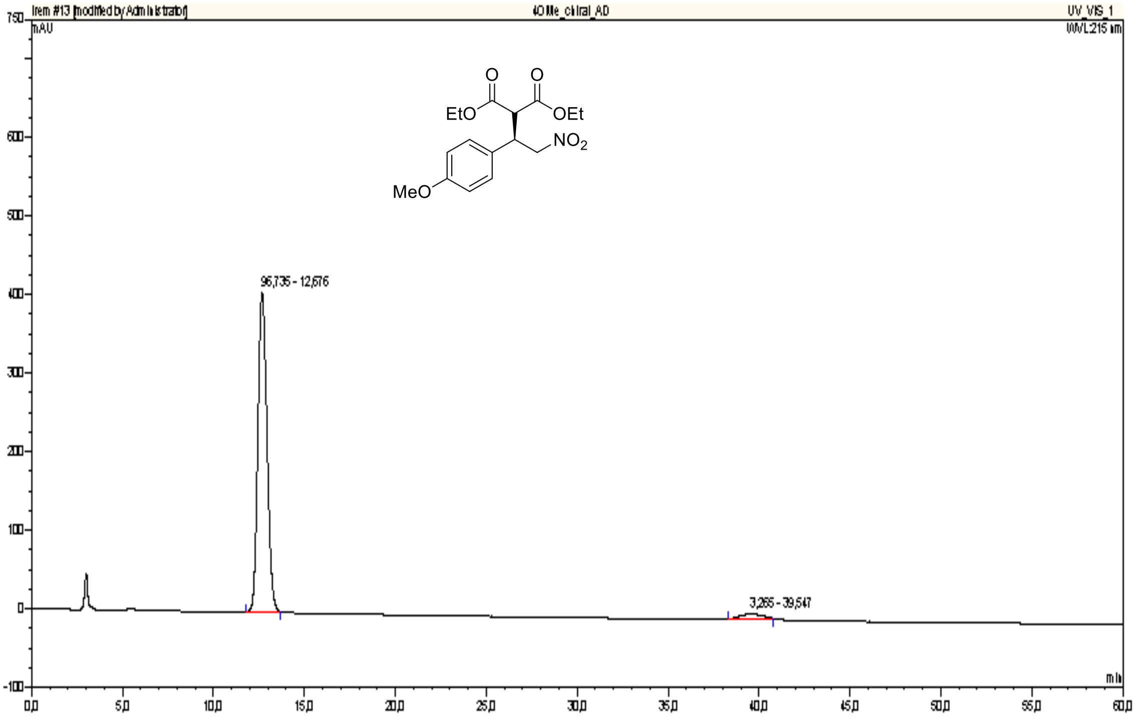

Figure S2.65 | HPLC chromatogram of *enantiomerically enriched adduct* **5j**

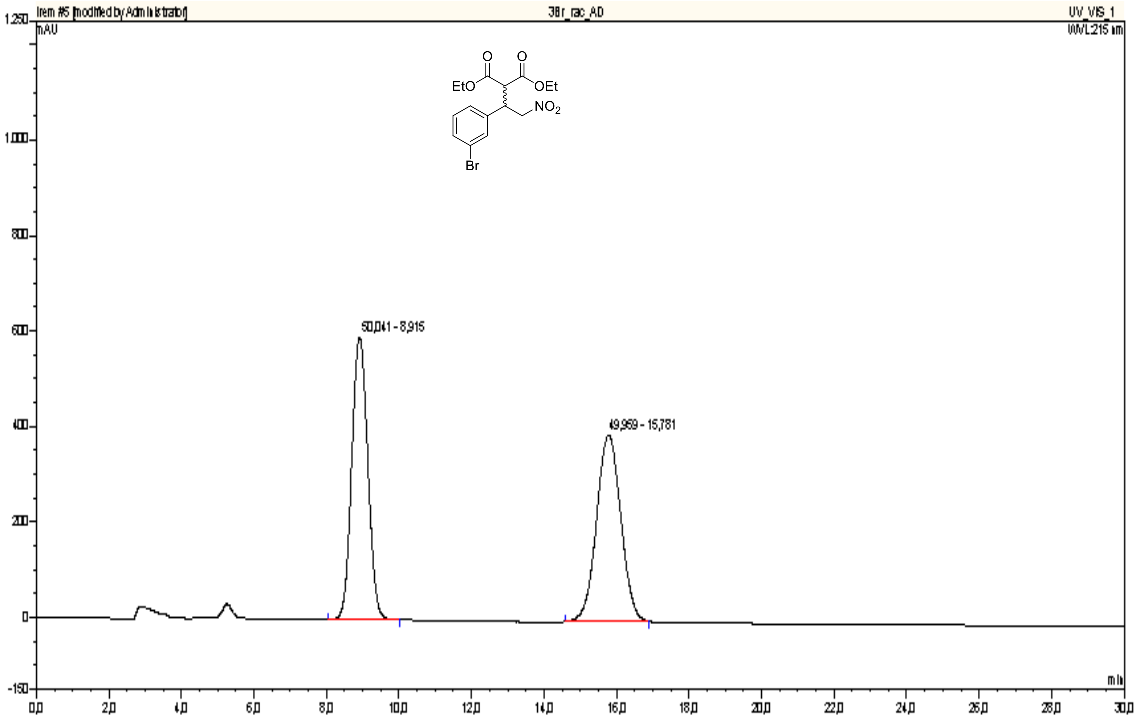

Figure S2.66 | HPLC chromatogram of *rac*-**5k**

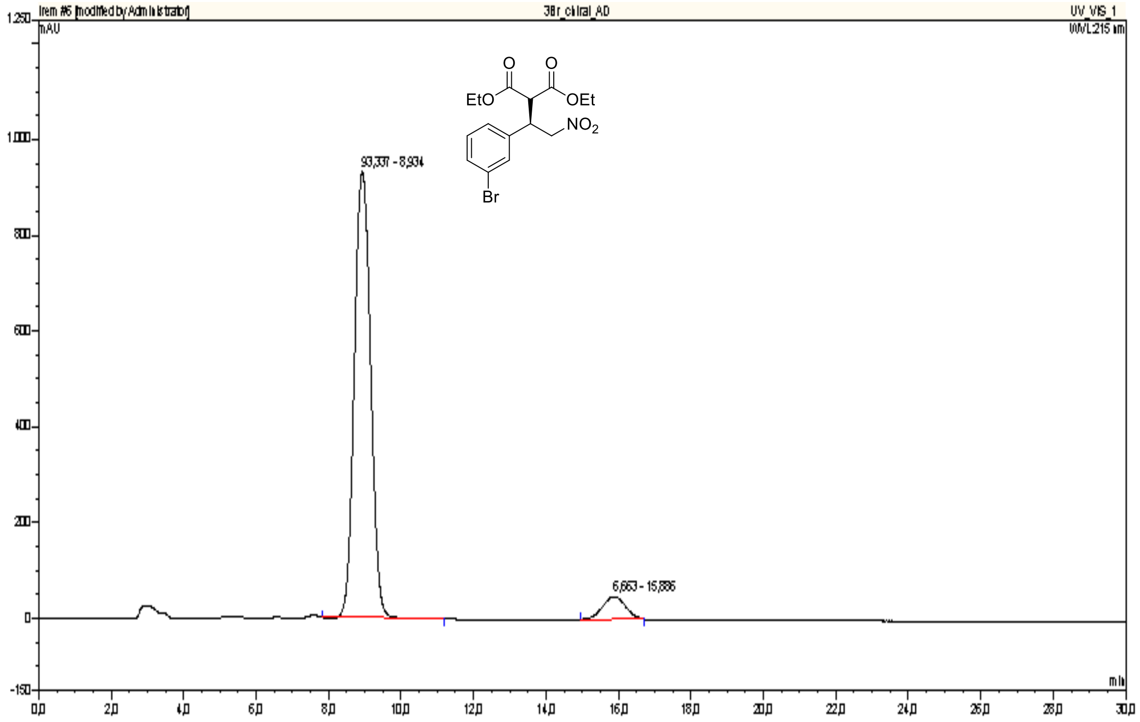

Figure S2.67 | HPLC chromatogram of *enantiomerically enriched adduct* **5k**

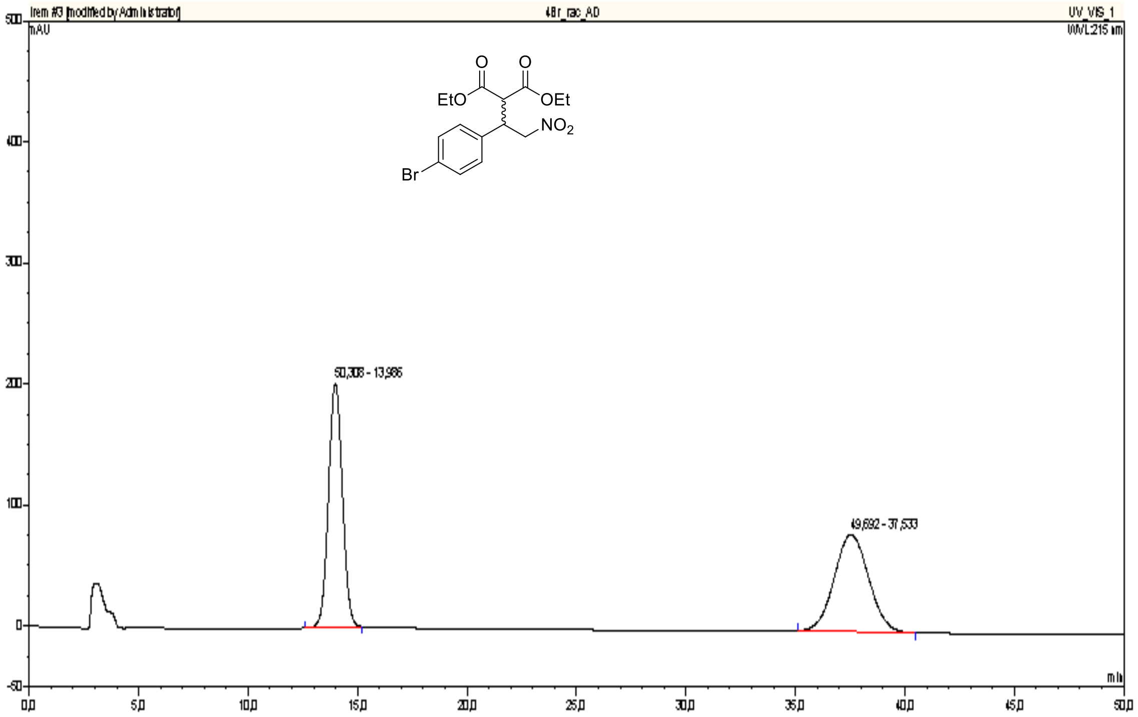

Figure S2.68 | HPLC chromatogram of *rac*-**5l**

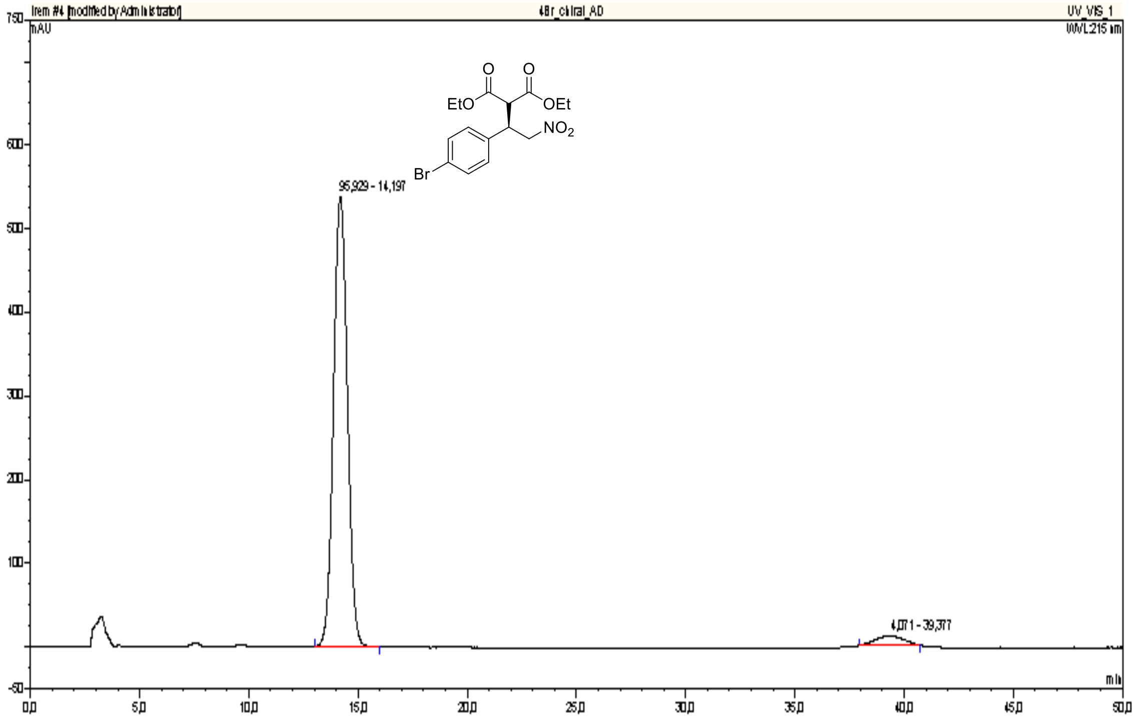

Figure S2.69 | HPLC chromatogram of *enantiomerically enriched adduct* **5l**

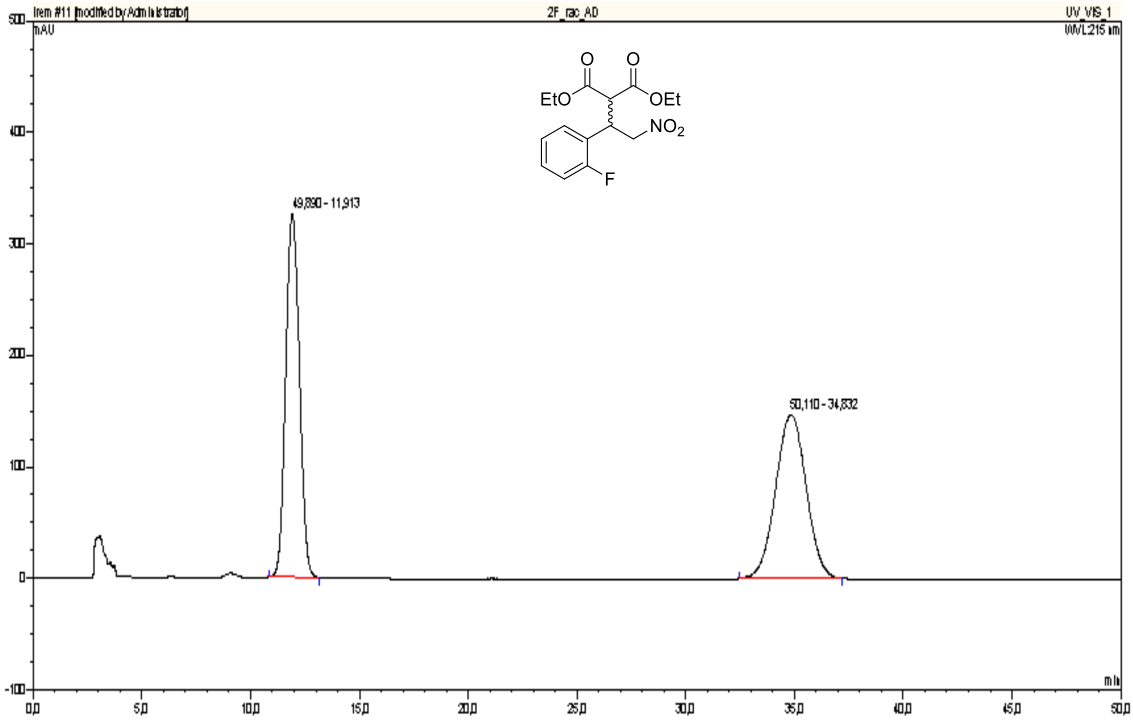

Figure S2.70 | HPLC chromatogram of *rac*-**5m**

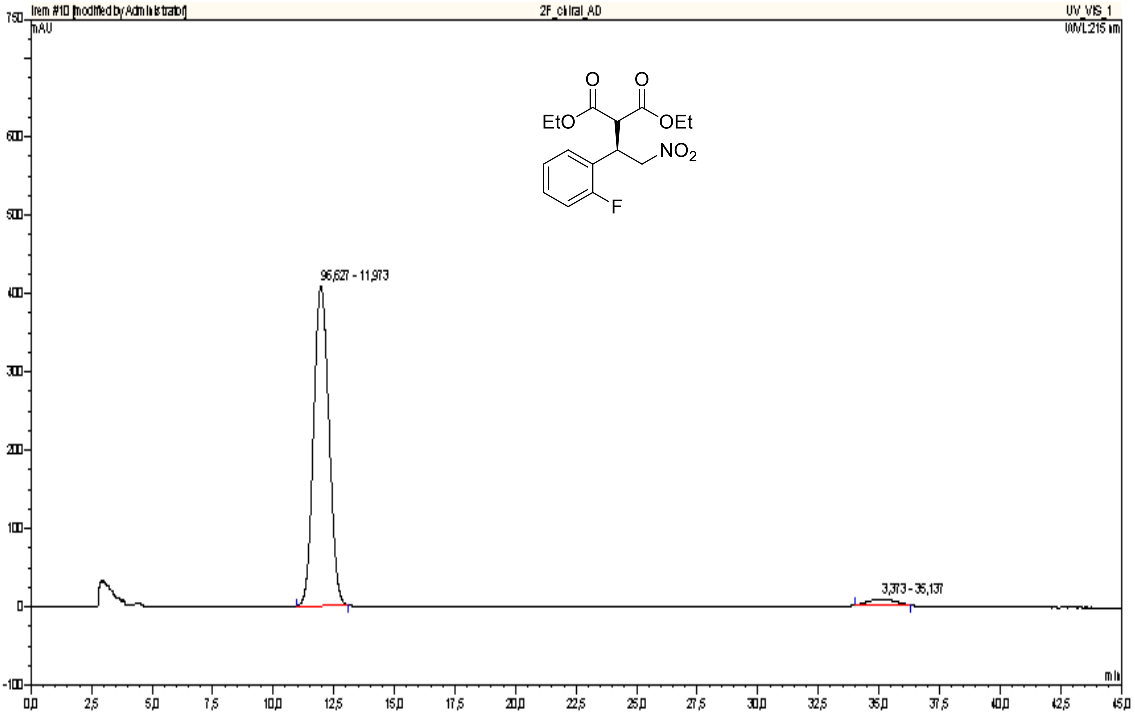

Figure S2.71 | HPLC chromatogram of *enantiomerically enriched adduct* **5m**

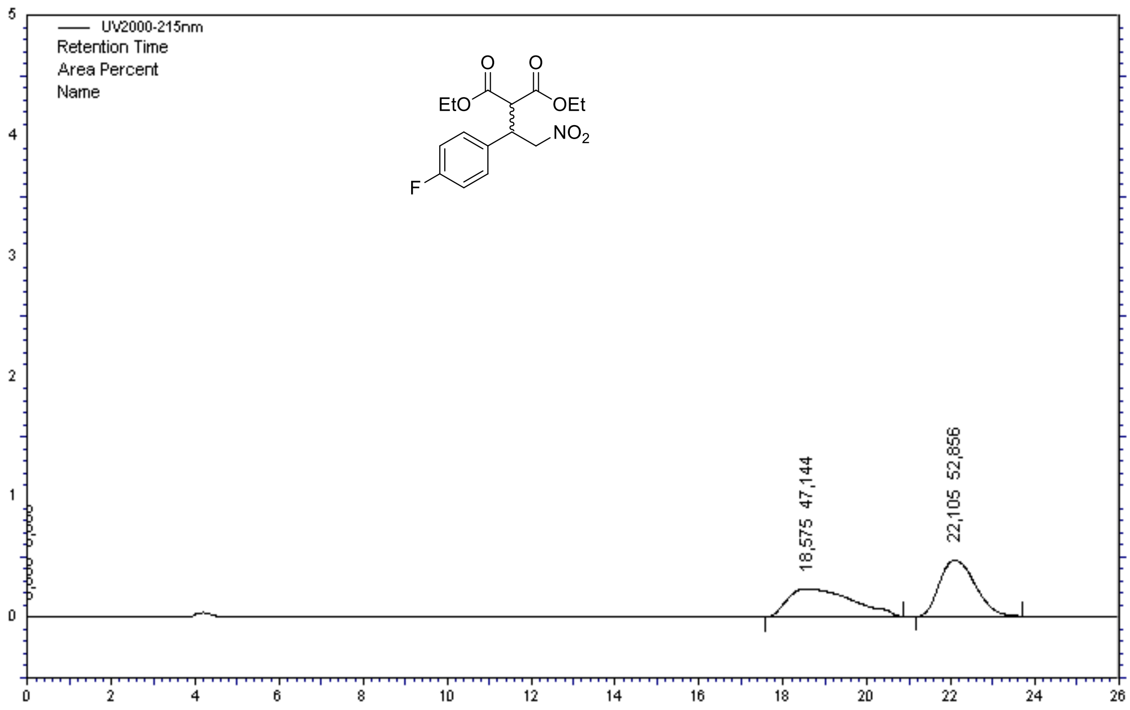

Figure S2.72 | HPLC chromatogram of *rac*-**5n**

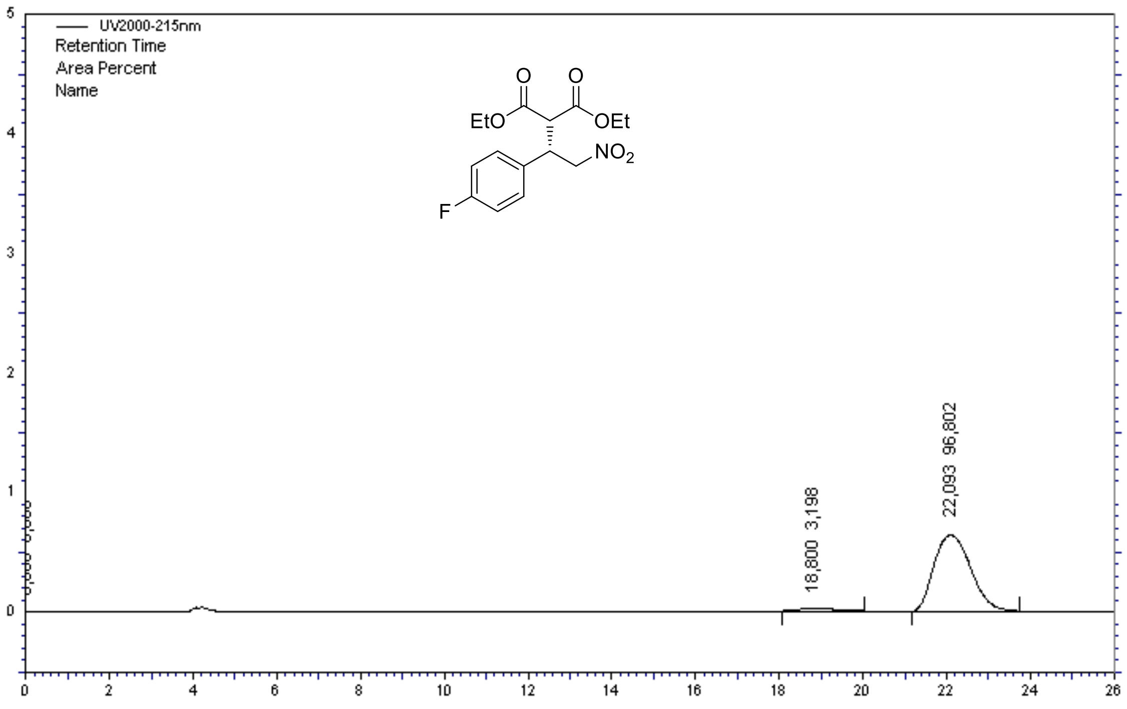

Figure S2.73 | HPLC chromatogram of *enantiomerically enriched adduct* **5n**

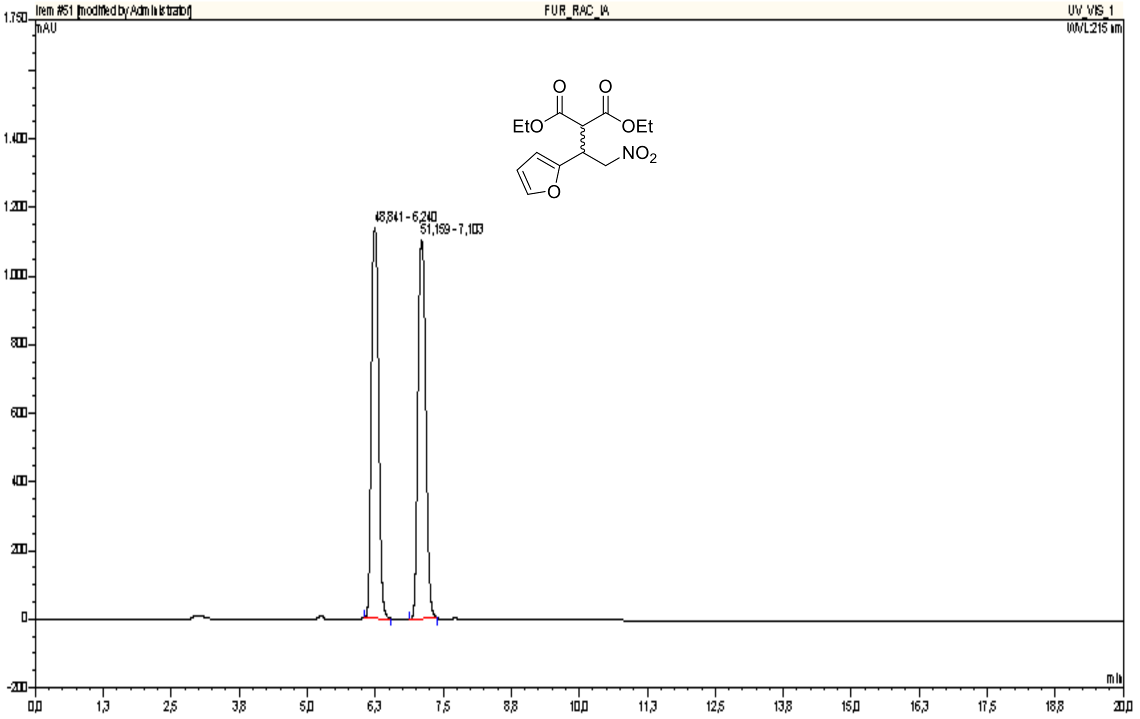

Figure S2.74 | HPLC chromatogram of *rac*-**5o**

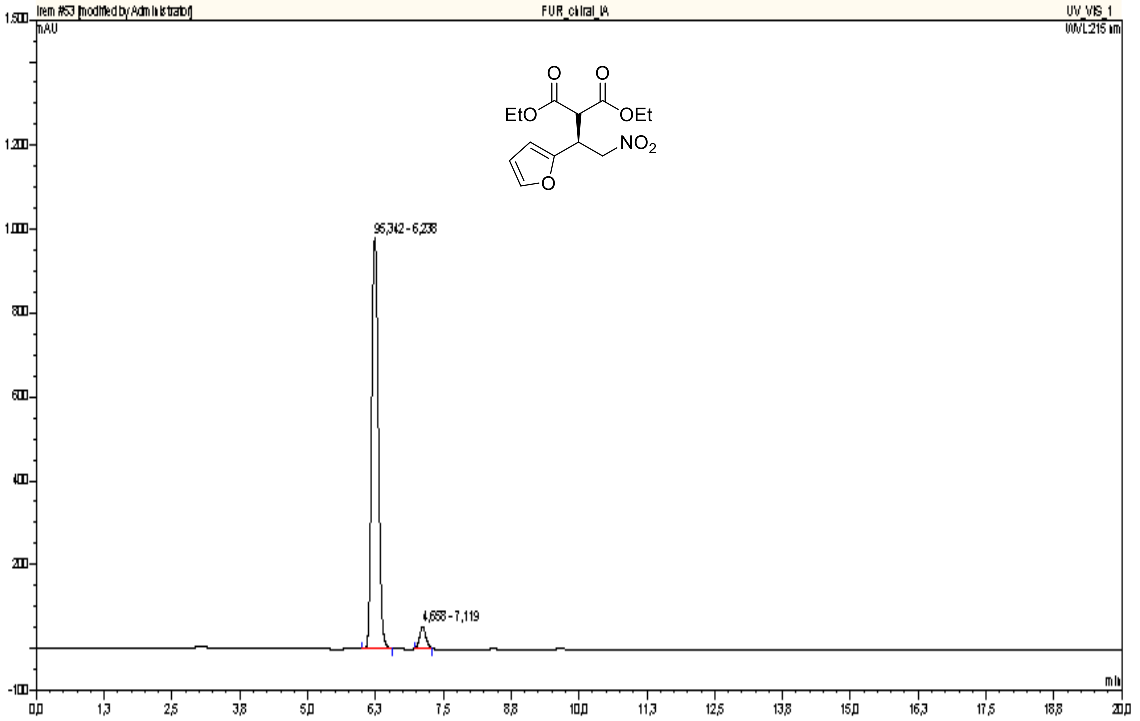

Figure S2.75 | HPLC chromatogram of *enantiomerically enriched adduct* **5o**

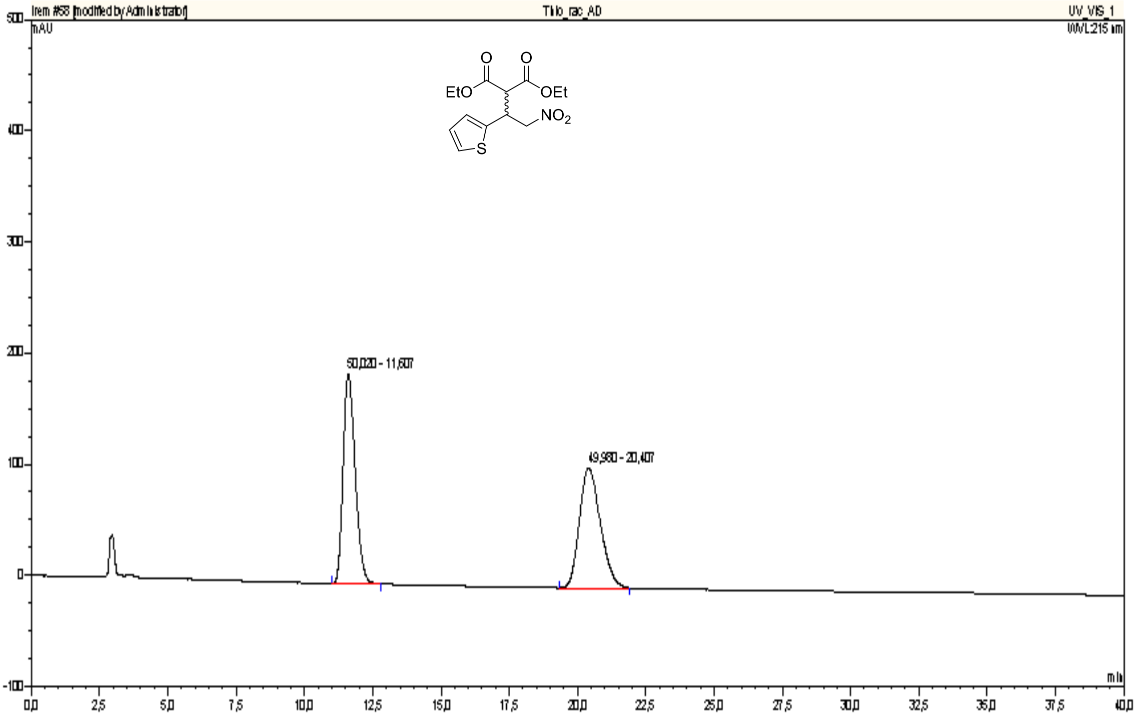

Figure S2.76 | HPLC chromatogram of *rac*-**5p**

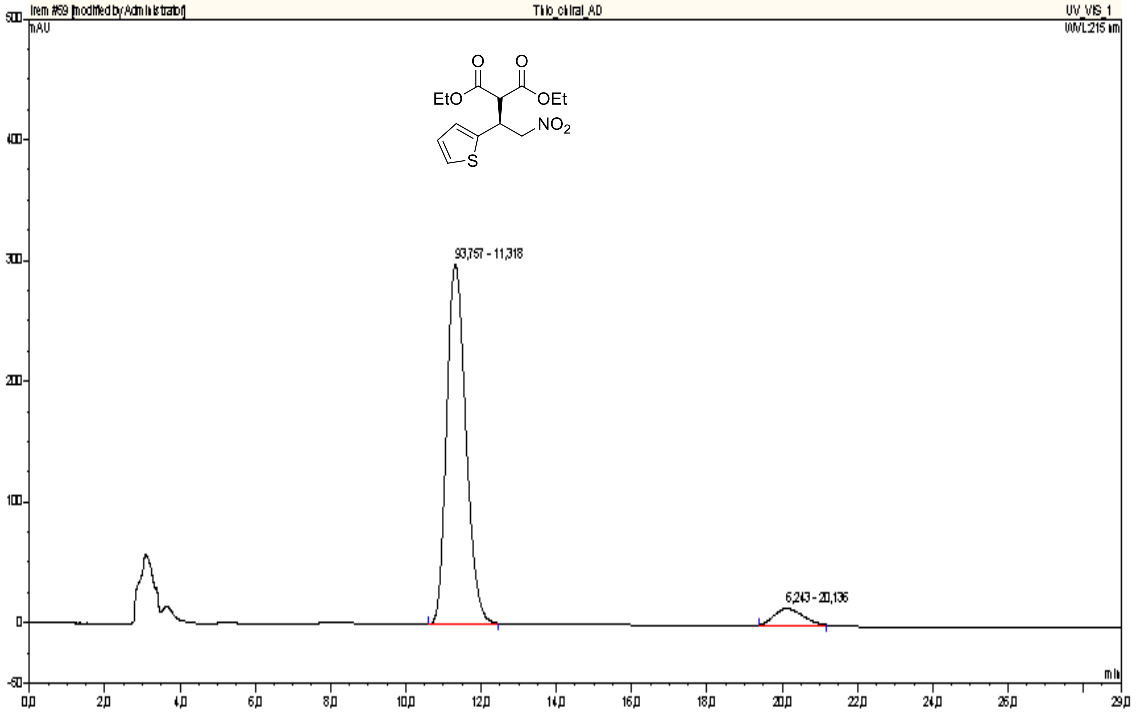

Figure S2.77 | HPLC chromatogram of *enantiomerically enriched adduct* **5p**

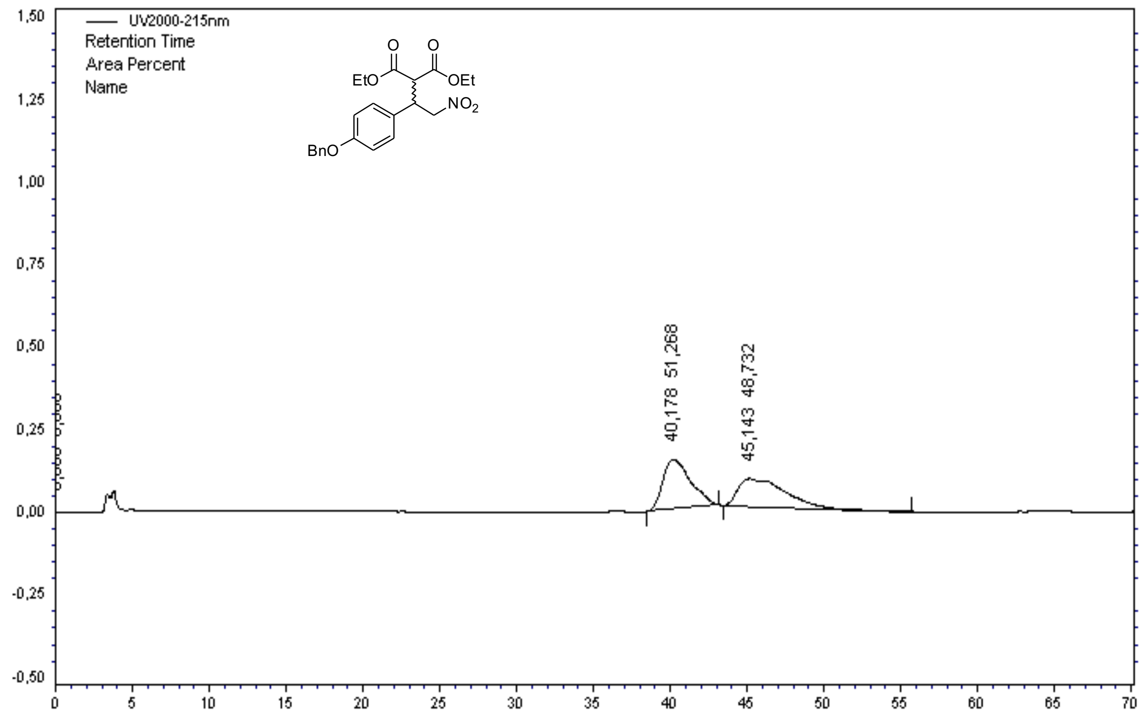

Figure S2.78 | HPLC chromatogram of *rac*-**5r**

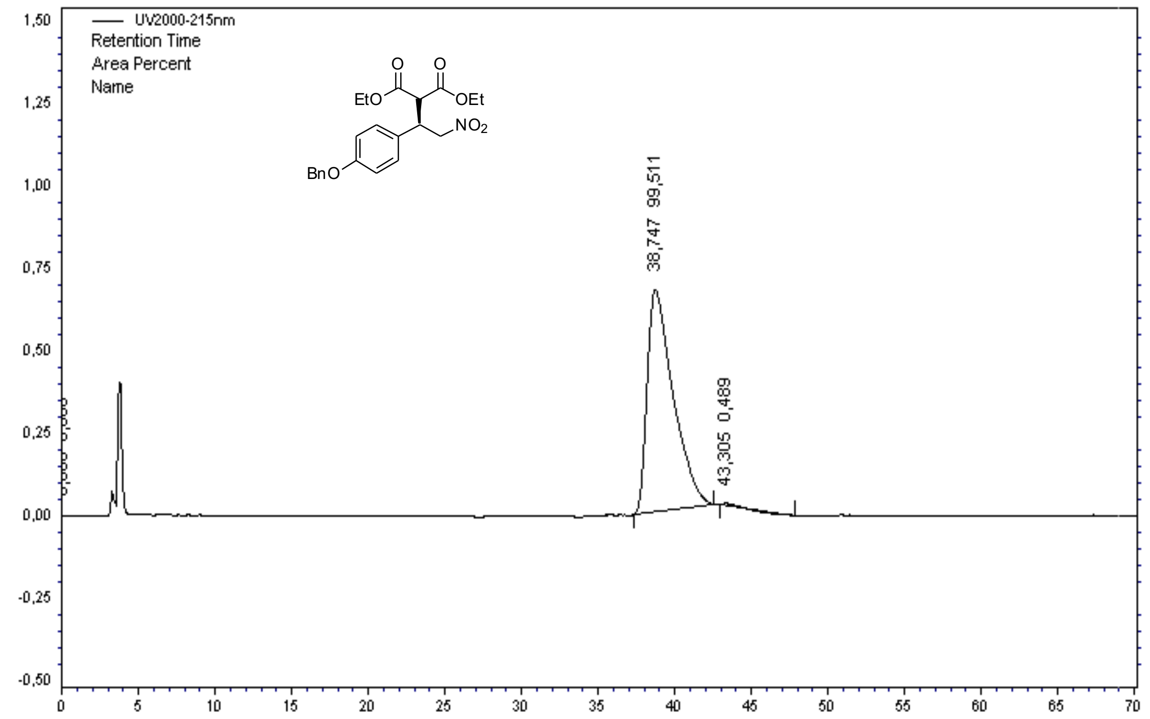

Figure S2.79 | HPLC chromatogram of *enantiomerically enriched adduct* **5r**

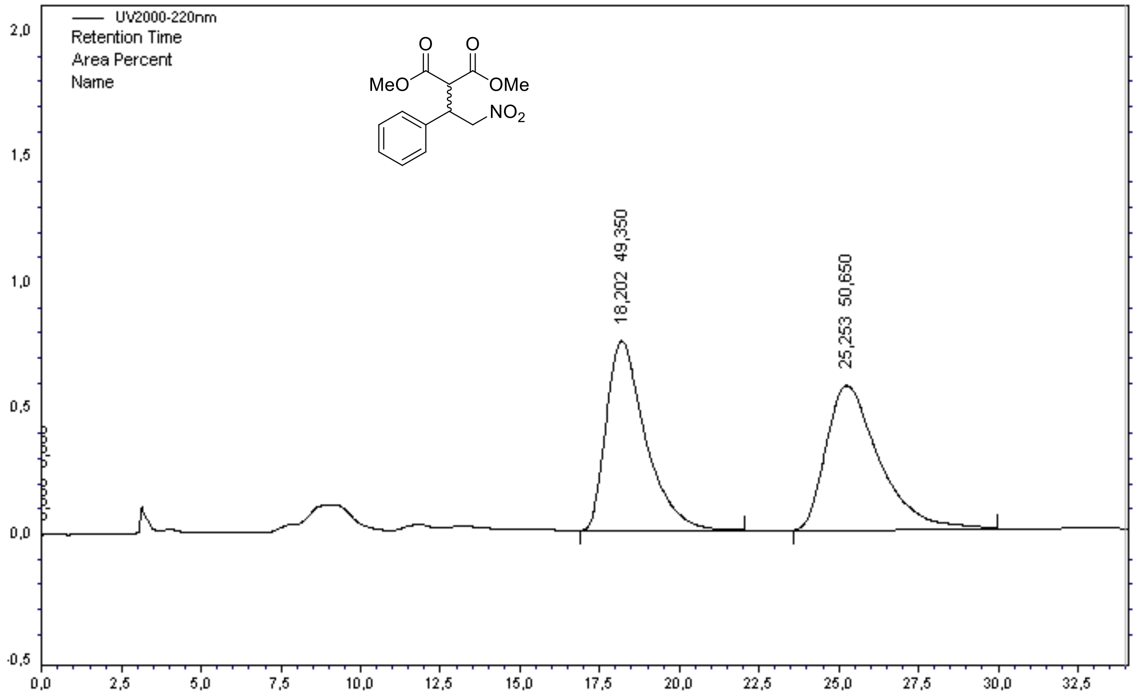

Figure S2.80 | HPLC chromatogram of *rac*-**5ab**

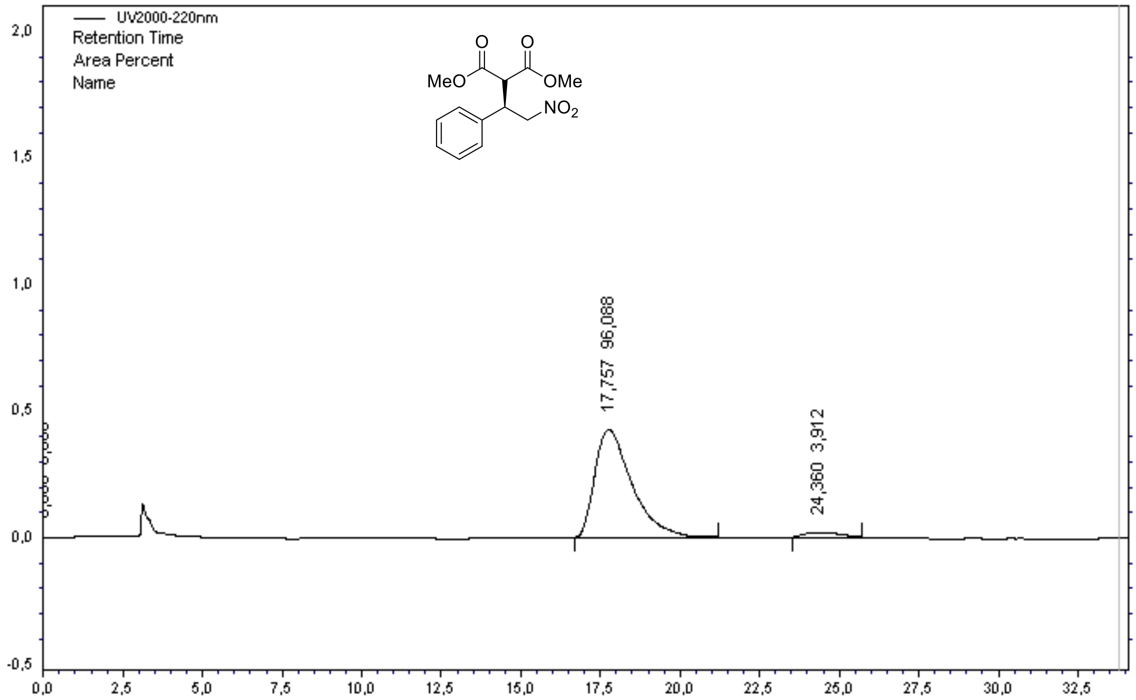

Figure S2.81 | HPLC chromatogram of *enantiomerically enriched adduct* **5ab**

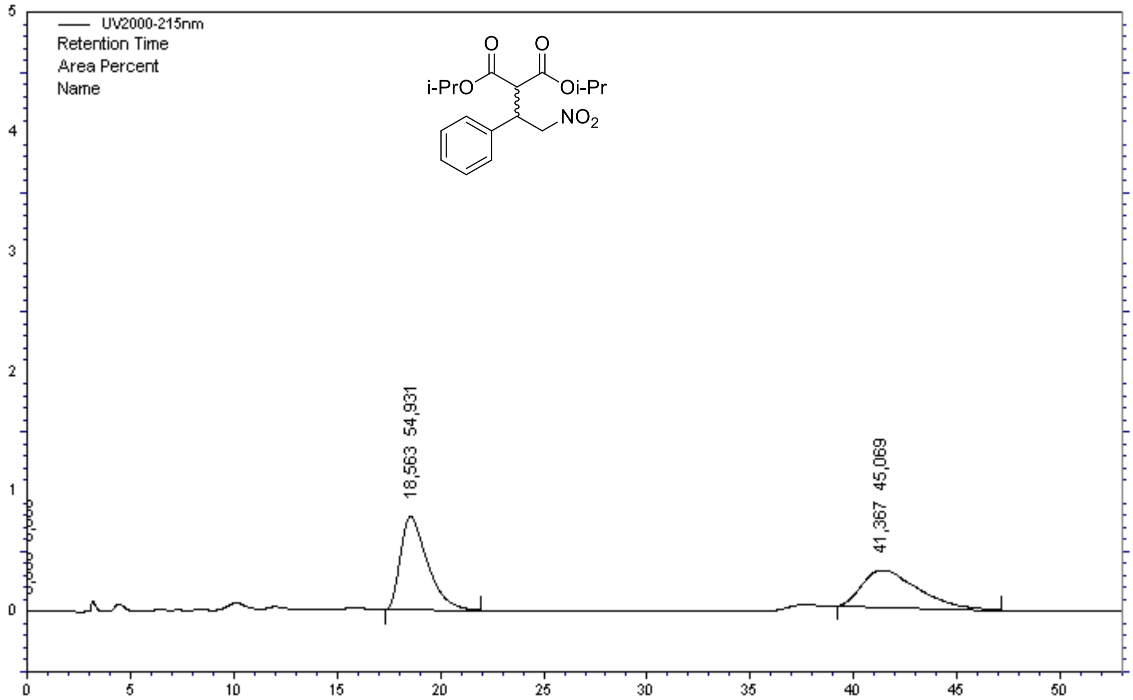

Figure S2.82 | HPLC chromatogram of *rac*-**5ac**

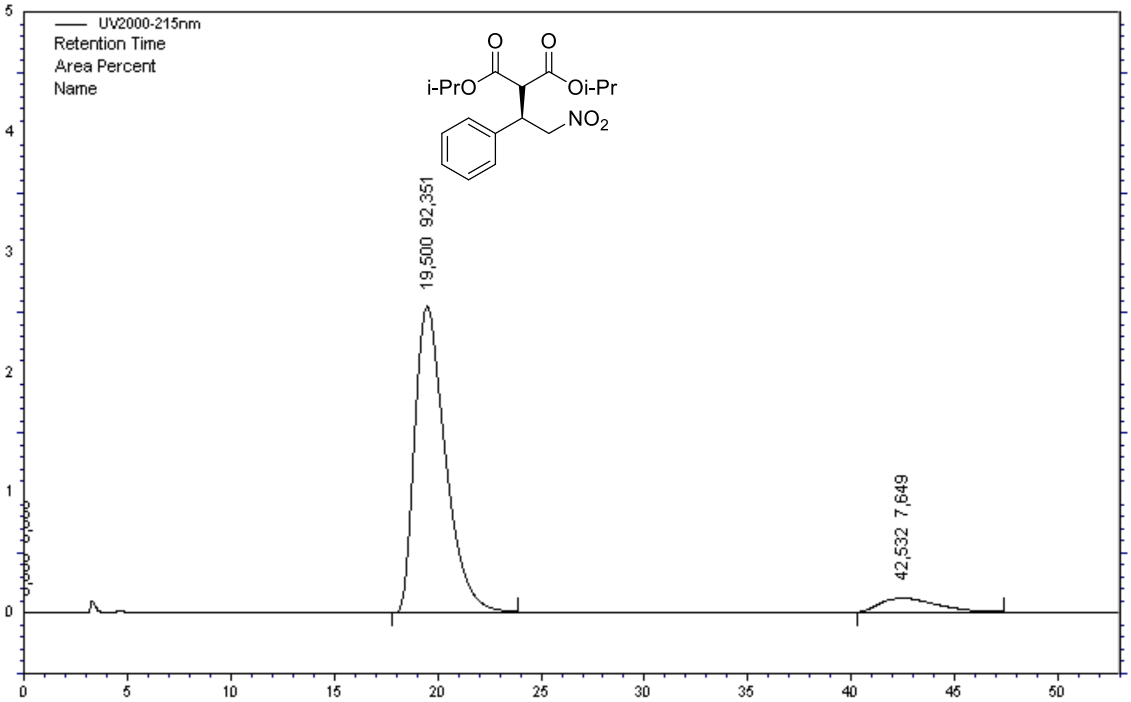

Figure S2.83 | HPLC chromatogram of *enantiomerically enriched adduct* **5ac**

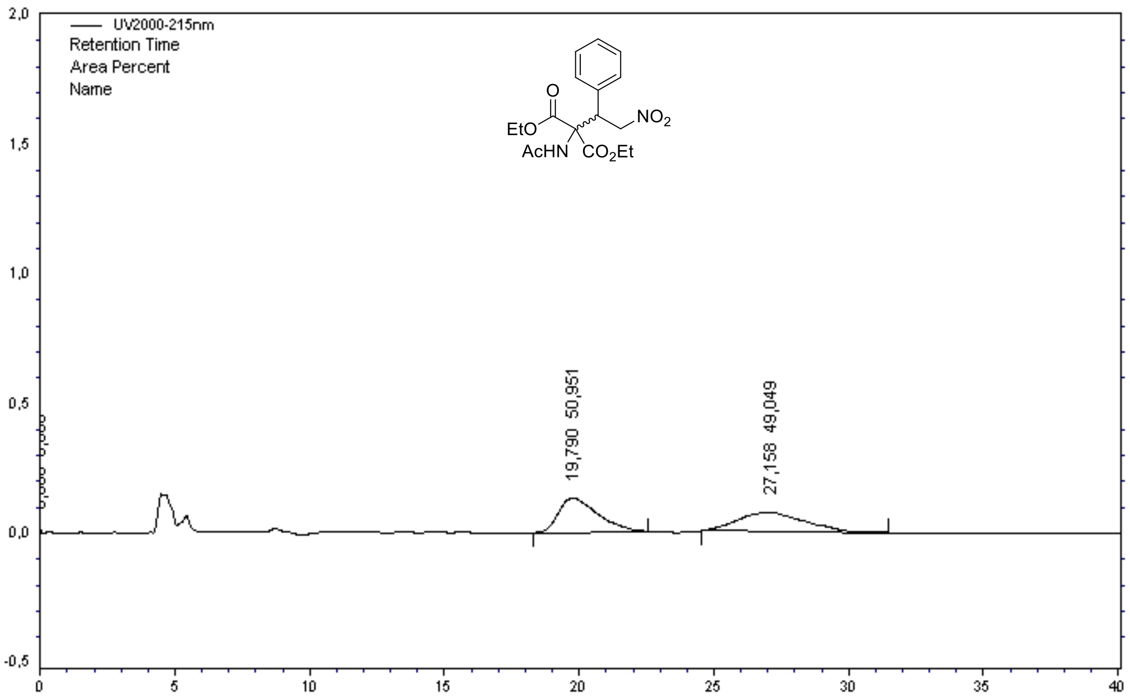

Figure S2.84 | HPLC chromatogram of *rac*-**5ad**

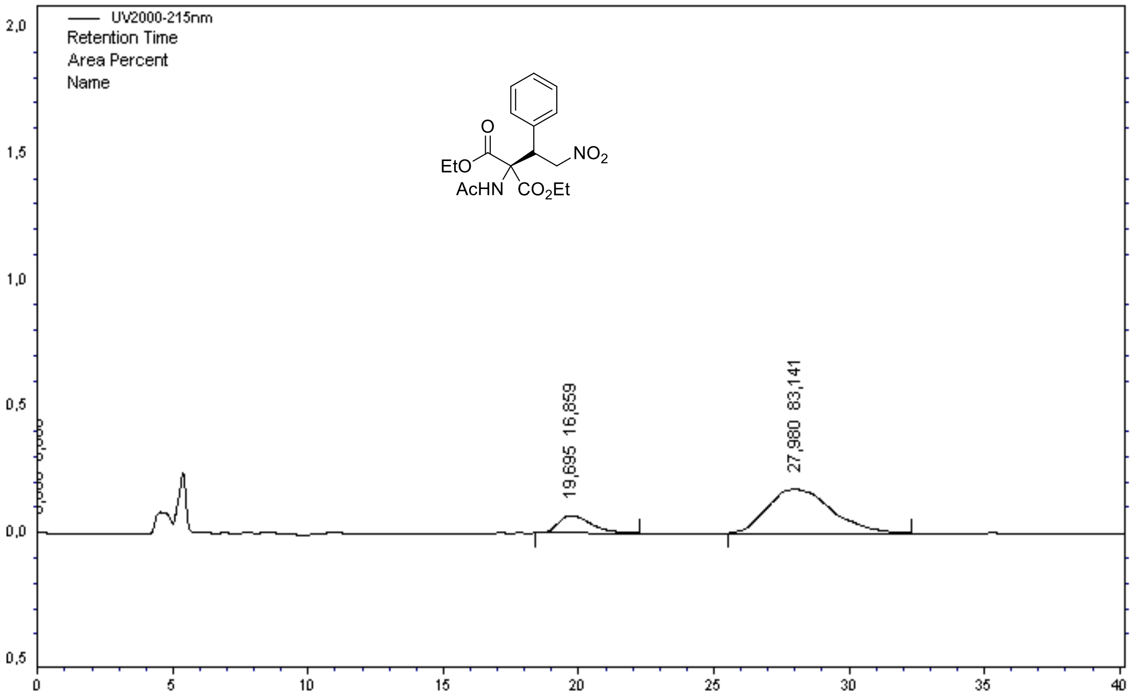

Figure S2.85 | HPLC chromatogram of *enantiomerically enriched adduct* **5ad**

2.7. REFERENCES

1. Isik, M.; Tanyeli, C. Cu-Catalyzed Selective Mono-N-pyridylation: Direct Access to 2-AminoDMAP/Sulfonamides as Bifunctional Organocatalysts. *J. Org. Chem.* **2013**, *78*, 1604−1611.
2. Evans, D. A.; Mito, S.; Seidel, D. Scope and Mechanism of Enantioselective Michael Additions of 1,3-Dicarbonyl Compounds to Nitroalkenes Catalyzed by Nickel(II)−Diamine Complexes. *J. Am. Chem. Soc.* **2007**, *129*, 11583–11592.
3. Andrés, J. M.; Manzano, R.; Pedrosa, R. Novel Bifunctional Chiral Urea and Thiourea Derivatives as Organocatalysts: Enantioselective Nitro-Michael Reaction of Malonates and Diketones. *Chem.—Eur. J.* **2008**, *14*, 5116–5119.
4. Almaşi, D.; Alonso, D. A.; Gomez-Bengoa, E.; Najera, C. Chiral 2- Aminobenzimidazoles as Recoverable Organocatalysts for the Addition of 1,3- Dicarbonyl Compounds to Nitroalkenes. *J. Org. Chem.* **2009**, *74*, 6163–6168.
5. Lee, M.; Zhang, L.; Park, Y.; Park, H. G. Chiral 2-aminobenzimidazole bifunctional organocatalysts: effect of di-CF3 and TFA on catalytic mechanisms. *Tetrahedron* **2012**, *68*, 1452–1459.
6. Okino, T.; Hoashi, Y.; Furukawa, T.; Xu, X.; Takemoto, Y. Enantio- and Diastereoselective Michael Reaction of 1,3-Dicarbonyl Compounds to Nitroolefins Catalyzed by a Bifunctional Thiourea. *J. Am. Chem. Soc.* **2005**, *127*, 119–125
